# A revision of the genus *Arenivaga* (Rehn) (Blattodea, Corydiidae), with descriptions of new species and key to the males of the genus

**DOI:** 10.3897/zookeys.384.6197

**Published:** 2014-02-26

**Authors:** Heidi Hopkins

**Affiliations:** 1 Division of Arthropods, Museum of Southwestern Biology, University of New Mexico, Albuquerque, NM, USA

**Keywords:** *Arenivaga*, Polyphagidae, Corydiidae, key, cockroach, sand cockroach, desert cockroach, new species

## Abstract

The cockroach genus *Arenivaga* is revised. Forty-eight *Arenivaga* species are recognized with nine previously known species and 39 described as new including the following: *A. pagana* **sp. n.**, *A. grandiscanyonensis* **sp. n.**, *A. haringtoni* **sp. n.**, *A. hopkinsorum* **sp. n.**, *A. umbratilis* **sp. n.**, *A. tenax* **sp. n.**, *A. impensa* **sp. n.**, *A. trypheros* **sp. n.**, *A. darwini* **sp. n.**, *A. nalepae* **sp. n.**, *A. sequoia* **sp. n.**, *A. mckittrickae* **sp. n.**, *A. gaiophanes* **sp. n.**, *A. belli* **sp. n.**, *A. estelleae* **sp. n.**, *A. delicata* **sp. n.**, *A. mortisvallisensis* **sp. n.**, *A. milleri* **sp. n.**, *A. pratchetti* **sp. n.**, *A. gumperzae* **sp. n.**, *A. rothi* **sp. n.**, *A. ricei* **sp. n.**, *A. adamsi* **sp. n.**, *A. nicklei* **sp. n.**, *A. akanthikos* **sp. n.**, *A. moctezuma* **sp. n.**, *A. paradoxa* **sp. n.**, *A. apaeninsula* **sp. n.**, *A. hebardi* **sp. n.**, *A. dnopheros* **sp. n.**, *A. aquila* **sp. n.**, *A. florilega* **sp. n.**, *A. galeana* **sp. n.**, *A. gurneyi* **sp. n.**, *A. pumila* **sp. n.**, *A. hypogaios* **sp. n.**, *A. diaphana* **sp. n.**, *A. nocturna* **sp. n.**, *A. alichenas* **sp. n.** All species are described or redescribed, major morphological features are illustrated, distributions are characterized, and the biology of the species is reviewed. A neotype series is designated for *A. investigata* Friauf & Edney.

## Introduction

The genus *Arenivaga* has not been revised in nearly a century (Hebard 1920). In that time only one new species, *Arenivaga investigata* (Friauf and Edney 1969), has been described. While revisions of this genus have been started twice since 1970, nothing has been completed or published. This represents a rather large taxonomic oversight as *Arenivaga* now proves to be the most species-rich genus of native cockroaches in the United States. How have these species-rich and extraordinary animals been overlooked for so long? The reasons include the historically poor funding of systematics of “minor” insect orders, the unfortunate and inaccurate reputation held by cockroaches as disgusting animals and therefore generally not “sexy” to study, and the knowledge, among interested parties, of how very difficult *Arenivaga* are taxonomically (Hebard 1920). Even though there has been no revisionary work on the group in 93 years, *Arenivaga* has been the subject of several physiological and ecological studies (Walthall and Hartman 1981; Edney 1968; Hawke and Farley 1971a, 1971b, 1973; Cohen and Cohen 1976, 1981; Edney and McFarlane 1974; Hartman et al. 1987; Edney et al. 1974, 1978; Jackson 1983; O’Donnell 1977; 1981, 1982; Appel et al. 1983). This work has scratched the surface of the phenology and physiology of these animals and revealed a little about the amazing adaptations that allow them to succeed in some of the harshest environments on earth. Here I provide a full revision of the genus, including redescriptions of the genus and its nine known species, descriptions of 39 new species, a key to the adult males, and distribution maps for each species. Novel morphological characters are also described. A total evidence phylogenetic hypothesis and biogeographic analysis of the genus, as well as an examination of the females of the genus, will be provided in separate publications.

**Figure 1.**
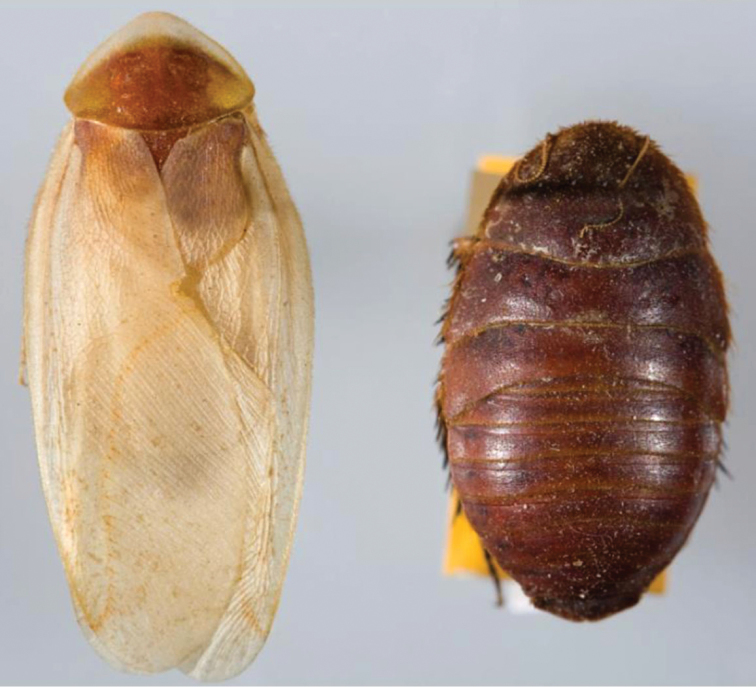
Sexual dimorphism in *Arenivaga*.

“…*we can state definitely that the present genus (Arenivaga) is the most difficult genera of Blattidae found in this country*” (Hebard 1920, p. 201). Having spent more than four years studying the genus, the reasons this group is so complex and challenging to understand includes: *Arenivaga* are nocturnal and subterranean making them particularly difficult to find in the field; once in collections the specimens are extremely fragile making them a challenge to handle in a laboratory setting; their external morphology provides very few diagnostic characters making species identification in most cases impossible without genitalic dissection and clearing; many species exhibit a labile morphological response to their environment making the phenotype within a species highly variable; the genus is strikingly sexually dimorphic (Figure 1) making morphological association of females with males within a species all but impossible; there is one molecular study that included *Arenivaga* (Djernaes et al. 2012) resulting in three *Arenivaga* sequences on GenBank making the DNA analysis of this genus a near complete black box from primers to protocols; finally, there is no prior phylogenetic work on the genus.

## Materials and methods

### Material examined

This revision is based on examination of more than 5200 adult male specimens. Often cockroaches in natural history collections are not identified to genus, or even to family. This problem is exacerbated by unevenly used classifications of Blattodea, which are not generally based in modern phylogenetic hypotheses, a problem being worked on currently (Djernaes et al. 2012; Beccaloni and Eggleton 2013). United States specimens are better represented in this monograph than Mexican specimens. Mexico represents a very diverse region for *Arenivaga*, but is less well-collected for the group than the US. Primary types were borrowed to aide in redescription of the nine described species; at this time it was discovered that both the holotype and all paratypes of *Arenivaga investigata* had been destroyed by dermestids, leaving only the pins and labels behind. A neotype series is therefore designated herein. Specimens were examined from the following collections:

AMNH American Museum of Natural History

ANSP Academy of Natural Science, Philadelphia

ASUT Arizona State University, Tempe

MLBM Monte L. Bean Life Science Museum, Brigham Young University

CAS California Academy of Science

CSCA California State Collection of Arthropods

CSLB California State University, Long Beach

J Cole Jeff Cole private collection

CUIC Cornell University Insect Collection

EMEC Essig Museum of Entomology, California

FSCA Florida State Collection of Arthropods

HEH Heidi Hopkins private collection

IMNH Idaho Museum of Natural History

LACM Los Angeles County Museum

MCZ Museum of Comparative Zoology, Harvard

MSB Museum of Southwestern Biology, Albuquerque

NAUF Northern Arizona University, Flagstaff

NVDA Nevada Department of Agriculture

OMNH University of Oklahoma

OSEC Oklahoma State University

OSUC Ohio State University Collection

PMNH Peabody Museum of Natural History, Yale

SDMC San Diego Natural History Museum

SEMC University of Kansas Snow Entomological Museum Collection

TAMU Texas A&M University

UAIC University of Arizona Insect Collection

UCMC University of Colorado Museum Collection

UCRC University of California, Riverside

UMMZ University of Michigan Museum of Zoology

USNM National Museum of Natural History, Smithsonian Institution

WB Warner Bill Warner private collection

Two prior researchers at the Smithsonian, Drs. Nickle and Gurney, had begun a revision of *Arenivaga* in the early 1970s. While that revision was in progress, one of the researchers passed away and the other went on to other projects; the revision was never completed and the loans were never returned. The Smithsonian assigned those loans to me if I would take on the paperwork challenge of contacting all the loaning institutions to transfer the original loans or issue me new loan papers. Most collections were excited to hear about the specimens that had been “on loan” for more than forty years, and more than happy to issue new loans with updated specimen counts. The ownership of a small minority of specimens (31) could not be determined and those are now housed in my personal collection.

## Data resources

The data underpinning the analyses reported in this paper are deposited in the Global Biodiversity Information Facility, http://ipt.pensoft.net/ipt/resource.do?r=arenivaga_locality_data.

## Taxonomic research in Blattodea and *Arenivaga*

### Taxonomic research in Blattodea

Blattodea are a sadly understudied order of insects, but the field has benefitted greatly from a few dedicated researchers, four of whom I will mention here to create the setting in which my work was done. A masterwork of cockroach morphology (and family-level taxonomy until a very few years ago) is *Evolutionary Studies of Cockroaches* by Francis McKittrick (McKittrick 1964). This beautiful work, her PhD dissertation, unfortunately provides the poorest coverage of the Corydiidae (formerly Polyphagidae) (Beccaloni and Eggleton 2011). There is one drawing of the male genitalia of *Arenivaga bolliana* from a dorsal aspect, which is not useful for diagnosis in *Arenivaga*. *Evolutionary Studies of Cockroaches* is a family-level work and not designed for diagnosis of individual species; therefore, I do not use McKittrick’s genitallic terms or abbreviations in this work, although Table 1 provides an equivalency of terms between her work and mine. Louis Roth described hundreds of new species over the course of his career but his families of specialization were Blattidae and Blattellidae and therefore also provide no reference for my work. Grandcolas (1996) is also a family-level work and has little application in my study. Table 1 provides terminological equivalency between his work and mine. Klaus-Dieter Klass produced a monumental work on cockroach genitalia (Klass 1997). This is an order-level work that examines genitalia of Blattodea and Mantodea by family. It has more coverage of Corydiidae than McKittrick’s work but all drawings are again from a dorsal aspect and are therefore not helpful in this work. Corydiidae have received little attention at the subfamily and generic levels, and none at all in nearly a century. I therefore turned to the previous work on *Arenivaga* to serve as my guide in my research (see Taxonomic History below).

**Table 1.** Terms used to describe genital phallomeres in this study and those of McKittrick and Grandcolas. Note that the sclerites of the left phallomere are not diagnostic in *Arenivaga* so only one term is needed, and that the small central sclerite, not recognized by McKittrick, and designated as N (neoformation) by Grandcolas, is diagnostic in *Arenivaga*.

This Study	McKittrick (1964) (Fig. 110)	Grandcolas (1996) (Figs 2 and 3)
Left Phallomere	L1, L2, L4	L1, L2d, L3v, L2v
Genital Hook	L3	L3d, L2d
Right Dorsal Phallomere	R3	R3d
Right Ventral Phallomere	R1, R2	R2
Small Central Sclerite	---	N

In the interests of clarity and to create greater user-friendliness I am using full language terms for the genital phallomeres as opposed to the abbreviations favored by previous authors. For example, the genital hook is a common term used in describing the genital morphology of Blattodea, but in some families it is not particularly hooklike, and the term has been reduced to a numbered sclerite of the left phallomere (see Table 1 “Genital Hook”). In Corydiidae the genital hook is distinct, hooklike, and frequently diagnostic. For this reason I believe it is easier for the reader to understand the text and use the key if they do not have to look up which sclerite is, for example, L3d. I use standard terms indicating direction within the animal (left, right, ventral, dorsal) and provide a diagram of same to assist the reader in navigating the genitalia (see Figure 7).

### Taxonomic research in Arenivaga

**Figure 2.**
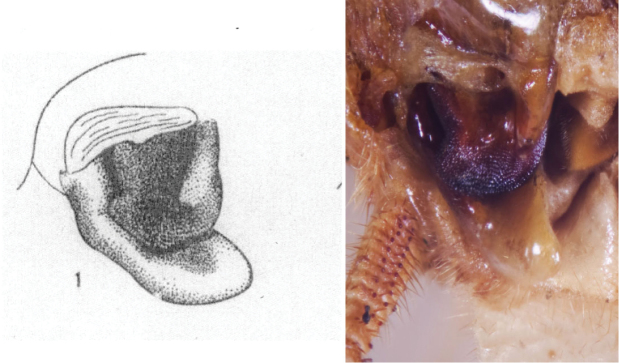
Hebard’s drawing of genitalia of *Arenivaga bolliana* next to a photograph of same (Hebard 1920). (Drawing 1, Plate VII from Hebard 1920, used with permission of the Transactions of the American Entomological Society).

**Figure 3.**
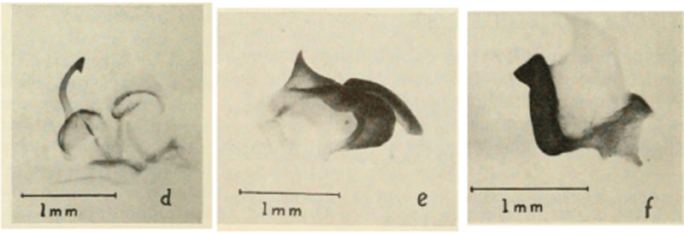
Photographs of the dissected and cleared genitalia provided when *Arenivaga investigata* was described (Friauf and Edney 1969). Note the extensive architecture that would be invisible if the phallomeres were left *in situ*. (Portions of Figure 1, Friauf and Edney 1969, used with permission of the Proceedings of the Entomological Society of Washington).

Other than the last species of *Arenivaga* described in 1969, (*Arenivaga investigata* Friauf and Edney 1969) no extant species had been determined by complete genitalic dissection and clearing, but rather by removal of the subgenital plate and examination and drawing *in situ* of the portions of the genital phallomeres so revealed (Figure 2). But when I attempted to remove subgenital plates from pinned specimens they could not be removed without damage to the plate or the specimen. I rehydrated specimens to make removal of the subgenital plate easier and to reduce damage, but in this molecular age I did not want to be adding water in any quantity to a specimen as this is known to degrade DNA and I had no way of knowing which specimens could prove vital to my future molecular analysis. Also, when I compared the completely dissected and cleared genitalia of *Arenivaga investigata* (Figure 3) to the *in situ* genitalia of the other eight species, I realized that there was considerable detail to the phallomeres on all sides that could not be seen from a static view of *in situ* genitalia. I therefore determined that *in situ* examination of *Arenivaga* genitalia would be insufficient to make species designations in most cases and it would be necessary to fully dissect and clear the holotype genitalia to ascertain true species designations. That was done, photographs of same were taken, and then drawings made from the photographs, which drawings appear in this monograph. Upon revision, the original nine species of *Arenivaga* all stand as good species.

## Methods and techniques

### Dissection of genitalia

**Figure 4.**
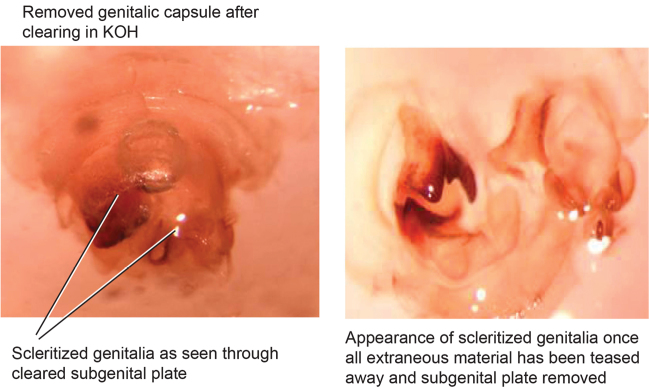
Photographs of cleared genitalia before and after unwanted material has been teased away.

Genitalia were dissected from dry specimens using a pair of microscissors to remove the entire genitalic capsule, taking care since dry specimens are very fragile. Specimens can be softened while preserving DNA by placing them for a short time in 95% ethanol after which the capsule may be cut off with less chance of damage to the specimen and less likelihood of the capsule being accidentally lost. Dissected capsules were then placed in 10% KOH solution at room temperature for three to five days to clear. There is considerable difference between specimens in the density of sclerotization of the phallomeres, so capsules in KOH were checked beginning on the third day and then returned to solution if desired clearing was not achieved. Once cleared, the capsule was removed from the KOH solution, rinsed in DI water, and placed on a slide or watch glass. Using microdissection tools and a dissecting scope the subgenital plate was detached on one side and opened like a flap. Microtools were then used to carefully dissect away and remove sclerites other than the sub and supra genital plates, the digestive tract and rectum, muscle tissue, and other non-sclerotized material in the genitalic capsule. Remaining structures were the supra and sub genital plates, the cerci, the sclerotized genital phallomeres and genital hook (Fig. 4). Cleared genitalia were stored in a few drops of glycerin in open glass dishes pinned next to the appropriate specimen until thoroughly examined and illustrated. Then they were placed in glycerin in genitalia vials mounted on the specimen pin.

On specimens dissected by previous worker the subgenital plate was left as a flap on the whole dry specimen or glued to a card attached to the pin. In both instances the subgenital plate is frequently broken off and lost or damaged. In some cases, when the genitalia were completely dissected the phallomeres were glued to a card and the card attached to the pin. These too are often broken or lost from the card. Therefore, I recurated into vials all genitalia I examined that were previously attached to a card on the specimen pin. This will provide more secure storage and preservation of vital structures.

### Cleaning specimens

Many *Arenivaga* specimens are covered in dust or sand from their subterranean lifestyle, or moth scales from being captured in black light traps with Lepidoptera. They often have ant heads attached to an appendage and some specimens harbor mites. Additionally, *Arenivaga* specimens are often greasy. Although dirty, these specimens are also too fragile to be easily thoroughly cleaned. In order to avoid damage to specimens I only cleaned holotypes, (the only specimens being photographed in most instances), and even then only cleaned the specimens to the extent that it was safe to do so.

### Measurements

Measurements are provided to show the range of size within a species. The Total Length (TL), Greatest Width (GW), whole body ratio (TL/GW), Pronotal Length (PL), Pronotal Width (PW), pronotal ratio (PL/PW), maximum distance between the eyes (EW), and maximum distance between the ocelli (OW) are given for the holotype. The maximum and minimum measurements of TL, GW, PL, and PW are provided for the entire species. All measurements were made with a Mitutoyo digital caliper or with a hand held micrometer used in conjunction with a Leica WILD M3C dissection microscope.

### Descriptions

Descriptions in this project focus on adult males, currently the only *Arenivaga* life stage identifiable to species. In many cases, genitalic dissection and clearing are the only way to make a reliable species identification. For each species, the diagnostic genitalic feature(s) are indicated with arrow(s) on the drawing of the genitalia. Descriptions are based on the holotype (with the exception of the redescriptions of *Arenivaga erratica*, *Arenivaga grata*, *Arenivaga rehni*, and *Arenivaga tonkawa*) which could not be located in a timely manner due to extensive renovations taking place at the holding institution (ANSP). Many species of *Arenivaga* are variable in external phenotype, including size and, especially, color; such variation is included in each description.

### Photographs and drawings

The dorsal and ventral habitus of all holotypes were photographed, as well as the pronotum and the anterior surface of the cranium (the face). In general, the best-preserved specimen was used for photography. All photographs were taken with a Visionary Digital BK+ light imaging system (www.visionarydigital.com, R. Larimer).

The genitalia of *Arenivaga* are complexly three-dimensional *in situ*. Genitalia of all species were drawn using six pencil hardnesses, one white pencil and paper stumps. The drawings show the various phallomeres, and diagnostic characters are indicated with arrows. All genitalia have been drawn in ventral aspect following dissection.

### Distributions

All distribution information is based on males. The type locality is provided for all species as well as a list of the label data for all specimens examined. Any illegible label data is indicated by question marks. My comments regarding label data are placed in brackets. Distribution maps are based on label data. Only rarely were latitude and longitude provided on the labels, thus each locality without these data was georeferenced using Google Earth. Coordinates were then entered into ArcGIS 10, and distribution maps produced.

## Structural orientation, terminology and morphological features

### Structural orientation and terminology

**Figure 5.**
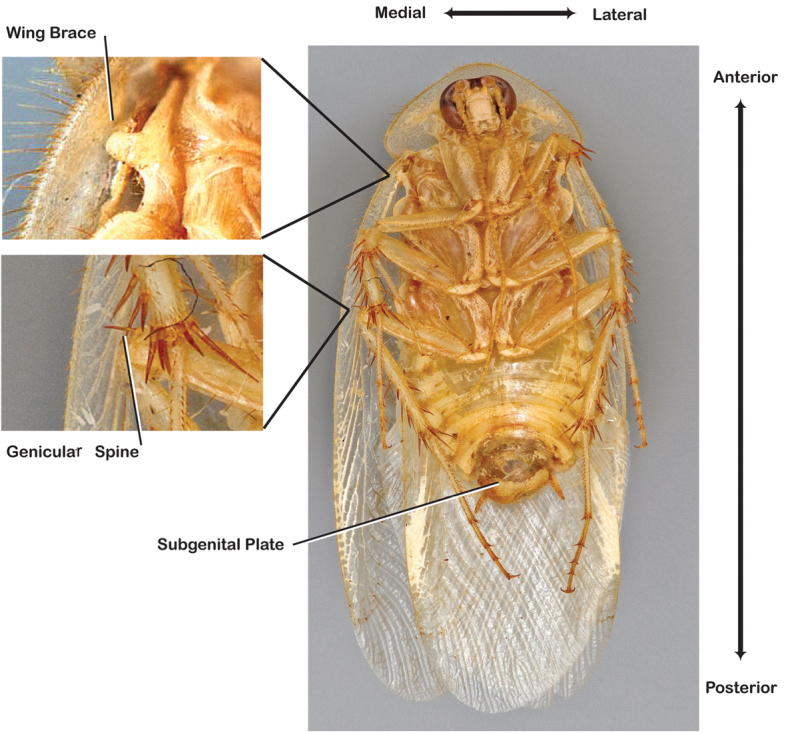
Labeled ventral habitus of *Arenivaga*.

**Figure 6.**
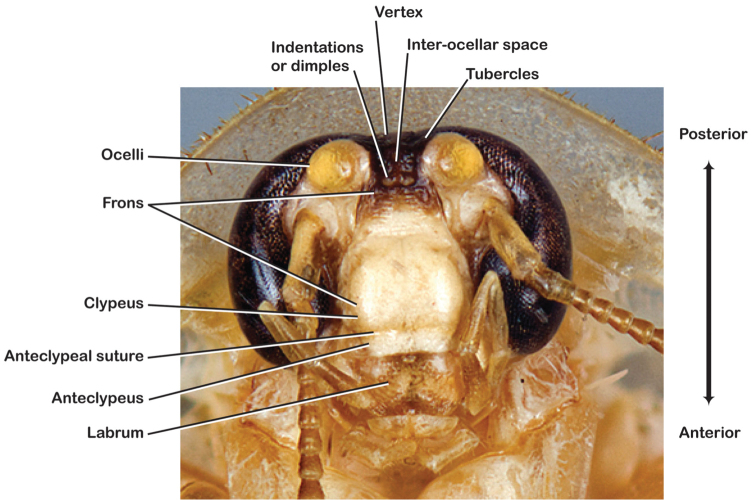
Labeled head of *Arenivaga*.

Orientation of the cockroach head can be particularly confusing because in insects, historically, much of the terminology is based on prognathous species. Since cockroaches are hypognathous the terminology requires some explanation. These and other descriptive adjectives (e.g. anterior, medial, etc.) are standardized here and shown in Figures 5 and 6. To preserve homology with other insect taxa, the area of the cranium with the mouthparts is the “anterior” part of the head, despite the fact that this part of the head is often directed ventrad.

**Figure 7.**
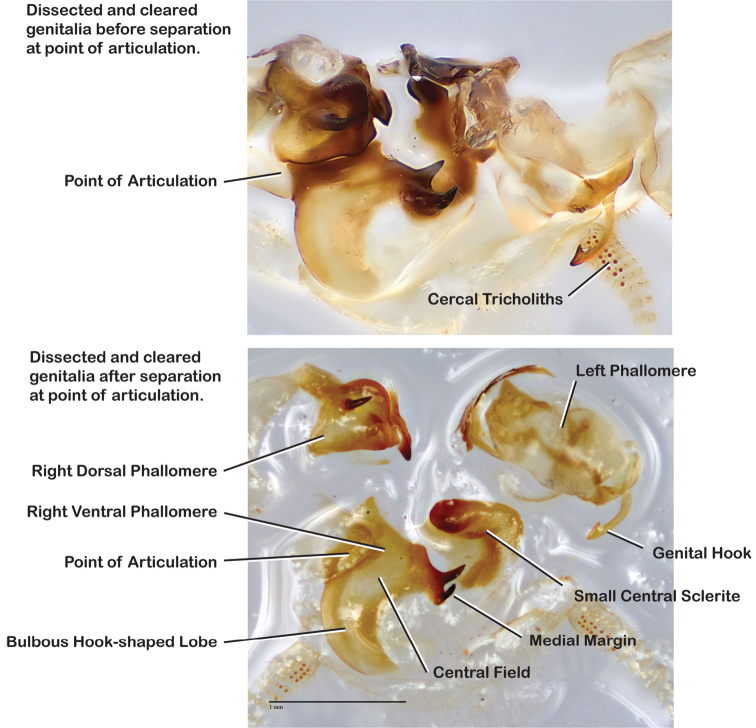
Labeled genitalia of *Arenivaga*.

The genitalia comprise three phallomeres, a small central sclerite and a genital hook, (Fig. 7). The left phallomere is rarely sclerotized to any great degree and is not drawn for most species. In a few species there are modifications to the left phallomere and in those cases the phallomere is drawn. In all drawings the right ventral phallomere has been separated from the right dorsal phallomere at the point where they articulate on the right side (Fig. 7). This was done so that the former may be rotated clockwise prior to drawing in order to show important details that are otherwise hidden. This permanent separation of the two phallomeres need not be done for species identification because the two phallomeres will open at the point of articulation sufficiently to see details.

### Morphological features

Adult *Arenivaga* are strikingly sexually dimorphic (Fig. 1). Because the species taxonomy in the group is based on males, the following discussion of morphology used in the descriptions focuses on males. Males of all species are winged. They are generally gracile and small-bodied with the wings extending well beyond the end of the abdomen in most species. The animal is dorso-ventrally flattened. Although color is given for each body part for each holotype it can only rarely be relied upon for species identification. It is apparent that *Arenivaga* sequester color from their food (H. Hopkins, pers. obs.), like all cockroaches are white when teneral and don’t set color for up to 24 hours after ecdysis, vary in color according to their habitat, and sequester varying amounts of uric acid which affects the appearance of color (Friauf and Edney 1969), all of which make color an unreliable character in *Arenivaga* except in a minority of species.

*Head*: Antennae are long and filiform, the eyes large and reniform, and the ocelli (of which there are two) are large and protruding. The size of the ocelli, distance between ocelli, and distance between the eyes are variable between species. The frons and post clypeus are joined and in most species bulging out from the face in a most unusual manner for Blattodea; in some species the area is less protruding. The clypeal suture is pronounced and the anteclypeus is flat. The labrum is broad. The coloration and indentations on the face are variable within and between species.

**Figure 8.**
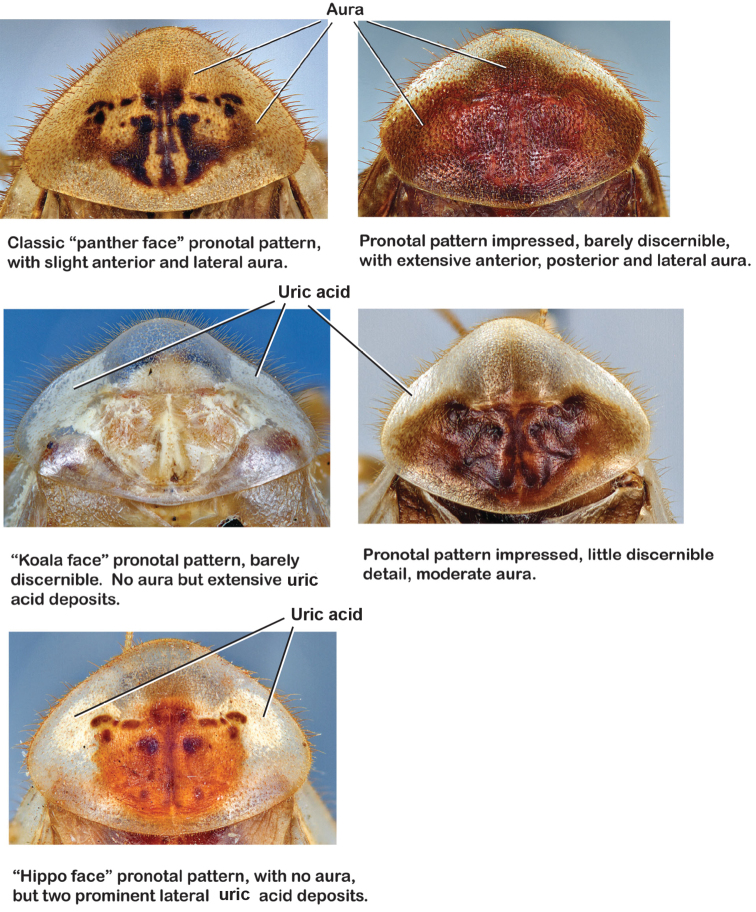
Examples of pronotal patterns, auras, and uric acid patches.

*Pronotum*: A large pronotum that covers the head like a hat or umbrella is the distinguishing character of cockroaches including *Arenivaga*. Their pronota entirely cover their heads and extend laterally, almost to the width of the body in many cases. All species’ pronota are setose on the dorsal surface with a thick band of short hair along the posteroventral margin and setae of varying lengths protruding from the anterior margin. Nearly all species of *Arenivaga* have a characteristic pronotal pattern though in some it is faint or diffuse and may be impressed or not. The pattern may be surrounded by an aura of varying extent or none at all. See Figure 8 for examples of pronotal patterns and auras. Pronotal aura should not be confused with patches of uric acid that are stored in the pronotum in many species. The aura radiates out from the pronotal pattern, may do so in all four directions, and is colored; uric acid patches are always located laterally on the pronotum (see Figure 8), are noncontiguous with the pronotal pattern, and are usually white though they may be a pale flesh-tone.

*Body*: The size range between species is considerable from tiny *Arenivaga pumila* (14.2 mm × 7.0 mm) to the largest *Arenivaga bolliana* (24.5 mm × 13.0 mm). The robustness is also wide-ranging from gracile *Arenivaga trypheros* (TL/GW 2.82) to broad *Arenivaga impensa* (TL/GW 2.16). All *Arenivaga* have two tarsal claws except for *Arenivaga darwini*, which has only one. The legs are heavily spined and genicular spines on the meso and metatibia were the defining character of the genus (Fig. 5). Some species have a wing brace, while others do not.

*Forewings*: The color, consistency of pigmentation (blotchiness), and degree of sheen of forewings are given in each description; most of these features are variable within species.

**Figure 9.**
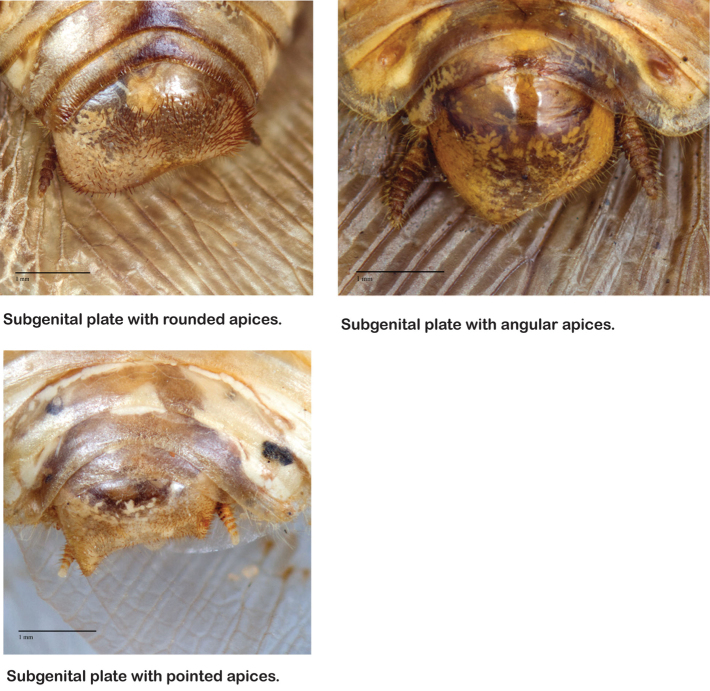
Examples of subgenital plates.

*Genitalia*: The genitalia of *Arenivaga* are complex. Variation in genitalia has been previously used to distinguish species (Rehn 1903, Caudell 1918, Hebard 1917, 1920, Friauf and Edney 1969) and it is the primary source of character information used to delimit species here. Although there is considerable variation in the genitalia across the group, in some cases the variation is subtle and species delimitation is difficult between potential species pairs. In addition to the striking variation in phallomeres, there is also variation in the genital hook and the subgenital plate (See Fig. 9). In one species, *Arenivaga rehni*, there appears an occasional rudimentary stylus on the right side of the subgenital plate in ventral view. Very occasionally there is the slightest hint of a second stylus on the left side in *Arenivaga rehni*. No other species showed evidence of styli. All close species designations (those that could be easily confused) are indicated in the individual descriptions and summarized in the Discussion section.

## Classification

### Taxonomic history

The name *Arenivaga* was first used by Rehn (1903) to circumscribe one of three subgenera of the genus *Homoeogamia* Burmeister, 1838. While describing a new species of *Homoeogamia* in 1893, Brunner placed the genus in the family Corydiidae. A year later, Saussure and Zehntner placed *Homoeogamia* in the subfamily Corydiinae (Saussure and Zehntner 1894). In 1903 Rehn erected three subgenera under *Homoeogamia*: *Homoeogamia* sensu strictu, *Arenivaga*, and *Eremoblatta*. He moved two species of *Homoeogamia* (*Homoeogamia bolliana* (Saussure, 1893) and *Homoeogamia apacha* (Saussure, 1893)) to the subgenus *Arenivaga*, and described one new species, *Homoeogamia erratica* (Rehn 1903). Caudell (1913), recognizing seven Nearctic subfamilies of cockroaches, raised *Arenivaga* and *Eremoblatta* to genus rank and placed them in the subfamily Corydiinae. Hebard (1917), using Kirby’s (1904) system of 16 cockroach subfamilies, placed *Arenivaga* in the subfamily Polyphaginae where it remained until the families Corydiidae and Polyphagidae, as well as the subfamilies Corydiinae and Polyphaginae, were recently synonymized by Beccaloni and Eggleton (2011). Hebard (1917) also described *Arenivaga rehni* Hebard 1917, and a year later, Caudell (1918) described two more species, *Arenivaga genitalis* Caudell 1918 and *Arenivaga floridensis* Caudell 1918. Hebard (1920) then revised the genus describing two new species, *Arenivaga tonkawa* Hebard, 1920 and *Arenivaga grata* Hebard, 1920. Finally, Friauf and Edney (1969) described *Arenivaga investigata* Friauf & Edney, 1969. The genus has not been revised since Hebard (1920).

#### Genus Arenivaga

(Rehn, 1903)

http://species-id.net/wiki/Arenivaga

Figures 33 –34

Homoeogamia (Arenivaga) Rehn 1903, Proceedings of the Academy of Natural Sciences of Philadelphia, Vol. 55, p. 188.
Arenivaga , Caudell 1913, Proceedings of the United States National Museum, Vol. 44, p. 605.

##### Type species.

*Arenivaga bolliana* (Saussure) by original designation.

##### Distribution.

**Figure 10.**
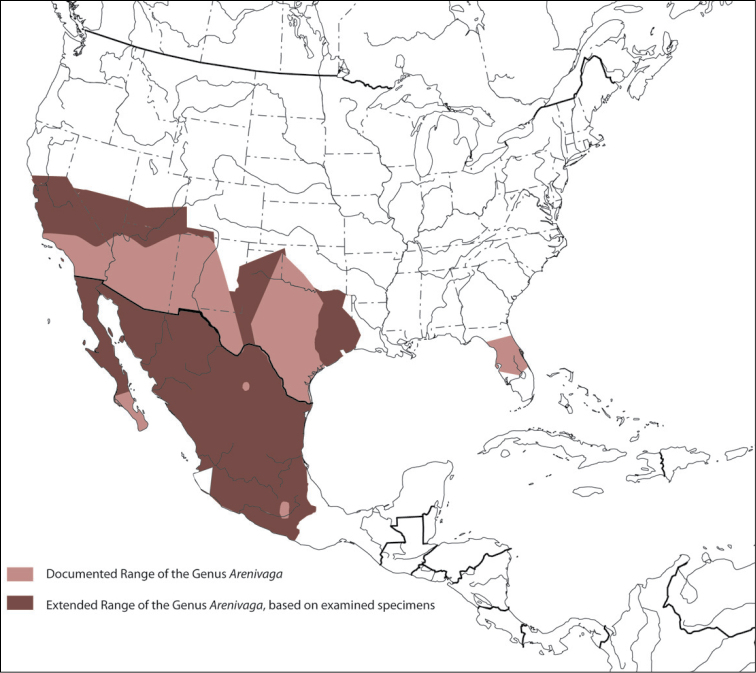
Previously documented and extended range of the genus *Arenivaga*.

The genus *Arenivaga* is found in central Florida and from Texas to California south into Mexico. They occur from about 39°N south to about 18°N (See Fig. 10).

##### Diagnosis.

Until now, *Arenivaga* were diagnosed from other Corydiid genera by the presence of cercal tricholiths (Fig. 7) and genicular spines on the meta- and mesothoracic legs (Fig. 5). The sister genus *Eremoblatta*, has cercal tricholiths but no genicular spines on the legs. A new species described in this work (*Arenivaga diaphana*) has polymorphic genicular spines, the majority of specimens studied having no genicular spines but two specimens were found with the characteristic *Arenivaga* genicular spine distribution. This undermines the character that until now separated *Arenivaga* from *Eremoblatta*. A gestalt of the phenotype of both sexes of the two genera allow easy determination between the two. But without some familiarity with both genera, or examples of both genera side by side, this method of determination is difficult. Generally speaking, *Eremoblatta* are smaller than *Arenivaga* and have pronota of consistent size with no pattern; the wings of the males are consistently glossy and wrinkled, and the females are considerably more hirsute than *Arenivaga* females. *Eremoblatta* do not appear to show intraspecific phenotypic variability due to variation in environment as do many *Arenivaga* species. The vast majority of *Arenivaga* specimens possess genicular spines on the meta- and mesothoracic legs, and the majority of specimens that lack genicular spines will be *Eremoblatta*. Genitalia provide a clear distinction as *Arenivaga* has a single-pronged genital hook and *Eremoblatta*’s is double-pronged.

##### Description.

**Male.** *Measurements*. Holotype TL = 24.6 mm, GW = 13.0 mm, PW = 8.64 mm, PL = 5.60 mm, TL/GW = 1.89, PL/PW = 0.65. EW = 0.40 mm; OW = 0.60 mm. Among paratypes range of TL 20.1–30.7 mm; range of GW 9.6–15.3 mm; range of PW 7.25–10.10 mm; range of PL 4.74–6.17 mm.

*Head*. Two ocelli large, ovoid and protruding; vertex flat, variable in color and width, most species with small ridges between apices of eyes that extend onto ocellar tubercles; interocellar space concave, of varying width, concavity depth and color. Frons color variable, tectiform, concave and/or with fine horizontal corrugations; margined on each side by ridges extending from medial margins of ocelli laterally to margins of clypeus with long or very long setae. Anterior portion of frons of variable color, bulbous to very bulbous in most species; clypeal suture with two proximal setae demarcating anteclypeus; labrum wide. Eyes large and reniform, medially emarginate, dark brown in life but color various in dried specimens. Antennae long, delicate and filiform, arising from medial emargination of eyes; antennomere number variable from ~53–67, though determination is made difficult by frequency of broken antennae on specimens.

*Pronotum*. Pronotum elliptical, variable in size, anterior margin convex, extending anteriorly over head; broad anterior margin translucent, waxy light brown. Setae of variable length along anterior margin; pale short dense setae projecting from ventral side of posterior margin; dorsal surface of pronotum covered with short setae; pronotal pattern may be impressed into surface or not, well demarcated or not, widely variable in color even within some species, with varying extent of aura; the pattern itself varies across the group and takes on certain distinctive appearances including semblance of “panther” or “hippo” faces, and, more rarely, a “koala” face pattern (Fig. 8).

*Body*. Abdomen and legs dorso-ventrally flattened; all legs heavily spinous and setose. Legs and body varying in color, often within a species; white deposits of uric acid visible through exoskeleton throughout body, legs, pronotum, and wing venation. Sternites rounded and setose laterally in most species. Wing brace (Fig. 5) may be present or absent but is consistent within each species. Tarsi with tarsomere I length equal to length of II-V combined; tarsomere IV shortest; with genicular spines on meso and metalegs (but see Diagnosis, above). Two tarsal claws present in all species but one. Subgenital plate asymmetrical with posterior edge emarginated, apices variable in shape; setose along posterior edge and posterior half of dorsal and ventral surfaces.

*Forewings*. Wings extended beyond abdominal apex to varying degrees; veins distinctly raised above surface anteriorly and laterally, becoming increasingly embedded in surface posteriorly and centrally; color ranges from pale clear golden tan to very dark brown; blotchiness absent in some species, consistent in others, variable in others; surface ranges from opaque to semi-opaque to translucent, and from matte to shiny; with variable length setae on anterior lateral edges decreasing to uniformly small posteriorly.

*Genitalia*. Distinctive and highly sculptural, the genitalia of *Arenivaga* distinguish and delimit species. This revision names and describes four phallomeres, though alternate interpretations of the limits of these structures are possible. While the structures are easy to homologize between species of the genus and close relatives, they are extremely difficult to homologize with the genitalia of other cockroach families or with a “generic” cockroach and no such analysis is attempted here. The phallomeres used in this revision are the right dorsal phallomere, the right ventral phallomere, the small central sclerite, and the left phallomere which includes the genital hook (Fig. 7). The two right phallomeres are hinged together on the lateral side of the animal but are disarticulated here prior to drawing so that as much detail as possible may be shown (Fig. 7).

##### Habitat and natural history.

**Figure 11.**
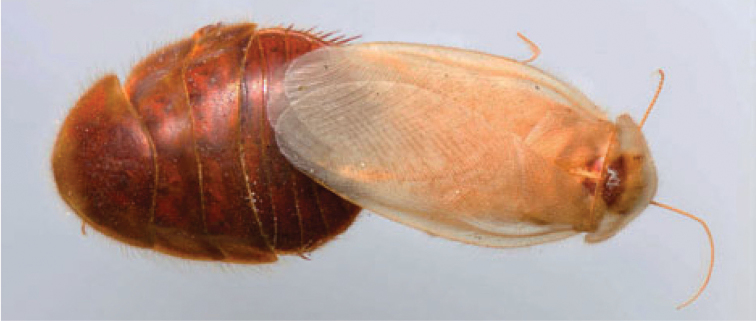
*Arenivaga* pair in copula.

*Arenivaga*, Latin for “sand runner”, are found in the American southwest, Mexico, and Florida (Fig. 10). Females and nymphs are subterranean in sandy, dune habitats, feeding on mycorrhizal fungi, leaf detritus of desert shrubs, and the seeds collected by the mammals whose burrows they cohabit (Cohen and Cohen 1976, Hawke and Farley 1973). Their cryptic life history has never been fully documented although their adaptations for life in the desert are well-studied (Walthall and Hartman 1981, Edney 1968, Hawke and Farley 1971a, 1971b, 1973, Cohen and Cohen 1976, 1981, Edney and McFarlane 1974, Hartman et al. 1987, Edney et al. 1974, 1978, Jackson 1983, O’Donnell 1977, 1981, 1982, Appel et al. 1983). Mature males, the only winged form, live most of their short lives above-ground (Appel et al. 1983). Females are most active near and at the surface during summer, which is most likely the mating season. Mature females “swim” to the surface after dark when the first few centimeters of sand have cooled. There, they wander the surface of the sand, presumably attracting males using pheromones (Hawke and Farley 1973). Courtship has never been observed, but mating proceeds in the typical end-to-end manner found in other Blattodea (Figure 11).

### Species treatments

#### Arenivaga adamsi sp. n.

http://zoobank.org/0FCD7D02-CD7F-41B9-82D3-2AD1F0B5F3E9

http://species-id.net/wiki/Arenivaga_adamsi

Figures 12 – 14

##### Type locality.

MEXICO, Sonora, Arroyo Cuchujaqui.

##### Material examined.

Holotype: ♂ in EMEC labeled “Arroyo Cuchujaqui, 7 mi. SE Alamos, Son., Mex. VI-19-63, Collr: W. A. Foster” “HOLOTYPE *Arenivaga adamsi* Hopkins, 2012” [red label with black border].

Paratypes (32): MEXICO: Sonora,Nogales, 6/1/1966, alive with fruit on truck (1, USNM); Sonora, Guaymas area, Ejido Buenos Aires, citrus, 5/22/2004, 27.59.093N 110.57.437W, SIB 2004.0040 (1, UAIC); Sonora, Guaymas area, Nacapule Canyon, 5/27/2003, 28.01N, 111.03W, SIB 2003.0027, Blue Tag 9000 (2, UAIC), Sonora, Guaymas area, SW bajada of Aguaje Mts., 5/19/2003, 28.001N, 111.06W, SIB 2003.0004, Blue Tag 9000 (1, UAIC); Sonora, Guaymas area, Nacapule Canyon, 5/21/2004, 28.01N, 111.03W, SIB 2004.0039 (1, UAIC); Sonora, Guaymas, 11/23/1969, Vars & Clifford (1, USNM); Sonora, Guaymas, 6/21/1962, AE Michelbacher (1, EMEC); Sonora, Guaymas, 7/23/1959, HE Evans (1, CUIC); Sonora, Navajoa, 6/24/1962, AE Michelbacher (1, EMEC); Sonora, Navajoa, 8/3/1952, C & P Vaurie (1, AMNH); Sonora, Navajoa, 8/18/1962, AE Michelbacher (1, EMEC); Sonora, Navajoa, 7/18/1972, J & MA Chemsak,A & M Michelbacher, at light (1, EMEC); Sonora, Arroyo Cuchujaqui,7 mi SE of Alamos, 6/19/1963, WA Foster (2, EMEC); Sonora, Alamos, 7/25-8/7/1953, Fred S. Truxal (4, LACM); Sonora, Cocorit, 6/11/1961, Menke & Stange (1, LACM); Sonora, 1.5 mi S of Presa de Mocuzari, 11/25/1967, R Rice, in rock pile (1, UAIC); Sinaloa, Baviri (playa) W of Los Mochis, 9/8/1986, Faulkner & Bloomfield (2, SDMC); Sinaloa, Los Mochis, 6/26/1962, AE Michelbacher (1, EMEC); Sinaloa, Los Mochis, 7/8/1922, CT Dodds, [Entire body missing] (1, CAS); Sonora, W side of Mazatan, 6/17/2012, N29.0017 W110.0863, 550 m, TR VanDevender & AL Reina-G, mesquite bosque, foothills thorn scrub on slopes (1, HEH); Sonora, W side of Mazatan, 8/13/12, 29.00472N, 110.14806W, 550 m, TR VanDevender & J. Palling (3, HEH); Sonora, 6 mi. NNW of C. Obregon, 6/2/1954, AA Alcorn (1, SEMC). USA: AZ, Cochise Co., Douglas, 7/22-25/1969, VD Roth (1, SWRS); AZ, Cochise Co. 28 mi E Douglas, Guadalupe Canyon, 6/24/1970 (1, SWRS). All paratypes labeled “Paratype *Arenivaga adamsi* Hopkins 2012” [blue label with black border].

##### Etymology.

The name is a noun in the genitive case. This species is named in honor and fond remembrance of Douglas Adams, whose writing makes me laugh, and who loved and respected all life on this planet.

##### Distribution.

This species is found in central Sonora and northern Sinaloa Mexico and southeastern Arizona. See Fig. 14.

##### Diagnosis.

*Arenivaga adamsi* may be confused with *Arenivaga moctezuma* but can be distinguished by the single large spine on the posterior end of the medial margin of the right dorsal phallomere. See Figs 11 and 104.

##### Description.

**Male.** *Measurements*. Holotype TL = 17.5 mm, GW = 9.0 mm, PW = 6.13 mm, PL = 4.33 mm, TL/GW = 1.94, PL/PW = 0.71. EW = 0.30 mm; OW = 0.30 mm. Among paratypes range of TL 15.8–20.7 mm; range of GW 7.3–10.3 mm; range of PW 5.47–6.33 mm; range of PL 3.60–4.57 mm.

**Figure 12.**
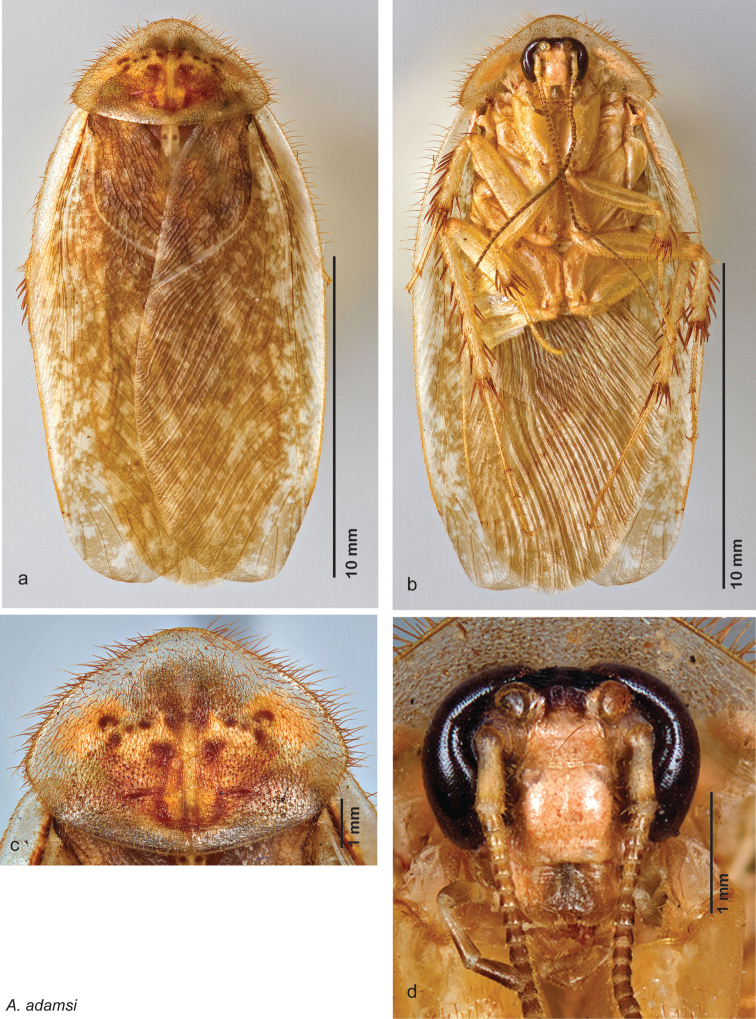
*Arenivaga adamsi*, **a** dorsal habitus **b** ventral habitus **c** pronotum **d** head.

*Head*. Two ocelli large, ovoid and protruding (0.40 × 0.30 mm); vertex dark brown with small ridges in rays around upper apices of eyes and extending onto ocellar tubercles; interocellar space concave, medium brown. Frons translucent peach-brown, unusually wide, posterior concave; anterior portion of frons bulbous and translucent peach-brown; translucent peach-brown smooth anteclypeus. See Fig. 12d.

*Pronotum*. Pronotum translucent waxy beige; dorsal surface of pronotum with short orange-brown setae that are thicker and longer laterally; pronotal pattern orange-brown or red-brown “panther face”, with scattered maculations of same color as pattern; discernible detail within pattern variable; slight aura. See Fig. 12c.

*Body*. Wing brace present. Two tarsal claws present. Legs and body light orange-brown; many specimens with brown maculations laterally on each sternite; subgenital plate light orange-brown; asymmetrical with rounded apices. See Fig. 12b.

*Forewings*. Wings extended well beyond abdominal apex (up to 40% of wing length); blotchy medium to dark brown depending on specimen; surface matte and opaque. See Fig. 12a.

**Figure 13.**
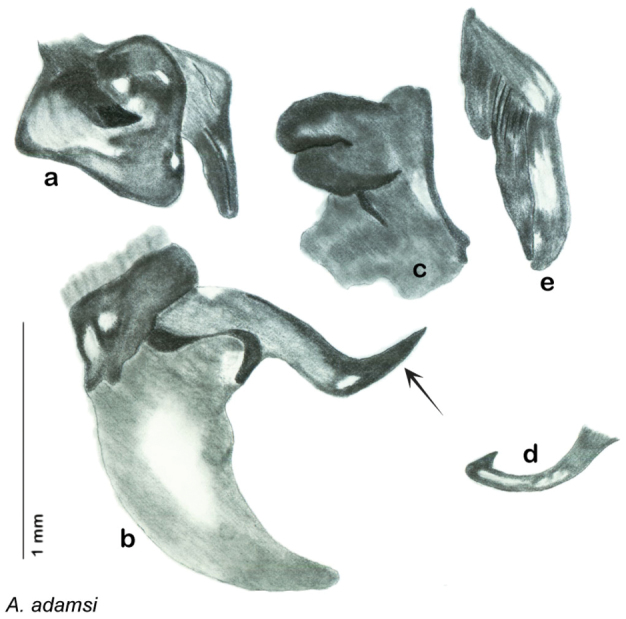
*Arenivaga adamsi*, genitalia: **a** right dorsal phallomere **b** right ventral phallomere **c** small central sclerite **d** genital hook **e** left phallomere. Arrow(s) indicate diagnostic characters (see text).

*Genitalia*. Right dorsal phallomere composed of lightly sclerotized, long, bulbous hook-shaped lobe, articulated with right ventral phallomere on lateral side; medial side of lobe deeply emarginated from medial edge of remainder of phallomere; central field shallow, cupped, lightly sclerotized; medial margin more heavily sclerotized, smooth, with long posterior projecting spine. Small central sclerite punctate, with large shagreened, rugose, medially projecting bilobed bulge; right ventral phallomere extends from articulation to form shagreened rounded structure, with prominent medially projecting spine located posteriorly; attached anteriorly is mildly dorsally projecting flanged smooth concave arm, that extends only slightly beyond depth of rest of phallomere, apex punctate. Folded anterior portion of left phallomere dramatically modified with sclerotized anterior wall and posteriorly projecting setose spine located ventrally. Genital hook with short extension to pointed head with moderate hook; arm smoothly curved. See Fig. 13.

**Figure 14.**
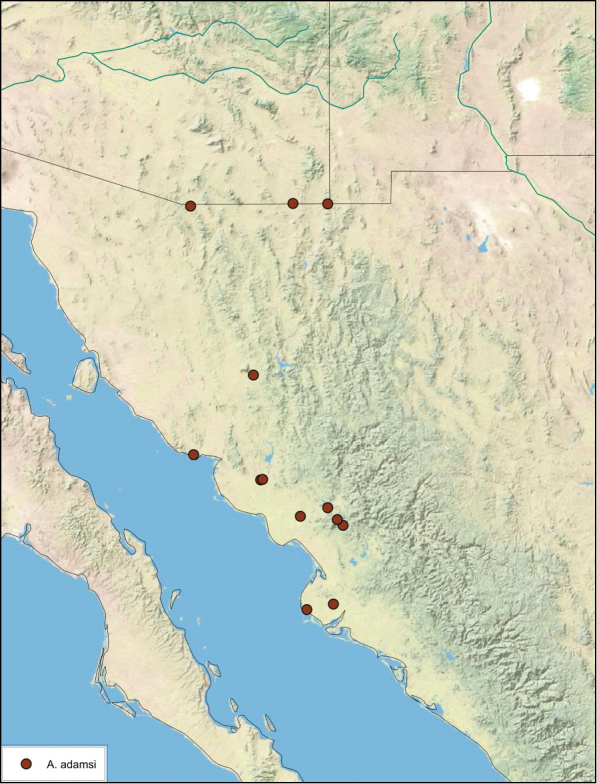
*Arenivaga adamsi*, distribution.

##### Habitat and natural history.

All life history elements remain unobserved.

#### Arenivaga akanthikos sp. n.

http://zoobank.org/7ED6E664-C9A5-4BEA-A5C7-EDDAF4284DA7

http://species-id.net/wiki/Arenivaga_akanthikos

Figures 15 – 17

##### Type locality.

MEXICO, Sonora, 8 km W of Carbo.

##### Material examined.

Holotype: ♂ in UAIC labeled “8 km. W. of Carbo, Son. Mex. 5-X-60, at light, Wm. W. Gibson Collector” “HOLOTYPE *Arenivaga akanthikos* Hopkins, 2012” [red label with black border].

Paratypes (1): MEXICO: Sonora, 8 mi W of Caborca, 3/20/1980, CE Griswold (1, EMEC). All paratypes labeled “Paratype *Arenivaga akanthikos* Hopkins 2012” [blue label with black border].

##### Etymology.

The name is an adjective in the nominative singular. This species is named from the Greek meaning thorny because of the amazing number of thorns present on its genitalia.

##### Distribution.

This species is found in the northwest part of Sonora, Mexico. See Fig. 17.

##### Diagnosis.

*Arenivaga akanthikos* can be distinguished by its having three spines on right dorsal phallomere, one on the right ventral phallomere, one on the small central sclerite, and one on the left phallomere. See Fig. 16.

##### Description.

**Male.** *Measurements*. Holotype TL = 18.3 mm, GW = 8.1 mm, PW = 5.84 mm, PL = 4.20 mm, TL/GW = 2.26, PL/PW = 0.72. EW = 0.25 mm; OW = 0.30 mm. No notable difference in size among paratypes.

**Figure 15.**
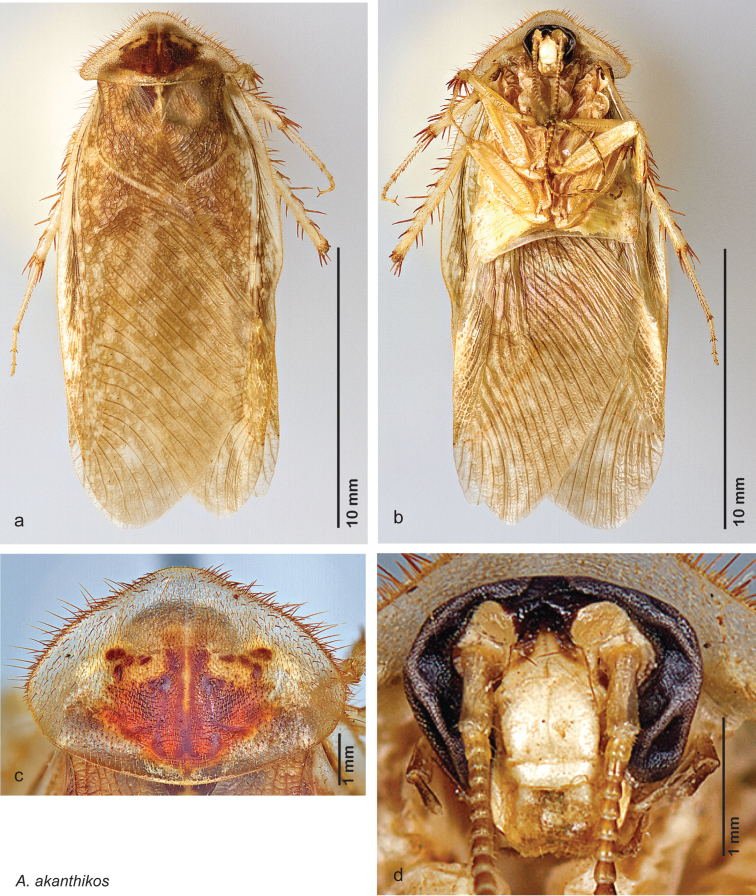
*Arenivaga akanthikos*, **a** dorsal habitus **b** ventral habitus **c** pronotum **d** head.

*Head*. Two ocelli large, ovoid and protruding (0.40 × 0.30 mm); vertex dark brown with small ridges in rays around upper apex of eyes and extending onto ocellar tubercles; interocellar space concave, dark brown, with two small round indentations. Frons waxy white with brown edges near ocelli; posterior concave with occasional long setae; bound on either side by ridges extending from inner apex of ocelli outwards to lateral edges of clypeus. Anterior portion of frons bulbous and waxy white; clypeal suture with two proximal setae, demarcates waxy white smooth anteclypeus. See Fig. 15d.

*Pronotum*. Pronotum average in size for genus; translucent waxy beige; variable length orange-brown setae along anterior margin; setae on dorsal surface of pronotum thicker and longer laterally; pronotal pattern dark orange-brown “panther face”, not impressed, detail discernible; small lateral and anterior aura. See Fig. 15c.

*Body*. Wing brace present. Legs and body light brown; one specimen with brown maculations laterally on each sternite; subgenital plate light brown with rounded apices. See Fig. 15b.

*Forewings*. Wings extended well beyond abdominal apex (~35% of wing length); blotchy medium to dark brown; surface matte and opaque. See Fig. 15a.

**Figure 16.**
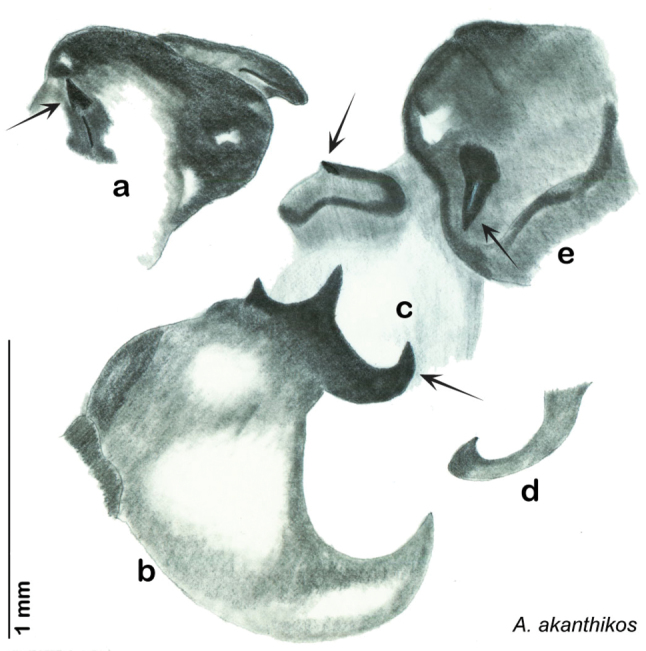
*Arenivaga akanthikos*, genitalia: **a** right dorsal phallomere **b** right ventral phallomere **c** small central sclerite **d** genital hook **e** left phallomere. Arrow(s) indicate diagnostic characters (see text).

*Genitalia*. Right dorsal phallomere composed of lightly sclerotized, unusually curved, bulbous hook-shaped lobe, articulated with right ventral phallomere on lateral side; medial side of lobe deeply emarginated from medial edge of remainder of phallomere; central field shallow, cupped, lightly sclerotized; medial margin more heavily sclerotized, smooth, with long posterior projecting spine and two medially projecting spines located on anterior third of medial margin. Small central sclerite concave, punctate, with large shagreened medially projecting wide upside down V-shape on ventral edge, point of which extends into small spine; right ventral phallomere extends from articulation to form shagreened rounded structure, with prominent medially projecting spine located posteriorly; attached anteriorly is flanged punctate concave arm that extends slightly beyond depth of rest of phallomere, edge shagreened. Folded anterior portion of left phallomere dramatically modified with sclerotized anterior wall and posteriorly projecting smooth spine located ventrally. Genital hook with short extension to pointed head with short hook. See Fig. 16.

**Figure 17.**
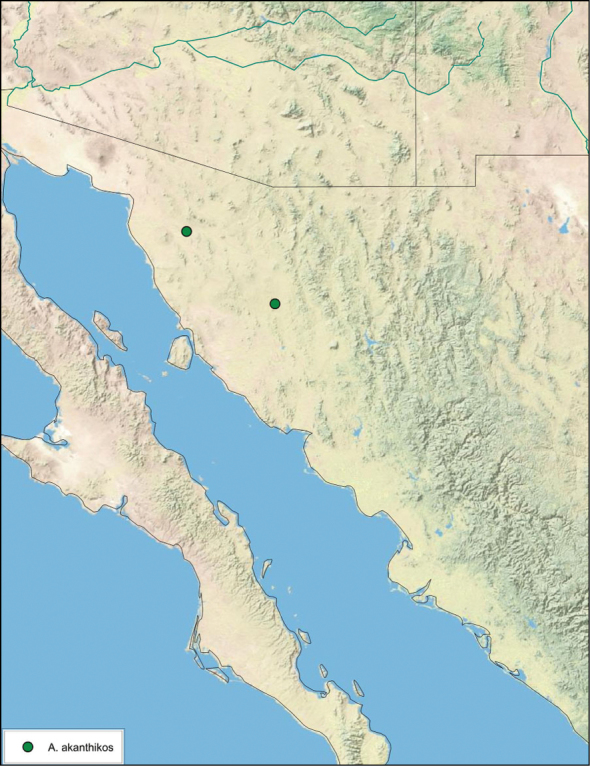
*Arenivaga akanthikos*, distribution.

##### Habitat and natural history.

All life history elements remain unobserved.

#### Arenivaga alichenas sp. n.

http://zoobank.org/CD31B67A-EF22-4F49-A50B-2B978ABB63E9

http://species-id.net/wiki/Arenivaga_alichenas

Figures 18 – 20

##### Type locality.

MEXICO, BC, hills S of Laguna el Rosario.

##### Material examined.

Holotype: ♂ in USNM labeled “MEXICO: Baja Calif., hills S of Laguna El Rosario 5-VIII-1973, leg. L.J. Oraak, ?.?. Simpson, ex. Lichen, bee collecting notes of L.J. Oraak (LJO-1076)” “HOLOTYPE *Arenivaga alichenas* Hopkins, 2012” [red label with black border].

Paratypes (1): MEXICO: BC, Valle de la Trinidad, 7/?/1927, LM Muey (1, SDMC). All paratypes labeled “Paratype *Arenivaga alichenas* Hopkins 2012” [blue label with black border].

##### Etymology.

This species is named from the Latin meaning “from lichen” because the holotype was taken from lichen.

##### Distribution.

This species is found in western and north-central Baja California Norte, Mexico. See Fig. 20.

##### Diagnosis.

*Arenivaga alichenas* may be diagnosed by very narrow hook-shaped lobe on the right dorsal phallomere and the sclerotized edge of the anterior portion of the small central sclerite. See Fig. 19.

##### Description.

**Male.** *Measurements*. Holotype TL = 14.3 mm, GW = 7.2 mm, PW = 5.00 mm, PL = 3.55 mm, TL/GW = 1.97, PL/PW = 0.71. EW = 0.70 mm; OW = 0.45 mm. No notable difference in measurements among paratypes.

**Figure 18.**
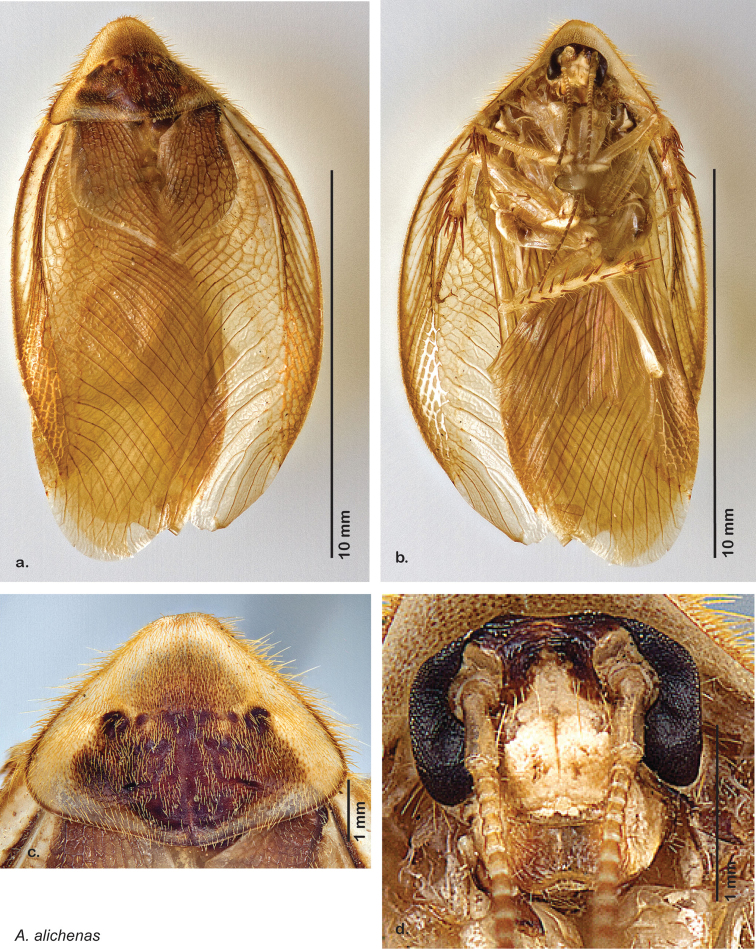
*Arenivaga alichenas*, **a** dorsal habitus **b** ventral habitus **c** pronotum **d** head.

*Head*. Two ocelli large, ovoid and protruding (0.30 × 0.20 mm); vertex medium brown, with small ridges in rays around upper apices of eyes and extending onto ocellar tubercles; interocellar space deeply concave, medium brown, lighter medially. Frons waxy white, concave; anterior portion of frons waxy white, bulbous; light brown anteclypeus. See Fig. 18d.

*Pronotum*. Pronotum small; translucent waxy beige with fine dark brown margin; dorsal surface of pronotum covered with dense golden brown setae, could be called furry; pronotal pattern dark orange-brown “panther face” with medium to dark brown aura; detail impressed. See Fig. 18c.

*Body*. Wing brace present. Legs and body light brown; subgenital plate dissected and cleared with angular apices. See Fig. 18b.

*Forewings*. Wings extended beyond abdominal apex (up to ~30% of total wing length); no blotchiness, medium to dark brown; surface translucent, with slight sheen. See Fig. 18a.

**Figure 19.**
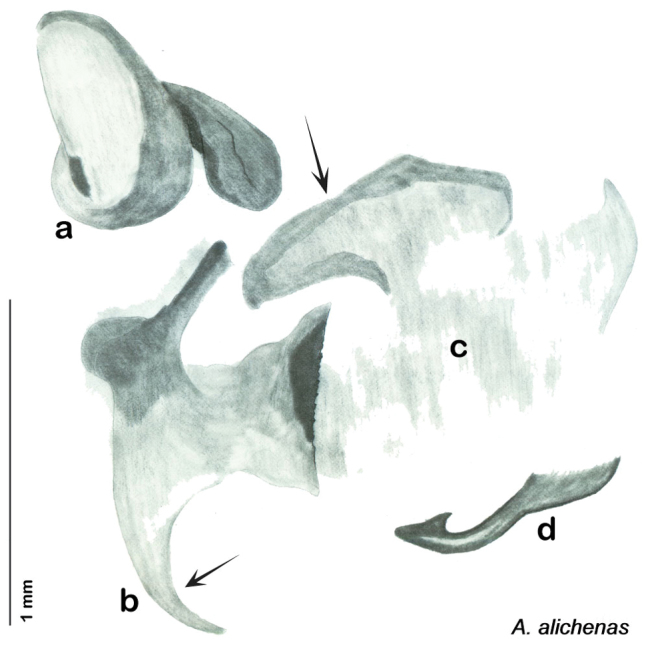
*Arenivaga alichenas*, genitalia: **a** right dorsal phallomere **b** right ventral phallomere **c** small central sclerite **d** genital hook. Arrow(s) indicate diagnostic characters (see text).

*Genitalia*. Right dorsal phallomere composed of bulbous lightly sclerotized narrow hook-shaped lobe, articulated with right ventral phallomere on lateral side; central field broad, lightly sclerotized, punctate; medial margin heavily sclerotized, shagreened with straight toothed edge. Small central sclerite concave, with punctate crescent shape with more sclerotized margins anteriorly; anterior rounded point curves back on itself; posterior margin attached to dorsal side of right dorsal phallomere. Right ventral phallomere extends from articulation to form flattened smooth lobe, increasingly punctate and sclerotized anteriorly; after narrow gap, wide rounded concave shagreened arm extending to depth of rest of phallomere. Folded anterior portion of left phallomere narrow, setose, otherwise unmodified. Genital hook with long extension to pointed head with slight concavity on short hook; arm smoothly curved. See Fig. 19.

**Figure 20.**
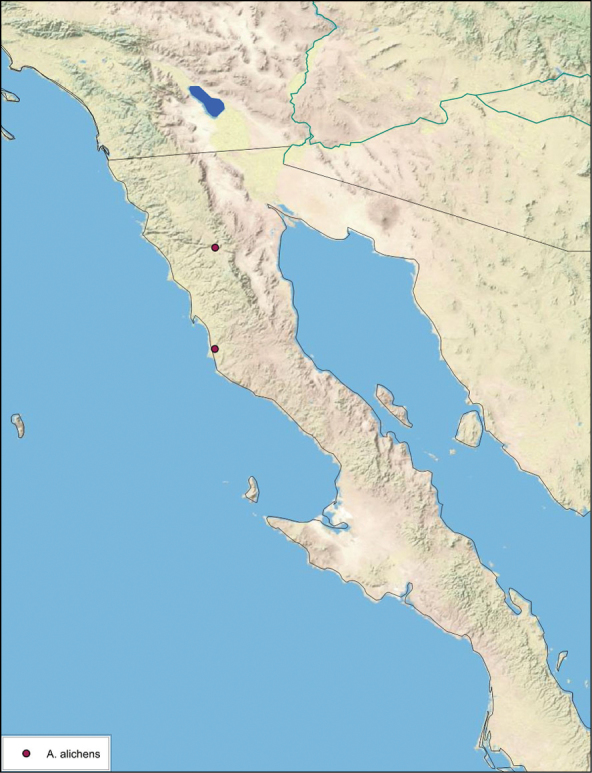
*Arenivaga alichenas*, distribution.

##### Habitat and natural history.

All life history elements remain unobserved.

#### Arenivaga apacha

(Saussure)

http://species-id.net/wiki/Arenivaga_apacha

Figures 21 – 23

[Homoeogamia] apacha Saussure 1893, Revue Suisse de Zoologie, I, Fasc. 2, p.296. [Chihuahua, Mexico.]
Homoeogamia apacha , Saussure and Zehntner 1894, Biol. Cent.-Amer., Orthopt., I, pp. 107–108. [Chihuahua, Mexico.]
Homoeogamia (Arenivaga) apacha Rehn 1903, Proceedings of the Academy of Natural Sciences of Philadelphia, Vol. 55, p. 188.
Homoeogamia (Arenivaga) apacha infuscata Caudell 1905, Proceedings of the US National Museum, Vol. 28, pp. 462-463. [Phoenix, Arizona.]
Arenivaga apacha (Saussure) Hebard 1917, Memoirs of the American Entomological Society, No. 2, pp. 236–239.
Arenivaga apacha (Saussure) 1920, Transactions of the American Entomological Society, Vol. 46, pp. 213–214.

##### Material examined

**(343).** USA: AZ, Chiricahua Mts., 8/21/1962, D.J. & J.N.Knull (1, OSUC); AZ, Chiricahua Mts., 9/4/1962, D.J. & J.N.Knull (1, OSUC); AZ, Chiricahua Mts., 7/9/1959, D.J. & J.N.Knull (1, OSUC); AZ, Chiricahua Mts., 7/17/1957, D.J. & J.N.Knull (1, OSUC); AZ, Chiricahua Mts., 7/22/1957, D.J. & J.N.Knull (1, OSUC); AZ, Chiricahua Mts., 8/2/1952, D.J. & J.N.Knull (1, OSUC); AZ, Chiricahua Mts., 7/19/1952, D.J. & J.N.Knull (2, OSUC); AZ, Fish Creek, Tonto NF, 5/9-10/1918, J.C.Bradley (1, ANSP); AZ, Gila Co., Tonto Natural Bridge SP, 9/11/2010, 34.19.16N 111.27.24, Warner & Smith, UVBL lights, (1, WB Warner); AZ, Pima Co., 7/27/1927, R.H.Beamer (1, ANSP); AZ, Globe, 6/19/1957, Butler & Werner, at light (3, UAIC); AZ, Globe, 6/2/1935, Parker (1, UCRC); AZ, Pima Co., Santa Rita Mts., Box Canyon, 7/21/1995, Olson,Hall et al. (1, UAIC); AZ, Pima Co., Santa Rita Mts., Box Canyon, 7/9/1976, D.Whitman #581 (1, EMEC); AZ, Pima Co., Rincon Mts., Mack Burn Site, 6/30/1995, Pitfall 5, Pitfall 2 (2, UAIC); AZ, Pima Co., Rincon Mts., Mack Burn Site, 8/12/1995, Pitfall 11 (2, UAIC); AZ, Pima Co., 8 mi. N of Vail, 6/26/1962, F.Werner, UV Light trap (1, UAIC); AZ, Pima Co., 8 mi. N of Vail, 8/30/1962, Werner & Nutting, UV Light trap (1, UAIC); AZ, Cochise Co., Miller Canyon, Huachuca Mts., 7/8/1974, 5000’, E.R.Hoebeke (1, CUIC); AZ, Cochise Co., Chiricahua Mts., Silver Creek Wash, 0.7 mi. W of Portal, 8/2/1966, 4870’, R.G.Beard, UV Light trap (2, CUIC); AZ, Cochise Co., Guadalupe Canyon in wash at entry into canyon, 8/4/1966, 4200’, R.G.Beard, UV Light trap (1, CUIC); AZ, Cochise Co., Pyeatt’s Ranch, 6/29/1953, 6000’, A & H Dietrich (3, CUIC); AZ, Cochise Co., Portal, 8/22/1959, 5000’, H.E.Evans, at light (1, CUIC); AZ, Cochise Co., Miller Canyon, Huachuca Mts., 6/25/1974, 5000’, E.R.Hoebeke (1, CUIC); AZ, Cochise Co., Miller Canyon, Huachuca Mts., 7/24/1974, 5000’, E.R.Hoebeke (1, CUIC); AZ, Cochise Co., Miller Canyon, Huachuca Mts., 6/28/1974, 5000’, T.L.McCabe (1, CUIC); AZ, Cochise Co., Miller Canyon, Huachuca Mts., 7/8/1974, 5000’, T.L.McCabe (2, CUIC); AZ, Cochise Co., Miller Canyon, Huachuca Mts., 6/25/1974, 5000’, T.L.McCabe (2, CUIC); AZ, Cochise Co., Miller Canyon, Huachuca Mts., 7/13/1974, 5000’, E.R.Hoebeke (1, CUIC); AZ, Cochise Co., Miller Canyon, Huachuca Mts., 7/7/1974, 5000’, E.R.Hoebeke (2, CUIC); AZ, Cochise Co., Miller Canyon, Huachuca Mts., 7/7/1974, 5000’, T.L.McCabe (1, CUIC); AZ, Cochise Co., Miller Canyon, Huachuca Mts., 6/20/1974, 5000’, T.L.McCabe (1, CUIC); AZ, Cochise Co., Miller Canyon, Huachuca Mts., 7/4/1974, 5000’, T.L.McCabe (1, CUIC); AZ, Cochise Co., Miller Canyon, Huachuca Mts., 7/21/1974, 5000’, T.L.McCabe, (1, CUIC); AZ, Cochise Co., Miller Canyon, Huachuca Mts., 7/5/1974, 5000’, T.L.McCabe (6, CUIC); AZ, Cochise Co., Miller Canyon, Huachuca Mts., 7/8/1974, 5000’, E.R.Hoebeke (2, CUIC); AZ, Cochise Co., Miller Canyon, Huachuca Mts., 7/12/1974, 5400’, E.R.Hoebeke (1, CUIC); AZ, Cochise Co., Miller Canyon, Huachuca Mts., 7/4/1974, 5000’, E.R.Hoebeke (1, CUIC); AZ, Graham Co., Noon Creek, Graham Mts., 7/8/1965, Werner & Butler (7, UAIC); AZ, Cave Creek, 8/22/1926, W.W.Jones (1, UAIC); AZ, Noon Creek, Mt. Graham, 7/28/1954, F.G.Werner, light (1, UAIC); AZ, Cochise Co., SWRS, Chiricahua Mts. 4 mi. W of Portal, 6/24/1956, O.L.Cartwright (1, UAIC); AZ, Pima Co., Sabino Canyon, Santa Catalina Mts., 8/5/1949, 3500’, G.M.Bradt (1, AMNH); AZ, Huachuca Mts., 7/8/1932, J.D.Beamer (1, SEMC); AZ, Cochise Co., Carr Canyon, Huachuca Mts., 6/3/1952, Cazier,Gertsch & Schrammel (1, AMNH); AZ, Cochise Co., Paradise, Chiricahua Mts., 7/3/1954, Cazier & Gertsch (1, AMNH); AZ, Chiricahua Mts., 7/8/1932, R.H.Beamer (2, SEMC); AZ, Graham Co., Wet Canyon, Graham Mts., 9/14/1950, 6000’-6500’, Gertsch & Cazier (1, AMNH); AZ, Cochise Co., Portal, 6/1/1952, Cazier,Gertsch & Schrammel (2, AMNH); AZ, Cochise Co., SWRS 5 mi. W of Portal, 6/19/1957, 5400’, M.Statham (1, AMNH); AZ, Cochise Co., SWRS 5 mi. W of Portal, 5/15/1956, 5400’, M.Statham (1, AMNH); AZ, Cochise Co., SWRS 5 mi. W of Portal, 7/19/1955, 5400’, W.J.Gertsch (1, AMNH); AZ, Cochise Co., SWRS 5 mi. W of Portal, 5/3/1956, 5400’, M.Statham (1, AMNH); AZ, Cochise Co., SWRS 5 mi. W of Portal, 7/7/1956, 5400’, C & M Cazier (1, AMNH); AZ, Cochise Co., Palmerlee, Miller Canyon, 6/29/1950, R.F.Smith (2, AMNH); AZ, Cochise Co., Tombstone, 8/?/1975, GSF (1, SDMC); AZ, Cochise Co., 2 mi. E of Portal, 7/15/1955, E.Ordway (1, AMNH); AZ, Gila Co., Globe, 7/16-17/1948, 3600’, Werner & Nutting, at light, mesquite-cholla (1, UAIC); AZ, Cochise Co., SWRS Cave Creek Canyon, Chiricahua Mts., 6/13/1938, 5400’, Burns & Burns (1, EMEC); AZ, Benson, 7/4/1947, E.R.Tinkham (2, USNM); AZ, Huachuca Mts., Catal. No. 28, Brooklyn Museum Colln. 1929 (1, LACM); AZ, Bowie, 7/14/1917, Wheeler (2, UMMZ); AZ, Santa Rita Mts., 7/10/1950, H.O.Wright (1, SEMC); AZ, Gila Co., White Mts., 5/16/1925, 6000’, O.C.Poling (1, UMMZ); AZ, Cochise Co., 10 mi. E of Sierra Vista, 8/8/1977, Allen & Duffy, collected at blacklight (1, CSCA); AZ, Yuma Co., Wellton, 3/3/1925, O.C.Poling (2, UMMZ); AZ, C.U.Lot 34, Cornell U. Lot 677 Sub.10 (1, CUIC); AZ, Cochise Stronghold, Dragoon Mts., 7/16/1958, C.W.O’Brien (4, UAIC); AZ, Cochise Co., Cochise Stronghold, 6/29/1969, L.S.Hawkins (1, CSCA); AZ, Pima Co., Madera Canyon, Santa Rita Mts., 8/9-20/1978, DKF (1, SDMC); AZ, Santa Rita Mts., 7/9/1947, L.D.Beamer (1, SEMC); AZ, Santa Cruz Co., Madera Canyon, Santa Rita Mts., 7/25/1959, 4880’, J.C.Franclemont (1, CUIC); AZ, Santa Cruz Co., Madera Canyon, Santa Rita Mts., 7/14/1959, 4880’, J.C.Franclemont (1, CUIC); AZ, Cochise Co., Miller Canyon, Huachuca Mts., 7/3/1974, 5000’, E.R.Hoebeke (1, CUIC); AZ, Cochise Co., Miller Canyon, Huachuca Mts., 7/2/1974, 5000’, T.L.McCabe (1, CUIC); AZ, Cochise Co., Miller Canyon, Huachuca Mts., 8/9/1974, 5000’, T.L.McCabe (1, CUIC); AZ, Cochise Co., Miller Canyon, Huachuca Mts., 6/24/1974, 5000’, E.R.Hoebeke (1, CUIC); AZ, Cochise Co., Miller Canyon, Huachuca Mts., 6/23/1974, 5000’, E.R.Hoebeke (2, CUIC); AZ, Cochise Co., Miller Canyon, Huachuca Mts., 7/4/1974, 5000’, E.R.Hoebeke (4, CUIC); AZ, Cochise Co., Miller Canyon, Huachuca Mts., 7/5/1974, 5000’, E.R.Hoebeke (1, CUIC); AZ, Cochise Co., Miller Canyon, Huachuca Mts., 7/16/1974, 5000’, E.R.Hoebeke (1, CUIC); AZ, Cochise Co., Miller Canyon, Huachuca Mts., 6/27/1974, 5000’, E.R.Hoebeke (1, CUIC); AZ, 8 mi. N of Vail, 8/7/1966, F.Werner family, UV trap (1, UAIC); AZ, Pima Co., Catalina Mts. Y camp N side, 7/20/1961, at light (1, FSCA); AZ, Cochise Co., Miller Canyon, Huachuca Mts., 8/3/1974, 5000’, T.L.McCabe (1, CUIC); AZ, Cochise Co., Miller Canyon, Huachuca Mts., 6/22/1974, 5000’, E.R.Hoebeke (1, CUIC); AZ, Gila Co., White Mts., 7/12/1935, 7000’, O.C.Poling (8, UMMZ); AZ, Gila-Pinal Co., Miami, Pinal Mts., 5/18-25/1925, 5000’, O.C.Poling (6, UMMZ); AZ, Cochise Co., Miller Canyon, Huachuca Mts., 8/13/1974, 5000’, E.R.Hoebeke (1, CUIC); AZ, Gila Co., Globe, 7/12/1925, C.J.Alden (2, UMMZ); AZ, Gila Co., White Mts., 7/11/1925, 6000’, O.C.Poling (1, UMMZ); AZ, Gila Co., White Mts., 6/27/1925, 7000’, O.C.Poling (1, UMMZ); AZ, Madera Canyon, Santa Rita Mts., 7/8/1970, W.E & C.A.Triplehorn (1, OSUC); AZ, Santa Cruz Co., Madera Canyon, Santa Rita Mts., 7/4/1980, R.H.Crandall (2, LACM); AZ, Cochise Co., 1 mi. S of Portal, 7/17/1965, 4800’, Davidson,Davidson & Cazier,, at light (1, ASU); AZ, Cochise Co., W side of Wilcox Dry Gulch near Cochise, 9/2/1991, 4200’, Miller & Sta???, (1, FSCA); AZ, Cochise Co., SWRS 5 mi. SW of Portal, 5/23/1979, 5300’, W.B.Warner, at light (1, ASUT); AZ, Cochise Co., SWRS 5 mi. SW of Portal, 7/2/1979, 5300’, W.B.Warner, at light (1, ASUT); AZ, Yavapai Co., Prescott, 7/15/1984, C.R.Ash, (1, UAIC); AZ, Yavapai Co., Prescott, 7/?/1985, D.Tuttle, (1, UAIC); AZ, Yavapai Co., Prescott, 7/?/1987, D.Tuttle, (3, UAIC); AZ, Yavapai Co., Prescott, 6/15/1986, D.Tuttle, (2, UAIC); AZ, Yavapai Co., Prescott, 7/28/1984, C.R.Ash, at light (1, UAIC); AZ, Yavapai Co., Montezuma Castle NM, 5/25/1993, S.W.Fondriest, black light trap, site 12, round blue label (1, NAUF); AZ, Yavapai Co., Montezuma Well NM, 7/13/1993, S.W.Fondriest, black light trap, site 10, round blue label (1, NAUF); AZ, Greenlee Co., Eagle Creek, 7/1/1977, 3700’, (1, ASUT); AZ, Mescal, 7/28/1927, L.A.Anderson, (1, ANSP); AZ, Huachuca, Kunze, (1, ANSP); AZ, Huachuca Mts., 7/8/1932, R.H.Beamer, (2, ANSP); AZ, Santa Rita Mts., 7/29/1979, R.H.Crandall, (2, LACM); AZ, Chiricahua Mts., 7/8/1932, R.H.Beamer, (1, ANSP); AZ, Santa Rita Mts., 8/29/1924, C.T.Vorhies, (1, ANSP); AZ, Santa Rita Mts., 6/16/1926, 4000’, C.T.Vorhies, (1, ANSP); AZ, Santa Rita Mts., 10/?/1936, Bryant, (1, UCRC); AZ, Santa Rita Mts., 6/21/1936, R.A.Flock, (1, ANSP); AZ, Pima Co., Sycamore Canyon, Santa Rita Mts., 7/8/1981, J.C.S?????, light trap (3, UAIC); AZ, Pima Co., Sycamore Canyon, Santa Rita Mts., 7/22/1981, J.C.Burne, (2, UAIC); AZ, Madera Canyon, Santa Rita Mts., 7/27/1947, L.M.Martin, (1, HEH ); AZ, Cochise Co., Chiricahua Mts. 5 mi. W of Portal, 6/15/1959, 5400’, L.A.Stange, (3, LACM); AZ, Cochise Co., Chiricahua Mts. South Fork, Cave Creek Camp, 5/20/1966, L.M.Martin, (2, LACM); AZ, Cochise Co., 5 mi. W of Portal, 6/6/1959, L.A.Stange, (1, LACM); AZ, Cochise Co., San Bernardino Ranch 13 mi. E of Douglas, 6/12/1959, L.A.Stange, (6, LACM); AZ, Cochise Co., 2 mi. E of Portal, 1/17/1959, L.A.Stange, (1, LACM); AZ, Gila Co., E.Verde River, 7 mi. N of Payson, 10/26/1959, Truxal & Martin, (1, LACM); AZ, Pinal Co., Oak Flat Cpgd. off US60, 9/18/2010, 33.18.28N 111.03.10W,, Warner & Gruber, Bill Warner, head lamp and UV light, (2, HEH); AZ, Cochise Co., SWRS Chiricahua Mts., 6/27/1960, 5400’, M.Cazier, (1, ASUT); AZ, Cochise Co., SWRS, 6/33/1993, L.R.Davis,Jr., (1, FSCA); AZ, Cochise Co., SWRS Chiricahua Mts., 5 mi. SW of Portal, 8/16-20/2000, 31.54.02N 109.13.39W, 5400’, M.J.Yoder, at lights (1, TAMU); AZ, Santa Rita Mts., 7/7/????, genitalia figured H1920 (1, ANSP); AZ, Santa Rita Mts., 7/17/1932, R.H.Beamer, (1, ANSP); AZ, Madera Canyon, Santa Rita Mts., 5/5/1948, L.M.Martin, (1, LACM); AZ, Madera Canyon, Santa Rita Mts., 8/14/1949, L.M.Martin, (1, LACM); AZ, Santa Cruz Co., Madera Canyon, Santa Rita Mts., 6/22/1955, 5800’, L.M.Martin, (1, LACM); AZ, Madera Canyon, Santa Rita Mts., 8/20/1953, R.J.Ford, (1, LACM); AZ, Box Canyon, Santa Rita Mts., 8/25/1949, L.M.Martin, (1, LACM); AZ, Madera Canyon, Santa Rita Mts., 8/7/1952, Kirkwood & Reid, (1, LACM); AZ, Madera Canyon, Santa Rita Mts., 8/18/1949, L.M.Martin, (1, LACM); AZ, Santa Rita Mts., 10/6/????, Univ. of Kan Lot 968 (2, ANSP); AZ, Madera Canyon, Santa Rita Mts., 7/27/1947, L.M.Martin, (1, LACM); AZ, Madera Canyon, Santa Rita Mts., 8/7/1947, L.M.Martin, (2, LACM); AZ, Madera Canyon, Santa Rita Mts., 9/2/1952, L.M.Martin, (1, LACM); AZ, Madera Canyon, Santa Rita Mts., 9/2/1953, L.Martin, (1, LACM); AZ, SWRS 5 mi. W of Portal, Chiricahua Mts., 6/7/1947, 5400’, J.W.Green, (1, CAS); AZ, Cochise Co., Huachuca Mts., floor of Carr Canyon, 8/8-9/1952, 5400’, Leech & Green, (1, CAS); AZ, Sawmill Canyon, Hualapai Mts., 9/10/1919, O.C.Poling, (2, ANSP); AZ, Madera Canyon, Santa Rita Mts., 7/26/1955, F.X.Williams, (2, CAS); AZ, Pima Co., 7/27/1927, R.H.Beamer, (1, ANSP); AZ, Peppersauce Canyon, Santa Catalina Mts., 8/10/1924, J.O.Martin, (1, CAS); AZ, Peppersauce Canyon, Santa Catalina Mts., 8/15/1924, E.P.VanDuzee, (4, CAS); AZ, Chiricahua Mts., 7/4/1940, L.A.Liporsky, (1, ANSP); AZ, Prescott, 8/21/1917, J.A.Kusche, (1, ANSP); AZ, Santa Rita Mts., E.A.Schwarz, (2, USNM); AZ, Carr Canyon, Huachuca Mts., 8/9/1940, E.S.Ross, (2, CAS); AZ, Portal, 6/17/1956, O.L.Cartwright, (1, USNM); AZ, Ft.Grant, H.G.Hubbard, (2, USNM); AZ, Globe, 8/6/1959, J.Helfer, (1, USNM); AZ, Globe, 8/12/1958, D.K.Duncan, (1, USNM); AZ, Madera Canyon, Santa Rita Mts., 6/17/1898, E.A.Schwarz, (1, USNM); AZ, Huachuca Mts., (1, USNM); AZ, Benson, (1, HEH ); AZ, Santa Rita Mts., 7/26/1925, (1, USNM); AZ, Oracle, (1, USNM); AZ, Pima Co., Stratton, Santa Catalina Mts., 7/27/1917, 6-7000’, Wheeler, (1, USNM); AZ, Huachuca Mts., (2, USNM); AZ, Yavapai Co., Bloody Basin, 9/18/1947, F.H.Parker, (1, HEH); AZ, Yavapai Co., 4 mi. N of Granite Dells, 7/12/1970, L.M.Martin, (3, LACM); AZ, Yavapai Co., Yarnell, Weaver Mts., 6/10/1937, L.K.Gloyd, 110, taken at light (1, UMMZ); AZ, Oracle, 6/16/1967, R.Rice, under stone (3, UAIC); AZ, Bisbee, 10/15/1959, J.M.Kraft, (1, ASUT); AZ, Cochise Co., Portal, 6/18/1964, 4700’, Mortenson & Cazier, at light (1, ASUT); AZ, Catalina Mts., ?/?/1917, 5500’, Cornell U. Lot 892 Sub. 146 (1, CUIC); AZ, Santa Cruz Co., Madera Canyon, Santa Rita Mts., 7/10-26/1964, 5100’, D.R.Davis, (1, USNM); AZ, Pima Co., Madera Canyon, Santa Rita Mts., 5/16/1963, 4400’, J.G.Franclemont, (1, CUIC); AZ, Chiricahua Mts., 7/20/1953, D.J. & J.N. Knull, (1, FSCA); AZ, Madera Canyon, Santa Rita Mts., 6/18-23/1962, 5000’, F.Werner, UV light trap (1, UAIC); AZ, Santa Cruz Co., Madera Canyon, Santa Rita Mts., 7/4/1979, R.H.Crandall, (1, LACM); AZ, Gila Co., Payson, 7/30/1988, 5000’, C.D.Ferris, (1, FSCA); AZ, Cochise Co., Portal, 6/16/1964, 4700’, Puckle,Mortenson & Cazier, at light (1, ASUT); AZ, Cochise Co., Portal, 7/3/1964, 4700’, Puckle,Mortenson & Cazier, at light (4, ASUT); AZ, Cochise Co., Portal, 6/7/1964, 4700’, Puckle,Mortenson & Cazier, at light (1, ASUT); AZ, Cochise Co., 1 mi. S of Portal, 7/31/1965, 4800’, Davidson,Davidson & Cazier, at light (1, ASUT); AZ, Cochise Co., Portal, 7/2/1964, 4700’, Puckle,Mortenson & Cazier, at light (1, ASUT); AZ, Chiricahua Mts., 6/27/1949, D.J. & J.N. Knull, (3, OSUC); AZ, Chiricahua Mts., 7/5/1949, D.J. & J.N. Knull, (1, FSCA); AZ, Chiricahua Mts., 7/9/1959, D.J. & J.N. Knull, (2, FSCA); AZ, Chiricahua Mts., 7/23/1959, D.J. & J.N. Knull, (1, OSUC); AZ, Prescott, 8/22/1917, O.C.Poling, (1, ANSP); AZ, Prescott, 7/14/1904, Kunze, genitalia figured H1920 (1, ANSP); AZ, Prescott, 7/18/1904, Kunze, (2, ANSP); AZ, Chiricahua Mts., 6/15/1939, D.J. & J.N. Knull, (1, OSUC); AZ, Chiricahua Mts., 6/27/1949, D.J. & J.N. Knull, (2, OSUC); AZ, Santa Cruz Co., Sycamore Canyon, 7/3/1974, D.G.Marqua, (1, LACM); AZ, Cochise Co., South Fork, Cave Creek, Chiricahua Mts., 6/1/1964, 5000’, Puckle,Mortenson & Cazier, (1, ASUT); AZ, Stewart For.Camp, Cave Creek Canyon, Chiricahua Mts., 9/13-14/1952, B.Malkin, (1, USNM); AZ, Carr Canyon, Huachuca Mts., 2/25-28/1964, R.F.Sternitsky, (2, PMNH); AZ, Cochise Co., Ramsey Canyon, Huachuca Mts., 9/3/1964, R.F.Sternitsky, (1, PMNH); AZ, Miller Canyon, Huachuca Mts., 6/24/1980, C.A.Olson, (1, UAIC); AZ, Miller Canyon at Tombstone, Huachuca Mts., 6/6/1964, 5800’, J.Burger, (1, UAIC); AZ, Cochise Co., 9 mi. S of MacNeal, 8/30/1958, D.D.Linsdale, (2, FSCA); AZ, Huachuca Mts., 7/20/1937, D.J. & J.N. Knull, (2, OSUC); AZ, Montezuma Pass, Huachuca Mts., 7/6/1956, 6600’, O.L.Cartwright, (1, USNM); AZ, Huachuca Mts., 8/19/1950, D.J. & J.N. Knull, (2, OSUC); AZ, Pima Co., Santa Rita Mts. N end, Rosemont area, McCleary Canyon, 7/15/1975, 5200’, Busacca & Olson, Anamax Mine Inventory, UV light (3, UAIC); AZ, Pima Co., Santa Rita Mts. N end, Rosemont area, Barrel Canyon, 9/10/1975, 4600’, Busacca & Olson, Anamax Mine Inventory, UV light (1, UAIC); AZ, Pima Co., Florida Canyon, Santa Rita Mts., 5/20/1978, M.W.Hetz, (1, UAIC); AZ, Cochise Co., Portal, 8/29-9/3/1974, J.D.Pinto, (1, UCRC); AZ, Oracle, (1, ANSP); AZ, Graham Co., Dripping Spring, Whitlock Mts., 8/5/1976, D.G.Chandler, *Arenivaga* sp. Det. D.G.Chandler (1, UAIC); AZ, Graham Mts., Noon Creek, 8/1/1957, G.D.Butler, (4, UAIC); AZ, Cochise Co., Portal, 6/27/1963, A.Raske, (1, EMEC); AZ, Cochise Co., Cave Creek Ranch, Portal, 8/1-3/1972, E.G.Linsley, (1, EMEC); AZ, Bog Springs Cpgd., Madera Canyon, Santa Rita Mts., 8/1-3/1975, Menke & Pulawski, (3, USNM); AZ, Pima Co., Bog Springs Cpgd., Madera Canyon, 7/10/1976, Doug Whitman, #575, black light (2, EMEC); AZ, Palmerlee, Banks, erratica (1, MCZ); AZ, Santa Cruz Co., Madera Canyon, Santa Rita Mts., 7/4/1979, R.H.Crandall, (1, LACM); AZ, San Bernardino Ranch 20 mi. E of Douglas, 7/7/1947, E.R.Tinkham, E.R.T., at light, (2, HEH); AZ, Maricopa Co., Wickenberg, 6/14/1963, J Doyen, (1, EMEC); AZ, Maricopa Co., Wickenberg, 8/20/1938, DJ & JN Knull, (1, OSUC); AZ, Cochise Co., Guadalupe Canyon 29 mi. E of Douglas, 8/15-16/1972, J Doyen, Black light trap (2, EMEC); AZ, Cochise Co., Chihuahua Mts. Tex Canyon, 9/8/1927, 5-600 ft., JA Kusche, (1, CAS); NM, Hidalgo Co., Cienega Lake,12.2 mi. N of jct. Portal Rd. & Hwy. 80, 8/9/1973, SI & SL Frommer, UV light, 7.20-10.30 pm (1, UCRC); NM, Cave Creek, 7/3/1947, H.S.Wallace, (2, SEMC); NM, Hidalgo Co., Cienaga Ranch nr. Rodeo, 7/12/1948, C & P Vaurie, (1, AMNH); NM, Hidalgo Co., Pelencio Mts., 7/23/1981, Olson & Thomas, (1, UAIC); AZ, Pima Co., Mt. Lemmon RA, Catalina Hwy. milepost 11.3, 6/11/2012, 32 22 21.0, 110 41 40.2, 5840 ft., DB Weissman, pine forest and shrubs (2, HEH); AZ, Mohave Co., Hualapai Mt. Rd. E of Kingman, milepost 12.5, 6/13/2012, 35 05 26.9, 113 52 17.8, 6000 ft., DB Weissman, pines, (1, HEH); AZ, Chiricahua Mts., Cave Creek Cnyn., Stewart Campground, 8/7/1974, GH Nelson, (1, FSAC); NM, Virden, 6/26/1963, R Enzie, (1, NMSU); AZ, Cochise Co., Tex. Can., 10/24/1958, 4600’, G & A Ferguson, (1, FSAC); AZ, Cochise Co., Tex. Can., 10/5/1960, GR Ferguson, (1, FSAC); AZ, Cochise Co., nr. Double Adobe, 6/28/1977, S McCleve, lite (1, FSAC); AZ, Cochise Co., near Fairbanks, 6/29/1973, S McCleve, lite (1, FSAC); AZ, Cochise Co., San Pedro R, nr. Fairbanks, 6/29/1973, S McCleve, lite (1, FSAC); AZ, Cochise Co., SWRS 5 mi W Portal, 5/2/1967, 5400’, VD Roth, (1, SWRS); AZ, Cochise Co., SWRS 5 mi W Portal, 7/8/1964, VD Roth, *Arenivaga* infuscata (Caud) (1, SWRS); AZ, Cochise Co., SWRS 5 mi W Portal, 5/23/1964, VD Roth, (1, SWRS); AZ, SWRS, 5/12/1956, (1, SWRS). Determiner label *Arenivaga apacha* Hopkins 2011” [white label with black border].

##### Distribution.

*Arenivaga apacha* is found in in the entire southeastern portion of Arizona and northwestwards from there. The appearance on the map that the distribution ends at the Mexican and New Mexican borders is without doubt a collection artifact. See Fig. 23.

##### Diagnosis.

*Arenivaga apacha* may be diagnosed by forward and backward facing spines, one on each end of the medial margin of the right dorsal phallomere. There is also a prominent spine on the right ventral phallomere. See Fig. 22.

##### Description.

**Male.** NB: Ventral surface of holotype shellacked. *Measurements*. Holotype TL = 20.9 mm, GW = 9.7 mm, PW = 6.07 mm, PL = 4.27 mm, TL/GW = 2.15, PL/PW = 0.70. EW = 0.40 mm; OW = 0.40 mm. Among paratypes range of TL 16.7–23.0 mm; range of GW 7.3–10.4 mm; range of PW 5.06–6.30 mm; range of PL 3.98–4.90 mm.

**Figure 21.**
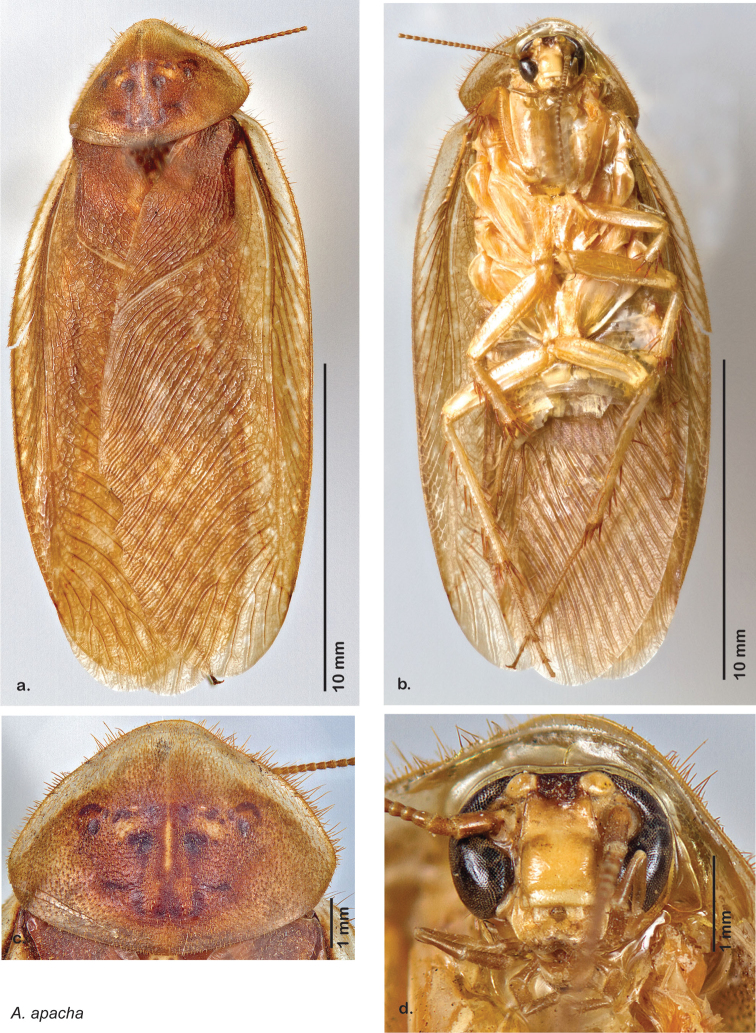
*Arenivaga apacha*, **a** dorsal habitus **b** ventral habitus **c** pronotum **d** head.

*Head*. Two ocelli large, ovoid and protruding (0.35 × 0.25 mm); vertex medium brown, with small ridges between apices of eyes and extending onto ocellar tubercles; interocellar space concave, medium brown. Frons light brown, concave with occasional very long setae; bound on either side by ridges extending from inner apex of ocelli outwards to lateral edges of clypeus. Anterior portion of frons light brown, bulbous; clypeal suture demarcates light brown anteclypeus. See Fig. 21d.

*Pronotum*. Pronotum translucent waxy beige, often only along anterior margin depending on specimen with remainder of pronotum medium orange-brown; variable length orange-brown setae along anterior margin; dorsal surface of pronotum covered with short orange-brown setae; pronotal pattern “panther face” ranging from light brown through every shade of orange-brown and brown with many shades of aura, usually extensive, depending on specimen; detail ranges from clear to indiscernible. See Fig. 21c.

*Body*. Wing brace present. Legs and body light orange-brown; subgenital plate light orange-brown; asymmetrical with posterior edge emarginated, rounded apices. See Fig. 21b.

*Forewings*. Wings extended beyond abdominal apex (up to ~40% of total wing length); dark-orange brown densely blotchy; majority of specimens with medium to dark brown or orange-brown densely blotchy wings; occasional specimens light or very dark, or with uniform coloration; surface opaque and matte. See Fig. 21a.

**Figure 22.**
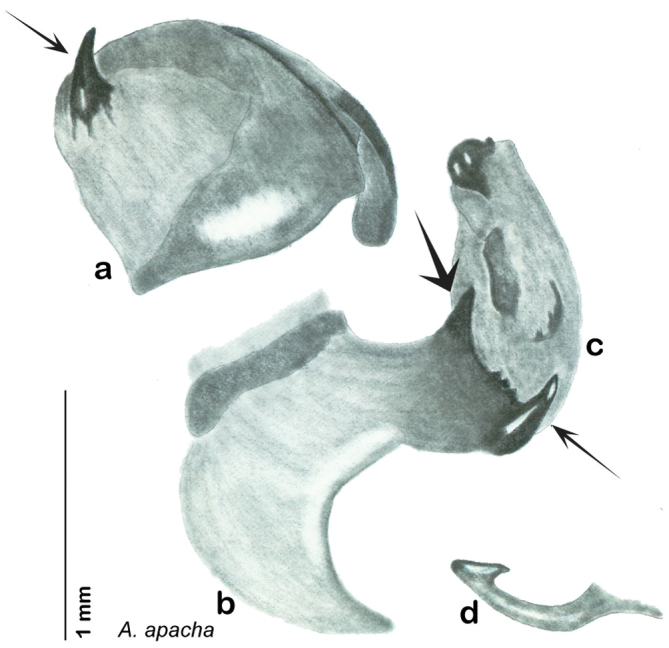
*Arenivaga apacha*, genitalia: **a** right dorsal phallomere **b** right ventral phallomere **c** small central sclerite **d** genital hook. Arrow(s) indicate diagnostic characters (see text).

*Genitalia*. Right dorsal phallomere composed of bulbous lightly sclerotized narrow hook-shaped lobe, articulated with right ventral phallomere on lateral side; central field broad, slightly sclerotized; medial margin heavily sclerotized, sinuous, with toothed edge; long posteriorly projecting spine and shorter anterior spine; anterior spine of varying sizes depending on specimen. Teeth along medial edge may be variously lengthened depending on specimen. Small central sclerite deeply concave, punctate, with medially projecting punctate lobe on ventral end. Right ventral phallomere extends from articulation into smooth lobe with prominent medially projecting spine at posteroventral corner; increasingly punctate and sclerotized anteriorly; after narrow gap, wide rounded concave shagreened arm extending beyond the depth of rest of phallomere. Folded anterior portion of left phallomere narrow, trifold, punctate, otherwise unmodified. Genital hook (missing from holotype) with moderate extension to pointed head with slight concavity on moderate hook; arm narrow and smoothly curved. See Fig. 22.

**Figure 23.**
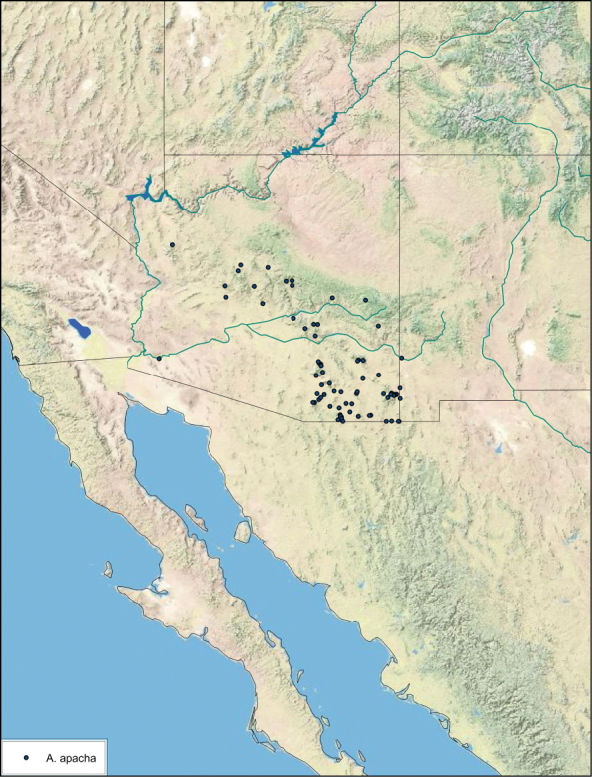
*Arenivaga apacha*, distribution.

##### Habitat and natural history.

*Arenivaga apacha* dig shelves in the mounds of *Dipodomys spectabilis* which they then line with materials taken from the mounds of the kangaroo rats (Cohen and Cohen 1976). They were also observed to cache seeds acquired from the rodent mounds, most particularly *Atriplex* seeds. When Cohen and Cohen measured the temperature of the cockroach shelves they were found to remain at an average of 16.5 degrees Celsius when the surrounding surface soil temperatures were as high as 60 degrees Celsius and never lower than 30 degrees Celsius. Additionally the humidity in the lined cockroach shelves was never found to be lower than 91% while that of the surrounding burrow was as low as 20%. This species of *Arenivaga* has found a way to create an ideal temperature and humidity controlled environment in an otherwise harsh climate. They also use the materials collected by the kangaroo rats for a larder.

#### Arenivaga apaeninsula sp. n.

http://zoobank.org/E5D8D314-BF5F-421E-98ED-FBD3DA74FE4C

http://species-id.net/wiki/Arenivaga_apaeninsula

Figures 24 – 26

##### Type locality.

MEXICO, BCS, 7 mi SW La Paz.

##### Material examined.

Holotype: ♂ in EMEC labeled “MEX: Baja Calif. Sur, La Paz, 7 mi. SW, VIII-2-66” “HOLOTYPE *Arenivaga apaeninsula* Hopkins, 2012” [red label with black border].

Paratypes (6): MEXICO: BCS, 7 mi SW of La Paz, 8/2/1966, Linsley,Chemsak & Hurd, at light (6, EMEC). All paratypes labeled “Paratype *Arenivaga apaeninsula* Hopkins 2012” [blue label with black border].

##### Etymology.

This species is named from the Latin meaning “from a peninsula” because all known specimens are from La Paz, BCS, Mexico.

##### Distribution.

This species is known only from the type locality in Baja California Sur, Mexico. See Fig. 26.

##### Diagnosis.

**Figure 24.**
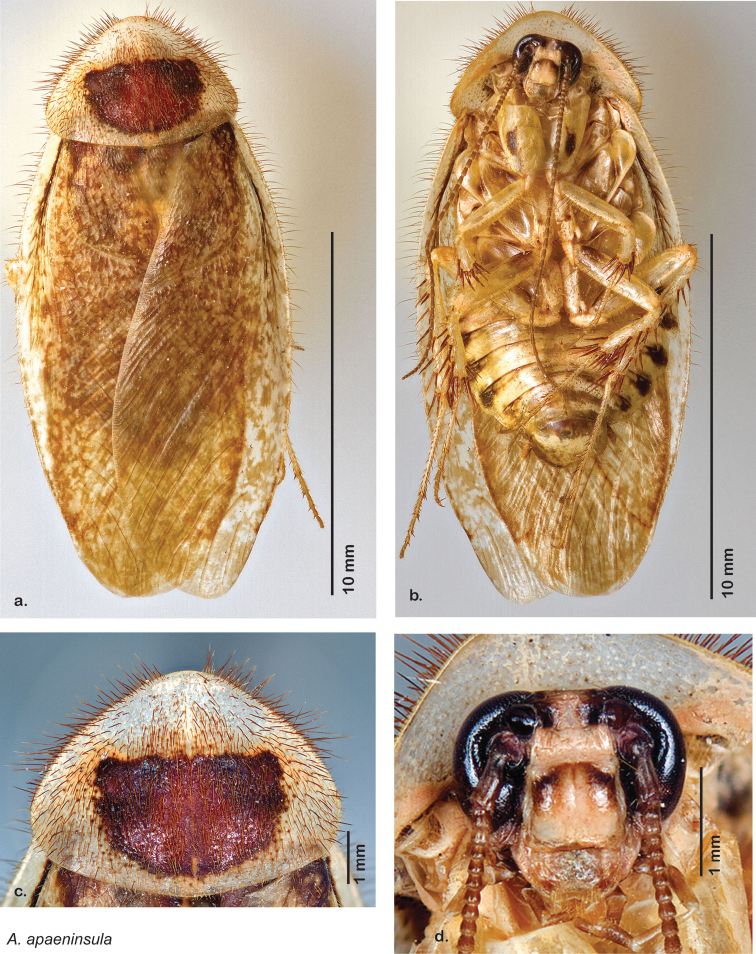
*Arenivaga apaeninsula*, **a** dorsal habitus **b** ventral habitus **c** pronotum **d** head.

*Arenivaga apaeninsula* may be distinguished by the dense brown setae on the pronotum and dark red-brown pronotal pattern with no discernible detail. Its striking pronotum and restricted range combine to make it easily diagnosed. See Figs 24c and 26.

##### Description.

**Male.** *Measurements*. Holotype TL = 16.1 mm, GW = 7.6 mm, PW = 5.48 mm, PL = 4.17 mm, TL/GW = 2.12, PL/PW = 0.76. EW = 0.50 mm; OW = 0.55 mm. No notable size differences among paratypes.

*Head*. Two ocelli large, ovoid and protruding (0.35 × 0.25 mm); vertex light brown with narrow dark brown band around apex of eyes and ocelli; with small ridges in rays around upper apices of eyes and extending onto ocellar tubercles; interocellar space concave, dark brown, light brown medially. Frons medium brown; posterior tectiform horizontally; anterior portion of frons bulbous, medium brown fading to light brown anteriorly; narrow light brown anteclypeus. See Fig. 24d.

*Pronotum*. Pronotum small, opaque waxy white; dorsal surface of pronotum with dense brown setae that are thicker and longer laterally; pronotal pattern dark red-brown “panther face”, not impressed, with no discernible detail; scattered brown freckles bordering pronotal pattern; no aura. See Fig. 24c.

*Body*. Wing brace absent. Legs and body light brown; large dark brown maculations on ventral surface of procoxa; medium brown maculations at proximal end of meso and meta femurs, located ventrally and wrapping dorsally; scattered medium brown maculations on mesocoxa. Sternites with dark brown maculations laterally on each; subgenital plate light brown with darker border and rounded apices. See Fig. 24b.

*Forewings*. Wings extended well beyond abdominal apex (~30% of wing length); medium to dark brown blotches; surface opaque and matte. See Fig. 24a.

**Figure 25.**
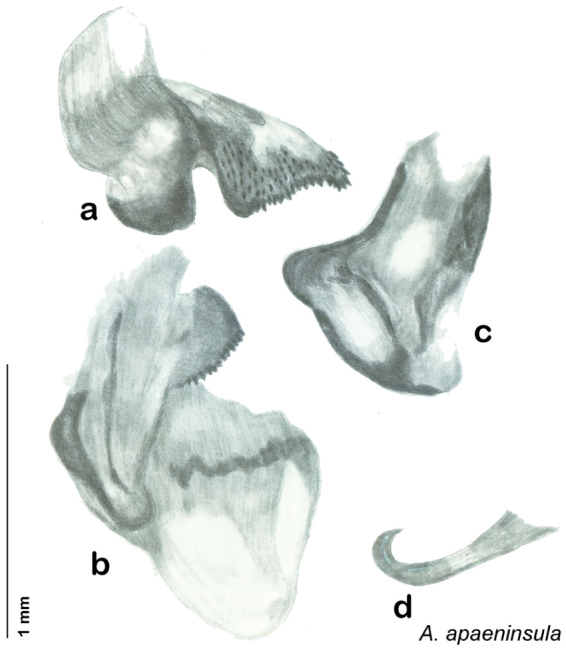
*Arenivaga apaeninsula*, genitalia: **a** right dorsal phallomere **b** right ventral phallomere **c** small central sclerite **d** genital hook. Arrow(s) indicate diagnostic characters (see text).

*Genitalia*. Right dorsal phallomere composed of lightly sclerotized, bulbous pointed lobe, articulated with right ventral phallomere on lateral side; central field lightly sclerotized. Small central sclerite so slight and transparent as to be virtually non-existent, unmodified. Articulation between right phallomere dramatically modified with smooth rounded knob posteriorly extending into raised ridge and then to shagreened or toothed flange anteriorly; right ventral phallomere extends from articulation to form shagreened rounded structure; after moderate gap, wide toothed flange with central concavity and rounded apices that extends to depth of rest of phallomere. Folded anterior portion of left phallomere dramatically modified into 3D triangle with rounded edges and concave surfaces; medial surface has deep concave dimple. Genital hook a wide sweeping curve with straight arm. See Fig. 25.

**Figure 26.**
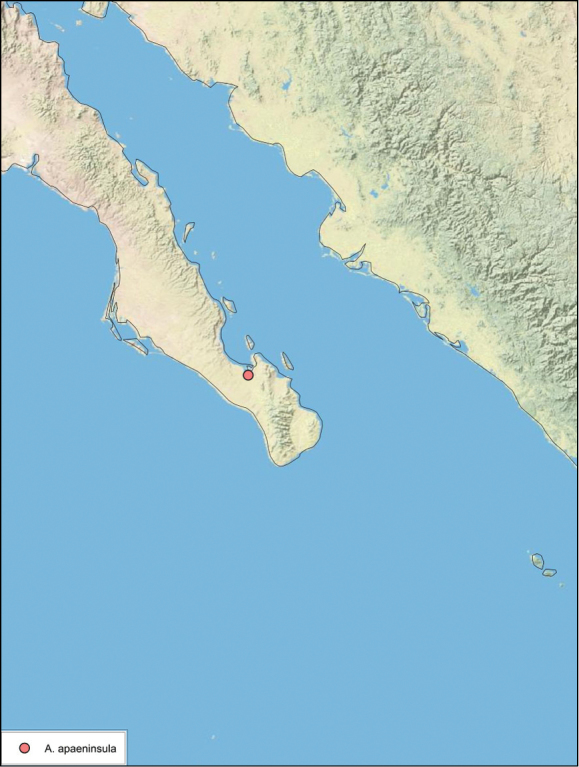
*Arenivaga apaeninsula*, distribution.

##### Habitat and natural history.

All life history elements remain unobserved.

#### Arenivaga aquila sp. n.

http://zoobank.org/31D849C4-8517-4A26-AA2E-31B2345DC052

http://species-id.net/wiki/Arenivaga_aquila

Figures 27 – 29

##### Type locality.

MEXICO, Morelos, 7.3 mi S of Yautepec.

##### Material examined.

Holotype: ♂ in SEMC labeled “MEXICO Morelos, 7.3 mi. S. Yautepec, 3000’, 17 Aug. 1962, Ordway & Roberts, *Arenivaga* sp. nr. *bolliana* (Saussure) Det. F.W.Fisk ‘80” “HOLOTYPE *Arenivaga aquila* Hopkins, 2012” [red label with black border].

Paratypes (23): MEXICO: Guerrero, Xalitla, 8 km N of Mezala, 9/17-23/1982, 580 m, Powell & Chemask (1, EMEC); Guerrero, 2.1 mi. NW of Cacahuamilpa, 8/10/1980, Schaffner, Weaver & Friedlander (2, TAMU); Guerrero, 10.3 mi. S of Iguala, 7/23/1981, Bogar,Schaffner &Friedlander (2, TAMU); Guerrero, 6.2 mi. SW of Xochipala, 7/6/1987, 5670 ft., Kovarik & Schaffner (1, TAMU); Guerrero, Cacahuamilpa, 9/11/1964, B.Rotger (1, UCMC); Morelos, 7.3 mi. S of Yautepec, 7/30/1963, 3300 ft., GW Byers (6, SEMC); Morelos, Alpuvec, 8/18/1957, WW Gibson, HIERBA, Rockefeller Collection, return to Cantrell (1, UMMZ); Morelos, 6.7 mi. S of Yautepec, 7/29/1963, Naumann & Willis, *Arenivaga* nr. *bolliana* det. FW Fisk 1980, 135 (1, SEMC); Morelos, Canada de Lobo, 20 km E of Cuernavaca, 7/7/1981, EM Fisher (1, CSCA); Morelos, Barrio de las Piedras #37, Jiutepec, 7/1/1998, E Brambila, under a rock (1, FSAC); Morelos, Canada de Lobo, 9/18/1964, B.Rotger (1, UCMC); Morelos, Iguala, *Arenivaga* nr. or = *rehni* teg:miaculat. as in *grata* det. Hebard 1931 (1, ANSP); Guerrero, Coacoyula, 10/24/1942, WF Foshag (2, USNM); Morelos, Barrio de las Piedras #37, Jiutepec, 6/28/1998, J Brambila, flying (1, FSAC); Morelos, Jojula, ?/?/1929, JJ White, Hebard Collection (1, ANSP). All paratypes labeled “Paratype *Arenivaga aquila* Hopkins 2012” [blue label with black border].

##### Etymology.

The name is an adjective in the nominative singular. This species is named from the Latin meaning dark-colored or swarthy because of its very dark color.

##### Distribution.

This species is found in the states of Morelos and Guerrero, Mexico. See Fig. 29.

##### Diagnosis.

*Arenivaga aquila* may be distinguished by the small ridge projecting from the ventrolateral edge of the folded portion of the left phallomere. See Fig. 28.

##### Description.

**Male.** *Measurements*. Holotype TL = 19.1 mm, GW = 8.4 mm, PW = 6.00 mm, PL = 4.17 mm, TL/GW = 2.27, PL/PW = 0.70. EW = 0.15 mm; OW = 0.40 mm. Among paratypes range of TL 18.0–21.5 mm; range of GW 8.2–10.1 mm; range of PW 5.60–6.74 mm; range of PL 3.94–4.59 mm.

**Figure 27.**
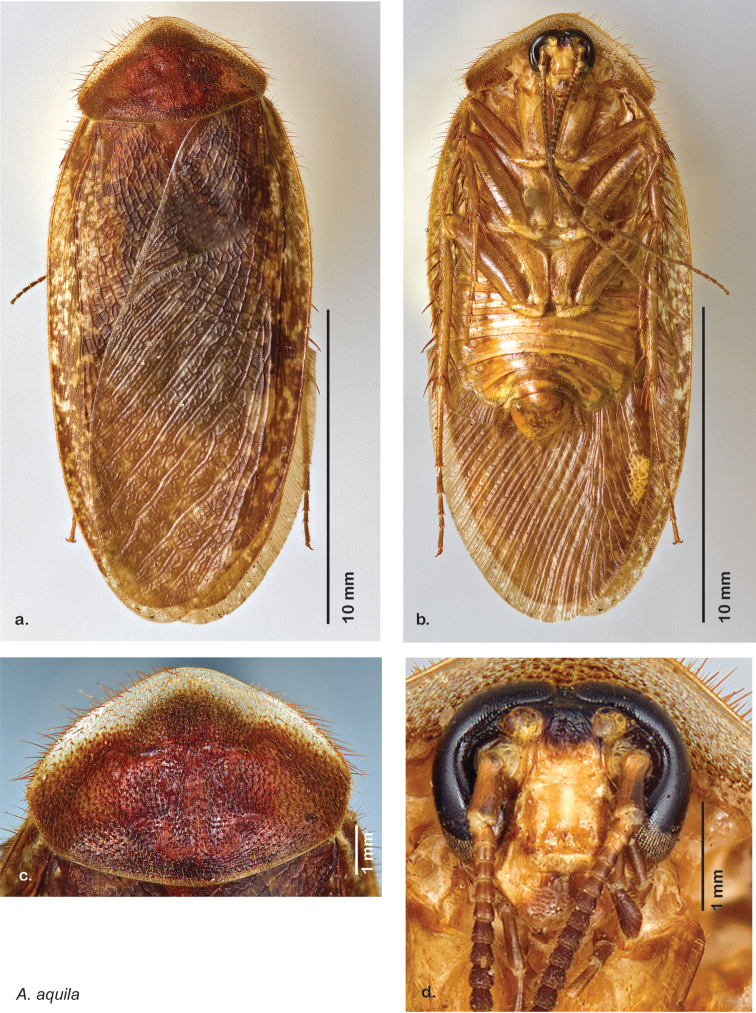
*Arenivaga aquila*, **a** dorsal habitus **b** ventral habitus **c** pronotum **d** head.

*Head*. Two ocelli large, ovoid and protruding (0.40 × 0.30 mm); vertex dark brown, with small ridges between apices of eyes and extending onto ocellar tubercles; interocellar space concave, dark brown, with two small oval indentations. Frons light brown; posterior concave; anterior portion of frons bulbous but much less so than in most species, light brown; light brown anteclypeus. See Fig. 27d.

*Pronotum*. Pronotum with translucent waxy beige anterior margin; aura so extensive that remainder of pronotum dark orange-brown and dark brown; pronotal pattern impressed “panther face”, difficult to discern. See Fig. 27c.

*Body*. Wing brace absent. Legs and body medium orange-brown; subgenital plate orange-brown; strongly asymmetrical with angular apices. See Fig. 27b.

*Forewings*. Wings extended well beyond abdominal apex (up to ~40% of wing length); blotchy dark brown to solid dark brown depending on specimen; surface matte and opaque. See Fig. 27a.

**Figure 28.**
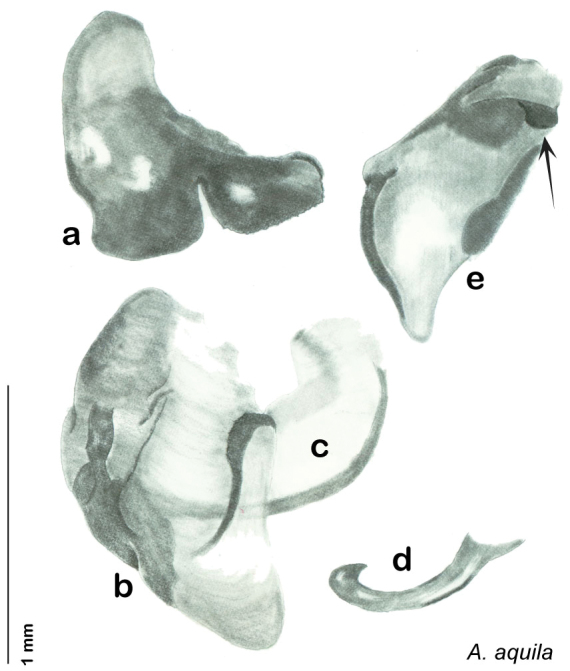
*Arenivaga aquila*, genitalia: **a** right dorsal phallomere **b** right ventral phallomere **c** small central sclerite **d** genital hook **e** left phallomere. Arrow(s) indicate diagnostic characters (see text).

*Genitalia*. Right dorsal phallomere composed of lightly sclerotized, short, narrow, bulbous lobe, articulated with right ventral phallomere on lateral side; central field lightly sclerotized, cupped; with narrow medial edge more sclerotized, punctate, ending anteriorly in small shagreened flange. Small central sclerite lightly sclerotized, finely punctate, flat of no discernible shape, posterior end connecting with dorsal side of right dorsal phallomere. Right ventral phallomere arises from deep articulation to form large punctate flattened medially projecting lobe; becoming wider and more sclerotized anteriorly; after narrow gap, wide shagreened flange. Folded anterior portion of left phallomere wide, setose, closed at both ends, with small nipple at one end of fold, and rough-edged, flattened projection offset at an angle from other end. Genital hook with short extension to rounded head and short hook; arm robust and smoothly curving. See Fig. 28.

**Figure 29.**
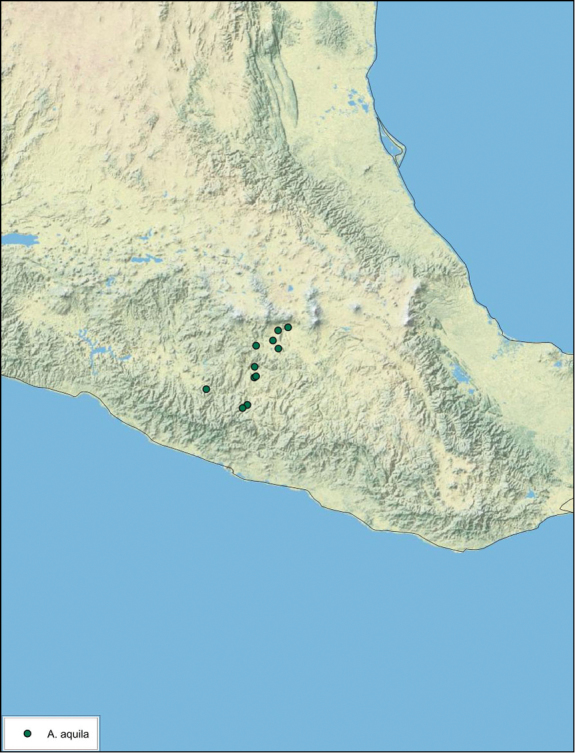
*Arenivaga aquila*, distribution.

##### Habitat and natural history.

All life history elements remain unobserved.

#### Arenivaga belli sp. n.

http://zoobank.org/C91E2047-7CE9-4610-AFD2-C30629249110

http://species-id.net/wiki/Arenivaga_belli

Figures 30 – 32

##### Type locality.

USA, California, San Bernardino Co., Granite Mountains, Cottonwood Wash.

##### Material examined.

Holotype: ♂ in LACM labeled “CALIF: San Bdno. Co., Granite Mts., #148623, Cottonwood Wash, 4000’ T9N, R13E, SE cor. S.31, 14-15 September 1990, J.P. & K.E.S. Donahue “HOLOTYPE *Arenivaga belli* Hopkins, 2012” [red label with black border].

Paratypes (83): USA: CA, San Bernardino Co., Mescal Range, ~2 air mi S of Mountain Pass, 7/10/1982, 4900 ft., JP & KE Donahue (2, LACM); CA, San Bernardino Co., Keystone Canyon, New York Mts., 9/3/1959, FP Sala (4, LACM); CA, San Bernardino Co., Mex.Well, Ivanpah Mts., 9/1/1945 (2, LACM); CA, San Bernardino Co., New York Mts., 8.5 mi S of Ivanpah, 9/11-12/1955, 5000 ft., CD MacNeill (2, EMEC); CA, San Bernardino Co., Granite Mts., Cottonwood Wash, 9/14-15/1990, 4000 ft., JP & KE Donahue (2, LACM); CA, San Bernardino Co., New York Mts., 9/11/1911?, RC Osburn (1, FSCA); CA, Lone Pine, 7/28/1940, DE Hardy (1, SEMC); CA, Kern Co., Shafter, 8/4/1957, LA Stange (1, LACM); UT, Kane Co., 5 mi N and 40 mi E of Kanab, 3/?-9/?/1985, D Giuliani, Antifreeze pit trap (1, CSCA); UT, Hanksville, WD Stanton (1, SDMC). All paratypes labeled “Paratype *Arenivaga belli* Hopkins 2012” [blue label with black border].

##### Etymology.

The name is a noun in the genitive case. This species is named for the late Dr. William Bell, who spent much time exploring the chemical ecology of social insects such as cockroaches and co-authored “Cockroaches: Ecology, Behavior and Natural History”.

##### Distribution.

This species is distributed from Lone Pine, CA in the west to Hanksville, UT in the north and east, and the Rice Dunes in the south. See Fig. 32.

##### Diagnosis.

*Arenivaga belli* is average in size and color for *Arenivaga* and very similar to *Arenivaga nalepae* and *Arenivaga milleri*. *Arenivaga belli* has the same right ventral phallomere as *Arenivaga milleri* (see Figs 31 and 103); it has a right dorsal phallomere very similar to *Arenivaga nalepae* in its medial margin, but the hook-shaped bulges are quite different (see Figs 31 and 112). Therefore a combination of the right dorsal phallomere and angular hook-shaped bulge as shown in Fig. 31 are what distinguish *Arenivaga belli*.

##### Description.

**Male.** *Measurements*. Holotype TL = 19.2 mm, GW = 9.4 mm, PW = 5.47 mm, PL = 3.66 mm, TL/GW = 2.04, PL/PW = 0.67. EW = 0.2 mm; OW = 0.3 mm. Among paratypes range of TL 17.7–21.2 mm; range of GW 7.6–9.4 mm; range of PW 5.32–6.38 mm; range of PL 3.66–4.33 mm.

**Figure 30.**
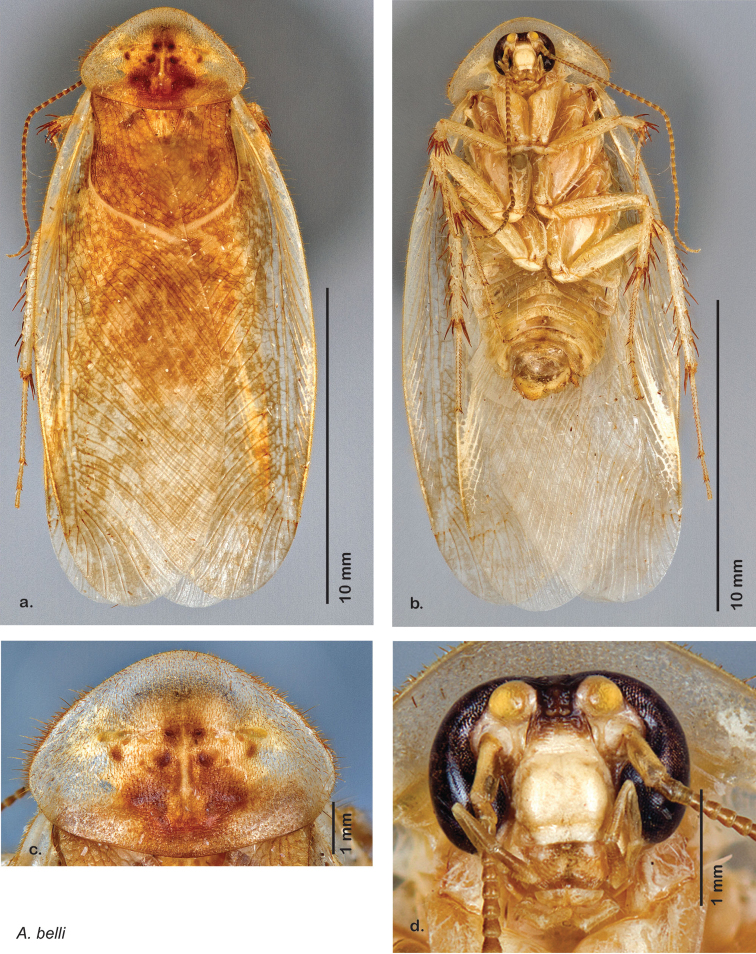
*Arenivaga belli*, **a** dorsal habitus **b** ventral habitus **c** pronotum **d** head.

*Head*. Two ocelli large, ovoid and protruding (0.40 × 0.30 mm); vertex medium brown with small ridges in rays around upper apices of eyes and extending onto ocellar tubercles; interocellar space concave, medium brown, with two deep set dimples medial to inner apex of ocelli; with two smaller dimples anterior to those which may be very hard to see; rugose in some specimens. Frons light brown fading to waxy white towards margin with clypeus; concave with small vertical corrugations. Clypeus waxy white and bulbous; ends in broad flat anteclypeus of same color. See Fig. 30d.

*Pronotum*: Pronotum translucent, waxy beige; dorsal surface of pronotum with short fine orange-brown setae centrally and posteriorly grading to longer, thicker setae laterally and anteriorly; pronotal pattern orange-brown “panther face” with little discernible detail in most specimens; no aura. See Fig. 30c.

*Body*. Wing brace present. Legs and body light orange-brown, subgenital plate with darker margin, strongly asymmetrical with rounded apices. See Fig. 30b.

*Forewings*. Wings extended well beyond abdominal apex (~40% of wing length); color uniform brown, to blotchy brown; surface matte and opaque. See Fig. 30a.

**Figure 31.**
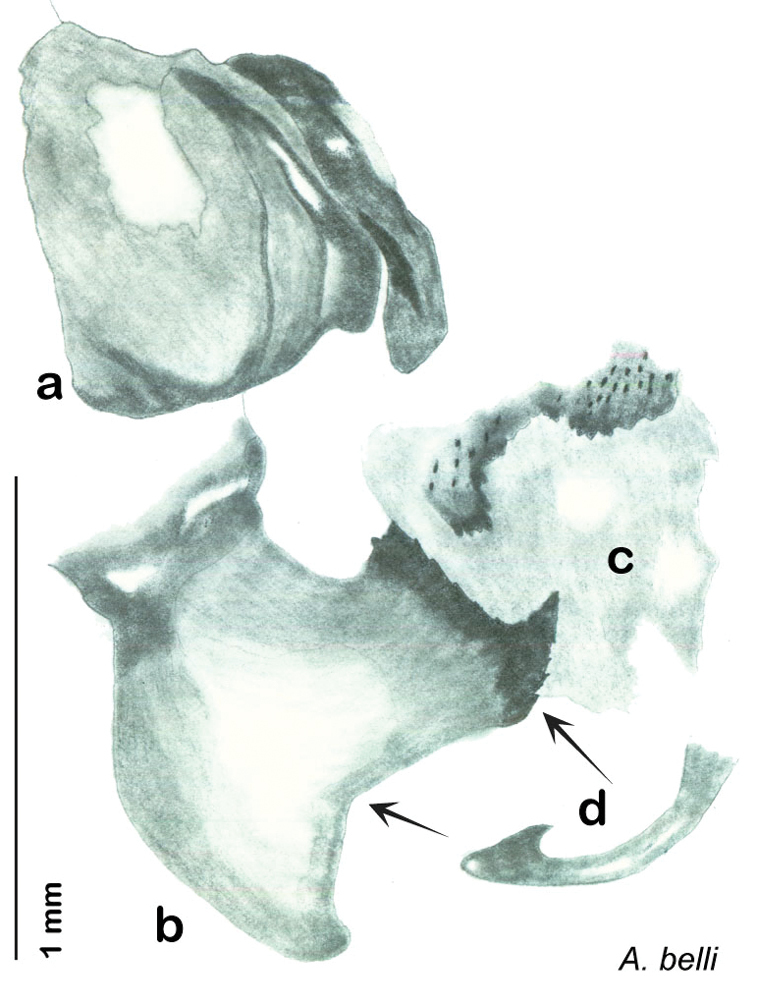
*Arenivaga belli*, genitalia: **a** right dorsal phallomere **b** right ventral phallomere **c** small central sclerite **d** genital hook. Arrow(s) indicate diagnostic characters (see text).

*Genitalia*. Right dorsal phallomere composed of bulbous lightly sclerotized hook-shaped lobe, articulated with right ventral phallomere on lateral side; central field broad, lightly sclerotized; medial margin heavily sclerotized, shagreened, with toothed edge extending into short spine near distal end. Small central sclerite flat and finely punctate with posteriorly projecting, shagreened crescent with elevated and toothed arms; right ventral phallomere extends from articulation to form rounded punctate structure at posterior apex but with shagreened corrugations at anterior apical end, followed by smaller offset shagreened projection, narrow gap, and rounded concave angled arm extending beyond depth of rest of phallomere. Folded anterior portion of left phallomere setose, otherwise unmodified. Genital hook with long extension to pointed head with slight concavity on short hook; arm smoothly curved. See Fig. 31.

**Figure 32.**
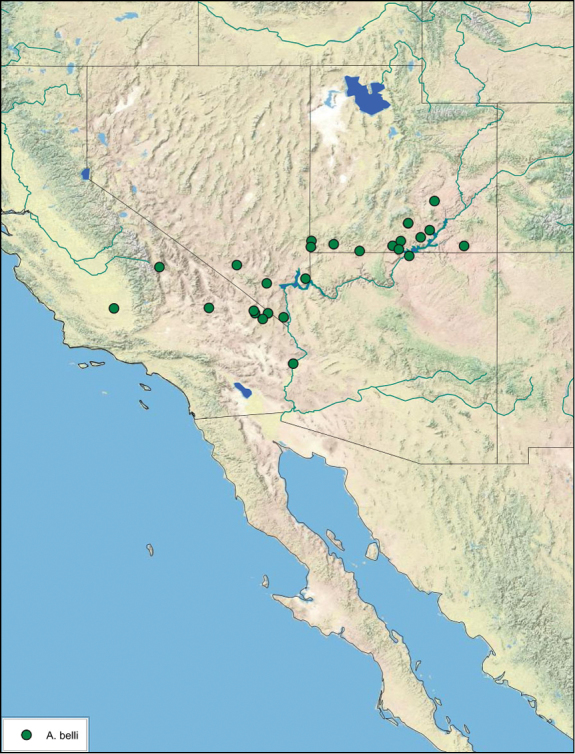
*Arenivaga belli*, distribution.

##### Habitat and natural history.

All life history elements remain unobserved.

#### Arenivaga bolliana

(Saussure)

http://species-id.net/wiki/Arenivaga_bolliana

Figures 33 – 35

Homoeogamia bolliana Saussure 1893, Revue Suisse de Zoologie, I, Fasc. 2, p.296. [Texas.]
Homoeogamia bolliana , Saussure and Zehntner 1894, Biol. Cent.-Amer., Orthopt., I, pp. 107. [New Mexico; Texas.]
Homoeogamia bolliana , Scudder 1900, Proc. Davenport Acad. Natu. Sci., VIII, p. 11. [Texas; New Mexico.]
Homoeogamia bolliana , Rehn 1902, Trans. Amer. Ent. Soc., XXVII, p. 331. [Round Mountain, Texas.]
Homoeogamia bolliana , Scudder and Cockerell 1902, Proc. Davenport Acad. Sci., IX, p. 19. [New Mexico; Las Cruces, NM.]
Homoeogamia (Arenivaga) bolliana Rehn 1903, Proceedings of the Academy of Natural Sciences of Philadelphia, Vol. 55, p. 188.
Homoeogamia (Arenivaga) bolliana var. *nigricans* Caudell 1904, Mus. Brooklyn Inst. A. & S. Sci. Bull., i. p. 107. [Esperanza Ranch, Brownville, Texas.]
Arenivaga bolliana (Saussure) Hebard 1917, Memoirs of the American Entomological Society, No. 2, pp. 223–227.
Arenivaga bolliana (Saussure), Hebard 1920, Transactions of the American Entomological Society, Vol. 46, pp. 201–203.

##### Material examined

**(294).** USA: TX, Abilene, 8/7/1973 (1, UCRC); TX, Uvalde Co., Garner S.P. 8 mi. N of Concan, 10/2-4/2002, 1800’, J.B.Heppner (2, FSCA); TX, Gillespie Co., Lange’s Mill, 6/5/1969, Board & Hafernik (1, TAMU); TX, San Ygnacio, 10/10/1999, W.F.Chamberlain (2, TAMU); TX, Kerr Co., Kerrville, 9/5/1964, W.F.Chamberlain (4, TAMU); TX, Kerr Co., Kerrville, 9/19/1964, W.F.Chamberlain (1, TAMU); TX, Kerr Co., Kerrville, 9/19/1962, W.F.Chamberlain (2, TAMU); TX, Kerr Co., Kerrville, 9/24/1998, W.F.Chamberlain (1, TAMU); TX, Kerr Co., 4 mi. N of Kerrville, 6/11/2004, W.F.Chamberlain (2,TAMU); USA, TX, Kerr Co., 4 mi. N of Kerrville, 6/4/2004, W.F.Chamberlain, (1, TAMU); USA, TX, Kerr Co., Kerrville, 9/16/2001, W.F.Chamberlain, (1, TAMU); USA, TX, Kerr Co., Kerrville, 8/9/1988, W.F.Chamberlain, (2, TAMU); USA, TX, Kerr Co., Kerrville, 6/16/1997, W.F.Chamberlain, (2, TAMU); USA, TX, Kerr Co., Kerrville, 10/16/1996, W.F.Chamberlain, (1, TAMU); USA, TX, Kerr Co., 6 mi. N of Kerrville, 8/17/1996, W.F.Chamberlain, (1, TAMU); USA, TX, Kerr Co., Kerrville, 3/25/2000, W.F.Chamberlain, (1, TAMU); USA, TX, Kerr Co., Kerrville, 6/2/1997, W.F.Chamberlain, (1, TAMU); USA, TX, Kerr Co., Kerrville, 9/20/1960, W.F.Chamberlain, (1, TAMU); TX, Kerr Co., Kerrville, 5/11/1992, W.F.Chamberlain, (1, TAMU); TX, Kerr Co., Kerrville, Guadalupe R., 5/25/1983, Olson, Thomas & Burne, (2, UAIC); TX, Coryell Co., Mother Neff S.P., 12 mi. W of Eddy, 7/17/1962, U KS Mex. Exped., (1, SEMC); TX, Morris Co., 8/22/1960, Cohn & Triplehorn, (1, FSCA); TX, Brazos Co., 11/11/1959, (1, TAMU); TX, Leon Co., 5 mi. N of Flynn, 5/25/1995, E.G.Riley, (1, TAMU); TX, Leon Co.,. 5 mi. SW of Oakwood, 6/29/2000, 31°34'7"N, 95°51'42"W, Godwin & Riley, (1, TAMU); TX, Leon Co., 5 mi. N of Flynn, 5/27/1994, E.G.Riley, (1, TAMU); TX, Maverick Co., Q.L.Nguyen, (1, TAMU); TX, Leon Co., 4 mi. NW Normangee, 9/27-10/6/2001, 31°04'N, 96°09'W, J.Yantis-59, (2, TAMU); TX, Leon Co., 5 mi. N of Flynn, 5/24/1994, E.Riley, (3, TAMU); TX, Wood Co., ca. 15 mi. N of Hawkins, 4/29/2000, 32°98'42"N, 95°10'04"W, W.Godwin, (3, TAMU); TX, Wood Co., Hawkins, 4 mi. N Jct 14 & 2869, 6/8-22/1996, W.Godwin, (1, TAMU); TX, Wood Co., ca. 18 mi. N of Hawkins, 5/14/1999, Yoder & Godwin, (1, TAMU); TX, Henderson Co., Cross Roads, 5/31/2001, E.G.Riley, (1, TAMU); TX, Dimmit Co., 9/10/1933, S.E.Jones, (1, ANSP); TX, Burleson Co., Lake Somerville, 8/9/1979, P.W.Kovarik, (1, TAMU); TX, Comal Co., Bulverde, 6/15-16/1996, Warner & Wappes, Bill Warner, (1, WB Warner); TX, Kleberg Co., 1 mi. SE of Kingsville, 6/9/1989, Schaffner, (1, TAMU); TX, Kleberg Co., Kingsville, C.T.Reed, (1, CUIC); TX, Anderson Co., Engeling WMA, 6/3/1995, E.G.Riley-130, (1, TAMU); TX, Three Rivers, 6/27/1938, D.W.Craik, (1, ANSP); TX, Kleberg Co., Kingsville, C.Reed, (1, ANSP); TX, San Patricio Co., Corpus Christi Lk. S.P., 8/18/1963, G.W.Byers, (2, SEMC); TX, Uvalde Co., 5/19/1918, J.C.Bradley, (1, ANSP); TX, Big Bend Reg., Summer 1928, F.F.Bibby, (1, ANSP); TX, Kerr Co., Kerrville, 9/14/1990, W.F.Chamberlain, (1, TAMU); TX, LaSalle Co., Chaparral WMA, 9/29-30/1989, J.Schaffner, (5, TAMU); TX, Kerr Co., Kerrville, 9/4/1964, W.F.Chamberlain, (1, TAMU); TX, Dimmit Co., 7/29/? (2, TAMU); TX, Dimmit Co., Chaparral WMA, Pasture 10, 10/10/2000, Raber & Riley, (4, TAMU); TX, Edwards Co., 24 mi. S Junction, Hwy. 377, 4/10/2002, 30°15'15"N, 99°57'48"W, Riley & Yoder, (1, TAMU); TX, Medina Co.,. 75 mi. S of D’Hanis, 9/17/1993, E.G.Riley, (8, TAMU); TX, Mason, 11/22/1969, B.L.Hofmann, (2, TAMU); TX, Chisos Mts., 6/30/1957, D.J. & J. N.Knull, (1, OSU); TX, Val Verde Co., Seminole Canyon SHA, 8/30/1986, East, Kovarik & Haack, (1, TAMU); TX, Val Verde Co., Seminole Canyon SHP, 6/3/1983, C.B.Barr, (1, EMEC); TX, Val Verde Co., Lake Walk near Del Rio, 5/27/1967, E.E.Remington, (1, PMNH); TX, Kerr Co., Kerrville, 5/28/1963, W.F.Chamberlain, (1, TAMU); TX, Nueces Co., Corpus Christi, Hardee’s on S Padre Island Dr., 6/29/1986, Weisman & Lightfoot, (1, CAS); TX, Hidalgo Co., Weslaco, 6/25/1979, G.W.Brooks, (1, TAMU); TX, Sinton, 8/31/1964, M.H.Sweet, (1, TAMU); TX, Hidalgo Co., Weslaco, 11/1/1940, P.T.Rihard, (2, TAMU); TX, San Patricio Co., Cd. Welder WA, 7/16/1989, J. Schaffner, (1, TAMU); TX, San Patricio Co., Corpus Christi SP, 7/12/1963, G.W.Byers & party, (1, SEMC); TX, San Patricio Co., Corpus Christi SP, 8/25/1962, H.R.Burke, (1, TAMU); TX, Brooks Co., 6 mi. S of Falfurrias, 10/10/1970, (1, TAMU); TX, Brooks Co., 7.3 mi. S of Falfurrias on hwy. 281, 4/27/1991, E.G.Riley, (1, TAMU); TX, Brooks Co., 7.3 mi. S of Falfurrias on hwy. 281 rest stop, 5/8/1989, E.G.Riley, (2, TAMU); TX, Milam Co., Sugarloaf Mt. 4 mi. N of Gause, 10/4-23/1992, 300’, Abbott,Godwin,Migura & Riley, (3, TAMU); TX, Milam Co., Sugarloaf Mt., 7/22/1992, 500’, Riley & Godwin, (1, TAMU); TX, Milam Co., 4 mi. N of Gause near Sugarloaf Mt., 4/18/1993, E.Riley, (1, TAMU); TX, Milam Co., Sugarloaf Mt., 5/30/1998, R.Turnbow, (1, FSCA); TX, Caldwell Co., 4.5 mi. E of McMahon, 6/2/1998, R.Turnbow, (1, FSCA); TX, Kenedy Co., 2.7 mi. S of Sarita, 4/27/1991, E.G.Riley, (1, TAMU); TX, Kenedy Co., Kenedy Ranch, Jaboncillos Pasture, sand dunes, 4/6-20/2001, 26°58'38"N, 97°40'59"W, Godwin & Riley, (2, TAMU); TX, Kenedy Co., Kenedy Ranch, Jaboncillos Pasture, sand dunes, 4/21/2001, 26°59'22"N, 97°40'11"W, Raber,Riley & Yoder, (1, TAMU); TX, Padre Island, 7/1/1965, Dr Lenczy, (1, LACM); TX, Bexar Co., Ebony Hill Res Station, 9/4/1984, Kendall & Kendall, (1, TAMU); TX, Bexar Co., Lab. Garden, 9/11/1970, R.O.Kendall, (1, TAMU); TX, Bexar Co., Ebony Hill Res Station, 5/11/1991, Kendall & Kendall, (1, TAMU); TX, Bexar Co., Ebony Hill Res Station, 11/1/1980, Kendall & Kendall, (1, TAMU); TX, Burnet Co., Inks Lake SP, 6/13/1972, J.S.Ashe, (1, TAMU); TX, Duval Co., 8.5 mi. (?) San Diego, 9/18/1993, E.G.Riley, (1, TAMU); TX, Kleberg Co., vicinity of Kingsville, C.Reed, (3, ANSP); TX, Austin, 8/15/1968, (1, UCRC); TX, McLennan Co., Waco, Texarcana, 5/11/1938, (1, ANSP); TX, Three Rivers, 6/27/1938, R.H.Beamer, (1, ANSP); TX, Shovel Mount, 9/5/1901(?), F.G.Schaupp, (1, ANSP); TX, San Antonio, 7/4/1953, E.S.Ross, (1, CAS); TX, Brownsville, June, (1, ANSP); TX, San Antonio, 7/7/1942, E.S.Ross, (1, CAS); TX, Waco, 7/1910(?), (1, MCZ); TX, San Antonio, Oct. 1942, E.S.Ross, (1, CAS); TX, Nueces Co., Clare (Hazel?) Bazemore Park, 4/10/1970, C.W.Griffin, (1, USNM); TX, Corpus Christi SP, 10/6/1951, A.B.Gurney, (2, USNM); TX, San Antonio, 4/1/1935, E.V.Walter, (1, USNM); TX, Zavalla Co., Nueces Riv., 6/2?/????, F.C.Pratt, (1, USNM); TX, Belfrage, (3, USNM); TX, Kerrville, 9/21/1951, A.B.Gurney, (2, USNM); TX, ?/?/1927, Clyde T. Reed, (1, USNM); TX, Bexar Co., Randolph Field, 10/4/1943, Pierce Brodkorb, (1, UMMZ); TX, Corpus Christi nr. Casa Blanca Lake, 12/17/1939, L.Berner, (1, UMMZ); TX, Ft. Sam Houston, 9/20/1950, J.E.Gentry, (1, UMMZ); TX, Burnet Co., Longhorn Cavern 11 mi. SW Burnet, 7/5/1959, 1200’, T.J.Cohn, (2, UMMZ); TX, Belfrage, H.S.Wallace, (1, UMMZ); TX, Dallas, C.V.Riley, (1, USNM); TX, Hidalgo Co., Edinburg, 4/?/1939, Stanley Mulaik, (3, UMMZ); TX, Cameron Co., Brownsville, 7/31-8/5/1912, (1, ANSP); TX, Austin, 10/?/1900, (1, UMMZ); TX, Kleberg Co., 3.5 mi. N Riviera, 6/29/1961, L.Westcott, (1, LACM); TX, Travis Co., 5 mi. NE Austin PO (WFBlair’s), 7/15/1955, 600-700’, T.J.Cohn, (6, USNM); TX, Goliad Co., 1 mi. S Goliad, 7/22/1955, 100’, T.J.Cohn, (4, USNM); TX, Belfrage, C.V.Riley, (1, USNM); TX, Belfrage [another illegible word], 9/?/1921, S.H.Scudder, (1, USNM); TX, Kerrville, ?/22/1908, F.C.Pratt, (1, USNM); TX, illegible label, S.H.Scudder, (1, USNM); TX, Brownsville, 12/2/1951, (1, USNM); TX, (1, USNM); TX, Austin, 6/4/1952, R.A.Stirton, (1, USNM); TX, Caldwell Lockhart SP, 4 mi. SW of Lockhart, 7/9/1955, 500-600’, Cohn & Matthews, (2, USNM); TX, Shovel Mount, 10/18/1901, F.G.Schaupp, (1, USNM); TX, Travis Co., ?/?/1931, J.K.G.Silvey, (1, UMMZ); TX, Dimmit Co., Catarina, 7/7/1948, Nutting & Werner, (1, UAIC); TX, Gonzales Co., Luling, 6/19/1953, M.Wasbauer, (3, EMEC); TX, Kenedy Co., Armstrong, 3/31/1962, H.Glick, (1, CSCA); TX, Kenedy Co., Armstrong, 6/13/1962, P.A.Glick, (2, CSCA); TX, Ringgold Barracks Schott, S.H.Scudder, (1, USNM); TX, Carrizo Springs, 8/28/1985, Dr. A Wadgynear, (1, USNM); TX, Sonora, 8/28/1924, O.G.Babcock, (1, USNM); TX, San Antonio, Fall 1947, H.C.Barnett, (1, USNM); TX, San Antonio, 8/8/1933, E.V.Walter, (1, USNM); TX, Mercedes, May 1034, Thayer, (1, USNM); TX, Cameron Co., Sabal Palm Grove Sanct., 10/20/1990, Carlow & Riley, (3, TAMU); TX, Cameron Co., Sabal Palm Grove Sanct., 7/26/1991, Riley & Carlow, (2, TAMU); TX, Cameron Co., Sabal Palm Grove WR, 10/18/2002, Raber & Riley, (1, TAMU); TX, LaFeria, 9/26/1963, P.T.Riherd, (1, TAMU); TX, Cameron Co., Sabal Palm Grove Sanct., 10/13-14/1988, E.G.Riley, (1, TAMU); TX, Cameron Co., LRGVNWR Voshell Unit, Brownsville, 6/5-6/2009, 25.88873°N, 97.43142°W, Heffern & Riley-1030, (4, TAMU); TX, LaFeria, 10/21/1959, P.T.Riherd, (1, TAMU); TX, Val Verde Co., 5/24/1948, Knull & Knull, (1, FSCA); TX, Val Verde Co., Comstock, 8/11/1975,Taylor & Sullivan, (4, LACM); TX, Langtry, Sept. 1979, H.Hartman, (1, MCZ); TX, Cameron Co., Brownsville, June, F.H.Snow, (1, ANSP); TX, Cameron Co., Sabal Palm Grove Ref. site 3, 9/18-10/2/2008, 25.84964°N, 97.41849°W, Lindgren FT, King & Riley-193, (2, TAMU); TX, Cameron Co., Sabal Palm Grove Ref. site 1, 9/3-18/2008, 25.84799°N, 97.41881°W, King & Riley, (1, TAMU); TX, Cameron Co., Brownsville, 6/21/1969, Board & Hafernik, (1, TAMU); TX, Cameron Co., Sabal Palm Grove Audubon Sanct., 5/5/1989, E.G.Riley, (1, TAMU); TX, Cameron Co., Brownsville, 12/9/1911, (1, ANSP); TX, Cameron Co., Sabal Palm Grove Sanct., 4/8/1994, E.G.Riley, (2, TAMU); TX, Val Verde Co., Seminole Canyon SHA, 8/30/1986, East, Kovarik &Haack, (3, TAMU); TX, Cameron Co., Esprza Rch Brownsville, 7/1/1930, (3, USNM); TX, Cameron Co., Brownsville, H.S.Barber, (2, USNM); TX, Cameron Co., Esprza Rch Brownsville, August, (2, USNM); TX, Uvalde Co., Speir Rch. 3 mi. NW Uvalde, 5/6/1977, Eichlin & Wasbauer, (1, CSCA); TX, Uvalde Co., Uvalde, 6/18/1920, Wickham, (1, USNM); TX, Maverick Co., Eagle Pass Horn., S.H. Scudder, (2, USNM); TX, Cameron Co., Brownsville, April, (1, USNM); TX, Cameron Co., 13.4 mi. E of Brownsville, 7/17/1962, (2, SEMC); TX, Hidalgo Co., Mission, 7/1/1961, R.L.Westcott, (1, LACM); TX, Cameron Co., Brownsville, 6 mi. N of PO, 8/20/1955, Bermler & Cohn, (4, UMMZ); TX, Hidalgo Co., Santa Ana WR, 7 mi. S of Alamo, 5/6/1967, A & M.E. Blauchard, (1, LACM); TX, Cameron Co., Esprza Rch Brownsville, 6/1/1915, (1, USNM); TX, Cameron Co., Brownsville, 5/1/1929, (5, USNM); TX, Cameron Co., Brownsville, Los Borregos, 6/5/1904, H.S.Barber, (2, USNM); TX, Cameron Co., Brownsville, 6/9/1962, P.A.Glick, (1, CSCA); TX, Cameron Co., Southmost, 6/13/1952, (1, SEMC); TX, Cameron Co., 8/3/1928, R.H.Beamer, (2, SEMC); TX, Cameron Co., Brownsville, 6/8/1920, R.D.Camp, (1, UMMZ); TX, San Antonio, at light on kitchen door at night, 6/13/1948, H.C.Barnett, (1, USNM); TX, Del Rio KOA, nite lite, 10/6/1974, (1, SDMC); TX, Las Paloma, 9/8/1968, Kirby & Phipps, (1, USNM); TX, Esprza Rch Brownsville, 8/1/1929, (1, USNM); FL, Gainesville, 9/10/1969, D.Bennett, (1, USNM); FL, Seminole, 10/?/1974, BJ Wyckoff, (1, MLBM); TX, Bexar Co., Leon Valley, 7/4/1968, GH & JM Nelson, (1, FSCA); TX, Lake Corpus Christi SP, 6/18/1971, GH Nelson, (2, FSCA); TX, Brewster Co., Big Bend NP near Pulliam Mtn., 8/16/1970, 6000 ft., RE Woodruff, (1, FSCA); TX, Travis Co., Austin, Breckenridge Field Lab, 10/6/1989, 550 ft., CR Nelson #5412, (1, MLBM); TX, Val Verde Co., Devils R, Dolan Falls, 8/5-7/1994, AM Hook & O Hernandez, (1, MLBM); TX, Uvalde Co., Garner SP, 6/17/1968, GH Nelson & family, (2, FSCA); TX, Dimmit Co., Chaparral WMA, 6/7-8/1992, AW Hook, (4, MLBM); TX, Starr Co., Falcon Heights, 10/9/1993, SM Clark, (1, MLBM); TX, Travis Co., BFL, 10/?/1997, SM Brandt, (1, MLBM); TX, Dimmit Co., Chaparral WMA, 8 mi W of Artesia Wells, 5/19/1999, 28.18.41N 99.24.25W, CR Nelson #6940, (1, MLBM); TX, Cameron Co., Sabal Palm Grove Sanctuary, 9/25/1996, SM Clark,(1, MLBM). MEXICO: Tamaulipas, Abasolo, 5/17/1952, Cazier,Gertsch & Schrammel, (2, AMNH); Tamaulipas, San Fernando, 8/26-27/1954, 700 ft., CD Michener & party, (5, SEMC); Tamaulipas, 8 mi. SW of Ciudad Victoria, 10/5/1958, 1500-2000 ft., TJ Cohn, (1, UMMZ); Tamaulipas, Hacienda Clementine 13 mi. ESE of Llera, 8/25/1955, 200 m, TJ Cohn, (1, UMMZ); Nuevo Leon, 16.5 mi. W of Linares, 7/22-24/1977, Peigler & Plitt, (1, TAMU); Nuevo Leon, 15 mi. W of Linares, 7/1-2/1973, Mastro & Schaffner, (1, TAMU); Tamaulipas, 12 mi. S of Nuevo Laredo, 7/9/1936, 400 ft., HR Roberts, (1, ANSP); Tamaulipas, Canon la Libertad, 4/4/1986, RW Jones, (1, TAMU); Nuevo Leon, Garza Garcia, 4/18/1955, L Ayola Jr., (1, UMMZ); Durango, near Pedricena, 8/27/1932, H Smith, (1, ANSP); Monterey, 12/12/1991, WF Chamberlain, (1, TAMU); Nuevo Leon, 17 km N of Sabinas Hidalgo, 5/24/1948, Nutting & Werner, (1, UAIC); Tamaulipas, 8 mi. E of Padilla Rancho Sta. Ana, 12/21/1941, Cantrell & Friauf, (2, UMMZ). Determiner label *Arenivaga bolliana* Hopkins 2011” [white label with black border].

##### Distribution.

*Arenivaga bolliana* is found across southern and eastern Texas and far northeastern Mexico. There are two isolated records in central and western Mexico, and two more in Florida. It is impossible to say if these are established populations or incidents of specimens transported by man or weather. It is hard to imagine four such incidences occurring and then the transported specimens being collected but it is remotely possible. See Fig. 35.

##### Diagnosis.

*Arenivaga bolliana* may be easily confused with *Arenivaga grata*. Both are large and dark in color, though the territory of *Arenivaga grata* is distinctly to the west of that of *Arenivaga bolliana*. *Arenivaga bolliana* may be diagnosed by the large angular right dorsal phallomere with very simple medial margin and no complexity in the point of articulation of the two right phallomeres. See Figs 35 and 73.

##### Description.

**Male.** **NB: Holotype is half spread therefore GW is estimated.** *Measurements*. Holotype TL = 24.6 mm, GW = 13.0 mm, PW = 8.64 mm, PL = 5.60 mm, TL/GW = 1.89, PL/PW = 0.65. EW = 0.40 mm; OW = 0.60 mm. Among paratypes range of TL 20.1–30.7 mm; range of GW 9.6–15.3 mm; range of PW 7.25–10.10 mm; range of PL 4.74–6.17 mm.

**Figure 33.**
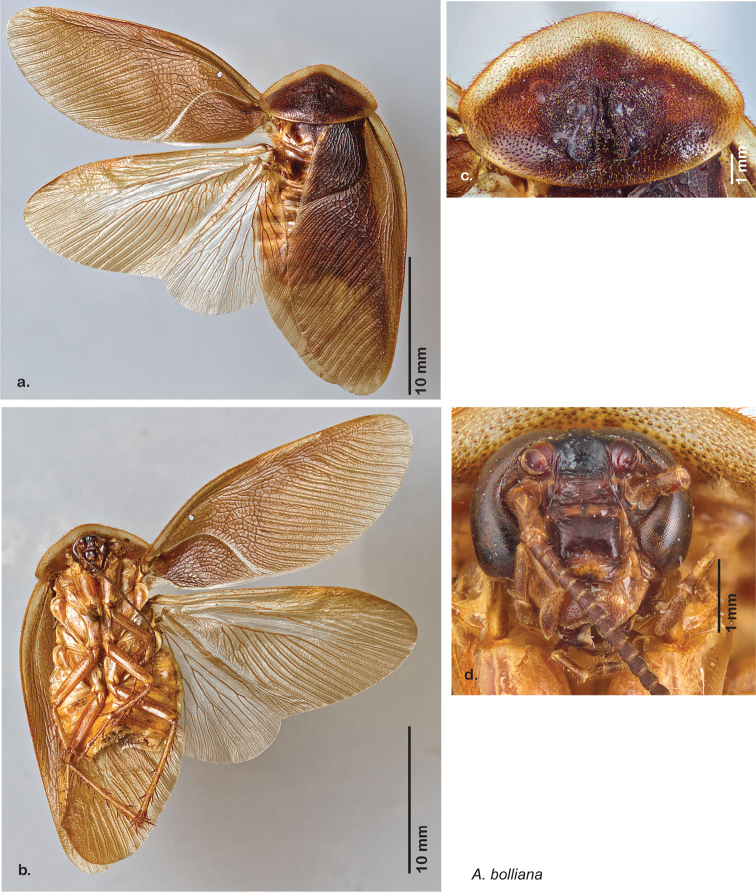
*Arenivaga bolliana*, **a** dorsal habitus **b** ventral habitus **c** pronotum **d** head.

*Head*. Two ocelli large, ovoid and protruding (0.50 × 0.40 mm); vertex dark brown, with small ridges between apices of eyes; interocellar space concave, dark brown. Frons medium brown, tectiform, concave with fine horizontal corrugations; bound on either side by ridges extending from inner apex of ocelli outwards to lateral edges of clypeus. Anterior portion of frons medium brown, bulbous; clypeal suture demarcates medium brown anteclypeus. See Fig. 33d.

*Pronotum*. Pronotum with broad anterior margin of translucent waxy beige, extending and narrowing laterally; variable length orange-brown setae along anterior margin; dorsal surface of pronotum covered with short orange-brown setae; pronotal pattern impressed ranging from medium brown to very dark brown, all with extensive aura; no discernible detail. See Fig. 33c.

*Body*. Wing brace absent. Legs and body medium orange-brown; subgenital plate asymmetrical with posterior edge emarginated, rounded apices. See Fig. 33b.

*Forewings*. Wings extended beyond abdominal apex (up to ~30% of the total wing length); color ranges from light brown with virtually no blotches to every level of blotchiness to uniform dark brown depending on specimen; surface opaque and matte. See Fig. 33a.

**Figure 34.**
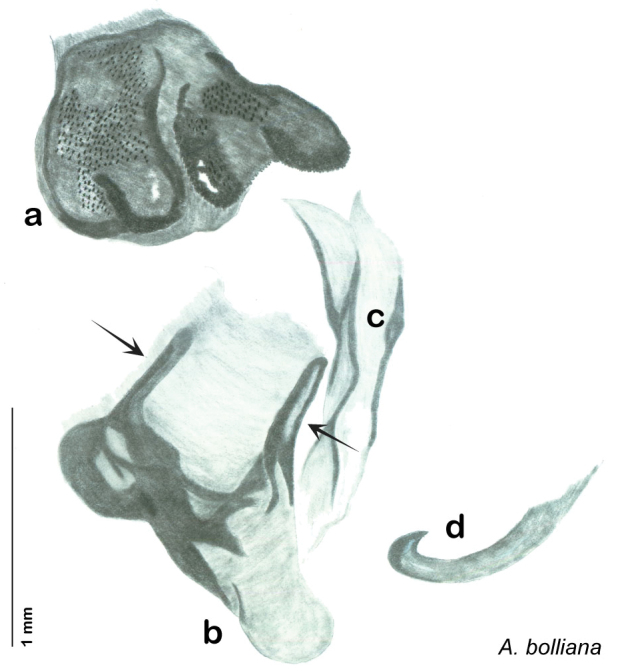
*Arenivaga bolliana*, genitalia: **a** right dorsal phallomere **b** right ventral phallomere **c** small central sclerite **d** genital hook. Arrow(s) indicate diagnostic characters (see text).

*Genitalia*. Right dorsal phallomere composed of bulbous lightly sclerotized wavy dorsally projecting lobe, articulated with right ventral phallomere on lateral side; central field broad, slightly sclerotized; medial margin sclerotized, with toothed edge and slight central indentation. Small central sclerite finely punctate, folded lengthwise and attached dorsally. Right ventral phallomere extends from articulation into shagreened lobe with broad indentation at posterior end and medial concavity; after narrow gap, wide concave shagreened flange. Folded anterior portion of left phallomere of moderate width, setose, otherwise unmodified. Genital hook with rounded head with moderate hook; arm smoothly curved. See Fig. 34.

**Figure 35.**
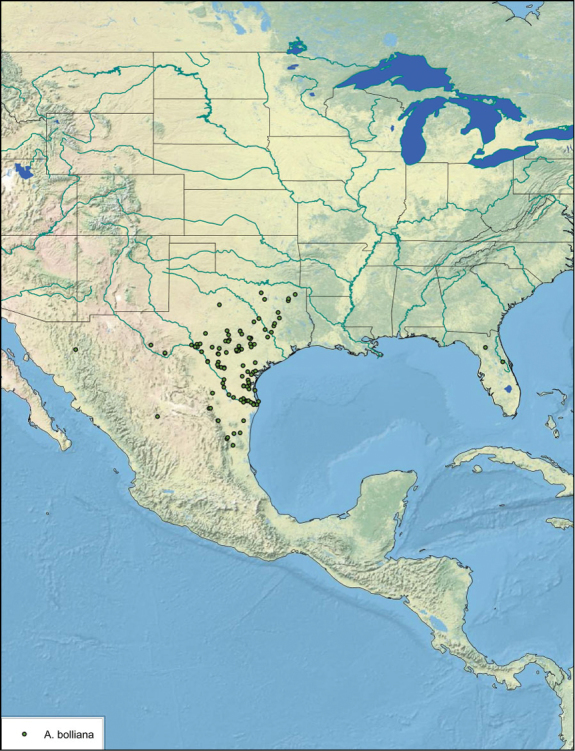
*Arenivaga bolliana*, distribution.

##### Habitat and natural history.

All life history elements remain unobserved.

#### Arenivaga darwini sp. n.

http://zoobank.org/CC9C923E-7E0F-4509-A6DF-8314E6E8BB81

http://species-id.net/wiki/Arenivaga_darwini

Figures 36 – 38

##### Type locality.

USA, California, Imperial County, 2 mi. NW of Glamis.

##### Material examined.

Holotype: ♂ in Essig labeled “CAL: Imperial Co., 2 mi. NW Glamis, III-29-77, Powell, on sand dunes” “HOLOTYPE *Arenivaga darwini* Hopkins, 2012” [red label with black border].

Paratypes (132): USA: CA, Imperial Co., Algodones Dunes, I8 at Ogilby Road, 3/7/1988, RE Woodruff, dunes at night (4, FSCA); CA, Imperial Co., 13 mi. SE of Glamis, 2/10/1972, AR Hardy, on sand dunes (1, UCRC); CA, Imperial Co., 2 mi. NW of Glamis, 3/29/1977, Powell, on sand dunes (20, EMEC); CA, Imperial Co., 3 mi. NW of Glamis, 4/3/1972, AR Hardy, sand dunes (1, UCRC); CA, Imperial Co., 1 mi. W of Glamis, ?/28/1965, ME Irwin, dunes (1, UCRC); CA, Imperial Co., 12 mi. W of AZ border, I10 dunes S of Ogilby exit, 2/27/1988, P Parrella, collected on sand dunes, Polyphagidae det. By P.Parrella 1988 (1, ASUT); CA, Imperial Co., 12 mi. W of AZ border, I10 dunes S of Ogilby exit, 2/27/1988, I Gallicano, collected on sand within 2 mi. of main road, Polyphagidae det. By Ian Gallicano 1988 (3, ASUT); CA, Imperial Co., 12 mi. W of AZ border, I10 dunes S of Ogilby exit, 2/27/1988, M Harding, Lying still on sand dunes, Polyphagidae det. By M Harding 1988 (1, ASUT); CA, Imperial Co., I10 0.5 mi. S of Ogilby Rd. exit, 2/27/1988, C Bagnoll, Lying still on sand dunes, Polyphagidae det. By C. Bagnoll 1988 (1, ASUT); CA, Imperial Co., 12 mi. W of AZ border, I10 dunes S of Ogilby exit, 2/27/1988, M Mustain, dunes at night, Polyphagidae det. By M.Mustain 1988 (1, ASUT); CA, Imperial Co., 12 mi. W of AZ border, I10 dunes S of Ogilby exit, 2/27/1988, R Shill, dunes at night, Polyphagidae det. By R Shill 1988 (1, ASUT); CA, Imperial Co., 12 mi. W of AZ border, I10 dunes S of Ogilby exit, 2/27/1988, L Davison, dunes at night (1, ASUT); CA, Imperial Co., 3.5 mi. NW of Glamis, 3/10/1973, Andrews & Hardy, on sand dunes (8, CSCA); CA, Imperial Co., Algodones Dunes, 7 mi. SE of Glamis, 3/25/-4/8/1979, 32.55.20N 114.59.14W, Site 4, dunes at night (2, CSCA); CA, Sand dunes E of Grays Well, 4/30/1952, ER Tinkham (2, USNM); CA, Imperial Co., Glamis sand dunes 5 mi. W of Ogilby, 5/29/1981, Werner,Olson,Hetz,Thomas,Burne,Frank,MacLachlan (1, UAIC); CA, Imperial Co., 3 mi. NW of Glamis, 4/4/1972, EA Kane, fluorescent black light (1, CSCA); CA, Imperial Co., 5 mi. W of Ogilby, 5/9/1959, V Roth (1, USNM); CA, Imperial Co., Imperial Sand Dunes RA, Wash Rd. ~7.2 mi. S of Hwy 78, 3/28/2002, 32.55.31N 114.58.52W, CB Barron, dunes at night (1, EMEC); AZ, Maricopa Co., Tempe, ASU campus, stairwell of life science bldg., 7/12/1988, Polyphagidae det. By E Rocklin 1988 (1, ASUT); AZ, Yuma Co., Yuma, 4/3/1959, L Anharal (1, UAIC); AZ, Yuma Co., lg.sand dunes SE of Yuma, 4/16/1994, 32.27N, 114.25W, WB Warner (1, WB Warner); AZ, Yuma Co., Mittry Lake boat launch, 2.5 mi. ??? of Laguna YPG, 11/27/1988, M Childs, Polyphagidae det. By M Childs 1988 (1, ASUT); AZ, Maricopa Co., 3 mi. SE of I10 on Ray Rd., 4/6/1988, KK Menze, empty fields dry soil, Polyphagidae det. By KK Menze 1988 (1, ASUT); AZ, dunes, 22 mi. E of San Luis, 6/1/1958 150 ft., ER Tinkham (20, USNM). MEXICO: Sonora, 27 mi. E of San Luis, 6/24/1957, ER Tinkham, dunes (2, USNM); Sonora, 10 mi. N of C. Sotelo near Bahia Adair, 3/13/1973,Andrews & Hardy,on sand dunes (10, CSCA); Sonora, 50 mi. SW of Sonoyta,3/12/1973, Andrews & Hardy, on sand dunes (41, CSCA); BC, 4.9 mi. SW of Algodones Dunes, 3/25/1986, 32.48.734N 114.48.234W, RH McPeak, blacklight (1, EMEC). All paratypes labeled “Paratype *Arenivaga darwini* Hopkins 2012” [blue label with black border].

##### Etymology.

The name is a noun in the genitive case. This species is named for Charles Darwin.

##### Distribution.

This species is distributed in and around Yuma, AZ and vicinity and down the eastern coast of the Sea of Cortez. See Figure 38.

##### Diagnosis.

*Arenivaga darwini* sp. n. has one tarsal claw on each leg, unique to the genus.

##### Description.

**Male.** *Measurements*. Holotype TL = 16.2 mm, GW = 9.8 mm, PW = 7.25 mm, PL = 4.45 mm, TL/GW = 1.65, PL/PW = 0.61. EW = 0.6 mm; OW = 0.5 mm. Among paratypes range in TL 13.1–17.3 mm; range in GW 8.6–10.9 mm; range in PW 5.55–7.67 mm; range in PL 3.5–4.45 mm.

**Figure 36.**
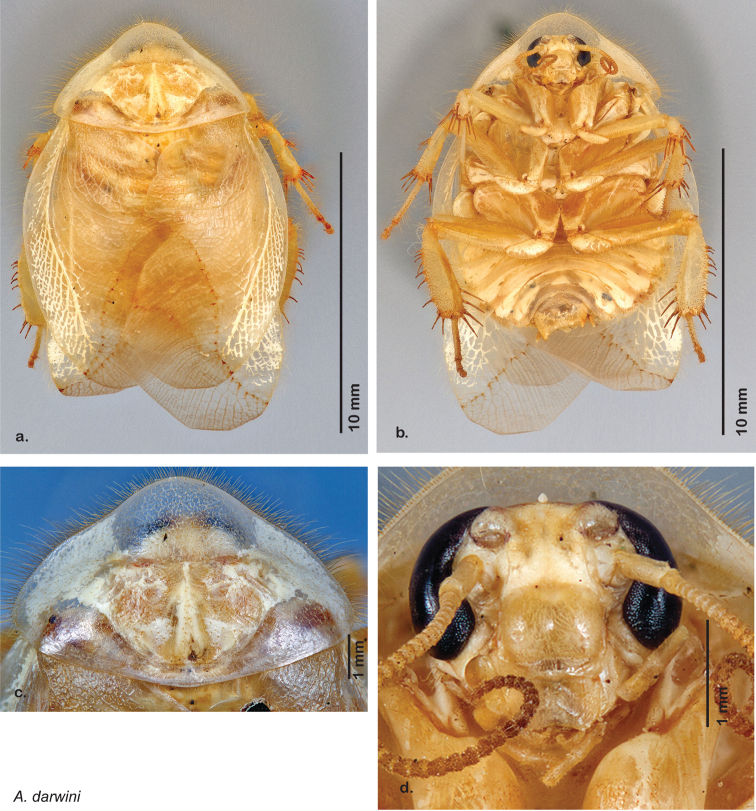
*Arenivaga darwini*, **a** dorsal habitus **b** ventral habitus **c** pronotum **d** head.

*Head*. Two ocelli large and ovoid (0.40 × 0.30 mm); vertex golden; interocellar space concave, golden fading to white towards frons. Frons concave; clypeus bulbous, golden with no notable sculpturing or setae, ends in broad flat anteclypeus. See Fig. 36d.

*Pronotum*. Pronotum unusually broad relative to length, pale, waxy beige-gold; anterior half of dorsal surface of pronotum covered in fine pale setae; posterior half sparsely setose; pronotal pattern ranges in color from same waxy beige-gold of background, to white and orange-brown depending on specimen; pattern is foreshortened “koala face”, indiscernible detail; no aura. See Fig. 36c.

*Body*. Wing brace present. One tarsal claw present. Legs and body golden with white deposits of uric acid particularly visible on outer margins of forewings. Abdominal sternites end in conspicuous points on lateral edges. Subgenital plate dramatically asymmetrical with pointed apices. See Fig. 36b.

*Forewings*. Wings extend beyond abdominal apex (~25% of wing length); forewings shorter than hindwings; color pale iridescent gold and translucent to transparent. Long golden setae on lateral edges. See Fig. 36a.

**Figure 37.**
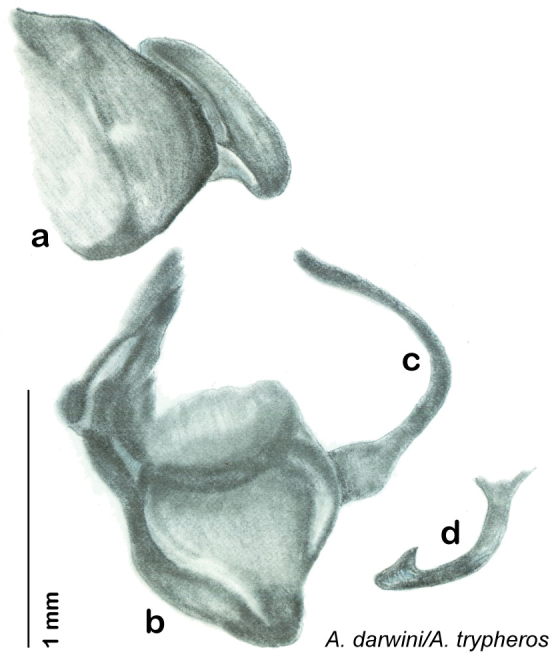
*Arenivaga darwini*, genitalia: **a** right dorsal phallomere **b** right ventral phallomere **c** small central sclerite **d** genital hook. Arrow(s) indicate diagnostic characters (see text).

*Genitalia*. Right dorsal phallomere composed of large bulbous lightly sclerotized pointed lobe, articulated with right ventral phallomere on lateral side; unmodified, covered in fine punctations. Small central sclerite consists solely of thin half circle of sclerotized material beginning midlaterally on right dorsal phallomere and sweeping around to rear of same phallomere; interior tissue of ring finely punctate. Right ventral phallomere extends from articulation to form structure rounded at posterior apex and expanding to shagreened and more sclerotized area dorsally; attached anteriorly is an L-shaped shagreened lobe bordered by rolled shagreened lip. Left phallomere unmodified. Genital hook with long extension to pointed head with slight indentation on short hook; arm with distinct bend. See Fig. 37.

**Figure 38.**
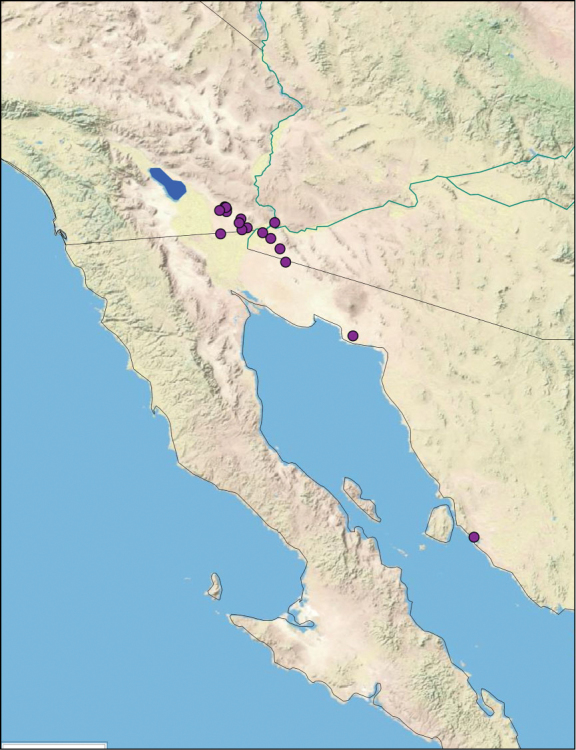
*Arenivaga darwini*, distribution.

##### Habitat and natural history.

This species occurs in sparsely vegetated sand dunes that are extremely dry and hot. More than one specimen carried mites. All other life history elements remain unobserved.

#### Arenivaga delicata sp. n.

http://zoobank.org/5AB46CDA-BE7E-435E-A292-CB50C62A7B05

http://species-id.net/wiki/Arenivaga_delicata

Figures 39 – 41

##### Type locality.

USA, California, San Bernardino Co., Old Woman Mts.

##### Material examined.

Holotype: ♂ in LACM labeled “CALIF. S. Bdno. Co: nr Sunflower Wash, 3300 ft., Old Woman Mts., T5N R18E SW ¼ sec 5, 28-29 May ’88, JP and KES Donahue, 121578” “HOLOTYPE *Arenivaga delicata* Hopkins, 2012” [red label with black border].

Paratypes (23): USA: CA, Riverside Co., N of Blythe, 1/29/1966, D Park, under rock on ground, Cal.Dept.Agr.6687-25 (1, CSCA); CA, Kern Co., 1 mi S of Willow Spring, 10/4/1960, WE Ferguson, at light (1, EMEC); CA, Riverside Co., 4 mi N of Blythe, 7/22/1975, Sumlin,Garcia & Drake, alluvial fan, UV light (1, UCRC); CA, Cottonwood Springs, 11/7/1950, ER Tinkham, red tag (1, USNM); CA, San Bernardino Co., Halloran Springs, 14 mi E of Baker, 4/15/1964, RL Langston (1, EMEC); CA, San Diego Co., San Diego, GR Crotch (1, ANSP); CA, Riverside Co., 4 mi NW of Desert Center, 6/20/1956, M Wasbauer (1, EMEC); CA, Providence Mts., 4/12/1934, ML Walton, Collection of ML Walton donated to LACM 1975 (1, LACM); CA, San Bernardino Co., Needles, 5/31/1968, JC Lambert, light trap (1, CSCA); CA, San Bernardino Co., Needles, 5/10/1983, D Pendleton (1, SDMC); CA, Mono Co., White Mts., Coldwater Canyon, 4/21-11/15/1983, D Giuliani, antifreeze pit trap (1, CSCA); CA, Kern Co., Iron Canyon E of Garlock, 4/1/1961, CA Toschi (1, EMEC). All paratypes labeled “Paratype *Arenivaga delicata* Hopkins 2012” [blue label with black border].

##### Etymology.

The name is an adjective in the nominative singular. This species is named *delicata* from Latin meaning delicate. Though not as short in total length as *Arenivaga pumila* or *Arenivaga ricei* it is one of the smallest and most delicate of the *Arenivaga*.

##### Distribution.

This species is distributed from Willow Spring, CA in the north and west to Blythe, CA in the south and east. See Fig. 41.

##### Diagnosis.

*Arenivaga delicata* is smaller than average for *Arenivaga* but may be confused with *Arenivaga mortisvallisensis* which has a sympatric distribution. *Arenivaga delicata* may be distinguished by the clamshell shape of the small central sclerite and the distinctive crossing band of teeth towards the anterior end. See Figs 40 and 109.

##### Description.

**Male.** *Measurements*. Holotype TL = 17.7 mm, GW = 8.0 mm, PW = 4.76 mm, PL = 3.23 mm, TL/GW = 2.21, PL/PW = 0.68. EW = 0.40 mm; OW = 0.30 mm. Among paratypes range of TL 14.7–17.7 mm; range of GW 6.9–8.4 mm; range of PW 4.28–4.76 mm; range of PL 3.07–3.42 mm.

**Figure 39.**
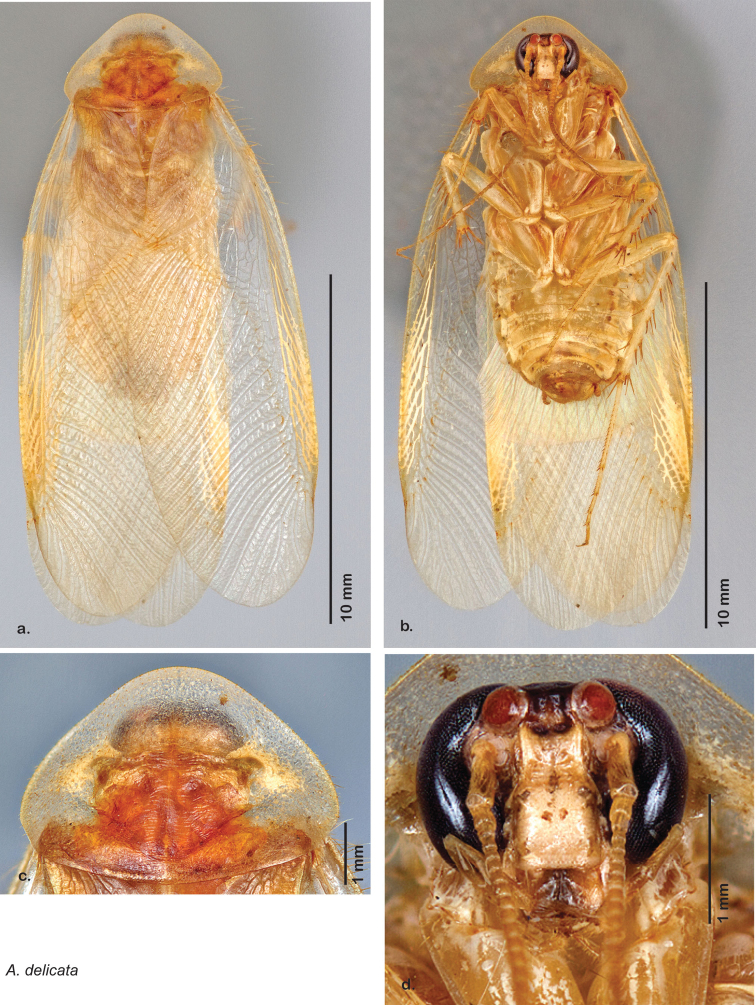
*Arenivaga delicata*, **a** dorsal habitus **b** ventral habitus **c** pronotum **d** head.

*Head*. Two ocelli very large, ovoid and protruding (0.4 × 0.3 mm); vertex dark brown with small ridges in rays around upper apex of eyes and extending onto ocellar tubercles; interocellar space concave, smooth, medium brown; two round indentations medial to ocelli. Frons pale beige, concave; clypeus bulbous; pale beige anteclypeus. See Fig. 39d.

*Pronotum*. Pronotum translucent, waxy beige; dorsal surface of pronotum with short fine light orange-brown setae centrally and posteriorly grading to longer, thicker setae laterally and anteriorly; pronotal pattern orange-brown “panther face” with no discernible detail; orange-brown posterior aura. See Fig. 39c.

*Body*. Wing brace present. Two tarsal claws present. Legs and body light brown with darker brown maculations laterally on each sternite; subgenital plate light brown; asymmetrical with rounded apices. See Fig. 39b.

*Forewings*. Wings extended well beyond abdominal apex (~45% of wing length); color golden with light to medium brown splotches depending on specimen; surface translucent and lustrous. See Fig. 39a.

**Figure 40.**
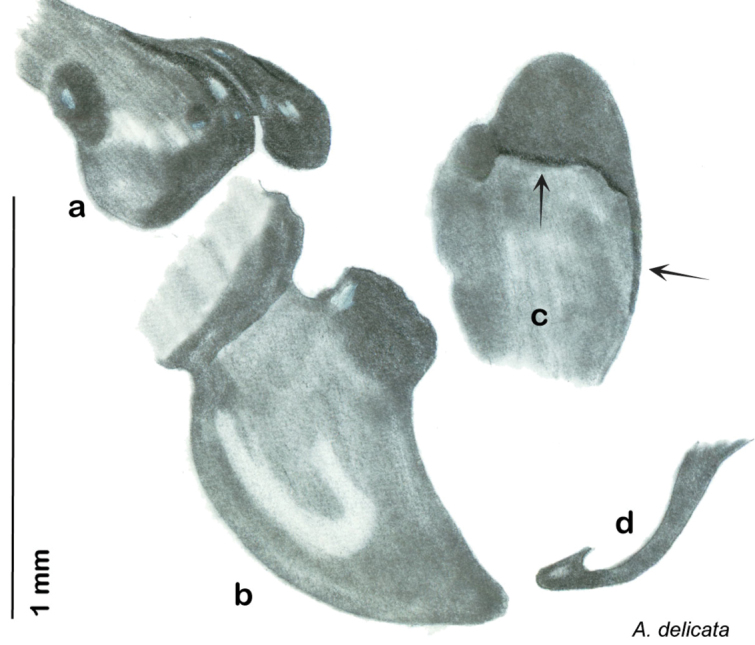
*Arenivaga delicata*, genitalia: **a** right dorsal phallomere **b** right ventral phallomere **c** small central sclerite **d** genital hook. Arrow(s) indicate diagnostic characters (see text).

*Genitalia*. Right dorsal phallomere composed of bulbous lightly sclerotized hook-shaped lobe, articulated with right ventral phallomere on lateral side; central field lightly sclerotized; medial margin short, shagreened with toothed margin. Small central sclerite shaped like clamshell, finely punctate with crossing band of teeth one third of distance from anterior end; right ventral phallomere extends from articulation to form rounded punctate structure; attached anteriorly is mildly dorsally projecting flanged arm, shagreened with roughly toothed edge. Folded anterior portion of left phallomere setose, otherwise unmodified. Genital hook with short extension to pointed rounded head and short hook; arm smoothly curving. See Fig. 40.

**Figure 41.**
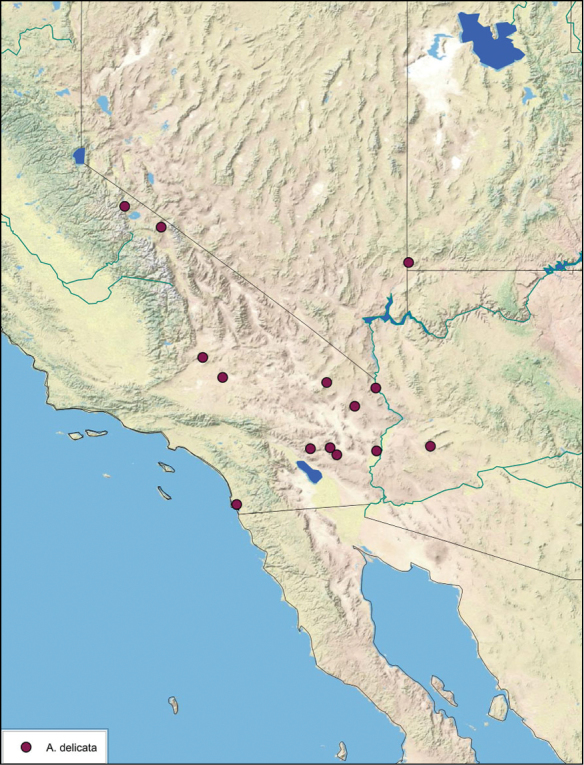
*Arenivaga delicata*, distribution.

##### Habitat and natural history.

All life history elements remain unobserved.

#### Arenivaga diaphana sp. n.

http://zoobank.org/5AB7F19E-30DE-4DC8-93F0-E86D3C48CAF6

http://species-id.net/wiki/Arenivaga_diaphana

Figures 42 – 44

##### Type locality.

MEXICO, BCN, 10.1 mi W Catavina.

##### Material examined.

Holotype: ♂ in SDMC labeled “MEX: Baja California Norte, 10.1 mi W. Catavina, 24-28 Feb 1988, N. Bloomfield, [green dot]” “HOLOTYPE *Arenivaga diaphana* Hopkins, 2012” [red label with black border].

Paratypes (123): MEXICO: BCN, 6 mi N of Guerrero Negro 3/16/1981, Andrews & Faulkner, Collected on sand dunes at night (1, CSCA); BCN, 6 mi NW of Rancho Ynez, 1/?/1976, 29.43N, 114.43W, 1800 ft, D Ward (1, IMNH); BCN, 36 mi N of San Felipe, 4/18-21/1961, FS Truxal (1, LACM); BCN, Catavina Canyon, 6/1/1981, Werner,Olson,Hetz,Thomas,Burne,Frank, MacLachlan (1, UAIC); BCN, 10.1 mi W of Catavina, 2/24-28/1988, N Bloomfield, green dot (7, SDMC); BCS, 13 mi E of San Ignacio, 3/3/1947, ER Tinkham, mileage 63,817, In pumacy soil by wolf spider hole (1, NMNH); BCN, Arroyo Catavina, 35 mi S of El Progreso, 4/2/1976, Doyen & Rudeblack, light trap (7, EMEC); BCN, El Crucero, 4/3/1976, J Doyen, sifting on sand dunes (1, EMEC); BCN, Punta San Fermin, 4/7-10/71, EL Sleeper, Collected at black light (1, CAS); BCN, Bahia San Luis Gonzaga, 4/3/1973, Doyen,Powell & Szerlip (1, EMEC); BCN, 6 mi. NW Rancho Santa Ynez, 1/?/1976, 29.43.N, 114.43W, 1800’, Dave Ward (1, IMNH); BCN, 22 mi. S of Catavina, 4/4/1982, Faulkner & Brown (10, SDMC); BC, Catavina, 4/13/1957, 29.43N, 114.40W, Farmer, (7, SDMC); BC, 2 mi. N of Catavina, 4/4/1935, 29.45, 114.40, CF Harbison (6, SDMC); BCN, 8 mi. NW of El Progreso, 4/17/1965, Cavagnaro, Ross & Vesterby (2, CAS); BCN, Diablito Canyon, east face Sierra San Pedro Martir, 4/5/1973, SL Szerlipat light (2, EMEC); BCN, 6 mi. NW Rancho Santa Ynez, 4/6/1977, 29.43N, 114.43W, 1800’, WH Clark, night, ex. Opuntia acanthocarpa (1, IMNH); BCN, Diablito Canyon, east face Sierra San Pedro Martir, 4/5-6/1973, J Powell, at light (1, CAS); BCN, San Pedro Martir Diablito Cyn., 3/26-27/1970, 2000’, Gruwell & Perkins, Collected at black light (4, CSLB); BC, Bahia de Los Angeles, 4/15/1947, 28.56 N, CF Harbison, (1 spec), Valle de Amarga (41 spcms.) (41, SDMC); BC, 51 mi. S of Catavina, Hwy. 1, 4/7/1982, Faulkner & Brown, (1, SDMC); BCN, 7 mi. N of Las Arrastras, 6/8/1967, Sleeper & Fisher, Collected at black light (1, CSLB); Lower Cal., Chapala Dry Lake, 6/21/1938, Michelbacher & Ross (1, CAS); Lower Cal., San Quintin, 6/7/1925, HH Keifer (1, CAS); BC, 4 mi. NW Rancho San Juan, 4/3-4/1961, AG Smith, under dung (1, CAS); BCN, 24 mi. N of Ba. San Luis Gonzaga, 4/14/1962, EL Sleeper, Collected at black light (1, CSLB); BCN, Ba. San Luis Gonzaga, 6/17/1970, A Tilzer, BL (1, CSLB); L. Calif., N end of Laguna Salada, 5/8/1939, ES Ross (15, CAS); Baja, N end of Laguna Salada, 5/8/1939, Ross & Michelbacher (4, CAS). All paratypes labeled “Paratype *Arenivaga diaphana* Hopkins 2012” [blue label with black border].

##### Etymology.

The name is an adjective in the nominative singular. This species is named from the Greek meaning diaphanous, light, or fairy-like because of its diaphanous appearance.

##### Distribution.

This species is located in central to southern Baja California Norte, Mexico. See Fig. 44.

##### Diagnosis.

*Arenivaga diaphana* may be distinguished by the long serrated edge on the medial margin of the right dorsal phallomere and the deeply incised central field of the same. See Fig. 44.

##### Description.

**Male.** *Measurements*. Holotype TL = 17.1 mm, GW = 8.5 mm, PW = 4.69 mm, PL = 3.73 mm, TL/GW = 2.01, PL/PW = 0.80 EW = 0.70 mm; OW = 0.30 mm. Among paratypes range of TL 15.5–17.9 mm; range of GW 6.2–7.6 mm; range of PW 3.78–4.69 mm; range of PL 2.88–3.71 mm.

**Figure 42.**
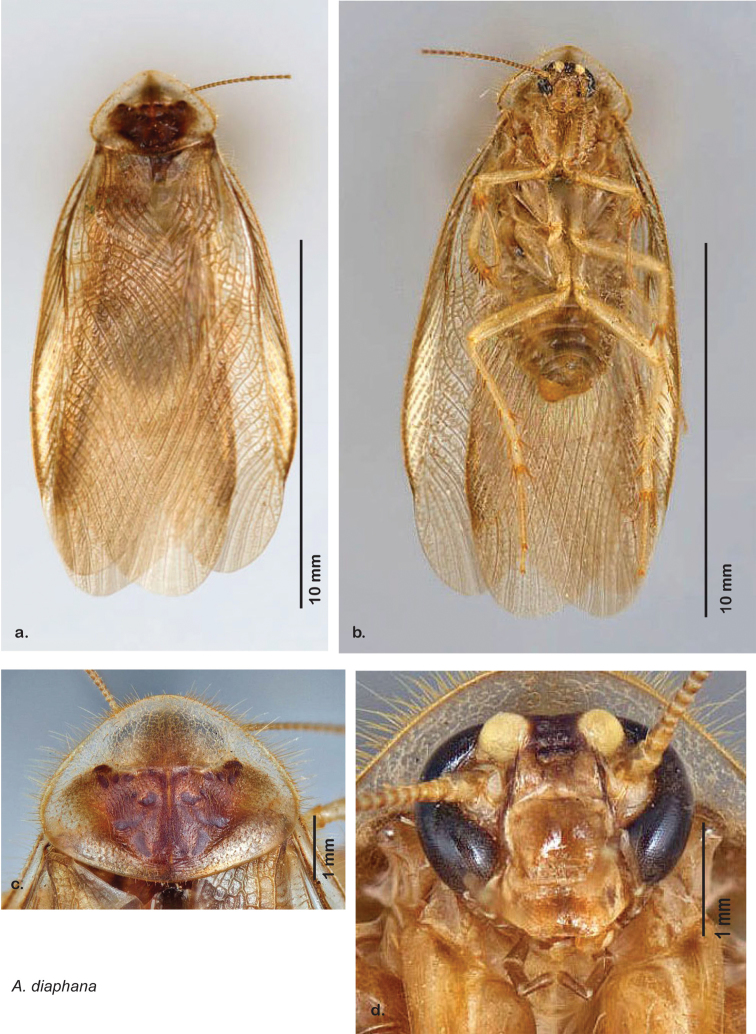
*Arenivaga diaphana*, **a** dorsal habitus **b** ventral habitus **c** pronotum **d** head.

*Head*. Two ocelli very large, ovoid and protruding (0.45 × 0.35 mm); vertex medium brown, with small ridges between apices of eyes and extending onto the ocellar tubercles; scattered small setae; interocellar space concave, medium brown. Frons light brown, concave with wide horizontal indentation posteriorly; anterior portion of frons light brown, bulbous; clypeal suture demarcates light brown anteclypeus; very wide labrum. See Fig. 42d.

*Pronotum*. Pronotum very small, translucent waxy beige; dorsal surface of pronotum covered with short pale gold setae that are longer and thicker laterally; pronotal pattern light yellow to dark brown “panther-face” depending on specimen, impressed, often with aura of a lighter shade. See Fig. 42c.

*Body*. Wing brace present. Two tarsal claws present. Legs and body from light brown to dark brown depending on specimen; subgenital plate color matching body color; angular apices. Uniquely amongst *Arenivaga*, specimens of *diaphana* often have no genicular spines. See Fig. 42b.

*Forewings*. Wings extended beyond abdominal apex (up to ~50% of total wing length); particularly diaphanous with band of darker venation laterally; translucent dark brown and hyaline. See Fig. 42a.

**Figure 43.**
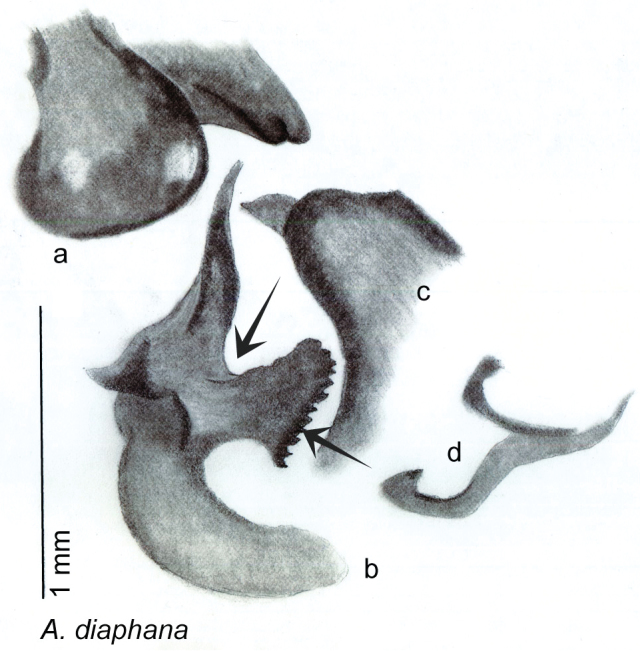
*Arenivaga diaphana*, genitalia: **a** right dorsal phallomere **b** right ventral phallomere **c** small central sclerite. Arrow(s) indicate diagnostic characters (see text).

*Genitalia*. Right dorsal phallomere composed of a bulbous lightly scleritized hook-shaped lobe, articulated with right ventral phallomere on lateral side; central field deeply emarginated anteriorly, remainder lightly scleritized; medial rim more heavily scleritized and toothed. Small right anterior dorsal sclerite attached to hook-shaped lobe of right dorsal phallomere posteriorly, punctate, with sinuous curve over toothed margin of right dorsal phallomere ending in a broad curve; small pointed extension projecting anteromedially from curve. Right ventral phallomere extends from articulation to form smooth rounded lobe, increasingly punctate and scleritized anteriorly; after a wide gap a rounded anteriorly projecting curved concave shagreenous arm, with toothed outer surface. Folded anterior portion of left phallomere narrow, setose, otherwise unmodified. Genital hook has long extension to pointed head and short hook; arm narrow with distinct bend. See Fig. 43.

**Figure 44.**
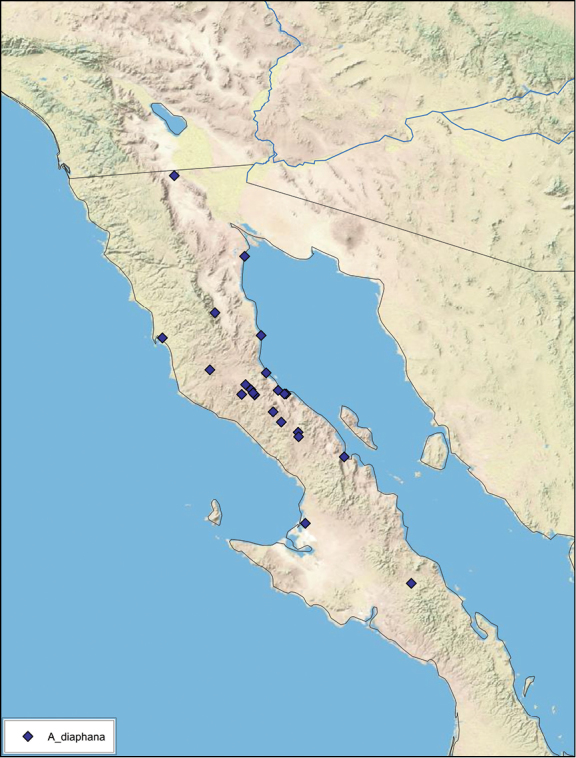
*Arenivaga diaphana*, distribution.

##### Habitat and natural history.

All life history elements remain unobserved.

#### Arenivaga dnopheros sp. n.

http://zoobank.org/58D8A942-BD79-49E1-A463-7F99C937ECBC

http://species-id.net/wiki/Arenivaga_dnopheros

Figures 45 – 47

##### Type locality.

MEXICO, Puebla, 12 mi S of Atlixco.

##### Material examined.

Holotype: ♂ in SEMC labeled “12 mi S Atlixco, Puebla, Mexico, VII-2-1953, 4900’, Univ. Kans. Mex. Expedition” “HOLOTYPE *Arenivaga dnopheros* Hopkins, 2012” [red label with black border].

Paratypes (8): MEXICO: Puebla, 3 mi. NW of Petlalcinqo, 8/29/1972, 4600 ft., Byers & Thornhill, *Arenivaga* nr. *bolliana* det. FW Fisk, 415 (2, SEMC); Puebla, 3 mi. SE of Petlalcingo, 10/6/1986, Miller & Stane (1, FSAC); Puebla, 13.7 mi. SW of Izucar de Matamoros, 7/31/1981, Bogar, Schaffner & Friedlander (1, TAMU); Puebla, 12 mi. S of Atlixco, 7/2/1953, 4900 ft., UK Mex. Expedition (2, SEMC); Puebla, 12 mi. NW of Petlalcingo, 7/3/1953, 4000 ft., UK Mex. Expedition (1, SEMC); Puebla, 11 mi. SE of Acatlan, 7/10/1952, Gilbert & MacNeil (1, EMEC). All paratypes labeled “Paratype *Arenivaga dnopheros* Hopkins 2012” [blue label with black border].

##### Etymology.

The name is an adjective in the nominative singular. This species is named from the Greek meaning dark, gloomy or murky because of its very dark color.

##### Distribution.

This species is found in the state of Puebla, Mexico. See Fig. 47.

##### Diagnosis.

*Arenivaga dnopheros* is very similar to *Arenivaga aquila*. *Arenivaga dnopheros* has larger projection on the left phallomere than *Arenivaga aquila*. The anterior arm of the right ventral phallomere is also much more shagreened with pronounced central indentation. These two characters may be used to distinguish *Arenivaga dnopheros*. See Figs 46 and 28.

##### Description.

**Male.** *Measurements*. Holotype TL = 21.9 mm, GW = 9.6 mm, PW = 6.34 mm, PL = 4.38 mm, TL/GW = 2.28, PL/PW = 0.69. EW = 0.25 mm; OW = 0.60 mm. Among paratypes range of TL 18.5–22.0 mm; range of GW 8.4–10.0 mm; range of PW 5.20–6.70 mm; range of PL 3.62–4.45 mm.

**Figure 45.**
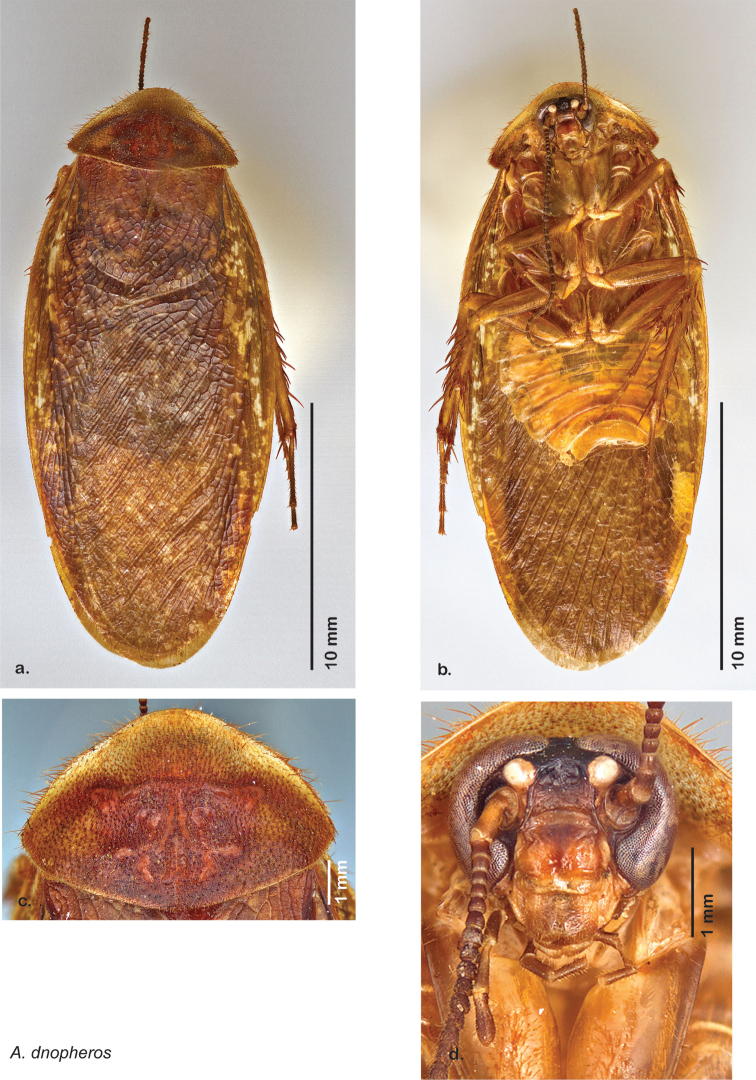
*Arenivaga dnopheros*, **a** dorsal habitus **b** ventral habitus **c** pronotum **d** head.

*Head*. Two ocelli large, ovoid and protruding (0.40 × 0.30 mm); vertex dark brown, with small ridges between apices of eyes and extending onto ocellar tubercles; interocellar space deeply concave, dark to medium brown, with <> shaped indentations. Frons medium brown; posterior concave but tectiform horizontally; anterior portion of frons bulbous but much less so than in most species, medium brown; narrow light brown anteclypeus. See Fig. 45d.

*Pronotum*. Pronotum with translucent waxy beige anterior margin; remainder of pronotum very dark orange-brown and dark brown; dorsal surface of pronotum with dense short orange-brown setae; pronotal pattern “panther face”, impressed; extensive dark aura. See Fig. 45c.

*Body*. Wing brace absent. Two tarsal claws present. Legs and body medium orange-brown; subgenital plate orange-brown; strongly asymmetrical with posterior edge only slightly emarginated and rounded apices. See Fig. 45b.

*Forewings*. Wings extended well beyond abdominal apex (up to ~40% of wing length); blotchy dark brown; surface matte or with very slight sheen and opaque. See Fig. 45a.

**Figure 46.**
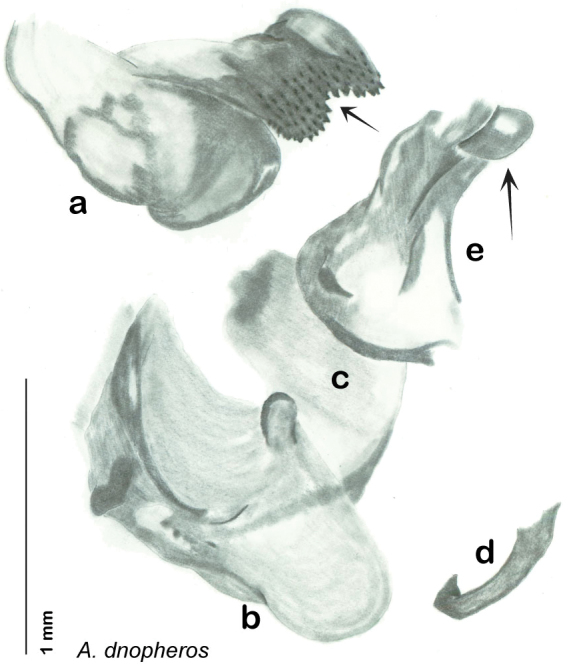
*Arenivaga dnopheros*, genitalia: **a** right dorsal phallomere **b** right ventral phallomere **c** small central sclerite **d** genital hook **e** left phallomere. Arrow(s) indicate diagnostic characters (see text).

*Genitalia*. Right dorsal phallomere composed of lightly sclerotized, narrow bulbous lobe, articulated with right ventral phallomere on lateral side; central field lightly sclerotized, slightly cupped; with narrow medial edge more sclerotized, punctate, ending anteriorly in small shagreened knob. Small central sclerite lightly sclerotized, finely punctate, flat of nondescript shape, posterior end connecting with dorsal side of right dorsal phallomere. Right ventral phallomere arises from deep articulation to form large punctate lobe; anteriorly moderate gap followed by two dorsally projecting sclerotized heavily toothed folds. Folded anterior portion of left phallomere wide, setose, closed at both ends, with small nipple at one end of fold, rough-edged, flattened projection offset at an angle from other end. Genital hook with short extension to rounded head; short hook; arm robust. See Fig. 46.

**Figure 47.**
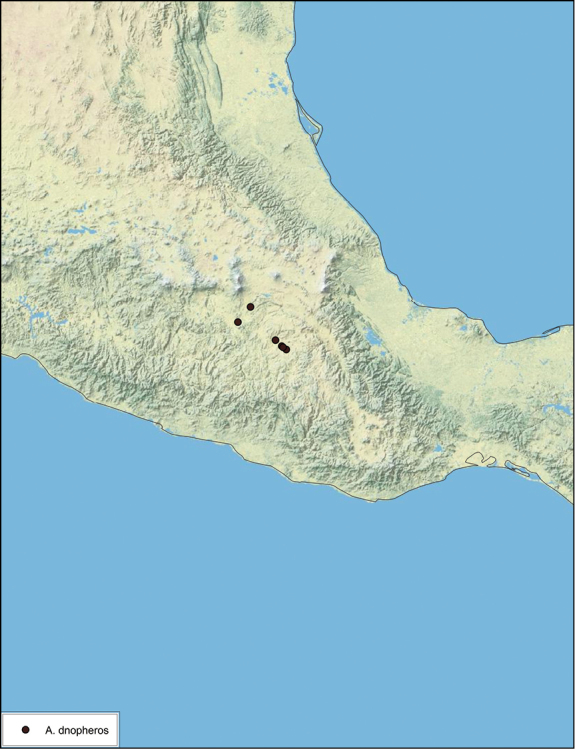
*Arenivaga dnopheros*, distribution.

##### Habitat and natural history.

All life history elements remain unobserved.

#### Arenivaga erratica

Caudell

http://species-id.net/wiki/Arenivaga_erratica

Figures 48 – 50

Homoeogamia (Arenivaga) erratica Rehn 1903, Proceedings of the Academy of Natural Sciences of Philadelphia, 55, pp. 177-192.
Arenivaga erratica (Rehn) 1913, Proceedings of the United States National Museum 44, pp. 595–614.
Arenivaga erratica (Rehn) 1917, Memoirs of the American Entomological Society, 2, pp. 1-284 + plates and index.
Arenivaga erratica (Rehn) 1920, Transactions of the American Entomological Society, 46(2), pp. 197–217.

##### Material examined

**(565).** USA: AZ, Pima Co., 4 mi. NNW Redington, 6/30/1962, Johnson & Smith, UV light trap (1, UAIC); AZ, Pima Co., Tucson Mts., 8/2/1962, D.J. & J.N.Knull, (1, FSCA); AZ, Pima Co., 8 mi. W of Tucson on Hwy. 86, 7/8/1937, Stange & Harding, (1, FSCA); AZ, Pima Co., 16 mi. W of Tucson, 8/13/1988, (1, LACM); AZ, Pima Co., Tucson Mts., 8/2/1962, D.J. & J.N.Knull, (1, OSUC); AZ, Santa Cruz Co., 10 mi. S of Patagonia, 5/27/1964, R.F.Sternitsky, (1, PMNH); AZ, Cochise Co., SWRS 5 mi. W of Portal, 6/28/1963, 5400’, V.Roth, (1, USNM); AZ, Pima Co., Tumamoc Hill, Tucson, 6/19/1967, R.Rice, UV light trap (4, UAIC); AZ, Douglas, 7/2/1963, black light trap,6318364 (1, USNM); AZ, Pima Co., Tucson Mt. Park, Caretaker’s House, 10/19/1981, S.Pechal, (1, UAIC); AZ, Pima Co., Sonoran desert Museum, 8/5-8/1962, W.L.Nutting, light trap, Soman (2, UAIC); AZ, Pima Co., Sonoran desert Museum, 8/21-24/1962, W.L.Nutting, light trap, Soman (3, UAIC); AZ, Pima Co., Sonoran desert Museum, 8/1-4/1962, W.L.Nutting, light trap, Soman, (1, HEH); AZ, Pima Co., Sonoran desert Museum, 8/5-8/1962, W.L.Nutting, light trap, Soman, (2, HEH); AZ, Ft. Grant, ?/?/1882, C.V.Riley, (1, USNM); AZ, Cochise Co., 5131 Bannock Street, Pueblo Del Sol, Huachuca Mts., 9/24/1985, R.S.Wielgus, UV light trap (1, ASUT); AZ, Cochise Co., 5131 Bannock Street, Pueblo Del Sol, Huachuca Mts., 6/5/1985, R.S.Wielgus, UV light trap (1, ASUT); AZ, Cochise Co., 1 mi. NE of Portal, 9/7/1959, J.M.Burns, (1, EMEC); AZ, Cochise Co., 4 mi. E of Portal, 8/21/1971, J.Doyen, J.Doyen Lot #71H6 outside enclosures (1, EMEC); AZ, Cochise Co., Chiricahua Mts., Portal Ranger Station, 7/30/1966, 4950’, R.G.Beard, UV light trap (1, CUIC); AZ, Cochise Co., SWRS, Portal, 9/5/1970, L.D. & M.D. Anderson, (1, UCRC); AZ, Cochise Co., Cave Creek Ranch, 8/21/1974, E.G. & J.M. Linsley, (1, EMEC); AZ, Pima Co., Peppersauce Canyon, Santa Catalina Mts., 8/17/1924, J.O.Martin, (1, CAS); AZ, Pima Co., Stratton, S Catalina Mts., 7/27/1917, 6-7000’, Wheeler, erratica (1, MCZ); AZ, Pima Co., Sabino Canyon, Santa Catalina Mts., 11/2/1915, J.F.Tucker, (1, ANSP); AZ, Pima Co., Sabino Canyon, Santa Catalina Mts., 7/12/1932, R.H.Beamer, (1, ANSP); AZ, Cochise Co., Portal, 6/17/1956, O.L.Cartweight, (1, USNM); AZ, Cochise Co., Portal, 6/18/1956, R. & K. Dreisbach, Dreisbach Collection return to Crandall (1, UMMZ); AZ, Benson, 9/?/1949, (1, EMEC); AZ, Cochise Co., Portal, 6/27/1963, A.Raske, at light (7, EMEC); AZ, Cochise Co., Portal, 7/18-9/1/1971, J.Doyen, pitfall trap, J.Doyen Lot #71G15 (2, EMEC); AZ, Cochise Co., 1 mi. S of Portal, 8/11/1965, 4800’, Davidson, Davidson & Cazier, at light (1, ASUT); AZ, Cochise Co., 1 mi. S of Portal, 6/23/1965, 4800’, Davidson,Davidson & Cazier, at light (1, ASUT); AZ, Cochise Co., 1 mi. S of Portal, 7/2/1965, 4800’, Davidson,Davidson & Cazier, at light (1, ASUT); AZ, Cochise Co., 1 mi. S of Portal, 7/18/1965, 4800’, Davidson,Davidson & Cazier, at light (1, ASUT); AZ, Cochise Co., 1 mi. S of Portal, 7/7/1965, 4800’, Davidson,Davidson & Cazier, at light (1, ASUT); AZ, Cochise Co., 1 mi. S of Portal, 7/16/1965, 4800’, Davidson,Davidson & Cazier, at light (1, ASUT); AZ, Cochise Co., 1 mi. S of Portal, 7/23/1965, 4800’, Davidson,Davidson & Cazier, at light (1, ASUT); AZ, Cochise Co., 1 mi. S of Portal, 7/8/1965, 4800’, Davidson,Davidson & Cazier, at light (1, ASUT); AZ, Cochise Co., 1 mi. S of Portal, 7/5/1965, 4800’, Davidson,Davidson & Cazier, at light (2, ASUT); AZ, Cochise Co., 1 mi. S of Portal, 7/4/1965, 4800’, Davidson,Davidson & Cazier, at light (1, ASUT); AZ, Cochise Co., 1 mi. S of Portal, 7/28/1965, 4800’, Davidson,Davidson & Cazier, at light (1, ASUT); AZ, Cochise Co., 1 mi. S of Portal, 6/22/1965, 4800’, Davidson,Davidson & Cazier, at light (1, ASUT); AZ, Cochise Co., Portal, 8/25/1964, L.D.Anderson, light (1, UCRC); AZ, San Simon, 5/?/1907, Hubbard, (1, USNM); AZ, Cochise Co., Portal, 6/4/1917, L.A.Stange, UV light trap (1, LACM); AZ, Cochise Co., Douglas, 10/7/1955, Truxal & Martin, (1, LACM); AZ, Cochise Co., Portal, 6/15/1959, L.A.Stange, (1, LACM); AZ, Cochise Co., San Bernardino Ranch, 13 mi. E of Douglas, 6/12/1959, L.A.Stange, (2, LACM); AZ, Cochise Co., 7/20/1927, R.H.Beamer, (1, SEMC); AZ, Cochise Co., San Bernardino Ranch, 8/?/????, 3750’, F.H.Snow, (1, SEMC); AZ, Mescal, 7/28/1927, L.A.Anderson, (1, SEMC); AZ, Benson, 8/5-6/1947, E.R.Tinkham, E.R.T., (2, HEH); AZ, Benson, 7/4/1947, E.R.Tinkham, E.R.T., (1, HEH); AZ, Benson, 10/10/1947, E.R.Tinkham, (1, USNM); AZ, Pima Co., Tucson, 7/16/1953, R.S.Beal, (1, EMEC); AZ, Cochise Co., Portal, 7/1/1964, 4700’, Puckle,Mortenson & Cazier, at light (19, ASUT); AZ, Cochise Co., Portal, 6/16/1964, 4700’, Puckle,Mortenson & Cazier, at light (7, ASUT); AZ, Cochise Co., Portal, 6/17/1864, 4700’, Puckle,Mortenson & Cazier, at light (5, ASUT); AZ, Cochise Co., Portal, 7/2/1964, 4700’, Puckle,Mortenson & Cazier, at light (6, ASUT); AZ, Cochise Co., Portal, 6/21/1964, 4700’, Puckle,Mortenson & Cazier, at light (2, ASUT); AZ, Cochise Co., Portal, 6/10/1964, 4700’, Puckle, Mortenson & Cazier, at light (3, ASUT); AZ, Cochise Co., Portal, 6/28/1964, 4700’, Puckle,Mortenson & Cazier, at light (4, ASUT); AZ, Cochise Co., Portal, 6/7/1964, 4700’, Puckle,Mortenson & Cazier, at light (1, ASUT); AZ, Cochise Co., Portal, 6/12/1964, 4700’, Puckle,Mortenson & Cazier, at light (2, ASUT); AZ, Cochise Co., Portal, 8/23/1982, R.A.Cunningham, (2, PMNH); AZ, Cochise Co., Portal, 6/25/1964, 4700’, Puckle,Mortenson & Cazier, at light (3, ASUT); AZ, Cochise Co., Portal, 6/29/1964, 4700’, Puckle, Mortenson & Cazier, at light (1, ASUT); AZ, Cochise Co., Portal, 6/22/1964, 4700’, Puckle,Mortenson & Cazier, at light (2, ASUT); AZ, Cochise Co., Portal, 7/15/1964, 4700’, Puckle,Mortenson & Cazier, at light (1, ASUT); AZ, Cochise Co., Portal, 6/2/1964, 4700’, Puckle,Mortenson & Cazier, at light (1, ASUT); AZ, Cochise Co., Portal, 6/3/1964, 4700’, Puckle,Mortenson & Cazier, at light (1, ASUT); AZ, Cochise Co., Portal, 6/23/1964, 4700’, Puckle,Mortenson & Cazier, at light (5, ASUT); AZ, Cochise Co., Portal, 6/20/1964, 4700’, Puckle,Mortenson & Cazier, at light (2, ASUT); AZ, Cochise Co., Portal, 6/19/1964, 4700’, Puckle,Mortenson & Cazier, at light (1, ASUT); AZ, Cochise Co., Portal, 7/3/1964, 4700’, Puckle,Mortenson & Cazier, at light (4, ASUT); AZ, Cochise Co., Portal, 8/16/1964, 4700’, Puckle,Mortenson & Cazier, at light (2, ASUT); AZ, Cochise Co., Portal, 7/25/1964, 4700’, Puckle,Mortenson & Cazier, at light (1, ASUT); AZ, Cochise Co., Portal, 7/23/1964, 4700’, Puckle,Mortenson & Cazier, at light (1, ASUT); AZ, Cochise Co., SWRS Portal, Chiricahua Mts,, 8/8/1968, L.D.Anderson, (1, UCRC); AZ, Cochise Co., US 666 4 mi. N of Sunsites, 8/16/1989, Skelley & Mason, street light (1, FSCA); AZ, Cochise Co., Portal, 6/26/1964, 4700’, Puckle,Mortenson & Cazier, at light (1, ASUT); AZ, Cochise Co., Portal, 6/24/1964, 4700’, Puckle,Mortenson & Cazier, at light (2, ASUT); AZ, Cochise Co., Portal, 6/15/1964, 4700’, Puckle,Mortenson & Cazier, at light (4, ASUT); AZ, Cochise Co., Portal, 6/14/1964, 4700’, Puckle,Mortenson & Cazier, at light (1, ASUT); AZ, Cochise Co., Portal, 6/13/1964, 4700’, Puckle,Mortenson & Cazier, at light (4, ASUT); AZ, Cochise Co., Portal, 6/27/1964, 4700’, Puckle,Mortenson & Cazier, at light (2, ASUT); AZ, Cochise Co., Portal, 6/9/1964, 4700’, Puckle, Mortenson & Cazier, at light (1, ASUT); AZ, Cochise Co., Portal, 6/8/1964, 4700’, Puckle,Mortenson & Cazier, at light (1, ASUT); AZ, Cochise Co., Portal, 6/11/1964, 4700’, Puckle,Mortenson & Cazier, at light (1, ASUT); AZ, Cochise Co., Portal, 6/18/1964, 4700’, Puckle,Mortenson & Cazier, at light (2, ASUT); AZ, Cochise Co., Portal, 6/5/1964, 4700’, Puckle,Mortenson & Cazier, at light (1, ASUT); AZ, Cochise Co., Portal, 6/4/1964, 4700’, Puckle,Mortenson & Cazier, at light (2, ASUT); AZ, Cochise Co., Portal, 7/7/1967, (1, USNM); AZ, Cochise Co., Portal, 7/18/1973, S.Frommer, (1, UCRC); AZ, Cochise Co., Texas Canyon, L.Dragoon Mts., 7/22/1983, Olson & Burns, (2, UAIC); AZ, Cochise Co., 32 mi. E of Douglas, 8/18/1967, (1, USNM); AZ, Cochise Co., Douglas, 7/12/1971, S.McCleve, at light (1, UAIC); AZ, Cochise Co., San Simon, M.J.Westfall, at light, for report to Fla.Pl.Board (1, USNM); AZ, Paradise, 8/?/????, Wickham collection 1933 (1, USNM); AZ, Cochise Co., 4 km. SW of Benson, 6/4/1991, L.R.Davis,Jr., T17S R20E Sec.20 (2, FSCA); AZ, Benson, 8/2/1941, R.A.Flock, (1, UCRC); AZ, Benson, 7/2/1941, R.A.Flock, (1, UCRC); AZ, Benson, 6/19/1941, R.A.Flock, (1, UCRC); AZ, Benson, 7/24/1941, R.A.Flock, (1, UCRC); AZ, San Carlos Reservation, 6/30/1967, (1, USNM); AZ, Cochise Co., San Pedro Reservation near Hereford, 8/20/1994, C.A.Olson, (2, UAIC); AZ, Chiricahua Mts., 8/28/1962, D.J. & J.N.Knull, (2, FSCA); AZ, Chiricahua Mts., 7/5/1949, D.J. & J.N.Knull, (1, FSCA); AZ, Cochise Co., SWRS Chiricahua Mts., 8/25/1968, L.D.Anderson, at lights (1, UCRC); AZ, Pima Co., Santa Rita Mts., Sycamore Canyon, 6/29-7/8/1981, J.C.Burne, Anamax Survey, illegible (2, UAIC); AZ, Apache Co., Canyon De Chelly NM, 8/18/2005, N.Cobb, (1, NAUF); AZ, Chiricahua Mts., 8/24/1962, D.J. & J.N.Knull, (2, OSUC); AZ, Chiricahua Mts., 7/14/1936, J.N.Knull, (3, OSUC); AZ, Chiricahua Mts., 7/15/1953, D.J. & J.N.Knull, (2, OSUC); AZ, Chiricahua Mts., 9/19/1947, D.J. & J.N.Knull, (1, OSUC); AZ, Chiricahua Mts., 7/22/1957, D.J. & J.N.Knull, (1, OSUC); AZ, Chiricahua Mts., 7/27/1957, D.J. & J.N.Knull, (2, OSUC); AZ, Huachuca Mts., 7/20/1937, D.J. & J.N.Knull, (1, OSUC); AZ, Chiricahua Mts., 7/22/1953, D.J. & J.N.Knull, (1, OSUC); AZ, Chiricahua Mts., 7/16/1959, D.J. & J.N.Knull, (1, OSUC); AZ, Chiricahua Mts., 7/29/1961, D.J. & J.N.Knull, (1, OSUC); AZ, Chiricahua Mts., 8/21/1962, D.J. & J.N.Knull, (1, OSUC); AZ, Chiricahua Mts., 7/23/1959, D.J. & J.N.Knull, (1, FSCA); AZ, Santa Cruz Village, Cobabi Mts., 8/10-12/1916, 32.1N, 111.54W, 3100’, (1, ANSP); AZ, Douglas, F.H.Snow, (1, ANSP); AZ, Santa Cruz Co., Badger, 7/31/1924, J.O.Mastio, (1, CAS); AZ, Chiricahua Mts., 6/3/1935, J.N.Knull, (1, OSUC); AZ, Prescott, 6/10/1902, Oslar, Homoeogamia erratica Paratype, genitalia figured H1920 (1, ANSP); NM, 10 mi. E of Deming, 7/12/1917, Cornell U. Lot 882,Sub.145 (1, CUIC); NM, Mesille Park, 7/12/1917, Cornell U. Lot 677,Sub 547 (1, CUIC); TX, Brewster Co., Hills W of Ord Mts., 8/1-15/1926, O.C.Poling, (2, ANSP); TX, Belfrage, (1, USNM); TX, Reeves Co., Pecos, 8/17/1935, T.H. & G.G.Hubbell, (1, UMMZ); TX, Brewster Co., Marathon, 9/26/1950, 4000’, Gertech & Cazier, (1, AMNH); TX, Culberson Co., Van Horn, 7/10/1948, C. & P. Vaurie, (2, AMNH); TX, Culberson Co., Van Horn, 7/10/1950, R.F.Smith, (4, AMNH); TX, El Paso Co., 7/17/1927, P.A.Radio, (1, SEMC); TX, Valentine, 7/12/1958, R.I.Sailer, (1, SEMC); TX, Valentine, 7/8/1917, (1, ANSP); TX, Hudspeth Co., McNary, 7/14/1948, Nutting & Werner, at light, mesquite area, W.L.N. (6, UAIC); TX, Terrell Co., Lester Canyon, 7/8/1948, 1360’, Nutting & Werner, at light desert, W.L.N. (4, UAIC); TX, Brewster Co., S.G.Ranch, 3/1-15/1926, O.C.Poling, (1, UMMZ); TX, Brewster Co., S.G.Ranch, 4/15-30/1926, O.C.Poling, (1, UMMZ); TX, Brewster Co., Terlingua, 5/3/1927, J.O.Martin, (1, CAS); TX, Brewster Co., Terlingua, 7/9/1994, W.F.Chamberlain, at light (1, TAMU); TX, Jeff Davis Co., Point of Rocks Rest Stop, 8/10/1992, Godwin & Riley, at UV light (1, TAMU); TX, Brewster Co., Hackberry Creek, Boquillas Road, 9/2/1912, Rehn & Hebard, one-figured H1917 (3, ANSP); TX, Brewster Co., Big Bend NP, Castalon area, Cottonwood Cpgd, 8/1/2003, 30.12.24N 103.14.14W, E.Riley, uv light (1, TAMU); TX, Brewster Co., Hills W of Ord Mts., 9/15-20/1926, O.C.Poling, (1, ANSP); TX, Brewster Co., Hills W of Ord Mts., ?/?/1928, O.C.Poling, (4, ANSP); TX, Brewster Co., Hills W of Ord Mts., 8/22-31/1926, O.C.Poling, (6, ANSP); TX, Brewster Co., Hills W of Ord Mts., 6/1-15/1926, O.C.Poling, (1, ANSP); TX, Brewster Co., Hills W of Ord Mts., 9/1-16/1926, O.C.Poling, (1, ANSP); TX, Davis Mts., 7/16/1946, E.C.VanDyke, (1, CAS); TX, Davis Mts., 5/27/1935, J.N.Knull, (1, OSUC); TX, Culberson Co., 3 mi. E of Van Horn, 8/14/1965, J.C.Schaffner, at light (1, TAMU); TX, Presidio, 9/5/1949, at light (3, USNM); TX, Culberson Co., Van Horn, 7/1/1947, B.Malkin, (2, USNM); TX, Presidio, 7/5/1945, Presidio 1334,lights on screen at residence, Lot No.45-16511 (2, USNM); TX, Presidio, 10/1/1947, lights at screen (1, USNM); TX, Presidio, 7/22/1944, lights at screen door (3, USNM); TX, Presidio, 6/6-20/1947, J.H.Russell, at light (2, USNM); TX, Presidio, 5/14/1944, (1, USNM); TX, Presidio, 3/14/1946, lights on screen at residence (1, USNM); TX, Presidio, 8/1/1953, at light (19, USNM); TX, Presidio, 3/26-5/15/1951, J.H.Russell, at lights (1, USNM); TX, Presidio, 2/18/1948, at light (4, USNM); TX, Presidio, May-June 1953, at light (38, USNM); TX, Presidio, 7/1-25/1951, at lights, *Arenivaga apacha* Sauss. Det.Gurney (2, USNM); TX, Presidio, 6/6/1957, J.H.Russell, at lights (1, USNM); TX, Presidio, 5/5/1945, at light (1, USNM); TX, 4 mi. N of El Paso, 8/30/1951, at lights (7, USNM); TX, Presidio, 2/28/1951, at lights (3, USNM); TX, Presidio, 2/2/1950, collected at light, *Arenivaga apacha* (Sauss) det. Gurney (1, USNM); TX, Presidio, 7/24/1948, collected at light (1, USNM); TX, Presidio, 10/15/1944, night on screen (1, USNM); TX, Presidio, June-July 1947, J.H.Russell, at lights (1, USNM); TX, Valentine, 7/13/1927, R.H.Beamer, (1, ANSP); TX, Koebele Collection, Heterogamia sp. (1, CAS); TX, McKelligan Canyon, El Paso, 6/15/1948, H.S.Barber et al., under stone (1, USNM); TX, Jeff Davis Co., Rest stop 9.5 mi. S of jct. Hwy 118 on 166, 8/9/1992, Godwin & Riley, uv light (1, TAMU); TX, El Paso, 7/14/1947, C.F.Haller, in Japanese Beetle trap at municipal airport (1, USNM); TX, Presidio, 7/12/1950, J.H.Russell, in Japanese Beetle trap at end of RR bridge (1, USNM); TX, 4 mi. N of El Paso, 8/30/1951, at lights (1, AMNH); NM, Hidalgo Co., Double Adobe Ranch, Animas Mts., 8/15/1952, 5500 ft., Leech & Green, (2, CAS); NM, Silver City, 9/2/1960, HG Rodeck, (1, UCMC); NM, Dona Ana Co., Anthony US 10 rest stop, 8/23-24/1997, 32.00675N, 106.58138W, Scott & Powers, at lights (1, UCMC); NM, Gage, 7/12/1952, RH & LD Beamer,LaBerge & Liang, (1, SEMC); NM, Chaco Canyon NM, 7/20/1962, SF Wood, (1, LACM); NM, San Juan Co., Ship Rock, 1986/1987, 6500 ft., D.Giuliani, Antifreeze pit trap (1, CSCA); NM, Bernalillo Co., Albuquerque, 9/18/1944, 5000 ft., WO Griesel, at lights (3, LACM); NM, Hidalgo Co., San Simon Valley, Jct. of State Line Rd and Rt.533, 7/8/1992, 4250 ft., SP Cover, 2 ft. deep in active Bannertail Kangaroo rat mound, open desert, mesquite & ephedra (2, MCZ); NM, Hidalgo Co., 9 mi. NW of Rodeo, 7/2/1973, 1300m, M Masters, Polyphagidae (1, CUIC); NM, Dona Ana Co., Pyramid Peak, FR Fosberg, Museum Coll.9076 (1, LACM); NM, Dona Ana Co., Pyramid Peak, 8/1/1930, FR Fosberg, Museum Coll.9686 (1, LACM); NM, Dona Ana Co., Pyramid Peak, 8/30/1930, FR Fosberg, Museum Coll.9685 (3, LACM); NM, Dona Ana Co., Pyramid Peak, 8/21/1930, FR Fosberg, Museum Coll.9279 (2, LACM); NM, Dona Ana Co., Pyramid Peak, 8/30/1930, FR Fosberg, Museum Coll.9685 (1, LACM); NM, SanJon, 7/31/1938, RP Allen, (1, CAS); NM, Eddy Co., White’s City Cpgd.Guadalupe Mts., 7/21/1989, JP & KES Donahue, #136,564 (2, LACM); NM, Bent, 7/1-15/1927, OC Poling, (3, ANSP); NM, Las Cruces, TDA Cockerell, (1, ANSP); NM, Luna Co., Deming, 7/19/1907, 4315 ft., Hebard & Rehn, at lights (8, ANSP); NM, Bent, 6/15-30/1927, OC Poling, (1, ANSP); NM, No.262 (1, USNM); NM, Hidalgo Co., Rodeo, 9/4-8/1959, JM Burns, (3, EMEC); NM, Hidalgo Co., Rodeo, 9/8/1959, DD Linsdale, (3, EMEC); NM, Las Cruces, 9/?/1895, Ckll 4713 (1, USNM); NM, Virden, 6/12/1959, GL Nielsen, *Arenivaga apacha* (Sauss.)det.AB Gurney 1959 (1, USNM); NM, Eddy Co., Site 7, 9/24/1979, 32.19.8N 103.47.3W, Murray & Schaffner, at lights (3, TAMU); NM, Socorro Co., La Sevilleta, 9/13/2008, 34.354N, 106.885W, EI Rodriguez, (1, NAUF); NM, Socorro Co., Sevilleta NWR, 9/9-23/2008, 34.3431N, 106.7417W, K.Wetherill, Creosote shrubland, Buchmann Funnel trap (2, MSB); NM, Socorro Co., Gran Quivira NM, 7/1-3/1964, 6600 ft., DR Davis, (1, USNM); NM, 2 mi. E of Tesuque Pueblo, 8/15/1934, 7000 ft., M.Hebard, (1, ANSP); NM, Mesille Park, 7/12/1917, (1, ANSP); NM, Roosevelt Co., Oasis SP, 8/31/1971, 4100 ft., Brown & Petrulis, at lights (2, PMNH); NM, University Park, 6/25/1960, A.Ross, (1, UAIC); NM, Lea Co., site 14, 9/23/1979, 32.22.8N 103.43.3W, Murray & Schaffner, at lights (1, TAMU); NM, McKinley Co., 5 mi. N of Tohatchi, 8/14/1948, Nutting & Werner, at lights, dry grass & juniper, sketch of genitalia, WLN (1, UAIC); NM, Hidalgo Co., 13 mi. N of Rodeo, 6/16/1956, E Ordway, (1, AMNH); NM, Eddy Co., Carlsbad Cavern, 8/15-18/1935, TH & GG Hubbell, (1, UMMZ); NM, Hidalgo Co., Rodeo, 6/8/1959, LA Stange, (1, LACM); NM, Luna Co., Deming, 7/2/1937, Burt, (1, USNM); NM, Luna Co., Deming, 7/12/1917, Wheeler, *Arenivaga* sp. near *rehni* det. TH Hubbell 1928 (1, UMMZ); NM, No.262 (1, AMNH); NM, Santa Fe, pack rat nest, 8/?/1961, B Miller, (1, USNM); NM, Sevilleta NWR, 9/10/2001, deep well fire site south transect, pitfall trap #14 (2, MSB); NM, Sevilleta NWR,McKenzie Flats, 8/10/2001, transect E km 1,West side juniper trap #1 (1, MSB); NM, Sevilleta NWR,McKenzie Flats, 8/10/2001, transect E km 0,East side juniper trap #3 (1, MSB); NM, Sevilleta NWR,McKenzie Flats, 6/26/2001, transect E km 1,East side juniper trap #3 (1, MSB); NM, Sevilleta NWR,McKenzie Flats, 9/10/2001, transect mid km 2,East side pitfall trap #2 (1, MSB); NM, Sevilleta NWR,McKenzie Flats, 10/8/2001, transect mid km 6,West side [pitfall trap#1 (1, MSB); NM, Sevilleta NWR,McKenzie Flats, 7/20/2001, transect mid km 4,East side pitfall trap #2 (1, MSB); NM, Socorro Co., Sevilleta NWR, 7/20/2001, South Transect pitfall trap #13 (1, MSB); NM, Socorro Co., Sevilleta NWR, 7/20/2001, deep well fire site north transect, pitfall trap #4 (1, MSB); NM, Socorro Co., Sevilleta NWR, 9/10/2001, deep well fire site north transect, pitfall trap #14 (1, MSB); NM, Socorro Co., Sevilleta NWR, 9/10/2001, deep well fire site south transect, pitfall trap #4 (1, MSB); TX, Presidio, 8/1/1953, collected at light, *Arenivaga apacha* (Sauss) det. Gurney (1, USNM); TX, Presidio, 7/11/1968, JE Hafernik, black light (2, TAMU); TX, Presidio Co., Big Bend Ranch St. Nat.Ar.,Aqua Adentro, 6/18-23/1990, D Judd, malaise trap (1, TAMU); TX, Presidio, 8/14/1928, ER Tinkham, (1, ANSP); TX, 3 mi. E of Van Horn, 8/14/1965, J.C.Schaffner, at light (1, TAMU); TX, Brewster Co., 22 mi. S of Marathon, 9/3/1960, L.A.Stange, (1, FSCA); TX, Jeff Davis Co., Fort Davis, 10/15-27/1927, 5000’, O.C.Poling, (2, UMMZ); TX, Alpine, 6/4/1952, J.E.Elkins, (1, SEMC); UT, Utah Co., Moab, Slick Rock Cmpgd., 9/5/1999, S. Belnap, (1, MLBM); UT, San Juan Co., Cottonwood Wash 10 mi SW Blanding, (1, MLBM); AZ, Navajo Co., along Rt. 87 N of Winslow, 8/9/2012, N35.04975 W110.59804, 1525 m, H Hopkins, HEH, (1, ); AZ, Cochise Co., Bar Boot Ranch, 9/?/2010, LJ Vitt & JP Caldwell, *Arenivaga* sp. det. K. Menard 2012, Catalog No. OMNH-21117, 21118, 21119 (3, OMNH); AZ, Cochise Co., 3 mi N Douglas, 8/17/1968, V. Roth, at light (1, FSAC); AZ, Cochise Co., SWRS 5 mi W Portal, 9/5/1963, 5400’, (1, SWRS); TX, Brewster Co., Big Bend NP, 7/8/1961, RH Arnett Jr. & E Van Tassell, Lot No. 475 (1, FSAC); AZ, Pima Co., in Sabino Cn., 7/1/1959, RH Arnett Jr., Lot No. 386 (1, FSAC); AZ, Pima Co., in Sabino Cn., 7/9/1959, RH Arnett Jr., Lot No. 399 (1, FSAC); AZ, Cochise Co., near Fairbanks, 6/29/1973, S. McCleve, lite (2, FSAC); AZ, Cochise Co., Portal, 6/25/1966, VD Roth, (1, SWRS); AZ, Cochise Co., Portal, 7/19/1964, VD Roth, *Arenivaga apacha* (Sauss.) (1, SWRS); NM, Hidalgo Co., Rodeo, 6/29/1971, (1, SWRS); NM, Rodeo, 9/17/1963, VD Roth, (1, SWRS); NM, Artesia, 7/19/1962, JR Eyer, (1, NMSU); NM, Carlsbad, 6/22/1964, (2, NMSU); NM, Dona Ana Co., Jornada Expt. Range, 7/28/1971, Ellstrom, 1 specimen-att. to light (2, NMSU); NM, Dona Ana Co., Mouth Baylor Can., 8/4-5/1979, 5100’, C. Ferris, (2, FSAC);MEXICO, Chihuahua, Samalayuca, 6/24/1947, Schramel, D. Rockefeller Exp. (24, AMNH); Chihuahua, Samalayuca, 6/24/1947, Gertsch, D. Rockefeller Exp. (2, AMNH); Chihuahua, Samalayuca, 6/24/1947, Cazier, D. Rockefeller Exp. (4, AMNH); Chihuahua, Samalayuca, 6/24/1947, Spieth, D. Rockefeller Exp. (10, AMNH); Chihuahua, Samalayuca, 6/24/1947, Michener, D. Rockefeller Exp. (1, AMNH); Durango, 2 mi. S of Menores de Arriba, 9/14/1950, RF Smith, (2, AMNH); Chihuahua, Samalayuca Dunes,33 mi. S of Ciudad Juarez, 6/25/1959, ER Tinkham, (2, USNM); Chihuahua, 16 mi. SE of Chihuahua, 7/27/1953, 4000 ft., SEMC Mex.Expedition (2, SEMC); Chihuahua, Ojo Laguna, 6/30/1947, Gertsch, D. Rockefeller Exp. (1, AMNH); Chihuahua, Primavera, 6/30/1947, 5500-6000 ft., Cazier, D. Rockefeller Exp. (1, AMNH); Chihuahua, Namiquipa Dist., 7/3/1947, 6500 ft., Cazier, D. Rockefeller Exp. (1, AMNH); Chihuahua, 15 mi. E of Parral, 7/15/1947, 5500 ft., Cazier, D. Rockefeller Exp. (1, AMNH); Chihuahua, 15 mi. E of Parral, 7/15/1947, 5500 ft., Schramel, D. Rockefeller Exp. (1, AMNH); Chihuahua, 8 mi. S of Gallego, 7/27/1953, 5000 ft., SEMC Mex.Expedition (1, SEMC); Chihuahua, Colonia Dublan, ?/?/1931, Beck & Call, (1, HEH); Chihuahua, 25 mi. SW of Camargo, 7/14/1947, Gertsch, D. Rockefeller Exp. (2, AMNH); Chihuahua, 25 mi. SW of Camargo, 7/14/1947, Michener, D. Rockefeller Exp. (1, AMNH); Chihuahua, 10 mi. N of Jimenez, 9/10/1950, RF Smith, (2, AMNH); Coahuila, 6 mi. W of Matamoros, 6/8/1961, UCM EI MEI (1, UCMC); Chihuahua, Catarinas, 7/25/1947, 5800 ft., Michener, D. Rockefeller Exp. (1, AMNH); Chihuahua, Catarinas, 7/25/1947, 5800 ft., Gertsch, D. Rockefeller Exp. (1, AMNH); Chihuahua, 20 mi. SW of Camargo, 7/13/1947, 4500 ft., Michener, D. Rockefeller Exp. (2, AMNH); Chihuahua, 20 mi. SW of Camargo, 7/13/1947, 4500 ft., Spieth, D. Rockefeller Exp. (1, AMNH); Durango, Durango, 8/4/1951, PD Hurd, (3, EMEC); Durango, 9 mi. W of Durango, 9/6/1950, RF Smith, (1, AMNH); Durango, 41 mi. NE of Durango, 7/28/1956, RE Beer & party, at light (1, SEMC); Chihuahua, 63 mi. W of Santa Barbara, 7/20/1947, 5500 ft., Cazier, D. Rockefeller Exp. (2, AMNH); Chihuahua, 13 mi. E of Cuauhtemoc, 7/11/1964, 6600ft, Chemsak & Powell, at lights (2, EMEC); Chihuahua, Chihuahua, 8/26/1981, Chemsak, at lights (1, EMEC); Chihuahua, Hwy.45 vicinity of Laguna Encinillas, 7/1/1987, GF Ballmer, at lights (1, UCRC); Chihuahua, Camargo, 6/16/1965, SJ Arnold, at lights (1, EMEC); Chihuahua, 15 mi S El Sueco, 9/?/1983, Gary Fritz, (1, NMSU); Chihuahua City, 7/10/1983, Gary Fritz, (1, NMSU); Chihuahua, Colonia Dublan, Anson Ca?l Jr, *Arenivaga apacha* (Sauss.) det. ABGurney ‘53 (1, MLBM). Determiner label *Arenivaga erratica* Hopkins 2011” [white label with black border].

##### Distribution.

This is a widespread species widely in New Mexico, the eastern half of Arizona, the far western part of Texas located south of New Mexico, south into central Mexico. Isolated records from Utah, Nevada, and a record from Colorado not included in the distribution map. See Fig. 50.

##### Diagnosis.

*Arenivaga erratica* may be diagnosed by shagreened or toothed ridge running along the anterolateral edge of the small dorsal sclerite. See Fig. 49.

##### Description.

**Male.** *Measurements*. Holotype stand-in TL = 17.3 mm, GW = 8.8 mm, PW = 5.73 mm, PL = 3.76 mm, TL/GW = 1.97, PL/PW = 0.66. EW = 0.25 mm; OW = 0.40 mm. Among paratypes range of TL 16.3–23.5 mm; range of GW 7.4–10.0 mm; range of PW 5.10–6.62 mm; range of PL 3.76–4.70 mm.

**Figure 48.**
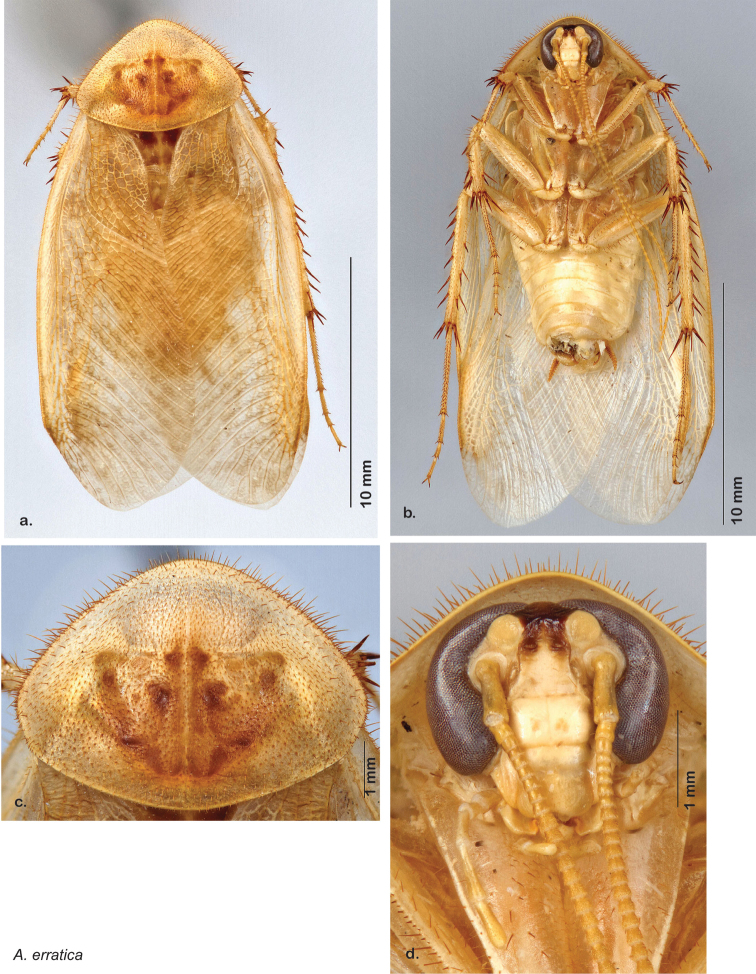
*Arenivaga erratica*, **a** dorsal habitus **b** ventral habitus **c** pronotum **d** head.

*Head*. Two ocelli large, ovoid and protruding (0.40 × 0.30 mm); vertex medium brown, with small ridges between apices of eyes extending on to ocellar tubercles; interocellar space slightly concave, medium brown with light brown medial line; two oval indentations laterally at base of interocellar space. Frons waxy white, flat; bound on either side by ridges extending from inner apex of ocelli outwards to lateral edges of clypeus; scattered long setae on frons and ridges. Anterior portion of frons waxy white, bulbous; clypeal suture demarcates waxy white anteclypeus. See Fig. 48d.

*Pronotum*. Pronotum translucent waxy beige; variable length orange-brown setae along anterior margin; dorsal surface of pronotum covered with short orange-brown setae that are denser and longer anteriorly and laterally; pronotal pattern medium orange-brown “panther face”, but runs through every shade to dark brown in other specimens; with no aura and little discernible detail, though considerable detail seen in some specimens. See Fig. 48c.

*Body*. Wing brace present. Legs and body light orange-brown; subgenital plate asymmetrical with posterior edge emarginated, rounded apices. See Fig. 48b.

*Forewings*. Wings extended beyond abdominal apex (up to ~35% of total wing length); color highly variable from light beige, to light brown, to light orange-brown, to medium brown; usually blotchy; surface semi-transparent and matte, or with faint sheen on many specimens. See Fig. 48a.

**Figure 49.**
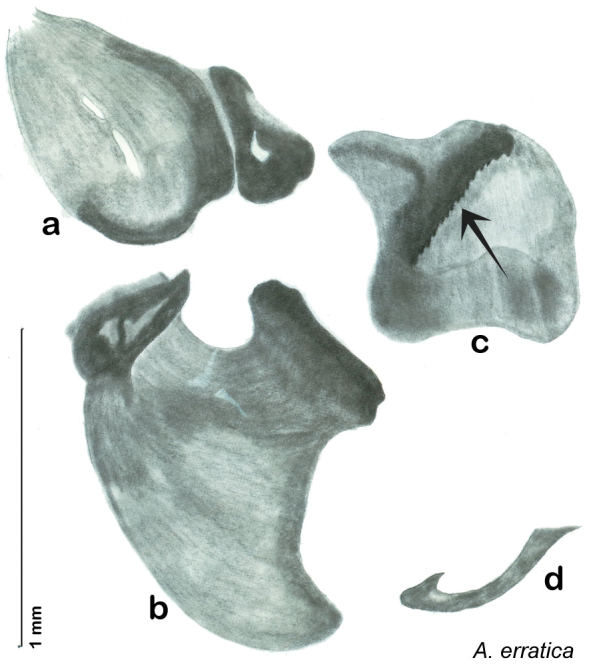
*Arenivaga erratica*, genitalia: **a** right dorsal phallomere **b** right ventral phallomere **c** small central sclerite **d** genital hook. Arrow(s) indicate diagnostic characters (see text).

*Genitalia*. Right dorsal phallomere composed of bulbous lightly sclerotized hook-shaped lobe, articulated with right ventral phallomere on lateral side; central field lightly sclerotized; medial margin sclerotized, shagreened, often with one or two small dorsally projecting teeth; medial margin extends beyond rest of phallomere at each end. Small central sclerite punctate, with sclerotized shagreened ridge, toothed along edge, along anterolateral side. Right ventral phallomere extends from articulation into bulbous love that narrows anteriorly; after narrow gap, rounded, punctate arm extending to depth of rest of phallomere. Genital hook with pointed head and moderate hook; arm moderate with smooth curve. See Fig. 49.

**Figure 50.**
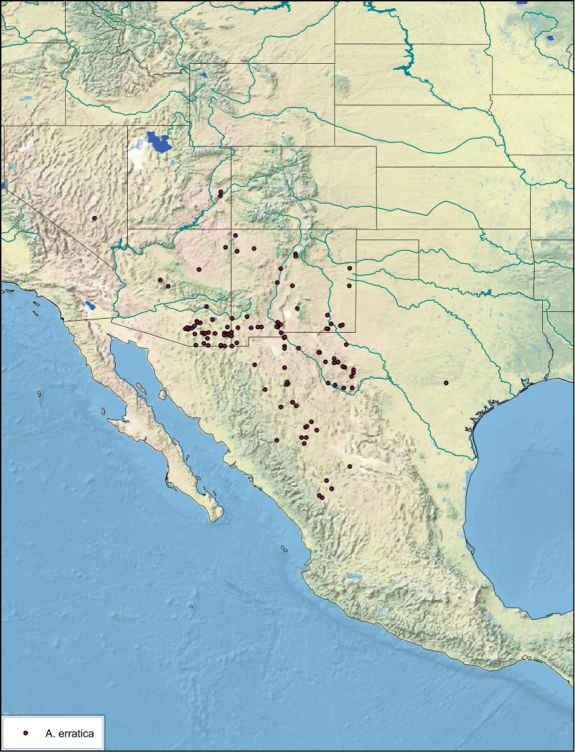
*Arenivaga erratica*, distribution.

##### Habitat and natural history.

All life history elements remain unobserved.

#### Arenivaga estelleae sp. n.

http://zoobank.org/0AF54DC2-9A96-494E-B967-976A5AAFD8ED

http://species-id.net/wiki/Arenivaga_estelleae

Figures 51 – 53

##### Type locality.

USA, California, San Diego Co., north of Fallbrook.

##### Material examined.

Holotype: ♂ in LACM labeled “July 27, 1957, 2.3 mi N. Fallbrook, San Diego Co., Calif., Lionel A Stange “HOLOTYPE *Arenivaga estelleae* Hopkins, 2012” [red label with black border].

Paratypes (41): USA: CA, San Diego Co., NAS Miramar 4, 11/13/1996, N Bloomfield, blacklite (1, SDMC); CA, San Diego Co., MCAS Miramar, 9/18/1998, N Bloomfield, blacklite (1, SDMC); CA, San Diego Co., Mission Gorge Dam, 7/26/1976, illegible (1, SDMC); CA, San Diego Co., San Diego, 6/10/1953, J Powell, at light (1, EMEC); CA, San Diego Co., Lake Hodges, 8/24/1976, DK Faulkner (1, SDMC); CA, San Diego Co., N San Vicente Res, 8/19/1976, DK Faulkner (1, SDMC); CA, San Diego Co., 2.3 mi N of Fallbrook, 7/27/1957, LA Stange (1, LACM); CA, San Diego Co., Boulevard Manzanita, 10/10/1980, R Messner (1, SDMC); CA, San Diego Co., Boulevard Manzanita, 10/5/1979, R Messner (1, SDMC); CA, San Diego Co., Boulevard Manzanita, 8/7/1979, R Messner (1, SDMC); CA, San Diego Co., Boulevard Manzanita, 7/12/1979, R Messner (4, SDMC); CA, San Diego Co., Boulevard Manzanita, 6/3/1980, R Messner (1, SDMC); CA, San Diego Co., Boulevard Manzanita, 5/28/1980, R Messner (1, SDMC); CA, San Diego Co., Rancho Santa Fe, 8/25/1958, JR Northern (1, LACM); CA, San Diego Co., San Diego, 6/19/1974, Munzenmaier & Patten, Japanese beetle trap, 74524-38, *Arenivaga* sp. Det. AR Hardy 1974 (1, CSCA); CA, San Diego Co., Boulevard Manzanita, 7/29/1979, R Messner (1, SDMC); CA, San Diego Co., Alpine, 7/18/1990, J Mitchell (1, SDMC); CA, San Diego Co.,, NAS Miramar 2, 5/19/1997, N Bloomfield, blacklite (1, USNM); CA, San Diego Co., San Diego, 7/17/1912, FE Maisdell (1, CAS); MEXICO: BC, 4 mi SW of La Zapopita, Valle de Trinidad, 4/16/1961, FS Truxal (4, LACM); BC, 11 mi E of Ojos Negros on road to Laguna Hanson, 8/9/1988, 1160 m, Weissman & Lightfoot, Stop #88-85 (1, CAS); BC, 8 mi E of Tecate, 7/6/1984, Brown & Tocco, green dot (1, CAS); BC, Ensenada, 8/30/1952 (2, USNM); BC, turnoff Hwy 1 to Motel Durado, 3 km S of Ensenada, 7/18/1977, D Weissman, coastal sand dunes (1, CAS); BC, 17 mi S of Ensenada, 6/14/1938, Michelbacher & Ross (1, CAS); BC, Hwy 2, 5.7 km W of El Condor at KM 88.7, 8/19/1995, 1210 m, Weissman & Lightfoot, Stop #95-65 (1, CAS); BC, 13 mi SW of La Zapopita, 6/14/1963, EL Sleeper, blacklite (2, CAS); BC, 17 mi S of Ensenada, 6/14/1938, Michelbacher & Ross, photo.spec. (1, LACM); BC, Km 56 on road to Sierra San Pedro Martir Park off Hwy 1, 8/4/1981, 900 m, Lightfoot & Weissman, #81-64 (1, CAS). All paratypes labeled “Paratype *Arenivaga estelleae* Hopkins 2012” [blue label with black border].

##### Etymology.

The name is a noun in the genitive case. This species is named for my grandmother, Estelle Beaumont, who taught me so much including how to be firm yet kind; also for the Latin stella, meaning star, because it is a lovely little roach.

##### Distribution.

This species is found in far southwestern California and northwestern Baja California Norte, Mexico. See Fig. 53.

##### Diagnosis.

*Arenivaga estelleae* is smaller and browner than average for *Arenivaga* and could be confused with the parapatric species *Arenivaga gaiophanes* (in its external appearance) and *Arenivaga paradoxa* (in its genitalia). These three species are most likely closely related. *Arenivaga estelleae* may be distinguished by the broad central field on the right dorsal phallomere and very wide gap on the right ventral phallomere. See Figs 52 and 124.

##### Description.

**Male.** *Measurements*. Holotype TL = 16.5 mm, GW = 8.9 mm, PW = 4.93 mm, PL = 3.31 mm, TL/GW = 1.85, PL/PW = 0.67. EW = 0.45 mm; OW = 0.45 mm. Among paratypes range of TL 15.1–20.6 mm; range of GW 7.2–9.8 mm; range of PW 4.57–5.91 mm; range of PL 3.31–4.25 mm.

**Figure 51.**
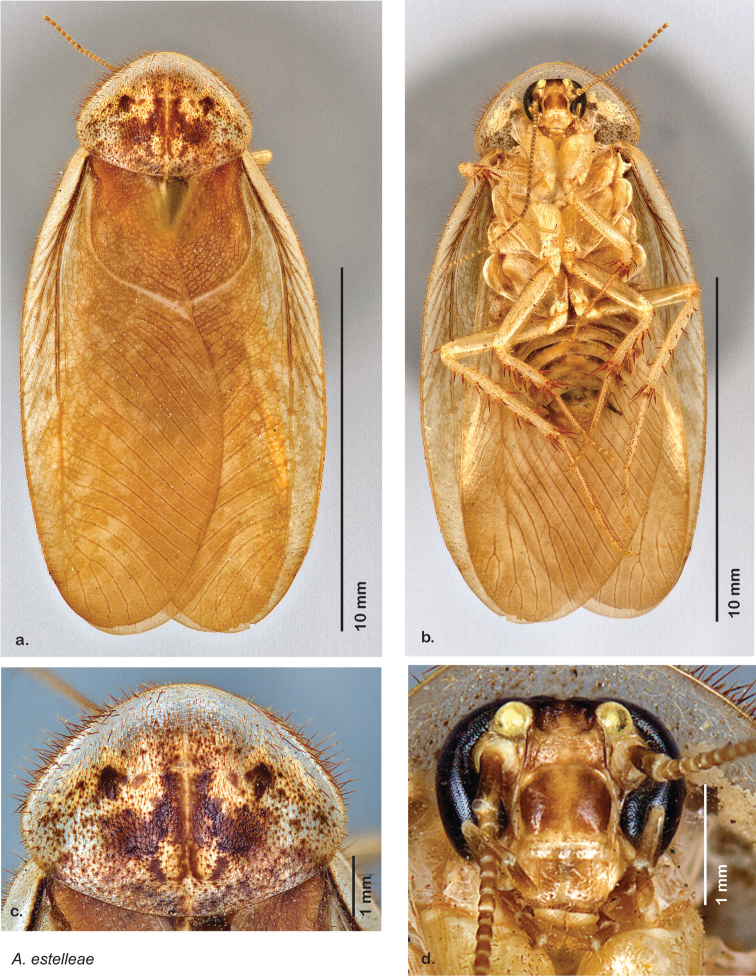
*Arenivaga estelleae*, **a** dorsal habitus **b** ventral habitus **c** pronotum **d** head.

*Head*. Two ocelli large, ovoid and protruding (0.3 × 0.2 mm); vertex flat and medium brown with small ridges in rays around upper apex of eyes and extending onto ocellar tubercles; interocellar space concave with fine horizontal corrugations; medium brown laterally shading to light brown in concavity; deep vertical indentations medial to ocelli. Frons light brown, concave; anterior portion of frons bulbous, light brown medially shading darker laterally; pale beige anteclypeus with one horizontal corrugation. See Fig. 51d.

*Pronotum*. Pronotum translucent, waxy beige; dorsal surface of pronotum with short fine brown setae centrally and posteriorly grading to longer, thicker setae laterally and anteriorly; pronotal pattern brown “panther face”; brown maculations scattered across posterior 80% of dorsal surface of pronotum; no aura. See Fig. 51c.

*Body*. Wing brace present. Two tarsal claws present. Legs beige, body light brown with dark brown markings on lateral anterior portion of each sternite; subgenital plate light brown; strongly asymmetrical with rounded apices. See Fig. 51b.

*Forewings*. Wings extended well beyond abdominal apex (~40% of wing length); color light brown to dark medium brown depending on specimen, faintly blotchy; surface matte and opaque. See Fig. 51a.

**Figure 52.**
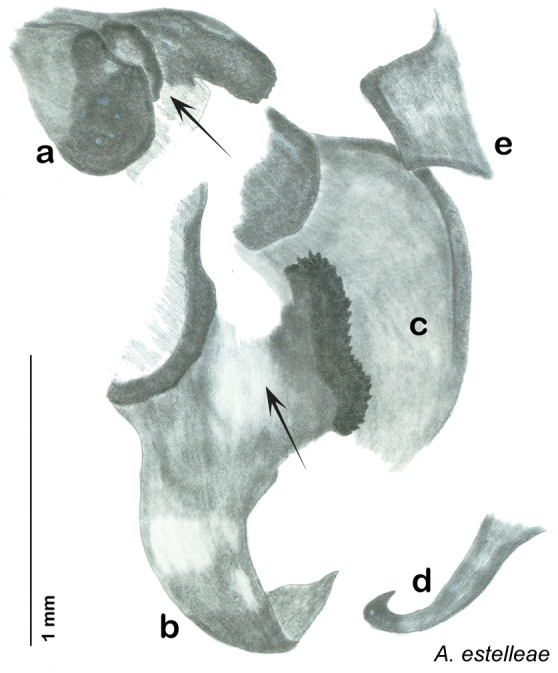
*Arenivaga estelleae*, genitalia: **a** right dorsal phallomere **b** right ventral phallomere **c** small central sclerite **d** genital hook **e** left phallomere. Arrow(s) indicate diagnostic characters (see text).

*Genitalia*. Right dorsal phallomere composed of bulbous lightly sclerotized hook-shaped lobe, articulated with right ventral phallomere on lateral side; central field sclerotized; medial margin with slight concavity, shagreened with toothed margin; anterior and posterior margins deeply emarginate, posterior margin curving back out to several teeth; interior of lateral articulation setose; broad rolled sclerotized lip projecting posteriorly from lateral articulation to several teeth. Small central sclerite broad, flat and finely punctate with posteriorly projecting, sclerotized lip at anterior end; right ventral phallomere extends from articulation to form rounded shagreened structure with acute edge; attached anteriorly is mildly dorsally projecting flanged arm, shagreened with roughly toothed edge. Folded anterior portion of left phallomere dramatically modified into heavily setose, medially projecting, scoop shape. Genital hook with short extension to pointed head with average hook; arm robust. See Fig. 52.

**Figure 53.**
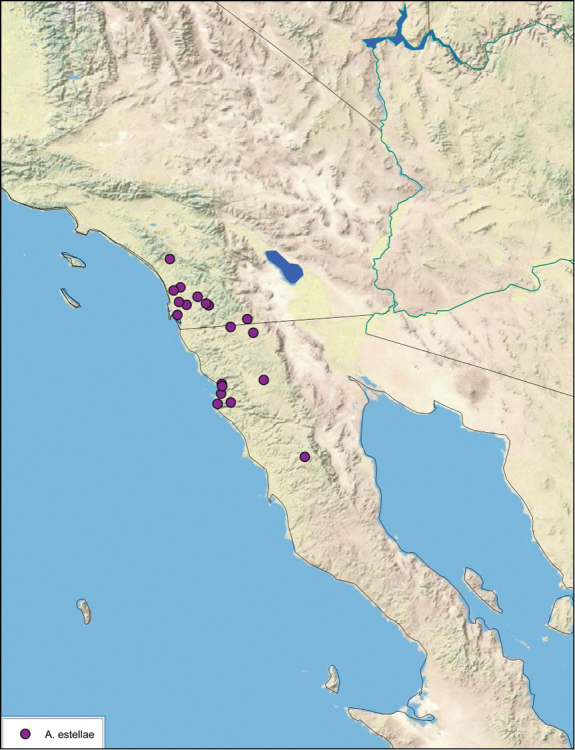
*Arenivaga estelleae*, distribution.

##### Habitat and natural history.

All life history elements remain unobserved.

#### Arenivaga floridensis

Caudell

http://species-id.net/wiki/Arenivaga_floridensis

Figures 54 – 56

Arenivaga floridensis Caudell 1918, Proceedings of the Entomological Society of Washington, 20(7), pp. 156–157.
Arenivaga floridensis Caudell 1920, Hebard, Transactions of the American Entomological Society, 46(2), pp. 197–217.

##### Material examined

**(44).** USA: FL, Polk Co., Auburndale, Lake Blue Scrub, 4/8/2009, P. Skelley, (1, FSCA); FL, Polk Co., Tiger Creek Preserve, 2.5 mi. SE Babson Park, 5/18-19/2006, Skelley and Almquist, sand sifts (2, FSCA); FL, Marion Co., Juniper Spring SP, 5/19/1990, P. Skelley, light (1, FSCA); FL, Levy Co.,. 25 mi. SW of Alachua Co. line, 6/28/1992, L.R.Davis Jr., (5, FSCA); FL, Volusia Co., in malt trap (1, FSCA); FL, Hillsborough Co., USF campus, 6/29/1972, (1, FSCA); FL, Putnam Co., Interlachen, 6/26/1992, L.R.Davis Jr., leg removed for DNA analysis T.Lamb X-2006 (1, FSCA); FL, Hillsborough Co., Vestavia Apts., 4/4/1977, Boyd, Blattidae (1, FSCA); FL, Alachua Co., 6/2/1986, E.G.Farnworth, (1, FSCA); FL, Levy Co.,. 25 mi. SW of Alachua Co. line on Rt. 24, 7/17/1993, L.R.Davis Jr., (1, UCRC); FL, Hillsborough Co., USF, Tampa, 6/6/1972, (1, FSCA); FL, Hillsborough Co., N. Tampa, 7/29/1972, (1, FSCA); FL, Levy Co., 3.9 mi. SW Archer, 4/1-7/1991, P. Skelley, pawpaw bloom (1, FSCA); FL, Highlands Co., Archbold Biol. Station, 3/22/1969, L.L.Pechuman, (1, CUIC); FL, Highlands Co., Archbold Biol. Station, 6/3/1966, R.G.Beard, (1, CUIC); FL, Highlands Co., Archbold Biol. Station, Lake Placid, 3/27-30/1959, J.G.Francelemont, (3, CUIC); FL, Highlands Co., Archbold Biol. Station, 3/27/1967, R.G.Beard, at 15 watt UV light (1, CUIC); FL, Highlands Co., Lake Placid, 7/13/1948, B.W.Crowder, (5, SEMC); FL, Lake Co., Ocala NF, 3/16/1956, Howden and Howell, (1, FSCA); FL, Highlands Co., 3/31/1961, J.C.Hanlon, in blacklight trap (1, FSCA); FL, Highlands Co., Archbold Biol. Station, 8 mi. S Lake Placid, 4/3/1974, G.C.Eickwort, (1, CUIC); FL, Tarpon Springs, 7/18/1983, Konger, (1, FSCA); FL, Orange Co., Orlando, 5/31/1924, F.W.Walker, (1, ANSP); FL, Co., Lakeland, 5/4/1912, W.T.Davis, genitalia figured H1920 (1, ANSP); FL, Highlands Co., Archbold Biol. Station, 2/14/1951, floridensis (1, USNM); FL, Orange Co., Orlando, 5/31/1924, F.W.Walker, (1, USNM); FL, Polk Co., Lake Streaty, 8/10/1938, T32S,R27E,Sec25 110/111, Hubbell and Friauf, (2, USNM); FL, Highlands Co., Archbold Biol. Station, Lake Placid, 2/2/1959, S.W.Frost, (1, USNM); FL, Clay Co., Gold Head Branch SP, 3/31/1956, T.H.Hubbell, (1, USUSNM); FL, Clay Co., Gold Head Branch SP, 5/6/1954, L.H.Krombein, (1, USNM); FL, Orange Co., Orlando, 5/10/1924, F.W.Walker, (1, UMMZ); FL, Levy Co.,. 2 mi SW Alachua County Line, Rt. 24, 6/6/1977, LR Davis, Jr., (1, FSCA). Determiner label *Arenivaga floridensis* Hopkins 2011” [white label with black border].

##### Distribution.

This species is found in central Florida. See Figure 56.

##### Diagnosis.

*Arenivaga floridensis* may be diagnosed by its locality or by the medially projecting knob by the posterior end of the point of articulation between the two right phallomeres. See Fig. 55.

##### Description.

**Male. NB: Holotype is half spread therefore GW is estimated.** *Measurements*. Holotype TL = 17.4 mm, GW = 9.0 mm, PW = 6.71 mm, PL = 4.26 mm, TL/GW = 1.93, PL/PW = 0.63. EW = 0.50 mm; OW = 0.55 mm. Among paratypes range of TL 13.8–20.7 mm; range of GW 7.7–10.7 mm; range of PW 5.43–7.24 mm; range of PL 3.82–4.69 mm.

**Figure 54.**
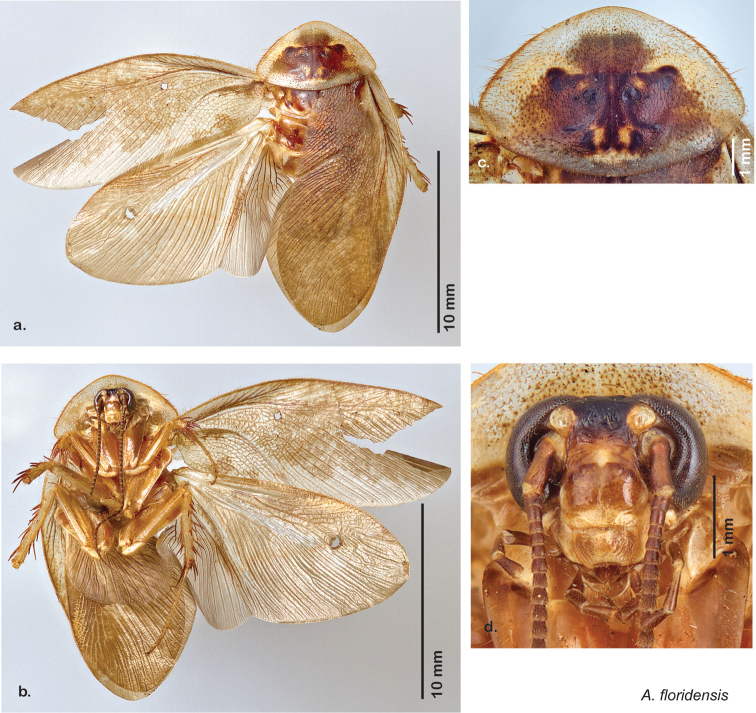
*Arenivaga floridensis*, **a** dorsal habitus **b** ventral habitus **c** pronotum **d** head.

*Head*. Two ocelli large, ovoid and protruding (0.40 × 0.30 mm); vertex dark brown, with small ridges between apices of eyes and scattered short setae; interocellar space concave, dark brown. Frons medium brown fading posteriorly to light brown, tectiform, concave; bound on either side by ridges extending from inner apex of ocelli outwards to lateral edges of clypeus; scattered long setae on ridges. Anterior portion of frons light brown with medium brown maculations, bulbous; clypeal suture demarcates light brown anteclypeus. See Fig. 54d.

*Pronotum*. Pronotum translucent waxy beige. Variable length orange-brown setae along anterior margin; dorsal surface of pronotum covered with short orange-brown setae; pronotal pattern “panther face” dark brown, with small to extensive aura depending on specimen; discernibility of detail variable but generally poor. See Fig. 54c.

*Body*. Wing brace present. Legs and body medium orange-brown; subgenital plate symmetrical with posterior edge emarginated, rounded apices. See Fig. 54b.

*Forewings*. Wings extended beyond abdominal apex (up to ~30% of total wing length); color uniform dark brown depending on specimen; surface opaque and matte, or with faint sheen on many specimens. See Fig. 54a.

**Figure 55.**
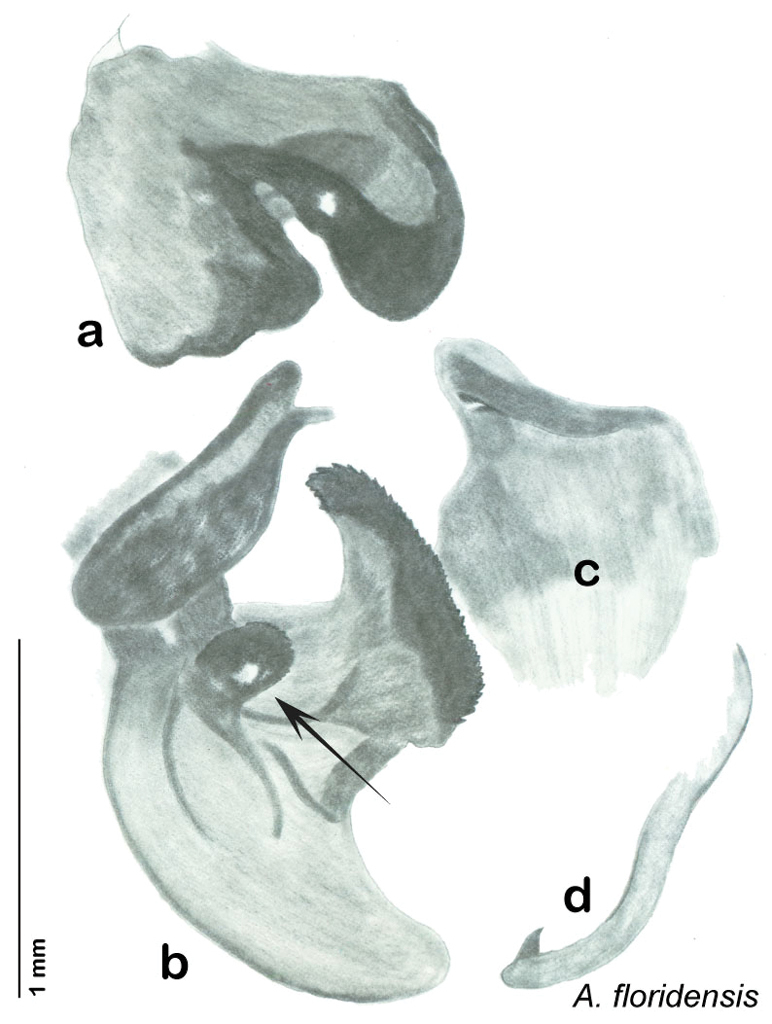
*Arenivaga floridensis*, genitalia: **a** right dorsal phallomere **b** right ventral phallomere **c** small central sclerite **d** genital hook. Arrow(s) indicate diagnostic characters (see text).

*Genitalia*. Right dorsal phallomere composed of bulbous lightly sclerotized hook-shaped lobe, articulated with right ventral phallomere on lateral side; central field lightly sclerotized; medial margin sclerotized, heavily toothed, curving ventrally at anterior end. Small central sclerite finely punctate, concave, with shagreened bulge along ventral edge. Right ventral phallomere extends from articulation into smooth flattened posteriorly projecting lobe with anteriorly projecting spine on medial edge; after ridge and moderate gap, rounded concave smooth flange. Folded anterior portion of left phallomere wide, setose, otherwise unmodified. Genital hook with pointed head with dimple along extension to a moderate hook. See Fig. 55.

**Figure 56.**
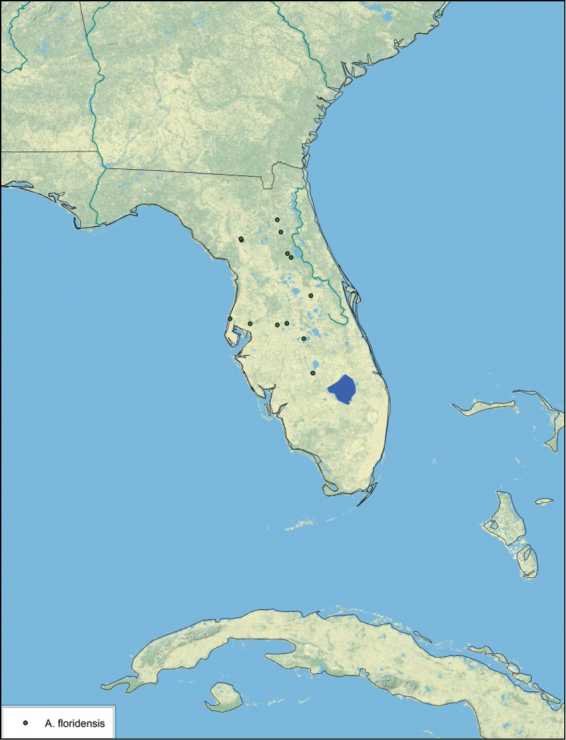
*Arenivaga floridensis*, distribution.

##### Habitat and natural history.

All life history elements remain unobserved.

#### Arenivaga florilega sp. n.

http://zoobank.org/6686685E-AA73-42D2-9D9B-F974A5DB8016

http://species-id.net/wiki/Arenivaga_florilega

Figures 57 – 59

##### Type locality.

MEXICO, Hidalgo, Zimapan.

##### Material examined.

Holotype: ♂ in EMEC labeled “Zimapan Hdgo. Mex. VI-11-14-51, on fls. of Eysenhardtia polystachya (Ort.), P.D.Hurd Collector” “HOLOTYPE *Arenivaga florilega* Hopkins, 2012” [red label with black border].

Paratypes: None at this time.

##### Etymology.

The name is an adjective in the nominative singular. This species is named from the Latin meaning flower gathering because the only known specimen was collected off the flowers of *Eysenhardtia polystachya*.

##### Distribution.

This species is known only from the type locality. See Fig. 59.

##### Diagnosis.

*Arenivaga florilega* may be confused with *Arenivaga galeana* but may be distinguished by the almost complete lack of sculpturing or sclerotization on the right dorsal phallomere, the posteriorly projecting lobe on the right ventral phallomere, and a round curved genital hook. See Figs 58 and 64.

##### Description.

**Male.** *Measurements*. Holotype TL = 19.7 mm, GW = 9.5 mm, PW = 5.37 mm, PL = 3.98 mm, TL/GW = 2.07, PL/PW = 0.74. EW = 0.15 mm; OW = 0.40 mm.

**Figure 57.**
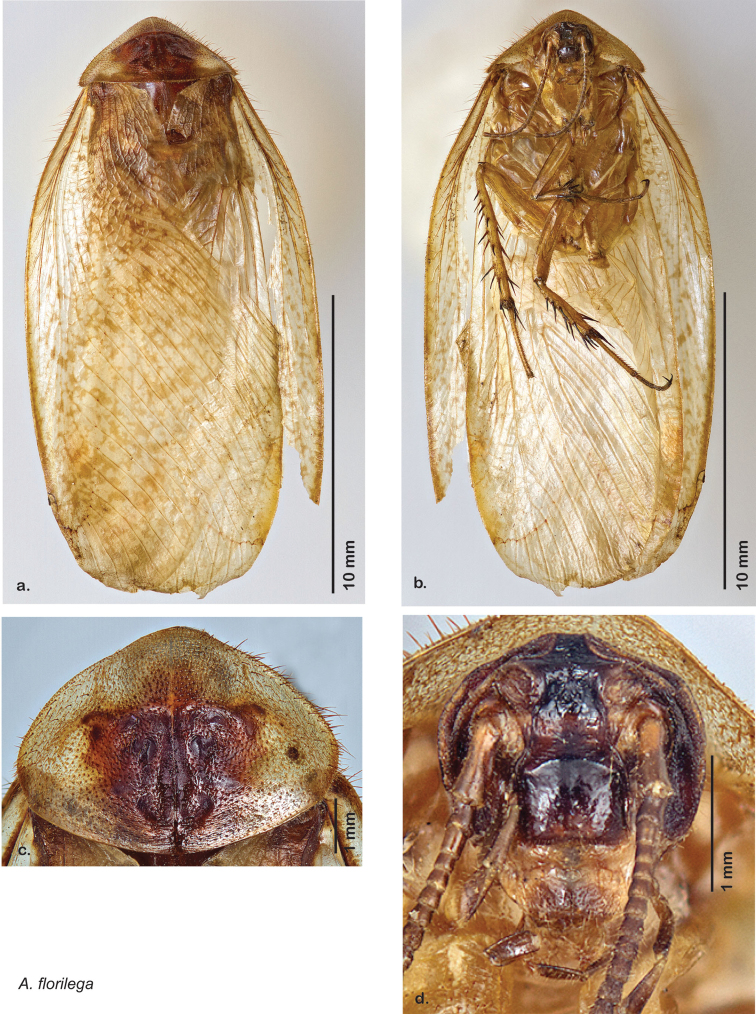
*Arenivaga florilega*, **a** dorsal habitus **b** ventral habitus **c** pronotum **d** head.

*Head*. Two ocelli large, ovoid and protruding (0.40 × 0.25 mm); vertex dark brown, with small ridges between apices of eyes and extending onto ocellar tubercles; interocellar space concave, dark brown. Frons dark medium brown; posterior concave; anterior portion of frons bulbous but much less so than in most species, dark medium brown; light brown anteclypeus. See Fig. 57d.

*Pronotum*. Pronotum translucent waxy light brown; dorsal surface of pronotum with dense orange-brown setae that are longer and thicker laterally; pronotal pattern dark orange-brown “panther face”, impressed; slight aura. See Fig. 57c.

*Body*. Wing brace absent. Two tarsal claws present. Legs and body medium brown; subgenital plate orange-brown; strongly asymmetrical with posterior edge only slightly emarginated and rounded apices. See Fig. 57b.

*Forewings*. Wings extended beyond abdominal apex; light brown with sparse medium brown blotches; surface translucent with mild sheen. See Fig. 57a.

**Figure 58.**
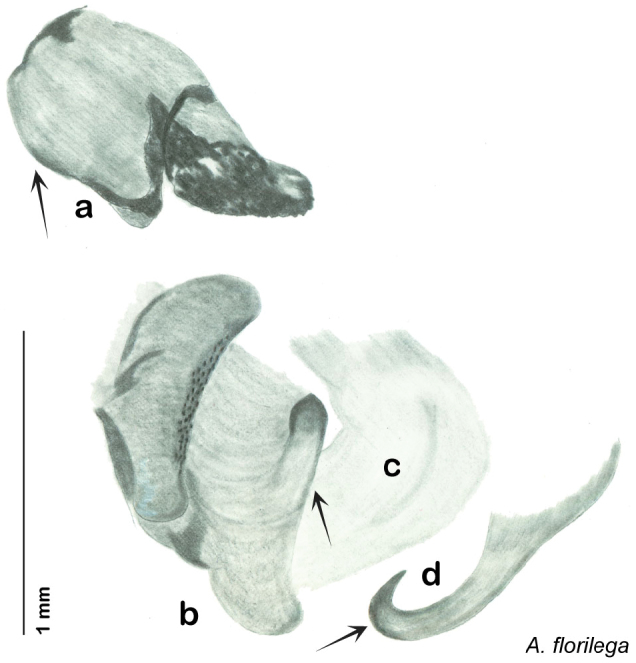
*Arenivaga florilega*, genitalia: a) right dorsal phallomere **b** right ventral phallomere **c** small central sclerite **d** genital hook. Arrow(s) indicate diagnostic characters (see text).

*Genitalia*. Right dorsal phallomere composed of lightly sclerotized, narrow, bulbous lobe, articulated with right ventral phallomere on lateral side; central field lightly sclerotized, cupped; with narrow medial edge more sclerotized, punctate, ending anteriorly in small shagreened flange. Small central sclerite lightly sclerotized, finely punctate, concave with two punctate lobes wrapping anteriorly around shagreened flange of right dorsal phallomere, posterior end connecting with dorsal side of right dorsal phallomere. Articulation between right phallomeres deep, concave and setose, with shagreened border adjacent to dorsal phallomere and smooth border adjacent to ventral phallomere. Right ventral phallomere consists of large punctate flattened medially projecting lobe; becoming wider and more sclerotized anteriorly; anteriorly narrow gap followed by wide shagreened flange. Folded anterior portion of left phallomere wide, setose, otherwise unmodified. Genital hook widely curved to sharp point; arm robust and straight. See Fig. 58.

**Figure 59.**
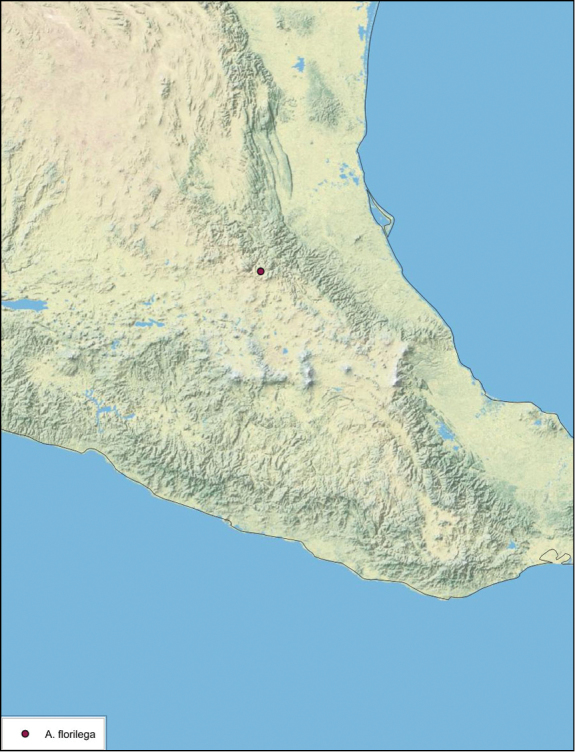
*Arenivaga florilega*, distribution.

##### Habitat and natural history.

All life history elements remain unobserved.

#### Arenivaga gaiophanes sp. n.

http://zoobank.org/6D0A707A-46BA-4AE1-B5EB-98BA4F4A2F56

http://species-id.net/wiki/Arenivaga_gaiophanes

Figures 60 – 62

##### Type locality.

USA, California, Riverside Co., Ranchos los Ninos de Luz.

##### Material examined.

Holotype: ♂ in LACM labeled “Riverside Co., Calif. Rancho Los Ninos De Luz, Murrieta Rd., 10 Oct. 71, 600’, J.A.Honey” “HOLOTYPE *Arenivaga gaiophanes* Hopkins, 2012” [red label with black border].

Paratypes (25): USA: CA, Riverside Co., Hemet, San Jacinto Rov., 6/14/2003, 33.45.46N 116.53.18W 526 m, GR Ballmer, at MV light, PhotoEloous 327:36,328:1-3 (1, UCRC); CA, Orange Co., Lower San Juan Cpgd., 7/27/1982, CW Melton (2, UCMC); CA, Riverside Co., Bundy Canyon, 9 mi S of Perris, 7/27/1978, 1660 ft., RJ Ford (2, LACM); CA, San Bernardino, Lake Arrowhead, 7/12/1964, EI Schlinger, at white light (1, UCRC); CA, Fallbrook, 7/8/1972, LD Anderson (1, UCRC); CA, Riverside Co., Rancho Los Ninos de Luz, Murrieta Rd., 10/10/1971, 600 ft., JA Honey (2, LACM); CA, Riverside Co., Menifee Valley, 7/4/1976, 33.39.19N 117.12 45W, SI & SL Frommer, at white light (1, UCRC); CA, Riverside Co., Menifee Valley, hills on W end, 8/6/20003, 3.39N, 117.13W, 1800 ft. JD Pinto, at light (1, UCRC); CA, Riverside Co., Riverside, 4/17/1972, BJ Taylor (1, UCRC); CA, Riverside Co., Menifee Valley, 8/20/1976, 33.39.19N 117.12 45W, CL Lacey, genitalia missing (1, UCRC); CA, Riverside Co., Menifee Valley, hills on W end, 6/29/1984, 33.39N, 117.13W, 1800 ft., JD Pinto (1, UCRC); CA, Riverside Co., Menifee Valley, hills on W end, 6/16/1978, 33.39N, 117.13W, 1899 ft., JD Pinto (1, UCRC); CA, Orange Co., San Juan Creek, 9/27/1954, RJ Ford (3, LACM); CA, Riverside Co., Menifee Valley, hills on W end, 6/1/2001, 33.39N, 117.13W, 1800 ft., JD Pinto (1, UCRC); CA, San Diego Co., Wildcat Canyon near Lakeside, 8/3-28/1962, SC Williams, scorpion pit trap in 1961 chaparral burn area (1, ASUT); CA, Riverside Co., Tenajas Ranger Station, 7/29/1967, JA Honey (2, LACM); CA, Riverside Co., Tenajas Ranger Station, 8/19/1967, JA Honey (1, LACM), CA, Riverside Co., Pinyon Flat Campground, 14 mi SW of Palm Desert on SR74, 7/3-4/2008, JA Cole (1, ANSP), CA, Riverside Co., Pinyon Flat Campground, 14 mi SW of Palm Desert on SR74, 6/28-29/2003, JA Cole (1, ANSP). All paratypes labeled “Paratype *Arenivaga gaiophanes* Hopkins 2012” [blue label with black border].

##### Etymology.

The name is an adjective in the nominative singular. This species is named gaiophanes for its beautiful uniform earth tone coloration, from Greek meaning “earth-colored”.

##### Distribution.

This species is distributed from Lake Arrowhead in the north, to Wildcat Canyon in the south, and from Hemet in the east, to San Juan Creek in the west. See Figure 62.

##### Diagnosis.

*Arenivaga gaiophanes* sp. n. is slightly smaller than average, warm brown in color with scattered blotches. It can be mistaken phenotypically for *Arenivaga sequoia* but the genitalia are distinct; the medial margin of the right dorsal phallomere is deeply indented in *Arenivaga gaiophanes* but not at all in *Arenivaga sequoia*. See Figs 61 and 142.

##### Description.

**Male.** *Measurements*. Holotype TL = 19.2 mm, GW = 9.2 mm, PW = 5.22 mm, PL = 3.81 mm, TL/GW = 2.09, PL/PW = 0.73. EW = 0.3 mm; OW = 0.4 mm. Among paratypes range of TL 15.6–19.2 mm; range of GW 7.5–9.2 mm; range of PW 5.02–6.00 mm; range of PL 2.97–4.24 mm.

**Figure 60.**
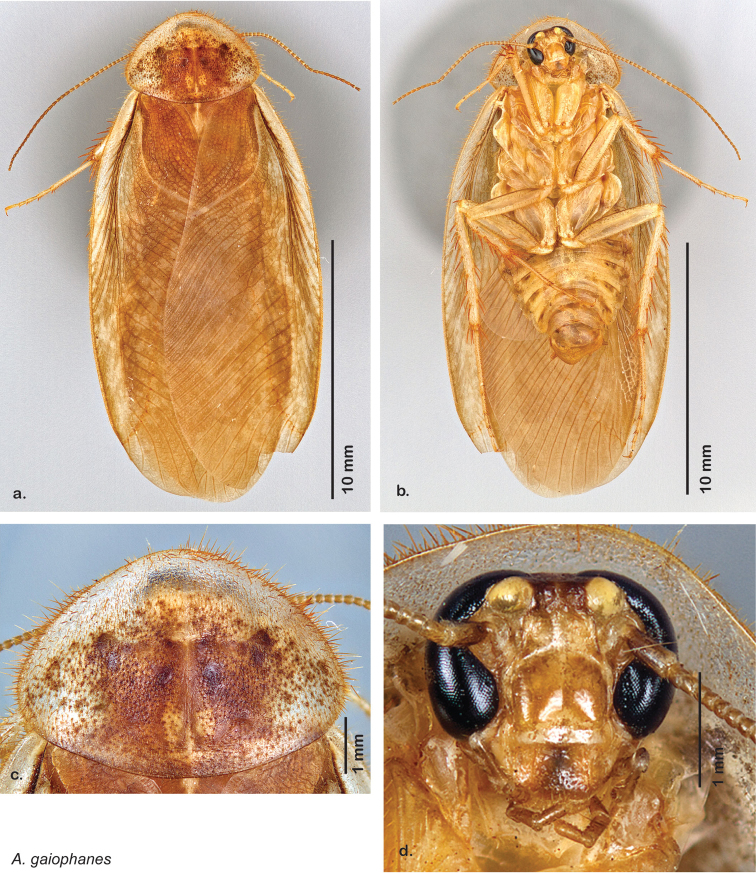
*Arenivaga gaiophanes* **a** dorsal habitus **b** ventral habitus **c** pronotum **d** head.

*Head*. Two ocelli large, ovoid and protruding (0.45 × 0.35 mm); vertex medium brown with small ridges in rays around upper apices of eyes and extending onto ocellar tubercles; interocellar space concave, rugose, medium brown. Posterior frons light brown, concave; anterior frons bulbous with slight central indentation; light brown anteclypeus. See Fig. 60d.

*Pronotum*. Pronotum translucent, waxy beige; dorsal surface of pronotum with short fine brown setae centrally and posteriorly grading to longer, thicker setae laterally and anteriorly; pronotal pattern brown “panther face”; brown maculations scattered across posterior 70% of dorsal surface of pronotum; no aura. See Fig. 60c.

*Body*. Wing brace present. Two tarsal claws present. Legs and body light orange-brown; darker maculation laterally on each sternite; subgenital plate dark orange-brown; strongly asymmetrical with rounded apices. See Fig. 60b.

*Forewings*. Wings extended well beyond abdominal apex (~40% of wing length); color uniform brown, to blotchy brown; surface matte and opaque. See Fig. 60a.

**Figure 61.**
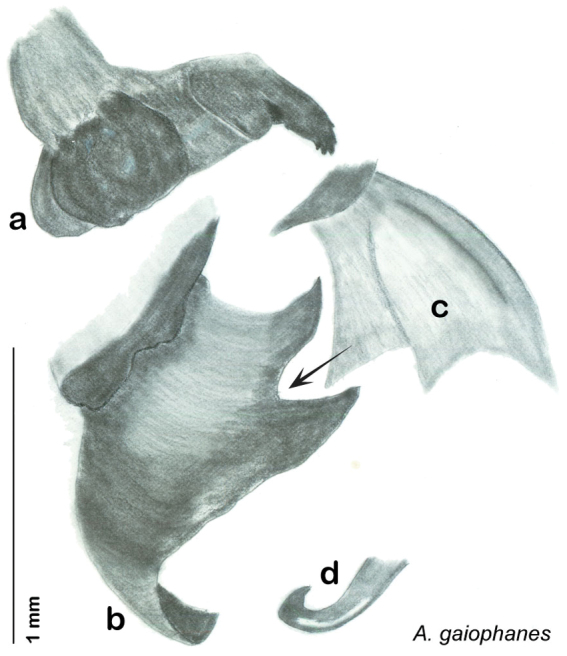
*Arenivaga gaiophanes*, genitalia: a) right dorsal phallomere **b** right ventral phallomere **c** small central sclerite **d** genital hook. Arrow(s) indicate diagnostic characters (see text).

*Genitalia*. Right dorsal phallomere composed of bulbous lightly sclerotized hook-shaped lobe, articulated with right ventral phallomere on lateral side; central field sclerotized; medial margin with deep V-shaped emargination projecting to two broad flat points; entire phallomere shagreened except hook-shaped lobe and anterior point; entire medial margin with short teeth; interior of lateral articulation setose; broad rolled sclerotized lip projecting posteriorly from lateral articulation. Small central sclerite broad, flat and finely punctate with posteriorly projecting, sclerotized lip at anterior end; right ventral phallomere extends from articulation to form rounded shagreened structure with fine corrugations; attached anteriorly is mildly dorsally projecting flanged forked arm, shagreened with roughly toothed edge. Folded anterior portion of left phallomere setose, otherwise unmodified. Genital hook with short extension to pointed head with short hook; arm robust. See Fig. 61.

**Figure 62.**
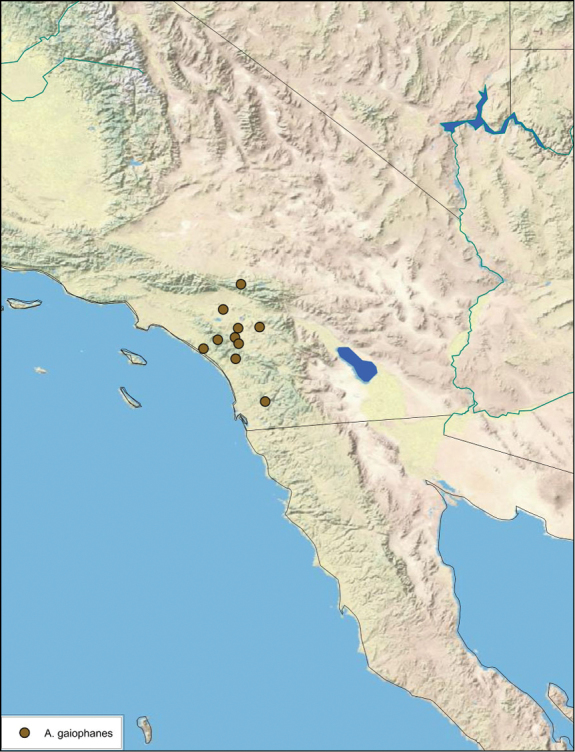
*Arenivaga gaiophanes*, distribution.

##### Habitat and natural history.

This species occurs in hilly or mountainous habitat. All other life history elements remain unobserved.

#### Arenivaga galeana sp. n.

http://zoobank.org/6F2CBB4C-2342-42B3-9F5B-2629FF170642

http://species-id.net/wiki/Arenivaga_galeana

Figures 63 – 65

##### Type locality.

MEXICO, Nuevo Leon, 3 mi E Galeana.

##### Material examined.

Holotype: ♂ in USNM labeled “MEXICO: 3 mi. E. Galeana, N.L. 5000’, Aug. 7–9, 1963, Duckworth & Davis” “HOLOTYPE *Arenivaga galeana* Hopkins, 2012” [red label with black border].

Paratypes (11): MEXICO: Nuevo Leon, 6.4 km W Iturbide, 7/16/1979, 24.44N, 99.56W, 1800 m, DC Darling (1, CUIC); Nuevo Leon, 3 mi. E of Galeana, 8/7-9/1963, 5000 ft., Duckworth & Davis (8, USNM); El Salto Falls 26 mi. W of Antiguo Morelos, Tamps., 7/11-14/1963, 2000 ft., Duckworth & Davis (2, [one missing label] USNM). All paratypes labeled “Paratype *Arenivaga galeana* Hopkins 2012” [blue label with black border].

##### Etymology.

The name is a noun in the genive case. This species is named for the Mexican town near where the majority of specimens originate, Galeana.

##### Distribution.

This species occurs in the Sierra Madre Oriental mountains of eastern Mexico. See Fig. 65.

##### Diagnosis.

*Arenivaga galeana* may be confused with *Arenivaga florilega* but may be distinguished by the right ventral phallomere which does not have a flattened, posteriorly projecting lobe as it does in *Arenivaga florilega*. Also the genital hook is the typical angular sort, not a sweeping curve as in *Arenivaga florilega*. See Figs 64 and 58.

##### Description.

**Male.** *Measurements*. Holotype TL = 22.3 mm, GW = 9.8 mm, PW = 5.84 mm, PL = 3.79 mm, TL/GW = 2.28, PL/PW = 0.65. EW = 0.25 mm; OW = 0.40 mm. Among paratypes range of TL 19.6–23.5 mm; range of GW 8.4–9.8 mm; range of PW 5.38–6.22 mm; range of PL 3.73–4.33 mm.

**Figure 63.**
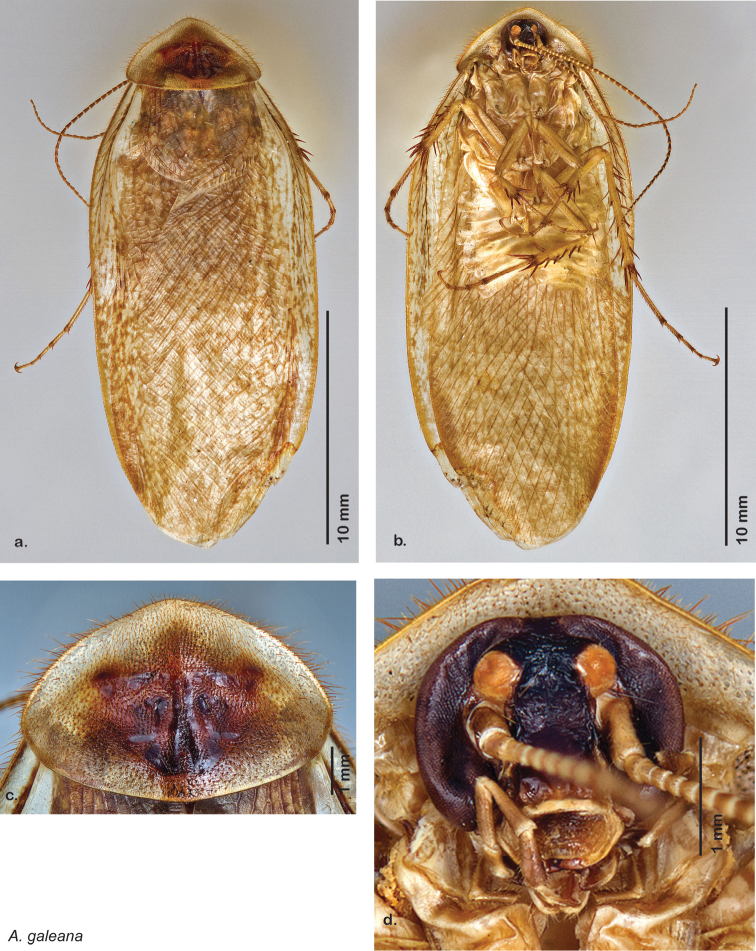
*Arenivaga galeana* **a** dorsal habitus **b** ventral habitus **c** pronotum **d** head.

*Head*. Two ocelli large, ovoid and protruding (0.50 × 0.30 mm); vertex dark brown, with small ridges between apices of eyes and extending onto ocellar tubercles; interocellar space concave, dark brown. Frons dark brown; posterior concave; anterior portion of frons slightly bulbous, anterior margin with medial point, medium brown; light brown anteclypeus. See Fig. 63d.

*Pronotum*. Pronotum translucent waxy beige; dorsal surface of pronotum with short orange-brown setae that are longer and thicker laterally; pronotal pattern dark orange-brown “panther face”, impressed, with medium orange-brown aura. See Fig. 63c.

*Body*. Wing brace absent. Two tarsal claws present. Legs and body light brown; subgenital plate dark orange-brown; strongly asymmetrical with rounded apices. See Fig. 63b.

*Forewings*. Wings extended beyond abdominal apex (up to 40% of total wing length); blotchy medium brown; surface translucent with mild sheen. See Fig. 63a.

**Figure 64.**
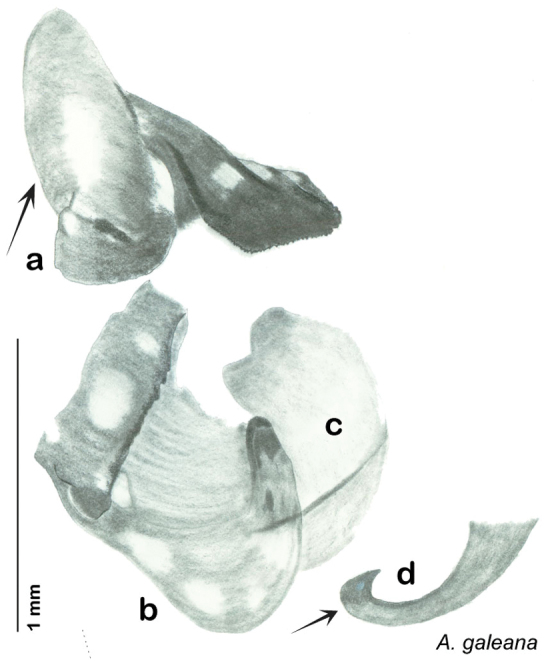
*Arenivaga galeana*, genitalia: a) right dorsal phallomere **b** right ventral phallomere **c** small central sclerite **d** genital hook. Arrow(s) indicate diagnostic characters (see text).

*Genitalia*. Right dorsal phallomere composed of lightly sclerotized, bulbous lobe, articulated with right ventral phallomere on lateral side; central field lightly sclerotized, cupped; with narrow medial edge more sclerotized, punctate, ending anteriorly in small shagreened knob. Small central sclerite lightly sclerotized, finely punctate, concave with two punctate lobes wrapping anteriorly around shagreened knob of right dorsal phallomere, posterior end connecting with dorsal side of right dorsal phallomere. Articulation between right phallomeres deep, concave and setose, with shagreened border adjacent to dorsal phallomere and faint smooth border adjacent to ventral phallomere; shagreened border coming to posteriorly projecting pointed extension. Right ventral phallomere consists of large punctate flattened medially projecting lobe with central indentation; becoming wider and more sclerotized anteriorly; anteriorly wide gap followed by wide shagreened flange with slight central concavity. Folded anterior portion of left phallomere wide, setose, enclosed at both ends with indentation at medial end, otherwise unmodified. Genital hook with short extension to pointed head and short hook; arm robust. See Fig. 64.

**Figure 65.**
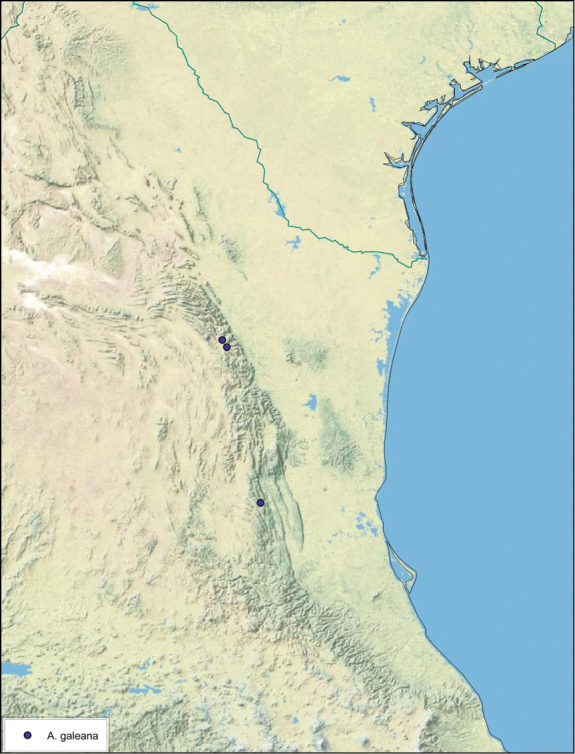
*Arenivaga galeana*, distribution.

##### Habitat and natural history.

All life history elements remain unobserved.

#### Arenivaga genitalis

Caudell

http://species-id.net/wiki/Arenivaga_genitalis

Figures 66 – 68

Arenivaga genitalis Caudell 1918, Proceedings of the Entomological Society of Washington, 20(7), pp. 154-156.
Arenivaga genitalis Caudell 1920, Hebard, Transactions of the American Entomological Society, 46(2), pp. 197-217.

##### Material examined

**(343).** USA: AZ, Pima Co., Lowell Ranger Sta., 7/6-20/1916, 32.18.5N, 110.49W, 2000’, 1:share with Clark and ANSP,1: *Arenivaga genitalis* Caud., Figured H 1920 (2, ANSP); AZ, Pinal Co., Florence, 7/17/1903, C.R.Biederman, (6, ANSP); AZ, Maricopa Co., Phoenix, _8/23/1966, R.S.Beal, at light. *Arenivaga* sp. Det. Rentz (1, NAUF); AZ, Pima Co., Redington Rd., 8/9/2003, 32.15.47N 110.39.11W, E.Riley, UV (1, TAMU); AZ, Maricopa Co., Phoenix, 6/9/1904, M.Hebard, *Arenivaga genitalis* Caudell TOPOTYPE (1, ANSP); AZ, Maricopa Co., Phoenix, 7/16-18/????, Wickham, *Arenivaga genitalis* Caudell TOPOTYPE (1, ANSP); AZ, Maricopa Co., Phoenix, 8/1/????, R.E.Kunze, *Arenivaga genitalis* Caudell TOPOTYPE (1, ANSP); AZ, Maricopa Co., Phoenix, 6/7/1904, M.Hebard, *Arenivaga genitalis* Caudell TOPOTYPE, Genitalia figured partially 1917, fully H 1920 (1, ANSP); AZ, Pima Co., Ft. Grant 60 mi. E of Tucson, (1, ANSP); AZ, Maricopa Co., Phoenix, 4/29/1902, Oslar, (1, ANSP); AZ, Maricopa Co., Tempe, 4/26/1902, Oslar, (1, ANSP); AZ, Pinal Co., Florence, 7/10/1903, C.R.Biederman, (1, ANSP); AZ, Pinal Co., Florence, 7/21/1903, C.R.Biederman, (1, ANSP); AZ, Pinal Co., Florence, 7/18/1903, C.R.Biederman, (1, ANSP); AZ, Pima Co., Sabino Canyon, Santa Catalina Mts., 5/9/1916, J.F.Tucker, (1, ANSP); AZ, Pinal Co., Florence, 7/19/1903, C.R.Biederman, (1, ANSP); AZ, Pinal Co., Florence, 6/6/1903, (1, ANSP); AZ, Pima Co., Sabino Canyon, Santa Catalina Mts., 11/2/1916, J.F.Tucker, (1, ANSP); AZ, Pinal Co., Picacho Peak SP, 8/4/1983, C.B.Barr, Collected at light (1, Essig); AZ, Pima Co., Tucson Mountain Park, 4/1/1969, R.E.Beer & party, *Arenivaga* sp. nr. *genitalis* Hebard (2, UK); AZ, Yuma Co., S Luis, 8/11/1940, E.C.Van Dyke, (1, CAS); AZ, Maricopa Co., Phoenix, 8/5/????, R.E.Kunze, Paratype No. 21879 U.S.N.M., *Arenivaga genitalis* Parat. “b” Caud. (1, USNM); AZ, Pima Co., Covered Wells, 4/12/1954, L.M.Martin, (2, LACM); AZ, Pinal Co., Florence, 5/8/1903, (1, ANSP); AZ, Pima Co., Tucson, 6/23/1963, Parker and Stange, (1, FSCA); AZ, Pinal Co., Florence, 5/10/1958, H.Tryon, (1, ASUT); AZ, Pima Co., 7/22/1927, L.D.Anderson, (1, ANSP); AZ, Pima Co., Sabino Canyon, Santa Catalina Mts., 4/26/1916, J.F.Tucker, (1, ANSP); AZ, Pima Co., Sabino Canyon, 7/31/1941, R.H.Beamer, (3, ANSP); AZ, Pima Co., 3/30/1923, O.C.Poling, (1, ANSP); AZ, Pima Co., Ajo, 3/25/1923, O.C.Poling, (1, ANSP); AZ, Pima Co., Sabino Canyon, Santa Catalina Mts., 4/20/1916, J.F.Tucker, (1, ANSP); AZ, Maricopa Co., Phoenix, 4/9/1902, Kunze, (1, ANSP); AZ, Maricopa Co., Phoenix, Oslar, (1, ANSP); AZ, Maricopa Co., Casa Grande, 4/7/1935, A.L.Melander, (1, MCZ); AZ, Maricopa Co., Paloverde, 4/21/1935, F.H.Parker, apacha (3, MCZ); AZ, Maricopa Co., Paloverde, 4/22/1935, F.H.Parker, (1, MCZ); AZ, Maricopa Co., Paloverde, 4/21/1935, F.H.Parker, (1, UCRC); AZ, Pima Co., Base of Tortolita Mts., S side, 6/14/1984, 3000’, R.S.Beal, black light (9, NAUF); AZ, Pima Co., Tucson, 7/12/1937, D.J. & J.N.Knull, 122, genitalia missing (1, OSUC); AZ, Pima Co., Sabino Canyon, Santa Catalina Mts., 7/30/1968, F.Werner, lt. (1, HEH); AZ, Pima Co., Sabino Canyon, Santa Catalina Mts., 5/21/1967, J.Hesselt, lt. (1, HEH); AZ, Gila Co., Gila River 3 mi. SW of Christmas, 6/4/1962, F.Werner, lt. trap, (3, UAIC); AZ, Pima Co., U of AZ campus, Tucson, ?/?/1917, R.Abel, Ent. 101, (1, HEH); AZ, Pima Co., Bear Canyon, Catalina Mts., 5/26/1994, M.Singer, *Arenivaga* sp. Det. B.Mathison (1, UAIC); AZ, Maricopa Co., Salt River at Bush Hwy. Bridge, 5/27/1995, B.C.&W.B.Warner & K.Miller, UV light (7, WB Warner); AZ, Maricopa Co., Coons Bluff on Salt River, 7/29/2010, 33.32.52N 111.38.39W, Bill Warner, UV light (5, WB Warner); AZ, Maricopa Co., Tortilla Flat, 7/15/1975, W.F.Chamberlain, (1, TAMU); AZ, Maricopa Co., 24 mi. N Gila Bend, Gillespie Dam, 7/16/1975, J.D.Pinto, (1, UCRC); AZ, IBP:Santa Rita Desert Site 01, 8/2/1971, Emergence trap SR08,Aregen (1, UAIC); AZ, Pima Co., Santa Rita Mts., 7/23/1978, R.H.Crandall, (1, LACM); AZ, Pima Co., Tucson, 4/22/1958, R.C.Whistler, (1, UAIC); AZ, Pima Co., Tucson, 4/12/1958, J.May, (1, UAIC); AZ, Pima Co., Tucson, 4/26/1958, R.Price, (1, UAIC); AZ, Pima Co., Tucson, 4/8/1958, J.Claney, (1, UAIC); AZ, Pima Co., Tucson, 4/20/1958, H.Nather, (1, UAIC); AZ, Pima Co., Sonoran Desert Museum, 8/5-8/1962, W.L.Nutting, lt. trap, S.Oman (2, UAIC); AZ, Pima Co., Sonoran Desert Museum, 8/1-4/1962, W.L.Nutting, lt.trap, S.Oman (1, UAIC); AZ, Pima Co., Sonoran Desert Museum, 8/9-16/1962, W.L.Nutting, lt.trap, S.Oman (1, UAIC); AZ, Pima Co., Tucson, 8/27/1938, D.J. & J.N.Knull, (1, OSUC); AZ, Pima Co., Tucson, 6/23/1939, D.J. & J.N.Knull, (2, OSUC); AZ, Pima Co., Tucson, 6/8/1937, D.J. & J.N.Knull, (1, OSUC); AZ, Pima Co., Tucson, 8/26/1946, R.H.Crandall, (1, LACM); AZ, Pima Co., Tucson, 7/10/1946, R.H.Crandall, (1, LACM); AZ, Pima Co., Sabino Canyon, Santa Catalina Mts., 7/7/1960, F.Werner & P.H.Johnson, UV light (2, UAIC); AZ, Pima Co., Sabino Canyon, Santa Catalina Mts., 8/1/1962, F.Werner,, UV light (2, UAIC); AZ, Pima Co., Tucson, 7/11/1959, L.B.Koenig, at light (1, FSCA); AZ, Pima Co., Tucson, 5/3/1958, CAWood, (1, UAIC); AZ, Maricopa Co., Gila Bend, 4/7/2008, L.A.Stange, (1, FSCA); AZ, IBP:Silverbell Site, Sect.21, T11S R9E, Invert.plot UA, 7/17/1971, Emergence trap 106 under ceramic (1, UAIC); AZ, Pima Co., Tucson, 7/3/1961, L.B.Koenig, at light (1, UAIC); AZ, Pima Co., Tucson, 6/21/1961, L.B.Koenig, at light (2, UAIC); AZ, Pima Co., W. of Tucson, Tucson Mt. Park, 3/25-27/1986, P.Skelley, light trap (1, FSCA); AZ, Pima Co., Tucson, 5/10/1942, A.L.Melander, (1, UCRC); AZ, Pima Co., Organ Pipe NM, 4/11/1965, G.L.Jensen & W.J.Turner, (1, EMEC); AZ, Pima Co., Organ Pipe NM, 4/11/1947, A.L.Melander, (1, UCRC); AZ, Pima Co., Organ Pipe NM, 4/18/1947, A.L.Melander, (1, UCRC); AZ, Pima Co., Organ Pipe NM, Quitobaquito Oasis, 4/11/1973, S&S Frommer, at white light,7.30pm-8.55pm (1, UCRC); AZ, Pima Co., Organ Pipe NM, 3/10/1984, Olson, (1, UAIC); AZ, Pima Co., Soldier Canyon, 14 km. NE of Tucson, 3/24/1986, 950m, Steiner & Lowry, at black light in Sonoran Desert scrub (1, USNM); AZ, Pima Co., Tucson, 6/12/1948, R.H.Crandall, (1, LACM); AZ, Pima Co., Tucson, 4/23/1958, Bruner, (3, UAIC); AZ, Pima Co., Tucson, 5/3/1958, T.?, (1, UAIC); AZ, Pima Co., Tucson, 5/2/1958, Moore, (1, UAIC); AZ, Pima Co., Tucson, 5/9/1958, Dobson, (1, UAIC); AZ, Pima Co., Tucson, 5/3/1958, Bruner, (1, UAIC); AZ, Pima Co., Tucson, 4/27/1958, Tilt, (1, UAIC); AZ, Pima Co., Tucson, 4/25/1958, R.R.Frost, (1, UAIC); AZ, Pima Co., Tucson, 10/12/1959, G.T.Bottger, blacklight trap (1, UAIC); AZ, Pima Co., Tucson, 4/16/1958, Peltz, (1, UAIC); AZ, Pima Co., Tucson, 4/20/1958, Crisman, (1, UAIC); AZ, Yuma Co., Yuma, 6/1/1937, R.C.,Dickson, (1, UCRiverside); AZ, Yuma Co., Yuma, 7/22/1925, Brooklyn Museum Collection 1929 (2, USNM); AZ, Yuma Co., Yuma, _5/20/1958,,, V.Roth,, at lights (1, UA); AZ, Yuma Co., Yuma, 1/1/1972, S.Kirkpatrick, on brick wall (1, ASUT); AZ, Yuma Co., Roll, 6/29/1939, L.L.Stitt, (1, ASUT); AZ, Maricopa Co., Tempe, 4/3/1972, S.C.Burns, near light (1, ASUT); AZ, Maricopa Co., Tempe, 4/8/1966, F.F.Hasbrouck, reared (1, ASUT); AZ, Maricopa Co., Tempe, 6/27/1964, F.F.Hasbrouck, at light (1, ASUT); AZ, Maricopa Co., Tempe, 6/20/1964, F.F.Hasbrouck, at light (1, ASUT); AZ, Maricopa Co., Mesa, 7/14/1959, S.A.Gorodenski, (1, ASUT); AZ, Maricopa Co., Mesa, Main St. and Recker Rd., 4/25/1966, Brennan, black light (7, ASUT); AZ, Maricopa Co., Ft. McDowell, 4/23/1964, G.McRaven, at light (1, ASUT); AZ, Maricopa Co., Germann and Higley, 5/9/1966, Brennan, black light (1, ASUT); AZ, Maricopa Co., Casa Grande, 4/22/1973, N.Kendle, on car window, Blattellidae (1, ASU); AZ, Maricopa Co., Liberty, 2/21/1974, B.Grandy, in house (1, ASUT); AZ, Maricopa Co., Thunderbird Park, 4/27/1979, Wielgus & Hasbrouck, at UVBL (1, ASUT); AZ, Maricopa Co., Tempe, 6/25/1964, F.F.Hasbrouck, at light (1, ASUT); AZ, Maricopa Co., Tempe, 4/24/1974, H.C.Conklin, at light (1, ASUT); AZ, Maricopa Co., Tempe, 10/1/1974, L.Porzer, on ground (1, ASUT); AZ, Pima Co., Tucson, vic. Ina/Oracle, 7/9/1989, W.L.Nutting, pool (2, UAIC); AZ, Pima Co., Tucson, 7/17/1966, M.L.Noller, at light (1, UAIC); AZ, Pima Co., Catalina SP, Santa Catalina Mts., 8/2/1996, Olson, (1, UAIC); AZ, Pima Co., ASDM, Tucson, 4/2/1953, W.W.Larson, light (1, UAIC); AZ, Pima Co., Tucson, 4/10/1969, F.G.Werner, UV trap (3, UAIC); AZ, Pima Co., Tucson, 4/11/1958, J.May, (1, UAIC); AZ, Pima Co., Tucson, 1/10/1969, R.H.Russell, (1, UAIC); AZ, Pima Co., Tucson, 11/6/1966, R.Rice, under board (7, UAIC); AZ, Pima Co., Tucson, 12/11/1966, R.Rice, under log (2, UAIC); AZ, Pima Co., Tucson, 1/29/1967, R.Rice, under door (4, UAIC); AZ, Pima Co., Tucson, 11/8/1967, R.Rice, under board (5, UAIC); AZ, Pima Co., Tucson, 1/22/1967, R.Rice, under board (6, UAIC); AZ, Pima Co., Tucson, R.Rice, black light trap (1, UAIC); AZ, Pima Co., Tucson, 1/24/1967, R.Rice, under door (2, UAIC); AZ, Pima Co., Helmet Peak, 1/31/1967, R.Rice, rock pile (2, UAIC); AZ, Pima Co., Tucson, 8/11/1965, R.Rice, black light trap (1, UAIC); AZ, Pima Co., Tucson Mts. Rear hill near A Mtn., 11/24/1966, rock pile (1, UAIC); AZ, Pima Co., 16 mi. W of Tucson, 8/13/1988, (1, LACM); AZ, Pima Co., Tucson, Catalina Foothills, 8/23/2001, 32.18N, 110.56W, 762.2m, W.Moore, WM01.029 (1, UAIC); AZ, Pima Co., Waterman Mts., 10/1990-4/1991, Olson & Van Devender, pitfall trap (27, UAIC); AZ, Pima Co., Waterman Mts., 5/19-7/7/1991, Olson, pitfall trap (3, UAIC); AZ, Pima Co., Waterman Mts., 4/6-5/19/1991, Olson & Van Devender, pitfall trap (11, UAIC); AZ, Maricopa Co., Phoenix, 4/1/1915, R.E.Kunze, (1, USNM); AZ, Maricopa Co., Phoenix, 4/1/1915, R.E.Kunze, genit. lost, Paratype No.21879 U.S.N.M., *Arenivaga genitalis* parat. “a” Caud. (1, USNM); AZ, Catalina Springs, 7/4/????, paratype d, *Arenivaga genitalis* parat. “c” Caud. (1, USNM); AZ, Yuma Co., Wellton, 6/3/1939, L.L.Stitt, at light,H-175 (1, USNM); AZ, Pima Co., Tucson, 4/15/1953, R.S.Beal, (1, EMEC); AZ, Pinal Co., Goldfield, 8/9/1950, R.S.Beal, (1, EMEC); AZ, Maricopa Co., Higley, 6/18/1917, E.G.Holt, at light (1, USNM); AZ, Maricopa Co., Tempe, 6/5/1951, H.S.Wallace, No.1811 (1, UMMZ); AZ, Maricopa Co., Tempe, 7/3/1951, H.S.Wallace, No.1833 (1, UMMZ); AZ, Maricopa Co., Tempe, 7/6/1951, H.S.Wallace, No.1836 (2, UMMZ); AZ, Maricopa Co., Tempe, 7/7/1951, H.S.Wallace, No.1838 (6, UMMZ); AZ, Maricopa Co., Tempe, 5/8/1951, H.S.Wallace, No.1770 (2, UMMZ); AZ, Maricopa Co., Tempe, 5/6/1951, H.S.Wallace, No.1769 (1, UMMZ); AZ, Maricopa Co., Tempe, 4/10/1951, H.S.Wallace, No.1754 (1, UMMZ); AZ, Maricopa Co., Tempe, 3/21/1951, H.S.Wallace, No.1754 (1, UMMZ); AZ, Maricopa Co., Tempe, 6/9/1951, H.S.Wallace, No.1752 (1, UMMZ); AZ, Maricopa Co., Tempe, 7/4/1951, H.S.Wallace, No.1834 (2, UMMZ); AZ, Maricopa Co., Tempe, 7/10/1951, H.S.Wallace, No.1841 (2, UMMZ); AZ, Maricopa Co., Tempe, 7/22/1951, H.S.Wallace, No.1857 (1, UMMZ); AZ, Maricopa Co., Tempe, 7/8/1951, H.S.Wallace, No.1840 (1, UM); AZ, Pinal Co., 10 mi. S of Casa Grande, 5/29/1942, E.R.Tinkham, (1, HEH); AZ, Pima Co., Sabino Canyon, 8/6/1959, K.V.Krombein, (1, USNM); AZ, Pima Co., Ajo, 4/23/1952, E.R.Tinkham, red tag T9 (1, USNM); AZ, Pima Co., Tucson, 4/11/1956, Fida, (1, UAIC); AZ, Pima Co., Tucson, 3/28/1958, Howell, (1, UAIC); AZ, Pima Co., Tucson, 4/20/1956, Robertson, (1, UAIC); AZ, Pima Co., Tucson, ?/11/1956, ?, (1, UAIC); AZ, Pima Co., Tucson, 4/9/1956, Massman, (1, UAIC); AZ, Pima Co., Tucson, 4/4/1952, Simons, (1, UAIC); AZ, Pima Co., Tucson, 6/30/1952, G.D.Butler, at light (1, UAIC); AZ, Pima Co., Tucson, 3/28/1957, Howell, (2, UAIC); AZ, Pima Co., Tucson, 5/3/1956, Brandt, (2, UAIC); AZ, Pima Co., Tucson, 3/1/1956, ?, (1, UAIC); AZ, Pima Co., Tucson, 4/9/1956, Togi, (1, UAIC); AZ, Pima Co., Tucson, 3/6/1951, Yunt, (1, UAIC); AZ, Pima Co., Tucson, 3/14/1951, Platt, (2, UAIC); AZ, Pima Co., Tucson, 6/27/1954, M.Crazier, (1, AMNH); AZ, Pima Co., Tucson, 6/30/1949, 2200’, G.M.Brandt, (3, AMNH); AZ, Pima Co., Tucson, 6/11/1954, M.Crazier, (1, AMNH); AZ, Pima Co., Ajo, 8/16/1952, C & P Vaurie, (2, AMNH); AZ, Pima Co., Tucson, 4/4/1950, R.W.Simpson, (1, UAIC); AZ, Pima Co., Tucson, 3/31/1950, R.W.Simpson, (1, UAIC); AZ, Pima Co., Tucson, 4/10/1950, R.W.Simpson, (1, UAIC); AZ, Yuma Co., Yuma, 10/4/1959, V.Roth, (1, UAIC); AZ, Pima Co., Sabino Canyon, Santa Catalina Mts., 7/30/1954, F.G.Werner, lt. (5, UAIC); AZ, Pima Co., Sabino Canyon, 7/9/1952, Beamer,LaBerge,Wolf,Liang & Winer, (1, SEMC); AZ, Pima Co., Sabino Canyon, Santa Catalina Mts., 7/2/1956, Butler & Werner, lt. (1, UAIC); AZ, Pima Co., Tucson, 4/13/1944, M.H.Froat,Jr., (1, UAIC); AZ, Pima Co., Tucson, 6/17/1930, J.Yuill, (1, UAIC); AZ, Pima Co., Tucson, 4/9/1940, S.L.Green, (1, UAIC); AZ, Pima Co., Tucson, 3/28/1943, T.J.Smith, in house, Lot 102 Sublot 416 (1, UAIC); AZ, Pima Co., Tucson, 4/?/1952, Nelson, (1, UAIC); AZ, Pima Co., Tucson, 6/18/1932, R.A.Flock, (1, UAIC); AZ, Pima Co., Tucson, 6/7/1932, R.A.Flock, (2, UAIC); AZ, Pima Co., Tucson, 6/27/1932, R.A.Flock, (1, UAIC); AZ, Pima Co., Tucson, 6/20/1932, R.A.Flock, (1, UAIC); AZ, Pima Co., Tucson, 7/5/1932, R.A.Flock, (1, UAIC); AZ, Pima Co., Tucson, 7/7/1932, R.A.Flock, (2, UAIC); AZ, Pima Co., Tucson, 7/1/1932, R.A.Flock, (1, UAIC); AZ, Pima Co., Tucson, 7/4/1932, R.A.Flock, (1, UAIC); AZ, Santa Cruz Co., near mouth of Peck Canyon, 4/17/1950, R.B.Miller, P.M. (1, UMMZ); AZ, Pima Co., Ajo, 3/31/1923, O.Poling, (1, ANSP); AZ, Pima Co., Sabino Canyon, Baboquivari Mts., 4/15/1954, L.M.Martin, (3, LACM); AZ, Pima Co., Sabino Canyon, Baboquivari Mts., 4/12/1954, L.M.Martin, (1, LACM); AZ, Pima Co., Sabino Canyon, Baboquivari Mts., 7/30/1949, L.M.Martin, (1, LACM); AZ, Pima Co., Tucson, 5/?/1949, (1, LACM); AZ, Maricopa Co., Tempe, 4/14/1951, H.S.Wallace, No.1757 (1, UMMZ); AZ, Pima Co., Tucson, 7/20/1975, D Foster, (1, NMSU); AZ, Pima Co., Tucson, 5/10/1964, 24-2600’, J Wehner, (2, FSCA); AZ, Pima Co., Tucson, 5/3/1964, J Wehner, (1, FSCA); CA, Yuma, H.Wickham, (1, ANSP); CA, Imperial Co., Andrade, 8/4/1966, M.Wasbauer, Fluorescent black light (1, CSCA); CA, Imperial Co., Bard, 7/11/1966, Ratcliff, Argon light trap (1, CSCA). MEXICO, Sonora, Pitiquito, 7/4/1952, C & P Vaurie, (3, AMNH); Sonora, 40 mi. S of Sonoyta, 3/19/1981, Werner,Olson & Burns, (2, UAIC); Sonora, 40 mi. N of El Saguaro, 3/18/1981, Werner,Olson & Borne, (1, UAIC). Determiner label *Arenivaga genitalis* Hopkins 2011” [white label with black border].

##### Distribution.

This species is found in south central Arizona, extending south into Mexico and west to southwestern Arizona. See Fig. 68.

##### Diagnosis.

*Arenivaga genitalis* may be diagnosed by the appearance of the right ventral phallomere which has three spines and a knob projecting from the dorsal surface. See Fig. 67.

##### Description.

**Male.** **NB: Holotype is broken and pieces are glued to card. Description is blend of holotype and another complete specimen.** *Measurements*. Holotype TL = 15.8 mm, GW = 7.7 mm, PW = 5.17 mm, PL = 3.60 mm, TL/GW = 2.05, PL/PW = 0.70. EW = 0.40 mm; OW = 0.50 mm. Among paratypes range of TL 15.3–21.8 mm; range of GW 6.9–9.8 mm; range of PW 5.00–6.34 mm; range of PL 3.60–4.66 mm.

**Figure 66.**
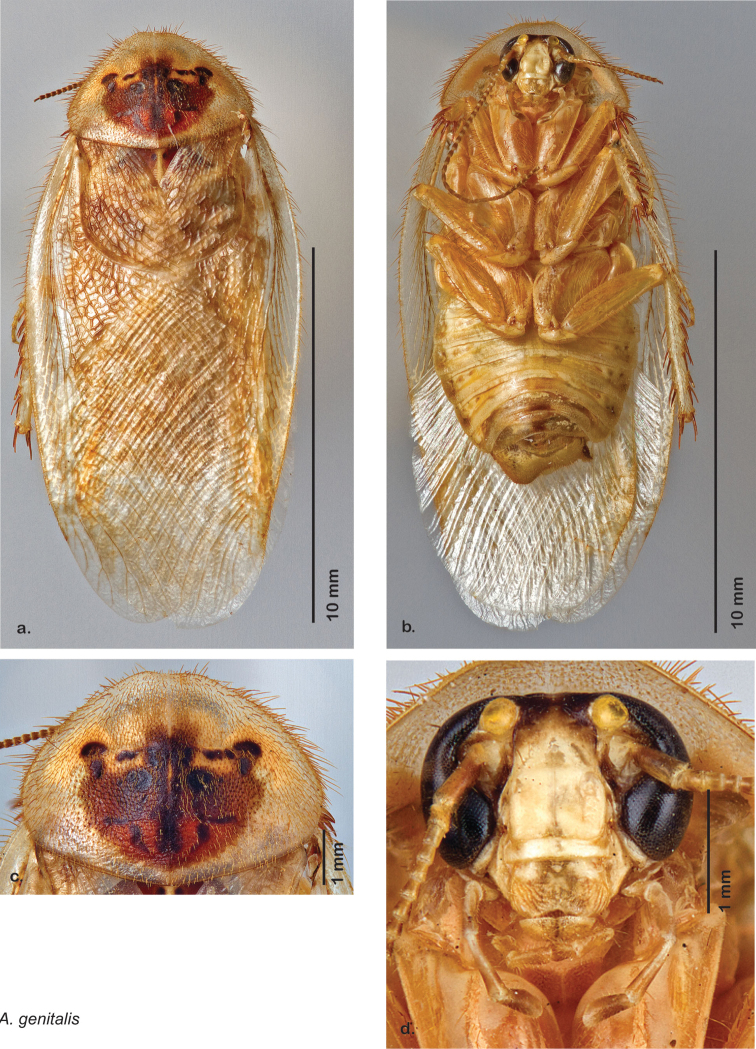
*Arenivaga genitalis* **a** dorsal habitus **b** ventral habitus **c** pronotum **d** head.

*Head*. Two ocelli large, ovoid and protruding (0.40 × 0.30 mm); vertex medium brown, with small ridges between apices of eyes extending on to ocellar tubercles, scattered short setae; interocellar space concave, light brown. Frons waxy white, concave; bound on either side by ridges extending from inner apex of ocelli outwards to lateral edges of clypeus; scattered long setae on ridges. Anterior portion of frons waxy white, bulbous; clypeal suture demarcates waxy white anteclypeus. See Fig. 66d.

*Pronotum*. Pronotum translucent waxy beige; variable length orange-brown setae along anterior margin; dorsal surface of pronotum covered with short orange-brown setae that are denser and longer anteriorly and laterally; pronotal pattern dark orange-brown “panther face”, dark brown in some specimens, with little to no aura (aura usually anterior) and discernible detail. See Fig. 66c.

*Body*. Wing brace present. Legs and body medium orange-brown; subgenital plate asymmetrical with posterior edge emarginated, rounded apices. See Fig. 66b.

*Forewings*. Wings extended beyond abdominal apex (up to ~30% of total wing length); color highly variable from light brown, to light orange-brown, to medium and dark brown; always blotchy; surface semi-transparent and matte, or with faint sheen on many specimens. See Fig. 66a.

**Figure 67.**
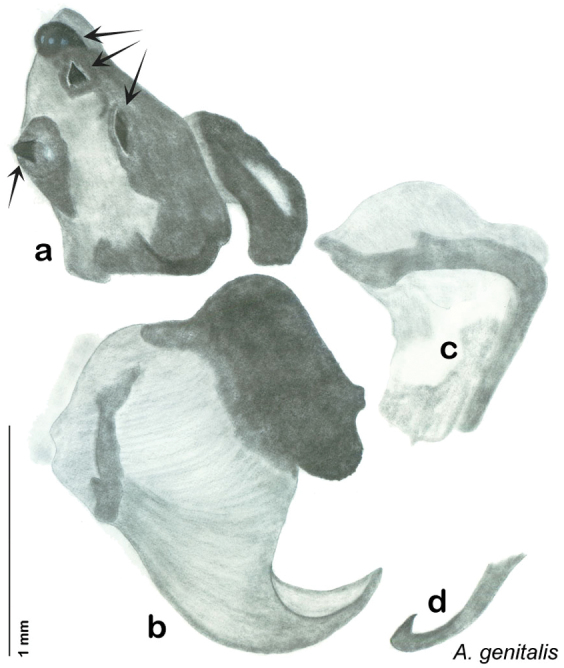
*Arenivaga genitalis*, genitalia: a) right dorsal phallomere **b** right ventral phallomere **c** small central sclerite **d** genital hook. Arrow(s) indicate diagnostic characters (see text).

*Genitalia*. Right dorsal phallomere composed of bulbous lightly sclerotized narrow ended hook-shaped lobe, articulated with right ventral phallomere on lateral side; central field lightly sclerotized; medial margin sclerotized, smooth, concave in ventral view; medial margin smoothly curved at anterior end, shagreened greatly extended knob at posterior end; dorsal edge has central emargination with small spine in center, broader spine posterior to that. Small central sclerite smooth, concave, with field of punctations on interoventral surface. Right ventral phallomere extends from articulation into posterior pointing spine, nearby similarly directed smooth knob with adjacent small spine, and nearby small punctate flange; recedes anteriorly in wavy punctate corrugations; after narrow gap, broad, wavy, punctate arm extending to depth of rest of phallomere. Folded anterior portion of left phallomere missing on holotype. Genital hook with broad pointed head and moderate hook; arm narrow. See Fig. 67.

**Figure 68.**
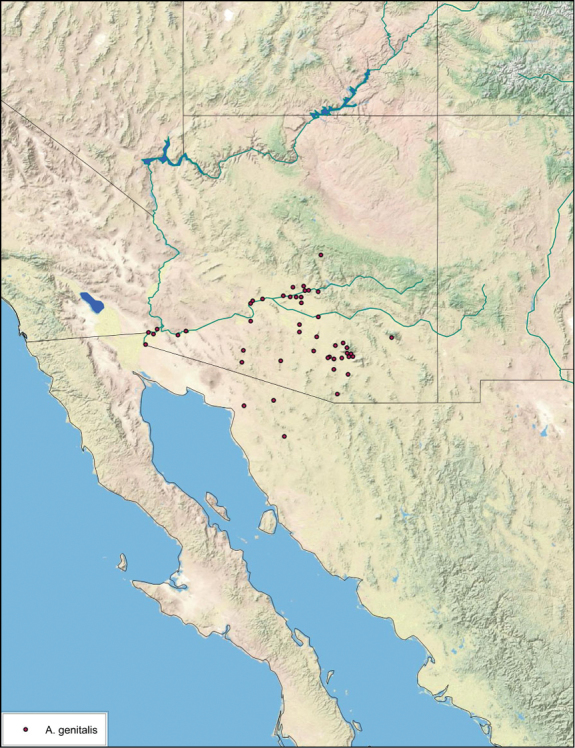
*Arenivaga genitalis*, distribution.

##### Habitat and natural history.

All life history elements remain unobserved.

#### Arenivaga grandiscanyonensis sp. n.

http://zoobank.org/4C6ACE63-5051-48ED-AD80-71CC3CD92053

http://species-id.net/wiki/Arenivaga_grandiscanyonensis

Figures 69 – 71

##### Type locality.

USA, Arizona, Mohave Co., Colorado River, Grand Canyon.

##### Material examined.

Holotype: ♂ in NAUF labeled “Mohave Co. AZ, Colorado R. GC, rm211.5R, 4/13/02, Coll. R.J.Delph, Ex: Light, Old High Water, blue label with ‘3’,” “HOLOTYPE *Arenivaga grandiscanyonensis* Hopkins, 2012” [red label with black border].

Paratypes (2): USA: AZ, Coconino Co., Colorado River GC, 5/10/2001, J Rundall, rm160.5L, ex.light old high water, blue label ‘3’, 1 specimen--NAU 106 (2, NAUF). All paratypes labeled “Paratype *Arenivaga grandiscanyonensis* Hopkins 2012” [blue label with black border].

##### Etymology.

This species is named for the only place this species has been documented, the Grand Canyon.

##### Distribution.

This species is found at the base of the Grand Canyon, Arizona. See Fig. 71.

##### Diagnosis.

*Arenivaga grandiscanyonensis* may be confused with *Arenivaga pagana* but may be distinguished by the shagreened tongue arching anteriorly out of the central field of the right dorsal phallomere. See Figs 70 and 121.

##### Description.

**Male.** *Measurements*. Holotype TL = 18.8 mm, GW = 9.2 mm, PW = 4.90 mm, PL = 4.28 mm, TL/GW = 2.04, PL/PW = 0.87. EW = 0.35 mm; OW = 0.30 mm. No notable differences in measurements among paratypes.

**Figure 69.**
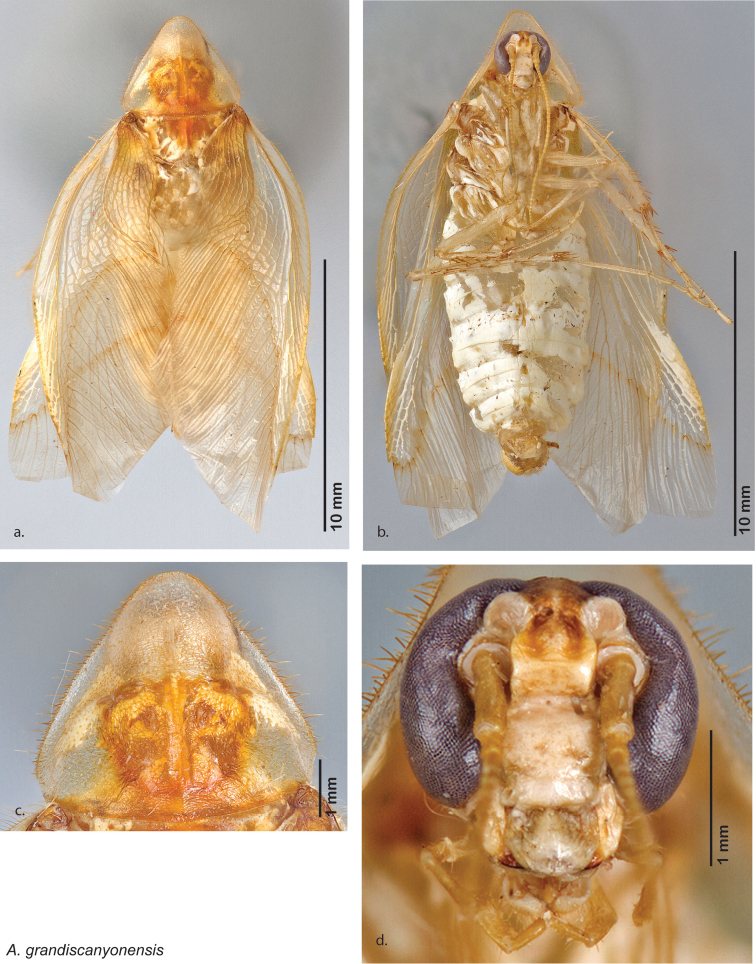
*Arenivaga grandiscanyonensis* **a** dorsal habitus **b** ventral habitus **c** pronotum **d** head.

*Head*. Two ocelli large, ovoid and protruding (0.35 × 0.25 mm); vertex medium brown with small ridges in rays around upper apices of eyes and extending onto ocellar tubercles; interocellar space concave, light brown, lighter brown medially, with two vertical indentations. Frons waxy white, posterior slightly concave; anterior portion of frons bulbous and waxy white; waxy white smooth anteclypeus. See Fig. 69d.

*Pronotum*. Pronotum translucent waxy beige; dorsal surface of pronotum with short orange-brown setae that are slightly thicker laterally; pronotal pattern orange-brown “panther face”, little discernible detail; no aura. See Fig. 69c.

*Body*. Wing brace present. Two tarsal claws present. Legs and body pale beige; subgenital plate light brown with darker margin; asymmetrical with angular apices. See Fig. 69b.

*Forewings*. Wings extended well beyond abdominal apex (~30% of wing length); translucent light beige and hyaline. See Fig. 69a.

**Figure 70.**
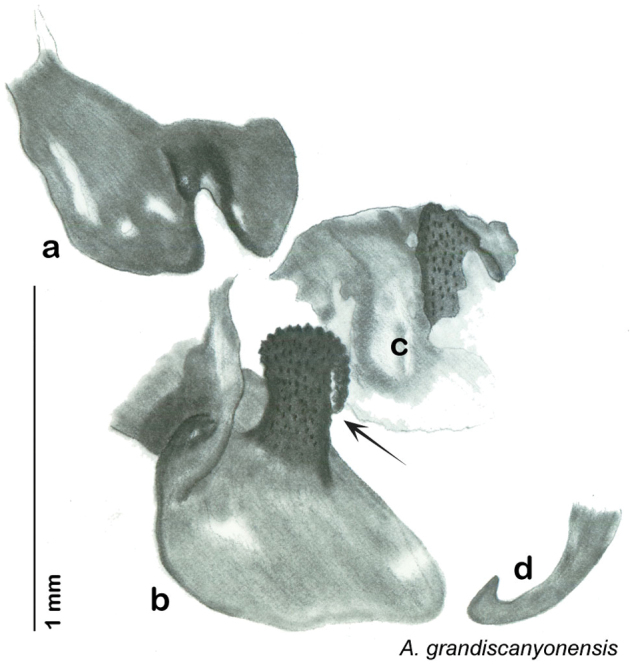
*Arenivaga grandiscanyonensis*, genitalia: a) right dorsal phallomere **b** right ventral phallomere **c** small central sclerite **d** genital hook. Arrow(s) indicate diagnostic characters (see text).

*Genitalia*. Right dorsal phallomere composed of a bulbous lightly sclerotized bulbous lobe, articulated with right ventral phallomere on lateral side; central field with anteriorly projecting flat shagreened arm with toothed edge. Small central sclerite with smooth curved sculpturing, posterior edge flattened and shagreened with laterally projecting smooth flap; right ventral phallomere extends from articulation to form smooth rounded structure becoming lightly punctate anteriorly; attached anteriorly is broad dorsally projecting punctate arm that extends only to depth of rest of phallomere. Folded anterior portion of left phallomere finely punctate, otherwise unmodified. Genital hook with short extension to pointed head with short hook and distinct bend in arm. See Fig. 70.

**Figure 71.**
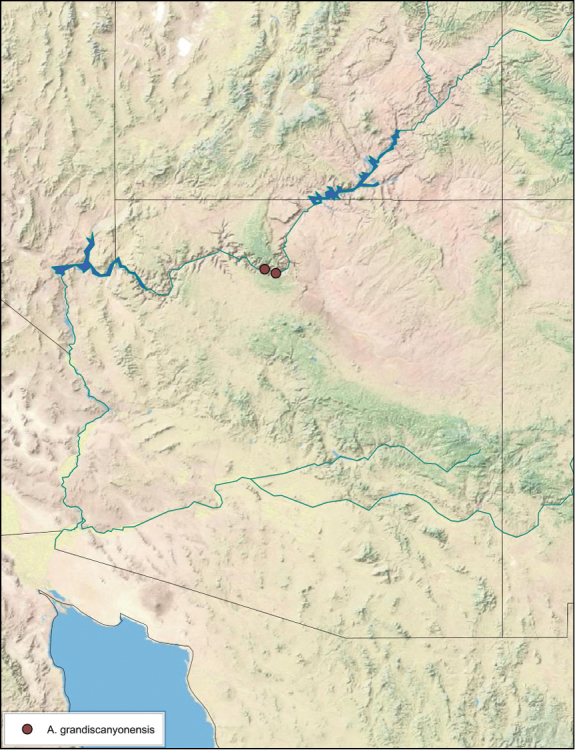
*Arenivaga grandiscanyonensis*, distribution.

##### Habitat and natural history.

All life history elements remain unobserved.

#### Arenivaga grata

Hebard

http://species-id.net/wiki/Arenivaga_grata

Figures 72 – 74

Arenivaga grata Hebard 1920, Transactions of the American Entomological Society, 46(2), pp. 197–217.

##### Material examined

**(169).** USA, AZ, Pima Co., Baboquivari Canyon W side of Baboquivari Mts., 7/23-27/1952, Leech & Green (2, CAS); AZ, Pima Co., Brown’s Canyon E side of Baboquivari Mts., 7/29-30/1952, Leech & Green (8, CAS); AZ, Co., Miami, 8/6/1941, E.L.Todd (1, ANSP); AZ, Pima Co., Baboquivari Mts., 7/1-15/1924, 4000’, O.C.Poling (23, UMMZ ); AZ, Pima Co., Baboquivari Mts., 7/24/1941, L.H.Banker (1, ANSP); AZ, Pima Co., Baboquivari Mts., 11/1-15/1928, O.C.Poling (1, MCZ); AZ, Pima Co., Baboquivari Mts., 9/?/1924, 4000’, O.C.Poling (1, UMMZ ); AZ, Pima Co., Baboquivari Mts., 9/15-30/1923, 5000’, O.C.Poling (5, UMMZ ); AZ, Pima Co., Baboquivari Mts. Camp, 7/4/1970, CA & WE Triplehorn (1, OSUC); AZ, Pima Co., SASI:Tucson Mountain Park, 9/17/1998, M.Ture, UV,Blattaria,Blattidae (1, UAIC); AZ, Pima Co., Tucson, S.R.Exp.Range, 9/20/2005, A.Beyerlein (1, UAIC); AZ, Pima Co., Molino Basin Picnic Area, Catalina Mts., 8/21/1973, 4690’, Garrison & Kolner, at light (1, ASUT); AZ, Cochise Co., Guadalupe Canyon, 29 mi. E of Douglas, 8/15-16/1972, J.Doyen, black light trap (1, EMEC); AZ, Santa Cruz Co., Pena Blanca Canyon,0.4 mi. NE of Castle Rock, 9/19/1973, 4200’, S.L.Szerlip, at blacklight (1, EMEC); AZ, Santa Cruz Co., Pena Blanca Lake, 8/12/1993, 31.38N, 111.08W, B.V.Brown (1, LACM); AZ, Pima Co., Chutum Vaya canyon, W slope of Baboquivari Mts., 8/4/1966, 31.43N, 111.37W, 3250’, F.Werner family, light trap (2, UAIC); AZ, Pima Co., Tucson Mtn. Park caretaker’s house, 10/19/1981, S.Pechal (1, UAIC); AZ, Pima Co., Molino Basin, Santa Catalina Mts., 7/28/1968, F.Werner (3, UAIC); AZ, Pima Co., Brown’s Canyon E side of Baboquivari Mts., 8/8/1953, G.D.Butler (1, UAIC); AZ, Pima Co., Brown’s Canyon E side of Baboquivari Mts., 9/6/1958, Menke & Stange (1, LACM); AZ, Pima Co., Brown’s Canyon E side of Baboquivari Mts., 9/5-6/1953, L.Martin (6, LACM); AZ, Pima Co., Quinlan Mts., 9/3/1931, E.R.Tinkham (1, ANSP); AZ, Pima Co., Sabino Canyon, 9/6/1951, E.R.Tinkham (2, USNM); AZ, Pima Co., Molino Basin, Santa Catalina Mts., 8/29/1951, C.D.MacNeill (2, EMEC); AZ, Pima Co., Kits Peak, Baboquivari Mts., 8/1-4/1916, 31.57N, 111.33W, 4050’, (2, ANSP); AZ, Pima Co., Baboquivari Mts., 11/1-15/1923, O.C.Poling (5, UMMZ ); AZ, Pima Co., Baboquivari Mts., 6/15-30/1924, 4000’, O.C.Poling (11, UMMZ ); AZ, Pima Co., Baboquivari Mts., 9/1-15/1923, 5000’, O.C.Poling (14, UMMZ ); AZ, Pima Co., Baboquivari Mts., 11/1-10/1923, O.C.Poling (1, UMMZ ); AZ, Pima Co., Baboquivari Mts., 5/15-30/1924, 4000’, O.C.Poling (8, UMMZ ); AZ, Pima Co., Baboquivari Mts., 10/1-15/1923, O.C.Poling (2, UMMZ ); AZ, Pima Co., Baboquivari Mts., 7/15-30/1924, 4000’, O.C.Poling (1, UMMZ ); AZ, Pima Co., Baboquivari, 6/1-15/1924, (1, UMMZ ); AZ, Pima Co., Baboquivari Mts., 10/15-30/1923, O.C.Poling (4, UMMZ ); AZ, Pima Co., Baboquivari Mts., F.H.Snow (1, ANSP); TX, Brewster Co., Chisos Mts. Juniper Canyon, 7/17/1928, F.M.Gaige, 238 (6, UMMZ ); TX, Brewster Co., Chisos Mts. Juniper Canyon, 7/21/1928, F.M.Gaige, 237 (4, UMMZ ); TX, Brewster Co., Chisos Mts. Upper Juniper Spr., 7/18-30/192?, F.M.Gaige (1, UMMZ ); TX, Brewster Co., Chisos Mts. Juniper Canyon, 7/12/1928, F.M.Gaige, 183 (1, UMMZ ); TX, Brewster Co., Chisos Mts. Juniper Canyon, 7/8/1928, F.M.Gaige, 150 (1, UMMZ ); TX, Brewster Co., Big Bend Basin, Big Bend NP, 6/27-7/4/1965, A & M Blanchard (2, LACM); TX, Brewster Co., Chisos Mts. Below the Basin area, 10/7/1982, E.G.Riley (1, TAMU); TX, Brewster Co., BBNP Green Gulch, 8/2/2003, 29.17.19N 103.16.37W, 4900’, E.G.Riley, UV light (15) (1, TAMU); TX, Brewster Co., BBNP Pine Canyon Camp Area no. 4, 10/1/2005, 29.15.59N 103.14.04W, 4700’, Raber & Riley, 57 (1, TAMU); TX, Brewster Co., The Basin, Big Bend NP, 10/4/1956, J.W.MacSwain (2, EMEC); TX, Brewster Co., Big Bend NP, Chisos Mt. Basin, 5/27/1974, J.R.Powers (1, EMEC); TX, Brewster Co., Chisos Mts., 7/9-12/1948, 5260’, Nutting & Werner, pinon-juniper-oak, *Arenivaga rehni* Hebard det.W.Nutting ‘50 (1, UAIC); MEXICO, Durango, Tlahualilo, 7/20/1934, CS Rude, 490 (2, TAMU); Durango, Tlahualilo, 7/1/1934, Mrs. CS Rude, 329 (1, TAMU); Durango, Tlahualilo, 5/27/1935, CS Rude, 1087 (1, TAMU); Durango, Tlahualilo, 7/19/1934, CS Rude, 478 (1, TAMU); Durango, Tlahualilo, 8/2/1934, CS Rude, 619 (1, TAMU); Durango, Tlahualilo, 8/16/1934, CS Rude, 891 (1, TAMU); Aguascalientes, Aguascalientes, 6/20/1953, C & P Vaurie, D.Rockefeller Mex.Exp.1953 (1, AMNH); Durango, Tlahualilo, 8/15/1934, Mrs. CS Rude, 884 (2, TAMU); Durango, Tlahualilo, 8/12/1934, CS Rude, 876 (1, TAMU); Durango, Tlahualilo, 7/9/1934, Mrs. CS Rude, 424 (1, TAMU); Durango, Tlahualilo, 8/7/1934, CS Rude, 682 (1, TAMU); Durango, Tlahualilo, 6/24/1934, CS Rude, 228 (1, TAMU); Durango, Gomez, Palacio, 5/?/1918, A.Busck. (2, USNM); Durango, Gomez, Palacio, 5/?/1918, A.Busck., *Arenivaga bolliana* Sauss. Det. A.N.C. (1, USNM); Durango, Tlahualilo, 5/15/1935, CS Rude, 1092 (1, TAMU); San Luis Potosi, Las Tablas, 10/11/1931, A Dampf (1, ANSP); Sonora, Guaymas area, Nacapule Canyon, 10/17/2003, 28.01N, 111.03W, SIB 2003.0038 (1, UAIC); Chihuahua, 63 mi. W of Santa Barbara, 7/20/1947, 5500 ft., Spieth, D.Rockefeller Exp. (1, AMNH); Coahuila, San Lorenzo, 5/?/1920, SH Scudder, 1214,Palmer,ex MCZ (1, ANSP); Coahuila, 17 mi. SE of Saltillo, 7/8/1980, Taylor & Sullivan (1, LACM); Coahuila, Torreon, 6/6/1927, A. Dampf (1, ANSP); AZ, Cochise Turkey Creek, 8/11/1975, S McCleve, lite (3, FSAC); TX, Chisos Mts. Basin, 6/25/1963, GH Nelson & family, ultraviolet light (1, FSAC); Coahuila, 28 mi SW Saltillo on road to Jame, 7/18/1963, RH Arnett, Jr., ER Van Tassell, Lot No. 747 (1, FSAC); Sonora, Rancho los Alisos, 9.4 km WSW of Aconchi, Sierra Aconchi, 9/2/12, 29.79833N, 110.31072W, 1301 m, TR Van Devender, AL Reina, Rocky canyon, sycamore riparian deciduous forest, oak woodland on slopes. (2(one in alcohol), HEH). Determiner label *Arenivaga grata* Hopkins 2011” [white label with black border].

##### Distribution.

This species is found in southeastern Arizona, with scattered records extending south into and across Mexico to the west side of the Sierra Madre Oriental, and an isolated record from far southern Texas. See Fig. 74.

##### Diagnosis.

*Arenivaga grata* may be confused phenotypically with *Arenivaga bolliana* but their distributions are distinct. If locality information is not available *Arenivaga grata* may be diagnosed by the unusual shape of the hook-shaped lobe and the prominent shagreened ridge running interior to the point of articulation, both on the right dorsal phallomere. See Fig. 73.

##### Description.

**Male.** *Measurements*. Holotype stand-in TL = 26.8 mm, GW = 11.5 mm, PW = 7.55 mm, PL = 5.16 mm, TL/GW = 2.33, PL/PW = 0.68. EW = 0.40 mm; OW = 0.70 mm. Among paratypes range of TL 21.4–29.2 mm; range of GW 9.5–12.8 mm; range of PW 6.36–8.35 mm; range of PL 4.22–5.38 mm.

**Figure 72.**
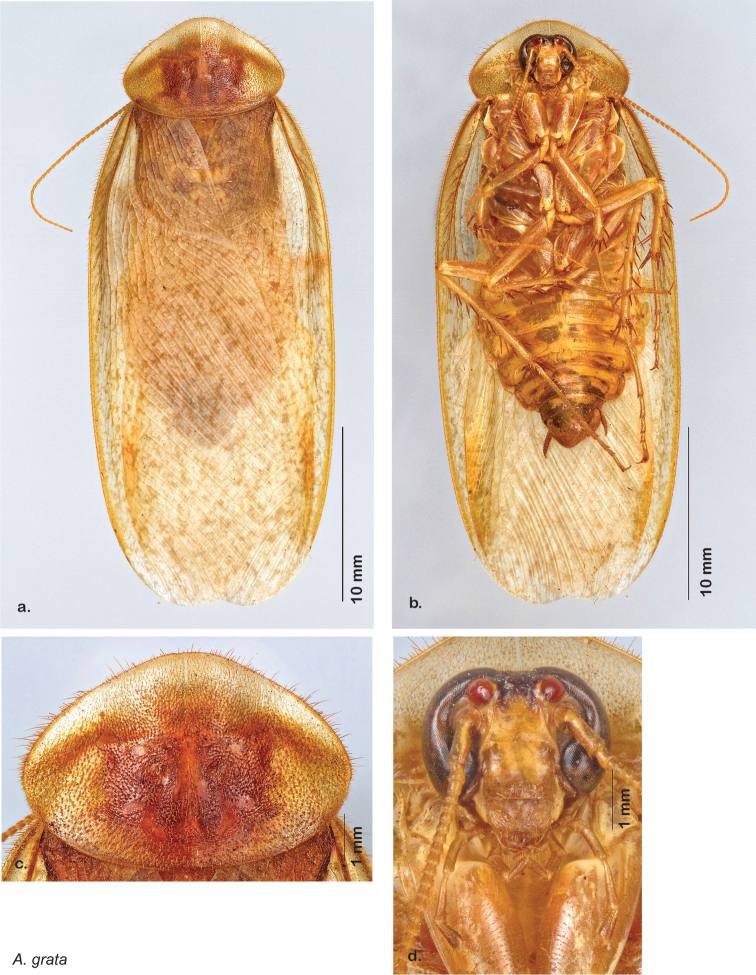
*Arenivaga grata* **a** dorsal habitus **b** ventral habitus **c** pronotum **d** head.

*Head*. Two ocelli large, ovoid and protruding (0.50 × 0.40 mm); vertex dark brown, with small ridges between apices of eyes extending on to ocellar tubercles, scattered short setae; interocellar space concave, medium to light brown; two round medium brown indentations laterally at the base of the interocellar space. Frons light brown, slightly concave; bound on either side by ridges extending from inner apex of ocelli outwards to lateral edges of clypeus; scattered setae on ridges. Anterior portion of frons light brown, bulbous; clypeal suture demarcates light brown anteclypeus. See Fig. 72d.

*Pronotum*. Pronotum with translucent waxy beige anterior margin; variable length orange-brown setae along anterior margin; dorsal surface of pronotum covered with short orange-brown setae; pronotal pattern medium orange-brown “panther face”, with little detail and complete lateral and posterior aura in light orange-brown; pronotal pattern runs from light orange-brown to dark brown in other specimens, pattern always little discernible, aura always complete laterally and posteriorly. See Fig. 72c.

*Body*. Wing brace absent. Legs and body medium orange-brown; subgenital plate asymmetrical with posterior edge only slightly emarginated, rounded apices. See Fig. 72b.

*Forewings*. Wings extended beyond abdominal apex (up to ~35% of total wing length); medium orange-brown with darker blotches; color variable in species from medium orange-brown, to medium and dark brown, always blotchy; surface opaque and matte. See Fig. 72a.

**Figure 73.**
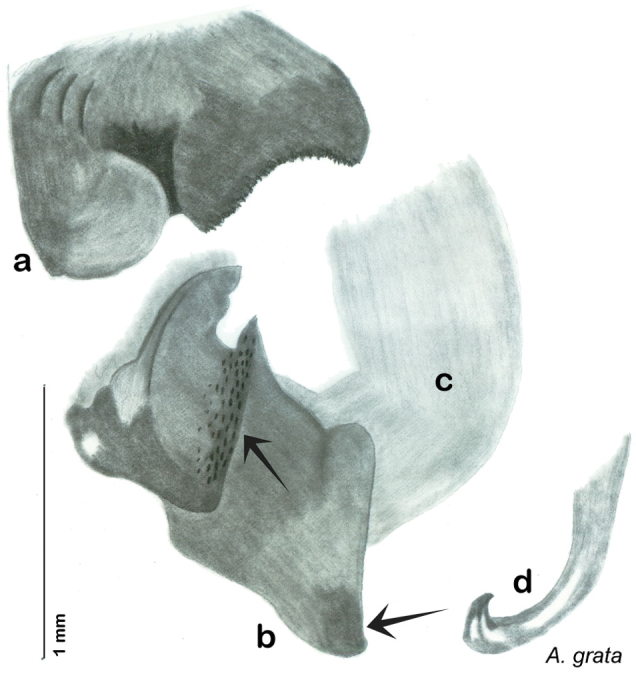
*Arenivaga grata*, genitalia: a) right dorsal phallomere **b** right ventral phallomere **c** small central sclerite **d** genital hook. Arrow(s) indicate diagnostic characters (see text).

*Genitalia*. Right dorsal phallomere composed of slightly bulbous lightly sclerotized narrow ended hook-shaped lobe with little to no curve to hook, articulated with right ventral phallomere on lateral side; central field lightly sclerotized; medial margin straight and contiguous with medial edge of hook-shaped lobe, lightly sclerotized, smooth, with no sculpturing of any kind. Small central sclerite nondescript in shape, flat, finely punctate. Right ventral phallomere extends from articulation into posterior pointing punctate lobe with small dorsal projection on posterior end; after moderate gap, broad, punctate flange with shagreened emarginate edge, extending to depth of rest of phallomere. Genital hook with broad pointed head and moderate hook with bent tip; arm broad and smoothly curving. See Fig. 73.

**Figure 74.**
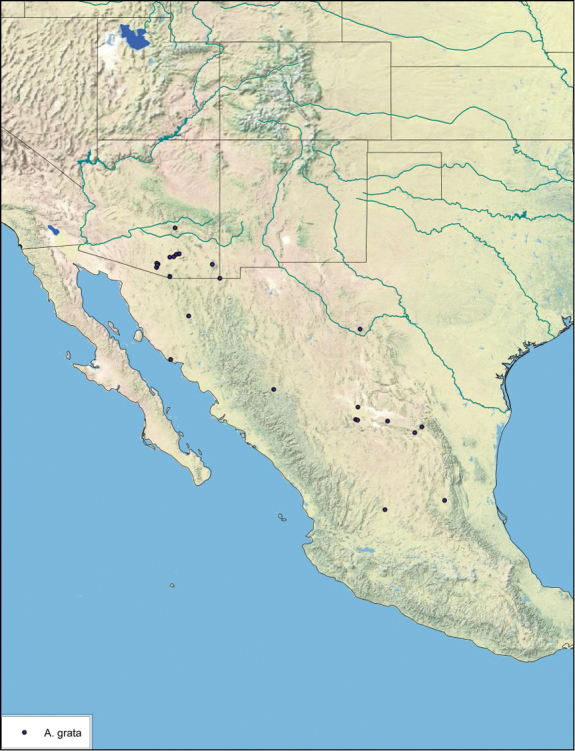
*Arenivaga grata*, distribution.

##### Habitat and natural history.

All life history elements remain unobserved.

#### Arenivaga gumperzae sp. n.

http://zoobank.org/E3E5D806-C42D-499E-848C-201739EECF9F

http://species-id.net/wiki/Arenivaga_gumperzae

Figures 75 – 77

##### Type locality.

MEXICO, Durango, near Pedricena.

##### Material examined.

Holotype: ♂ in ANSP labeled “near Pedricena, Durango, Mex, 8/27/1932, Hobart & Smith, ANS Lot 467” “HOLOTYPE *Arenivaga gumperzae* Hopkins, 2012” [red label with black border].

Paratypes (33): USA: TX, Pecos Co., 28 mi S of Ft. Stockton, 6/3/1998, R Turnbow, Blacklight (2, FSCA); TX, Kerrville, 9/?/1961 (1, TAMU); TX, Val Verde Co., Seminole Canyon State Historic Area, 8/30/1986, East, Kovarick & Haack (1, TAMU); TX, Kerrville, 9/4/1964, WF Chamberlain (1, TAMU); TX, Val Verde Co., Seminole Canyon State Historic Area, 4/1-7/1985, CB Barr, human dung pitfall (1, EMEC); TX, Presidio, 9/16/1929, ER Tinkham, (1, ANSP); TX, Pecos Co., 28 mi S of Ft. Stockton, Hwy. 385 rest stop, 4/19/1997, 30.28.57N 102.55.52W, E Riley, 469, (1, TAMU); TX, LaSalle Co., Chaparral WMA, Pasture 11, 9/11-10/10/2003, B Raber, pitfall, acacia area (1, TAMU); TX, Dimmit Co., 7/12/1940 (1, TAMU); TX, BBNP, Big Bend Basin, 6/27-7/4/1965, A & ME Blanchard (1, LACM); TX, Presidio, 3/26-5/15/1951, JH Russell, at lights (1, USNM). MEXICO: Coahuila, 5 mi. S of Monclova, 8/9/1977, EI Schlinger (1, EMEC); Coahuila, La Gloria, S of Monclova, 8/24/1947, 3300 ft., Cazier, D Rockefeller Exp. (6, AMNH); Coahuila, Torreon, 6/15/1957 (1, EMEC); Coahuila, 26 mi. E of Cuatro Cienegas, 8/2/1959, 1850 ft., TJ Cohn, #131 (4, UMMZ); Coahuila, 5 mi S of Hermanas, 8/1/1959, 1350 ft., TJ Cohn, #129 (1, UMMZ); Nuevo Leon, Monterrey, 4/23/1957, colectado por estudiante, Rockefeller Collection, return to Cantrell (2, UMMZ); Tamps, Santa Engracia, 11/2/1953, J Salazar, colectado por estudiante, Rockefeller Collection, return to Cantrell (1, UMMZ); Nuevo Leon, Monterrey, 12/12/1991, WF Chamberlain, at light (1, TAMU). All paratypes labeled “Paratype *Arenivaga gumperzae* Hopkins 2012” [blue label with black border].

##### Etymology.

The name is a noun in the genitive case. This species is named for the author’s close friend, Linda Gumperz, who lost a short, brave battle with pancreatic cancer four days before the start of the author’s PhD program. The first one is for you Linda, as promised.

##### Distribution.

This species is distributed from Ft. Stockton, Pecos County, Texas in the north, to Pedricena, Durango, Mexico and Linares, Nuevo Leon, Mexico in the south. The western limit is Presidio, Presidio County, Texas and the eastern is Falcon State Recreation Area, Zapata County, Texas. See Fig. 77.

##### Diagnosis.

*Arenivaga gumperzae* may be distinguished by the long posteriorly projecting extension of the medial margin of the right dorsal phallomere, which ends in a two-pronged hook. See Fig. 76.

##### Description.

**Male.** *Measurements*. Holotype TL = 19.0 mm, GW = 8.6 mm, PW = 6.3 mm, PL = 4.2 mm, TL/GW = 2.21, PL/PW = 0.67. Dimensions are average for the genus, approximating those of *Arenivaga erratica*. EW = 0.5 mm; OW = 0.4 mm. In paratypes, no notable variations in dimensions from those of holotype.

*Head*. Two ocelli, large, ovoid and protruding (0.4 × 0.3 mm); vertex dark brown, interocellar space deeply concave, dark brown. Frons concave, cream with pale brown edges and strongly demarcated from interocellar space; anterior frons cream, bulbous; anteclypeus cream and smooth. See Fig. 76d.

*Pronotum*. Pronotum light brown; dorsal surface of pronotum covered in setae which are longer and denser anteriorly; pronotal pattern dark brown “panther face”; with scattered fine dark brown maculae; slight aural lines laterally and slight anterior aura. See Fig. 76c.

*Body*. Wing brace present. Two tarsal claws present. Legs and body pale brown subgenital plate asymmetrical, with rounded apices. See Fig. 76b.

*Forewings*. Wings extended well beyond abdominal apex (~ 50% of wing length); wings in most specimens light brown and opaque with long dark brown lines running apically from humeral angle; with scattered brown maculations. See Fig. 76a.

*Genitalia*. Right dorsal phallomere composed of large bulbous lightly sclerotized hook-shaped lobe, articulated with right ventral phallomere on lateral side. Posterior margin with strongly sclerotized ridge that extends towards posterior abdominal opening and ends in two hooks set at approximately right angles to each other. Small central sclerite with U-shaped structure positioned horizontally and intersecting with ridge of other phallomere. Dorsal arm of U-shaped structure longer than ventral arm, each with pointed apices. Right ventral phallomere extending from articulation to form structure rounded at posterior apex but with corrugations at anterior apical end, with rounded concave arm extending beyond depth of rest of phallomere. Left phallomere unmodified. Genital hook with moderate extension to pointed head and short hook; arm with bend. See Fig. 77.

**Figure 75.**
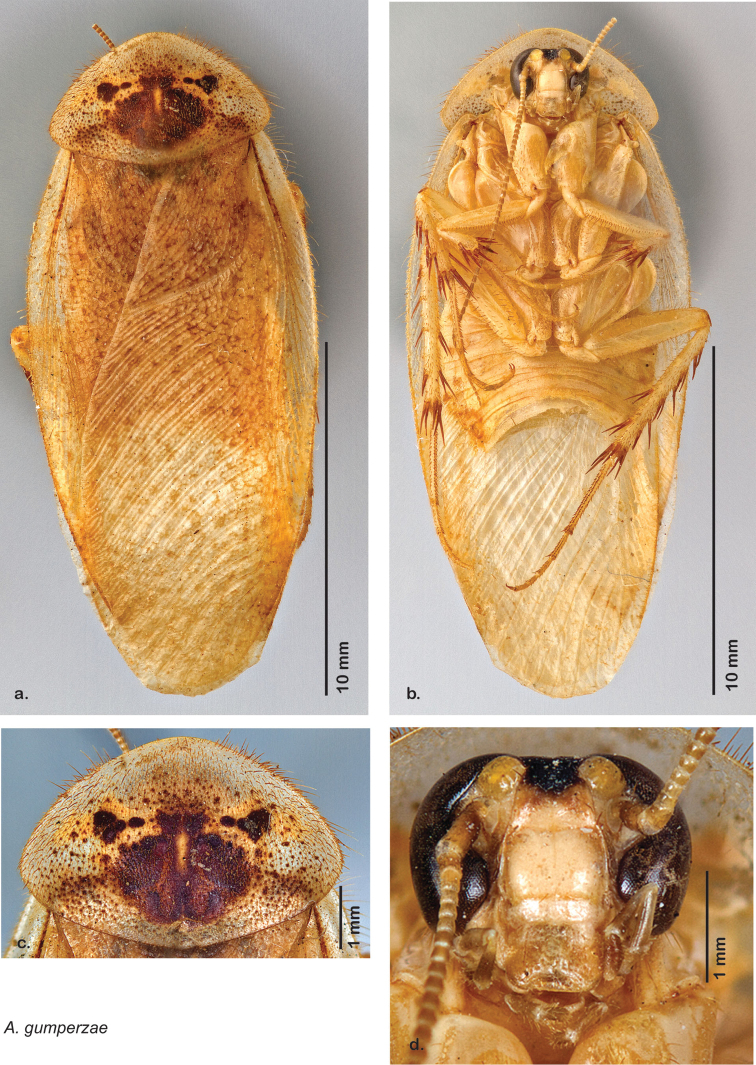
*Arenivaga gumperzae* **a** dorsal habitus **b** ventral habitus **c** pronotum **d** head.

**Figure 76.**
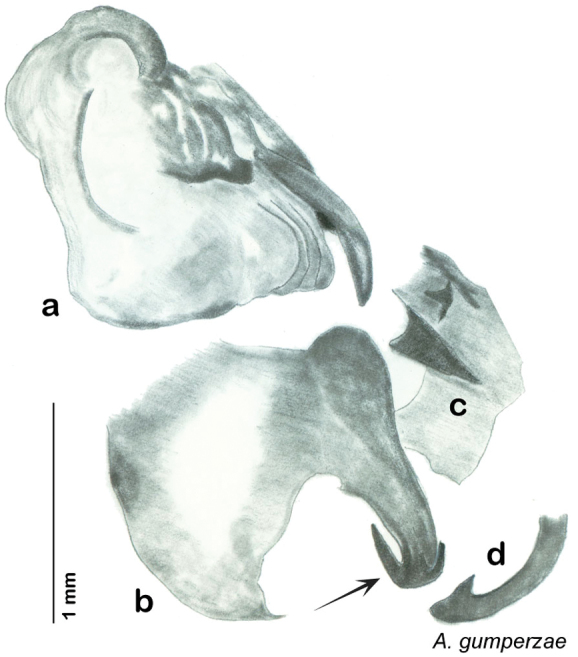
*Arenivaga gumperzae*, genitalia: a) right dorsal phallomere **b** right ventral phallomere **c** small central sclerite **d** genital hook. Arrow(s) indicate diagnostic characters (see text).

**Figure 77.**
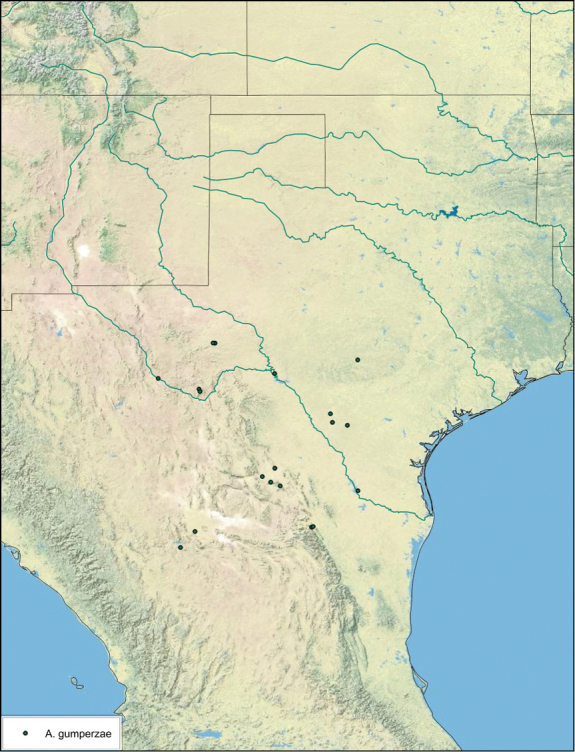
*Arenivaga gumperzae*, distribution.

##### Habitat and natural history.

This species occurs in terrain that is dry, hot, and sandy. All other life history elements remain unobserved.

#### Arenivaga gurneyi sp. n.

http://zoobank.org/FAD72561-AF9D-4101-873D-56DFFE9C98A6

http://species-id.net/wiki/Arenivaga_gurneyi

Figures 78 – 80

##### Type locality.

MEXICO, Michoacan, Acahuato.

##### Material examined.

Holotype: ♂ in USNM labeled “Acahuato, Michoacan, MEXICO, Alt. 3000 ft., August 19, 1941, Coll. H. Hoogstraal” “HOLOTYPE *Arenivaga gurneyi* Hopkins, 2012” [red label with black border].

Paratypes (7): MEXICO: Michoacan, Acahuato, 8/19/1941, 3000 ft., H Hoogstraal (1, USNM); Michoacan, Acahuato, 8/19/1941, 3000 ft., H Hoogstraal, ‘035’ on 2 specimens (3, ANSP); San Jose Purua [locality decision by HHopkins 2012, specimens sent to ANSP], 8/4/1947, Hodge, Data uncertain. “San Jose Purua, Mex., VII-4-1947, Hodge” but may be San Jose de Pimas in Sonora near Hermosillo. Also may belong to ANSP, as I suspect this. 3 males. ABG (3, ANSP). All paratypes labeled “Paratype *Arenivaga gurneyi* Hopkins 2012” [blue label with black border].

##### Etymology.

The name is a noun in the genitive case. This species is named for the late Dr. Ashley B. Gurney, who with Dr. David Nickle was the last to work on revising *Arenivaga*.

##### Distribution.

This species is found in the state of Michoacan, Mexico. See Fig. 80.

##### Diagnosis.

*Arenivaga gurneyi* may be distinguished by the very short hook-shaped lobe on the right dorsal phallomere and the curious shape of the medial margin on same. See Fig. 79.

##### Description.

**Male.** *Measurements*. Holotype TL = 19.2 mm, GW = 9.8 mm, PW = 6.79 mm, PL = 4.43 mm, TL/GW = 1.96, PL/PW = 0.65. EW = 0.25 mm; OW = 0.50 mm. Among paratypes range of TL 18.0–21.6 mm; range of GW 8.7–10.3 mm; range of PW 6.30–7.73 mm; range of PL 4.20–5.03 mm.

**Figure 78.**
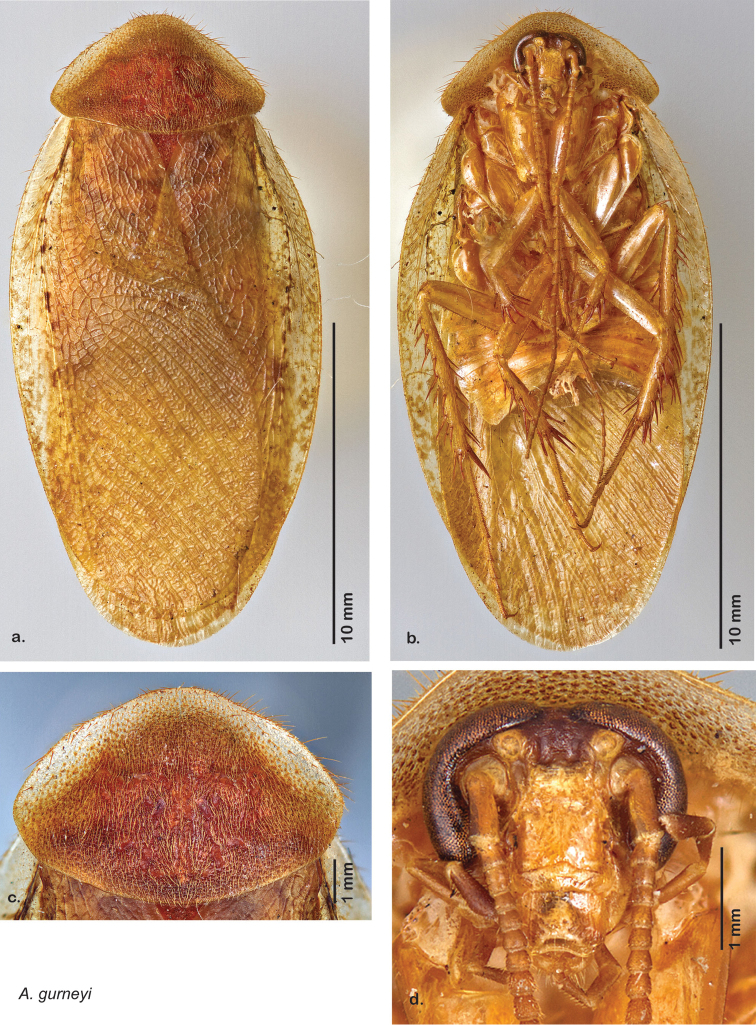
*Arenivaga gurneyi* **a** dorsal habitus **b** ventral habitus **c** pronotum **d** head.

*Head*. Two ocelli, somewhat smaller and rounder than usual, ovoid and protruding (0.35 × 0.30 mm); vertex medium brown, with small ridges between apices of eyes and extending onto ocellar tubercles; interocellar space concave, medium brown. Frons light brown; posterior tectiform horizontally with fine corrugations. Anterior frons slightly bulbous, light brown; light brown anteclypeus. See Fig. 78d.

*Pronotum*. Pronotum translucent waxy beige; dorsal surface of pronotum with orange-brown setae, dense in some specimens; pronotal pattern impressed, medium orange-brown, with lighter or much darker orange-brown aura depending on specimen; area of pattern too dark and often too setose to discern detail. See Fig. 78c.

*Body*. Wing brace absent. Two tarsal claws present. Legs and body medium orange-brown; subgenital plate orange-brown; asymmetrical with rounded apices. See Fig. 78b.

*Forewings*. Wings extended beyond abdominal apex (up to 30% of total wing length); blotchy medium to dark brown; surface opaque and matte. See Fig. 78a.

**Figure 79.**
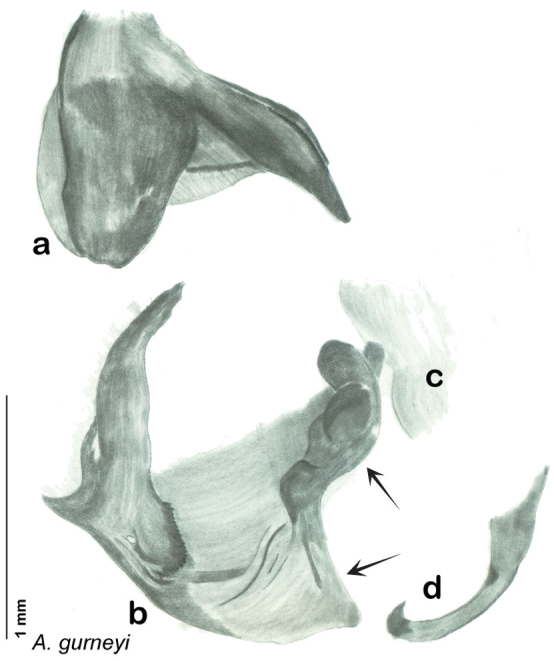
*Arenivaga gurneyi*, genitalia: a) right dorsal phallomere **b** right ventral phallomere **c** small central sclerite **d** genital hook. Arrow(s) indicate diagnostic characters (see text).

*Genitalia*. Right dorsal phallomere composed of lightly sclerotized cup, articulated with right ventral phallomere on lateral side; posterior projecting bulbous lobe entirely absent; medial edge with small smooth projection posteriorly, becoming more heavily sclerotized and punctate anteriorly, making right angle extending medially, ending in shagreened knob. Small central sclerite lightly sclerotized, finely punctate, existing only as flap attached to dorsal side of right angle and knob of dorsal phallomere. Articulation between right phallomeres deep, concave and setose, with toothed border adjacent to dorsal phallomere posterior end of which projects out to point. Right ventral phallomere consists of large punctate medially projecting lobe with central indentation; anteriorly is attached large shagreened anteriorly oriented pointed lobe. Folded anterior portion of left phallomere wide, setose, enclosed at both ends, otherwise unmodified. Genital hook widely curving to short point; arm slender. See Fig. 79.

**Figure 80.**
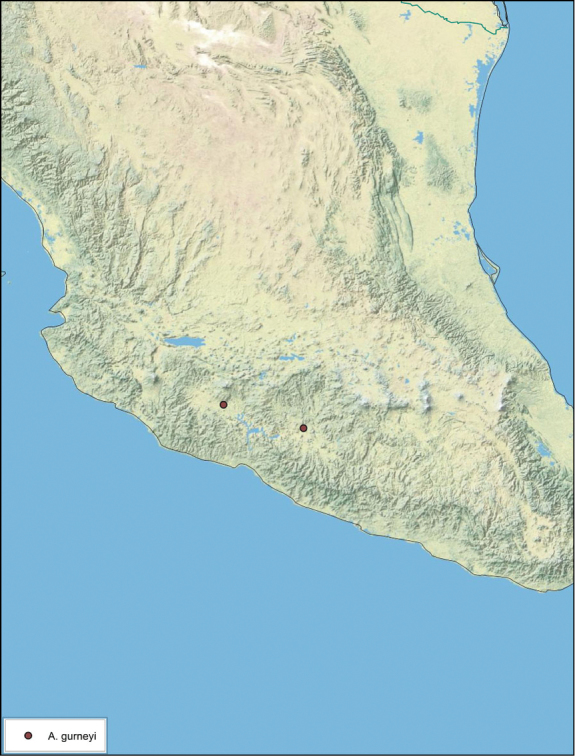
*Arenivaga gurneyi*, distribution.

##### Habitat and natural history.

All life history elements remain unobserved.

#### Arenivaga haringtoni sp. n.

http://zoobank.org/F4319ADE-81E1-462D-A5BB-0D68C2D6BE00

http://species-id.net/wiki/Arenivaga_haringtoni

Figures 81 – 83

##### Type locality.

USA, Arizona, Mohave Co., near Kingman.

##### Material examined.

Holotype: ♂ in NVDA labeled “2 mi NE Gold Butte, NV, Clark Co., VI-16-1988, R.C. Bechtel, J.L. Carpenter,. J.B. Knight Collectors, Black Light Trap” “HOLOTYPE *Arenivaga haringtoni* Hopkins, 2012” [red label with black border].

Paratypes (6): USA: AZ, Yuma Co., Alamo Crossing, 9/7/1959, CE Benson (1, UAIC); AZ, Mohave Co., near Kingman, 7/9/1920, OC Poling, A.erratica (Rehn) det. Hebard 1932 (1, ANSP); AZ, Yuma Co., (now La Paz Co.), Alamo Crossing, 9/7/193?, C.E.Benson (1, UAIC); NV, Clark Co., 2 mi NE Gold Butte, 7/14/1977, RC Bechtel, JB Knight & DF Zoller Black Light Trap (2, NVDA); NV, Clark Co., Cedar Basin, 7/22/1976, 4400 ft., RC Bechtel, JB Knight & DF Zoller, Black Light Trap (1, NVDA). All paratypes labeled “Paratype *Arenivaga haringtoni* Hopkins 2012” [blue label with black border].

##### Etymology.

The name is a noun in the genitive case. This species is named in honor of Donald Harington, author of “The Cockroaches of Stay More”, a priceless novel about wonderful animals.

##### Distribution.

This species is found in west central Arizona and southeastern Nevada. See Fig. 83.

##### Diagnosis.

*Arenivaga haringtoni* may be distinguished by its dark brown color as it is the only species of its color found in its range. If locality information is not available it may be distinguished by the long medial margin on the right dorsal phallomere, which extends posteriorly some distance beyond the rest of the phallomere. See Fig. 82.

##### Description.

**Male.** *Measurements*. Holotype TL = 17.6 mm, GW = 8.7 mm, PW = 5.82 mm, PL = 4.04 mm, TL/GW = 2.02, PL/PW = 0.69. EW = 0.65 mm; OW = 0.45 mm. Among paratypes range of TL 16.9–18.3 mm; range of GW 7.6–10.7 mm; range of PW 5.33–6.28 mm; range of PL 4.04–4.45 mm.

**Figure 81.**
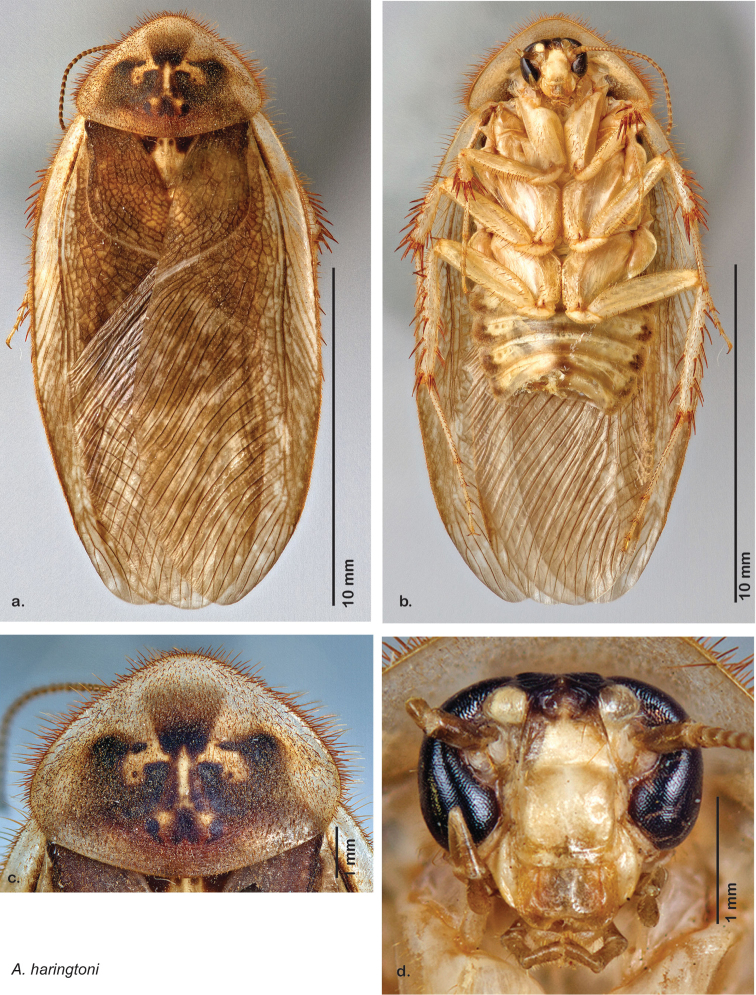
*Arenivaga haringtoni* **a** dorsal habitus **b** ventral habitus **c** pronotum **d** head.

*Head*. Two ocelli large, ovoid and protruding (0.35 × 0.25 mm); vertex dark brown with small ridges in rays around upper apices of eyes and extending onto ocellar tubercles; pale medial line; interocellar space concave, dark brown, light brown medially, with two oval indentations. Frons translucent beige, posterior concave; anterior portion of frons bulbous and translucent beige; translucent beige smooth anteclypeus. See Fig. 81d.

*Pronotum*. Pronotum translucent, waxy beige; dorsal surface of pronotum with short orange-brown setae that are thicker and longer laterally; pronotal pattern dark brown to red-brown “panther face”, with some discernible detail and extensive aura, generally on all sides. See Fig. 81c.

*Body*. Wing brace present. Two tarsal claws present. Legs and body light brown; sternites medium brown laterally; subgenital plate light brown with darker posterior edge; asymmetrical with rounded apices. See Fig. 81b.

*Forewings*. Wings extended well beyond abdominal apex (~40% of wing length); color varies from uniform medium brown to blotchy medium brown; surface may be matte and opaque or translucent and lustrous. See Fig. 81a.

**Figure 82.**
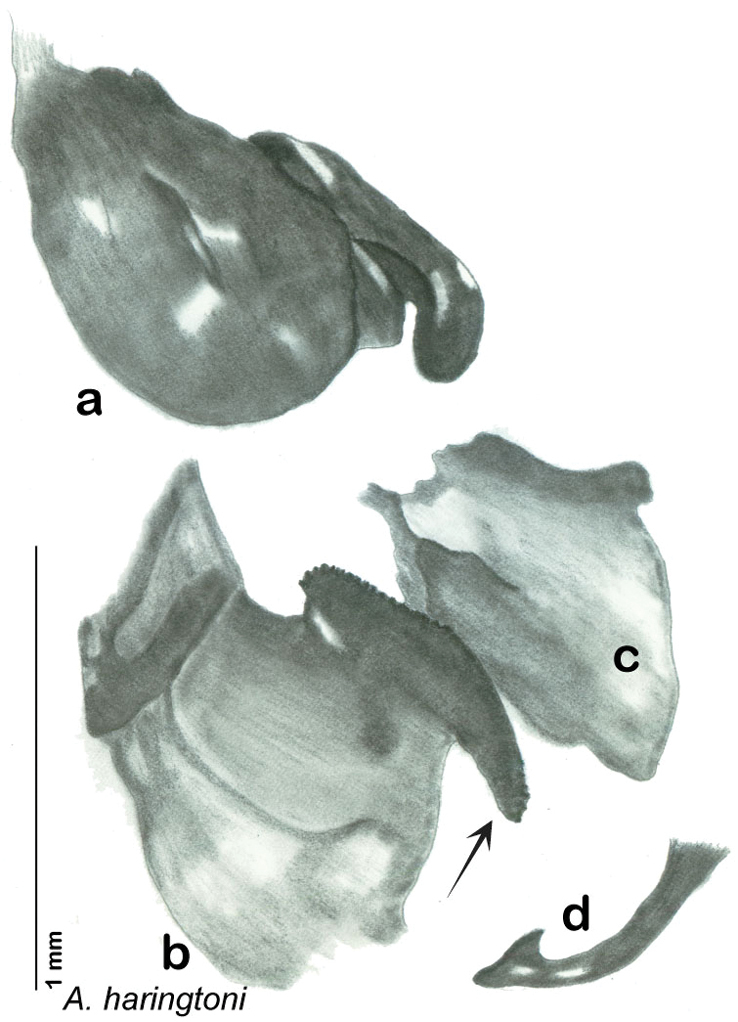
*Arenivaga haringtoni*, genitalia: a) right dorsal phallomere **b** right ventral phallomere **c** small central sclerite **d** genital hook. Arrow(s) indicate diagnostic characters (see text).

*Genitalia*. Right dorsal phallomere composed of lightly sclerotized bulbous lobe, articulated with right ventral phallomere on lateral side; central field lightly sclerotized; medial margin more heavily sclerotized, shagreened with rough edge, very slightly arcuate centrally. Small central sclerite nearly flat, nondescript in shape, finely punctate, rugose anteriorly; right ventral phallomere extends from articulation to form smooth rounded structure becoming punctate and corrugated anteriorly; attached anteriorly is mildly dorsally projecting flanged concave punctate arm that extends beyond depth of rest of phallomere, exterior surface shagreened. Folded anterior portion of left phallomere setose and punctate, otherwise unmodified. Genital hook with long extension to pointed head, and slight indentation on moderate hook; distinct bend in arm. See Fig. 82.

**Figure 83.**
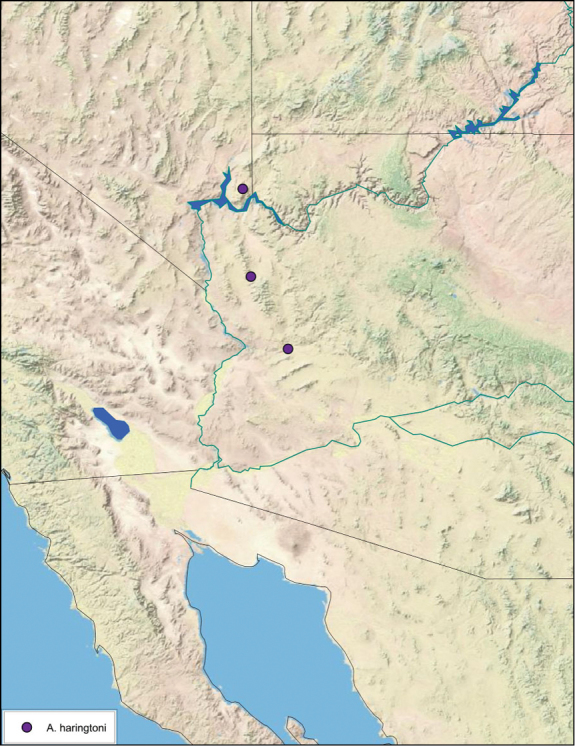
*Arenivaga haringtoni*, distribution.

##### Habitat and natural history.

All life history elements remain unobserved.

#### Arenivaga hebardi sp. n.

http://zoobank.org/5107CE7A-2609-4690-92B8-A16DB52E787B

http://species-id.net/wiki/Arenivaga_hebardi

Figures 84 – 86

##### Type locality.

MEXICO, Sonora, Ciudad Obregon.

##### Material examined.

Holotype: ♂ in UAIC labeled “CD. OBREGON, SON. MEXICO, 9-VIII-1960, at light, Wm. W. Gibson Collector” “HOLOTYPE *Arenivaga hebardi* Hopkins, 2012” [red label with black border].

Paratypes (3): MEXICO: Sonora, Obregon, 7/29/1952, C & P Vaurie (2, AMNH); Sonora, Ciudad Obregon, 8/9/1960, WW Gibson, at light (1, UAIC). All paratypes labeled “Paratype *Arenivaga hebardi* Hopkins 2012” [blue label with black border].

##### Etymology.

The name is a noun in the genitive case. This species is named for the great Orthoptera researcher of the early 20^th^ century and last reviser of this genus, Morgan Hebard.

##### Distribution.

This species is found in and around Ciudad Obregon, Sonora, Mexico. See Fig. 86.

##### Diagnosis.

*Arenivaga hebardi* may be distinguished by the robust double hook at the posterior and of the medial margin of the right dorsal phallomere. See Fig. 85.

##### Description.

**Male.** *Measurements*. Holotype TL = 17.7 mm, GW = 8.4 mm, PW = 5.94 mm, PL = 4.22 mm, TL/GW = 2.11, PL/PW = 0.71. EW = 0.15 mm; OW = 0.30 mm. No notable size variation among paratypes.

**Figure 84.**
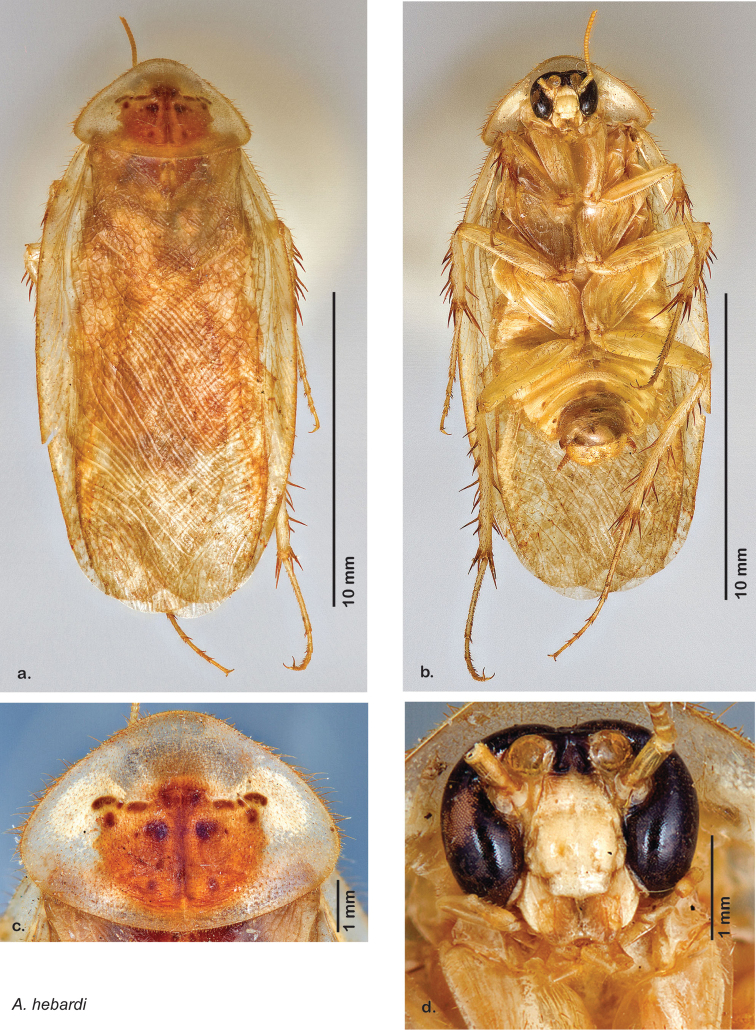
*Arenivaga hebardi* **a** dorsal habitus **b** ventral habitus **c** pronotum **d** head.

*Head*. Two ocelli large, ovoid and protruding (0.50 × 0.40 mm); vertex medium brown, with small ridges between apices of eyes and extending onto ocellar tubercles; interocellar space concave, medium brown, with arrowhead-shaped indentation. Frons light brown; posterior concave, with shallow horizontal corrugations; anterior portion of frons bulbous, light brown; wide light brown anteclypeus with medial point. See Fig. 84d.

*Pronotum*. Pronotum translucent waxy beige; dorsal surface of pronotum with dense very short light orange-brown setae; pronotal pattern medium orange-brown “hippo face” with some discernible detail; no aura. See Fig. 84c.

*Body*. Wing brace present. Two tarsal claws present. Legs and body light orange-brown; subgenital plate light orange-brown with darker border; asymmetrical with rounded apices. See Fig. 84b.

*Forewings*. Wings extended well beyond abdominal apex (up to ~30% of wing length); blotchy medium orange-brown; surface matte and opaque. See Fig. 84a.

**Figure 85.**
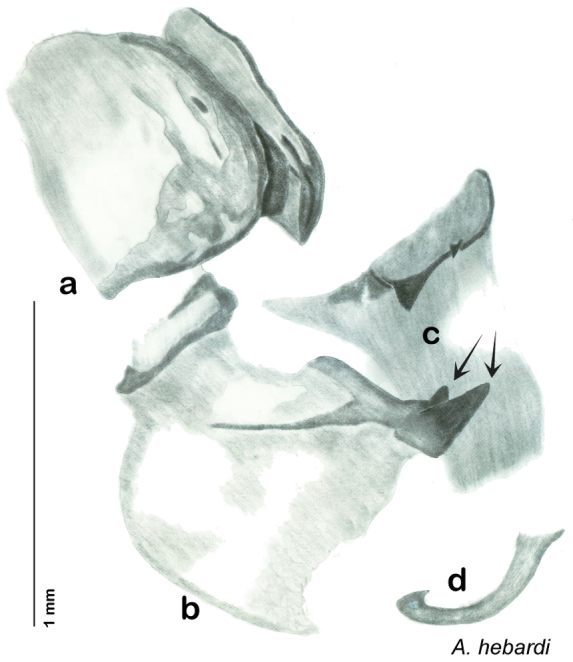
*Arenivaga hebardi*, genitalia: a) right dorsal phallomere **b** right ventral phallomere **c** small central sclerite **d** genital hook. Arrow(s) indicate diagnostic characters (see text).

*Genitalia*. Right dorsal phallomere composed of lightly sclerotized, broad bulbous lobe, articulated with right ventral phallomere on lateral side; central field lightly sclerotized, slightly cupped; medial edge more sclerotized, smooth, with robust posteriorly projecting spine and immediately adjacent medially projecting smaller spine. Small central sclerite lightly sclerotized, finely punctate, concave, anterior end with smooth posteriorly projecting ridge; smooth flat point at dorsal end of ridge with one or two very small spines along rest of ridge. Right ventral phallomere arises from articulation to form large punctate rounded lobe, becoming more sclerotized, corrugated, shagreened and narrow anteriorly; small shagreened fold in moderate gap followed by wide dorsally flanged, concave arm, smooth to punctate, extending to slightly greater depth than rest of phallomere. Folded anterior portion of left phallomere narrow and setose, otherwise unmodified. Genital hook with short extension to pointed head; short hook; arm delicate with distinct bend. See Fig. 85.

**Figure 86.**
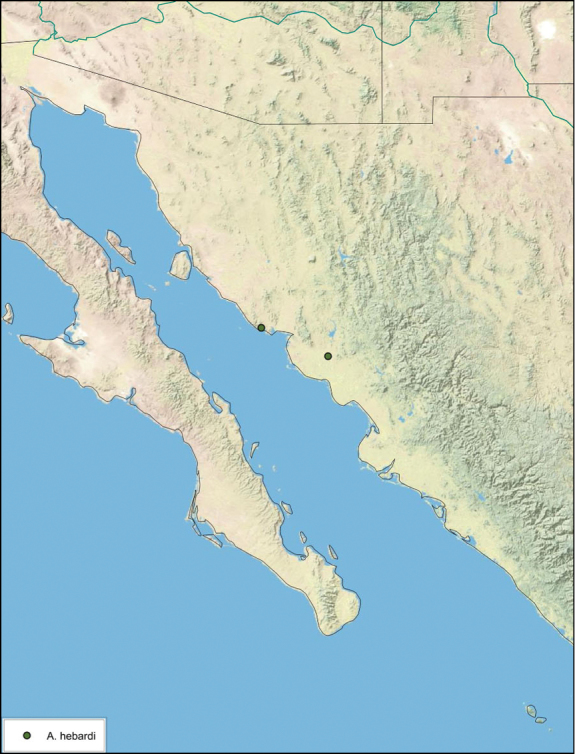
*Arenivaga hebardi*, distribution.

##### Habitat and natural history.

All life history elements remain unobserved.

#### Arenivaga hopkinsorum sp. n.

http://zoobank.org/12BF5D0A-3635-40A2-86DA-F80F3CD49889

http://species-id.net/wiki/Arenivaga_hopkinsorum

Figures 87 – 89

##### Type locality.

USA, Arizona, Santa Cruz Co., Nogales.

##### Material examined.

Holotype: ♂ in USNM labeled “Nogales, Ariz., July 9, 1965, J.E.Mills” “HOLOTYPE *Arenivaga hopkinsorum* Hopkins, 2012” [red label with black border].

Paratypes (319): USA: AZ, Pima Co., Brown Canyon, Baboquivari Mts., 9/6/1953, L Martin (3, LACM); AZ, Santa Cruz Co., Nogales, 7/15/1952, DJ & JN Knull (7, OSUC); AZ, Santa Cruz Co., Nogales, 7/7/1949, DJ & JN Knull (6, OSUC); AZ, Santa Cruz Co., Nogales, 7/7/1949, DJ & JN Knull (1, FSCA); AZ, Santa Cruz Co., Nogales, 6/7/1961, JM Kaiser, at lights (2, USNM); AZ, Santa Cruz Co., Nogales, 7/8/1961, FA Allen, 61-19807 (1, USNM); AZ, Santa Cruz Co., Nogales, 6/30/1903, Oslar, Homoeogamia apache Sauss, 8 (1, USNM); AZ, Santa Cruz Co., Patagonia, 8/7/1940, at lights (1,UCRC); AZ, Santa Cruz Co., Nogales, 6/15/1967, R Rice, under metal sheet (19, UAIC); AZ, Santa Cruz Co., Nogales, 12/3/1966, R Rice, under metal sheet (2, UAIC); AZ, Santa Cruz Co., Baboquivari Mts., 8/3/1967, Hessel & Ritchie, 46001 (1, UAIC); AZ, Pima Co., Brown Canyon, Baboquivari Mts., 8/4/1962, Werner & Johnson, UV Light trap (3, UAIC); AZ, Pima Co., Tucson, 4/3/1959, RS Beal (1, ASUT); AZ, Pima Co., Organ Pipe Cactus NM, 4/3/1966, CW Obrien, Dripping Springs at night (1, FSCA); AZ, Pima Co., Baboquivari Mts., 8/4/1966, 31.43N, 111.37W, 3250 ft., Werner family, Chutum Vaya Canyon, W. slope (2, UAIC); AZ, Pima Co., Baboquivari Mts., 8/8/1988, Werner & Olson, Solano Canyon, UV (1, UAIC); AZ, Pinal Co., Jct. Hwy. 84 & I8, 7/23/2000, WB Warner, lights (1, WB Warner); AZ, Santa Cruz Co., Pena Blanca Lake, 7/9/1994, M Siner, *Arenivaga* sp. Det. B. Mathison 1994 (1, UAIC); AZ, Pima Co., Kitt Peak, 6/17/1990, 1370-2130 m, DB & BI Weissman #90-52 (2, CAS); AZ, Pima Co., Brown Canyon, Baboquivari Mts., 6/27-28/1957, at lights (2, UAIC); AZ, Pima Co., Baboquivari Mts., 4/28/1938, JA Comstock (1, LACM); AZ, Santa Cruz Co., Nogales, 7/25/1903, Oslar (1, LACM); AZ, Pima Co., Sabino Canyon, 8/12/1932, RH Beamer *Arenivaga apacha* (Sauss.) Det. Hebard 1935 (1, SEMC); AZ, Santa Cruz Co., Tumacacori Pk., Tumacacori Mts., 7/28/1948, WL Nutting, chaparral area, WLN (1, UAIC); AZ, Pima Co., Brown Canyon, Baboquivari Mts., 7/27/1948, 3800 ft., Nutting & Werner, at lights, sycamore-oak-mesquite, WLN (1, UAIC); AZ, Pima Co., Baboquivari Mts., FH Snow, Homoeogamia erratica Rehn (1, CSCA); AZ, Pima Co., Baboquivari Mts., 7/17/1950, RH & LD Beamer (7, SEMC); AZ, Pima Co., Brown Canyon, Baboquivari Mts., 6/8/1952, Cazier, Gertsch & Schrammel (14, AMNH); AZ, Santa Cruz Co., Nogales, 4 mi. N of Jct. Hwy.289 & I9, 7/27/1977, JD Pinto, Black light (2, UCRC); AZ, Santa Cruz Co., 12 mi. E of Nogales, 9/1/1969, PN Jump (1, LACM); AZ, Santa Cruz Co., Rio Rico, 8/5/1995, BC & WB Warner (1, WB Warner); AZ, Pima Co., Sycamore Canyon, Baboquivari Mts., 10/6-9/1910, 3700 ft., *Arenivaga apacha* (Sauss.) Hebard Collection (1, ANSP); AZ, Pima Co., Kitt Peak, Baboquivari Mts., 8/1-4/1916, 31.57N, 111.33W, 4050 ft., *Arenivaga apacha* (Sauss.) Det. Hebard 1917, 1 specimen-figured 1920 (3, ANSP); AZ, Santa Cruz Co., Patagonia Mts., Solano Canyon, 8/8/1988, Werner, UV (2, UAIC); AZ, Pima Co., near Kitt Peak, Baboquivari Mts., 9/7-9/1916, 32.0N, 111.36W, 3600 ft. (1, ANSP); AZ, Pima Co., Baboquivari Mts., 4/28/1925, 4500 ft., AA Nichol (1, ANSP); AZ, Santa Cruz Co., Pena Blanca Lake, 7/20/1972, 3950 ft., B Harding (2, LACM); AZ, Santa Cruz Co., Washington Mts., Nogales, 7/15/1920 (1, ANSP); AZ, Santa Cruz Co., Nogales, 6/30/1903 (1, ANSP); AZ, Pinal Co., I10 rest stop 33 mi. SE of Phoenix, 7/16/2009, W Warner, Na Lights (1, WB Warner); AZ, Pima Co., Brown Canyon, Baboquivari Mts., 7/9/1959, V Roth (1, UAIC); AZ, Pima Co., Brown Canyon, Baboquivari Mts., 6/1-15/1923, 5000 ft., O Poling (9, ANSP); AZ, Santa Cruz Co., W.slope Patagonia Mts. on Lochiel Rd., 7/28/1958, 5330 ft., W Nutting, mesquite-chaparral, WLN, 1 specimen-*Arenivaga apacha* (Sauss.) det.WL Nutting 1950 (2, UAIC); AZ, Santa Cruz Co., Cayetano Mts. near Calabasas, 2/16/1919, 3800 ft., RD Camp 2122, *Arenivaga apacha* (Saussure) 1920 det. TH Hubbell (1, UMMZ); AZ, Pima Co., Baboquivari Mts., 5/1-15/1924, OC Poling, *Arenivaga apacha* (Saussure) det. TH Hubbell 1932 (1, UMMZ); AZ, Santa Cruz Co., Nogales, 11/5/1966, R Rice, under metal sheet (1, UAIC); AZ, Santa Cruz Co., Nogales, 4/16/1966, R Rice, under metal sheet (2, UAIC); AZ, Pima Co., Baboquivari Mts., FH Snow, 1 specimen-*Arenivaga apacha* (Sauss.) det. Hebard 1926 (2,ANSP); AZ, Pima Co., Allison Dam, Fresnal Mts., Baboquivari Mts., 4/22/1923, O Poling (5, ANSP); AZ, Pima Co., Brown Canyon, Baboquivari Mts., 4/15-30/1923, 5000 ft., O Poling (10, ANSP); AZ, Santa Rita Mts., 6/12/1936, RA Flock (1, ANSP); AZ, Patagonia, 7/29/1941, RH Beamer (1, ANSP); AZ, Santa Cruz Co., Tumacacori Mts., Yanks Spring, Sycamore Canyon, 8/3/1952, Leech & Green (1, CAS); AZ, Santa Cruz Co., Patagonia Mts., 2.5 mi. W of Harshaw, 8/2/1952, Leech & Green (1, CAS); AZ, Santa Cruz, Patagonia on Sonoita Cr., 10/14/1927, JA Kusche (1, CAS); AZ, Santa Cruz Co., Washington Mts., Nogales, 7/15/1920, JA Kusche, 1 specimen-*Arenivaga apacha* (Sauss.) det. Hebard 1924 (3, CAS); AZ, Pima Co., Brown Canyon, Baboquivari Mts., 7/29-30/1952, Leech & Green (3, CAS); AZ, Pima Co., W. side of Baboquivari Canyon, Baboquivari Mts., 7/25-27/1952, Leech & Green (3, CAS); AZ, Cochise Co., Chiricahua Mts., Cave Creek, 7/?/1927, 6-9800 ft., JA Kusche (1, CAS); AZ, Huachuca Mts., 8/20/1903, Oslar, Univ. of Kan. Lot 968 (2, ANSP); AZ, Santa Cruz Co., Nogales, 6/28/1957, Spitzer, 57 10547 (1, USNM); AZ, Santa Cruz Co., Nogales, 7/10/1947, Byers, 47 10508 (1, USNM); AZ, Santa Cruz Co., Nogales, 6/17/1903, Oslar (1, USNM); AZ, Santa Cruz Co., Nogales, 8/23/1903, Oslar (1, USNM); AZ, Pima Co., Baboquivari Mts., 9/1-15/1923, 5000 ft., OC Poling, 13 specimens-*Arenivaga apacha* (Saussure) det. TH Hubbell 1932 (14, UMMZ); AZ, Pima Co., Baboquivari Mts., 5/15-30/1924, 4000 ft., OC Poling, 4 specimens-*Arenivaga apacha* (Saussure) det. TH Hubbell 1931/2 (35, UMMZ); AZ, Pima Co., Baboquivari Mts., 5/1-15/1924, OC Poling, 14 specimens-*Arenivaga apacha* (Saussure) det. TH Hubbell 1932 (15, UMMZ); AZ, Pima Co., Baboquivari Mts., 6/15-30/1924, 4000 ft., OC Poling, *Arenivaga apacha* (Saussure) det. TH Hubbell 1932 (4, UMMZ); AZ, Pima Co., Baboquivari Mts., 9/?/1924, 4000 ft., OC Poling, 2 specimens-*Arenivaga apacha* (Saussure) det. TH Hubbell 1932 (3, UMMZ); AZ, Pima Co., Baboquivari Mts., 7/1-15/1924, 4000 ft., OC Poling (1, UMMZ); AZ, Pima Co., Baboquivari Mts., 11/1-15/1923, OC Poling, *Arenivaga apacha* (Saussure) det. TH Hubbell 1932 (1, UMMZ); AZ, Pima Co., Baboquivari Mts., 10/?/1924, 4-5000 ft., OC Poling (1, UMMZ); AZ, Pima Co., Baboquivari Mts., 6/1-15/1924, OC Poling (1, UMMZ); AZ, Pima Co., Baboquivari Mts., 9/15-30/1923, OC Poling, *Arenivaga apacha* (Saussure) det. TH Hubbell 1932 (2, UMMZ); AZ, Santa Cruz Co., Pena Blanca, Oro Blanco Mts., 5/27/1963, LM Martin (1, LACM); AZ, Santa Cruz Co., Pena Blanca, Oro Blanco Mts., 8/2/1960; LM Martin (1, LACM); AZ, Pima Co., Baboquivari Mts., 4/24/1938, JA Comstock (1, LACM); AZ, Pima Co., Ajo, 3/25/1923, O Poling (1, ANSP); CA, San Bernardino, dry bed Mojave R. 6 mi. E of Yermo, 8/27/1952, Leech & Green (1, CAS); MEXICO: Sonora, Hermosillo, 6/21/1957, Chemsak & Rannells, at light (8, EMEC); Sonora, San Carlos, Caracol Penn., 5/19/2003, 27.57N, 111.03W, SIB 2003.0000, blue tag 9000 (2, UAIC); Sonora, NW San Carlos near Rancho Palo Fiero, 5/20/2003, 27.58N, 111.05W, SIB2003.0010, blue tag 9000 (2, UAIC); Sonora, Aconchi Rio Sonora, dry wash, 6/12/1982, Olson, Thomas & Burne (2, UAIC); Sonora, Hermosillo, 7/9-16/1953, B Malkin (1, CAS); Sonora, 5 mi. S of Santa Ana, 7/10/1970, B & R Harding (2, LACM); Sonora, Pitiquito, 7/4/1952, C & P Vaurie (1, ANSP); Sonora, 5 mi. NE of Magdalene, 9/27/1953, ER Tinkham (1, USNM); Sonora, Desemboque, 8/1-15/1953, B Malkin (39, CAS); Sonora, Desemboque, 7/17-31/1953, B Malkin (4, CAS); Sonora, Desemboque, 9/1-10/1953, B Malkin (5, CAS); Sonora, Desemboque, 8/20-31/1953, B Malkin (2, CAS); Sonora, Tiburon Island (north end), 7/10/1952, C & P Vaurie (2, AMNH); Sonora, Tiburon Island (south end), 7/13/1952, C & P Vaurie (3, AMNH); Sonora, Puerto Libertad, 2/3/1935, N Bloomfield (1, SDMC). All paratypes labeled “Paratype *Arenivaga hopkinsorum* Hopkins 2012” [blue label with black border].

##### Etymology.

The name is a noun in the genitive case. This species is named for my parents, Richard and Alberta Hopkins, with deep gratitude for an extraordinary childhood; and all the encouragement since.

##### Distribution.

This species is found in northwestern Sonora, Mexico and southeastern and south central Arizona. The lone specimen from southern California is believed to have an incorrect label or carried there by an outside agent. See Fig. 89.

##### Diagnosis.

*Arenivaga hopkinsorum* may be confused with *Arenivaga adamsi* but can be distinguished by the presence of two large spines (and occasionally a small third spine) on the medial margin of the right dorsal phallomere. See Figs 88 and 13.

##### Description.

**Male.** *Measurements*. Holotype TL = 18.3 mm, GW = 9.2 mm, PW = 6.10 mm, PL = 4.07 mm, TL/GW = 1.99, PL/PW = 0.67. EW = 0.30 mm; OW = 0.40 mm. Among paratypes range of TL 15.5–22.3 mm; range of GW 7.6–10.3 mm; range of PW 5.25–6.63 mm; range of PL 3.85–4.49 mm.

**Figure 87.**
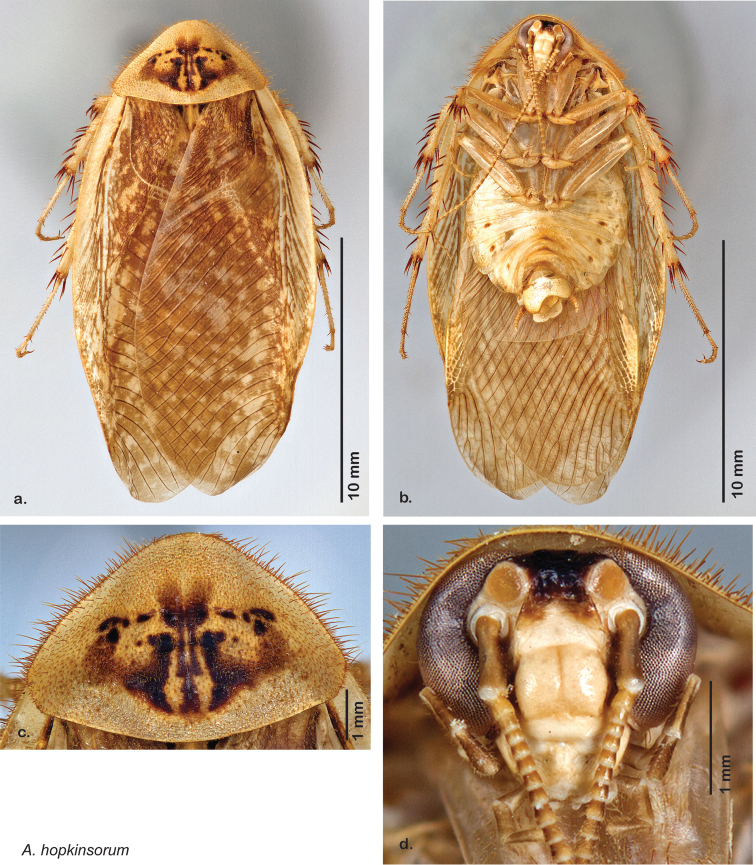
*Arenivaga hopkinsorum* **a** dorsal habitus **b** ventral habitus **c** pronotum **d** head.

*Head*. Two ocelli large, ovoid and protruding (0.40 × 0.30 mm); vertex dark brown with small ridges in rays around upper apex of eyes and extending onto ocellar tubercles; interocellar space concave, dark brown, lighter brown anteriorly, with horizontal indentation. Frons waxy white, posterior concave; anterior portion of frons bulbous and waxy white; waxy white smooth anteclypeus. See Fig. 87d.

*Pronotum*. Pronotum opaque, light brown; dorsal surface of pronotum with short orange-brown setae that are thicker and longer laterally; pronotal pattern dark brown “panther face”, in some specimens so dark as to obscure detail; slight lateral and anterior aura. See Fig. 87c.

*Body*. Wing brace present. Two tarsal claws present. Legs and body light brown; sternites of many specimens with fine brown line and brown maculations laterally; subgenital plate light brown; asymmetrical with particularly deep central emargination and angular apices. See Fig. 87b.

*Forewings*. Wings extended well beyond abdominal apex (up to 50% of wing length); blotchy medium to dark brown depending on specimen; surface matte and opaque. See Fig. 87a.

**Figure 88.**
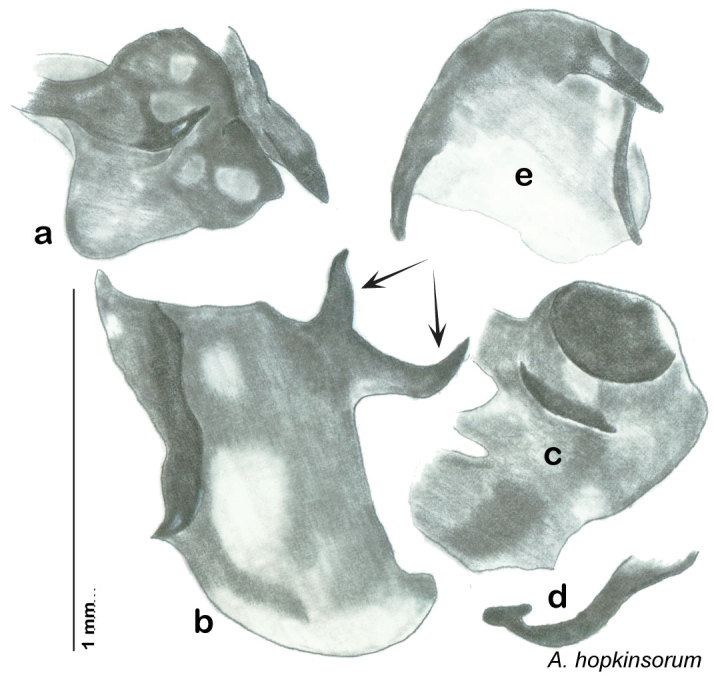
*Arenivaga hopkinsorum*, genitalia: a) right dorsal phallomere **b** right ventral phallomere **c** small central sclerite **d** genital hook **e** left phallomere. Arrow(s) indicate diagnostic characters (see text).

*Genitalia*. Right dorsal phallomere composed of lightly sclerotized bulbous hook-shaped lobe, articulated with right ventral phallomere on lateral side; central field lightly sclerotized; medial margin more heavily sclerotized, narrow with two prominent spines. Small central sclerite deeply concave, punctate, with two sclerotized, punctate lobes on ventral wall; right ventral phallomere extends from articulation to form smooth rounded structure, punctate, with prominent medially projecting spine located posteriorly; attached anteriorly and at an acute angle is mildly dorsally projecting narrowly flanged concave punctate arm that extends beyond depth of rest of phallomere. Folded anterior portion of left phallomere dramatically modified with sclerotized anterior wall and posteriorly projecting spine located ventrally. Genital hook with moderate extension to pointed head with short hook; arm smoothly curving. See Fig. 88.

**Figure 89.**
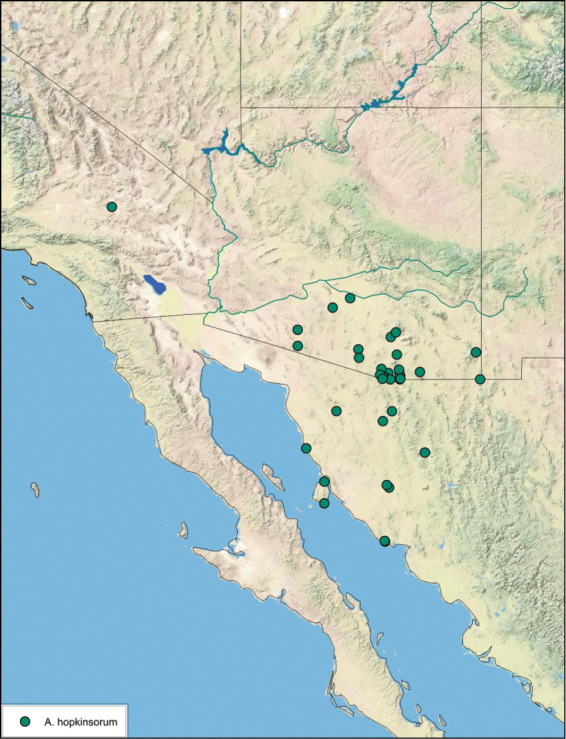
*Arenivaga hopkinsorum*, distribution.

##### Habitat and natural history.

All life history elements remain unobserved.

#### Arenivaga hypogaios sp. n.

http://zoobank.org/EBD47441-6E8C-438D-B164-CA6EC82E1D4E

http://species-id.net/wiki/Arenivaga_hypogaios

Figures 90 – 92

##### Type locality.

MEXICO, Coahuila, 15 mi N San Pedro de las Colonias.

##### Material examined.

Holotype: ♂ in USNM labeled “MEX: Coahuila 15 miles N of San Pedro de las Colonias, 2 Jul 1959, E.R.T.” “HOLOTYPE *Arenivaga hypogaios* Hopkins, 2012” [red label with black border].

Paratypes (12): MEXICO: San Luis Potosi, Nunez 22 mi. NE of Villa Hidalgo, 8/27/1959, 4900 ft., Cohn & Cantrall, #37 (1, UMMZ); Coahuila, 15 mi. N of San Pedro de Las Cobrias, 7/2/1959, ERT (2, USNM). USA: TX, Terrel Co., Lozier Canyon, 7/8/1948, WL Nutting, *Arenivaga* sp. near *rehni*?, Det. WL Nutting 1950 (2, USNM). All paratypes labeled “Paratype *Arenivaga hypogaios* Hopkins 2012” [blue label with black border].

##### Etymology.

The name is a noun in the nominative singular. This species is named from the Greek meaning underground, in recognition of its subterranean life.

##### Distribution.

This species is found from the central Texas-Mexico border south through central Mexico including the states of Coahuila and San Luis Potosi. See Fig. 92.

##### Diagnosis.

*Arenivaga hypogaios* may be confused with *Arenivaga florilega* and *Arenivaga galeana* but may be distinguished by the difference in genital hooks, and the narrower anterior arm in *Arenivaga hypogaios* on the right ventral phallomere. *Arenivaga hypogaios* also has a ridge of serrations on the lateral side of the open field of the right dorsal phallomere which is not present in *florilega* or *galeana*. See Figs 91, 58 and 64.

##### Description.

**Male.** *Measurements*. Holotype TL = 23.0 mm, GW = 9.7 mm, PW = 6.92 mm, PL = 4.82 mm, TL/GW = 2.37, PL/PW = 0.70. EW = 0.20 mm; OW = 0.50 mm. Among specimens examined range of TL 17.4–23.0 mm; range of GW 8.2–9.7 mm; range of PW 6.34–6.92 mm; range of PL 4.45–4.82 mm.

**Figure 90.**
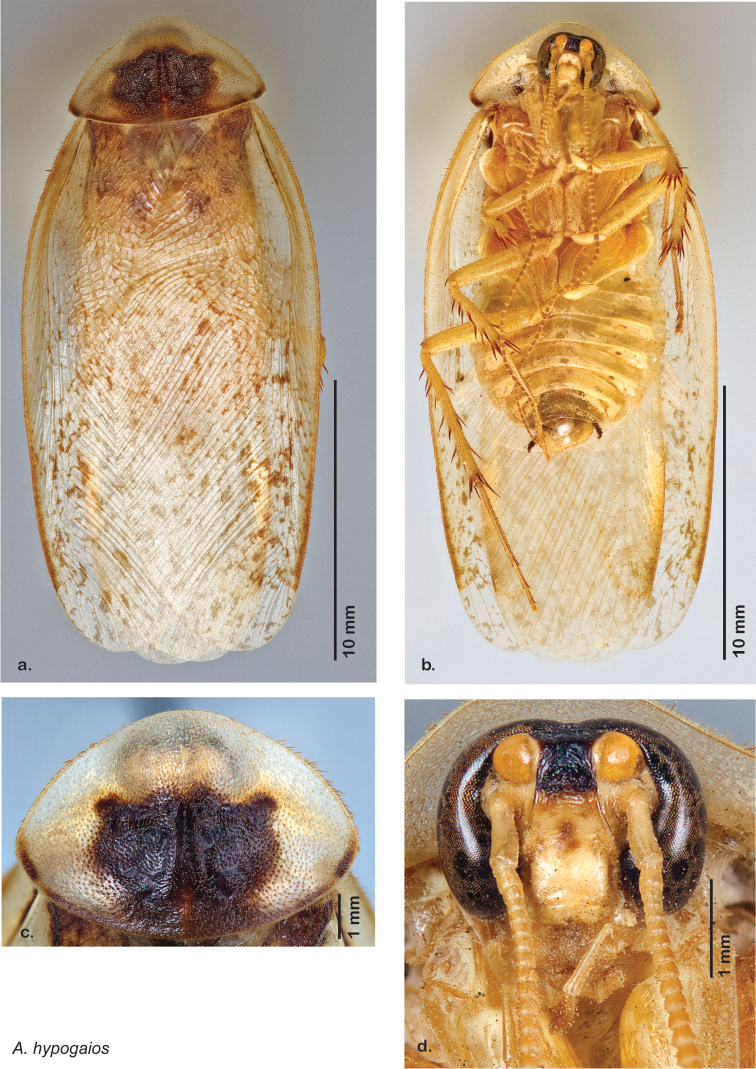
*Arenivaga hypogaios* **a** dorsal habitus **b** ventral habitus **c** pronotum **d** head.

*Head*. Two ocelli large, ovoid and protruding (0.50 × 0.40 mm); vertex dark brown, with small ridges between apices of eyes and extending onto ocellar tubercles; interocellar space concave, dark brown. Posterior frons light brown, tectiform horizontally then concave; anterior frons light brown, bulbous; light brown anteclypeus. See Fig. 90d.

*Pronotum*. Pronotum translucent waxy beige with dark brown posterior border; dorsal surface of pronotum with short orange-brown setae; pronotal pattern dark brown “hippo face”; impressed, no discernible detail; no aura. See Fig. 90c.

*Body*. Wing brace very small to absent. Two tarsal claws present. Legs and body light orange-brown; subgenital plate light orange-brown; asymmetrical with rounded apices. See Fig. 90b.

*Forewings*. Wings extended beyond abdominal apex (up to 40% of total wing length); light brown with widely scattered blotches medium orange-brown to medium brown; surface opaque and matte, or with slight sheen. See Fig. 90a.

**Figure 91.**
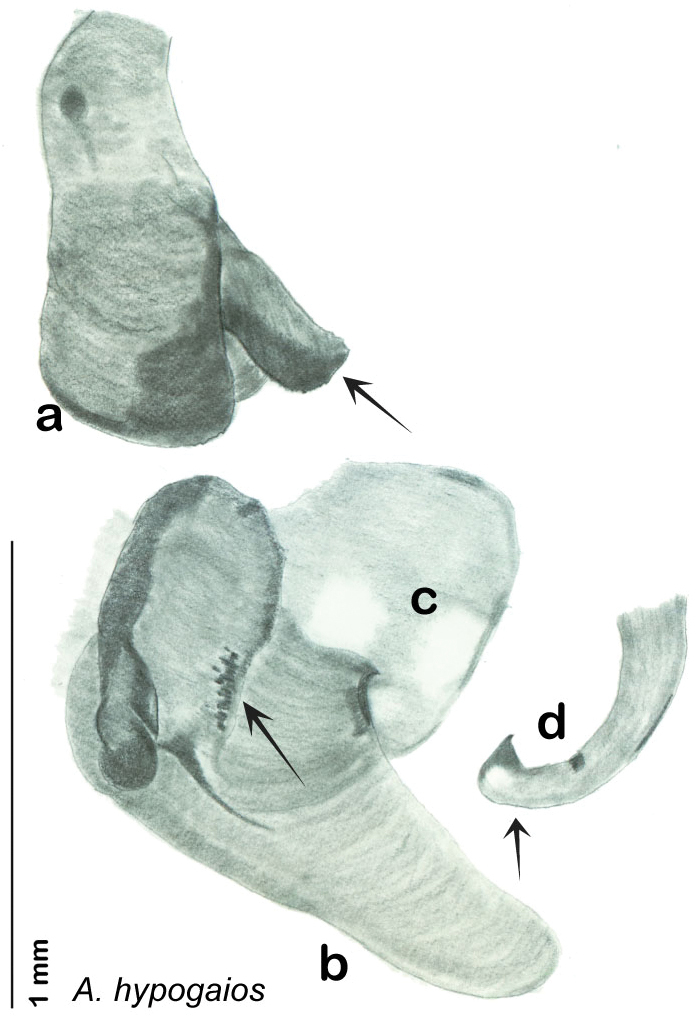
*Arenivaga hypogaios*, genitalia: a) right dorsal phallomere **b** right ventral phallomere **c** small central sclerite **d** genital hook. Arrow(s) indicate diagnostic characters (see text).

*Genitalia*. Right dorsal phallomere composed of lightly sclerotized, long, bulbous lobe, articulated with right ventral phallomere on lateral side; central field lightly sclerotized, deeply cupped; very short punctate medial edge, short shagreened ridge on lateral side of cup. Small central sclerite lightly sclerotized, finely punctate, flat; posterior end connecting with dorsal side of right dorsal phallomere. Articulation between right phallomeres extends into right ventral phallomere consisting of punctate to shagreened medially projecting lobe that is medially flattened; anteriorly moderate gap followed by small shagreened flange open-ended anteriorly. Folded anterior portion of left phallomere wide, with dense, short setae medially, otherwise unmodified. Genital hook with short extension to pointed head and short, wide hook; arm robust with distinct bend. See Fig. 91.

**Figure 92.**
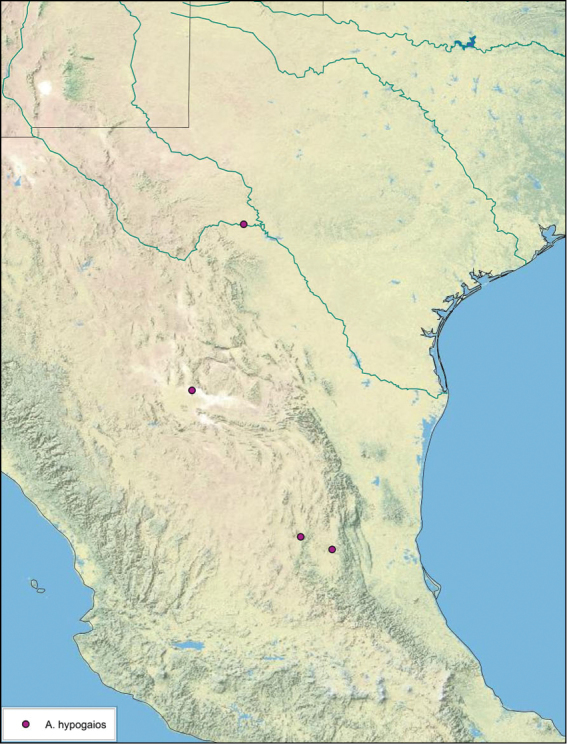
*Arenivaga hypogaios*, distribution.

##### Habitat and natural history.

All life history elements remain unobserved.

#### Arenivaga impensa sp. n.

http://zoobank.org/CA7785A1-33E2-4BB6-B4B3-0ABA6CD93288

http://species-id.net/wiki/Arenivaga_impensa

Figures 93 – 95

##### Type locality.

USA, Arizona, Kingman.

##### Material examined.

Holotype: ♂ in ANSP labeled “Kingman, Ariz. IX-10-1920 (O.C. Poling)” “HOLOTYPE *Arenivaga impensa* Hopkins, 2012” [red label with black border].

Paratypes (14): USA: AZ, Oak Creek Canyon, 4 mi. N of Sedona, 8/24/1966, RS Beal (1, NAUF); AZ, Indian Garden, Grand Canyon, 7/24/1934, 3800 ft., EL Bell & FE Lutz (2, AMNH); AZ, Coconino Co., Grand Canyon, 6/18/1954, M Cazier (1, AMNH); AZ, Coconino Co., Midgley Bridge, Oak Creek Canyon, 8/25/1952, Leech & Green (1, CAS); AZ, Mohave Co., Hualapai Mts., 7/15/1920, OC Poling (1, ANSP); AZ, Coconino Co., West Fork, Oak Creek Canyon, 9/19/1979, MW Sanderson, A79-31 (1, NAUF); AZ, Yavapai Co., WCCER, 8/18/2007, 34.92N, 112.834W, S Dorr 1 (1, NAUF); AZ, Yavapai Co., Grasshopper Flat, 8/6/1964, TL Bedwell (1, NAUF); AZ, Sawmill Canyon, Hualapai Mts., 8/30/1919, OC Poling (1, ANSP). All paratypes labeled “Paratype *Arenivaga impensa* Hopkins 2012” [blue label with black border].

##### Etymology.

The name is an adjective in the nominative singular. This species is named from the Latin meaning ample, large, or strong because of its large size for *Arenivaga*.

##### Distribution.

This species is found in the northwestern corner of Arizona. See Fig. 95.

##### Diagnosis.

The external phenotype of *Arenivaga impensa* may be confused with *Arenivaga tonkawa* but the genitalia distinguished them. *Arenivaga impensa* has a narrower hook-shaped lobe than *tonkawa* and the structures of the medial margin of the right dorsal phallomere are very different. See Figs 94 and 148.

##### Description.

**Male.** *Measurements*. Holotype TL = 24.0 mm, GW = 11.1 mm, PW = 7.56 mm, PL = 5.23 mm, TL/GW = 2.16, PL/PW = 0.69. EW = 0.45 mm; OW = 0.60 mm. Among paratypes range of TL 18.7–24.6 mm; range of GW 9.0–11.8 mm; range of PW 6.73–8.14 mm; range of PL 4.40–5.23 mm.

**Figure 93.**
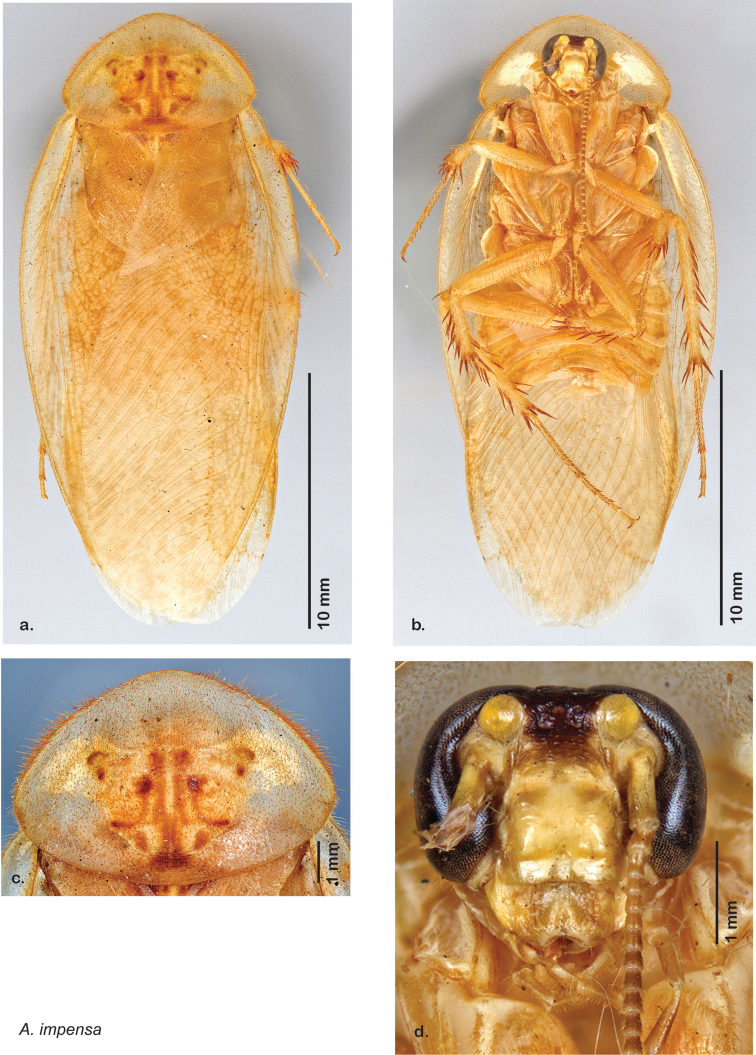
*Arenivaga impensa* **a** dorsal habitus **b** ventral habitus **c** pronotum **d** head.

*Head*. Two ocelli large, ovoid and protruding (0.45 × 0.40 mm); vertex brown with small ridges in rays around upper apex of eyes and extending onto ocellar tubercles; interocellar space only slightly concave, brown, with three small round indentations at points of an equilateral triangle. Posterior frons concave, brown grading into waxy beige, with horizontal corrugations; anterior portion of frons bulbous and waxy beige; waxy beige smooth anteclypeus. See Fig. 93d.

*Pronotum*. Pronotum large, translucent waxy beige; dorsal surface of pronotum with short orange-brown setae that are thicker and longer laterally; pronotal pattern orange-brown “panther face”, with little discernible detail; no aura. See Fig. 93c.

*Body*. Wing brace present. Two tarsal claws present. Legs and body light brown; some specimens with brown maculations laterally on each sternite; subgenital plate orange-brown; asymmetrical with rounded apices. See Fig. 93b.

*Forewings*. Wings extended well beyond abdominal apex (~35% of wing length); blotchy orange-brown to medium brown; surface opaque and matte. See Fig. 93a.

**Figure 94.**
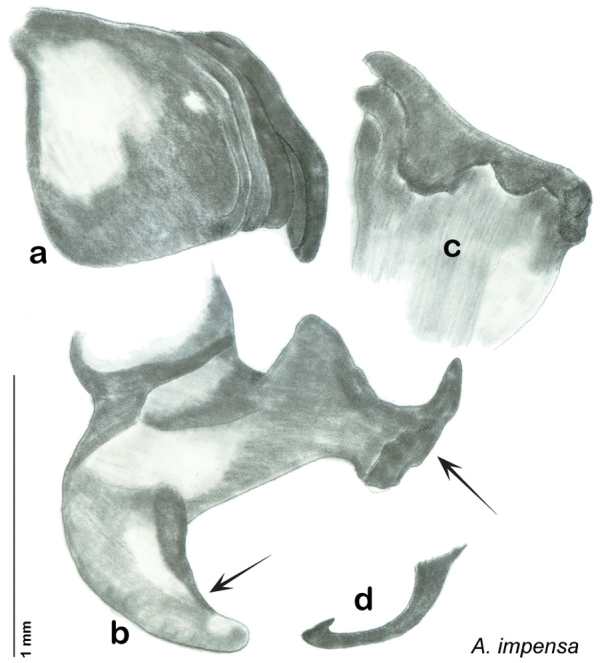
*Arenivaga impensa*, genitalia: a) right dorsal phallomere **b** right ventral phallomere **c** small central sclerite **d** genital hook. Arrow(s) indicate diagnostic characters (see text).

*Genitalia*. Right dorsal phallomere composed of lightly sclerotized bulbous hook-shaped lobe, articulated with right ventral phallomere on lateral side; central field lightly sclerotized with deep open rectangular area posterior-medially; medial margin more heavily sclerotized, with short toothed region at posterior end, and prominent flat spine adjacent, remainder of margin uneven. Small central sclerite with uneven margins nearly flat and finely punctate; anterior end bent posteriorly, shagreened with uneven toothed edge; right ventral phallomere extends from articulation to form smooth rounded structure, punctate, corrugated and narrowed anteriorly; attached anteriorly is mildly dorsally projecting flanged concave punctate arm that extends beyond depth of posterior portion of phallomere. Genital hook with moderate extension to rounded head with moderate hook; arm delicate with distinct bend. See Fig. 94.

**Figure 95.**
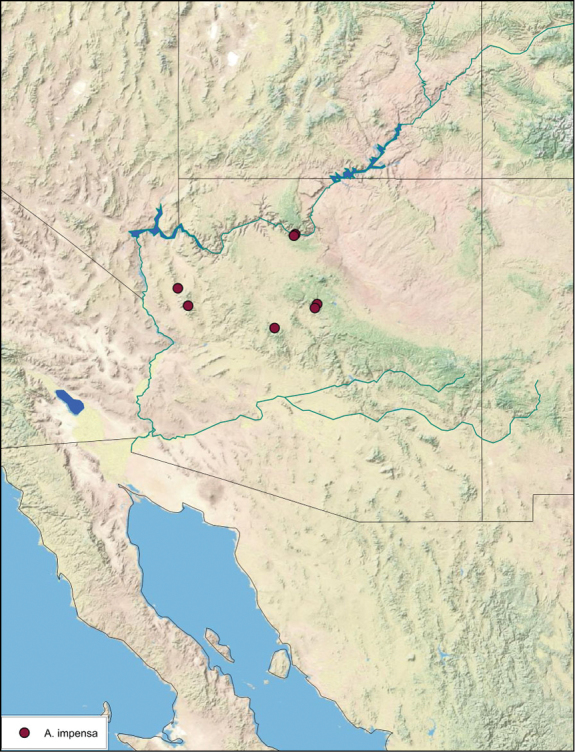
*Arenivaga impensa*, distribution.

##### Habitat and natural history.

All life history elements remain unobserved.

#### Arenivaga investigata

Friauf & Edney

http://species-id.net/wiki/Arenivaga_investigata

Figures 96 – 98

Arenivaga investigata Friauf and Edney 1969, Proceedings of the Entomological Society of Washington, 20(7), pp. 154–156.

##### Neotype locality.

USA, California, Riverside County, Windy Point.

##### Material examined

**(811).** Neotype: ♂ in EMEC labeled “CA, Riverside Co., Windy Point 3 mi. S of Whitewater, 1/25-26/1977, Doyen,Rude,Bentzien, J.Doyen Lot #77A2.1” “NEOTYPE *Arenivaga investigata* Hopkins, 2014” [red label with black border].

Neoparatypes (6): USA: CA, Riverside Co., Coachella Valley Dunes 3 mi. W of Thousand Palms, 5/19/1959, 200’, E.R.Tinkham, (6, USNM). All paratypes labeled “Neoparatype *Arenivaga investigata* Hopkins 2014” [blue label with black border].

##### Other material examined.

USA: CA, San Diego Co., Palm Canyon Cpgd Anza-Borrego SP, 6/4/1970, Crazier,Frencke, and Welch, taken at light (1, ASUT); CA, Imperial Co., Algodones Dunes, I8 at Ogilby Rd., 3/7/1988, R.E. Woodruff, dunes at night (1, FSCA); CA, Imperial Co., Algodones Dunes, I8 0.7 mi. W of Ogilby Rd., 3/5/1988, R.E. Woodruff, blacklight trap (3, FSCA); CA, Imperial Co., Algodones Dunes WA, Ted Kipf Rd. NW of Glamis, 3/25-28/2002, 33.01.29N 115.06.53W, 110m, K.Will et al., pitfall trap (1, EMEC); CA, Imperial Co., Imperial Sand Dunes RA, Wash Rd. ca. 7.2 mi. S Hwy. 78, 3/28/2002, 32.55.31N 114.58.52W, C.B. Barr, on dunes at night (1, EMEC); CA, Imperial Co., Hwy. 78 10.5 mi. SW of Glamis, 3/4/1987, R.A. Cunningham, (1, PMNH); CA, San Bernardino Co., Cronise Valley, 4/29/1956, B.J. Adelson/M.Wasbauer/J.Powell/P.D.Hurd, (4, EMEC); CA, San Diego Co., Borego, 4/20/1951, C.D. MacNeill, (1, CAS); CA, San Diego Co., Borego Valley, 6/6/1940, (1, CAS); CA, L.A. Co., Lovejoy Buttes, Mojave Desert, 4/15/1946, L. Martin, collected at light (1, LACM); CA, San Diego Co., Borrego Springs, 3/30/1960, M. Wasbauer, (1, CSCA); CA, Imperial Co., 17 mi. NW of Glamis, 6/27-28/1978, J.Doyen/J.Powell, white light, pitfall trap, JDLot #78F2 (4, EMEC); CA, Imperial Co., Seeley, 4/7/1971, E.L. Paddock, pit trap at night, CA Dept. of Agr. 71D19-85 (1, CSCA); CA, Imperial Co., El Centro, 4/16/1973, R.A. Flock, (5, CSCA); CA, Imperial Co., El Centro, 5/5/1973, (4, CSCA); CA, Imperial Co., Calipatria, 3/28/1962, Killgore coll., argon light trap (1, CSCA); CA, Imperial Co., El Centro, 4/16/1973, R.A. Flock, (1, CSCA); CA, Imperial Co., 6 mi. W of Glamis, 8/5/1966, M. Wasbauer, bowl traps in sand (1, CSCA); CA, San Diego Co., Borrego Springs, 6/22/1958, L.A. Stange, (1, LACM); CA, San Diego Co., Borrego, 5/3/1956, B.J.Adelson/P.D.Hurd/M.Wasbauer, (10, EMEC); CA, San Diego Co., Borego, 4/25-26/1955, P.D.Hurd, on one specimen “Cryptantha augustifolia” (6, EMEC); CA, San Diego Co., Ocotillo, 9/15/1947, G.A.Marsh, light (1, EMEC); CA, San Diego Co., Borrego, 5/8/1953, J.Powell, (1, EMEC); CA, San Diego Co., Borego, 4/25/1955, P.Wygodynsky, (1, EMEC); CA, Imperial Co., Glamis Sand Dunes,5 mi. W of Ogilby, 5/29/1981, Werner,Olson,Hetz,Thomas,Burne,Frank,McLachlan, (1, UAIC); CA, San Bernardino Co., Kelso Sand Dunes, 5/1/1974, 2500’, Eichlin & Hardy, (1, CSCA); CA, Riverside Co., Mule Mts., I13, 2/13/1983, Andrews & Gilbert, (1, CSCA); CA, Riverside Co., 11.8 mi. WNW Inca RR Siding, 4/7/1994, 33.49.4N 114.57.3W, R.R.Snelling, (1, LACM); CA, Imperial Co., Algodones Dunes, 1–2 mi. W of Glamis, 4/7/2008, 32.59N 115.06?, Bill Warner, (1, W.B.Warner); CA, Imperial Co., 1 mi. W of Glamis, 3/29/1979, R.L.Aalbu, collected on slip face of dune (1, CSCA); CA, Imperial Co., 5 km. N of Glamis sand dunes, 4/17/1974, D.Giuliani, (1, CSCA); CA, Riverside Co., Palm Springs, 5/23/1940, W.L.Swisher, light (2, LACM); CA, Riverside Co., Palm Springs, 6/24/1956, A.Menke,Jr., (1, LACM); CA, Riverside Co., Palm Springs, 7/8/1956, L.A.Stange, (1, LACM); CA, Riverside Co., Palm Springs, 7/19/1958, L.A.Stange, (1, LACM); CA, Riverside Co., Cathedral City, 6/14/1958, L.A.Stange, (1, LACM); CA, Riverside Co., Cathedral City, 7/21/1952, R.Tinglof, UCLA Coll. Accessioned LACM 1965 (1, LACM); CA, Riverside Co., Pushawalls Canyon, Indio Hills, 7/14/1947, (1, LACM); CA, Riverside Co., Palm Springs, 5/24/1940, Brereton & King, lights (1, LACM); CA, Riverside Co., Palm Springs, 5/24/1940, (1, CSCA); CA, Riverside Co., Palm Springs, 7/6/1950, W.A.McDonald, (1, USNM); CA, Riverside Co., Palm Springs, 7/16/1950, D.C.Blodget, (1, USNM); CA, Imperial Co., 3 mi. NW of Glamis, 9/15-16/1972, Wasbauer & Hardy, blacklight trap (1, CSCA); CA, Imperial Co., 3.5 mi. NW of Glamis, 3/10/1975, Andrews & Hardy, sand dune (1, CSCA); CA, Riverside Co., Thermal, 6/18/1956, M.Wasbauer, (4, EMEC); CA, Riverside Co., Thermal, 7/17/1956, M.Wasbauer, (1, EMEC); CA, Imperial Co., 3 mi. NW of Glamis, 3/3-4/1972, E.A.Kane, sand dunes (3, CSCA); CA, Co., 25 mi. W of Blythe, 8/18-19/1927, Cornell University Lot 542 sub 326, *Arenivaga apacha* Sauss.Det.Hebard 1929 (1, ANSP); CA, Imperial Co., Algodones Dunes 2 mi. W of Sand Hills Rest Area, 4/26/1980, D.K.Faulkner, (1, SDMC); CA, Imperial Co., 13 mi. W of Winterhaven, 6/13/1958, V.Roth, (1, UAIC); CA, Imperial Co., Imperial Dam, 5/28/1954, W.McDonald, UCLA Coll. Accessioned LACM 1965 (1, LACM); CA, Imperial Co., Algodones Sand Hills, W.G.Reeder, blattidae,500224 (4, USNM); CA, Riverside Co., Hopkins Well, 4/14-16/1958, P.D.Hurd/J.Powell, (15, EMEC); CA, Imperial Co., Algodones Dunes,5.6 mi. NW & 1 mi. SW of Glamis, 3/20/1993, 270’, J.P. & K.E.S. Donahue, 164,060-A (5, LACM); CA, Imperial Co., Algodones Dunes, S of Ruthven, 4/6/2000, 32.55.30N 114.59.34W, 120 m, D.Yanega, at light (2, UCRC); CA, Imperial Co., Algodones Dunes, S of Ruthven, 4/30/2001, 32.55.30N 114.59.34W, Hawks & Yanega, (5, UCRC); CA, Imperial Co., 5 mi. N of Ogilby, 4/17/1965, R.C.Dickson, at light (1, UCRC); CA, San Bernardino Co., Saratoga Springs, Death Valley, 7/10-12/1953, (2, USNM); CA, Calexico, 8/11/1914, J.C.Bradley, Cornell U. Lot 882,Sub.146, *Arenivaga apacha* (Sauss.)Det.Hebard 1935 (1, CUIC); CA, Imperial Co., Salton Sea, North Shore Area, 3/30/1983, R.A.Cunningham, (1, PMNH); CA, Cronese, 4/28/1952, Timberlake, at light (1, UCRC); CA, 1000 Palms, D.W.Cherney, (1, UCRC); CA, 2 mi. S of Oasis, ?/7/1936, Timberlake, at light (1, UCRC); CA, Riverside Co., Blythe, 7/22/1916, W.F.Barr, *Arenivaga apacha* 9Sauss.) Det.Strch.1952 (1, USNM); CA, Imperial Co., Imperial Valley, Summer 1966, D.A.Ward, (1, UCRC); CA, Imperial Co., Holtville, 7/2/1929, P.W.Oman, (1, SEMC); CA, Imperial Co., 8 mi. E of Holtville, 4/29/1961, D.S.Verity, (1, LACM); CA, Imperial Co., 8 mi. E of Holtville, 6/24/1958, G.H.Nelson, to light (1, UMMZ); CA, Brawley, 4/7/1925, (1, LACM); CA, San Diego Co., Borego SP, 6/4/1956, A.Menke, (1, LACM); CA, San Diego Co., Borego SP, 6/6/1940, around lights (2, LACM); CA, Los Angeles Co., (2, LACM); CA, Colorado Desert, 8/11/1917, Wheeler, *Arenivaga* wheeleri n.sp.M.S.Det.T.H.Hubbell 1928 (1, UMMZ); CA, San Bernardino Co., Yermo, 4/11/1936, J.A.Comst., L.J.Muchmore (2, LACM); CA, San Bernardino Co., Yermo, 7/5/1939, W.M.Pearce, ex.coll.M.A.Cazier (1, AMNH); CA, San Bernardino Co., Yermo, 5/15/1939, T.G.Altken, ex.coll.M.A.Cazier (1, AMNH); CA, San Bernardino Co., Deadman’s Point, 9/9/1963, R.J.Hamton, (1, HEH); CA, Riverside Co., Palm Desert, 3/26/1953, AH & SK Rindge, collection of Fred H Rindge (1, AMNH); CA, Riverside Co., Palm Desert, 6/22/1956, M.Wasbauer, (3, EMEC); CA, Riverside Co., Dos Palmas Spa, 4/5/1937, G.Willett, (1, LACM); CA, Riverside Co., Coachella Valley Dunes,2 mi. W of Indio, 5/25/1957, 10’, E.R.Tinkham, (1, USNM); CA, Riverside Co., Coachella Valley, 10/23/1938, JAC, (1, LACM); CA, Riverside Co., Coachella Valley, 10/30/1938, J.A.Comstock, (1, LACM); CA, Riverside Co., N end of Salton Sea, 5/19/1955, F.S.Truxal, (4, LACM); CA, Imperial Co., 2 mi. S of Palo Verde, 6/28/1978, D.J.Powell, (1, EMEC); CA, Riverside Co., N shore near Salton Sea, 5/30/1992, R.L.Allen, white light, Blattodea:Polyphagidae, *Arenivaga* (1, LACM); CA, San Bernardino Co., Stovepipe Wells Hostel, Death Valley NM, 4/2/1954, F.B.Turner, at light (1, EMEC); CA, Inyo Co., 7 mi. NE of Panamint Springs, 5/16/1969, Rude & Doyen, (3, EMEC); CA, Kern Co., Red Rock Canyon, 5/2/1968, J.T.Doyen/J.Powell, blacklight (6, EMEC); CA, San Diego Co., 6 mi. E of Banner, 7/13/1963, H.L.Griffin/J.Powell/P.Welies, at light (5, EMEC); CA, San Bernardino Co., Death Valley NM,2 mi. NE Saratoga Springs, 3/17/1978, P.Rude, at UV light (1, EMEC); CA, Inyo Co., Shoshone, 5/4/1962, R.W.Thorp, at light (1, EMEC); CA, San Diego Co., Borego, 4/28/1955, R.C.Schuster, (1, EMEC); CA, San Diego Co., Borrego, 4/21/1960, J.Powell, (3, EMEC); CA, San Diego Co., Borrego Springs, 6/11/1965, G.R.Ballmer, light (1, UCRC); CA, San Diego Co., Borego, 4/28/1955, R.O.Schuster, 1 specimen-*Arenivaga* sp.Det.H.F.Strohecker (2, EMEC); CA, San Diego Co., Borego, 5/14/1949, J.E.Giliaspy, coll. At light (1, FSCA); CA, San Diego Co., Borego, 4/24/1959, Timberlake, at light (3, UCRC); CA, San Diego Co., Anza Borrego off 53 June oasis, 4/3/1993, S.O’Keefe, bl.sand at night (1, EMEC); CA, Inyo Co., Owens Riv. 2 mi. NE of Lone Pine, 5/11/1969, P.A.Opler, blacklight (1, EMEC); CA, Inyo Co., Olancha, 6/26/1949, H.E.Cott, (1, FSCA); CA, Inyo Co., 3 mi. S of Olancha, 8/6/1948, Hurd & MacSwain, *Arenivaga apacha* (Sauss.) Det.H.F.Strohecker (1, FSCA); CA, San Bernardino Co., Afton Canyon, 3/24/1997, M.S.Caterino, (2, EMEC); CA, San Bernardino Co., Afton Rd. 23 mi. SW of Baker, 4/23/1977, Kitayama,Cave & Chemsak, on sand at UV light (1, EMEC); CA, San Bernardino Co., Yermo, 4/22/1949, R.v.d.Bosch, light trap (1, FSCA); CA, San Bernardino Co., Saratoga Springs, Death Valley NM, 4/15/1965, C.W.O’Brien, (1, FSCA); CA, Imperial Co., Holtville, 7/15/1989, (1, FSCA); CA, Imperial Co., Holtville, 5/8/1997, W.F.Chamberlain, at light (5, TAMU); CA, San Bernardino Co., Death Valley Juno, 4/22/1935, A.L.Melander, (1, UCRC); CA, Inyo Co., Darwin Canyon,5.7 mi. NE of Darwin, 7/6/1991, 2520’, J.P. & K.E.S. Donahue, T18S,R41E,SE1/4 Sec.34,#21,874 (7, LACM); CA, San Bernardino Co., S. side of Kelso Dunes, 6/19/1999, Ballmer,Hawks,Powells & Yanega, (4, UCRC); CA, San Bernardino Co., Baker, Mohave Desert Scrub, 8/26/2002, L. Stange, at light (1, FSCA); CA, Riverside Co., 2 mi. W of Hopkins Well, 3/3/1959, J.W.MacSwain, (5, EMEC); CA, Riverside Co., Hopkins Well, 3/7/1959, W.E.Ferguson, at light (2, EMEC); CA, Riverside Co., Hopkins Well, 6/20/1966, J.W.MacSwain, (1, EMEC); CA, Riverside Co., 5 mi. e of Mt. Eagle, 7/8/1976, Doug Whitman, #570 (8, EMEC); CA, Riverside Co., Indio, 4/16/1965, J.Doyen, at light (3, EMEC); CA, Riverside Co., Indio, 8/4/1959, C.R.James, (1, FSCA); CA, Riverside Co., 4 mi. S of Palm Desert, 7/2/1963, R.L.Langston, at light (1, EMEC); CA, Riverside Co., Deep Canyon at station, 6/17/1975, P.McNally, white light (1, UCRC); CA, Riverside Co., Coachella Valley, Miles Ave. 0.5 mi. SW of Washington St., 2/19/1990, G.R.Ballmer, pitfall trap (2, UCRC); CA, Needles, 4/1-6/1918, J.C.Bradley, (1, ANSP); CA, San Bernardino Co., Kelso Dunes,8 mi. SW of Kelso, 7/14-15/1974, J.Doyen, on ground at light, Lot No.74G13 (3, EMEC); CA, Imperial Co., 16 mi. W of AZ/CA border on I10,sand dunes off Ogilby, 2/27/1988, M.Thomas, crawling on sand dune, Polyphagidae Det. Max Thomas 1988 (1, ASUT); CA, San Bernardino Co., Essex, 4/5/1966, 1700’, P.A.Opler, (1, EMEC); CA, San Bernardino Co., 9 air mi. S of Baker, Zzyzx Springs, 4/20/1977, Chemsak, (1, EMEC); CA, San Bernardino Co., 9 air mi. S of Baker, Zzyzx Springs, 7/1/1978, J.Doyen, Lot #78F5 (2, EMEC); CA, San Bernardino Co., 9 air mi. S of Baker, Zzyzx Springs, 4/18-23/1984, S.Hawley, blacklight trap (1, ); CA, San Bernardino Co., 9 air mi. S of Baker, Zzyzx Springs, 4/18-23/1984, R.Gill, (2, EMEC); CA, San Bernardino Co., Zzyzx Springs, 4/20/1984, D.Sandri, (1, EMEC); CA, San Bernardino Co., Soda Springs (Zzyzx) W side of Soda Dry Lake,9 air mi. S of Baker, 5/22-24/1982, J.P.Donahue, (1, LACM); CA, Riverside Co., Eagle Mt. Dunes, E corner of JoshUAIC Tree NM, 6/17/1961, (13, USNM); CA, Riverside Co., Indio, 8/?/1950, (1, USNM); CA, Riverside Co., Indio, 7/2/1961, (1, USNM); CA, Riverside Co., 3 mi. W of Indio, 7/2/1956, M.Wasbauer, (4, EMEC); CA, Riverside Co., Indio, 6/7/1956, M.Wasbauer, (1, EMEC); CA, Riverside Co., Indio, 4/24-26/1953, G.Yamamoto/A.Fukushima, (5, USNM); CA, Riverside Co., Indio, 5/9/1952, (1, USNM); CA, Riverside Co., Indio, 4/30/1952, (1, USNM); CA, Riverside Co., Indio, 6/15/1955, (1, USNM); CA, Riverside Co., Indian Wells, 4/29/1952, (1, USNM); CA, Riverside Co., Indian Wells, 4/17/1952, (2, USNM); CA, Riverside Co., Indian Wells, 4/24-25/1953, B.Markley/W.R.Lower/M.C.Anderson, (10, USNM); CA, Riverside Co., Indian Wells, 9/7/1938, R.B.Cowles, (4, USNM); CA, Riverside Co., Indian Wells, 4/5/1931, A.C.Browne, Collected from desert flora, (3, HEH); CA, Riverside Co., Indian Wells, 3/12/1938, R.B.Cowles, (2, USNM); CA, Riverside Co., 3 mi. W of Indio, 5/19/1959, (3, USNM); CA, Riverside Co., Coachella Valley Dunes 3 mi. W of Thousand Palms, 5/3/1958, 200’, E.R.Tinkham, (5, USNM); CA, Riverside Co., Coachella Valley 9 mi. W of Palm Springs, 4/2/1954, E.R.Tinkham, (1, USNM); CA, Riverside Co., Coachella Valley Dunes 3 mi. W of Thousand Palms, 5/19/1959, 200’, E.R.Tinkham, (1, USNM); CA, Riverside Co., Coachella Valley Dunes 9 mi. W of Palm Springs, 5/27/1954, E.R.Tinkham, (1, USNM); CA, Riverside Co., Coachella Valley Dunes 1 mi. N of Palm Desert, 5/15/1954, E.R.Tinkham, (1, USNM); CA, Riverside Co., Whitewater, 7/9/1950, J.W.MacSwain, (1, USNM); CA, Riverside Co., Palm Springs, 6/?/1965, E.B.Edney, (2, USNM); CA, Riverside Co., Indio, 4/29/1962, V.Theresa Luine, (1, UCRC); CA, Riverside Co., San Gorgonio Pass, Windy Point, 6/16/1975, P.McNally, (4, UCRC); CA, Riverside Co., Whitewater, 7/9/1950, P.D.Hurd, (1, FSCA); CA, Riverside Co., Windy Point sand dunes nr. Palm Springs, broad form, bred from nymphs (1, UCRC); CA, Riverside Co., Windy Point 3 mi. S of Whitewater, 1/25-26/1977, Doyen,Rude,Bentzien broad form, J.Doyen Lot #77A2.1 (1, EMEC); CA, Riverside Co., Coachella Valley, Mt.View Rd. 0.5 mi. N of jct. w/ Varner Rd., 5/17/1995, G.R.Ballmer, (1, UCRC); CA, 4 mi. W of Thousand Palms, 4/25/1968, E.R.Tinkham, (3, USNM); CA, 3 mi. W of Thousand Palms, 5/10/1960, (1, USNM); CA, Riverside Co., Palm Springs, 1-6/?/1966, Edney, (6, USNM); CA, Riverside Co., Palm Springs, 7/19/1958, L. Stange, (1, LACM); CA, Riverside Co., Palm Springs, 6/5/1967, R.Rice, in sand under bush (1, UAIC); CA, Calexico, 8/11/1914, J.C.Bradley, (1, ANSP); CA, Riverside Co., Palm Springs, 6/1/1937, P.D.Gerhardt, at light (1, UAIC); CA, San Felipe, 9/9/1938, Timberlake, at light (1, UCRC); CA, Riverside Co., Blythe, 5/24/1935, G.M.Kohle, *Arenivaga erratica* Rehn Det. Rehn 1941 (1, ANSP); CA, Indio, 4/19/1962, Erwin #134 (1, USNM); CA, Riverside Co., Blythe, 8/9-10/1959, K.L.Japport, Rentz, argon light trap (4, USNM); CA, Riverside Co., Palm Springs, 4/?/1966, C.&V. Brandes, (1, USNM); CA, San Diego Co., Mason Valley, 4/14/1945, D.Meadows, (1, USNM); CA, Imperial Co., Experiment Farm, 6/7/1912, J.C.Bridwell, (3, USNM); CA, Imperial Co., Imperial Sand Dunes, 3/28/2002, G.M.Nishida, at MV light (2, EMEC); CA, Riverside Co., Coachella Valley 3 mi. W of Indio, 5/9/1955, E.R.Tinkham, mated pair (1, USNM); CA, Riverside Co., Coachella Valley, Indio, 7/5/1957, E.R.Tinkham, (2, USNM); CA, Riverside Co., Coachella Valley 4 mi. W of Indio, 4/18/1955, E.R.Tinkham, (1, USNM); CA, Riverside Co., Coachella Valley 9 mi. W of Palm Springs Dunes, 5/27/1954, E.R.Tinkham, (1, USNM); CA, Kelso Dunes, 6/24-25/1954, 2400’, E.R.Tinkham, (2, USNM); CA, Riverside Co., Coachella Valley Dunes 2 mi. W of Indio, 5/25/1957, 10’, E.R.Tinkham, (1, USNM); CA, San Diego Co., Ocotillo, 6/13/1949, G.A.Marsh, (1, USNM); CA, San Diego Co., Anza Borrego SP, Borrego Palm Canyon Cpgd., 4/10/1993, R.L.Allen, (1, LACM); CA, Riverside Co., Palm Springs, 5/24/1940, (1, CAS); CA, Riverside Co., Palm Springs, 5/28/1939, P.D.Gerhardt, at light (1, UAIC); CA, Inyo Co., Death Valley 1.5 mi. N 2 mi. W Ashford Mill, 3/23/1984, 200’, D.Giuliani, sand dunes (1, CSCA); CA, Inyo Co., 4.3 mi. NE Saratoga Springs, Death Valley NM, 4/16-18/1973, A.R.Hardy, sand dunes (4, CSCA); CA, San Bernardino Co., Baker, 5/29/1950, C.D. MacNeill, (1, EMEC); CA, 65 mi. ENE of Indio,13 mi. NE & 2.5 mi. E of Desert Center, 4/3/1960, E.R.Tinkham, (2, USNM); CA, Riverside Co., Desert Center, 8/31/1946, (1, LACM); CA, Inyo Co., Tecopa, 6/17/1954, Belkin & McD., UCLA Coll. Accessioned LACM 1965 (1, LACM); CA, 29 Palms, 5/?/1952, E.R.Tinkham, (1, USNM); CA, Riverside Co., Coachella Valley 2 mi. W of Indio, 5/25/1955, E.R.Tinkham, (1, USNM); CA, Sand dunes E of Gray’s Well, 7/18/1953, E.R.Tinkham, (1, USNM); CA, San Diego Co., Borego SP, 4/11/1949, E.S.Ross, (2, CAS); CA, Holtville, 6/23/1946, E.C.VanDyke, (6, CAS); CA, Imperial Co., Coyote Wells, 5/5/1922, O.C.Poling, (4, ANSP); CA, 5 mi. W of Blythe, 8/19-20/1927, CUIC University (3, ANSP); CA, Death Valley, 10/?/1926, J.D.Gunder, (1, CAS); CA, Coachella, 5/23/1929, E.C.VanDyke, (1, CAS); CA, Riverside Co., Palm Springs, 4/22-27/1933, E.P.Van Duzee, (5, CAS); CA, Kern Co., 5/?/????, (1, ANSP); CA, Coachella, 5/19/1923, E.C.VanDyke, (1, CAS); CA, Riverside Co., 3/30/1918, E.R.Leach, (2, CAS); CA, Coachella, 5/10/1928, E.C.VanDyke, (1, CAS); CA, Coachella, 5/22-25/1928, E.C.VanDyke, (3, CAS); CA, Riverside Co., Indian Wells, 4/24/1922, K.R.Coolidge, at light (1, ANSP); CA, 25 mi. W of Blythe, 8/18-19/1927, Cornell University Lot 542 sub 326 (6, ANSP/CUIC); CA, Holtville, 7/2/1929, R.H.Beamer, (1, ANSP); CA, Needles, 4/1-6/1918, (1, ANSP); CA, Needles, 4/1-6/1918, J.C.Bradley, Cornell University Lot 882 sub 146 (1, CUIC); CA, Kern Co., Red Rock Canyon, 5/2/1968, J.T.Doyen, black light (1, EMEC); CA, Inyo Co., Owen’s River 2 mi. NE of Lone Pine, 5/11/1969, P.A.Opler, black light (1, EMEC); CA, Inyo Co., 31 mi. NE of Big Pine, 7/8/1966, 6000’, C.W.O’Brien, at night (1, FSCA); CA, Heber, 7/10/1926, (1, UCRC); CA, Inyo Co., 9 mi. N of Olancha nr Cottonwood Charcoal Kilns, 5/24/1969, R.Hardy, (1, UCRC); CA, Riverside Co., Wiley’s Well Rd. at I10, 3/28/2001, 33.36.22N 114.54.30W, D.Yanega, (2, UCRC); CA, San Bernardino Co., 23 mi. E of 29 Palms, 4/30/1971, R.Hardy, (1, UCRC); CA, San Bernardino Co., Kelso Dunes, 4/16-18/1974, Andrews & Wasbauer, (3, CSCA); CA, Indio, 8/31/1961, C.Myers, (1, CSCA); CA, Riverside Co., Blythe, 7/8/1956, A.Menke, Jr., (1, LACM); CA, Riverside Co., Indian Wells, 4/1/1938, R.B.Cowles, UCLA Coll. Accessioned LACM 1965 (2, LACM); CA, Riverside Co., Indian Wells, 4/12/1957, C.W.Schaefer, UCLA Coll. Accessioned LACM 1965 (1, LACM); CA, Riverside Co., Indian Wells, 4/21/1956, Schlek, UCLA Coll. Accessioned LACM 1965 (1, LACM); CA, Riverside Co., Blythe, 8/20/1927, Cornell University (3, ANSP/CUIC); CA, Riverside Co., 5 mi. W of Blythe, 8/19-20/1927, Cornell University Lot 542 sub 328, *Arenivaga apacha* (Sauss.) Hebard 1937 (1, CUIC); CA, Riverside Co., 5 mi. W of Blythe, 8/19-20/1927, Cornell University Lot 542 sub 328 (1, CUIC); CA, Riverside Co., 25 mi. W of Blythe, 8/18-19/1927, Cornell University Lot 542 sub 326, *Arenivaga apacha* (Sauss.) Hebard 1929 (2, CUIC/UCRC); CA, Riverside Co., 18 mi. W of Blythe, 4/8/195?, Timberlake, at light (5, UCRC); CA, Riverside Co., Blythe, 8/25/1973, (1, UCRC); CA, Inyo Co., Shoshone, 9/10/1954, UCLA Coll. Accessioned LACM 1965 (1, LACM); CA, Inyo Co., Bailey Canyon, Carlego, 7/2/1940, (1, LACM); CA, San Bernardino Co., Amboy Crater, 7/22/1956, J.F.Lawrence, 102 (1, EMEC); CA, San Bernardino Co., Amboy Crater, 6/6/1957, J.M.Burns, (1, EMEC); CA, San Bernardino Co., 10 mi. E of 29 Palms, 6/11/1966, fluorescent black light (6, CSCA); CA, Imperial Co., 6 mi. W of Glamis, 8/5/1966, M.Wasbauer, bowl traps in sand (2, CSCA); CA, Imperial Co., Andrade, 8/4/1966, M.Wasbauer, fluorescent black light (1, CSCA); CA, Cornise, 4/28/1937, orthoptera, *Arenivaga apacha* (Sauss.) det. H.F.Strohecker (1, FSCA); CA, Imperial Co., 3 mi. SW of Glamis, 7/12/1974, J.Doyen, black light trap (1, EMEC); CA, San Diego Co., Ocotillo, 6/13/1949, (1, EMEC); CA, San Diego Co., Ocotillo, 9/15/1947, G.A.Marsh, light (1, EMEC); CA, San Bernardino Co., Pisgah Crater, 4/7/1962, Norris & Heath, Sta.33 (1, LACM); CA, San Bernardino Co.,. 3 mi. S of I15, Basin Pond, 7/15/1984, Faulkner & Brown, (1, SDMC); CA, San Bernardino Co., Baker, 7/31/1955, Menke & Truxal, (1, LACM); CA, San Bernardino Co., Cronise Valley, 4/29/1956, M.Wasbauer/J.Powell, (3, EMEC); CA, San Diego Co., Borrego, 5/3/1956, B.J.Adelson, (1, EMEC); CA, San Diego Co., Borego, 4/23/1955, P.D.Hurd, (4, EMEC); CA, Riverside Co., Hopkins Well, 4/16/1958, P.D.Hurd/J.Powell, (2, EMEC); CA, Ft. Yuma, 8/16/1951, origin unknown, FY 51,H25 (1, CSCA); CA, Riverside Co., Thousand Palms, 7/28/1958, P.Opler, (1, EMEC); CA, Imperial Co., Glamis Sand Dunes,5 mi. W of Ogilby, 5/29/1981, Werner,Olson,Hetz,Thomas,Burne,Frank & MacLachlan, (2, UAIC); CA, 4 mi. W of Thousand Palms, 4/25/1968, E.R.Tinkham, (1, USNM); CA, Riverside Co., Indio, 6/25/1939, J.C.von Bloeker, (1, LACM); CA, Inyo Co., “The Dunes” Panamint Valley, 4/28/1974, 2600’, Eichlin & Hardy, (1, CSCA); CA, Inyo Co., Panamint Valley Dunes, 9/14/1975, Andrews & Hardy, (1, CSCA); CA, San Bernardino Co., Death Valley NM, Saratoga Springs, 5/3/1974, D.Giuliani, (2, CSCA); CA, San Bernardino Co., Marble Mts. 1 mi. E of Kelbaker Rd., 5/7-8/10/1981, R.Aalbu, antifreeze trap (1, CSCA); CA, Riverside Co., Blythe, 7/26/1946, Hurd & Barr, 17NW (1, USNM); CA, Riverside Co., 20 mi. W of Blythe, 7/4/1951, J.W.MacSwain, (1, USNM); CA, Riverside Co., Blythe, 6/21/1946, W.F.Barr, (1, USNM); CA, San Diego Co., Borego, 4/27/1954, J.G.Rozen, *Arenivaga apacha* (Sauss.) det. H.F.Strohecker (1, USNM); CA, Coachella Valley,2 mi. NW of Indio, 5/9/1955, E.R.Tinkham, (1, USNM); CA, Coachella Valley,3 mi. W of Indio, 5/9/1955, E.R.Tinkham, (1, USNM); CA, Coachella Valley,3 mi. W of Indio, 5/25/1955, E.R.Tinkham, (1, USNM); CA, Coachella Valley,4 mi. W of Indio, 4/16/1955, E.R.Tinkham, (1, USNM); CA, Saratoga Springs, Death Valley, 4/16/1965, J.B.Snell, (1, USNM); CA, Riverside Co., Indio, 4/20/1939, (1, LACM); CA, Riverside Co., Hopkins Well, 4/27/1949, L.W.Quate, coll. at light, *Arenivaga apacha* (Sauss.) det.H.F.Strohecker (1, USNM); CA, Riverside Co., Strawberry Valley, S of Jacinto Mt., 3/4/1910, Grinnell,Jr., (1, USNM); CA, San Bernardino Co., Yermo, 4/11/1949, R.v.d.Bosch, light trap (1, USNM); CA, San Diego Co., Borego, 5/1/1952, P.D.Hurd, *Arenivaga apacha* (Sauss.) det. H.F.Strohecker 1953 (2, USNM); CA, San Diego Co., Borego, 4/24/1949, J.E.Gillaspy, coll. At light (1, USNM); CA, Riverside Co., Cathedral City, 7/16/1950, B.Adelson, electric light (1, USNM); CA, Riverside Co., Whitewater, 7/9/1950, J.W.MacSwain, (1, USNM); CA, Riverside Co., Mecca, 4/25/1952, P.D.Hurd, (1, USNM); CA, San Bernardino Co., Cronise Camp, 4/16/1953, J.Linsley, (2, USNM); CA, Salt Creek Death Valley, 4/14/1965, J.L.Pierce, collected at light (1, USNM); CA, Stovepipe Wells Death Valley, 4/5/1966, D.Ramsey, at light (1, USNM); CA, Stovepipe Wells Death Valley, 4/5/1966, K.St??????, on table at light (1, USNM); CA, Stovepipe Wells Death Valley, 4/14/1965, W.H.Tyson, collected at light (1, USNM); CA, Stovepipe Wells Death Valley, 4/15/1965, W.E.Ferguson, (1, USNM); CA, Stovepipe Wells Death Valley, 4/5/1966, P.Gibbs, collected at light (1, USNM); CA, Stovepipe Wells Death Valley, 4/6/1966, R.E.Main, collected at light (1, USNM); CA, Riverside Co., Hopkins Well, 4/29/1952, J.G.Rozen, (1, USNM); CA, Riverside Co., Hopkins Well, 4/14-16/1958, P.D.Hurd/J.Powell, (8, USNM/ EMEC); CA, Imperial Co., El Centro, 8/?/1981, H.W.Browning, (2, UCMC); CA, Imperial Co., El Centro, 8/6/1963, R.Flock, (1, UCMC); CA, Olancha, 4/27/1959, R.P.Allen, (2, CSCA); CA, Imperial Co., 3.5 mi. NW of Glamis, 3/10/1973, Andrews & Hardy, sand dune (1, CSCA); AZ, Yuma Co., Wellton, 2/25-26/1925, O.C.Poling, (13, UMMZ); AZ, Yuma Co., Wellton, 3/3/1925, O.C.Poling, (3, UMMZ); AZ, Yuma Co., Wellton, 3/25/1925, O.C.Poling, (17, UMMZ); AZ, Yuma Co., Mohave Valley nr. Wellton, 3/5/1925, O.C.Poling, at light, (1, UMMZ); AZ, Yuma Co., Wellton, 3/6/1925, O.C.Poling, (7, UMMZ); AZ, Yuma Co., Welton, 6/28/1950, R.F.Smith, (1, AMNH); AZ, Yuma Co., Sentinel, 7/23/1941, R.H.Beamer, (20, ANSP); AZ, Yuma Co., Ehrenberg, 6/24/1938, F.H.Parker, (2, ); AZ, Maricopa Co., Gila Bend, 6/19/1953, UCLA Coll. Accessioned LACM 1965 (2, LACM); AZ, Yuma Co., Aztec, 4/16/1954, Menk & Stange, (1, LACM); AZ, Yuma Co., Mohawk Dunes 12 mi. E of Tacna, 3/6/1988, R.E.Woodruff, blacklight trap (7, FSCA); AZ, Yuma Co., Aztec, 7/7/1957, Stange & Harding, (2, FSCA); AZ, Yuma Co., San Cristobal Dunes, Goldwater Range, 5/2-4/1997, Olson,Schwalbe et al, UV (3, UAIC); AZ, La Paz Co., Ehrenberg, 3/31/2001, BC & WB Warner, Bill Warner, (4, ); AZ, Yuma Co., Dateland, 6/14/1964, A.G.Raske, (1, EMEC); AZ, Yuma Co., Yuma, 6/11/1937, D.J. & J.N.Knull, (1, OSU); AZ, Yuma Co., Yuma, 1/6/1967, R.S.Funk, at light, (1, NAU); AZ, Yuma Co., Yuma, 3/23/1965, R.S.Funk, (1, NAU); AZ, Yuma Co., Yuma desert 9 mi. E of San Luis, 3/18/1980, Werner,Olsen,Metz & MacLachlan, (2, UAIC); AZ, Yuma Co., Yuma, 4/29/1959, D.Muse, at light, (1, UAIC); AZ, Yuma Co., 10 mi. E of Tacna, N end of Mohawk Dunes, 3/30/2001, WB & BC Warner, Bill Warner, (2, ); AZ, Yuma Co., 10 mi. E of Tacna, N end of Mohawk Dunes, 3/21/1997, WB & BC Warner, night (5, WB Warner ); AZ, Yuma Co., N end of Mohawk Dunes 0.2 mi. N of BMG Range, 4/1/1994, Warners, (2, WB Warner); AZ, Yuma Co., 10 mi. E of Tacna, Mohawk Dunes, 3/20/1998, 32.41.8N 113.47.4W, B & W Warner, Bill Warner, (2, ); AZ, Yuma Co., 7.5-8 mi. E Tacna, sand dunes, 3/21/1998, 32.41.8N 113.48.4W, B & W Warner, Bill Warner, (2, ); AZ, Yuma Co., Mohawk Dunes 10 mi. E of Tacna, 6/4/2010, 32.41.45N 113.47.22W, WB Warner, Bill Warner, UV (1, ); AZ, Yuma Co., 6 mi. SE of Parker, 7/9/1966, Davidson & Cazier, (2, ASUT); AZ, Yuma Co., Goldwater Mil Rge, San Cristobal Dunes, 3/3/1997, CA Olson, pitfall traps (2, UAIC); AZ, Yuma Co., Goldwater Mil Rge, San Cristobal Dunes, 3/2/1997, CA Olson, UV (1, UAIC); AZ, Yuma Co., Yuma, 3/29/1960, D.Muse, at light, desert (1, UAIC); AZ, Yuma Co., Yuma, 5/5/1973, Walker, at night (1, ASUT); AZ, 22 mi. E of San Luis, 4/16/1960, (1, USNM); AZ, Yuma Co., Yuma, 3/12/1956, G.Lorenz, (1, UAIC); AZ, Mohave Co., Sacramento Wash at Franconia Sta. 0.4 mi. N of I40, 7/28/1989, 1200’, J.P.Donahue, #137,707 (1, LACM); AZ, Yuma Co., Yuma, 4/26/1935, J.D.Ball, (1, UAIC); AZ, Yuma Co., Yuma, 5/14/1959, D.Muse, at lights (1, UAIC); AZ, 5 mi. W of Bouse, 8/1/1957, E.R.Tinkham, dunes (2, USNM); AZ, (1, USNM); AZ, Yuma Co., Yuma, 5/15/1939, T.G.Aitken, ex coll. M.A.Cazier (1, AMNH); AZ, Ehrenberg, Colorado River, Palmer, *Arenivaga apacha* (Sauss.) Hebard Collection (1, ANSP); AZ, Pima Co., Ajo, 7/23/1938, R.I.Sailer, (1, ANSP); AZ, Mohave (now La Paz) Co., 3 mi. SE of Parker, 6/28/1978, J.Powell, black light trap (2, EMEC); NV, Nye Co., Big Dune, 4/19/1976, D.Giuliani, (1, CSCA); NV, Nye Co., Big Dune, 4/29/1974, 2500’, R.Hardy, (3, CSCA); NV, Nye Co., Big Dune, 4/28/1975, Andrews & Hardy, cereal bowl pit trap under (2, CSCA); NV, Nye Co., Carrara, 4/18/1969, F.G.Andrews, (1, CSCA); NV, Clark Co., 10 mi. NE of Las Vegas, 4/27/1975, Andrews & Hardy, sand dune association (1, CSCA); NV, Nye Co., Pahrump, 7/7/1959, D.F.Zoller, (1, FSCA); NV, Nye Co., Pahrump, 7/4/1959, F.D.Parker, light trap (1, FSCA) NV, Mercury, 8/6/1964, 5M(TB) (1, USNM); NV, Nye Co., Pahrump, 6/?/1959, DF Zoller, 1 specimen-*Arenivaga apacha* (Sauss.) det. HF Strohecker (6, NV Dept. of Ag); NV, Nye Co., Pahrump, 7/7/1959, DF Zoller, (1, NV Dept. of Ag); NV, Nye Co., Pahrump, 7/29/1959, FD Parker, (1, NV Dept. of Ag); NV, Nye Co., Pahrump, 8/4/1959, FD Parker, light trap (2, NV Dept. of Ag); NV, Nye Co., Pahrump, 8/2/1959, FD Parker, light trap (1, NV Dept. of Ag); NV, Nye Co., Pahrump, 7/30/1959, FD Parker, light trap (1, NV Dept. of Ag); NV, Lincoln Co., Ash Springs, 6/20/1966, 2400 ft., RW Lauderdale, (1, NV Dept. of Ag); NV, Nye Co., Big Dune, 5/16/1982, RC Bechtel & RW Rust, 3 specimens-black light trap (6, NV Dept. of Ag); NV, Nye Co., Big Dune, 4/29/1982, RC Bechtel & RW Rust, black light trap (2, NV Dept. of Ag); NV, Clark Co., Glendale, 8/3/1961, RC Bechtel, (1, NV Dept. of Ag); NV, Nye Co., Lava Dune, 5/15/1982, RC Bechtel & RW Rust, black light trap, T14S R49E (1, NV Dept. of Ag); NV, Nye Co., Amargosa Dune, 4/5/1994, JLP RWR, (1, NV Dept. of Ag); CA, Brawley, 4/7/1925, *Arenivaga erratica* Rehn det. Caudell (1, NV Dept. of Ag); CA, Riverside Co., Palm Springs, 5/29/1939, B Brookman, (1, NV Dept. of Ag); CA, Riverside Co., Snow Creek Game Refuge, 8 mi W Palm Springs, 6/27/1956, LD Moore, (3, MLBM); CA, Riverside Co., Sand dunes near Palm Springs, 5/29/1954, AH Barnum, (7, MLBM); CA, Ocotillo Wells, 3/18/1966, DR Estes, (7, FS Ento.); CA, Riverside Co., Mecca, 8/?/1955, LD Moore, light trap (2, MLBM); CA, San Diego Co., Borrego Springs, 9/3/1982, TB Moore, *Arenivaga apacha* (Saussure) det. AH Barnum 2005 (1, MLBM); CA, Riverside Co., Mecca, 4/27/1960, LD Moore, light trap (1, MLBM); CA, Riverside Co., Mecca, 6/1/1960, LD Moore, light trap (1, MLBM); CA, Riverside Co., Mecca, 7/20/1960, LD Moore, light trap (1, MLBM); CA, Riverside Co., Thermal, 9/11/1970, LD Moore, (1, MLBM); CA, Riverside Co., Mecca, 6/21/1955, LD Moore, (2, MLBM); CA, Riverside Co., Thermal, 6/21/1955, LD Moore, 1 specimen-light trap (2, MLBM); CA, Riverside Co., LaQuinta, 6/?/1968, LD Moore, light trap (2, MLBM); CA, San Bernardino Co., 2.5 mi W Amboy Crater on road, 3/5/1988, MM Fuller, 0.5 mi S of National Trails Hwy. in lava field at night, attracted to flashlight. (1, MSB); AZ, Mohave Co., 3 mi SE Parker, 6/28/1978, J Powell, black light trap (2, EMEC); AZ, Maricopa Co., Gila River at Airport Rd., 4/29-5/19/2011, 33.21.06N, 112.30.13W, Bill Warner, barrier pitfall traps on river sand (3, WB Warner); AZ, Maricopa Co., Gila River at Airport Rd., 4/12-14/2011, 33.21.06N, 112.30.13W, Bill Warner, human dung baited pitfall traps (2, WB Warner); AZ, Maricopa Co., Gila River at Airport Rd., 4/14-22/2011, 33.21.06N, 112.30.13W, Bill Warner, barrier pitfall traps on river sand (2, WB Warner). MEXICO: Sonora, La Choya, 6/12/1952, Cazier,Gertsch & Schrammel, (7, AMNH); Sonora, 20 mi. SE of San Luis RC, 6/6/1959, DH Tuttle, (2, USNM); Sonora, coastal dunes, Puerto Penasco, 6/23/1957, ER Tinkham, (3, USNM); Sonora, 6 mi. E of Punta Penasco, 6/22/1957, ER Tinkham, (7, USNM); Sonora, Punta Penasco, 4/10/1963, FG Andrews, (1, LA State College); Sonora, Punta Penasco, 6/23/1957, ER Tinkham, (2, USNM); Sonora, 15 mi. N of Puerto Penasco, 7/21/1973, F Werner, at light (1, UAIC); Sonora, Punta Penasco, 10/1/1989, G Simmons, (1, UAIC); Sonora, Rocky Point, 4/16/1965, JW Wienko, (1, UAIC); Sonora, El Gulfo de Santa Clara, 3/16/1958, V Roth, (1, UAIC); Sonora, Cholla Bay, 4/18/1959, A Ross, on ground (2, UAIC); Sonora, Rocky Point, 2/17/1934, (3, SDMC); Sonora, Rocky Point, 7/27/1956, C & M Cazier, (6, AMNH); Sonora, Puerto Penasco, 6/11/1952, Cazier,Gertsch & Schrammel, (1, AMNH); Sonora, 47 mi. W of Sonoyta, 8/9/1957, ER Tinkham, (1, USNM); Sonora, San Luis, 5/24/1938, (4, CAS); Sonora, Laguna Prieta, 5/25/1938, (3, CAS); Sonora, San Luis, 5/24/1938, (1, LACM); Sonora, E San Luis, 10/6/1953, Ruckman,Let & Ames, (1, USNM); Sonora, Dunes 22 mi. E of San Luis, 6/1/1958, 150 ft., ER Tinkham, (1, USNM); Sonora, Cabin 245 Choya Bay, 9/8/1968, BL Burch, (1, ASUT); Sonora, Choya Bay, 7/30/1969, M Kolner, at light (2, ASUT); BC, 36 mi. N of San Felipe, 4/18-21/1961, FS Truxel, (2, LACM); BC, San Felipe, 2/21-26/1971, Santa Barbara Malacological Society (1, CAS); BC, 23.9 km W of Mexicali on Hwy. 2, 4/13/1979, DB Weissman, #79-45 (2, CAS); BC, San Felipe, 5/18/1963, JC Ball, (4, UCRC); BC, 89 km S of Mexicali on Hwy. 5, 7/30/1978, Weissman & Lightfoot, #78-109 (1, CAS); BC, 88.3 km S of Mexicali on Hwy. 5, 9/17/1979, Weissman,Lightfoot & Love, 79-163 (1, CAS); BC, 10.3 mi. SW of Los Medanos, 3/27/1964, Irwin & Ball, at light on sand dunes (3, UCRC); BC, 4.9 mi. SW of Algodones, 3/25/1986, 32.48.734N 114.48.234W, RH McPeak, black light (1, EMEC); BC, San Felipe, 3/25/1963, GI Stage, (1, HEH ); BC, Rancho Potrero, 5/7-8/1959, D Patterson, (1, USNM); BC, 5 mi. N of San Felipe, Playa del Sol camp, 6/16/1973, Williams & Blair, SCW #309(2) (1, CAS); Sonora, Puerto Penasco, 7/?/1960, AH Barnum, (1, MLBM). Determiner label *Arenivaga investigata* Hopkins 2011” [white label with black border].

##### Distribution.

This species is found from northern Baja California and northwestern Sonora, Mexico northwards through southeastern California, southwestern Arizona, and southern Nevada. See Fig. 98.

##### Diagnosis.

*Arenivaga investigata* may be diagnosed by the single spine on the right ventral phallomere. See Fig. 97.

##### Description.

Male. NB: Holotype is destroyed by dermestid beetles. Neotype designated and described here. *Measurements*. Holotype TL = 18.3 mm, GW = 11.3 mm, PW = 8.00 mm, PL = 5.49 mm, TL/GW = 1.62, PL/PW = 0.69. EW = 0.70 mm; OW = 0.70 mm. Among paratypes range of TL 15.0–24.8 mm; range of GW 6.5–11.5 mm; range of PW 4.73–8.13 mm; range of PL 3.73–5.82 mm.

**Figure 96.**
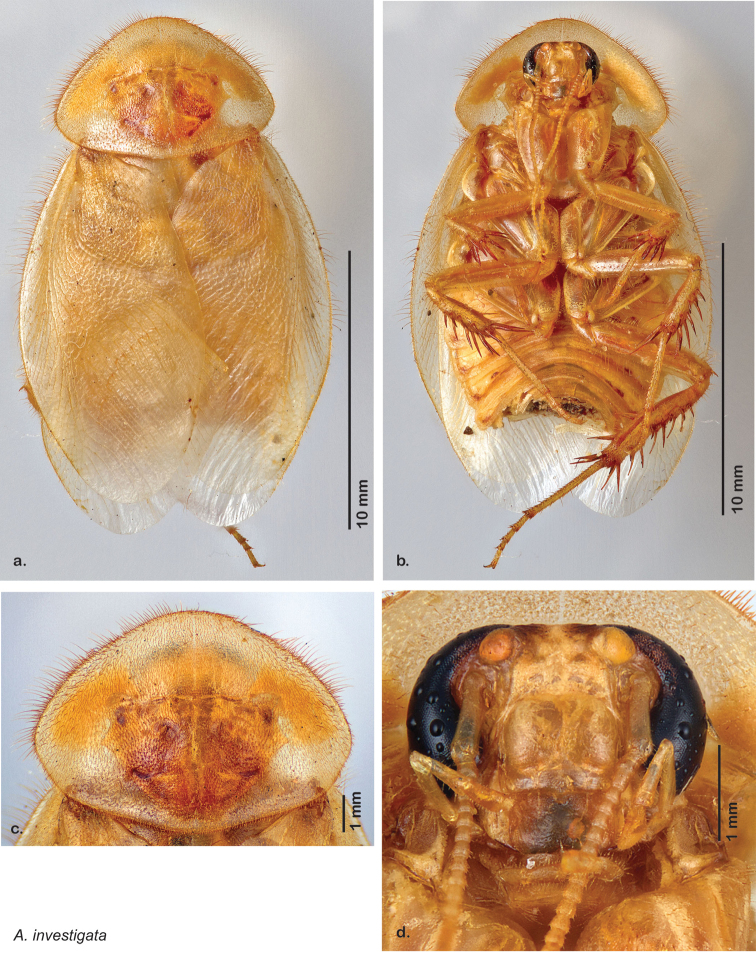
*Arenivaga investigata* **a** dorsal habitus **b** ventral habitus **c** pronotum **d** head.

*Head*. Two ocelli large, ovoid and protruding (0.40 × 0.35 mm); vertex light orange-brown, with darker small ridges between apices of eyes extending on to ocellar tubercles, scattered short setae; interocellar space slightly concave, light orange-brown. Frons light orange-brown, concave; bound on either side by ridges extending from inner apex of ocelli outwards to lateral edges of clypeus; scattered long setae on ridges. Anterior portion of frons light orange-brown, very bulbous; clypeal suture demarcates light orange-brown anteclypeus. See Fig. 96d.

*Pronotum*. Pronotum translucent waxy beige; variable length orange-brown setae along anterior margin; dorsal surface of pronotum covered with short orange-brown setae that are denser and longer anteriorly and laterally; pronotal pattern variable in color from light orange-brown through every shade to medium brown, “panther face”, no aura, usually discernible detail. See Fig. 96c.

*Body*. Wing brace present. Legs and body medium orange-brown; subgenital plate strongly asymmetrical with posterior edge emarginated, rounded apices. See Fig. 96b.

*Forewings*. Wings extended beyond abdominal apex a great distance in some specimens, but only a short distance in others; color, like size, highly variable from pale with no blotches through medium brown with darker blotches; surface opaque and matte. See Fig. 96a.

**Figure 97.**
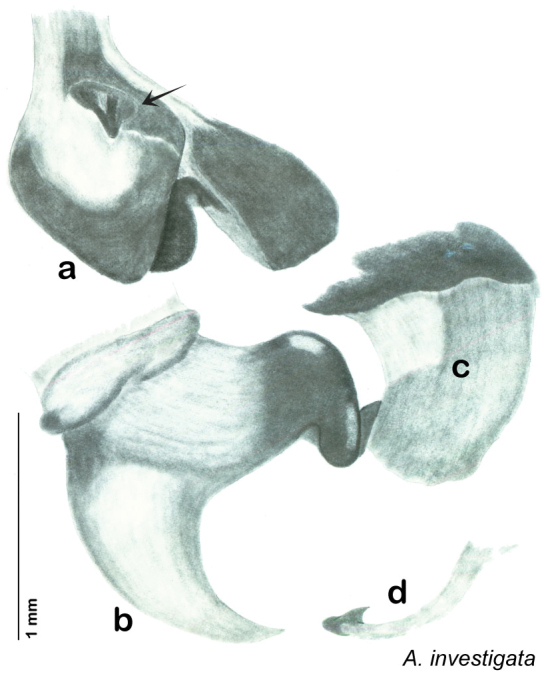
*Arenivaga investigata*, genitalia: a) right dorsal phallomere **b** right ventral phallomere **c** small central sclerite **d** genital hook. Arrow(s) indicate diagnostic characters (see text).

*Genitalia*. Right dorsal phallomere composed of bulbous lightly sclerotized narrow ended hook-shaped lobe, articulated with right ventral phallomere on lateral side; central field lightly sclerotized; medial margin sclerotized, smooth, concave in ventral view; medial margin smoothly curved at anterior end, shagreened greatly extended knob at posterior end with dorsally pointing thick blunt spine. Small central sclerite smooth, concave, with field of punctations on interoventral surface. Right ventral phallomere extends from articulation smooth bulbous lobe with posteromedial pointing spine that may have narrow or broad base; recedes anteriorly to collar-like rim; after moderate gap, long, narrow, flanged, concave arm, extending to greater depth than rest of phallomere. Folded anterior portion of left phallomere moderately wide, finely punctate and setose. Genital hook with long pointed head and depression along short hook; arm smoothly curving. See Fig. 97.

**Figure 98.**
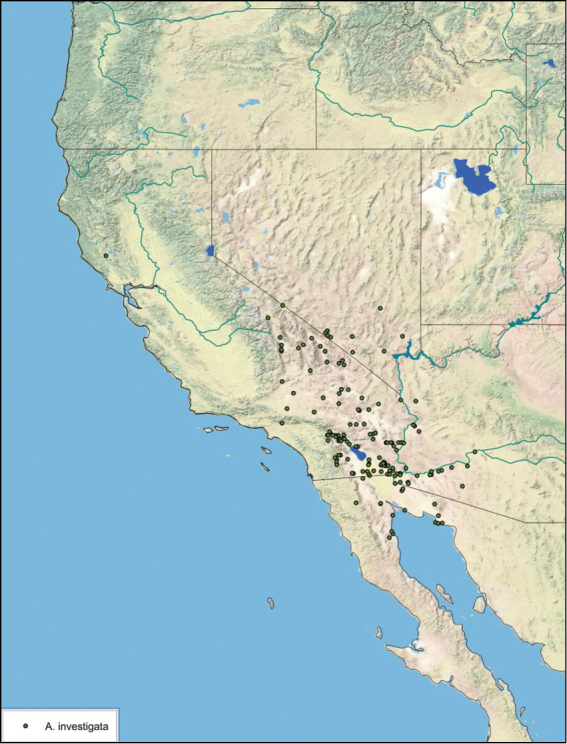
*Arenivaga investigata*, distribution.

##### Habitat and natural history.

All life history elements remain unobserved.

#### Arenivaga mckittrickae sp. n.

http://zoobank.org/0D018D80-7DA6-46A6-A669-AB1AECB6377D

http://species-id.net/wiki/Arenivaga_mckittrickae

Figures 99 – 101

##### Type locality.

USA, California, San Bernardino Co., UC Burns Reserve.

##### Material examined.

Holotype: ♂ in UCRC labeled “CA: San Bernardino Co, Pioneertown, 1350 m, UC Burns Reserve, 25 June 1995, J. Freilich, 37-78N 5-50E” “HOLOTYPE *Arenivaga mckittrickae* Hopkins, 2012” [red label with black border].

Paratypes (24): USA: CA, Los Angeles Co., Mint Canyon, 9/4/1946, P Greeley (3, LACM); CA, Los Angeles Co., Mint Canyon, 8/15/1946, 2000 ft., P Greeley, *Arenivaga* (1, LACM); CA, Los Angeles Co., Mint Canyon, 7/1/1946, 2000 ft., P Greeley (1, LACM); CA, Wrightwood, San Gabriel Mts., 6/26/1986, GH Nelson, Merc.Vap.Lite (3, FSCA); CA, San Bernardino Co., Mojave River Forks, 10 km SE of Hesperia, 7/11/1986, (3, UCRC); CA, San Bernardino Co., 4 mi. N of Cajon Pass, 6/12/1966, Middlekauff & Rentz (1, EMEC); CA, San Bernardino Co., Yucca Valley, Skyline Rd., UC Burns Res., 9/17/1994, W Sakai, black light (1, UCRC); CA, San Bernardino Co., San Bernardino Mts., Cushionberry Grade, 7/8/1986, GF Pratt (1, UCRC); CA, San Bernardino Co., Pioneertown, UC Burns Res., 7/9/1994, J Frellich (1, UCRC); CA, Los Angeles Co., Bouquet, 7/23/1937, N Westerland (2, LACM); CA, Los Angeles Co., Bouquet Canyon, 6/23/1937, N Westerland, at lights (1, LACM); CA, Sierra Madre, 4/10/1940 (2, LACM); CA, San Mateo (1, LACM); CA, Riverside Co., Joshua Tree NM, 9/22/1979, CD Nagano, U.Covington Flat (1, LACM); CA, San Bernardino Co., Pioneertown, UC Burns Res., 8/30/1995, 1350 m, J Frellich, 37-78N 5-50E (1,UCRC). All paratypes labeled “Paratype *Arenivaga mckittrickae* Hopkins 2012” [blue label with black border].

##### Etymology.

The name is a noun in the genitive case. This species is named for F.A. McKittrick, author of the incomparable “Evolutionary Studies of Cockroaches”.

##### Distribution.

This species is distributed from Mint Canyon, Los Angeles Co., in its northern and western extents to Joshua Tree NM, Riverside Co., in its southern and eastern extents. Its distribution follows the San Gabriel Mountain range of southern California. One specimen labeled “San Mateo” may be mislabeled or transported to that locality. See Fig. 101.

##### Diagnosis.

*Arenivaga mckittrickae* is distinguished by the sinuous medial margin and narrow hook-shaped lobe on the right dorsal phallomere as well as the unusually shaped head on the genital hook. See Fig. 100.

##### Description.

**Male.** *Measurements*. Holotype TL = 18.4 mm, GW = 7.6 mm, PW = 4.98 mm, PL = 3.58 mm, TL/GW = 2.42, PL/PW = 0.72. EW = 0.3 mm; OW = 0.45 mm. Among paratypes range of TL 16.9–19.8 mm; range of GW 7.4–9.3 mm; range of PW 5.10–5.79 mm; range of PL 3.20–4.06 mm.

**Figure 99.**
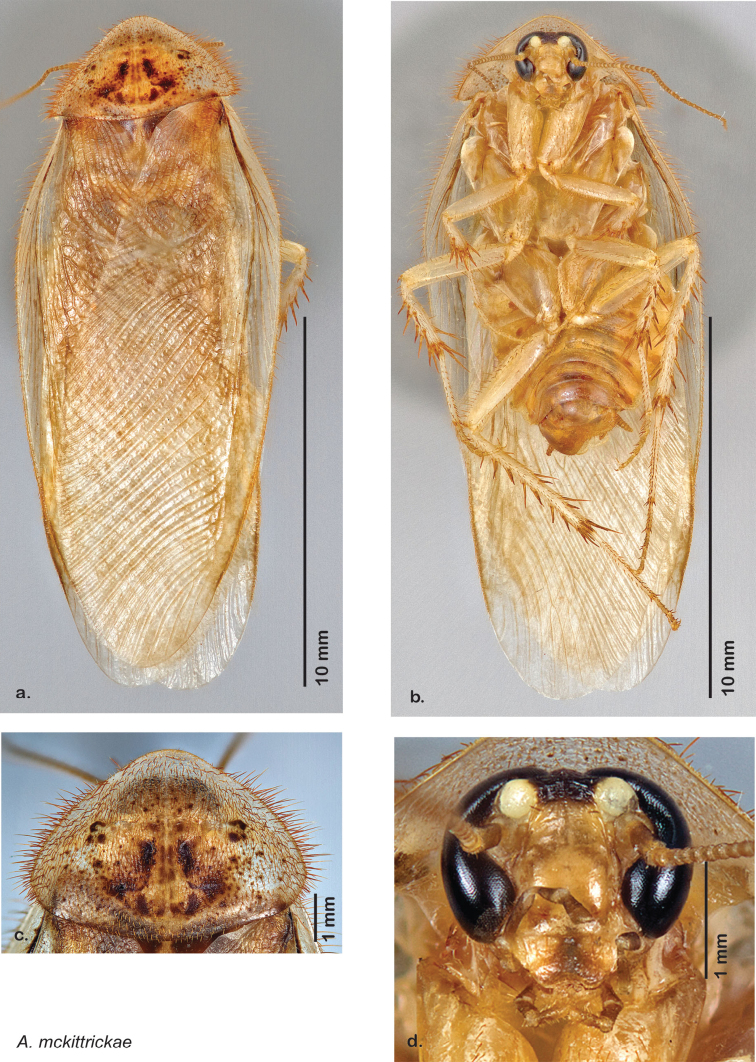
*Arenivaga mckittrickae* **a** dorsal habitus **b** ventral habitus **c** pronotum **d** head.

*Head*. Two ocelli large, ovoid and protruding (0.40 × 0.30 mm); interocular space mildly convex and dark brown with small ridges in rays around upper apex of eyes and extending onto ocellar tubercles, interocellar space concave, with faint horizontal corrugations. Posterior frons concave, uniformly light brown; anterior frons bulbous with slight central indentation at posterior end; broad, flat, light brown anteclypeus. See Fig. 99d.

*Pronotum*. Pronotum translucent, waxy beige; dorsal surface of pronotum with short fine brown setae centrally and posteriorly grading to longer, thicker setae laterally and anteriorly; pronotal pattern brown “panther face”; some detail discernible; brown maculations scattered across posterior 70% of dorsal surface of pronotum. See Fig. 99c.

*Body*. Wing brace present. Two tarsal claws present. Legs and body light orange-brown, with darker maculation laterally on each sternite; subgenital plate dark orange-brown; strongly asymmetrical with rounded apices. See Fig. 99b.

*Forewings*. Wings extended well beyond abdominal apex (~50% of wing length); color varies from uniform brown, to blotchy brown, to light orange-brown; surface ranges from glossy translucent to matte opaque. See Fig. 99a.

**Figure 100.**
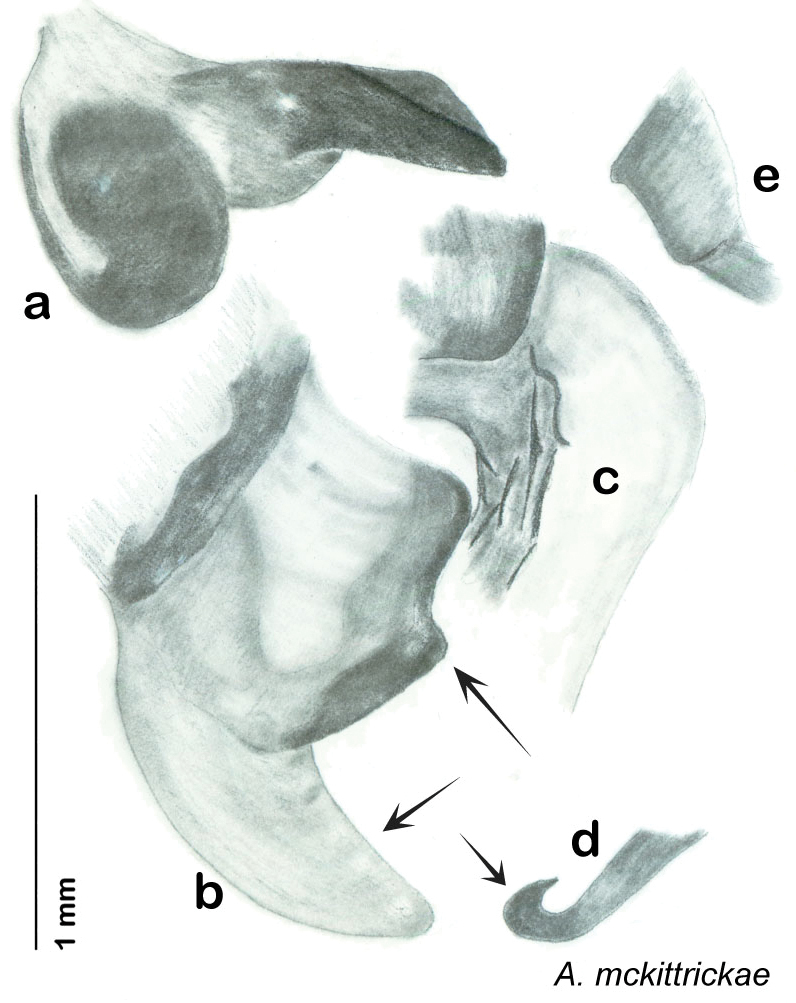
*Arenivaga mckittrickae*, genitalia: a) right dorsal phallomere **b** right ventral phallomere **c** small central sclerite **d** genital hook **e** left phallomere. Arrow(s) indicate diagnostic characters (see text).

*Genitalia*. Right dorsal phallomere composed of bulbous sclerotized hook-shaped lobe, articulated with right ventral phallomere on lateral side; central field lightly sclerotized; increasingly punctate towards rim; rim shagreened; inner rim forms sweeping S-curve with added straight edge posteriorly; phallomere otherwise unmodified. Small central sclerite rugose, punctate, with posteriorly projecting, more sclerotized lip at anterior end; right ventral phallomere extends from articulation to form rounded shagreened structure with small interiorly directed nipple; attached anteriorly is mildly dorsally projecting flanged arm, shagreened with roughly toothed edge. Folded anterior portion of left phallomere with lightly sclerotized indentation, otherwise unmodified. Genital hook with blunt rounded head with short hook; arm robust and slightly shorter than usual in *Arenivaga*. See Fig. 100.

**Figure 101.**
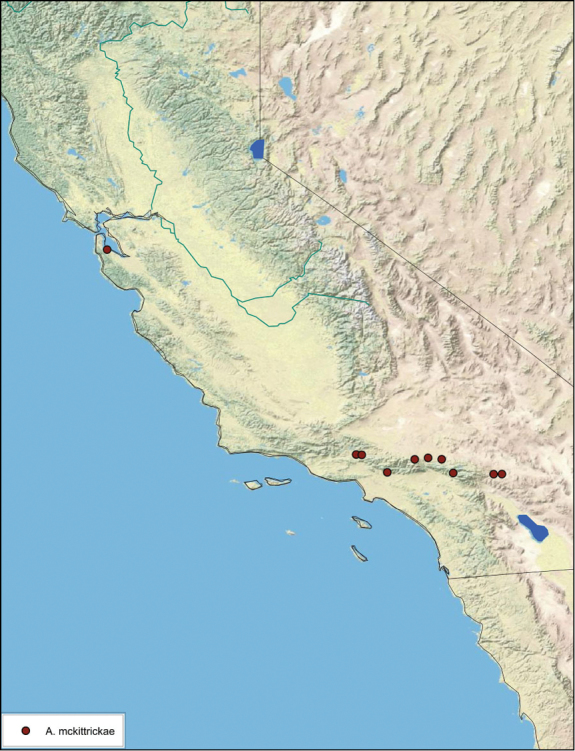
*Arenivaga mckittrickae*, distribution.

##### Habitat and natural history.

All life history elements remain unobserved.

#### Arenivaga milleri sp. n.

http://zoobank.org/E197CE33-847B-43C1-A982-691D8DF7732A

http://species-id.net/wiki/Arenivaga_milleri

Figures 102 – 104

##### Type locality.

USA, California, Mono Co., Benton.

##### Material examined.

Holotype: ♂ in FSCA labeled “Benton, Mono Co., Calif., VII-20-50, H. A. Hunt Collector” “HOLOTYPE *Arenivaga milleri* Hopkins, 2012” [red label with black border].

Paratypes (29): USA: CA, Mono Co., Benton, 7/16/1972 (7, CSCA); CA, Mono Co., Benton, 8/10/1972 (3, CSCA); CA, Mono Co., Benton, 7/10/1940, JG Shanafelt (3, LACM); CA, Mono Co., Benton Station, 6/10/1940, JG Shanafelt (2, LACM); CA, Mono Co., Benton, 8/5/1940, JG Shanafelt (3, LACM); CA, Mono Co., 4 mi N of Benton, 6/12-8/31/1980, 5600 ft., D Giuliani, antifreeze pit trap (1, CSCA); CA, Inyo Co., Fish Lake Valley, 6 mi S and 4 mi E of Oasis, 10/20/1983-6/1/1986, 5200 ft., D Giuliani, antifreeze pit trap (2, CSCA); CA, Inyo Co., 8/1/1922, OC Poling (1, ANSP); CA, Inyo Co., Westguard Pass, White Mts., 7/19/1968, J Scott, at light (1, EMEC); NV, Mercury, 8/9/1964, 12M(TB) (2, USNM); NV, Churchill Co., Blow Sand Mts., T15N, R30E, 8/2/1979, RC Bechtel, LM Hanks, DL Horton & RW Rust, Black Light Trap, 1 specimen-*Arenivaga erratica* Rehn det. RC Bechtel ‘80 (2, NVDA); NV, Churchill Co., Blow Sand Mt. 28 mi SSE Fallon, 8/3/1979, RW Rust, *Arenivaga erratica* Rehn det. RC Bechtel ‘80 (1, NVDA); NV, Washoe Co., Reno, 7/26/1982, JB Knight (1, NVDA). All paratypes labeled “Paratype *Arenivaga milleri* Hopkins 2012” [blue label with black border].

##### Etymology.

The name is a noun in the genitive case. This species is named for my PI Dr. Kelly Miller, an outstanding systematist, patient PI and friend, in grateful appreciation for giving the lover of cockroaches a place in your beetle lab.

##### Distribution.

This species is found in the Mohave Desert along the California-Nevada border and northwards. An isolated specimen from northwest California is probably mislabeled or transported. See Fig. 104.

##### Diagnosis.

*Arenivaga milleri* may be confused with *Arenivaga belli* but is distinguished by the lack of a spine on the medial margin of the right dorsal phallomere. See Figs 103 and 31.

##### Description.

**Male.** *Measurements*. Holotype TL = 17.9 mm, GW = 10.7 mm, PW = 6.05 mm, PL = 3.70 mm, TL/GW = 1.67, PL/PW = 0.61. EW = 0.40 mm; OW = 0.40 mm. Among paratypes range of TL 17.2–20.0 mm; range of GW 7.45–10.7 mm; range of PW 5.37–6.05 mm; range of PL 3.54–4.07 mm.

**Figure 102.**
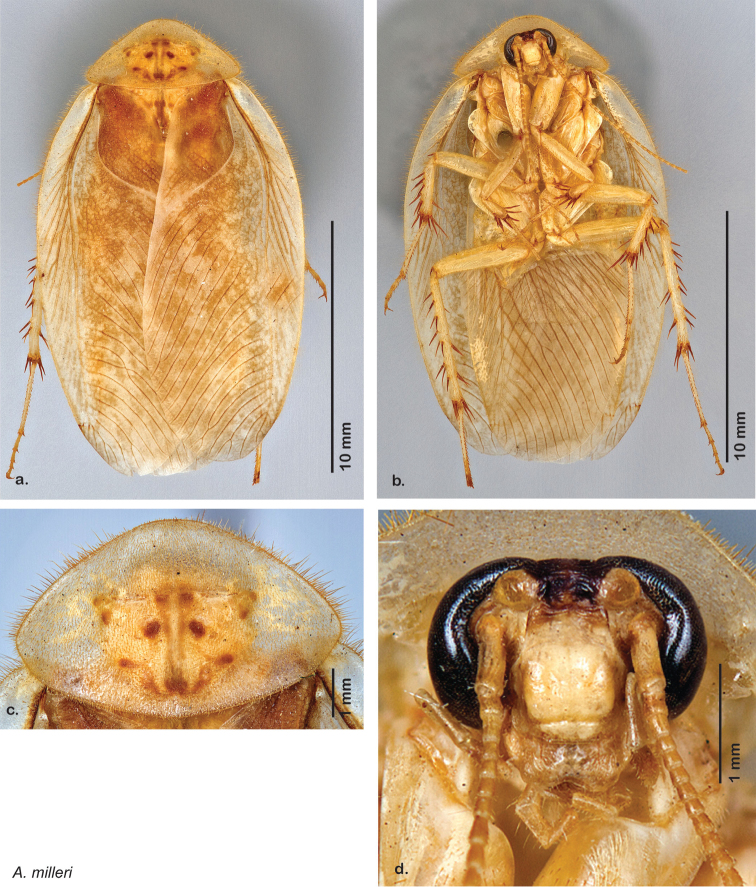
*Arenivaga milleri* **a** dorsal habitus **b** ventral habitus **c** pronotum **d** head.

*Head*. Two ocelli very large, ovoid and protruding (0.4 × 0.25 mm); vertex brown with small ridges in rays around upper apices of eyes and extending onto ocellar tubercles; interocellar space deeply concave, smooth, brown fading to light brown at intersection with frons with two light brown circular indentations at base of interocular space. Posterior frons light brown fading quickly to waxy white, mildly concave with horizontal corrugations on brown portion, anterior portion of frons bulbous and waxy white; waxy white smooth anteclypeus. See Fig. 102d.

*Pronotum*. Pronotum translucent, waxy beige; dorsal surface of pronotum densely setose with fine light brown setae; pronotal pattern light orange-brown “panther face” with moderate detail discernible; no aura. See Fig. 102c.

*Body*. Wing brace present. Two tarsal claws present. Legs and body light brown; subgenital plate light brown with orange-brown margin; asymmetrical with rounded apices. See Fig. 102b.

*Forewings*. Wings extended well beyond abdominal apex (~30% of wing length); color light brown to medium brown depending on specimen and blotchy; surface matte and opaque. See Fig. 102a.

**Figure 103.**
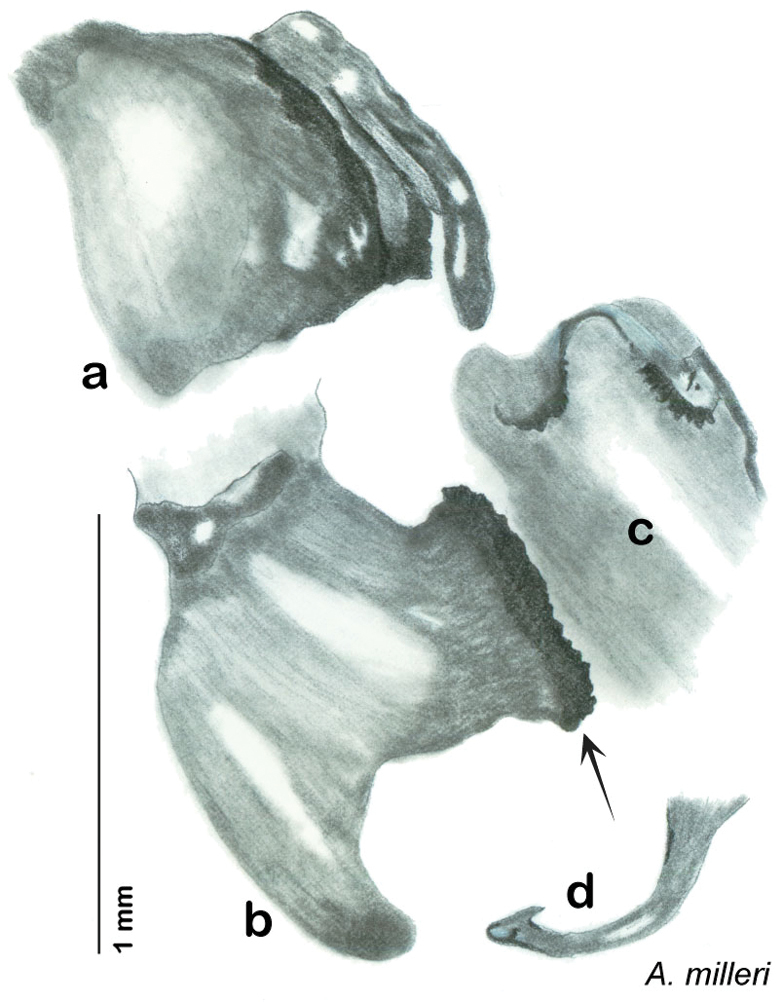
*Arenivaga milleri*, genitalia: a) right dorsal phallomere **b** right ventral phallomere **c** small central sclerite **d** genital hook. Arrow(s) indicate diagnostic characters (see text).

*Genitalia*. Right dorsal phallomere composed of bulbous lightly sclerotized hook-shaped lobe, articulated with right ventral phallomere on lateral side; central field lightly sclerotized; medial margin heavily sclerotized, shagreened, with slightly wavy toothed edge. Small central sclerite flat and finely punctate with posteriorly projecting, heavily toothed flanges dorsally and ventrally; right ventral phallomere extends from articulation to form structure rounded and punctate at posterior apex receding into narrower corrugations apically, followed by punctate rounded concave arm extending beyond depth of rest of phallomere. Folded anterior portion of left phallomere setose, otherwise unmodified. Genital hook with moderate extension to pointed head with very slight concavity on short hook; arm smoothly curving. See Fig. 103.

**Figure 104.**
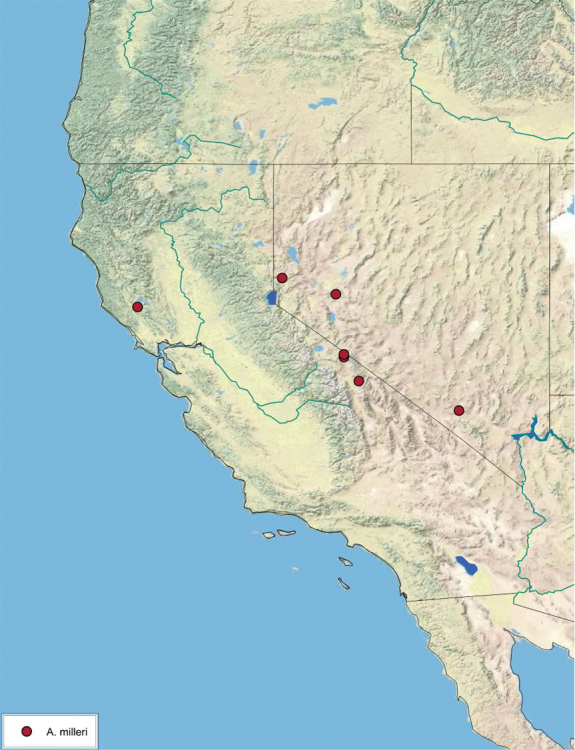
*Arenivaga milleri*, distribution.

##### Habitat and natural history.

All life history elements remain unobserved.

#### Arenivaga moctezuma sp. n.

http://zoobank.org/F550FA6F-0039-44DD-943A-882ED7C70A64

http://species-id.net/wiki/Arenivaga_moctezuma

Figures 105 – 107

##### Type locality.

MEXICO, Sonora, 4.3 mi E Moctezuma.

##### Material examined.

Holotype: ♂ in SDMC labeled “MEXICO: Sonora, 4.3 mi E Moctezuma, 18-21 July 1987, N. Bloomfield” “HOLOTYPE *Arenivaga moctezuma* Hopkins, 2012” [red label with black border].

Paratypes (3): MEXICO: Sonora, 4.3 mi E of Moctezuma, 7/18-21/1987, N Bloomfield (3, SDMC). All paratypes labeled “Paratype *Arenivaga moctezuma* Hopkins 2012” [blue label with black border].

##### Etymology.

The name is a noun in the genitive case. This species is named for the town Moctezuma, near which all known specimens originate.

##### Distribution.

This species is known only from the type locality. See Fig. 107.

##### Diagnosis.

*Arenivaga moctezuma* may be mistaken for *Arenivaga adamsi* but is distinguished by a smaller, glabrous spine on the left phallomere and a much smaller and simpler small central sclerite. See Figs 106 and 13.

##### Description.

**Male.** *Measurements*. Holotype TL = 18.1 mm, GW = 8.9 mm, PW = 5.70 mm, PL = 4.25 mm, TL/GW = 2.03, PL/PW = 0.75. EW = 0.50 mm; OW = 0.50 mm. No notable difference in size among paratypes.

**Figure 105.**
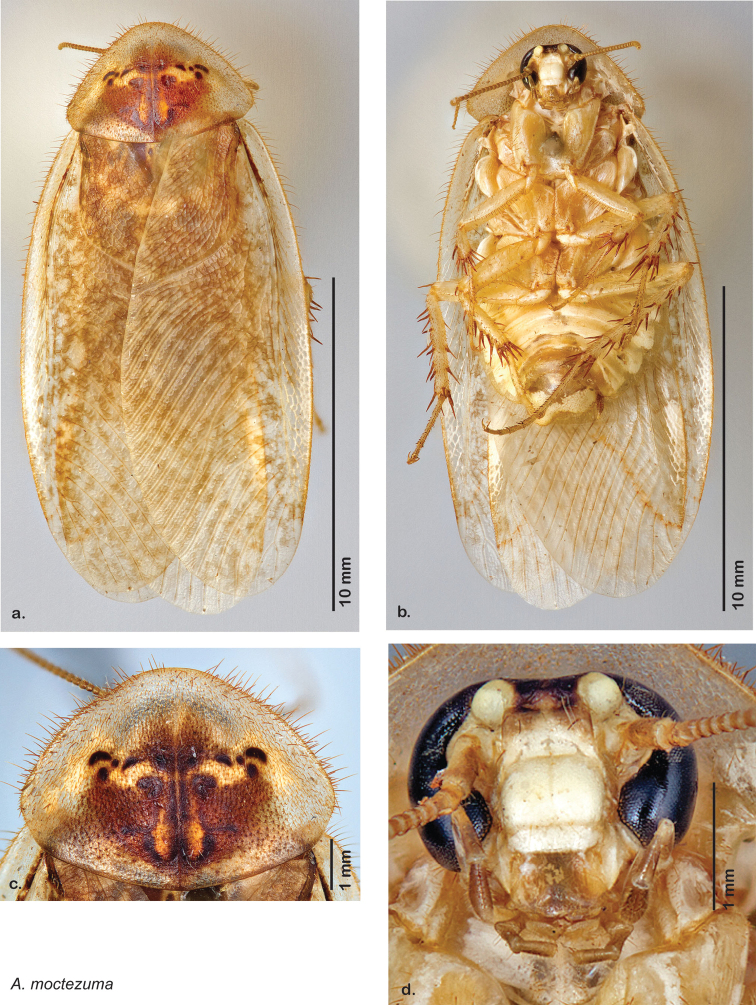
*Arenivaga moctezuma* **a** dorsal habitus **b** ventral habitus **c** pronotum **d** head.

*Head*. Two ocelli large, ovoid and protruding (0.40 × 0.30 mm); vertex dark brown with small ridges in rays around upper apices of eyes and extending onto ocellar tubercles; pale midline; interocellar space concave, medium brown, lighter anteriorly with three indentations at points of an equilateral triangle, top one round, bottom two eyebrow-shaped. Frons waxy white with brown edges near ocelli; posterior concave; anterior frons bulbous and waxy white; waxy white smooth anteclypeus. See Fig. 105d.

*Pronotum*. Pronotum translucent waxy beige; dorsal surface of pronotum with short orange-brown setae that are thicker and longer laterally; pronotal pattern dark orange-brown “panther face” with considerable detail discernible; lateral and anterior aura. See Fig. 105c.

*Body*. Wing brace present. Two tarsal claws present. Legs and body light brown; subgenital plate light brown; asymmetrical with rounded apices. See Fig. 105b.

*Forewings*. Wings extended well beyond abdominal apex (~35% of wing length); blotchy medium to dark brown; surface matte and opaque. See Fig. 105a.

**Figure 106.**
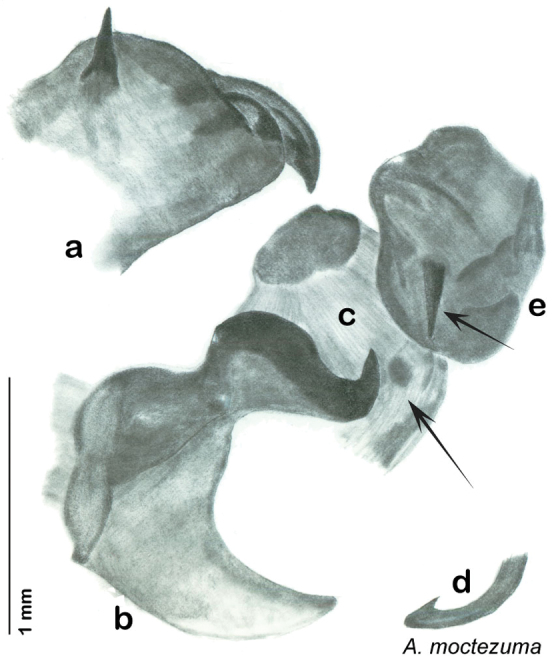
*Arenivaga moctezuma*, genitalia: a) right dorsal phallomere **b** right ventral phallomere **c** small central sclerite **d** genital hook **e** left phallomere. Arrow(s) indicate diagnostic characters (see text).

*Genitalia*. Right dorsal phallomere composed of lightly sclerotized, unusually curved, bulbous hook-shaped lobe, articulated with right ventral phallomere on lateral side; medial side of lobe deeply emarginated from medial edge of remainder of phallomere; central field deep, cupped, lightly sclerotized; medial margin wide, more heavily sclerotized, smooth, with long ventrally projecting spine and one dorsally projecting spine located midway along margin. Small central sclerite concave, punctate, with large shagreened medially projecting wide upside-down-V-shape on anterior edge; right ventral phallomere extends from articulation to form shagreened rounded structure, with prominent medially projecting two-prong spine located posteriorly; attached anteriorly is flanged punctate concave arm that extends slightly beyond depth of rest of phallomere, edge shagreened. Folded anterior portion of left phallomere dramatically modified with sclerotized punctate anterior wall and posteriorly projecting smooth spine located ventrally. Genital hook with moderate extension to pointed head with moderate hook; curve of arm reduced. See Fig. 106.

**Figure 107.**
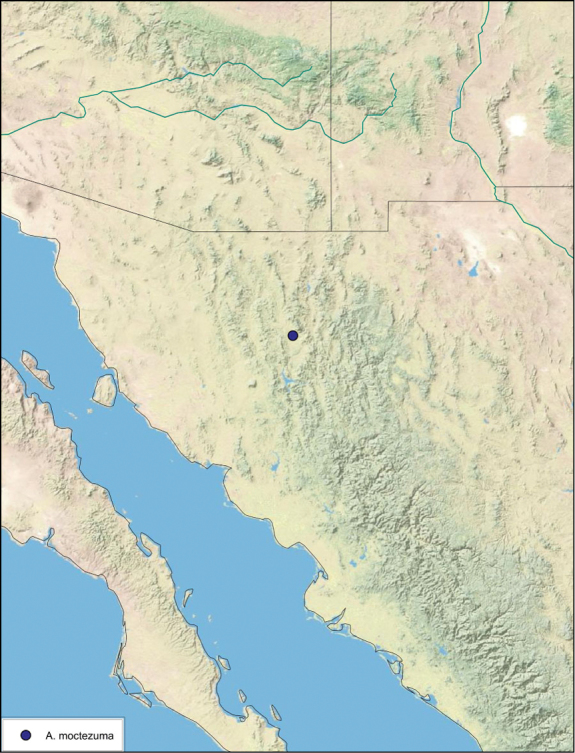
*Arenivaga moctezuma*, distribution.

##### Habitat and natural history.

All life history elements remain unobserved.

#### Arenivaga mortisvallisensis sp. n.

http://zoobank.org/3086A1CB-8F24-43DE-89B7-E9EF84621413

http://species-id.net/wiki/Arenivaga_mortisvallisensis

Figures 108 – 110

##### Type locality.

USA, California, Inyo Co., Death Valley, Sand Spring

##### Material examined.

Holotype: ♂ in CSCA labeled “CALIF: Inyo Co., Sand Spring 15.5 mi NW Scottys Castle, 3100’, II-87 to IX-1987, Antifreeze pit trap” “HOLOTYPE *Arenivaga mortisvallisensis* Hopkins, 2012” [red label with black border].

Paratypes: None at this time.

##### Etymology.

This species is named for its locality, Death Valley, CA.

##### Distribution.

This species is only known from one specimen from Death Valley NM, CA. See Fig. 110.

##### Diagnosis.

*Arenivaga mortisvallisensis* is smaller than average for *Arenivaga* but may be confused with *Arenivaga delicata* which has sympatric distribution. *Arenivaga mortisvallisensis* may be distinguished by the medial margin of the right dorsal phallomere that projects anteriorly in pronounced manner into rounded, shagreened lobe with toothed margin. See Figs 109 and 40.

##### Description.

**Male.** *Measurements*. Approximate due to poor condition of specimen. Holotype TL = 15.3 mm, GW = 6.2 mm, PW = 4.3 mm, PL = 3.24 mm, TL/GW = 2.47, PL/PW = 0.75. EW = 0.60 mm; OW = 0.40 mm.

**Figure 108.**
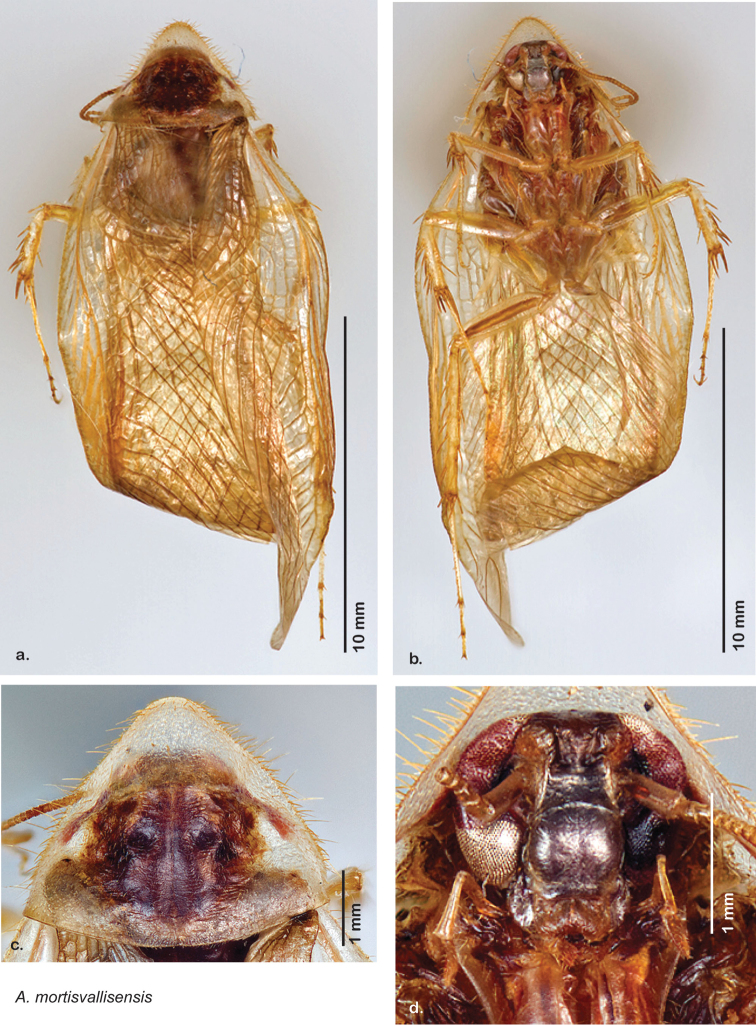
*Arenivaga mortisvallisensis* **a** dorsal habitus **b** ventral habitus **c** pronotum **d** head.

*Head*. Two ocelli large, ovoid and less protruding than on most species (0.3 × 0.2 mm); vertex unusually broad, dark brown with small ridges in rays around upper apices of eyes and extending onto ocellar tubercles; interocellar space concave, smooth, dark brown; two teardrop shaped indentations medial to ocelli. Frons very dark brown, posterior half flat, tectiform just below indentations; anterior portion of frons bulbous, very dark brown; very dark brown smooth anteclypeus. See Fig. 108d.

*Pronotum*. Pronotum translucent, waxy beige; dorsal surface of pronotum with short fine golden setae centrally and posteriorly grading to longer, thicker setae laterally and anteriorly; pronotal pattern dark brown “panther face”, impressed, with little discernible detail; no aura. See Fig. 108c.

*Body*. Wing brace present. Two tarsal claws present. Legs and body orange-brown; abdomen missing from specimen; species, or perhaps just this specimen, unique in its almost complete absence of deposits of uric acid, and those only very minimally in forewings. Subgenital plate cleared in KOH with genitalia therefore color uncertain; strongly asymmetrical with rounded apices. See Fig. 108b.

*Forewings*. Wings extended beyond abdominal apex though distance cannot be estimated as abdomen is missing; transparent pale brown with veins darker brown; surface hyaline. See Fig. 108a.

**Figure 109.**
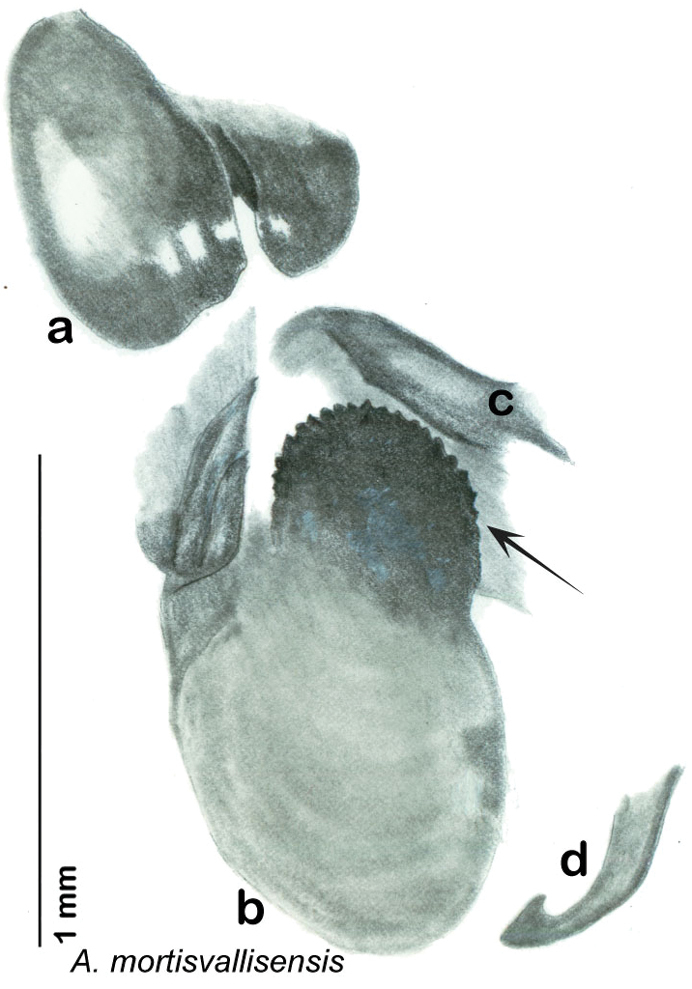
*Arenivaga mortisvallisensis*, genitalia: a) right dorsal phallomere **b** right ventral phallomere **c** small central sclerite **d** genital hook. Arrow(s) indicate diagnostic characters (see text).

*Genitalia*. Right dorsal phallomere composed of bulbous lightly sclerotized lobe, articulated with right ventral phallomere on lateral side; central field lightly sclerotized; medial margin strongly projected anteriorly into rounded, shagreened lobe with toothed margin. Small central sclerite of nondescript shape, finely punctate, mostly flat with ventrally bent rim slightly more sclerotize and densely punctate; right ventral phallomere extends from articulation to form rounded somewhat elongate smooth structure that becomes shorter and punctate anteriorly; attached anteriorly is mildly dorsally projecting flanged arm, shagreened with lightly toothed edge. Folded anterior portion of left phallomere setose, otherwise unmodified. Genital hook with short distance to pointed head with short hook; arm very delicate. See Fig. 109.

**Figure 110.**
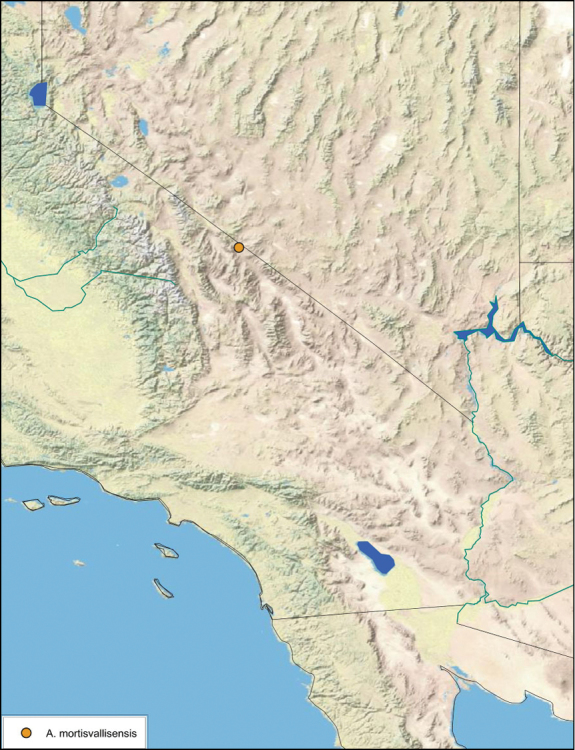
*Arenivaga mortisvallisensis*, distribution.

##### Habitat and natural history.

All life history elements remain unobserved.

#### Arenivaga nalepae sp. n.

http://zoobank.org/FEC7E11C-1FB4-48F3-BCE1-2BD1CD580790

http://species-id.net/wiki/Arenivaga_nalepae

Figures 111 – 113

##### Type locality.

USA, California, Riverside Co., Box Canyon.

##### Material examined.

Holotype: ♂ in LACM labeled “CALIF., Riverside Co., Box Canyon, Mecca Hills, 600 ft. el., 12 Sept. 1986, J P & KES Donahue” “HOLOTYPE *Arenivaga nalepae* Hopkins, 2012” [red label with black border].

Paratypes (73): USA: CA, Riverside Co., Box Canyon, Mecca Hills, 9/12/1986, 600 ft., JP & KES Donahue (14, LACM); CA, Imperial Co., Imperial Valley, near Wister, 10/27/1990, minus 75 ft., JP & KES Donahue, T9S R13E Sec.35, #149799 (1, LACM); CA, Riverside Co., Lamb Canyon, 2 mi. NW of Gilman Hot Springs, 3/7-11/27/1988, 1500 ft., FG Andrews, Pit trap (1, CSCA); CA, Riverside Co., Painted Canyon, 9/13/1979-1/7/1979, FG Andrews, E.glycol pit trap in desert wash (1, CSCA); CA, Riverside Co., Pinyon Flat, 8/5/1966, CA & MJ Tauber (1, EMEC); CA, Riverside Co., S side of Orocopia Mts., 3/25/1990, 900 ft., JP & KB Donahue,T7S R13B SW 1/4S.30, #2911 (1, LACM); CA, San Diego Co., Borrego Springs, 11/21/1958, JW Baker Jr., Black light trap, 58K25-1 (1, CSCA); CA, Riverside Co., Chiriaco Pass, 9/18/1971 (2, UCRC); CA, Imperial, 13 mi. NW of Glamis, 10/9/1993, 33.06.3N 115.15.3W, 250 ft., RR & C Snelling, black light (1, LACM); CA, Colton(?), 9/?/1949, O Cluh(?) (1, UCRC); CA, Fresno Co., Waltham Creek, 4 mi W of Coalinga, 8/28/1952, Leech & Green, dry bed (1, CAS); CA, San Bdno. Co., Cajon Wash, 8/4/40, 2000’, Collected by J. C. vonBloeker (1, LACM); CA, Los Angeles Co., Black Butte, Antelope Valley, 8/22/1959, G Sphon (3, LACM); CA, LA Co., Black Butte, Antelope Valley, 7/25/1959, G Sphon, one specimen genitalia incomplete (5, LACM); CA, Inyo Co., Dunmovin, 9/6/1948, SA Sher, *Arenivaga erratica* Rehn det. HF Strohecker 1953 (1, USNM); CA, Los Angeles Co., Whitehorn Picnic Area, Angeles NF, 8/22/1959, JA Honey (1, LACM); CA, Los Angeles, 6 mi. W of Lancaster, 10/3-5/1960, JA Chemsak (1, EMEC); CA, Kern Co., 8/29/1949, McKittrick, [one specimen missing head] (3, LACM); CA, Boron, 8/9/1959, J Helfer, black dot (1, USNM); CA, Los Angeles Co., Juniper Hills, 8/26/1973, A.V. Evans (1, LACM); CA, Kern Co., 4 mi NE of Mohave, 9/17/1966, TR Haig (6,CSCA); CA, Kern Co., Red Rock Canyon SP, Ricardo Ranger Station, 8/31-9/1/1991, 2700 ft., JP Donahue T9S R37E Sec.34,#24,431 (3, LACM); CA, Kern Co., Bakersfield, 4/6/1981, M Bock (1, LACM); CA, Kern Co., Bakersfield, 8/?/1954, R Smith, Cal.Dept.Agr.59H14-13, ex building (2,CSCA); CA, Fresno Co., Ciero Hills 18 air mi. SW of Mendota, 3/16/1975, J.T.Doyen, at light (2,EMEC); CA, Kings Co., Kettleman, 8/29/1972, L Bookout, *Arenivaga* sp. Det.AR Hardy 1972, Cal.Dept.Agr.37260, 7255-24, black light (2,CSCA); CA, Kern Co., near Buttonwillow, 9/27/1962, JR Anderson, ex burrow of Citellus beecheyi (1, EMEC); CA, Inyo Co., Saline Valley Salt Marsh, 1060’, 7/1/1976, D. Giuliani, collected at blacklight (1, CSCA); CA, Inyo Co., Inyo Mts., Lead Canyon, 9/2/1976, 6-6500 ft., D Giuliani, BLM Survey, Inyo Co. Saline Valley 1976 site 3 (3, LACM); CA, Inyo Co., Eureka Valley Dunes, 9/4/1975, D Giuliani (3, CSCA); CA, Inyo Co., Eureka Valley Dunes, 7/13/1975, Andrews & Hardy (1, CSCA); NV, Mercury, 8/14/1964, 1BB25M(T) (1, USNM); AZ, Yuma Co., nr. Tacna, on dunes, night, 12/16/2010, 32.696N, 113.79W, 148 m, AD Smith (3, HEH). MEXICO: BC, San Felipe, 6/15/1952, Cazier, Gertsch & Schrammel (1, AMNH). All paratypes labeled “Paratype *Arenivaga nalepae* Hopkins 2012” [blue label with black border].

##### Etymology.

The name is a noun in the genitive case. This species is named for Christine Nalepa, who loves cockroaches, encourages that love in others, and co-authored “Cockroaches: Ecology, Behavior and Natural History”, a book that had a profound effect on me.

##### Distribution.

This species is distributed from Saline Valley Salt Marsh in its northern and western extents to San Felipe, Baja California Norte, Mexico in its southern and eastern extents. See Fig. 113.

##### Diagnosis.

*Arenivaga nalepae* sp. n. is average in size and coloration for *Arenivaga*. It can be mistaken phenotypically for many other species, and its genitalia closely resemble that of *Arenivaga belli*, with whom it is probably closely related. The shape of the hook-shaped lobe on the right dorsal phallomere and the overall proportions of the right ventral phallomere are two distinguishing characters of this species. See Figs 112 and 31.

##### Description.

**Male.** *Measurements*. Holotype TL = 20.4 mm, GW = 8.8 mm, PW = 6.22 mm, PL = 4.08 mm, TL/GW = 2.32, PL/PW = 0.65. EW = 0.25 mm; OW = 0.25 mm. Among paratypes range of TL 15.9–22.7 mm; range of GW 6.9–11.0 mm; range of PW 4.83–7.75 mm; range of PL 3.43–4.61 mm.

**Figure 111.**
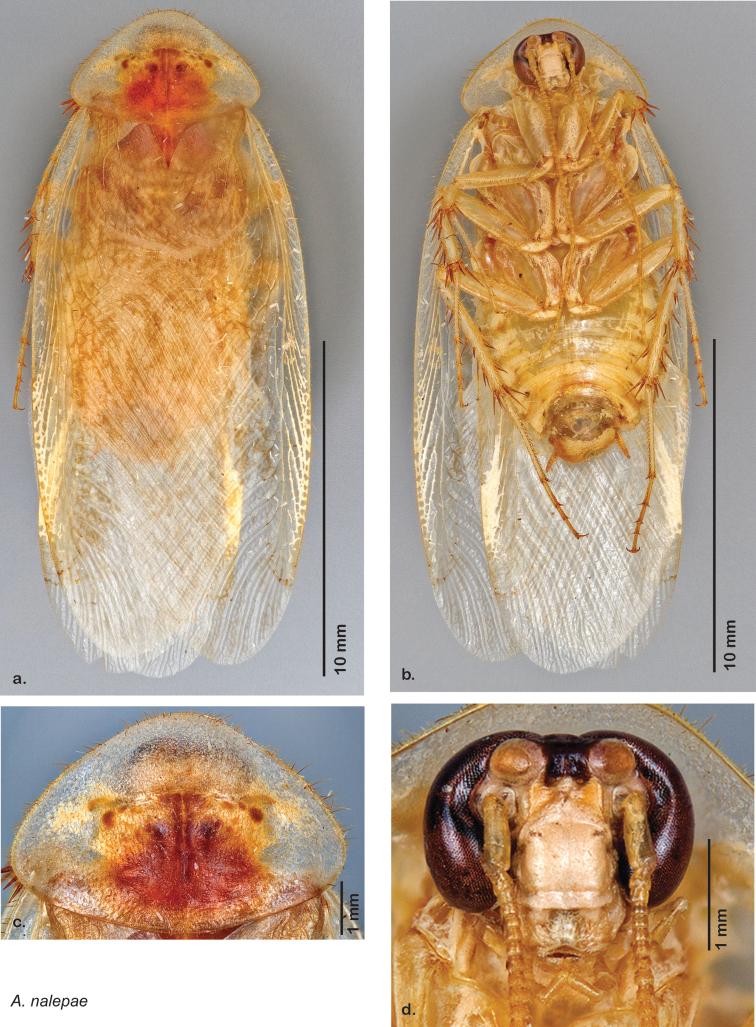
*Arenivaga nalepae* **a** dorsal habitus **b** ventral habitus **c** pronotum **d** head.

*Head*. Two ocelli very large, ovoid and protruding (0.5 × 0.4 mm), surrounded by waxy beige >0.1 mm border; vertex dark brown with small ridges in rays around upper apices of eyes and extending onto ocellar tubercles; interocellar space concave, dark brown with central medium brown dimple and two deep set medium brown dimples medial to inner apex of ocelli. Posterior frons pale orange-brown fading to waxy white towards clypeus, concave; anterior frons waxy white, bulbous; broad flat waxy white anteclypeus. See Fig. 111d.

*Pronotum*. Pronotum translucent, waxy beige; dorsal surface of pronotum with short fine brown setae laterally and anteriorly; pronotal pattern orange-brown “panther face” with little discernible detail; slight lateral aura. See Fig. 111c.

*Body*. Wing brace present. Two tarsal claws present. Legs and body light orange-brown, darker maculation laterally on each sternite; subgenital plate with darker orange-brown border; strongly asymmetrical with rounded apices. See Fig. 111b.

*Forewings*. Wings extended well beyond abdominal apex (~40% of wing length); light beige with occasional orange-brown blotches depending on specimen; surface translucent with slight sheen. See Fig. 111a.

**Figure 112.**
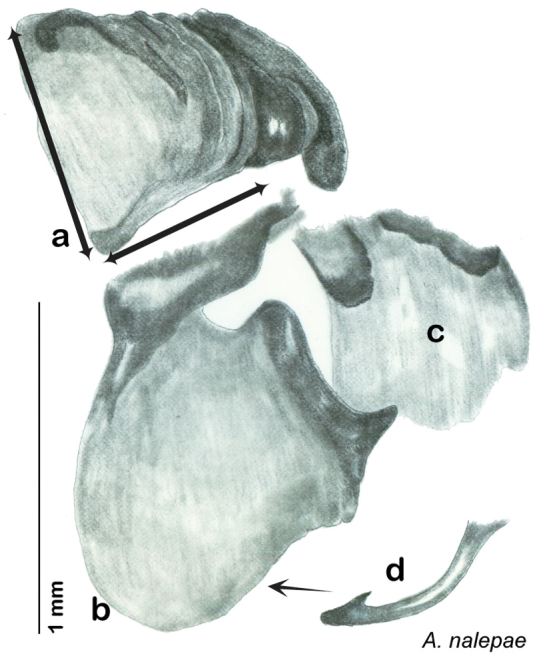
*Arenivaga nalepae*, genitalia: a) right dorsal phallomere **b** right ventral phallomere **c** small central sclerite **d** genital hook. Arrow(s) indicate diagnostic characters (see text).

*Genitalia*. Right dorsal phallomere composed of bulbous lightly sclerotized hook-shaped lobe, articulated with right ventral phallomere on lateral side; central field slightly sclerotized; medial margin heavily sclerotized, extending into smooth spine near distal end. Small central sclerite flat and finely punctate with posteriorly projecting, shagreened crescent in which dorsal arm of crescent is more prominently raised and toothed than ventral arm; right ventral phallomere extends from articulation to form rounded punctate structure at posterior apex but with shagreened corrugations at anterior apical end, followed by smaller offset shagreened projection and then by rounded concave arm extending beyond depth of rest of phallomere. Folded anterior portion of left phallomere setose, otherwise unmodified. Genital hook with long extension to pointed head with slight concavity on short hook; arm has distinct bend. See Fig. 112.

**Figure 113.**
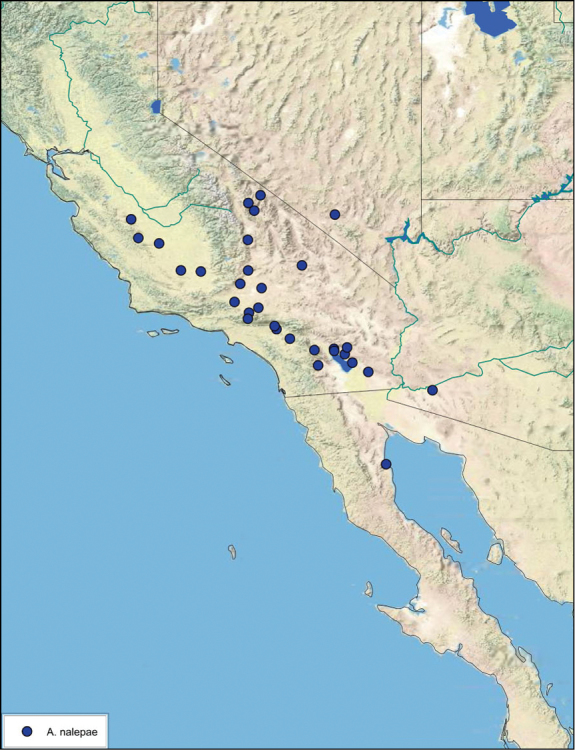
*Arenivaga nalepae*, distribution.

##### Habitat and natural history.

This species is found in varied habitat from seashore to mountains to inland sand dunes and lakeshores. All other life history elements remain unobserved.

#### Arenivaga nicklei sp. n.

http://zoobank.org/6D09FDC0-2F98-4FCA-A510-02682881B662

http://species-id.net/wiki/Arenivaga_nicklei

Figures 114 – 116

##### Type locality.

MEXICO, BC, 14.4 mi S Campo Alfonsina.

##### Material examined.

Holotype: ♂ in SDMC labeled “MEX: Baja Cal. Nor., 14.4 mi. S. Campo Alfonsina, X-20/26-87, Norris Bloomfield, green dot” “HOLOTYPE *Arenivaga nicklei* Hopkins, 2012” [red label with black border].

Paratypes (16): MEXICO: BC, Isla San Lorenzo Sur, canyon at SW end, 7/27/1986, Weissman & Lightfoot, Stop #86-86 (7, CAS); BCS, 10 mi S of Punta Prieta, 6/21/1938, Michelbacher & Ross, photo.spec. (1, CAS); BC, Isla de Cedros, trail from El Pueblo to Cerro de Cedros, 9/28/1984, 0-180 m, Weissman & Lee, Stop #84-64 (1, CAS); BC, 19 mi SW of Campo Alfonsina (canyon), 10/27-28/1987, N Bloomfield, green dot (2, SDMC); BC, 14.4 mi S of Campo Alfonsina, 10/20-26/1987, N Bloomfield, green dot (4, SDMC). USA: AZ, Pima Co., Organ Pipe Cactus NM, 3/23/1953, & H Dietrich (1, CUIC). All paratypes labeled “Paratype *Arenivaga nicklei* Hopkins 2012” [blue label with black border].

##### Etymology.

The name is a noun in the genitive case. This species is named for Dr. David Nickle, who with Dr. Ashley Gurney was the last to work on revising *Arenivaga* and was most generous with his knowledge upon learning of my work.

##### Distribution.

This species in found in central Baja and the nearby islands. See Fig. 116.

##### Diagnosis.

*Arenivaga nicklei* can be distinguished by its very prominent lobe on the small central sclerite and two prominent spines on the medial margin of the right dorsal phallomere. See Fig. 115.

##### Description.

**Male.** *Measurements*. Holotype TL = 18.4 mm, GW = 9.5 mm, PW = 5.20 mm, PL = 3.94 mm, TL/GW = 1.94, PL/PW = 0.76. EW = 0.20 mm; OW = 0.35 mm. Among paratypes range of TL 16.1–20.0 mm; range of GW 6.75–9.5 mm; range of PW 5.00–6.05 mm; range of PL 3.94–4.23 mm.

**Figure 114.**
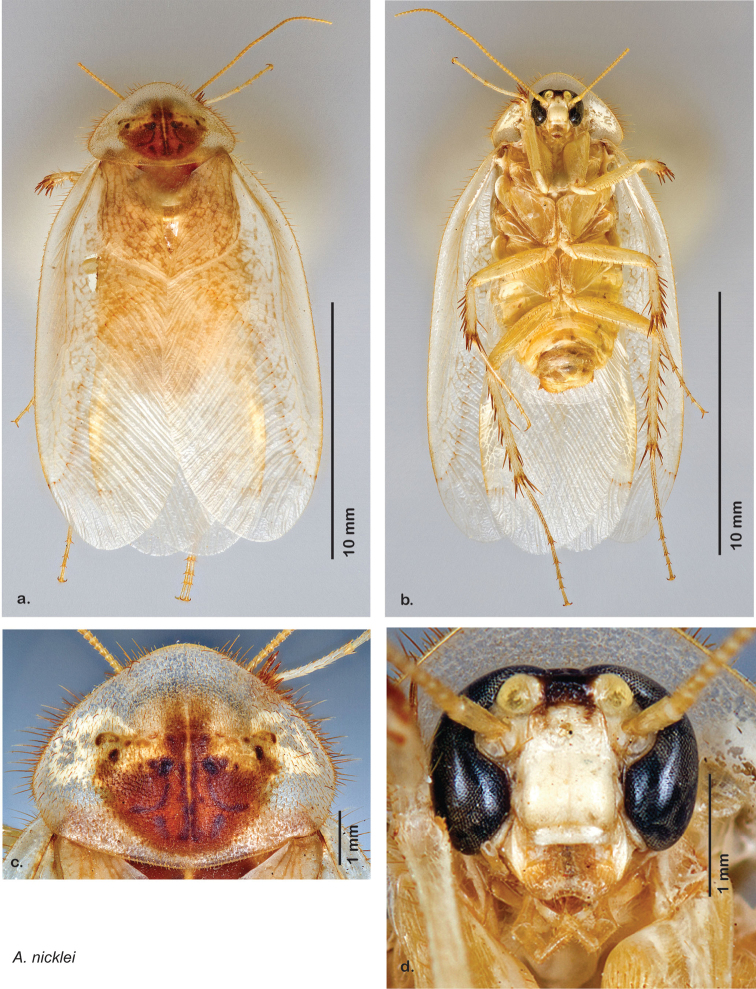
*Arenivaga nicklei* **a** dorsal habitus **b** ventral habitus **c** pronotum **d** head.

*Head*. Two ocelli large, ovoid and protruding (0.40 × 0.30 mm); vertex medium brown with small ridges in rays around upper apices of eyes and extending onto ocellar tubercles; interocellar space concave, medium brown, with small round central indentation. Posterior frons waxy white, unusually wide, concave; anterior portion of frons bulbous and waxy white; waxy white smooth anteclypeus. See Fig. 114d.

*Pronotum*. Pronotum translucent waxy beige; dorsal surface of pronotum with short orange-brown setae that are thicker and longer laterally; pronotal pattern orange-brown to dark brown “panther face”, with considerable detail no aura. See Fig. 114c.

*Body*. Wing brace present. Two tarsal claws present. Legs and body light brown; many specimens with yellow-brown maculations laterally on each sternite; subgenital plate light brown with darker margin; asymmetrical with rounded apices. See Fig. 114b.

*Forewings*. Wings extended well beyond abdominal apex (up to 30% of wing length); blotchy light to medium brown depending on specimen; surface matte and opaque. See Fig. 114a.

**Figure 115.**
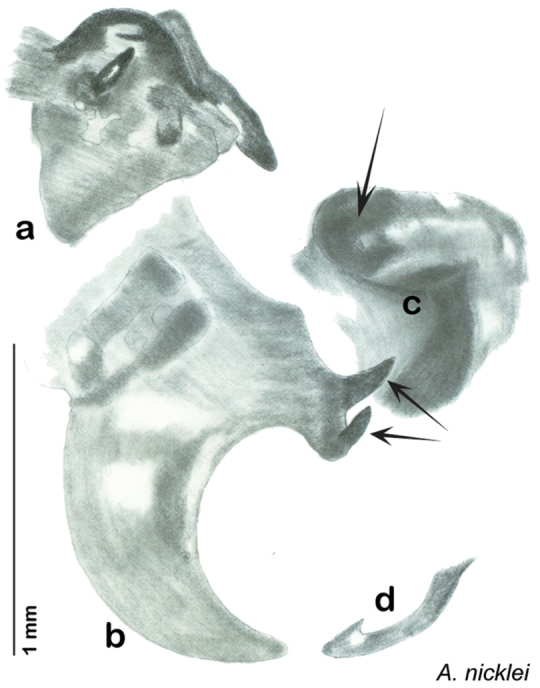
*Arenivaga nicklei*, genitalia: a) right dorsal phallomere **b** right ventral phallomere **c** small central sclerite **d** genital hook. Arrow(s) indicate diagnostic characters (see text).

*Genitalia*. Right dorsal phallomere composed of lightly sclerotized, unusually long, bulbous hook-shaped lobe, articulated with right ventral phallomere on lateral side; medial side of lobe deeply emarginated from medial edge of remainder of phallomere; central field shallow, cupped, lightly sclerotized; medial margin more heavily sclerotized, smooth, with long posterior projecting spine and second medially projecting spine located midway along medial margin. Small central sclerite concave, punctate, with large shagreened medially projecting bulge on ventral edge, second smaller punctate bulge above first; right ventral phallomere extends from articulation to form shagreened rounded structure, with prominent medially projecting spine located posteriorly; attached anteriorly is mildly dorsally projecting flanged punctate concave arm, that extends beyond depth of rest of phallomere. Folded anterior portion of left phallomere tri-fold, with small bulge on posterior fold, setose. Genital hook with moderate extension to pointed head with slight indentation along line to moderate hook; arm with distinct bend. See Fig. 115.

**Figure 116.**
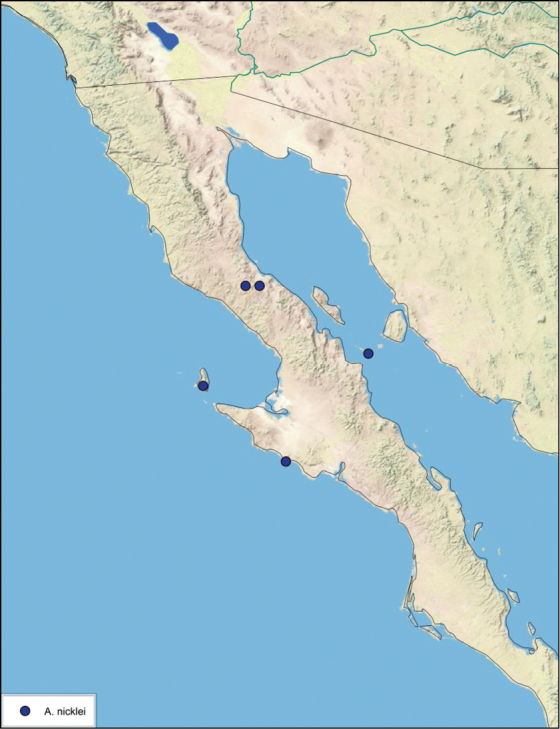
*Arenivaga nicklei*, distribution.

##### Habitat and natural history.

All life history elements remain unobserved.

#### Arenivaga nocturna sp. n.

http://zoobank.org/331ED73E-ED88-4D7B-8CA3-DF35AB3416B6

http://species-id.net/wiki/Arenivaga_nocturna

Figures 117 – 119

##### Type locality.

MEXICO, Baja California, Rancho Union.

##### Material examined.

Holotype: ♂ in SDMC labeled “Rancho Union, Baja California Mex., April 17, 1947, Charles F. Harbison Collector” “HOLOTYPE *Arenivaga nocturna* Hopkins, 2012” [red label with black border].

Paratypes (8): MEXICO: BC, Angeles Bay, Gulf of CA, 6/26/1921, EP Van Duzee, *Arenivaga erratica* Rehn det. Hebard 1922 on one specimen (2, CAS); BC, Angeles Bay, Gulf of CA, 6/26/1921, EP Van Duzee, Hebard Collection (2, ANSP); BC, Rancho Union, 4/17/1947, CF Harbison (1, SDMC); BC, Bahia de LA, 6/2/1981, Werner,Olson,Hetz,Thomas,Burne,Frank & MacLachlan (2, UAIC); Coahuila, ca. 7.2 mi. SSW of Cuatro Cienegas, 10/10/1978, C.E. Dunnr (1, ANSP). All paratypes labeled “Paratype *Arenivaga nocturna* Hopkins 2012” [blue label with black border].

##### Etymology.

The name is an adjective in the nominative singular. This species is named from the Latin meaning nocturnal or “of the evening”.

##### Distribution.

This species is found in southeastern Baja California Norte, Mexico. See Fig. 119.

##### Diagnosis.

*Arenivaga nocturna* can be distinguished by its narrow, sweeping hook-shaped lobe on the right dorsal phallomere, as well as the broad short spine on the medioventral side of the posterior end of the medial margin of the same phallomere. See Fig. 118.

##### Description.

**Male.** *Measurements*. Holotype TL = 20.7 mm, GW = 9.2 mm, PW = 6.53 mm, PL = 4.61 mm, TL/GW = 2.25, PL/PW = 0.71. EW = 0.30 mm; OW = 0.50 mm. Among paratypes range of TL 17.1–21.7 mm; range of GW 8.3–9.8 mm; range of PW 5.70–6.72 mm; range of PL 4.05–4.63 mm.

**Figure 117.**
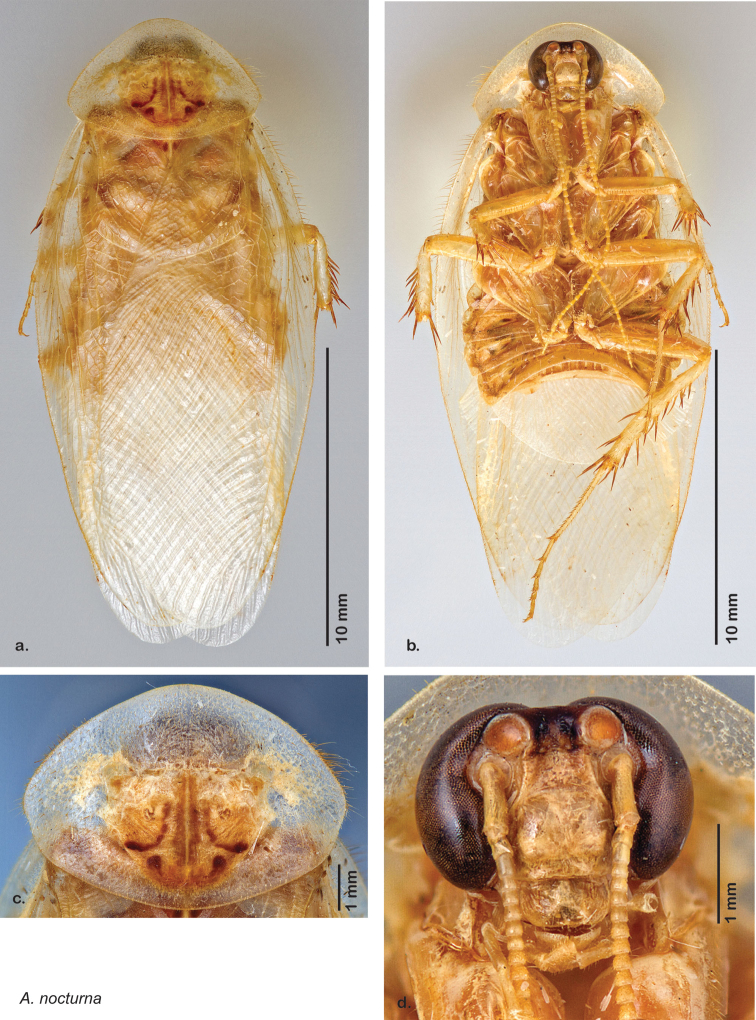
*Arenivaga nocturna* **a** dorsal habitus **b** ventral habitus **c** pronotum **d** head.

*Head*. Two ocelli large, ovoid and protruding (0.50 × 0.35 mm); vertex medium brown, with small ridges between apices of eyes and extending onto ocellar tubercles; interocellar space concave, medium brown, with two pale round indentations, pale medially. Posterior frons light brown, concave; anterior frons light brown, bulbous; light brown anteclypeus. See Fig. 117d.

*Pronotum*. Pronotum translucent waxy beige; dorsal surface of pronotum covered with short orange-brown setae that are longer and thicker laterally; pronotal pattern light orange-brown “panther face”; little discernible detail; no aura. See Fig. 117c.

*Body*. Wing brace present. Two tarsal claws present. Legs and body medium orange-brown; subgenital plate light orange-brown, strongly asymmetrical with rounded apices. See Fig. 117b.

*Forewings*. Wings extended beyond abdominal apex (up to 40% of total wing length); pale golden beige with no markings to light brown with scattered medium brown blotches; surface translucent and hyaline in most specimens, though matte and opaque in some. See Fig. 117a.

**Figure 118.**
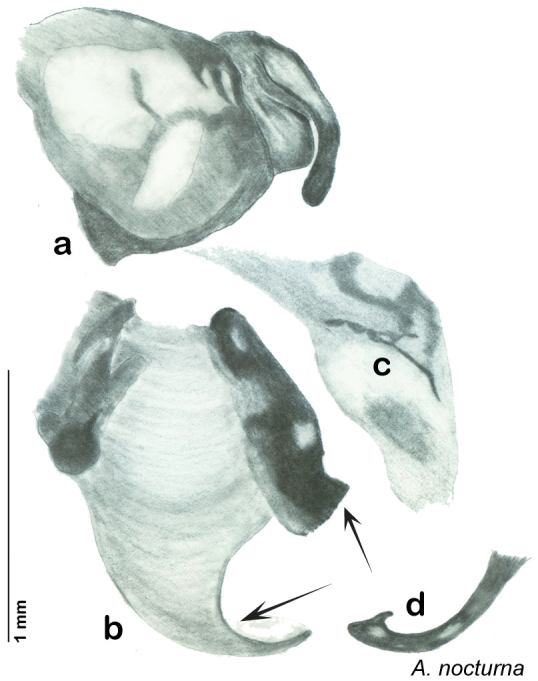
*Arenivaga nocturna*, genitalia: a) right dorsal phallomere **b** right ventral phallomere **c** small central sclerite **d** genital hook. Arrow(s) indicate diagnostic characters (see text).

*Genitalia*. Right dorsal phallomere composed of bulbous lightly sclerotized hook-shaped lobe, articulated with right ventral phallomere on lateral side; central field slightly sclerotized; medial margin heavily sclerotized, smooth anteriorly, becoming punctate then shagreened posteriorly, extending posteriorly into shagreened knob with short dorsally projecting spine. Small central sclerite large for this sclerite, concave, punctate with toothed patch along ventral edge. Right ventral phallomere extends from articulation to form large smooth rounded, increasingly punctate and sclerotized anteriorly; one smaller punctate ridge anteriorly, followed by moderate gap and then by long rounded concave shagreened arm extending beyond depth of rest of phallomere. Folded anterior portion of left phallomere setose, otherwise unmodified. Genital hook with moderate extension to pointed head with slight concavity on short hook; arm long, narrow, barely curved. See Fig. 118.

**Figure 119.**
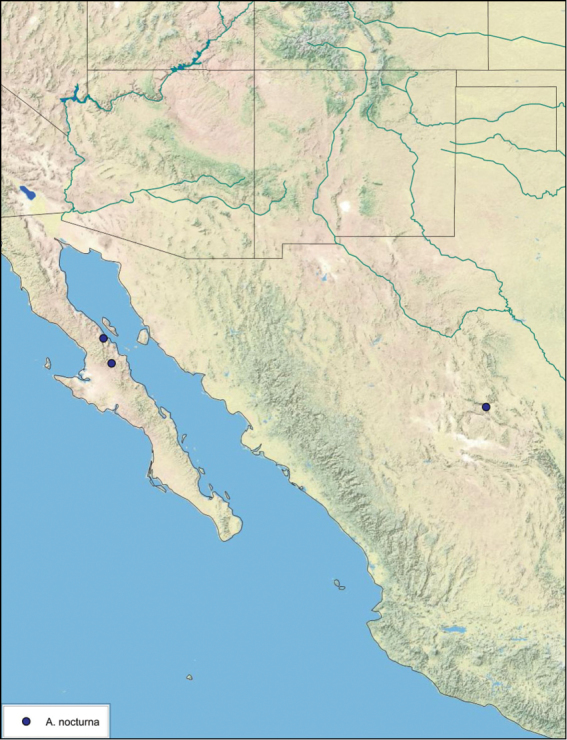
*Arenivaga nocturna*, distribution.

##### Habitat and natural history.

All life history elements remain unobserved.

#### Arenivaga pagana sp. n.

http://zoobank.org/65A4DC70-14C8-422B-B4C7-2D0185D1D002

http://species-id.net/wiki/Arenivaga_pagana

Figures 120 – 122

##### Type locality.

USA, Arizona, Mohave Co., Colorado River, Grand Canyon.

##### Material examined.

Holotype: ♂ in NAUF labeled “Mohave Co. AZ, Colorado R. GC, blue label with ‘3’, rm202.5L, 9/4/03, Coll. R.J.Delph, Ex: Light, New High Water” “HOLOTYPE *Arenivaga pagana* Hopkins, 2012” [red label with black border].

Paratypes (9): USA: AZ, Mohave Co., Colorado River GC, 9/4/2003, RJ Delph, blue label with ‘3’, rm 202.5L, ex.light, new high water (2, NAUF); AZ, Mohave Co., Colorado River GC, 9/9/2001, J Rundall, blue label with ‘3’, rm 198.0R, ex.light, old high water (1, NAUF); AZ, Mohave Co., Colorado River GC, 9/9/2002, RJ Delph, blue label with ‘3’, rm 186.5L, ex.light, old high water, NAU 107 (1, NAUF); AZ, Coconino Co., 10 mi. N & 4 mi. W of Page, 3/?-9/?/1985, 6000 ft., D Giuliani, antifreeze pit trap (2, CSCA); AZ, Coconino Co., Colorado River GC, 9/1/2002, RJ Delph, blue label with ‘3’, rm37.3L, ex.light, old high water (1, NAUF); AZ, Coconino Co., Colorado River GC, 10/18/1982, 855 m, LE Stevens, blue label with ‘3’, M:53R, Nankowap, sand dunes at night, Polyphagidae, *Arenivaga* det. D.Lightfoot (1, NAUF); AZ, Mojave Co., Virgin River Gorge, 8/3/1976, A.Strong (1, UCRC). All paratypes labeled “Paratype *Arenivaga pagana* Hopkins 2012” [blue label with black border].

##### Etymology.

The name is an adjective in the nominative singular. This species is named from the Latin meaning “of the country”, or rustic.

##### Distribution.

This species is found north of the Grand Canyon from southwestern Utah through northwestern Arizona to southeastern Nevada. See Fig. 122.

##### Diagnosis.

*Arenivaga pagana* can be distinguished by the unusual 90 degree bend in the medial margin of the right dorsal phallomere. See Fig. 121.

##### Description.

**Male.** *Measurements*. Holotype TL = 16.5 mm, GW = 9.1 mm, PW = 5.00 mm, PL = 3.46 mm, TL/GW = 1.81, PL/PW = 0.69. EW = 0.20 mm; OW = 0.30 mm. Among paratypes range of TL 16.2–16.6 mm; range of GW 6.9–9.1 mm; range of PW 4.50–5.00 mm; range of PL 3.43–3.89 mm.

**Figure 120.**
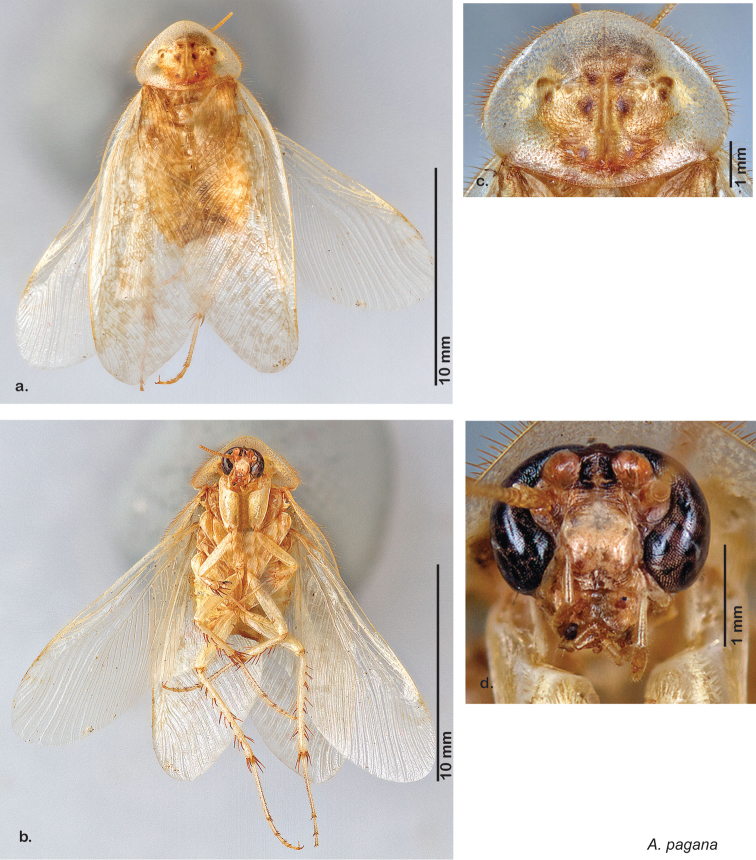
*Arenivaga pagana* **a** dorsal habitus **b** ventral habitus **c** pronotum **d** head.

*Head*. Two ocelli very large, ovoid and protruding (0.4 × 0.30 mm); vertex dark brown with small ridges in rays around upper apices of eyes and extending onto ocellar tubercles; interocellar space concave, smooth, medium brown, lighter brown medially, with two white horizontal indentations at base of interocular space. Posterior frons light transparent brown posteriorly fading to waxy beige anteriorly, slightly concave, with horizontal corrugations; anterior frons bulbous and waxy beige; light transparent brown smooth anteclypeus. See Fig. 120d.

*Pronotum*. Pronotum translucent, waxy beige; dorsal surface of pronotum with short orange-brown setae that are slightly thicker laterally; pronotal pattern varying from yellow to orange-brown to dark orange-brown “panther face”, with little discernible detail; no aura. See Fig. 120c.

*Body*. Wing brace present. Two tarsal claws present. Legs and body light brown with darker brown maculations laterally on each sternite; subgenital plate light brown with darker margin; asymmetrical with rounded apices. See Fig. 120b.

*Forewings*. Wings extended well beyond abdominal apex (~40% of wing length); translucent light beige with light brown blotches; surface varies from hyaline to very slight sheen depending on specimen. See Fig. 120a.

**Figure 121.**
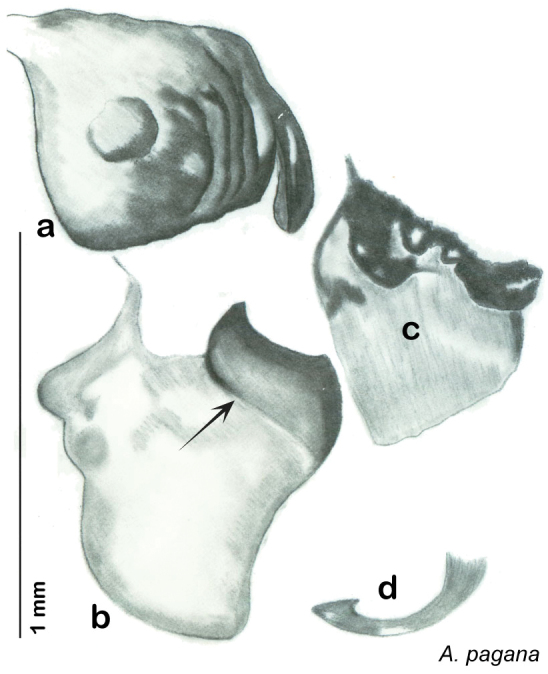
*Arenivaga pagana*, genitalia: a) right dorsal phallomere **b** right ventral phallomere **c** small central sclerite **d** genital hook. Arrow(s) indicate diagnostic characters (see text).

*Genitalia*. Right dorsal phallomere composed of bulbous lightly sclerotized hook-shaped lobe, articulated with right ventral phallomere on lateral side; central field lightly sclerotized; medial margin more heavily sclerotized, shagreened with rough edge, edge extends into slight point one third back from posterior end; anterior end of rim forms posteriorly curving point; anterior end of margin thickened for s short distance interiorly. Small central sclerite nearly flat, nondescript in shape, finely punctate with posteriorly projecting shagreened flanges at anterior end; right ventral phallomere extends from articulation to form smooth rounded structure becoming punctate and rugose anteriorly; attached anteriorly is mildly dorsally projecting flanged concave punctate arm that extends beyond depth of rest of phallomere. Folded anterior portion of left phallomere finely setose, otherwise unmodified. Genital hook with moderate extension to pointed head with short hook; arm with distinct bend. See Fig. 121.

**Figure 122.**
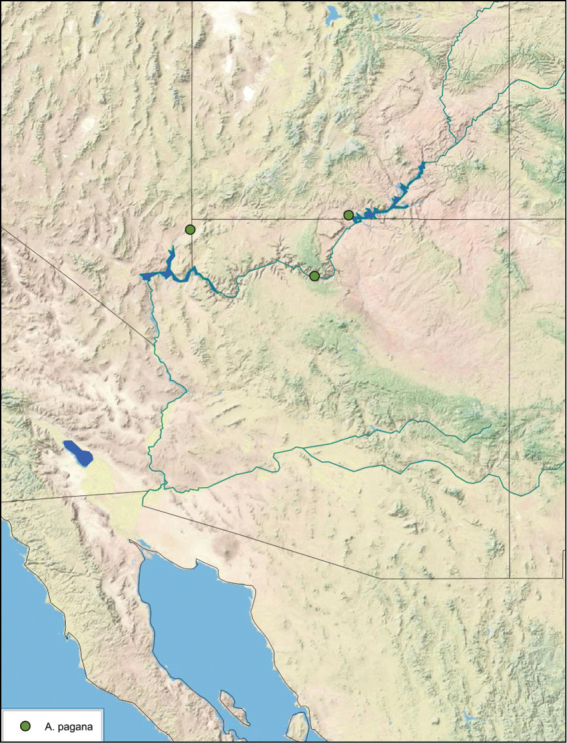
*Arenivaga pagana*, distribution.

##### Habitat and natural history.

All life history elements remain unobserved.

#### Arenivaga paradoxa sp. n.

http://zoobank.org/B5B9ABB1-4D6A-4A66-974A-3CC4755E6821

http://species-id.net/wiki/Arenivaga_paradoxa

Figures 123 – 125

##### Type locality.

MEXICO, BC, 15 mi S San Quintin.

##### Material examined.

Holotype: ♂ in SDMC labeled “MEXICO: Baja Ca. Norte, 15 mi S San Quintin (dunes), 12 July 1986, Bloomfield, green dot” “HOLOTYPE *Arenivaga paradoxa* Hopkins, 2012” [red label with black border].

Paratypes: None at this time.

##### Etymology.

The name is an adjective in the nominative singular. This species is from the Latin meaning strange or marvelous because of its strange modifications of all genital phallomeres.

##### Distribution.

This species is known only from the type locality in on the west coast of Baja California, Mexico. See Fig. 125.

##### Diagnosis.

*Arenivaga paradoxa* is very like *Arenivaga estelleae* but can be distinguished by the serrated and deeply sinuous medial margin on the right dorsal phallomere, as well as the large horseshoe-shaped gap on the right ventral phallomere. It shares with *Arenivaga estelleae* and *Arenivaga pumila* the odd scoop-shaped modification on the left phallomere. See Figs 124, 52 and 130.

##### Description.

**Male.** *Measurements*. Holotype TL = 18.1 mm, GW = 9.6 mm, PW = 5.00 mm, PL = 3.62 mm, TL/GW = 1.88, PL/PW = 0.72. EW = 0.45 mm; OW = 0.50 mm.

**Figure 123.**
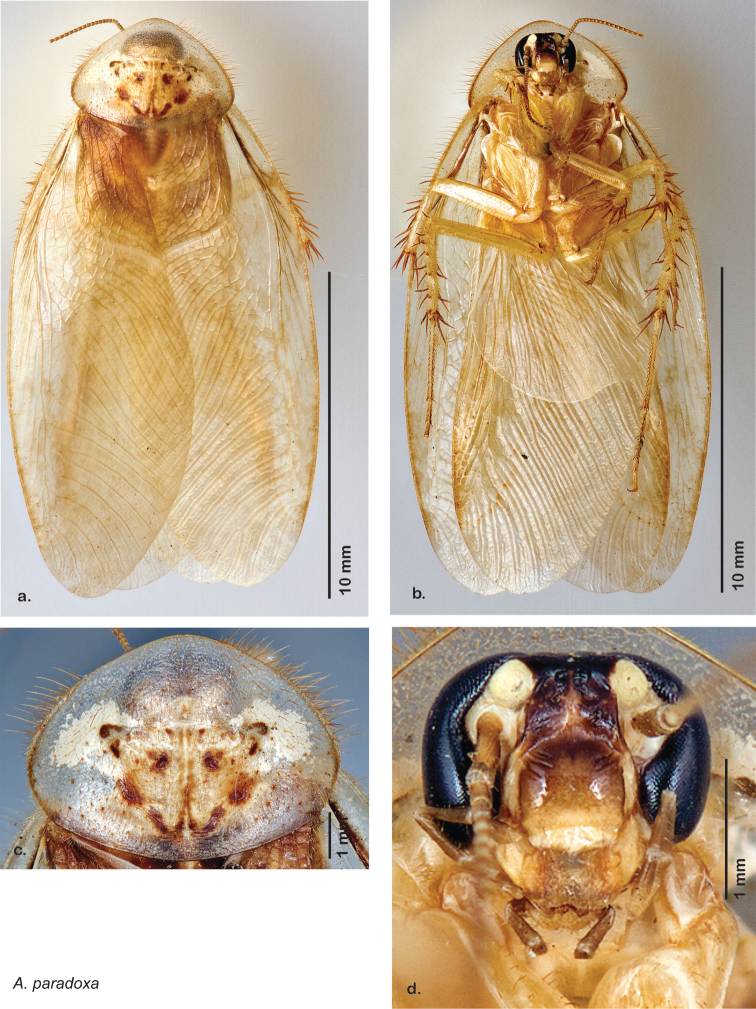
*Arenivaga paradoxa* **a** dorsal habitus **b** ventral habitus **c** pronotum **d** head.

*Head*. Two ocelli large, ovoid and protruding (0.30 × 0.25 mm); vertex dark brown with small ridges in rays around upper apices of eyes and extending onto ocellar tubercles; interocellar space very slightly concave, nearly flat, dark brown, lighter center line with two short horizontal linear indentations. Posterior frons medium brown; slightly concave; anterior frons bulbous, medium brown fading to light brown anteriorly, pointed posteriorly; waxy white smooth anteclypeus. See Fig. 123d.

*Pronotum*. Pronotum translucent waxy beige, with fine medium brown border; dorsal surface of pronotum with short orange-brown setae that are thicker and longer laterally; pronotal pattern light and medium brown “panther face”, with moderate detail discernible; scattered small brown maculations on posterior half of pronotum; no aura. See Fig. 123c.

*Body*. Wing brace present. Two tarsal claws present. Legs and body light brown; subgenital plate light brown; asymmetrical with rounded apices. See Fig. 123b.

*Forewings*. Wings extended well beyond abdominal apex; very light brown with light brown blotches; surface translucent with very slight sheen. See Fig. 123a.

**Figure 124.**
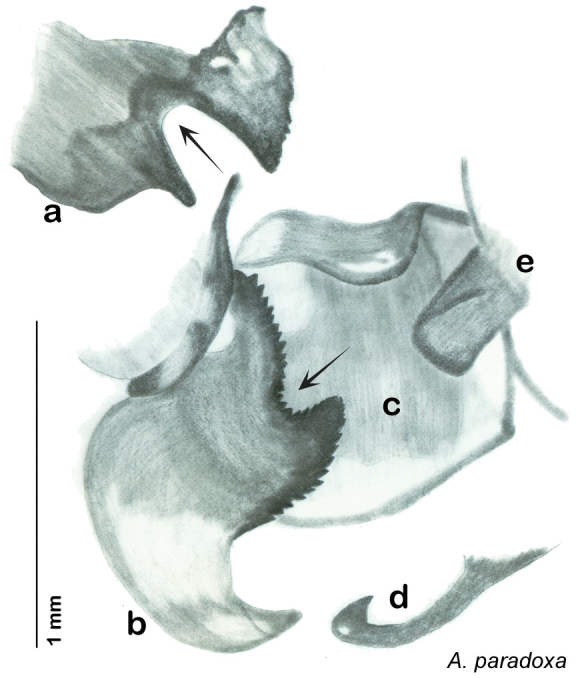
*Arenivaga paradoxa*, genitalia: a) right dorsal phallomere **b** right ventral phallomere **c** small central sclerite **d** genital hook **e** left phallomere. Arrow(s) indicate diagnostic characters (see text).

*Genitalia*. Right dorsal phallomere composed of lightly sclerotized, bulbous hook-shaped lobe, articulated with right ventral phallomere on lateral side; medial side of lobe becoming more sclerotized and shagreened as it recedes anteriorly; central field lightly sclerotized; medial margin comprised of two toothed waves. Small central sclerite delicate, concave, finely punctate, with thin sweeping margin that swings forward and attaches to dorsal side of bulbous hook-shaped lobe; anterior edge slightly or punctate and folded back posteriorly; right ventral phallomere extends from and unusually wide articulation to form punctate rounded structure, becoming shagreened on anterior side, with prominent medially projecting flat rough-edged spine located anteriorly; attached anteriorly after wide gap is broad flanged punctate concave arm that extends to depth of adjacent spine, edge toothed. Folded anterior portion of left phallomere dramatically modified into heavily setose, medially projecting, scoop shape. Genital hook with moderate extension to pointed head with broad hook, arm with shallow curve. See Fig. 124.

**Figure 125.**
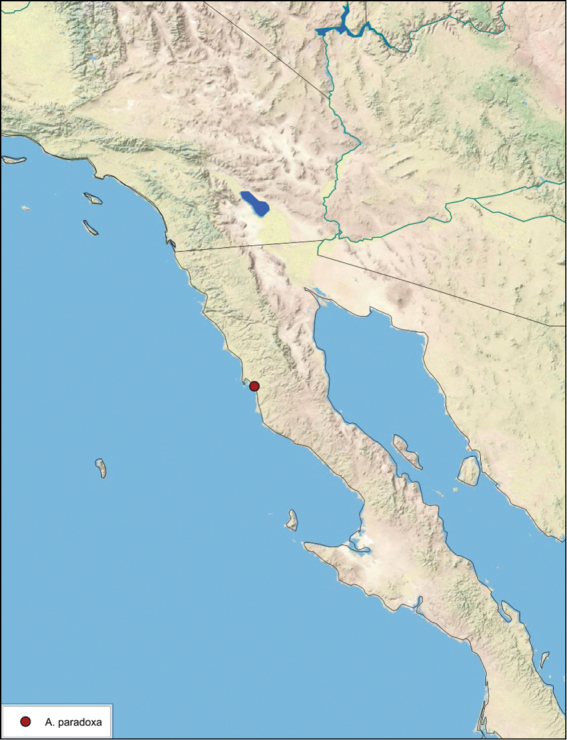
*Arenivaga paradoxa*, distribution.

##### Habitat and natural history.

All life history elements remain unobserved.

#### Arenivaga pratchetti sp. n.

http://zoobank.org/A2799EE6-7FEC-485A-85D7-109D22F90A07

http://species-id.net/wiki/Arenivaga_pratchetti

Figures 126 – 128

##### Type locality.

USA, California, Riverside Co., Rice Dunes.

##### Material examined.

Holotype: ♂ in EMEC labeled “ARIZ: Mohave Co. [now La Paz Co.], 3 mi. SE of Parker, VI-28-78, J.Powell, black light trap” “HOLOTYPE *Arenivaga pratchetti* Hopkins, 2012” [red label with black border].

Paratypes (15): USA: CA, Riverside Co., Rice Dunes, 19 Sept 1977, A.R. Hardy & FG Andrews, cereal bowl pit trap (1, CSCA); CA, LA Co., Black Butte, Antelope Valley, 8/22/1959, G Sphon (1, LACM); CA, LA Co., Black Butte, Antelope Valley, 10/5/1959, Honey & Sphon (1, LACM); CA, San Bernardino Co., Kelso Dunes, 9 air mi SW of Kelso, 6/29/1978, J Powell, black light (2,EMEC); CA, San Bernardino Co., Kelso Dunes, 9 air mi S of Kelso, 6/29-30/1978, Doyen & Rude, Pitfall trap (3, EMEC); AZ, Mohave Co. (now La Paz Co.), 3 mi. SE of Parker, 6/28/1978, J.Powell, black light trap (7, EMEC). All paratypes labeled “Paratype *Arenivaga pratchetti* Hopkins 2012” [blue label with black border].

##### Etymology.

The name is a noun in the genitive case. This species is named for the one and only Terry Pratchett, creator of Disc World and many happy hours of reading. May the strength and durability of these creatures I so love impart those gifts to him in full measure in his fight against Alzheimer’s.

##### Distribution.

This is species is found in the southern Mohave Desert of California and far western Arizona. See Fig. 128.

##### Diagnosis.

The external phenotype of *Arenivaga pratchetti* may be confused with that of *Arenivaga investigata* with whom it is sympatric but *Arenivaga pratchetti* has dramatically pointed apices on its subgenital plate which distinguish it. See Fig. 9.

##### Description.

**Male.** *Measurements*. Holotype TL = 16.7 mm, GW = 7.6 mm, PW = 5.35 mm, PL = 3.97 mm, TL/GW = 2.12, PL/PW = 0.74. EW = 0.25 mm; OW = 0.20 mm. Among paratypes range of TL 16.4–18.6 mm; range of GW 7.6–9.0 mm; range of PW 5.32–6.09 mm; range of PL 3.93–4.48 mm.

**Figure 126.**
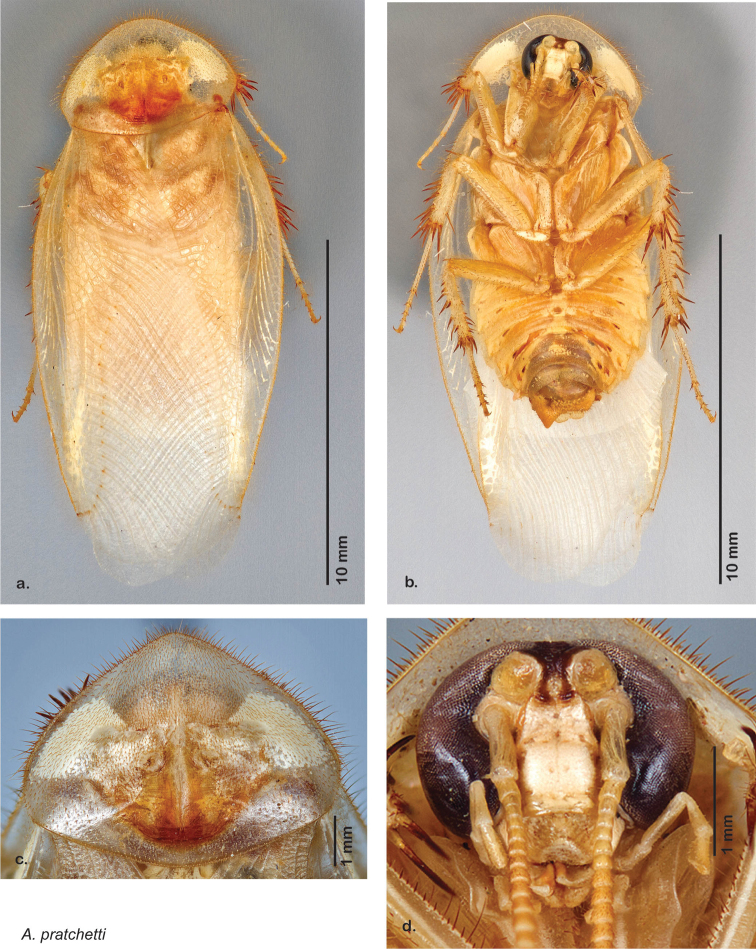
*Arenivaga pratchetti* **a** dorsal habitus **b** ventral habitus **c** pronotum **d** head.

*Head*. Two ocelli very large, ovoid and protruding (0.5 × 0.35 mm); vertex medium brown with small ridges in rays around upper apices of eyes and extending onto ocellar tubercles; interocellar space deeply concave, smooth, medium brown with light brown arrowhead shape medially. Frons and clypeus waxy white; posterior frons with horizontal corrugations, slightly concave; anterior portion of frons bulbous; waxy white smooth anteclypeus. See Fig. 126d.

*Pronotum*. Pronotum translucent, waxy beige; dorsal surface of pronotum with short orange-brown setae that are slightly thicker laterally; pronotal pattern “koala face” varying from yellow to orange-brown to dark orange-brown depending on specimen but in all instances with very little detail discernible; no aura. See Fig. 126c.

*Body*. Wing brace present. Two tarsal claws present. Legs and body light brown with pale yellow striping brown maculations laterally on each sternite; subgenital plate light brown; strongly asymmetrical with pointed apices. See Fig. 126b.

*Forewings*. Wings extended well beyond abdominal apex (~30% of wing length); light golden beige; surface translucent with very slight sheen. See Fig. 126a.

**Figure 127.**
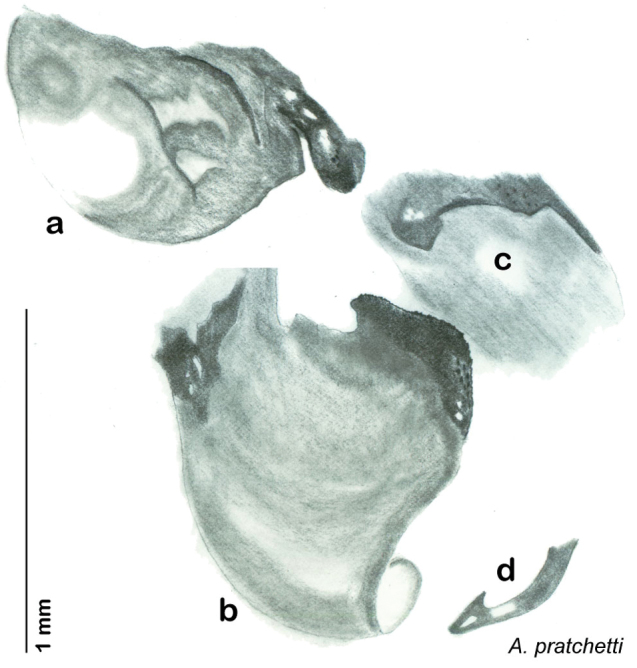
*Arenivaga pratchetti*, genitalia: a) right dorsal phallomere **b** right ventral phallomere **c** small central sclerite **d** genital hook.

*Genitalia*. Right dorsal phallomere composed of bulbous lightly sclerotized hook-shaped lobe, articulated with right ventral phallomere on lateral side; central field lightly sclerotized; medial margin more heavily sclerotized, shagreened with toothed edge and slight thickening centrally creating small bulge along rim. Small central sclerite nearly flat, nondescript in shape, finely punctate with posteriorly projecting shagreened curved flange at anterior end; right ventral phallomere extends from articulation to form smooth rounded structure becoming punctate and rugose anteriorly; attached anteriorly is mildly dorsally projecting flanged concave punctate arm that extends greater than depth of phallomere. Folded anterior portion of left phallomere finely setose, otherwise unmodified. Genital hook with moderate extension to pointed head with short hook; arm with distinct bend. See Fig. 127.

**Figure 128.**
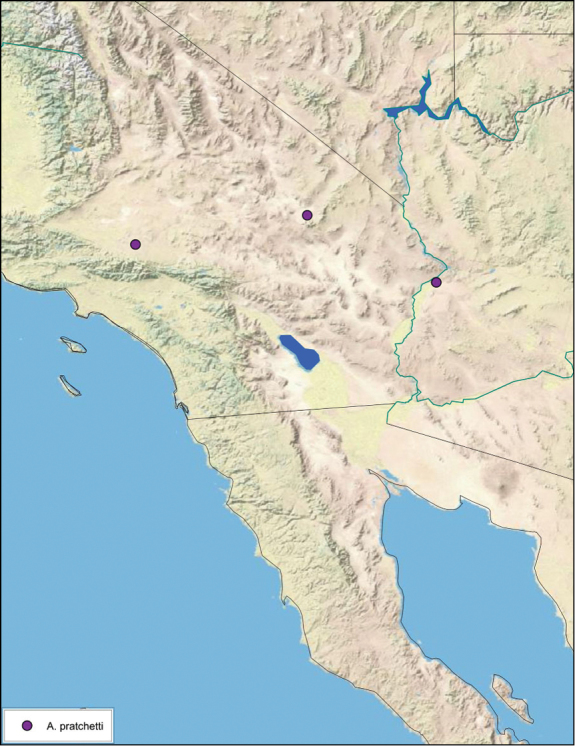
*Arenivaga pratchetti*, distribution.

##### Habitat and natural history.

All life history elements remain unobserved.

#### Arenivaga pumila sp. n.

http://zoobank.org/84440053-AC59-43E9-8F0B-51BEE1741D3E

http://species-id.net/wiki/Arenivaga_pumila

Figures 129 – 131

##### Type locality.

MEXICO, BCS, San Hilario.

##### Material examined.

Holotype: ♂ in CAS labeled “MEX., Baja Calif. Sur, San Hilario, El.1000’, XI-5-68, E.L.Sleeper & F.J. Moore, Collected at BLACKLITE” “HOLOTYPE *Arenivaga pumila* Hopkins, 2012” [red label with black border].

Paratypes (33): MEXICO: BCS, 25 mi S of Santa Rosalia, 7/25/1938, Michelbacher & Ross, genitalia missing on one specimen (5, CAS); BCS, 5.7 mi SE of Mulege, 7/7/1979, Andrews, Hardy & Giuliana, walking dunes at night (1, CSCA); BCS, 38.8 km S of Santa Rosalia, 5/30/1973, Sleeper 61965-6, polyphagidae, *Arenivaga* n. sp. A, det. FW Fisk 81 (3, CSLB); BCS, 25 mi S of Santa Rosalia, 7/25/1938, Michelbacher & Ross, photo.spec. (1, USNM); BCS, 15 mi N of El Refugio, 7/4/1938, Michelbacher & Ross (7, CAS); BCS, Magdalena Bay, 7/18/1938, Ross & Michelbacher (1, CAS); BCS, Arroyo San Gregorio, 13 air km WNW of La Purissima, 4/24-26/1983, Wasbauer & Slansky, taken at lights (1, CSCA); BC, 4 mi NW of Rancho San Juan, 4/3-4/1961, AG Smith, at lantern (1, CAS); BCS, 27 mi W of La Paz, 11/18/1968, 1000 ft., Sleeper & Moore#1, E1, blacklite (1, CAS); BCS, 1/20-21/1980, Sleeper 77884.7 (1, CSLB); BC, 1 mi S of Mulege, 8/27/1959, Radford & Werner, light trap (1, UAIC); BCS, 5 mi S of Mulege, 10/15-16/1990, S McElfresh (2, UCRC); BCS, San Hilario, 11/5/1968, 1000 ft., Sleeper & Moore, blacklite (3, CAS); BCS, 7.4 km W of Santa Rita on road to Puerto Chale, river crossing at El Medano, 12/30/1978, Weissman,Love,Lee & Mullinex, Stop 79-12, polyphagidae (2, CAS); BCS, 9 km SE of Santa Rita (km 148), 8/25/1977, 75 m, Fisher & Westcott (1, CAS); BCS, 19 mi SW of San Miguel Comondu, 11/14-15/1968, 800 ft., Sleeper & Moore (1, CAS); BCS, 3.3 km S El Cien, 9/26/1981, D Faulkner & F Andrews, at blacklight (1, CSCA). All paratypes labeled “Paratype *Arenivaga pumila* Hopkins 2012” [blue label with black border].

##### Etymology.

The name is an adjective in the nominative singular. This species is named from the Latin meaning small or dwarfish because of its small size.

##### Distribution.

This species is found throughout Baja California Sur, Mexico. See Fig. 131.

##### Diagnosis.

*Arenivaga pumila* is easily distinguished by its small size. It is the smallest in overall size of the *Arenivaga* identified to date, though *Arenivaga ricei* is shorter in total length than some specimens of *pumila*.

##### Description.

**Male.** *Measurements*. Holotype TL = 14.2 mm, GW = 7.0 mm, PW = 4.16 mm, PL = 3.13 mm, TL/GW = 2.03, PL/PW = 0.75. EW = 0.05 mm; OW = 0.20 mm. Among paratypes range of TL 10.6–14.6 mm; range of GW 4.9–7.0 mm; range of PW 4.02–4.27 mm; range of PL 2.80–3.14 mm.

**Figure 129.**
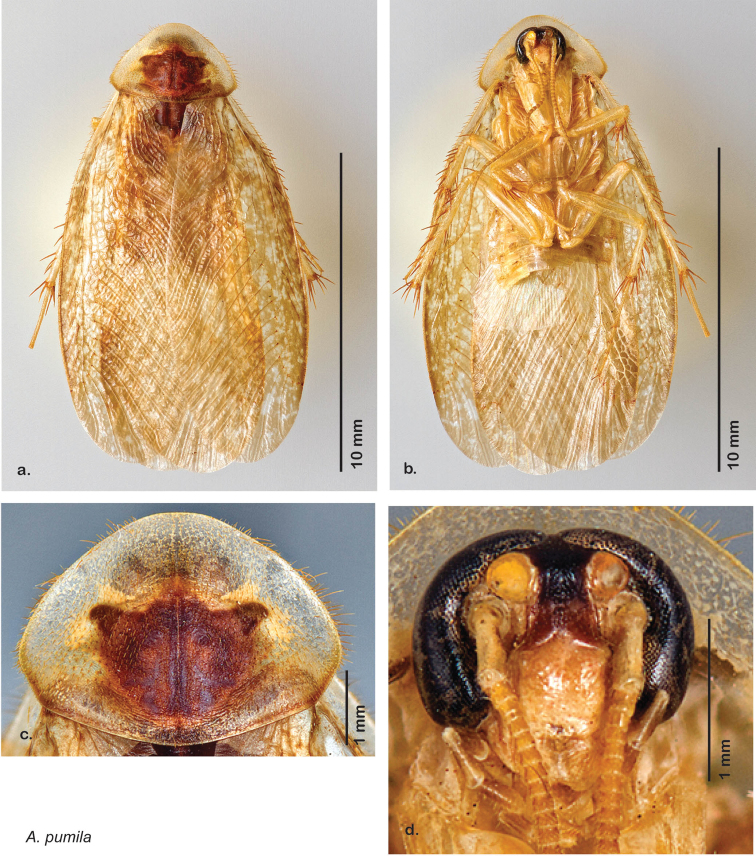
*Arenivaga pumila* **a** dorsal habitus **b** ventral habitus **c** pronotum **d** head.

*Head*. Two ocelli large, ovoid and protruding (0.30 × 0.25 mm); vertex dark brown; interocellar space concave, dark brown. Posterior frons medium brown, concave; anterior frons light brown, bulbous, posterior margin with medial point; light brown anteclypeus. See Fig. 129d.

*Pronotum*. Pronotum translucent waxy beige; dorsal surface of pronotum with short orange-brown setae; pronotal pattern light to medium orange-brown or medium brown “panther face”; no discernible detail; no aura. See Fig. 129c.

*Body*. Wing brace absent. Two tarsal claws present. Legs and body light orange-brown; subgenital plate light orange-brown; asymmetrical with long apex pointed, short apex rounded. See Fig. 129b.

*Forewings*. Wings extended beyond abdominal apex (up to 40% of total wing length); blotchy medium orange-brown to medium brown; surface translucent, matte or with slight sheen. See Fig. 129a.

**Figure 130.**
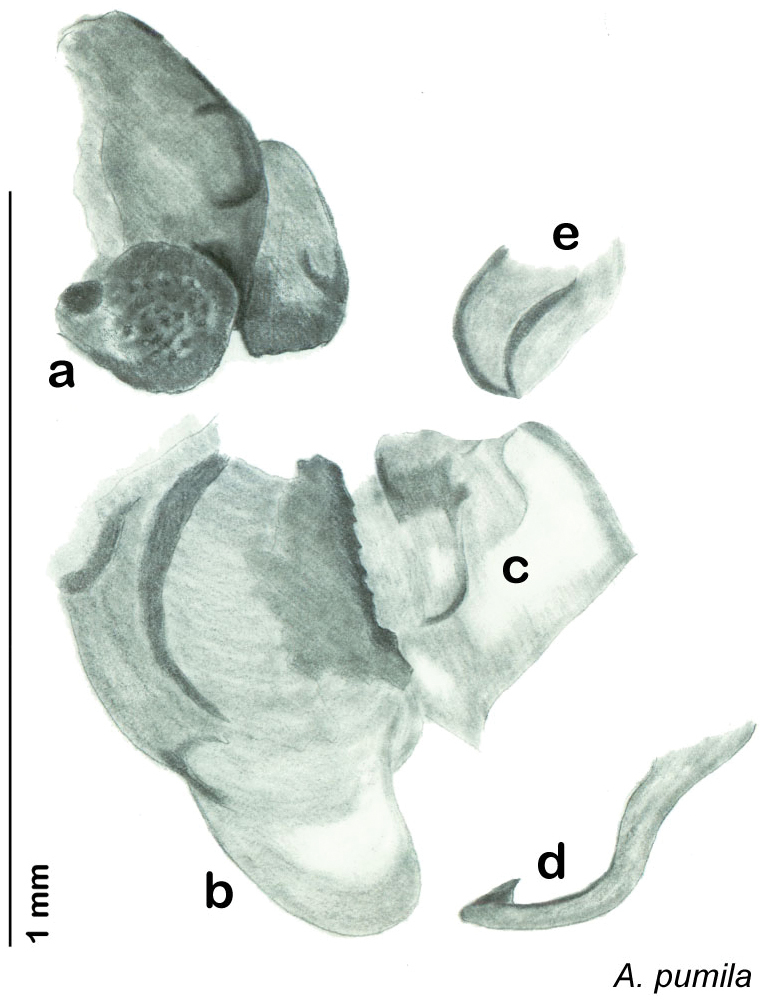
*Arenivaga pumila*, genitalia: a) right dorsal phallomere **b** right ventral phallomere **c** small central sclerite **d** genital hook **e** left phallomere. Arrow(s) indicate diagnostic characters (see text).

*Genitalia*. Right dorsal phallomere composed of lightly sclerotized, bulbous lobe, articulated with right ventral phallomere on lateral side; central field lightly sclerotized, deeply cupped; punctate as approaching medial edge which is toothed and has central concavity. Small central sclerite in two parts, lightly sclerotized, finely punctate; first part concave with slight anterior bulge, posterior end connecting with dorsal side of right dorsal phallomere; second part small lightly sclerotized square sitting adjacent to bulge of first part; attached to anterior end of left phallomere. Articulation between right phallomeres extends into right ventral phallomere consisting of punctate to shagreened medially projecting lobe with central indentation; anteriorly narrow gap followed by shagreened flange. Folded anterior portion of left phallomere dramatically modified into setose, medially projecting, scoop shape. Genital hook with moderate extension to pointed head and moderate hook; arm with distinct bend. See Fig. 130.

**Figure 131.**
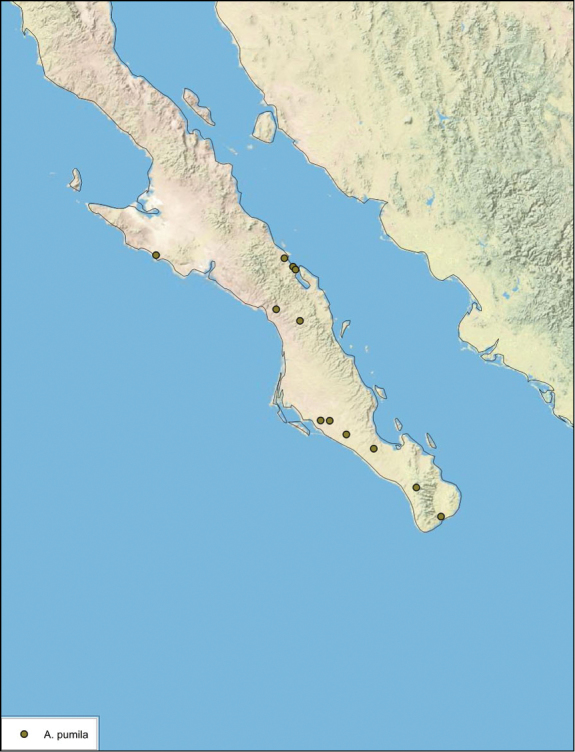
*Arenivaga pumila*, distribution.

##### Habitat and natural history.

All life history elements remain unobserved.

#### Arenivaga rehni

Hebard

http://species-id.net/wiki/Arenivaga_rehni

Figures 132 – 134

Arenivaga rehni Hebard 1917, Memoirs of the American Entomological Society, 2, pp. 1–284 + plates and index.
Arenivaga rehni Hebard 1920, Transactions of the American Entomological Society, 46(2), pp. 197–217.

##### Material examined

**(485).** BCS, San Miguel, 7/3/1938, Michelbacher & Ross (3, CAS); BCS, 6 mi. N of Triunfo, 7/15/1938, Michelbacher & Ross (2, CAS); BCS, Venancio, 7/17/1938, Michelbacher & Ross (7, CAS); BCS, 25 mi. S Santa Rosalia, 7/25/1938, Michelbacher & Ross (1, USNM); BCS, 20 mi. N of Comondu, 7/23/1938, Michelbacher & Ross (1, USNM); BCS, 3 mi. N of San Pedro, 7/6/1938, Michelbacher & Ross (4, CAS); BCS, 17 mi. S of Ensenada, 6/14/1938, Michelbacher & Ross (2, CAS); BCS, Triunfo, 7/13/1938, Michelbacher & Ross (2, CAS); BCS, Triunfo, 7/7/1938, Michelbacher & Ross (2, CAS); BCS, 15 mi. N of El Refugio, 7/4/1938, Michelbacher & Ross (8, CAS); BCS, San Domingo, 7/19/1938, Michelbacher & Ross (1, CAS); BCS, 15 mi. W of La Paz, 7/5/1938, Michelbacher & Ross (2, CAS); BCS, 5 mi. S of Miraflores, 7/10/1938, Michelbacher & Ross (1, CAS); BCS, 4 mi. S of Mission San Javier, 5/18/1969, 1000 ft., S.C.Williams, #201(2) (2, CAS); BCS, Playa los Cerritos, 3/24/1986, Faulkner & Bloomfield, UV light, green dot (1, CAS); BCS, 1/4 m S. Rancho Buena Vista, 25 ft., S.C.Williams, #190(2) (2, CAS); BCS, Cabo Pulmo, 9/10-11/1984, sea level, J.P & K.E. Donahue (3, CAS); BCS, Gulf of Baja California, Isla Danzante, 7/16/1984, Weissman & Lightfoot, S84-31 (1, CAS); BCS, 6 mi. SE of Santa Rita, 6/24/1967, Sleeper & Fisher, Black light (2, CAS); BCS, 9 km SE Santa Rita (km148), 8/25/1977, 75 m, Fisher & Westcott (1, CAS); BCS, 1 km S of San Lucas turnoff on Hwy.1 at km 176, 7/13/1978, Weissman & Lightfoot, Stop 47 (2, CAS); BCS, 9.9 mi. W of Ramal a los Naranjos, 3/23/1986, Faulkner & Bloomfield, green dot (2, CAS); BCS, S end of Isla San Francisco, 4/10/1974, JT Doyen (1, CAS); BCS, 6 km W of Hwy.1 at km 54, 9/26/2003, Bill Warner (2, WB Warner); BCS, 1 km W of Ranal de los Naranjos, 7/3/2002, 23.14.15N 109.57.22, Bill Warner, UV light (1, WB Warner); BCS, Ro. Palmarito, 10/30/1961 (1, USNM); BCS, S end of Isla San Francisco, 4/11-12/1974, JT Doyen (2, CAS); BCS, 12.8 mi. SSE Santa Rosalia, 9/23/1981, Faulkner & Andrews, green dot (4, SDMC); BCS, Santiago, NW side of town, 9/26/1979, Weissman,Lightfoot & Love, Stop 79-204 (1, CAS); BCS, 4 mi. S El Pescadero, 10/23-24/1968, Sleeper & Moore, Black light (1, CAS); BCS, 1.5 mi. E of San Jorge, 7/25/1971, Real & Main, UV light (1, CAS); BCS, area just N of Santa Inez, 4/26/1979, 518 m, DB Weissman, #79-97 (1, CAS); BCS, Puerto Escondido, 4/25/1979, DB Weissman, #79-97 (1, CAS); BCS, S end of Isla San Francisco, 4/11-12/1974, L.Cheng (1, CAS); BCS, El Sargento, 7/29/1971, Real & Main, UV light (10, CAS); BCS, 6 mi. E of San Jose del Cabo, 10/26-27/1968, 200 ft., Sleeper & Moore, Black light (2, CAS); BCS, San Jose del Cabo, garden area, 7/19/1978, Weissman & Lightfoot, #78-69 (1, CAS); BCS, 10 mi. SW of San Jose del Cabo, 9/1/1959, Radford & Werner, light trap (13, UAIC); BCS, 15 km. E of San Jose del Cabo, 11/8-9/1982, Sclinger,Irwin & Griswold (2, EMEC); BCS, #6 Puerto L.Mateos Rd.17.8 mi. W of Mex. 1, 8/29/1981, RE Love (1, CAS); BCS, 15 mi. W of Mulege, 8/24/1995, 290 m, Weissman & Lightfoot, Stop 95-79 (1, CAS); BCS, Mulege, 8/29/1995, Weissman & Lightfoot, Stop 95-92 (1, CAS); BCS, 5 mi. W of San Bartolo, 7/13/1938, Michelbacher & Ross (1, CAS); BCS, El Marmol, 9/24/1941, Ross & Bohart (1, CAS); BCS, 2 mi. NE of El Rosario, 12/7/1958, HB Leech (1, CAS); BCS, 9 mi. S of Todos Santos on Hwy. 17, 1/14/1959, HB Leech (2, CAS); BCS, San Pedro ~4 mi. S of Todos Santos, 1/13/1959, HB Leech (2, CAS); BCS, Chapala Dry Lake, 9/25/1941, Ross & Bohart (1, CAS); BCS, 7 mi. N of Santa Anita on Hwy. 19, 1/7/1959, HB Leech (2, CAS); BCS, 14.5 km E of Mex. Hwy.19 on road to La Burrera, 4/22/1979, 304 m, DB Weissman, #79-87 (4, CAS); BCS, 3 mi. E of La Burrera, 10/17-18/1968, 1800 ft., Sleeper & Moore, Black light (2, CAS); BCS, 1 mi. W of La Burrera, 10/17-18/1968, 1500 ft., Sleeper & Moore, Black light (1, CAS); BCS, Puerto Chileno, 11/26/1961, (1, USNM); BCS, San Jose del Cabo, 11/11/1952, CF Harbison (1, SDMC); BCS, 2.5 mi. SE of Todos Santos, 10/16-17/1968, 200 ft., Sleeper & Moore, Black light (2, CAS); BCS, Todos Santos, garden area, 7/22/1978, Weissman & Lightfoot, Stop 76 (1, CAS); BCS, La Burrera Wash, 9/27/1979, Weissman,Lightfoot & Love, Stop 79-209 (1, CAS); BCS, 3 mi. W of San Miduel de Comondu, 4/21/1969, 1500 ft., SC Williams, #181(1) (9, CAS); BCS, San Jose del Cabo, 12/30/1967, CF Harbison (2, SDMC); BCS, Isla San Jose,1 mi. S of Punta Colorado, 4/8-9/1974, JT Doyen (6, CAS); BCS, Gulf of California, Isla San Jose, 4/12/1962, ??.59N 110.38W, CF Harbison (1, SDMC); BCS, 1.5 mi.N of El Pilar, 11/6-7/1968, 1000 ft., Sleeper & Moore, Black light (1, CAS); BCS, Isla Cayo, 4/11/1974, Doyen,Cheng & Lewin (1, CAS); BCS, Isla del Carmen, canyon on W side of island near Punta Baja, 7/19/1984, Weissman & Lightfoot, S84-37 (5, CAS); BCS, Isla del Carmen, NW side, 7/18/1984, Weissman & Lightfoot, S84-34 (2, CAS); BCS, Isla del Carmen,1st major canyon S of Punta Cholla, 7/18/1984, Weissman & Lightfoot, S84-35 (2, CAS); BCS, dunes ~4.5 mi. S of Mulege, 9/13/1983, Evans,Smith & Snelling (1, CAS); BCS, 6 mi. SW of Santiago, 8/31/1959, Radford & Werner, light trap (2, UAIC); BCS, Cabo San Lucas, Hotel Finisterra, 9/8-14/1978, JP & KE Donahue (3, LACM); BCS, 19 mi. NW of Cabo San Lucas, 10/1/1967, GA Marsh (2, CAS); BCS, 5 mi. E of San Lucas, Playa Barco Varado, 4/11/1991, Ballmer & Mayor, at light (2, UCRC); BCS, Cabo San Lucas, 10/26/1941, ?F Gander (1, SDMC); BCS, 7.8 km E of Cabo San Lucas at km 7.8 on Hwy.1, 4/23/1979, DB Weissman, #79-91 (2, CAS); BCS, 7 mi. S of Punta Colorada (arroyo), 12/23-30/1987, N.Bloomfield, green dot (3, SDMC); BCS, Arroyo San Gregorio,13 air km WNW of La Purissima, 4/24-26/1983, Wasbauer & Slansky (4, CSCA); BCS, 27.7 mi. NE of Arroyo San Miguel, 4/1/1985, Bloomfield & Faulkner, green dot (2, CAS); BCS, Mulege, 8/22-23/1966, Ray Bandar (1, CAS); BCS, 6.4 mi. W Hwy. 1 to San Isidro, 3/21/1986, Faulkner & Bloomfield, green dot (2, CAS); BCS, 250 ft., JP & KE Donahue, #88,852 (3, CAS); BCS, Las Baracas,30 km E of Santiago, 4/1-6/1982, Paul DeBach, malaise trap (2, EMEC); BCS, Las Baracas,30 km E of La Ribera, 3/21-24/1982, Irwin & Schlinger (1, EMEC); BCS, Rancho Las Barracas 30 km E of Santiago, 11/6-8/1982, Irwin,Griswold & Schlinger (1, EMEC); BCS, Las Barracas, 5/13/1983, P DeBach, malaise trap (1, UCRC); BCS, Las Barracas, 6/9/1984, P DeBach, malaise trap (1, UCRC); BC, SW base Los Frailes, 2/18/1960, D Porter (1, USNM); BCS, Las Barracas, 4/16/1984, P DeBach, malaise trap (1, UCRC); BCS, San Francisco de la Sierra, 10/16/1997, DK Faulkner, green dot (1, SDMC); BCS, 1 mi. E El Triunfo, 10/10-13/1989, N.Bloomfield, green dot (3, SDMC); BCS, Las Barracas, 9/5/1983, Thomas & Olson (1, UAIC); BCS, Los Barriles, 5/3-4/1979, M.Wasbauer, at light (1, CSCA); BC, 7 mi. S Punta Colorada (arroyo), 12/23-30/1987, N.Bloomfield, green dot (2, CAS); BC, Loreto, 12/7/1977, 23.16N (1, SDMC); BC, 1.2-5.4 mi. N of Santa Ines, 12/5-9/1967, N.Bloomfield, green dot (3, CAS); BC, Sierra Juarez, Tajo Canyon, 4/2/1953, green dot (1, CAS); BC, Cantiles (Tajo) Canyon, 4/20/1955, 32.17N (1, SDMC); BC, L.Cantillas Cyn. Sierra Juarez, 3/20/1967, Opler & Powell (2, EMEC); BC, 7 mi. SE of San Quintin, 4/20/1947, CF Harbison (2, SDMC); BCS, Puerto San Tomas, 8/29/1967, M.Lieberman (5, CAS); BC, Isla de Cedros, canyon SW of Punta Norte, 4/2/1983, green dot (2, CAS); BC, Isla de Cedros, canyon SW of Punta Norte, 3/31/1983, green dot (2, CAS); BC, 5-7 km NW of Catavina on Hwy.1, 8/5/1981, Lightfoot & Weissman, #81-68 (1, CAS); BC, 3.5 mi. NNW of Catavina on Hwy. 1, 9/2/1985, 2000 ft., JP & KE Donahue, #96,262;#89,284 (3, CAS); BC, 2.6 mi. SE of Catavina, 3/23/1981, Faulkner & Andrews, green dot (2, SDMC); BC, 3.2 km N of Catavina on Hwy.1, 7/9/1978, Weissman & Lightfoot, Stop 32 (1, CAS); BC, 7.3 km NW of Catavina, 9/15/1988, 2000 ft., JP & KE Donahue, #124,936 (1, LACM); BC, 2 mi. N of El Rovenir, 4/6-7/1961, AG Smith, gas lantern (1, CAS); BC, Bahia de los Angeles, 5/10/1952, JP Figg-Hobyn (1, CAS); BC, 13 mi. SW La Zapopita, 6/14/1963, EL Sleeper (1, CAS); BC, Bahia de Las Animas, 9/5/1985, sea level, JP & KE Donahue, #96,431 (1, CAS); BCS, Sierra de la Giganta, mouth of Arroyo Comondu,16.4 mi. NE of La Poza Grande, 9/17/1985, 400 ft., JP & KE Donahue, #97,621 (1, CAS); BC, Puertecitos, 11/28/1964, G Sphon (2, CAS); BC, 1.8 km NE of Millers Landing (Beach dune), 5/27-28/1973, 35 m, EL Sleeper, 28114cb (1, CAS); BC, 38 km NW of Bahia de Los Angeles on Hwy.1, 8/6/1981, 290 m, Lightfoot & Weissman, #81-72 (4, CAS); BC, Punta San Fermin, 4/7-10/1971, EL Sleeper, black light (4, CAS); BC, Isla de Cedros, Cerro de Cedros, 7/1/1983, 183 m, Weissman & Lightfoot, #83-83 (1, CAS); BCS, 9 mi. N of Cabo San Lucas, 9/9/1988, EG Riley, black light (2, TAMU); BC, nr. La Zapopita de Trinidad, 4/9-14/1961, FS Truxal (1, LACM); USA, CA, In-Ko-Pah Gorge, E.Jacumba, 4/15/1942, 2000 ft., HR Roberts (3, ANSP); BCS, Playa San Lucas 11 road mi. S of Santa Rosalia, 9/4/1983, sea level, JP & KE Donahue (2, CAS); BCS, 3 mi. NE San Isidro (La Purisima), 4/2/1985, Bloomfield & Faulkner, green dot (2, CAS); BC, 6 mi. N of Guerrero Negro, 10/13/1981, Andrews & Faulkner, green dot (1, SDMC); BC, 7 km N of Guerrero Negro on Hwy.1, 7/10/1978, Weissman & Lightfoot, Stop 34 (1, CAS); BC, Rancho Union, 4/17/1947, CF Harbison (2, SDMC); BCS, Las Barracas, 10/15/1985, P DeBach, black light (3, UCRC); BCS, Las Barracas 30 km E of Santiago, 12/1-7/1982, P DeBach (1, EMEC); BCS, Las Barracas 30 km E of Santiago, 11/6-8/1982, Irwin,Griswold & Schlinger (3, EMEC); BCS, Las Barracas, 11/21/1984, P DeBach, black light (1, UCRC); BCS, La Paz, 8/22/1941, F.Ander (3, SDMC); BCS, 7 mi. SW of La Paz, 8/6/1966, Linsley,Chemsak & Hurd, at light (4, CSCA); BCS, 14 mi. NW of La Paz (in Cardon area), 4/23/1974, R Hardy (1, CSCA); BC, San Vincente, 5/14/1938, CE Norland (1, LACM); BC, Canyon del Tajo, Sierra Juarez, 4/1/1953, J Powell, at light (1, EMEC); BCS, S. Santa Rosalia (?), 5/30/1973, black light (1, CSLB); BC, 1 mi. S of Mulege, 8/27/1959, Radford & Werner, light trap (2, UAIC); BC, N of Guerrero Negro, 6/19-20/1973, 280 m, EL Sleeper, black light (1, CAS); BC, 20 km N of Ensenada on Hwy. 1, Weissman & Lightfoot, Stop 23 (1, CAS); BC, 4 mi. NW Rancho San Juan, 4/3-4/1961, AG Smith, gas lantern (1, CAS); BC, nr. Km 31 sign on Hwy.1, 9/19/1979, Weissman, Lightfoot & Love, #79-173 (1, CAS); BC, Diablito Canyon, E face of San Pedro Martir, 4/5/1973, SL Szerlip, at light (1, EMEC); BC, 0.4 km W of km 60 on road to Sierra San Pedro Martir NP off Hwy.1, 7/26/1978, 752 m, Weissman & Lightfoot, Stop 86 (1, CAS); BC, km 59.7 on road to San Pedro Martir NP, 8/20/1995, 1010 m, Weissman & Lightfoot, Stop 95-68 (1, CAS); BC, Mesa La Pitahaya, 9/2/1988, 30.00N, 115.35W, 1000 ft., JP & KE Donahue, #124,134 (2, LACM); BC, 9.4 km W of Penjamo, 6/20/1973, 550 m, EL Sleeper (2, CAS); BC, 19 mi. SW Campo Alfonsina (canyon), 10/27-28/1987, N Bloomfield, green dot (1, SDMC); BC, 14.4 mi. S Campo Alfonsina, 10/20-26/1987, N Bloomfield, green dot (6, SDMC); BC, Baia San Luis Gonzaga, 4/3/1973, Doyen,Powell & Szerlip, at light (1, EMEC); BC, 1.5 mi. N of Rancho Punjamo, 8/14/1971, Real & Main, UV light (2, CAS); BC, San Bartolo, 10/24/1941, 23.45N, FF Gander (1, SDMC); BC, 24 mi. N Bahia San Luis Gonzaga, 4/14/1962, EL Sleeper, black light (2, CAS); BC, Santo Tomas, 7/8/1953, WJ & JW Gertsch (1, AMNH); BC, Arroyo 13 mi. N of San Ignacio, 4/2/1961, AG Smith (3, CAS); BC, Punta Prieta, 3/17/1947, 28.56N, CF Harbison (2, SDMC); BC, 12 mi. E of El Rosario, 6/10/1979, black light (1, UCRC); BC, Canyon de Guadalupe, 5/3/1964, EM Fisher (1, LACM); BC, 10 mi. NNW of Catavina on Hwy.1, 9/1-2/1983, 2400 ft., JP & KE Donahue (1, CAS); BCS, 1.4 km S of turnoff to La Poza Grande, 7/15/1978, Weissman & Lightfoot, Stop 54 (1, CAS); BCS, Playa los Cerritos,11.2 mi. S Todos Santos, 9/28/1981, Andrews & Faulkner, Dark brown, green dot, black light (3, SDMC); BCS, Playa los Cerritos, 10/8/1983, Andrews & Faulkner, Dark brown, green dot, black light (1, CAS); BCS, San Jose del Cabo, G Eisen (2, ANSP); BCS, San Jose del Cabo (5, ANSP); BCS, San Jose del Cabo, Eisen, Abdomen Mount B1:2A (5, ANSP); BCS, San Jose del Cabo, Eisen, Abdomen Mount B1:3 (1, ANSP); BCS, San Jose del Cabo, Eisen (2, CAS); BCS, San Jose del Cabo, Eisen, Abdomen Mount B1.2 (1, ANSP); BCS, Isla Magdalena, Howlands Lagoon, sand dunes, 7/8/1983, Weissman & Lee, Stop #83-93 (8, CAS); BCS, Isla Magdalena canyon I km NW of Puerto Magdalena, 8/8/1983, Faulkner & Lightfoot, Stop #83-94 (1, CAS); BCS, La Paz, 10/21/1979, WF Chamberlain (2, TAMU); BCS, San Miguel, 7/3/1938, Michelbacher & Ross (1, CAS); BC, 1.3 mi. NW of El Triunfo, 1/20/1959, HB Leech (1, CAS); BCS, Comondu, Haines (1, CAS); BCS, Sierra El Tasti, 10/?/1893, Eisen (1, CAS); BC, Catavina, 9/25/1941, Ross & Bohart (1, CAS); BC, Venancio, 7/17/1938, Michelbacher & Ross (2, CAS);, *Arenivaga* sp. undet. Det. FW Fisk 1980 (1, SEMC); BCS, Comondu, 3/?/1889, Haines (2, ANSP); BCS, Env. De la Paz,?/?/1914, L Digguet, Museum Paris (1, ANSP); BCS, Golfo de California, Isla Cerralvo, 4/15/1962, 24.14N, 109.51W, CF Harbison, Accn. No. 1576 (2, SDMC); BC, Golfo de California, Isla Cerralvo, canyon W side nr. Punta El Limon, 7/15/1985, Weissman, Lightfoot & Faulkner, S85-85 (4, CAS); BC, 6 mi. W of San Felipe, 6/4/1967, Davis & Webb (1, CAS); BCS, 44 mi. NW of Vizcaino on road to Bahia Tortuga, 8/22/1995, 85 m, Weissman & Lightfoot, Stop #95-72 (2, CAS); BCS, Comondu Viejo, 4/4-5/1980, D Davis (1, USNM); BC, Santo Domingo, 5/8/1938, WE Simonds (1, CAS); BCS, 3 mi. N of San Antonio, 10/9-10/1968, 1200 ft., Sleeper & Moore, black light (3, CAS); BCS, 12 km E of San Antonio, 12/27/1978, P. Rude, UV and white light (3, EMEC); BCS, Sierra de la Laguna,5 mi. S of San Antonio, 9/2-3/1983, Thomas & Olson (1, UAIC); BCS, Playa El Coyote, Bahia Concepcion, 9/8-9/1985, sea level, JP & KE Donahue, #96-807 (6, LACM); BCS, Playa El Coyote, Bahia Concepcion, 9/11-12/1988, 25 ft., JP & KE Donahue, #124,558 (9, LACM); BCS, La Paz, 10/6/1968, 50 ft., Sleeper & Moore, black light (3, CAS); BCS, La Paz, 12/19/1973, W.Middlekauff (2, EMEC); BCS, La Paz, 12/26/1974, W.Middlekauff (1, EMEC); BCS, Hwy.1,35 km W of La Paz, 8/25/1995, 340 m, Weissman & Lightfoot, #95-82 (2, CAS); BCS, 8 mi. SE of La Paz, 10/13/1968, 1000 ft., Sleeper & Moore, black light (1, CAS); BCS, 18 km SW of La Paz, 6/1/1973, 118 m, EL Sleeper, 24110CdI (CapeThormFor.) (1, CAS); BCS, 26 mi. W of La Paz, 8/11-13/1966, Chemsak, Doyen & Powell, black/white lights (5, EMEC); BCS, 7 mi. SW of La Paz, 8/6/1966, Linsley, Chemsak & Hurd, at light (3, EMEC); BCS, 26 mi. W of La Paz, 8/11/1966, JT Doyen (1, EMEC); BCS, Sierra El Tasti, 10/?/1893, Eisen (2, ANSP); BCS, 0.5 km N of La Paz on Hwy.1, 7/16/1978, Weissman & Lightfoot, Stop 59 (7, CAS); BCS, La Paz across street from Hotel Posado, 4/21/1979, D.Weissman, #79-85 (5, CAS); BCS, 25 mi. W of La Paz, 8/30/1959, Radford & Werner, light trap (3, UAIC); BCS, La Paz, 11/9/1941, 24.10N, CF Harbison (6, SDMC); BCS, Grounds of Guaycura Hotel, 12/4-6/1961 (4, USNM); BCS, Grounds of Guaycura Hotel, 11/3/1961, (1, USNM); BCS, La Paz, 3/24/1958, K Bechtel (1, USNM); BC, El Barril, 3/27/1947, 28.16N, CF Harbison (1, SDMC); BC, Las Flores, LA Bay, 4/12/1947, CF Harbison (1, SDMC); BC, 14.4 mi. S of Campo Alfonsina, 10/20-26/1987, N Bloomfield, green dot (2, SDMC); BC, Aquajito Spring, Valle de la Trinidad, 7/?/1927, CF Harbison (1, SDMC); BC, Nr. La Zapopita, Valle de Trinidad, 4/9-14/1961, FS Truxal (1, LACM); BCS, 8 km W of La Paz, D Weissman, #79-84 (1, CAS); BCS, 22 mi. W of La Paz, 6/25/1967, Sleeper & Fisher, black light (1, CAS); BC, Santa Rosalia, 10/20/1930, EH Quayle (1, SDMC); BCS, Pichilingue Bay N of La Paz floating on water, 4/16/1974, RA Lewin (1, CAS); BCS, 3 mi. NE of San Isidro, 4/3/1985, Bloomfield & Faulkner, green dot (1, CAS); BCS, 1 mi. N of lighthouse on Cabo Falso, vegetated dune, 9/29/2003, Bill Warner, (1, WB Warner); BCS, Playa San Cristobal, 4/16/1984, Brown & Dodero, green dot (1, CAS); BCS, Cabo San Lucas, 6/11/1973, black light (2, CAS); BCS, 8/1-2/1978, 60096.8,black light (1, CAS); BCS, 9 mi. N of Cabo San Lucas, 9/15/1988, EG Riley, black light (1, TAMU); BCS, 3.3 mi. S of El Cien, 9/26/1981, Faulkner & Andrews, green dot (1, SDMC); BCS, 1.4 km S of turnoff to La Poza Grande N of Villa Insurgentes, 7/15/1978, Weissman & Lightfoot, Stop 54 (2, CAS); BCS, Hwy.1, 12 mi. NE of Villa Insurgentes, 9/7/1983, 250 ft., JP & KE Donahue (3, CAS); BCS, La Paz, 7/28/1969, W Middlekauff (1, EMEC); BCS, La Paz, 11/7/1941, 24.10N, FF Gander (1, SDMC); BCS, La Paz, 11/9/1941, 24.10N (2, SDMC); BCS, Isla Santa Margarita, NW island sand dunes, 7/7/1983, Faulkner, green dot (1, CAS); BCS, Isla Santa Margarita, sand dunes 3 km SW of Puerto Cortes, 7/7/1983, Weissman & Lightfoot (4, CAS); BCS, 8.8 mi. E of San Ignacio,4.3 mi. N of KP 59.5, 9/7/1985, 650 ft., JP & KE Donahue, #96,707 (1, CAS); BCS, Hwy.1, 0-4 km N of San Ignacio, at night, 7/12/1978, Weissman & Lightfoot, Stop 42 (1, CAS); BCS, 4.2 mi. W of Miraflores, 9/30/1981, Andrews & Faulkner, green dot (2, SDMC); BCS, Road to San Pedro De la Soledad, off Hwy.1, 8/27/1995, 23.14N, 109.57W, 820 m, Weissman & Lightfoot, Stop #95-87 (4, CAS); BCS, Isla Monserrate, canyon area W side, middle of island, 7/20/1984, Weissman & Lightfoot, S84-40 (2, CAS); BCS, Isla San Marcos, 7/17/1985, Weissman & Lightfoot, Stop #85-86 (1, CAS); BCS, Isla del Espiritu Santo, 7/8/1985, Weissman & Lightfoot, #85-76 (1, CAS); BCS, Isla Santa Cruz, 7/10/1985, Weissman & Lightfoot, #85-78 (2, CAS); BCS, 1st wash on road to Miraflores off Hwy.1, 1/3/1979, Weissman,Love,Lee & Mullinex, Stop 79-27 (1, CAS); BCS, 1.5 mi. NW of Miraflores, 10/28-29/1968, 700 ft., Sleeper & Moore, black light (2, CAS); BCS, 4.3 mi. SW of Miraflores, 9/12-13/1988, EG Riley, black light (2, TAMU); BCS, 0.8 km W of Hwy.1 on road to Miraflores, 9/26/1979, 210 m, Weissman,Lightfoot & Love (2, CAS); BCS, 0.25 km S of Miraflores turnoff on Hwy.1, 4/24/1979, D.Weissman, #79-93 (3, CAS); BCS, 64.5 km E of Villa Insurgentes on Hwy.1, 9/29/1979, #79-213 (1, CAS). Determiner label *Arenivaga rehni* Hopkins 2011” [white label with black border].

##### Distribution.

This species is found the length and breadth of the Baja peninsula and adjacent islands as well as isolated records in far southern California. See Fig. 134.

##### Diagnosis.

*Arenivaga rehni* is a highly variable species phenotypically. Its genitalia superficially resembles that of *Arenivaga grata* but *Arenivaga rehni* may be diagnosed by the narrow central field and broad heavily shagreened concavity interior to the point of articulation on the right dorsal phallomere, and the posterior projection on the lobe of the right ventral phallomere. See Fig. 133.

##### Description.

**Male.** *Measurements*. Holotype stand-in TL = 19.0 mm, GW = 8.3 mm, PW = 5.89 mm, PL = 4.23 mm, TL/GW = 2.29, PL/PW = 0.72. EW = 0.25 mm; OW = 0.45 mm. Among paratypes range of TL 15.3–26.7 mm; range of GW 6.9–11.4 mm; range of PW 5.18–7.83 mm; range of PL 3.90–5.27 mm.

**Figure 132.**
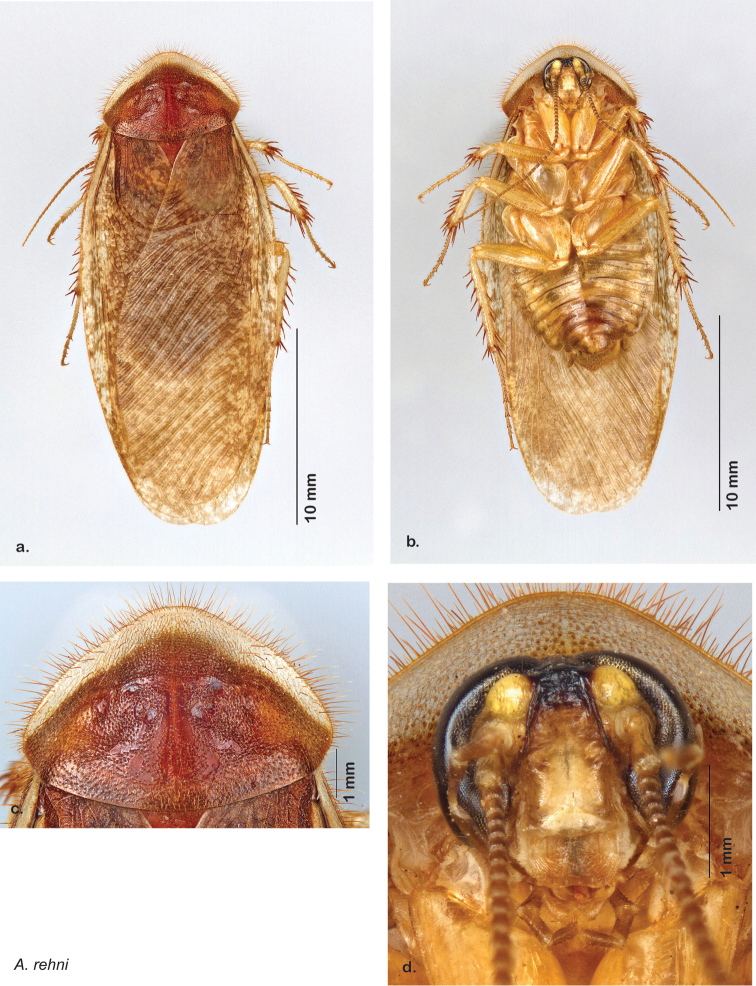
*Arenivaga rehni* **a** dorsal habitus **b** ventral habitus **c** pronotum **d** head.

*Head*. Two ocelli large, ovoid and protruding (0.40 × 0.30 mm), with one long seta in middle of upper ocellar rim; vertex very dark brown, with small ridges between apices of eyes extending on to ocellar tubercles, scattered short setae; interocellar space concave, very dark brown. Frons light brown, concave; bound on either side by dark brown ridges extending from inner apex of ocelli outwards to lateral edges of clypeus; scattered long setae on ridges. Anterior portion of frons light brown, bulbous; clypeal suture demarcates light brown anteclypeus. See Fig. 132d.

*Pronotum*. Pronotum translucent waxy beige; variable length orange-brown setae along anterior margin; dorsal surface of pronotum covered with short orange-brown setae that are denser and longer anteriorly and laterally; pronotal pattern dark orange-brown “panther face”, with no discernible detail and complete aura laterally and posteriorly; color of pattern and extent of aura highly variable within species; color from light orange-brown through all shades to very dark brown but always with no detail discernible; aura missing or complete laterally and posteriorly. See Fig. 132c.

*Body*. Wing brace absent. Legs and body light to medium orange-brown; subgenital plate asymmetrical with posterior edge emarginated, rounded apices, often with one or both styli present in very rudimentary form. See Fig. 132b.

*Forewings*. Wings extended beyond abdominal apex (up to ~40% of total wing length); medium brown with darker blotches; color highly variable in species from light brown, through all shades to dark brown; always blotchy; surface opaque and matte. See Fig. 132a.

**Figure 133.**
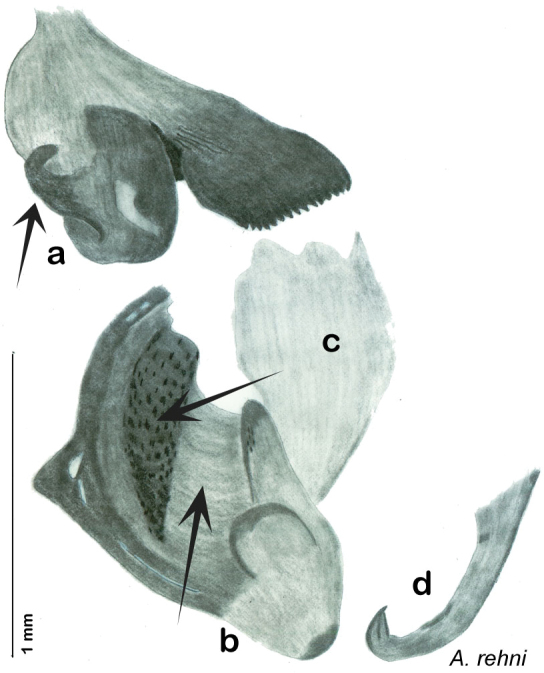
*Arenivaga rehni*, genitalia: a) right dorsal phallomere **b** right ventral phallomere **c** small central sclerite **d** genital hook. Arrow(s) indicate diagnostic characters (see text).

*Genitalia*. Right dorsal phallomere composed of bulbous lightly sclerotized lobe, articulated with right ventral phallomere on lateral side; central field narrow, lightly sclerotized; medial margin with small punctate region anteriorly, otherwise, smooth, lightly sclerotized and contiguous with medial margin of bulbous lobe; wide, heavily shagreened to toothed, concavity interior to point of articulation. Small central sclerite nondescript in shape, flat, punctate. Right ventral phallomere extends from articulation to shagreened lobe with posterior pointing smooth projection; after moderate gap, broad, shagreened flange with toothed edge, extending to depth of rest of phallomere. Genital hook with broad pointed head and moderate hook with bent tip; arm robust. See Fig. 133.

**Figure 134.**
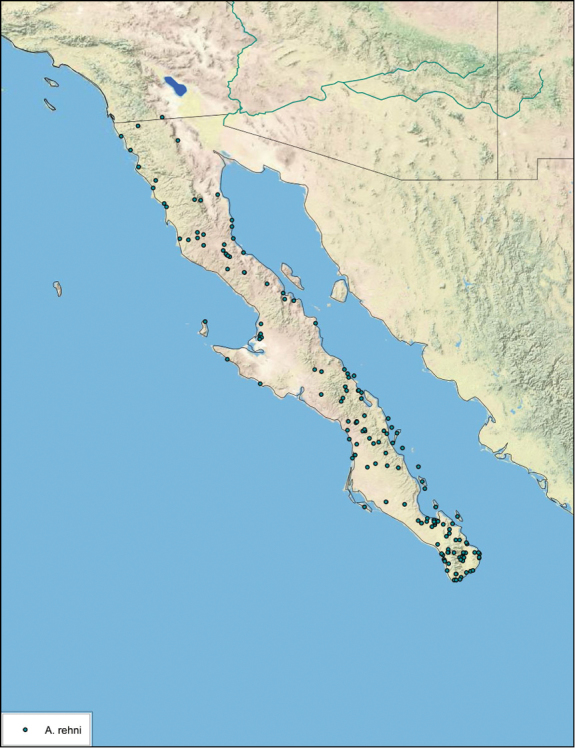
*Arenivaga rehni*, distribution.

##### Habitat and natural history.

All life history elements remain unobserved.

#### Arenivaga ricei sp. n.

http://zoobank.org/82FB4E20-4701-4A6A-960E-130B2A6F2CDC

http://species-id.net/wiki/Arenivaga_ricei

Figures 135 – 137

##### Type locality.

USA, Texas, Val Verde County, Langtry.

##### Material examined.

Holotype: ♂ in USNM labeled “Langtry, Tex., May 22, 1977, W.W. Walthol, R.R. Stewart “HOLOTYPE *Arenivaga ricei* Hopkins, 2012” [red label with black border].

Paratypes (8): USA: TX, Val Verde Co., 14 mi. NW of Del Rio,Hwy.90, 5/27/1972, RCA Rice, from rock dust in shallow caves along dry arroyo (4, USNM); TX, Langtry, died at Beltsville MD in captivity, Feb. and Apr. 1978, from Joann Alexander (2, USNM); TX, Langtry, 11/6/1976, Walthol & Stewart (1, USNM). All paratypes labeled “Paratype *Arenivaga ricei* Hopkins 2012” [blue label with black border].

##### Etymology.

The name is a noun in the genitive case. This species is named for Rob Rice, enthusiastic worldwide collector of Corydiid cockroaches, and collector of the first of this species.

##### Distribution.

This species is found along the Rio Grande River in Val Verde County, Texas. See Fig. 137.

##### Diagnosis.

*Arenivaga ricei* sp. n. is characterized by its short pumpkin seed-like shape and pale unmarked wings. While it is superficially similar to *Arenivaga darwini* and *Mylacris grolator*, it has two tarsal claws unlike *Arenivaga darwini*, which has one, and has genicular spines on the meso and metalegs unlike *Mylacris grolator* which has none.

##### Description.

**Male.** *Measurements*. Holotype TL = 12.9 mm, GW = 7.84 mm, PW = 5.7 mm, PL = 3.57 mm, TL/GW = 1.65, PL/PW = 0.63. This is the shortest species of *Arenivaga* in total length and with its curious shape has the lowest TL/GW ratio of any species. EW = 0.4 mm; OW = 0.5 mm. In paratypes, no notable variations in dimensions from those of holotype.

**Figure 135.**
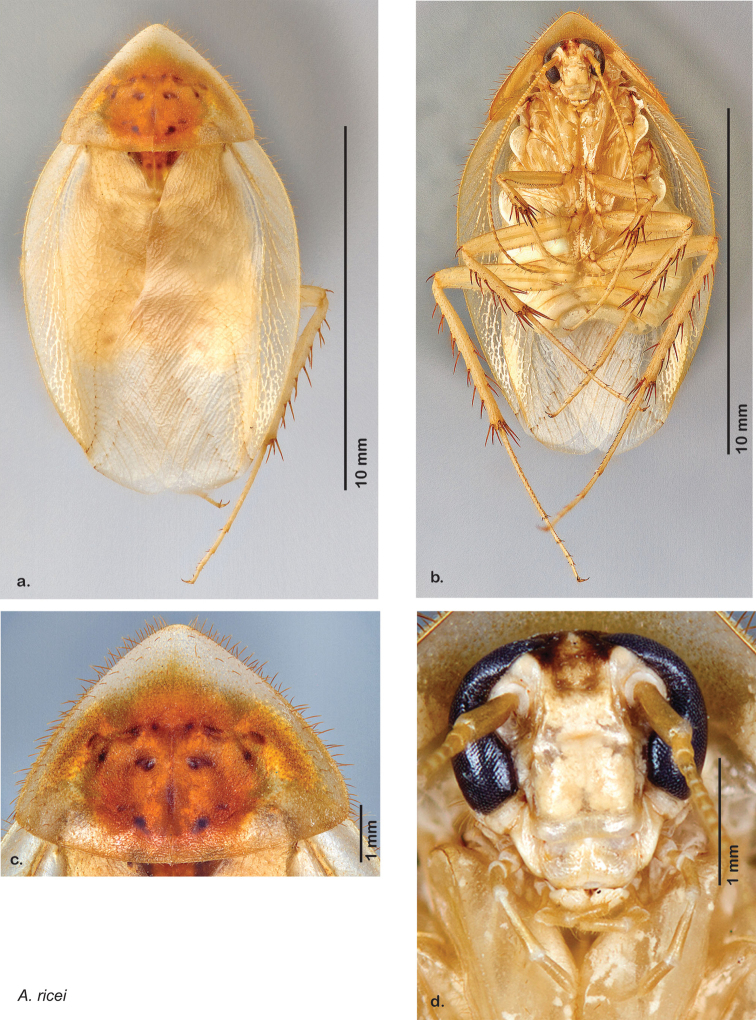
*Arenivaga ricei* **a** dorsal habitus **b** ventral habitus **c** pronotum **d** head.

*Head*. Two ocelli ovoid and not as protruding as on many species (0.3 × 0.20 mm); vertex brown with pale central line and small ridges in rays around upper apices of eyes; interocellar space concave, brown laterally fading to lighter brown towards center line. Posterior frons slightly tectiform horizontally, waxy white, smooth and shiny; anterior frons bulbous, waxy white, smooth and shiny; anteclypeus wide, smooth and waxy white. See Fig. 135d.

*Pronotum*. Pronotum translucent beige, anterior half of dorsal surface of pronotum also covered in fine pale setae with scattering of thicker orange-brown setae throughout; pronotal pattern orange-brown “hippo face” with extensive aura; some detail discernible. See Fig. 135c.

*Body*. Wing brace present. Two tarsal claws present. Legs and body pale brown; subgenital plate white with darker margin, asymmetrical, with rounded apices. See Fig. 135b.

*Forewings*. Wings extend only a short distance beyond abdominal apex (~ 20% of wing length); pale translucent brown with no sheen. See Fig. 135a.

**Figure 136.**
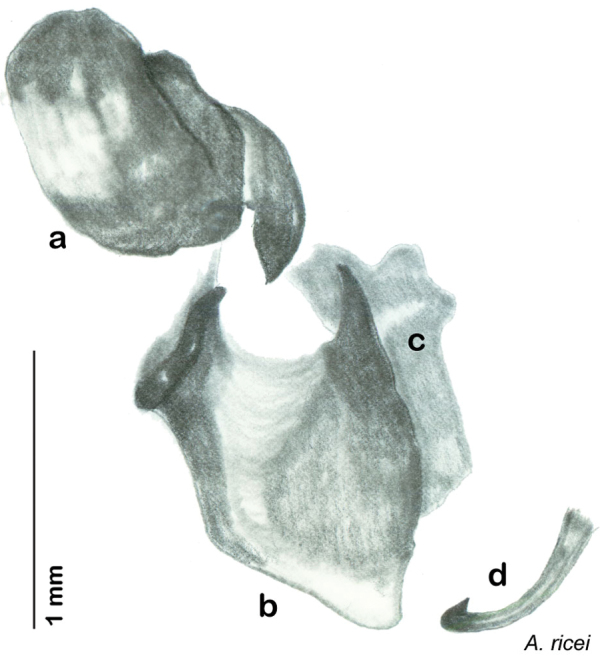
*Arenivaga ricei*, genitalia: a) right dorsal phallomere **b** right ventral phallomere **c** small central sclerite **d** genital hook.

*Genitalia*. Right dorsal phallomere composed of large bulbous lightly sclerotized hook-shaped lobe, articulated with right ventral phallomere on lateral side; anterior edge with small teeth leading to large ventrally projecting spine that is shagreened on its exterior surface. Small central sclerite is minutely punctate over entire surface with no sclerotized structures. Right ventral phallomere extends from articulation to form structure rounded at posterior apex but with corrugations at anterior apex, with rounded concave arm extending beyond depth of rest of phallomere; arm heavily sclerotized at its apex and shagreened over its external surface. Left phallomere unmodified. Genital hook with short extension to pointed head and slight dimple on short hook; arm gently curving. See Fig. 136.

**Figure 137.**
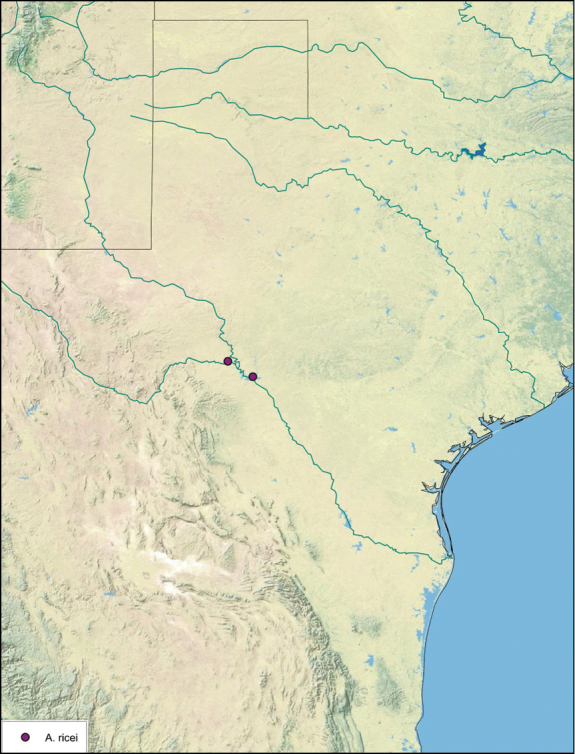
*Arenivaga ricei*, distribution.

##### Habitat and natural history.

This species occurs in terrain that is dry, hot, and dusty; it has been collected in shallow caves along dry arroyos. All other life history elements remain unobserved.

#### Arenivaga rothi sp. n.

http://zoobank.org/AE40A344-BA80-414C-BA4A-16A469412DBF

http://species-id.net/wiki/Arenivaga_rothi

Figures 138 – 140

##### Type locality.

MEXICO, Coahuila, 8 mi N Viesca.

##### Material examined.

Holotype: ♂ in EMEC labeled “MEX: Coah., sand dunes at Bilbao, 8 mi. N. Viesca, V-30/31-1981, J. Doyen, J. Liebherr, at blacklight” “HOLOTYPE *Arenivaga rothi* Hopkins, 2012” [red label with black border].

Paratypes (13): USA: TX, Presidio, May-June 1953, 53-10102, Presidio-3268L, at lights (8, USNM); TX, Presidio, 6/?-8/?/1955, JH Russell, at electric light (1, USNM); TX, Presidio, 6/17/1954, JH Russell, at light, 54-6842, Int. 3399L, (1, HEH); TX, Presidio, 5/14/1944, Presidio 1334, Lot No. 44-16214 (1, LACM). MEXICO: Coahuila, Bilbao 8 mi N of Viesca, 5/30-31/1981, Doyen & Liebherr, on sand at night (2, EMEC); Coahuila, 10 mi E of San Pedro de las Colonias, 7/3/1959, ER Tinkham, low dunes (1, USNM). All paratypes labeled “Paratype *Arenivaga rothi* Hopkins 2012” [blue label with black border].

##### Etymology.

The name is a noun in the genitive case. This species is named for the late Louis M. Roth who devoted his career to the description of new cockroach species and co-authored Cockroaches: Ecology, Behavior and Natural History.

##### Distribution.

This species is found in north central Mexico in the state of Coahuila up to Presidio County, Texas. See Fig. 140.

##### Diagnosis.

*Arenivaga rothi* may be distinguished by the very wide gap on the right ventral phallomere and the unusually narrow, pointed head on the genital hook. See Fig. 139.

##### Description.

**Male.** *Measurements*. Holotype TL = 21.0 mm, GW = 10.2 mm, PW = 6.64 mm, PL = 4.65 mm, TL/GW = 2.06, PL/PW = 0.70. EW = 0.20 mm; OW = 0.40 mm. Size range among paratypes: TL 17.9–21.8 mm, GW 8.2–10.4 mm, PW 5.70–7.00 mm, PW 4.40–4.82 mm.

**Figure 138.**
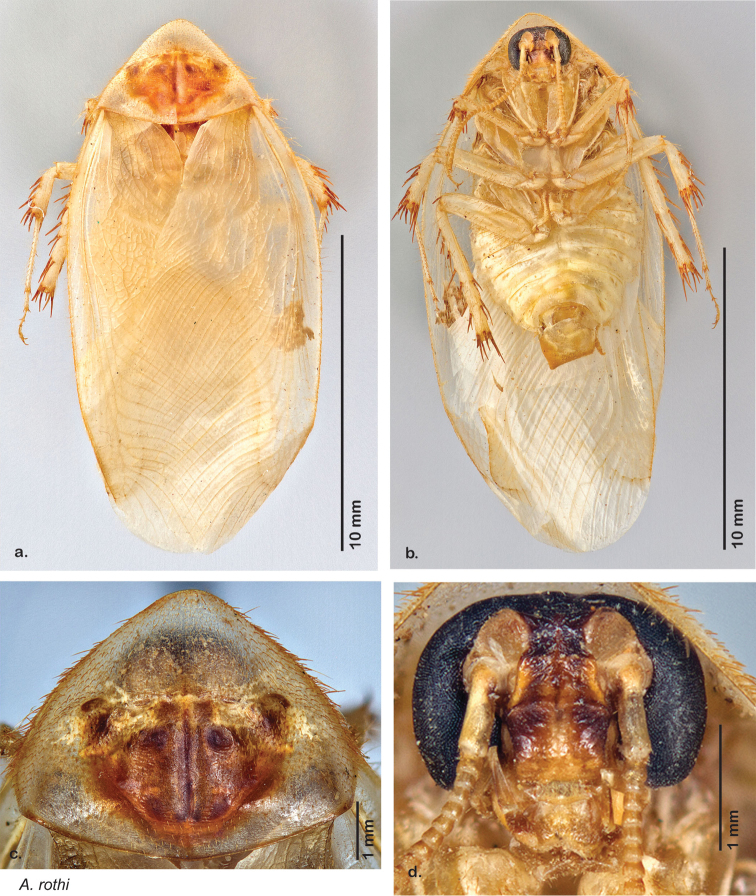
*Arenivaga rothi* **a** dorsal habitus **b** ventral habitus **c** pronotum **d** head.

*Head*. Two ocelli large, ovoid and protruding (0.50 × 0.40 mm); vertex dark brown, with small ridges between apices of eyes and extending onto ocellar tubercles; interocellar space concave, dark brown, light brown medially, with two pale spots medial to base of ocelli. Frons light brown; posterior concave; anterior portion of frons bulbous, light brown; wide light brown anteclypeus. See Fig. 138d.

*Pronotum*. Pronotum translucent waxy beige; dorsal surface of pronotum with dense very short light orange-brown setae that are thicker and longer laterally; pronotal pattern light orange-brown “hippo face” with little discernible detail; no aura. See Fig. 138c.

*Body*. Wing brace present. Two tarsal claws present. Legs and body light brown; sternites with small pale maculations and fine lines laterally on each; subgenital plate light orange-brown with darker border; asymmetrical with pointed apices. See Fig. 138b.

*Forewings*. Wings extended well beyond abdominal apex (up to ~35% of wing length); very pale translucent white-beige or brown; surface translucent and matte or with dull sheen. See Fig. 138a.

**Figure 139.**
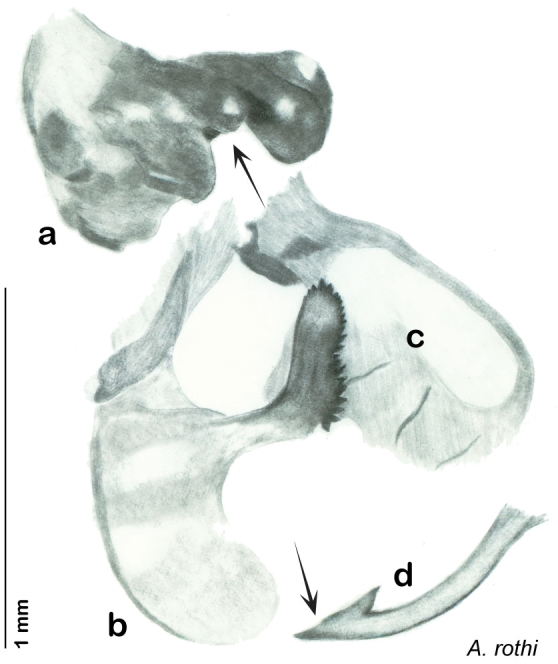
*Arenivaga rothi*, genitalia: a) right dorsal phallomere **b** right ventral phallomere **c** small central sclerite **d** genital hook. Arrow(s) indicate diagnostic characters (see text).

*Genitalia*. Right dorsal phallomere composed of lightly sclerotized, bulbous hook-shaped lobe, articulated with right ventral phallomere on lateral side; central field lightly sclerotized, punctate and deeply foreshortened; medial edge rolled outwards from central field, heavily sclerotized, shagreened to toothed. Small central sclerite lightly sclerotized, finely punctate, concave with posterior rim attaching to dorsal posterior point of dorsal phallomere, anterior end folded posteriorly into wide punctate cup. Right ventral phallomere arises from articulation to form smooth rounded lobe becoming sclerotized and shagreened medially and anteriorly; small shagreened fold in moderate gap followed by wide dorsally curving shagreened lip with central convexity. Folded anterior portion of left phallomere setose, otherwise unmodified. Genital hook with long extension to pointed head; short hook with slight dimple near point; arm smoothly curved and relatively short. See Fig. 139.

**Figure 140.**
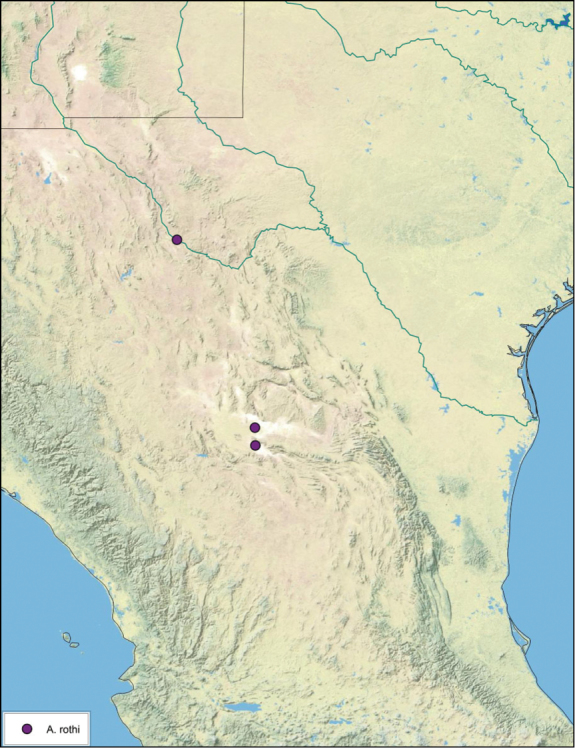
*Arenivaga rothi*, distribution.

##### Habitat and natural history.

All life history elements remain unobserved.

#### Arenivaga sequoia sp. n.

http://zoobank.org/30135D08-F2F6-4D53-981E-83DF37C7E05F

http://species-id.net/wiki/Arenivaga_sequoia

Figures 141 – 143

##### Type locality.

USA, California, Los Angeles County, Mt. Washington.

##### Material examined.

Holotype: ♂ in LACM labeled “CALIF., L. A. CO: Mt. Washington (LA), 22-26.vi.76, coll J P Donahue” “HOLOTYPE *Arenivaga sequoia* Hopkins, 2012” [red label with black border].

Paratypes (116): USA: CA, Glendale, 8/5/1989, WF Chamberlain (3, TAMU); CA, Glendale, 3/21/1947, EI Schlinger (1, FSCA); CA, Los Angeles Co., Big Tujunga Canyon, 4/22/1972 (1, LACM); CA, Los Angeles Co., San Pedro Defense Fuel Support Pt., ?/?/1996, 33.46.25N 118.18.5W, Rogers & Matori, Pitfall trap (1, UCRC); CA, Los Angeles Co., Coldwater Canyon, 7/15/1984, S Ziff (2, LACM); CA, Los Angeles Co., Los Angeles, Mt. Washington Dist., 6/1/2001, 34.05.910N 118.13.177W, 840 ft., JP Donahue (1, LACM); CA, Los Angeles Co., Pasadena, 7/3/2002, 34.13N, 118.13W, K McNassor (1, LACM); CA, Los Angeles Co., Westwood Hills, 4/8/1934, P Miller (1, ANSP); CA, Los Angeles Co., Los Angeles, Mt. Washington Dist., 6/22-26/1976, JP Donahue (1, LACM); CA, Los Angeles Co., Los Angeles, Mt. Washington Dist., 8/4/1983, 840 ft., JP & KE Donahue (1, LACM); CA, Los Angeles Co., Los Angeles, Mt. Washington Dist., 7/27/1976, 840 ft., JP & KE Donahue (1, LACM); CA, Los Angeles Co., Los Angeles, Mt. Washington Dist., 1/14/1976, 840 ft., JP & KE Donahue (1, LACM); CA, Los Angeles Co., Sulphur Springs Cmp., San Gabriel Mts., 7/28/1993, 5200 ft., AV Evans (1,LACM); CA, Altadena, 7/16/1975, RH Crandall Jr. (1,LACM); CA, Los Angeles Co., Pasadena, 7/8/2002, K McNassor (1, LACM); CA, Los Angeles Co., Franklin Canyon, 6/5/1966, S Ziff (1, LACM); CA, Eaton Canyon, San Gabriel Mts., 9/5/1963, RH Crandall Jr. (1, LACM); CA, Los Angeles Co., Hungry Valley, 4 air mi. S of Gorman, 7/16/1975, J Powell, at black light (1, EMEC); CA, Los Angeles Co., Burbank, 7/12/1960, FP Sala, Orthoptera Blattidae det. F.Sala, (3, HEH); CA, Los Angeles Co., Soledad Canyon, 9/17/1979, A Comproni II, Collection of C.Hamera, (1, SDMC); CA, Los Angeles Co., So. Pasadena, 7/28/1942, at light (1, LACM); CA, Los Angeles Co., Los Angeles, Mt. Washington Dist., 11/24/1975, 840 ft., JP & KE Donahue (3, LACM); CA, Los Angeles Co., Los Angeles, Mt. Washington Dist., 11/13/1975, 840 ft., JP & KE Donahue (1, LACM); CA, Los Angeles Co., Los Angeles, 8/31/1930, J Hornung (1, LACM); CA, Los Angeles, 7/?/1968, Guardian Pest Control (1, CSCA); CA, Los Angeles, Mt. Wilson, 8/10/1909, F Grinnell Jr. (1, ANSP); CA, Los Angeles Co., El Segundo Sand Dunes, 6/15/1938, WD Pierce, El Segundo Sand Dunes Biological Survey (3, LACM); CA, Los Angeles Co., El Segundo Sand Dunes, 7/13/1938, WD Pierce (1,LACM); CA, Los Angeles Co., El Segundo Sand Dunes, 5/20/1939, WD Pierce (1, LACM); CA, Los Angeles Co., Waldon Canyon, 7/26/1947, LE Myers, light (2, CSCA); CA, Los Angeles Co., Burbank, 7/24/1954, at light (2, LACM); CA, Los Angeles Co., El Segundo Sand Dunes, 4/28/1945, L Martin, Ericameria ericoides (1, LACM); CA, Whittier, 8/14/1949 (1, USNM); CA, Oak Grove Park, La Canada, 8/15/1950, GP Taylor (1, SDMC); CA, Santa Barbara, Aliso Canyon 6 mi. SW of New Cayuma, 7/9/1965, D Bragg (1, EMEC); CA, Loomis, 6/25/1939, D Meadows (1, LACM); CA, Temecula, 9/9/1930, JA Hornung (1, LACM); CA, LaGrange, 7/20/1962, RP Allen (1, CSCA); CA, Stanislaus Co., Turlock, 5/3/1970, RR Snelling (3, LACM); CA, Stanislaus Co., Raines Park, 8/2/1974, J Denk, ultraviolet light (1, SDMC); CA, Stanislaus Co., Turlock, 7/12/1959, RR Snelling, at light, 59G29-16 (2, CSCA); CA, Stanislaus Co., LaGrange, 7/27/1959, RP Allen, light trap, 59H14-1, *Arenivaga erratica* Rehn det. Buxton 1966 (1, CSCA); CA, Los Angeles Co., Southgate, 5/26/1939 (1, USNM); CA, Inyo Co., Sequoia NP, Potwisha, 6/18/1929, Van Dyke Collection (1, CAS); CA, Inyo Co., Sequoia NP, Potwisha, 7/16/1931, EO Van Dyke (1, CAS); CA, Inyo Co., Sequoia NP, Potwisha, 7/1/1941, EO Van Dyke (12, CAS); CA, Inyo Co., Sequoia NP, Potwisha, 6/13/1929, EO Van Dyke (3, CAS); CA, Inyo Co., Sequoia NP, Potwisha, 6/20/1929, EO Van Dyke (1, CAS); CA, Three Rivers, 8/5/1940, DE Hardy (1, ANSP); CA, Kernville, 7/24/1940, DE Hardy (1, ANSP); CA, Madera Co., San Joaquin Exp. R., 7/29/1953, HE Childs (1,USNM); CA, Coachella Valley, Snow Creek, 5/11/1952, ER Tinkham (1, USNM); CA, Los Angeles Co., Tanbark Flat, 7/3/1950, FX Williams (1, CAS); CA, Contra Costa Co., Antioch NWR, 7/10/1990, Hsu & Powell, lights (3, EMEC); CA, Contra Costa Co., Antioch, 1/24/1975, J Doyen, sifting sand, J.Doyen Lot 75 A1.1 (2, EMEC); CA, Contra Costa Co., Antioch NWR, 9/30/1981, J Powell (1, EMEC); CA, Contra Costa Co., Antioch, 7/3/1953, Marah & Schuster, *Arenivaga erratica* Rehn det. HF Strohecker (1, EMEC); CA, Madera Co., Lake Millerton, 5/29/1992, WF Chamberlain, at light (2, TAMU); CA, Madera Co., Millerton Lake RA, 5/10/1997, WF Chamberlain, at light (1, TAMU); CA, Walker Pass, 7/13/1961, EI Schlinger (1, UCRC); CA, Monterey Co., 4 mi. E of Arroyo Seco Guard Station, 5/9/1975, 650 ft., J Chemsak (1, EMEC); CA, Monterey Co., Marina dunes, 4/3/1988, GR Ballmer (1, UCRC); CA, Inyo Co., Sequoia NP Hosp. Flat Cpgd., 7/7/1973, LJ Orsak, black light (1, EMEC); CA, Ventura (1, LACM); CA, Santa Paula, 7/1/1939, (1, USNM); CA, San Diego Co., San Ysidro, 6/24/1969, light trap, plant quarantine division, USDA (1, CSCA); CA, Orange Co., 4 mi. E of Olive, 8/3/1980, JW Wilcox, black light (2, CSCA); CA, Laguna Beach, 7/10/1919 (1, USNM); CA, Fresno Co., Kerman, 8/3/1988, KS Hagen (2, EMEC); CA, Fresno Co., Piedra, 8/6/1982, RF Gill (1, EMEC); CA, Fresno Co., Pine Flat Dam, 6/3/1968, EA Kane (1, CSCA); CA, Sutter Co., Sutter Buttes, 2/6/1980-2/4/1981, AR Hardy, antifreeze pit trap (1, CSCA); CA, Kern Co., Frasier Park, 6/20/1948, AT McClay, *Arenivaga erratica* Rehn det. Strohecker (1, FSCA); CA, Kern Co., 11 mi. W & 1 mi. N of Wasco, 7/26-27/1965, JP Bruen, at light (8, EMEC). All paratypes labeled “Paratype *Arenivaga sequoia* Hopkins 2012” [blue label with black border].

##### Etymology.

The name is a noun in the nominative singular. This species is named for the fact that it occurs, amongst other places, in Sequoia National Park and Sequoia National Forest–the first desert sand roach species found in a forest.

##### Distribution.

This species is found throughout the western half of southern and central California and on one off-shore island. See Fig. 143.

##### Diagnosis.

*Arenivaga sequoia* can be identified by the narrow dorsally turning hook-shaped lobe on the right dorsal phallomere and the wide gap on the right ventral phallomere. See Fig. 142.

##### Description.

**Male.** *Measurements*. Holotype TL = 18.5 mm, GW = 9.4 mm, PW = 5.65 mm, PL = 3.90 mm, TL/GW = 1.97, PL/PW = 0.69. EW = 0.4 mm; OW = 0.5 mm. Among paratypes range of TL 13.6–22.0 mm; range in GW 6.1–10.0 mm; range in PW 5.00–6.65 mm; range in PL 3.30–4.64 mm.

**Figure 141.**
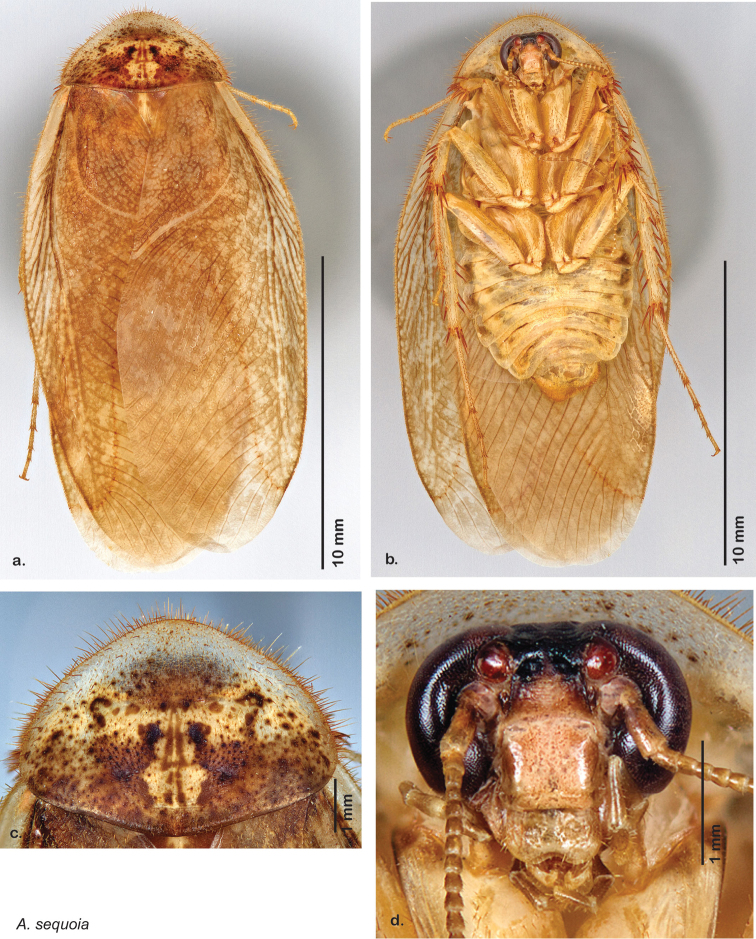
*Arenivaga sequoia* **a** dorsal habitus **b** ventral habitus **c** pronotum **d** head.

*Head*. Two ocelli large, ovoid and protruding (0.40 × 0.25 mm); vertex dark brown with small ridges in rays around upper apices of eyes and extending onto ocellar tubercles; interocellar space concave, dark brown anteriorly and laterally fading to light brown towards center and frons. Posterior frons concave; color of frons grades from narrow dark brown line at peak of ridges to light brown at center; faint vertical corrugations. Anterior frons bulbous with central indentation at posterior end; anteclypeus broad, flat, light brown. See Fig. 141d.

*Pronotum*. Pronotum translucent, waxy beige anteriorly shading to chestnut at posterior end; anterior half of dorsal surface of pronotum with short fine orange-brown setae with scattering of longer, thicker setae throughout; pronotal pattern light brown to brown “panther face” depending on specimen; brown maculations of same color as pattern scattered across posterior 70% of dorsal surface of pronotum; appearance of lateral and posterior aura beneath maculations. See Fig. 141c.

*Body*. Wing brace present. Two tarsal claws present. Legs and body light orange-brown, darker at joints, lateral edges of sternites, and posterior margin of subgenital plate; subgenital plate asymmetrical with rounded apices. See Fig. 141b.

*Forewings*. Wings extended well beyond abdominal apex (~40% of wing length); color varies from uniform brown, to blotchy brown, to light brown. See Fig. 141a.

**Figure 142.**
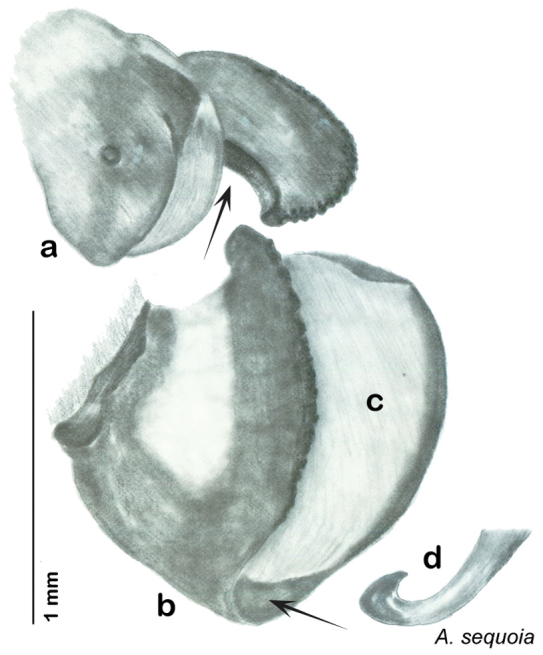
*Arenivaga sequoia*, genitalia: a) right dorsal phallomere **b** right ventral phallomere **c** small central sclerite **d** genital hook. Arrow(s) indicate diagnostic characters (see text).

*Genitalia*. Right dorsal phallomere composed of bulbous sclerotized hook-shaped lobe, articulated with right ventral phallomere on lateral side; central field lightly sclerotized; setae projecting inwards over central field from lateral rim; medial margin of lobe punctate gradating to heavily toothed edge. Small central sclerite flat, unmodified, finely punctate; right ventral phallomere extends from articulation to form structure rounded at posterior apex and expanding to punctate and more sclerotized area dorsally; attached anteriorly after wide gap is dorsally projecting flanged arm, shagreened with toothed edge. Left phallomere unmodified. Genital hook with moderate extension to pointed head with short hook; arm with shallow bend. See Fig. 142.

**Figure 143.**
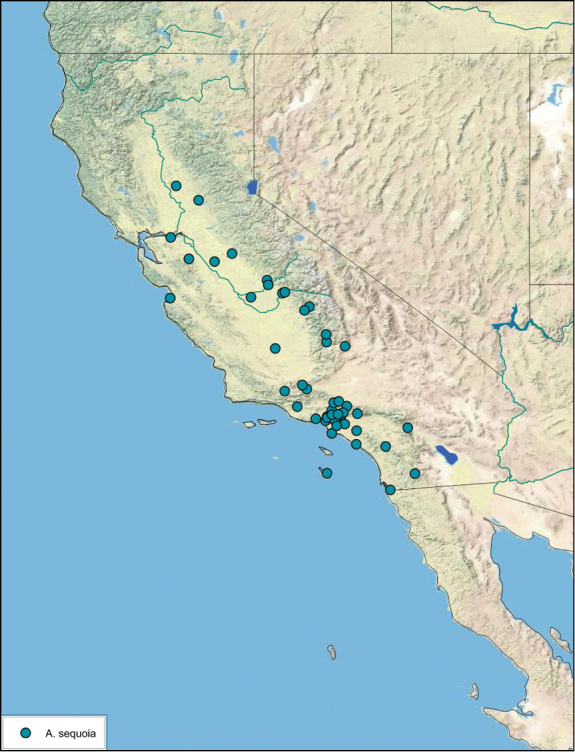
*Arenivaga sequoia*, distribution.

##### Habitat and natural history.

This species occurs from in varied terrain at elevations of 5 m to over 1700 m along the western slopes of the southern Sierra Nevada Mountains and westwards to the coast. All other life history elements remain unobserved.

#### Arenivaga tenax sp. n.

http://zoobank.org/5749CACF-DB41-4538-B1CD-4E7C58D91615

http://species-id.net/wiki/Arenivaga_tenax

Figures 144 – 146

##### Type locality.

USA, New Mexico, Otero Co., White Sands NM.

##### Material examined.

Holotype: ♂ in MSB labeled “USA: NM, Otero Co., White Sands NM, Interdune Veg., WSNMF, 4008’ UV trap, 32.46.64N 106.10.84W, 14 Jul 2010, EH Metzler” “HOLOTYPE *Arenivaga tenax* Hopkins, 2012” [red label with black border].

Paratypes (50): USA: AZ, Cochise Co., Willcox, 7/20/1970, S Kozloski, black light (16, ASUT); AZ, Portal, 6/28/1956, OL Cartwright, from No. 3 rat nest Neotoma (1, USNM); TX, Monahan Sandhills SP, 7/10/1994, WF Chamberlain, at light, *Arenivaga erratica* Rehn det. Stiehan 94 (1, TAMU); TX, El Paso, 6/28/1947, Cazier, D Rockefeller Exp. (1, AMNH); TX, El Paso Co., 12 mi NNE of Fabens, 4/23/1998, 31.40.31N 106.02.30W, EG Riley, UV light (2, TAMU); TX, El Paso Co., I10 rest stop 1 mi SE of Fabens, 7/1/1994, W & B Warner (1, WB Bill Warner); TX, El Paso Co., Clint, 6/9/1977, CR Burgess, light trap, *Arenivaga erratica* Rehn det. J.Stidham 98 (1, TAMU); TX, El Paso Co., Hueco Tanks, 5/15/1971, Murray & Gaumer, at light (1, TAMU); TX, El Paso Co., El Paso, 8/?/????, GW Dunn (1, ANSP); TX, El Paso Co., 7/17/1927, PA Readio (1, ANSP); TX, Culberson Co., Guadalupe Mts., 6/15/2002, W Reeves, *Arenivaga erratica* Rehn det. Roth 2002 (1, MCZ); NM, Bernalillo Co., Albuquerque, 6/8/1953, JR Stuntz, Truth or Consequences establishment thru Ralph E.Heal,e49,537288 (1, USNM); NM, Hidalgo Co., Rodeo, 8/4/1967, 4100 ft., LD Anderson (1, UCRC); NM, Las Cruces, 6/? or 7/?/1961, JH Russell, at lights (1, USNM); NM, Otero Co., White Sands NM, 7/14/2010, 32.46.64N 106.10.84W, EH Metzler, interdune vegetation (1, MSB); NM, Otero Co., White Sands NM, 6/20/2009, 32.46.69N 106.11.38W, 4000 ft., EH Metzler, interdune vegetation (1, MSB); NM, Dona Ana Co., Las Cruces, 7/26/1982, CA Sutherland (1, NMSU); NM, Dona Ana Co., Las Cruces, 10/5/1985, CA Sutherland, lite (1, NMSU); NM, Portales, 6/29/1965, LL Garcia (1, NMSU); NM, Sandoval Co., Coronado SP, 8/21/1985, Baumann,Huish,Nelson, Wells,Whiting,Bernalillo, *Arenivaga erratica* (Rehn) Det. AH Barnum 2010 (1, MLBM); NM, Dona Ana Co., Dona Ana, on side of house, 4/29/2002, J Grimes, Blattaria MISC (1, NMSU); NM, Dona Ana Co., Ft. Selden SP, 6/16/1979, CD Ferris (1, FSAC); AZ, Cochise Co., Hwy. 186 at Blue Sky Rd., 8/28-10/9/2011, 32.12.52N 109.46.54W, WB Warner, black cup barrier pitfalls (2, Bill Warner); AZ, Cochise Co., Hwy. 186 at Blue Sky Rd., 7/29-8/28/2011, 32.12.52N 109.46.54W, WB Warner, black cup barrier pitfalls (6, Bill Warner); AZ, Cochise Co., Birch Rd., 4.1 mi. E of Hwy. 191, 7/28-10/9/2011, 31.58.43N 109.46.41W, WB Warner, black cup barrier pitfalls (1, Bill Warner); NM, Mesilla Park, 7/12/1917 (1, ANSP); NM, Sheridan Canyon, Big Hachet Mountains, HA Pilsbry (1, ANSP); AZ, Cochise Co., SWRS, Cave Creek Canyon, Chiricahua Mts., 8/8/1961, 5400 ft., (Eades)taken with light at night (1, ANSP). MEXICO: Ahumada, 7/22/1952, RB & JM Salander, at light (1, ANSP). All paratypes labeled “Paratype *Arenivaga tenax* Hopkins 2012” [blue label with black border].

##### Etymology.

The name is an adjective in the nominative singular. This species is named from the Latin meaning tenacious.

##### Distribution.

This species is found in New Mexico, far northwestern Texas, far southeastern Arizona, and neighboring parts of Mexico. See Fig. 146.

##### Diagnosis.

*Arenivaga tenax* can be distinguished by the small medial spine on the medial margin of the right dorsal phallomere and the toothed distal end of that margin. See Fig. 145.

##### Description.

**Male.** *Measurements*. Holotype TL = 19.2 mm, GW = 8.1 mm, PW = 5.22 mm, PL = 3.89 mm, TL/GW = 2.37, PL/PW = 0.75. EW = 0.20 mm; OW = 0.30 mm. Among paratypes range of TL 19.2–21.7 mm; range of GW 7.8–10.0 mm; range of PW 5.22–6.61 mm; range of PL 3.89–4.33 mm.

**Figure 144.**
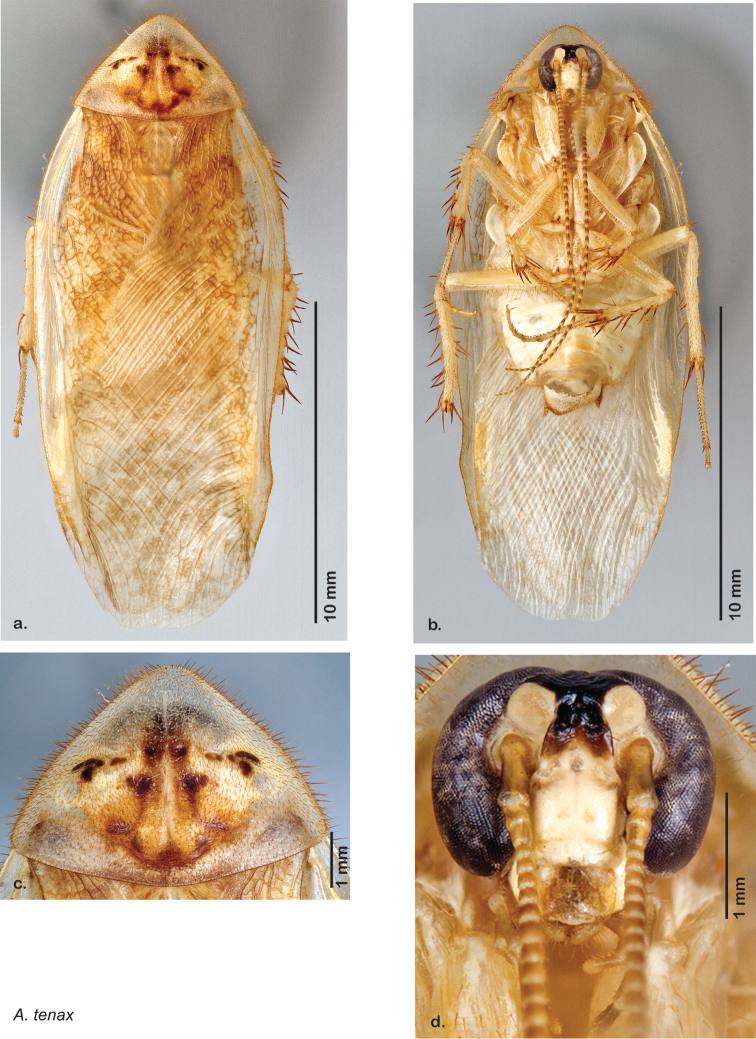
*Arenivaga tenax* **a** dorsal habitus **b** ventral habitus **c** pronotum **d** head.

*Head*. Two ocelli large, ovoid and protruding (0.40 × 0.30 mm); vertex dark brown with small ridges in rays around upper apices of eyes and extending onto ocellar tubercles; interocellar space concave, dark brown, with three small round indentations at points of an equilateral triangle. Posterior frons dark brown grading into waxy white; concave, smooth with anterior portion of frons bulbous and waxy white; waxy white smooth anteclypeus. See Fig. 144d.

*Pronotum*. Pronotum translucent waxy beige; dorsal surface of pronotum with short orange-brown setae that are thicker and longer laterally; pronotal pattern brown “panther face” with some discernible detail; no aura. See Fig. 144c.

*Body*. Wing brace present. Two tarsal claws present. Legs and body light brown, many specimens with brown maculations laterally on each sternite; subgenital plate light brown; asymmetrical with angular apices. See Fig. 144b.

*Forewings*. Wings extended well beyond abdominal apex (~40% of wing length); blotchy medium brown; surface opaque and matte or with very slight sheen. See Fig. 144a.

**Figure 145.**
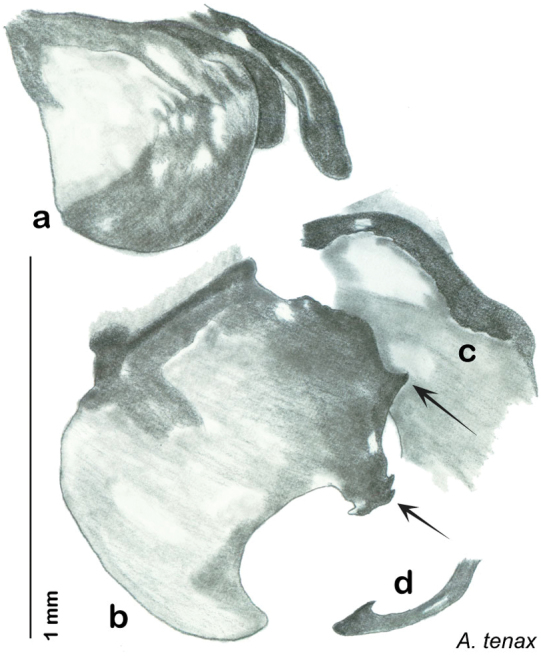
*Arenivaga tenax*, genitalia: a) right dorsal phallomere **b** right ventral phallomere **c** small central sclerite **d** genital hook. Arrow(s) indicate diagnostic characters (see text).

*Genitalia*. Right dorsal phallomere composed of lightly sclerotized bulbous hook-shaped lobe, articulated with right ventral phallomere on lateral side; central field lightly sclerotized; medial margin more heavily sclerotized, with short toothed region at posterior end, and short flat spine medially, otherwise smooth. Small central sclerite nearly flat and finely punctate, with raised sinuous line of teeth at lateral edge; right ventral phallomere extends from articulation to form smooth rounded structure, shagreened, corrugated and narrowed anteriorly; attached anteriorly is mildly dorsally projecting flanged concave punctate arm, with shagreened edge, that extends to depth of posterior portion of phallomere. Folded anterior portion of left phallomere setose, otherwise unmodified. Genital hook with moderate extension to pointed head with moderate hook; arm with smooth shallow curve. See Fig. 145.

**Figure 146.**
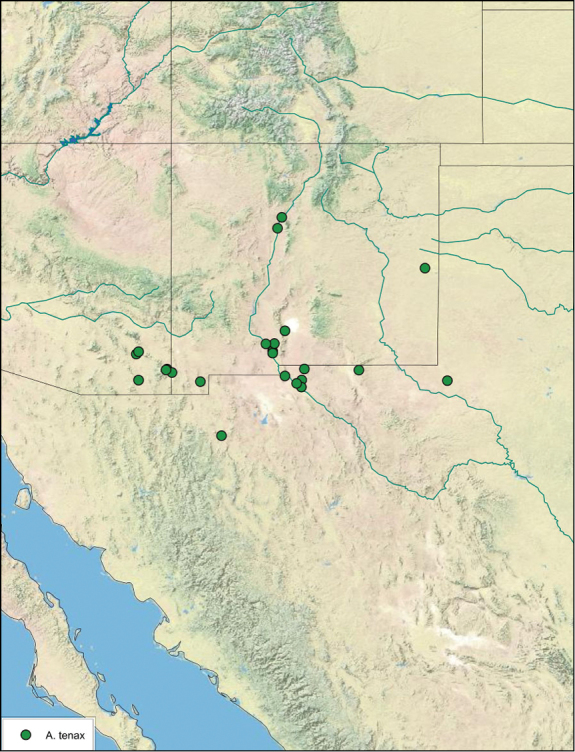
*Arenivaga tenax*, distribution.

##### Habitat and natural history.

All life history elements remain unobserved.

#### Arenivaga tonkawa

Hebard

http://species-id.net/wiki/Arenivaga_tonkawa

Figures 147 – 149

Arenivaga tonkawa Hebard 1920, Transactions of the American Entomological Society, 46(2), pp. 197–217.

##### Material examined

**(795).** USA: AZ, Pima Co., near Kits Pk. Baboquivari Mts., 8/7-9/1916, 32.00N, 111.36W, 3600’, (1, ANSP); AZ, Pima Co., Baboquivari Mts., 7/24/1941, B.Hodgden, (4, ANSP); AZ, Pima Co., Baboquivari Mts., Schaeffer Canyon (R&H), 9/18/1924, 5160’-5500’, (2, ANSP); AZ, Pima Co., near NW base of Baboquivari Mts. (R&H), 9/17/1924, 3000’, (2, ANSP); AZ, Pima Co., Baboquivari Mts., 7/24/1941, L.H.Banker, (5, ANSP); AZ, Pima Co., Baboquivari Mts., 7/24/1941, R.H.Beamer, (1, ANSP); AZ, Pima Co., Sabino Canyon, 7/31/1941, R.H.Beamer, (3, ANSP); AZ, Pima Co., Sabino Canyon, Santa Catalina Mts., 6/5/1916, J.F.Tucker, (1, ANSP); AZ, Pima Co., Kvitak, E of Quijotoa Mts. (R&H), 9/16/1924, 1530’, (1, ANSP); AZ, Pima Co., Lowell Ranger Sta., 7/6-20/1916, 32.185N, 110.49W, 2700’, (1, ANSP); AZ, Pima Co., roadside mine, Coyote Mts. (R&H), 9/14/1924, 2800’, (1, ANSP); AZ, Florence, 7/1/1922, C.R.Biederman, 1 specimen with A. erratica det. label by Hebard (2, ANSP); AZ, Pima Co., Ajo (R&H), 9/18/1922, 1800’, at light (1, ANSP); AZ, Pima Co., Sabino Basin Sta. Catalinas, 8/15-21/1916, 32.22N 110.46.5W, 3800’, share w/ Clark and ANSP, A.erratica det. label by Hebard (1, ANSP); AZ, Pima Co., Robles Pass, Tucson Mts. (R&H), 9/27/1924, 2700’-3000’, (3, ANSP); AZ, Madera Canyon, Santa Rita Mts., 7/26/1955, F.X.Williams, (2, CAS); AZ, Yuma Co., 4 mi. W of Salome, 6/8/1958, MacNeill & MacNeill, (2, CAS); AZ, Yuma Co., S Luis, 8/11/1940, E.C. Van Dyke, (1, CAS); AZ, Yuma Co., Palomas, 8/8/1917, C.U.Biol.Exp. (1, ANSP); AZ, 6 mi. S of Florence, 7/23/1924, E.P.Van Duzee, (7, CAS); AZ, Florence, 7/28/1917, Wheeler, (1, MCZ); AZ, Pinal Co., Oak Flat Cpgd. off US60, 7/18/2010, 33.18.28N 111.03.10W, Warner & Gruber, Bill Warner, headlamp & UV light (1, WB Warner ); AZ, Pinal Co., I8 10 mi. E of Gila Bend, 1/18-3/1-2002, Bill Warner (1, WB Warner); AZ, Mohave Co., Burro Creek Cpgd. 16 mi. S of Wikieup, 8/31/1991, Strange & Miller, (1, FSCA); AZ, Maricopa Co., 13401 N Scottsdale Rd. Scottsdale, 9/26/1968, R.D.Hill, (1, ASUT); AZ, Apache Junction, 4/25/1966, Brennan, blacklight (3, ASUT); AZ, Dome, 7/21/1924, E.P.Van Duzee, (3, CAS); AZ, Pima Co., Bog Springs Cpgd., Madera Canyon, 7/10/1976, D.Whitman #575, blacklight (1, EMEC); AZ, Santa Cruz Co., Madera Canyon, 7/24/1982, D.Colby, blacklight, von blockers, RCH013 (2, LACM); AZ, Santa Cruz Co., Madera Canyon, 8/2/1981, D.Colby, blacklight,RCH008 (1, LACM); AZ, Santa Cruz Co., Santa Rita Lodge, Madera Canyon, 7/27-28/1997, 31.72N, 110.87W, Evans & Russell, (1, LACM); AZ, Madera Canyon, Santa Rita Mts., 6/18-23/1962, 5000’, F.Werner, UV light trap (1, UAIC); AZ, Santa Cruz Co., Madera Canyon, Santa Rita Mts., 7/17/1980, R.H.Crandall, (2, LACM); AZ, Santa Cruz Co., Madera Canyon, Santa Rita Mts., 7/26-31/1965, R.H.Crandall, (3, LACM); AZ, Santa Cruz Co., Madera Canyon, Santa Rita Mts., 8/4/1981, R.H.Crandall, (2, LACM); AZ, Santa Rita Mts., 7/24/1979, R.H.Crandall, (1, LACM); AZ, Santa Rita Mts., 7/23/1978, R.H.Crandall, (1, LACM); AZ, Santa Cruz Co., Madera Canyon, Santa Rita Mts., 9/2/1959, 4880’, J.C.Franclemont, (2, CUIC); AZ, Santa Cruz Co., Madera Canyon, Santa Rita Mts., 7/10/1959, 4880’, J.C.Franclemont, (1, CUIC); AZ, Santa Cruz Co., Madera Canyon, Santa Rita Mts., 7/9/1959, 4880’, J.C.Franclemont, (1, CUIC); AZ, Santa Cruz Co., Madera Canyon, Santa Rita Mts., 7/14/1959, 4880’, J.C.Franclemont, (1, CUIC); AZ, Santa Cruz Co., Madera Canyon, Santa Rita Mts., 7/24/1959, 4880’, J.C.Franclemont, (1, CUIC); AZ, Santa Cruz Co., Madera Canyon, Santa Rita Mts., 7/8/1959, 4880’, J.C.Franclemont, (1, CUIC); AZ, Santa Cruz Co., Madera Canyon, Santa Rita Mts., 7/20/1959, 4880’, J.C.Franclemont, (1, CUIC); AZ, Santa Cruz Co., Madera Canyon, Santa Rita Mts., 7/31/1980, R.H.Crandall, (1, LACM); AZ, Pima Co., Tucson Mts., 8/11/1962, Knull & Knull, (1, OSUC); AZ, Yuma Co., Wellton, 8/9/1917, Corn.Univ.Exped. Lot 542, Sub 5? (1, CUIC); AZ, Yuma Co., Wellton, 6/13/19?9, L.L.Stitt, A-175,at light trap (1, USNM); AZ, Wickenburg, 8/20/1938, Knull & Knull, (5, OSUC); AZ, Yuma Co., Yuma, 6/1/1937, R.C.Dickson, (1, UCRC); AZ, Yuma Co., Yuma, 7/16/1957, V.Roth, at lights (1, UAIC); AZ, Yuma Co., Yuma, 4/29/1959, D.Muse, at lights (2, UAIC); AZ, Yuma Co., Yuma, 4/24/1959, D.Muse, at lights (1, UAIC); AZ, Yuma Co., Yuma, 7/14/1925, E.E.Russell, 4270 (1, ASUT); AZ, Yuma Co., Yuma, Sheep Tank Mine, Kora Mts., 10/29/1958, V.Roth, (1, UAIC); AZ, Yuma Co., Yuma, 5/14/1959, D.Muse, at lights (2, UAIC); AZ, Yuma Co., Yuma, 7/22/1925, (1, USNM); AZ, Yuma Co., 15 mi. SE of Alamo Crossing, 7/14/1962, Werner & Johnson, UV light trap (1, UAIC); AZ, Roll, 6/29/1939, L.L.Stitt, A. genitalis det. by A.B.Gurney (1, ASUT); AZ, Yuma Co., 11 mi. E of Wenden, 7/30/1965, K.W.Brown, at UV light (2, UCRC); AZ, Wickenburg, 7/8/1937, Knull & Knull, (3, OSUC); AZ, Pima Co., Sabino Canyon, Santa Catalina Mts., Summer 1967, J.Hessel, light trap (3, UAIC); AZ, Pima Co., Catalina SP, 7/27/1983, Barr & Barr, at light (1, EMEC); AZ, Pima Co., Sabino Canyon, Santa Catalina Mts., 8/9/1953, F.G.Werner, (1, UAIC); AZ, Yuma Co., Palm Canyon, Kofa Mts., 4/8/1963, R.L.Langston, (1, EMEC); AZ, Yuma Co., Palm Canyon, 4/6/1963, W.H.Ewert, (1, UCRC); AZ, Pima Co., Tucson, 7/6/1967, R.Steslak, (1, UAIC); AZ, Pima Co., 16 mi. SE of Tucson, 6/15/1964, A.G.Raske, at light (1, EMEC); AZ, S base of Tortolita Mts., 6/30/1984, R.S.Beal, black light (1, NAUF); AZ, Pima Co., Tucson, 9/8/1967, M.Druckenduod, (2, USNM); AZ, Pima Co., Tucson, 6/24/1944, R.A.Flock, (1, UCRC); AZ, Pima Co., Tucson, 8/4/1946, R.H.Crandall, (1, LACM); AZ, Pima Co., Sabino Canyon, 1/2/1964, Tauber & Toschi, (1, EMEC); AZ, Pima Co., Peppersauce Canyon, Santa Catalina Mts., 7/8/1961, P.H.Johnson, UV light trap (1, UAIC); AZ, Pima Co., Tucson, 7/12/1937, Knull & Knull, (3, OSUC); AZ, Pima Co., Tucson, 6/3/1937, Knull & Knull, (1, OSUC); AZ, Pima Co., Organ Pipe NM, 6/25/1965, (1, USNM); AZ, Pima Co., W slope of Chutum Vaya Canyon Baboquivari Mts., 8/4/1966, 31.431N, 111.37W, 3250’, F.Werner fam, light trap (1, UAIC); AZ, Salome, 7/25/1952, (1, USNM); AZ, Maricopa Co., Phoenix, 9/11/1942, P.C.Grassman, (1, UCRC); AZ, Pima Co., Tucson, Avra Valley Rd., 8/8/1998, Harrison & Sohns, (1, USNM); AZ, Pima Co., Tumamoc Hill, Tucson, 6/10-11/1967, R.Rice, rock pile (2, UAIC); AZ, Pima Co., Tucson, 10/10/1965, R.Rice, black light trap (2, UAIC); AZ, Pima Co., Tucson, 10/9/1965, R.Rice, under board, A.erratica det. label by R.Rice (1, UAIC); AZ, Gila Co., Globe, 6/2/1935, Parker, (1, MCZ); AZ, Gila Co., Parker Ranch, Six-shooter Canyon, Globe, 8/22/1932, Leech & Green, (1, CAS); AZ, Pima Co., Batamote Well, Ajo Valley (R&H), 10/16/1924, 1200’, (2, ANSP); AZ, Topock, 10/9/1917, O.C.Poling, (1, ANSP); AZ, Pima Co., Tucson, Jun/Jul 1910, L.C.Reynolds, A.erratica det. label by Hebard (1, CAS); AZ, Pima Co., St. Xavier Mon., 8/12/1924, J.O.Martin, (1, CAS); AZ, Pima Co., Tucson, 7/31/1993, Valentine & Valentine, A.erratica det. label by Roth (3, MCZ); AZ, Gila Bend, 8/20/1924, E.P.Van Duzee, (4, CAS); AZ, Maricopa Co., Maricopa Rd & I10, 7/4/1973, at light (4, ASUT); AZ, Maricopa Co., Maricopa Rd & I10, 6/27/1973, at light (1, ASUT); AZ, Maricopa Co., Tempe, 9/12/1971, C.Weller, on floor (1, ASUT); AZ, Maricopa Co., 7.5 mi. SSE of Bumble Bee, 9/17/1971, 2000’, Kolner & Covert, at light (1, ASUT); AZ, Santa Rita Mts., 7/26/1925, (4, USNM); AZ, Pima Co., Molino Basin, Santa Catalina Mts., 7/31/1968, F.Werner, (1, UAIC); AZ, Pima Co., Agua Caliente Cave,6 mi. E of Amado, 11/9/1968, 300’, W.D.Peachey, in from main entrance, very dry in cave, at least 1 other alive (1, UAIC); AZ, Pima Co., Molino Basin, Santa Catalina Mts., 7/28/1968, F.Werner, (1, UAIC); AZ, Santa Cruz Co., Madera Canyon, Santa Rita Mts., 7/10-26/1964, 5100’, D.R.Davis, Bog Spring Cpgd. (1, USNM); AZ, Pima Co., Tucson, Olson res., 8/2/1989, C.A.Olson, UV (2, UAIC); AZ, Pima Co., Tucson Mt. Park, caretaker’s house, 11/1/1981, S.Prchal, (1, UAIC); AZ (1, MCZ); AZ, Pima Co., Tucson, C.Bendier, (1, MCZ); AZ, Patagonia, Sonoita Cr., 10/14/1927, J.A.Kusche, (1, CAS); AZ, Yuma Co., Yuma, 7/27/1907, Hebard & Rehn, A.erratica det label by Hebard (1, ANSP); AZ, Roosevelt, Cornell Univ. Lot 445, Sub 5 (1, CUIC); AZ, Yavapai Co., Montezuma Well NM, 9/19/1993, S.M.Fondriest, site 24 (1, NAUF); AZ, Yavapai Co., Montezuma Castle NM, 8/1/1993, S.M.Fondriest, at light, site 23 (2, NAUF); AZ, Pima Co., Waterman Mts., 8/3/1980, Olson & VanDavender, UV, A.erratica det label by Olson (1, UAIC); AZ, Higley, 7/1/1917, (1, ANSP); AZ, Maricopa Co., Phoenix, 10/5/1904, M.Hebard, A.erratica det label by Hebard (1, ANSP); AZ, Maricopa Co., Phoenix, 5/9/1972, K.Mathieson, entrance to gopher hole (1, ASUT); AZ, Maricopa Co., Papago Park, Phoenix, 10/30/1970, L.McGill, (1, ASUT); AZ, Maricopa Co., Currey Corner, 5/27/1966, Brennan, black light (1, ASUT); AZ, Maricopa Co., Salt River, 5/2/1966, Brennan, black light (2, ASUT); AZ, Pima Co., Tucson, 10/28/1984, R.T.Huber, (1, UAIC); AZ, Pima Co., IBP:Santa Rita Range Res., Dest. Sample Plot, 6/29/1970, (1, UAIC); AZ, Pima Co., Tucson, Vic. of Ina & Oracle, 8/17/1987, W.L.Nutting, in pool (2, UAIC); AZ, Salome, 5/5/1920, O.C.Poling, A.erratica det label by Hebard (1, ANSP); AZ, Yuma Co., Yuma, 8/21/1930, Mrs.Smith, (1, ANSP); AZ, Maricopa Co., Phoenix, 10/2/1933, R.H.Crandall, (1, LACM); AZ, Maricopa Co., Phoenix, 4/19/1933, R.H.Crandall, (1, LACM); AZ, Maricopa Co., Phoenix, 10/30/19?3, R.H.Crandall, (1, LACM); AZ, Maricopa Co., Phoenix, 10/2/1933, R.H.Crandall, (1, LACM); AZ, Maricopa Co., Cave Creek, 10/9/2004, E.Pelton, (1, NAUF); AZ, Parker, 8/10/1942, R.A.Flock, (1, UCRC); AZ, Maricopa Co., Phoenix, 10/15/1933, R.H.Crandall, (1, LACM); AZ, Maricopa Co., Gila Bend, 9/12/1957, R.C.Dickson, (2, UCRC); AZ, Maricopa Co., Aguila, 8/21-22/1927, Cornell Univ. Lot 542 Sub 330,A.erratica det label by Hebard (1, CUIC); AZ, Florence, 7/28/1917, Cornell Univ. Lot 882 Sub 145 (1, CUIC); AZ, Maricopa Co., Sept. 1970, W.Mastriani, (1, EMEC); AZ, Maricopa Co., Tonopah, 7/20/2000, 33.29.40N 112.56.11W, 360m, D.Yanega, at light (2, UCRC); AZ, Maricopa Co., Scottsdale, 10/13/1991, R.M.Gillmore, at light (1, FSCA); AZ, Maricopa Co., Mesquite Flat, 7/6/1968, Noler & Burger, UV light (1, HEH); AZ, Maricopa Co., Phoenix, 9/11/1942, P.C. Grassman, (1, UCRC); AZ, Maricopa Co., 23rd Ave. Sewage Treatment Plant 3R, 9/17/1979, (3, UAIC); AZ, Maricopa Co., Phoenix, 8/1/1965, R.S.Beal, at light (1, NAUF); AZ, Coconino Co., Walnut Canyon 6 mi. ESE of Flagstaff, 8/7/1964, 6500 ft., JG Franclemont, (2, CUIC); AZ, Coconino Co., Walnut Canyon 3 mi. E of Flagstaff, 7/?/?/????, 6600 ft., Hsu,Powell & Prentice, black light (1, EMEC); AZ, Coconino Co., West Fork 16 mi. SW of Flagstaff, 8/17/1964, 6500 ft., JG Franclemont, (1, CUIC); AZ, Coconino Co., Tuba City, 6/27/1967, Davidson & Cazier, at light (1, ASUT); AZ, Hackberry near Kingman, 8/8/1920, OC Poling, *Arenivaga erratica* det. Hebard 1932 (1, ANSP); AZ, Mohave Co., Boulder Springs near Kingman, 8/2/1920, OC Poling, (1, ANSP); AZ, Pima Co., Tucson, 6/15/1932, RA Flock, (1, NAUF); AZ, Pima Co., Tucson, 6/28/1932, RA Flock, (1, UAIC); AZ, Pima Co., Tucson, 6/28/1932, RA Flock, (1, NAUF); AZ, Yuma Co., Yuma, 9/18/1959, D Muse, at light (1, UAIC); AZ, Cochise Co., Tex Canyon Chiricahua Mts., 8/28/1927, ?700 ft., JA Kusche, (1, CAS); AZ, Poza Nuevo, Organ Pipe National Monument, 7/20/1981, P Bennett, at light (1, UAIC); AZ, Oak Creek Canyon, 7/22/1958, CW OBrien, lights (2, UAIC); AZ, Pima Co., Tucson, 6/25/1932, R.A.Flock, (1, UAIC); AZ, Pima Co., Tucson, 6/21/1932, R.A.Flock, (1, UAIC); AZ, Pima Co., Tucson, 6/18/1932, R.A.Flock, (1, UAIC); AZ, Pima Co., Tucson, 7/9/1932, R.A.Flock, (1, UAIC); AZ, Pima Co., Tucson, 7/14/1932, R.A.Flock, (1, UAIC); AZ, Pima Co., Tucson, 7/6/1932, R.A.Flock, (1, UAIC); AZ, Yuma Co., Yuma, ?/?/1899, H.Brown, H.S.Wallace No.968,Homoeogamia (Polyphaga) erratica A.N.C. (2, UMMZ,USNM); AZ, Sawmill Canyon, Hualapai Mts., 9/22/1919, O.C.Poling, *Arenivaga erratica* Rehn Det. Hebard 1922 (1, UMMZ); AZ, Welton, 7/9/1917, Wheeler, *Arenivaga erratica* Rehn Det. T.H.Hubbell 1932 (1, UMMZ); AZ, Pima Co., Sabino Canyon, Santa Catalina Mts., 6/6/1916, J.F.Tucker, *Arenivaga erratica* Rehn Det. T.H.Hubbell 1932 (1, UMMZ); AZ, Avondale Ranch, Agua Fria R., 8/7/1917, Wheeler, *Arenivaga erratica* Rehn Det. T.H.Hubbell 1928,1932 (2, UMMZ); AZ, Yuma Co., Yuma, 8/18/1930, Leonora K. Gloyd, 179, *Arenivaga erratica* Rehn Det. T.H.Hubbell 1931,1932 (2, UMMZ); AZ, Pima Co., Tucson, 7/7/1947, R.E.Elbe, Collection of H.S.Wallace (1, UMMZ); AZ, Maricopa Co., Phoenix, 9/10/1936, (1, UMMZ); AZ, Pima Co., Batamote Well, Ajo Valley (R&H), 9/16/1924, 1200’, (1, ANSP); AZ, Casa Grande NM, Coolidge, 6/15/1949, L.Arnberger, (1, LACM); AZ, Pima Co., Tucson, 8/6/1935, 2460’, JRTB, Mendenhall,Phoenix, (1, HEH); AZ, Madera Canyon, Santa Rita Mts., 8/16/1932, Kirkwood & Reid, (1, LACM); AZ, Benson, 8/7/1947, E.R.Tinkham, E.R.T., (1, HEH); AZ, Madera Canyon, Santa Rita Mts., 9/12/1953, E.R.Tinkham, (2, USNM); AZ, Sabino Basin, 9/19/????, C.H.T.Townsend, (1, USNM); AZ, Madera Canyon, Santa Rita Mts., 7/20/1948, J.F.Curry, Collected at light (1, CSCA); AZ, Madera Canyon, Santa Rita Mts., 8/20/1949, L.M.Martin, (2, LACM); AZ, Madera Canyon, Santa Rita Mts., 8/7/1947, L.M.Martin, (1, LACM); AZ, Madera Canyon, Santa Rita Mts., 8/15/1949, L.M.Martin, (1, LACM); AZ, Madera Canyon, Santa Rita Mts., 8/16/1947, L.M.Martin, (1, LACM); AZ, Madera Canyon, Santa Rita Mts., 7/12/1956, Martin, Comstock & Rees, (1, LACM); AZ, Molino Basin, Santa Catalina Mts., 8/29/1951, C.D.McNeill, (4, EMEC); AZ, 10 mi. E of Diablo Canyon, 7/16/1947, E.R.Tinkham, E.R.T., (1, HEH); AZ, Pima Co., Saguaro NM, 6/6/1966, Gordon & Brach, (1, LACM); AZ, Pinal Co., Hidden Valley,5 mi. SW Maricopa, 11/3-5/1958, K.Roever, (5, LACM); AZ, Pima Co., Ajo, 8/16/1952, C.& P.Vaurie, (1, AMNH); AZ, Pima Co., Organ Pipe NM, 8/6/1955, Werner & Butler, (2, UAIC); AZ, Pima Co., Organ Pipe NM, 6/14/1952, Cazier, Gertsch & Schrammel, (10, AMNH); AZ, Pima Co., Quitobaquito, Organ Pipe NM, 6/13/1952, Cazier, Gertsch & Schrammel, (3, AMNH); AZ, Yuma Co., Yuma, 8/6/1948, C.& P.Vaurie, (1, AMNH); AZ, 36 mi. E of Gila Bend, 7/21/1955, G.D.Butler, at light (4, UAIC); AZ, Maricopa Co., Gila Bend, 7/22/1948, C.& P.Vaurie, (5, AMNH); AZ, Pima Co., Sabino Canyon, west slope station, Catalina Mts., 7/26/1948, 2500’, W.Nutting, Sycamore-oak-mesquite, W.L.N., drawing of genitalia (1, UAIC); AZ, Santa Rita Mts., 7/12/1950, J.Arnold, (1, SEMC); AZ, Pima Co., Sabino Canyon, 7/9/1952, E.H.&L.D.Beamer,LaBerge,Wolf, Liang&Winer, (2, SEMC); AZ, Pima Co., Santa Catalina Mts., 6/26/1933, Bryant, Lot 272 (1, UAIC); AZ, Pima Co., S. of mouth of Sabino Canyon, Santa Catalina Mts., 9/2/1950, 2600’, Cohn,Boone&Cazier, (1, AMNH); AZ, Pima Co., Tucson, 7/5/1954, Cazier&Gertsch, (2, AMNH); AZ, Pima Co., Tucson, 6/27/1954, M.Cazier, (1, AMNH); AZ, Pima Co., Tucson, 6/30/1949, 2200’, G.M.Brandt, (1, AMNH); AZ, Pima Co., Tucson, 7/18/1953, G.M.Brandt, (1, AMNH); AZ, Yuma Co., Laguna Dam, Yuma, 8/10/1948, 1000’, Nutting & Werner, at light, willow area, W.L.N., drawing of genitalia (1, UAIC); AZ, Yuma Co., Hope, 8/12/1948, 1400’, Nutting & Werner, at light, greenwood desert, W.L.N., *Arenivaga erratica* Rehn det.W.Nutting 1950 (1, UAIC); AZ, Yuma Co., Hope, 8/12/1948, 1400’, Nutting & Werner, at light, greenwood desert, W.L.N., drawing of genitalia (1, UAIC); AZ, Pima Co., Tucson, (1, AMNH); AZ, Madera Canyon, Santa Rita Mts., 8/8/1947, L.Martin, (1, LACM); AZ, Pima Co., Tucson, 9/18/1937, E.D.Ball, (1, UAIC); AZ, Pima Co., Tucson, 9/?/1929, S.B.Tatum, (1, UAIC); AZ, Madera Canyon, Santa Rita Mts., 8/18/1949, L.Martin, (1, LACM); AZ, Pima Co., Tucson, 9/?/1929, W.P.Stockwell, (2, UAIC); AZ, Yuma Co., Yuma, 5/27/1952, G.Butlwe, (1, UAIC); AZ, Pima Co., Tucson, 9/30/1938, Bryant, (1, UAIC); AZ, Pima Co., Tucson, 9/30/1939, Bryant, (1, UAIC); AZ, Pima Co., Tucson, 10/10/1939, Bryant, (2, UAIC); AZ, Pima Co., Tucson, 10/2-25/1916, Ac.1920 (1, AMNH); AZ, Pima Co., Tucson, 9/29/1940, E.L.Peterson, (2, UAIC); AZ, Pima Co., Tucson, 10/?/1929, C.Dierking, (2, UAIC); AZ, Pima Co., Tucson, 10/15/1927, L.C.Bailey, (3, UAIC); AZ, Pima Co., Tucson, 9/18/1935, M.Hattis, (1, UAIC); AZ, Pima Co., Tucson, 10/10/1927, T.Knight, (1, UAIC); AZ, Pima Co., Tucson, 9/?/1929, J.S.Thornber, (1, UAIC); AZ, Pima Co., Tucson, 7/?/1929, at light (1, UAIC); AZ, Pima Co., Tucson, 10/2/1923, (1, UAIC); AZ, Pima Co., Tucson, 10/4/1923, (1, UAIC); AZ, Pima Co., Tucson,1130 E.Helen St., 10/8/1937, L.P.Wehrle, Lot 102,Sublot 416, *Arenivaga erratica* (Rehn) Det.E.R.Tinkham 1938 (1, HEH); AZ, Pima Co., Tucson, 11/11/1939, J.Sprecher, (1, UAIC); AZ, Pima Co., Tucson, 10/7/1939, D.Foote, (1, UAIC); AZ, Pima Co., Tucson, 9/25/1943, M.H.Frost,Jr., *Arenivaga erratica* (Rehn) (1, UAIC); AZ, Pima Co., Tucson, 10/15/1942, L.Middleton, *Arenivaga erratica* (Rehn) MFJr. (1, UAIC); AZ, Pima Co., Tucson, 10/14/1935, (1, UAIC); AZ, Pima Co., Tucson, 10/10/1923, (1, UAIC); AZ, Wickenburg, 7/5/1950, H.O.Wright, (1, SEMC); AZ, Pima Co., Tucson, 7/2-8/1932, R.A.Flock, (5, UAIC); AZ, Pima Co., Tucson, 6/12-27/1932, R.A.Flock, (13, UAIC); AZ, Pima Co., Tucson, 5/20/1932, R.A.Flock, (1, UAIC); AZ, Pinal Co., Maricopa, 10/17/1927, J.A.Kusche, (2, CAS); AZ, 10 mi. W. of Casa Grande, 8/31/1942, E.O.VanDyke, (1, CAS); AZ, Hope, 7/19/1946, E.O.VanDyke, (1, CAS); AZ, Tuba City, 8/1/1937, R.P.Allen, (1, CAS); AZ, Tuba City, 7/18/1937, R.P.Allen, (1, CAS); AZ, Tuba City, ?/9/1937, R.P.Allen, (1, CAS); AZ, Coconino Co., Rainbow Lodge, Navajo Mt., 7/14/1933, 6500’, H.N.Hultgren, Ansel F. Hail Expedition 1933 (1, CAS); AZ, Pima Co., Alamo Canyon, Ajo Mt., 7/?/1923, Leech & Green, (1, CAS); AZ, Pima Co., Organ Pipe NM, Campground, 8/25/1979, C.Melton, (1, UCMC); AZ, Pima Co., Tucson, KOA Campground 20 mi. N on I10, 9/5/1980, S,W.Nichols, at night on concrete wall, *Arenivaga erratica* Rehn Det.F.W.Fisk 1980 (1, CUIC); AZ, Pima Co., Organ Pipe Cactus NM, 6/4/1956, A.Menke, (1, LACM); AZ, Madera Canyon, Santa Rita Mts., 9/11/1951, L.Martin, (1, LACM); AZ, Hope, 17002, 33.721149, -113.694339, E.O.VanDyke, (1, CAS); AZ, Florence, 7/28/1917, Wheeler, (1, USNM); AZ, Pima Co., Organ Pipe Cactus NM, 4/16/1947, E.R.Tinkham, E.R.T., (1, HEH); AZ, C.V.Riley, (1, USNM); AZ, Pima Co., Sabino Canyon, 8/6/1959, K.V.Krombein, (1, USNM); AZ, Pima Co., Tucson, 7/28/1954, R.S.Beal, (2, EMEC); AZ, Pima Co., Tucson, 9/1/1947, R.S.Beal, *Arenivaga erratica* Rehn det. H.F.Strohecker (1, EMEC); AZ, Pleasant Lake, 7/7/1952, R.H.&L.D.Beamer, LaBerge & Liang, (1, SEMC); AZ, Santa Cruz Co., Madera Canyon, 8/27/1977, G.Forbes, (1, SDMC); AZ, Santa Rita Mts., 7/10-12/1950, J.Arnold, (2, SEMC); AZ, Santa Rita Mts., 7/5/1950, J.G.Rosen, (2, SEMC); AZ, Santa Rita Mts., 7/12/1950, R.H.&L.D.Beamer, (3, SEMC); AZ, Santa Cruz Co., Madera Canyon, 7/31/1992, Faulkner & Gillen, (1, SDMC); AZ, Hayden, 8/?/1940, (1, UAIC); AZ, Santa Cruz Co., Madera Canyon, 8/9-20/1978, Brown & Faulkner, (1, SDMC); AZ, Madera Canyon, Santa Rita Mts., 9/12/1951, L.Martin, (1, LACM); AZ, Madera Canyon, Santa Rita Mts., 8/24/1951, L.Martin, (1, LACM); AZ, Maricopa Co., 19 mi. NE Mesa, 7/31/1960, Wood, Warren & Shurtleff, light (1, SEMC); AZ, Maricopa Co., Gillespie Dam, Gila Bend, 8/9/1948, 1000’, Nutting & Werner, tamerix-willow in desert, at light, W.L.N. (5, UAIC); AZ, Madera Canyon, Santa Rita Mts., 8/17-18/1949, L.Martin, (1, LACM); AZ, Madera Canyon, Santa Rita Mts., 8/8/1947, L.Martin, (1, LACM); AZ, W.Gila Valley, 7/30/1957, V.Roth, (1, UAIC); AZ, Topock, 10/9/1917, O.C.Poling, (1, ANSP); AZ, Yuma (now La Paz) Co., 0.5 mi. S of Parker Dam, 8/10/1983, R.E.Wagner, at black light (1, UCRC); AZ, Yuma (now La Paz) Co., Colorado River at Parker, 8/15/1963, Tauber & Toschi, (1, EMEC); AZ, Sawmill Canyon, Hualapai Mts., 9/22/1919, OC Poling, (1, ANSP); TX, Carrizo Springs, A.Wadgymar, PARATYPE; 1 specimen genitalia figured H1920 (5, ANSP); TX, Weslaco, 11/1/1940, P.T.Riherd, light trap (12, TAMU); TX, Eastland Co., 5/3/1921, G.O.Wiley, (1, ANSP); TX, Kenedy Co., Kenedy Ranch, Jaboncillos Pasture, sand dunes, 4/20-22/2001, 26.58.38N 97.40.59W, Godwin & Riley, malaise trap (4, TAMU); TX, Round Mt., (3, ANSP); TX, Jim Wells Co., La Copita Res. Sta. 8 mi. W of Ben Bolt, 5/20/1987, J.C.Schaffner, at light (2, TAMU); TX, Sabinal, 5/1/1910, F.C.Platt, (1, ANSP); TX, San Antonio, 9/18/1927, Palmer, (1, ANSP); TX, Kerrville, 4/11/1907, F.C.Platt, at light (2, ANSP); TX, Mission, 7/5/1939, R.I.Sailer, (1, ANSP); TX, Goliad, Jul.1928, W.A.Cushman, (1, ANSP); TX, Brownsville, 4/30/1895, C.H.T.Townsend, (1, ANSP); TX, Brownsville, May.1922, (2, ANSP); TX, Hays Co., Aug.1936, E.P.C., (1, ANSP); TX, Kingsville, C.T.Reed, Cornell Univ. Lot 912 Sub 811 (1, CUIC); TX, Burleson Co., Old River Ranch ca. 3 mi. E of Clay, 10/12/1998, W.Godwin, (2, TAMU); TX, Brown Co., Lake Brownwood SP, 4/29/1995, E.G.Riley-80, UV (2, TAMU); TX, Georgetown, 1 specimen-S.H.Scudder, Palmer-cave (2, ANSP); TX, vicinity of Kingsville, C.Reed, (1, ANSP); TX, Bexar Co., 2/5/1929, (2, ANSP); TX, Uvalde Co., 5/19/1918, J.C.Bradley, (1, ANSP); TX, Brownsville, 6/1/1924, J.N.Knull, (1, OSUC); TX, Pharr, 4/27/1948, R.P.Dow, (1, FSCA); TX, Dimmit Co., Winterhaven, S.E.Jones, (3, ANSP); TX, Hidalgo Co., Tex.Exp.Sta., 6/14/1931, light trap, 485 (2, ANSP); TX, Dimmit Co., Tex.Exp.Sta., 6/7/1933, H.J.Reinhard, light trap (2, ANSP); TX, Kenedy Co., Kenedy Ranch, Jaboncillos Pasture, sand dunes, 4/21/2001, 27.01.294N 97.43.114W, W.Godwin, UV light (1, AMU); TX, Kenedy Co., Kenedy Ranch, Jaboncillos Pasture, sand dunes, 4/21/2001, 26.59.22N 97.40.11W, Raber,Riley & Yoder, UV light (1, TAMU); TX, Bexar Co., 2/5/1929, H.B.Parks, (4, TAMU); TX, Corpus Christi, 8/24/1969, C.W.Griffin, Suntide Refining, at light (1, USNM); TX, Nueces Co., Clare (should be Hazel) Bazemore Park, 4/10/1970, C.W.Griffin, UV light (1, USNM); TX, Kenedy Co., Kenedy Ranch, Jaboncillos Pasture, sand dunes, 4/21/2001, 27.01.293N 97.43.114W, Gillogly & Schaffner, MV light (1, TAMU); TX, Kleberg Co., Riviera, 7/25/1961, H.R.Burke, (1, TAMU); TX, San Patricio Co., Corpus Christi SP, 5/20-21/1981, Doyen & Liebherr, (1, EMEC); TX, Dimmit Co., Tex.Exp.Sta., 10/21/1933, S.E.Jones, (1, TAMU); TX, Bexar Co., Mt.View Acres Ebony Hill Res. Station, 3/21/1972, Kendall & Kendall, (2, FSCA); TX, Comal Co., Bulverde, 4/15-16/1996, Warner & Wappes, Bill Warner, UVBL, (1, WB Warner); TX, Atascosa Co., 14 mi. S of Lyttle, 5/26/1994, Godwin & Gibson, BL (1, TAMU); TX, Uvalde Co., 4/1/1994, J.W.Stewart, at light (1, TAMU); TX, Carrizo Springs, June 1885, A.Wadgymar, (1, CAS); TX, Starr Co., 3 mi. E of Falcon Hts., 4/26/1991, E.G.Riley, (1, TAMU); TX, Dimmit Co., Tex.Exp.Sta., 6/7/1933, H.J.Reinhard, light trap (1, CUIC); TX, Weslaco, 11/21/1940, Riherd, light trap (4, TAMU); TX, Edinburg, 10/28/1971, P.T.Riherd, (1, TAMU); TX, Kerrville, 5/25/1984, W.F.Chamberlain, at light (2, TAMU); TX, Kerrville, 6/16/1997, W.F.Chamberlain, at light (1, TAMU); TX, Burnet Co., Inks Lake SP, 4/19/1980, C.W.Agnew, (2, TAMU); TX, Nueces Co., Corpus Christi, 4/10/1970, C.W.Griffin, (2, TAMU); TX, Padre Island, 7/1/1965, Dr.Lenczy, (1, LACM); TX, Uvalde Co., Dec. 1920, Bridwell, (1, USNM); TX, Austin, May.1942, E.J.Gerberg, (1, USNM); TX, Austin, Aug.1942, E.J.Gerberg, (2, USNM); TX, Cameron Co., 7/24/1955, S.W.Bromley, (1, USNM); TX, Hemphill Co., Canadian (should be Gene Howe) WMA, 7/11-12/1974, E.L.Todd, (1, USNM); TX, New Braunfels, 7/26/1942, E.S.Ross, (1, USNM); TX, Kinney Co., Brackettville, 6/20-25/1988, S.A.Stockwell, black light (2, EMEC); TX, Brownsville, 8/6/1937, C.S.Rude, (1, TAMU); TX, San Patricio Co., Welder (should be Welder Flats) WMA, 7/16/1989, J.Schaffner, (1, TAMU); TX, Karnes Co., 7/23/1928, R.H.Beamer, (1, ANSP); TX, Pecos Co., Jul.-Aug. 1966, C.W.Neeb, light trap (1, TAMU); TX, Shovel Mount, 9/10/1901, F.G.Schaupp, (1, ANSP); TX, Brownsville, June, F.H.Snow, (1, ANSP); TX, Carrizo Springs, F.G.Schaupp, (1, MCZ); TX, Brownsville, 3/11/?, G.Dorner, (1, ANSP); TX, Starr Co., 3 mi. E of Falcon Mts., 7/28/1991, Carlow & Riley, at UV light (1, TAMU); TX, San Antonio, 7/10/1939, J Vich, (1, CUIC); TX, Camp Bullis in tent, 3/16/1969, Spencer, (1, SDMC); TX, Schaupp, (2, MCZ); TX, Duval Co., 6.3 mi. W of San Diego, 4/4/1970, C.W.Griffin, Sepulveda Ranch, collected at black light (1, USNM); TX, Duval Co., Saenz Ranch, Benavides on FM2295, 5/20/1993, Goodwin & Abbott, UV light (1, TAMU); TX, Bexar Co., San Antonio, 3/20/1982, R.R.Thomas, (1, TAMU); TX, Wichita Co., 5/24/1975, L.Burt, Grass (1, UCRC); TX, Hall Co., 5/27/1968, D.D.Collins, (2, USNM); TX, Hall Co., 6 mi. SE of Turkey, 5/6/1970, Brien & Huddleston, Temik Project, Pitfall trap (1, USNM); TX, Waco, [tiny illegible label] (1, MCZ); TX, Starr Co., 7/5/1938, R.H.Beamer, (1, ANSP); TX, Jim Wells Co., La Copita Res. Sta. 8 mi. W of Ben Bolt, 5/20/1987, J.C.Schaffer, taken at light (1, TAMU); TX, Starr Co., 10 mi. N of Sullivan City, 4/13/1994, R.L.Aalbu, on road at night (1, CSCA); TX, Austin, 10/?/1899, (4, UMMZ); TX, Travis Co., ?/?/1931, S.U.G.Silvey, (1, UMMZ); TX, Brownsville, C.H.T.Townsend, (1, USNM); TX, Esperanza Ranch, Brownsville, 8/29/????, Brooklyn Museum Colln. 1929 (1, USNM); TX, Kerr Co., Kerrville, 4/11/1907, F.C.Pratt, at light (3, USNM); TX, Brownsville, 4/30/1895, C.H.T.Townsend, (2, USNM); TX, Brownsville, 4/14/1925, (1, USNM); TX, Esperanza Ranch, Brownsville, 8/?/1922, Catal.No.31,Brooklyn Museum Colln.1929,Homoeogamia erratica Rehn A.N.C.,BI (2, USNM); TX (1, USNM); TX, Belfrage, C.V.Riley, (1, USNM); TX, Sabino, 5/?/1910, F.C.Pratt, (1, USNM); TX, San Antonio, 9/18/1927, Palmer, 1215.S.H.Scudder Coll. (4, USNM); TX, Eagle Pass, 3/30/1908, Jones & Pratt, at light, (1, HEH); TX, Roanoke, 2/3/1932, F.C.Bishopp, Bishopp No. 18617 (3, USNM); TX, Ballinger, 10/5/1911, H.Pinkus, at light (1, USNM); TX, Carrizo Springs, 8/28/1885, Dr.A.Wadgymar, Collection C.V.Riley (1, USNM); TX, Belfrage, (2, USNM); TX, Bexar Co., Ft. Sam Houston, 11/4/1953, B.J.Adelson, electric light (1, EMEC); TX, Bexar Co., Ft. Sam Houston, 6/18/1952, B.J.Adelson, (1, EMEC); TX, Mills Co., 11 mi. SE of Goldthwaite, 7/12/1955, 1300-1400’, T.J.Cohn, (1, USNM); TX, near Casa Blanca Lake, Corpus Christi, 12/17/1938, L.Berner, (2, UMMZ); TX, Menard, 10/1/1946, L.J.Bottimer, photo spec. (1, USNM); TX, San Antonio, 4/1/1935, E.V.Walter, T#6378 (1, USNM); TX, San Antonio, 11/13/1934, L.Seaton, T#6377 (1, USNM); TX, Cotulla, 5/12/1906, Crawford & Pratt, at light (1, USNM); TX, Kenedy Co., Armstrong, 6/13/1962, P.A.Glick, (1, CSCA); TX, Kenedy Co., Armstrong, 3/31/1962, H.Glick, (2, CSCA); TX, Randall Co., Palo Duro Canyon SP, 5/12/1961, L.M.Martin, Reid, Rees & Ford (2, LACM); TX, Jeff Davis Co., Fort Davis, 10/13/1953, R.H.Reid, (1, LACM); TX, Kleberg Co., 3.5 mi. N of Riviera, 6/29/1961, R.L.Westcott, (1, LACM); TX, San Patricio Co., Lake Corpus Christi SP4 mi. SW of Mathis, 7/30/1955, 100’, T.J.Cohn, (1, USNM); TX, Lubbock Co., Lubbock, 9/27/1968, Rutledge, (2, USNM); TX, Lubbock Co., 9 mi. E of Lubbock, 10/1/1970, G.R.Graves, (2, USNM); TX, Lubbock Co., 2 mi. N of Lubbock, 9/10/1970, G.R.Graves, (1, USNM); TX, Lubbock Co., Lubbock, 9/30/1970, S.D.Kemper, (1, USNM); TX, Goliad Co., 7/?/1928, R.A.Cushman, (1, USNM); TX, Lubbock Co., 5/6/1967, J.Hatfield, (1, USNM); TX, Lubbock Co., 9/29/????, J.England, (1, USNM); TX, Lubbock Co., Lubbock, 10/10/1968, J.Stroebele, (1, USNM); TX, Lubbock Co., Lubbock, 10/3/1968, C.Vars, (1, USNM); TX, Lubbock Co., Lubbock, 4/11/1957, A.Brown, (1, USNM); TX, Lubbect (?), 10/12/1968, R.Fulkerson, (1, USNM); TX, Lubbock Co., Lubbock, 9/12/1970, G.W.Brothers, (1, USNM); TX, Justiceberg, 10/13/1968, Ward & Huddleston, white and UV light (1, USNM); TX, San Antonio, 3/23/1927, W.Ewing, on ground under stone (1, USNM); TX, Laguna Madre,25 mi. SE of Harlingen, 5/30/1948, Hardy & Woolley, nest of Nectoma micropus,46-10489 (1, USNM); TX, Hamilton Co., 11/24/1968, R.A.Padney, (1, USNM); TX, Jim Wells Co., 7/29/1969, J.Snelgrove, (1, USNM); TX, Jim Wells Co., 7/22/1969, J.Snelgrove, (1, USNM); TX, Weslaco, 6/26/1931, *Arenivaga apacha* Rehn Heb.,4551 (1, TAMU); TX, Webb Co., Laredo, 5/20-24/1948, Nutting & Werner, mesquite area, drawing of genitalia, W.L.N. (1, UAIC); TX, Brownswood (should be Brownwood), 7/8/1919, Acc.23972 (1, AMNH); TX, Austin, 4/28/1950, H.T.Spieth, (2, AMNH); TX, Hidalgo Co., Edinburg, 4/?/1938, S.Mulaik, (1, UMMZ); TX, Taylor Co., Camp Barkley (should be Barkeley) near Abilene, 10/10/1943, C.L.Remington, (1, PMNH); TX, Uvalde Co., Tampke Ranch Cave,5 mi. S of Utopia, 2/11/1966, Reddell & McKenzie, at twilight in main passage (1, USNM); CA, Borrego Springs, Pegleg Canyon, 11/10/1957, at dusk, (1, HEH); CA, Mt.Springs, 7/25/1938, Jean Russell, (1, SEMC); CA, Imperial Co., Ocotillo, 7/17/1988, B.Morris, (1, SDMC); CA, Imperial Co., Palo Verde Valley, 7/28/1959, K.L.Japport, (1, CSCA); CA, Imperial Co., Bard, 6/29/1959, Salazar, Argon light trap (2, CSCA); CA, Imperial Co., Bard, 7/11/1966, Ratcliff, Argon light trap (6, CSCA); CA, Imperial Co., Bard, 8/26/1959, Kilgore, Cotton (1, CSCA); CA, Imperial Co., Bard, 6/20/1961, Harrison, Argon light trap (1, CSCA); CA, Imperial Co., Bard, 9/24/1959, Kilgore, light trap (4, CSCA); CA, Imperial Co., Winterhaven, 10/20/1959, H.Blakemore, light trap (6, CSCA); CA, Temecula, 9/11/1930, (1, LACM); CA, Temecula, 9/9/1930, (1, LACM); CA, Riverside Co., Ripley, 8/16/1946, P.D.Hurd, (1, EMEC); CA, Imperial Co., Bard, 10/26-27/1959, H.Blakemore, Argon light trap (13, CSCA); CA, Imperial Co., Bard, 11/12/1959, H.Blakemore, Argon light trap (1, CSCA); CA, Imperial Co., Bard, 10/26/1959, Argon light trap (1, CSCA); CA, Imperial Co., Bard, 11/12/1959, Colby & Balion, Argon light trap (1, CSCA); CA, Imperial Co., Haughtelin, Lake, Bard, 7/21-22/1953, (1, USNM); CA, Imperial Co., Ft. Yuma Sta., 5/18/1952, on an inspection table, CA Dept.. Agr.No.52F4 (1, CSCA); CA, Imperial Co., Imperial Valley near Wister, T9S R13E Sec.35, 10/27/1990, ~75’, JP & KES Donahue, (1, LACM); CA, Imperial Co., 2 mi. NW of Glamis, 11/1/1974, Doyen & Powell, in pitfalls (1, EMEC); CA, Riverside Co., Blythe, 7/22/1963, (7, CSCA); CA, Riverside Co., Blythe, 6/21/1961, Maxwell, Argon light trap (1, CSCA); CA, Riverside Co., Blythe, 9/15/1960, K.L.Japport, (2, CSCA); CA, Riverside Co., Blythe, 6/23-25/1961, W.E.Gunderson, Argon light trap (2, CSCA); CA, Riverside Co., Blythe, 7/26/1946, Hurd & Barr, 17NW (1, EMEC); CA, Riverside Co., Blythe, 6/15/1959, E.W.Magoon, (1, FSCA); CA, Imperial Co., Imperial Dam, 6/28/1954, W.McDonald, (3, LACM); CA, Riverside Co., Deep Canyon, 11/12/1969, O.C. & J. Wheeler, *Arenivaga* Det. Saul I. Frommer (2, UAIC); CA, San Bernardino Co., 10 mi. NE of Earp, 4/17/1964, R.L.Langston, blacklight trap (1, EMEC); CA, San Bernardino Co., 10 mi. NE of Earp, 4/17/1964, D.D.Linsdale, (1, EMEC); CA, Riverside Co., Blythe, 7/8/1956, J.I.Stage, (1, EMEC); CA, San Diego Co., Pamo Guard Station, Ramona, 8/23/1949, (1, SDMC); CA, San Diego Co., Borego, 6/19/1956, (1, SDMC); CA, Port El Ysidro, 8/22/1931, E.R.Tinkham, (1, ANSP); CA, San Diego Co., San Felipe Valley, 8/30/1946, C.Henne, (3, LACM); CA, San Diego Co., Lakeside U-Totum, 8/22/1978, Faulkner & Brown, (1, SDMC); CO, Montezuma Co., Battle Rock 13 mi. W of Cortez, 9/3/1989, 1650 ft., Speiler,Weissmann & Penhall, at light (1, UCMC); CO, Mesa Co., COL.Mon.night (Colorado National Monument), 7/28/1962, Lanham et al., (1, UCMC); CO, Montezuma Co., Durango, 8/?/1900, FJ Olsen, *Arenivaga erratica* det. Hebard 1916 (1, USNM); OK, Jackson Co., Jct. Hwy. 6 & Red River, 6/17/1995, E.G.Riley, 141,UV (1, TAMU); OK, Comanche Co., Wichita NF, 6/10/1926, T.H.Hubbell, 17,small square blue label (1, UMMZ); OK, Murray Co., 4/22/1933, R.D.Bird, (1, ANSP); OK, Grandfield, 7/5/1937, Standish-Kaiser, (1, ANSP); OK, Cheyenne, 6/7/1937, Standish-Kaiser, (1, ANSP); OK, Lugert, 7/7/1937, Standish-Kaiser, (1, ANSP); NV, Lincoln Co., 15.7 mi. N of Jct. Hwy.168 on Meadow Valley Rd., 8/13/2005, 36.53.9N 114.39.8?, W.B.Warner, Meadow Valley Wash riparian zone (2, WB Warner); UT, Washington Co., Red Cliffs Rec, Area, !4 mi. SW Leeds, 7/11/1982, 3200’, J.P. & K.E Donahue, (1, LACM); UT, Washington Co., 10905, C.C.Searl, (1, SDMC); UT, Arches NM, 8/1/1950, DM Allred, (1, SDMC); CA, Riverside Co., Thermal, 9/28/1960, LD Moore, (3, MLBM); CA, 4 mi S El Cajon, 7/21/1967, T Ashley 67-26, (1, FSCA); CA, San Bernardino Co., Needles, 9/20/2005, 34.52N, 114.38W, 620 ft., SM Clark & RC Mower, *Arenivaga apacha* (Saussure) det. AH Barnum 2010 (7, MLBM); UT, Washington Co., Virgin R, 9/3/1993, (3, MLBM); UT, Washington Co., 3 mi E Gunlock on Santa Clara R, 8/19/1967, Barnum & Moore, (1, MLBM); UT, Washington Co., 5 mi S Hurricane, 7/12/1978, GH Nelson, ultaviolet light (1, FSCA); NV, Lincoln Co., Beaver Dam SP, 8/11/1971, GM Nishida & DF Zoller, light trap, *Arenivaga erratica* Rehn det. RC Bechtel ‘71 (1, NVDA); NV, Clark Co., 5 mi NW Moapa, 7/21/1962, RC Bechtel & FD Parker, light trap, 1 specimen-*Arenivaga erratica* Rehn det. HF Strohecker (3, NVDA); NV, Clark Co., St. Thomas Gap, 6/13/1984, RC Bechtel & JP Young, black light trap, T17S R71E S31 (1, NVDA); NV, Clark Co., Warm Springs, 8/1/1996, RW Baumann, *Arenivaga erratica* (Rehn) det. AH Barnum 2010 (1, MLBM); NV, Clark Co., Warm Springs LDS Rec. Area, 8/18/1997, 36.43.22N 114.43.00W, Baumann & Huillet, *Arenivaga erratica* (Rehn) det. AH Barnum 3/1999 (1, MLBM); NV, Clark Co., Warm Springs, 7/19/1990, RW Baumann, *Arenivaga erratica* (Rehn) det. AH Barnum 2010 (5, MLBM); NV, Mercury NTS, 7/25/1960, MLBM-AEC code 1BF25e (1, MLBM); OK, Woodward Co., Woodward, 9/14/1966, DC Arnold, in building (1, OSEC); OK, Stephens Co., Comanche, 5/7/1974, DC Arnold, at light (1, OSEC); OK, Lugert, 7/7/1937, Standish-Kaiser, (1, OSEC); OK, Beckham Co., Sandy Sanders WMA, 5/?/2010, LJ Vitt & JP Caldwell, Catalog No. OMNH-20889, *Arenivaga* sp. det. K. Menard 2012 (1, OMNH); AZ, Graham Co., Pinaleno Mt. Hwy. 366 milepost 120.6, 6/10/2012, 32 40 10.2 109 47 20.0, 5100 ft., DB Weissman, oaks and shrubs (1, HEH); AZ, Kofa Mtns., 7/2/1969, W. Rosenberg Collection (1, FSAC); AZ, Pima Co., Organ Pipe Cactus NM, 4 mi N Lukeville, 7/21/1974, JB Heppert, at (UV) blacklight (1, FSAC); AZ, Tucson Mts., Gilbert Ray Campground, 7/27/1975, GH Nelson, ultaviolet light (1, FSAC); AZ, Tucson Mts., Gilbert Ray Campground, 7/29/1975, GH Nelson, ultaviolet light (1, FSAC); AZ, Maricopa Co., 10 mi N Scottsdale, 7/?/1962, T. Blaine Moore, *Arenivaga erratica* (Rehn) Det. AH Barnum 2005 (1, MLBM); AZ, Pima Co., Organ Pipe Cactus NM, 6/29/1962, T. Blaine Moore, *Arenivaga erratica* (Rehn) Det. AH Barnum 2005 (3, MLBM); AZ, Pima Co., Organ Pipe Cactus NM Campground, 8/7/1970, T. Blaine Moore, Night Lt., *Arenivaga erratica* (Rehn) Det. AH Barnum 2005 (2, MLBM); AZ, Pima Co., Organ Pipe Cactus NM Campground, 8/7/1970, Andrew H. Barnum, Night Lt. (2, MLBM); AZ, Wickenburg, 8/15/1957, JC Schaffner, (1, MLBM); AZ, Maricopa Co., 19 mi NE Mesa, 7/31/1960, SL Wood, JB Karren, H Shurtleff, at light, *Arenivaga erratica* (Rehn) Det. AH Barnum 2010 (3, MLBM); AZ, Pima Co., Colossal Cave, 8/25/1970, RE Woodruff, blacklight trap (1, FSAC); AZ, Pima Co., nr. Sabino Canyon, 7/21/1958, RH Arnett, Jr., at light 342 (1, FSAC); AZ, Pima Co., nr. Sabino Canyon, 6/30/1959, RH Arnett, Jr., Lot No. 385 (5, FSAC); AZ, Maricopa Co., Painted Rock Petroglyphs, 9/18/2011, 33 01 23.1N 113 02 51.8W, 570’, DB Weissman, (2, HEH); TX, San Antonio, 4/2/1940, KC Emerson, (1, OSEC); TX, Dimmit Co., Chaparral WMA, 6/7-8/1992, AW Hook, (8, MLBM); TX, Zapata Co., Falcon, 5/5/1999, SM Clark, (1, MLBM); TX, Starr Co., Falcon Heights, 10/9/1993, SM Clark, (1, MLBM); TX, Travis Co., Austin, Bull Creek, Spicewood Springs Rd., 4/24/1991, CR Nelson, #5661, Photograph voucher for C. Riley Nelson (1, MLBM); TX, 6-7/?/1962, G Nichols, (1, FSAC); UT, St. George, 9/9/1954, (Goodarzy) (GF Knowlton), (1, ANSP); NV, Esmeralda Co., Clayton Valley 2 mi S of Silver Peak, 8/22/1924, 4350 ft., (R&H), (1, ANSP). MEXICO: Sonora, Desemboque,5 km village in arroyo near well, 8/8-10/1953, B Malkin, (3, CAS); Sonora, 18 mi. E of El Puerto, 8/7/1960, Arnaud Jr.,Ross & Rentz, (3, CAS); Tamaulipas, San Fernando, 8/26/1954, 700 ft., (2, SEMC); Sonora, *Arenivaga erratica* det. Rehn (1, ANSP); Sonora, Poza Coyote, 7/5/1952, P & C Vaurie, (1, AMNH); Sonora, Rocky Point, 10/5/1953, MA Cazier, (1, AMNH); Sonora, Desemboque, 8/1-15/1953, B Malkin, (1, CAS); Sonora, Hermosillo, 7/9-16/1953, B Malkin, (1, CAS); Coahuila, 5 mi. S of Hermanas, 8/1/1959, 1350 ft., T Cohn, #129 (1, UMMZ); Tamaulipas, Nuevo Laredo, 5-6/?/1930, A Dampf, (1, ANSP); Tamaulipas, 8 mi. N of Jimenez, 6/15/1953, UK Mex. Expedition (1, SEMC); Tamaulipas, San Fernando, 8/27/1954, 700 ft., CD Michener & party, (1, SEMC); Tamaulipas, Abasolo, 5/17/1952, Cazier,Gertsch & Schrammel, (1, AMNH); Monterey, 7/12/1991, WF Chamberlain, at light (2, TAMU); BC, 2 mi S of Tijuana, 8/21/1931, ER Tinkham, (1, ANSP); Sonora, 10 mi. E of Sonoita on Hwy. 2, 9/24/1967, C Cushner, (3, EMEC); BC, ? Tanks, 11/15/1936, CF Harbison, (1, SDMC); Tamaulipas, 8 mi. E of Padilla Rancho Sta. Ana, 12/21/1941, Cantrell & Friauf, #27 (2, UMMZ); Sonora, 20 mi. S of Sonoita, 9/22/1867, CF Harbison, (1, SDMC); Sonora, 8 km W of Carbo, 10/5/1960, WW Gibson, at light (1, UAIC). Determiner label *Arenivaga tonkawa* Hopkins 2011” [white label with black border].

##### Distribution.

This species has a disjunct distribution comprising Texas, western Oklahoma and northeastern Mexico in one part, and Arizona, far southern California, southern Nevada, southern Utah, western Colorado and northwestern Mexico in the other part. See Fig. 149.

##### Diagnosis.

*Arenivaga tonkawa* varies widely phenotypically but may be diagnosed by always having two adjacent spines on the posterior end of the medial margin of the right dorsal phallomere combined with a small central sclerite with folded over anterior edge. See Fig. 148.

##### Description.

**Male.** *Measurements*. Holotype stand-in TL = 20.2 mm, GW = 8.9 mm, PW = 5.92 mm, PL = 4.28 mm, TL/GW = 2.27, PL/PW = 0.72. EW = 0.15 mm; OW = 0.40 mm. Among paratypes range of TL 15.7–25.5 mm; range of GW 7.3–12.5 mm; range of PW 5.02–8.55 mm; range of PL 3.83–5.25 mm.

**Figure 147.**
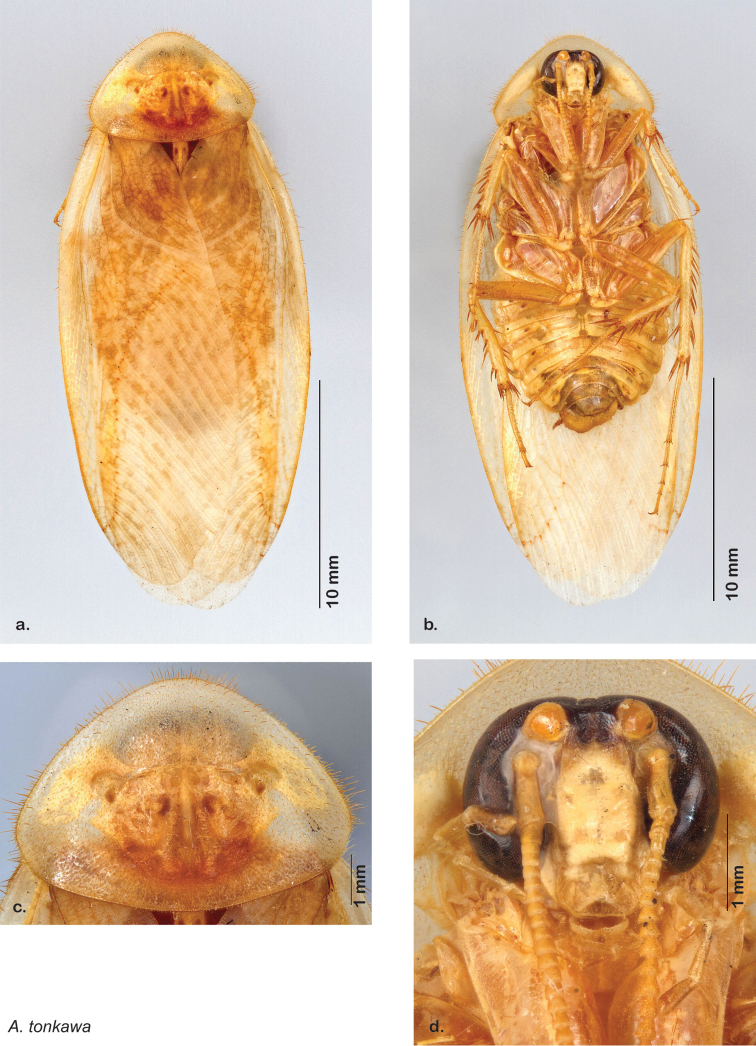
*Arenivaga tonkawa* **a** dorsal habitus **b** ventral habitus **c** pronotum **d** head.

*Head*. Two ocelli very large, ovoid and protruding (0.50 × 0.40 mm); vertex dark brown, with small ridges between apices of eyes extending on to ocellar tubercles; interocellar space concave, dark brown grading to medium brown medially; two oval indentations laterally at base of interocellar space. Frons light brown, concave; bound on either side by ridges extending from inner apex of ocelli outwards to lateral edges of clypeus; scattered long setae on ridges. Anterior portion of frons light brown, bulbous; clypeal suture demarcates light brown anteclypeus. See Fig. 147d.

*Pronotum*. Pronotum translucent waxy beige; variable length orange-brown setae along anterior margin; dorsal surface of pronotum covered with short orange-brown setae that are denser and longer anteriorly and laterally; pronotal pattern medium orange-brown “panther face”, with little detail and no aura; within the species pronotal pattern runs from light brown through every shade to dark brown, some with considerable discernible detail, but always with no aura. See Fig. 147c.

*Body*. Wing brace present. Legs and body medium orange-brown; subgenital plate asymmetrical with posterior edge emarginated, rounded apices. See Fig. 147b.

*Forewings*. Wings extended beyond abdominal apex (up to ~35% of total wing length); light orange-brown with darker blotches; within the species color variable from light orange-brown, to dark orange-brown, always blotchy; surface opaque and matte. See Fig. 147a.

**Figure 148.**
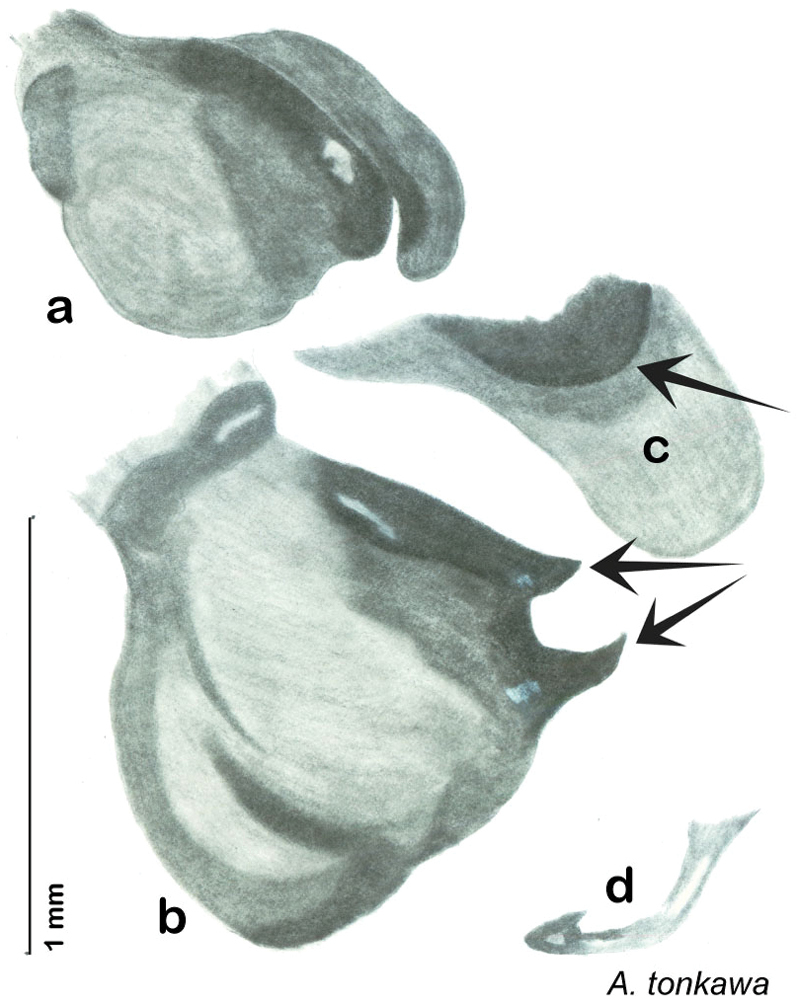
*Arenivaga tonkawa*, genitalia: a) right dorsal phallomere **b** right ventral phallomere **c** small central sclerite **d** genital hook. Arrow(s) indicate diagnostic characters (see text).

*Genitalia*. Right dorsal phallomere composed of bulbous lightly sclerotized lobe, articulated with right ventral phallomere on lateral side; central field lightly sclerotized; medial margin sclerotized, smooth, with two adjacent spines at posterior end; spines may vary in size and exact placement from specimen to specimen. Small central sclerite smooth, concave, with punctate, posteriorly pointing lip at anterior end. Right ventral phallomere extends from articulation into smooth lobe, punctate towards point of articulation; narrows anteriorly in punctate corrugations; after narrow gap, broad, punctate, posteriorly curving arm extending to depth of rest of phallomere. Genital hook with narrow pointed head and short hook; arm short with distinct bend. See Fig. 148.

**Figure 149.**
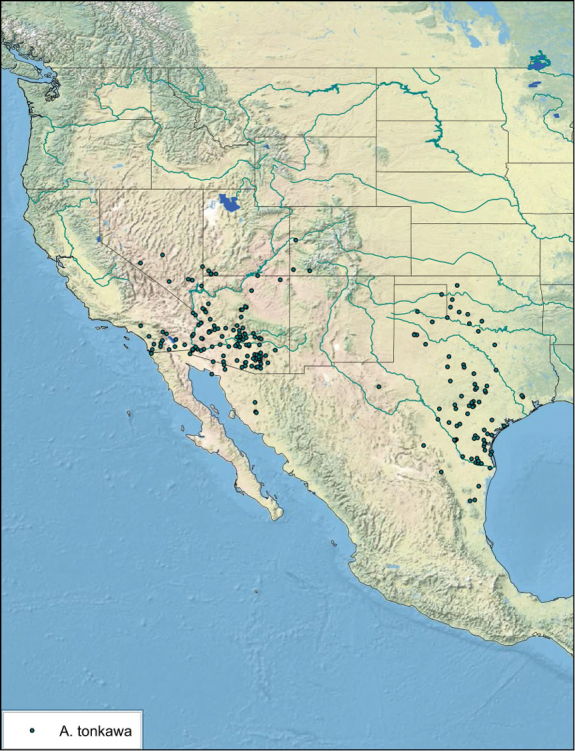
*Arenivaga tonkawa*, distribution.

##### Habitat and natural history.

All life history elements remain unobserved.

#### Arenivaga trypheros sp. n.

http://zoobank.org/66C1C0DB-931A-4E11-8D21-EB09F5F6A7E7

http://species-id.net/wiki/Arenivaga_trypheros

**Figure 150.**
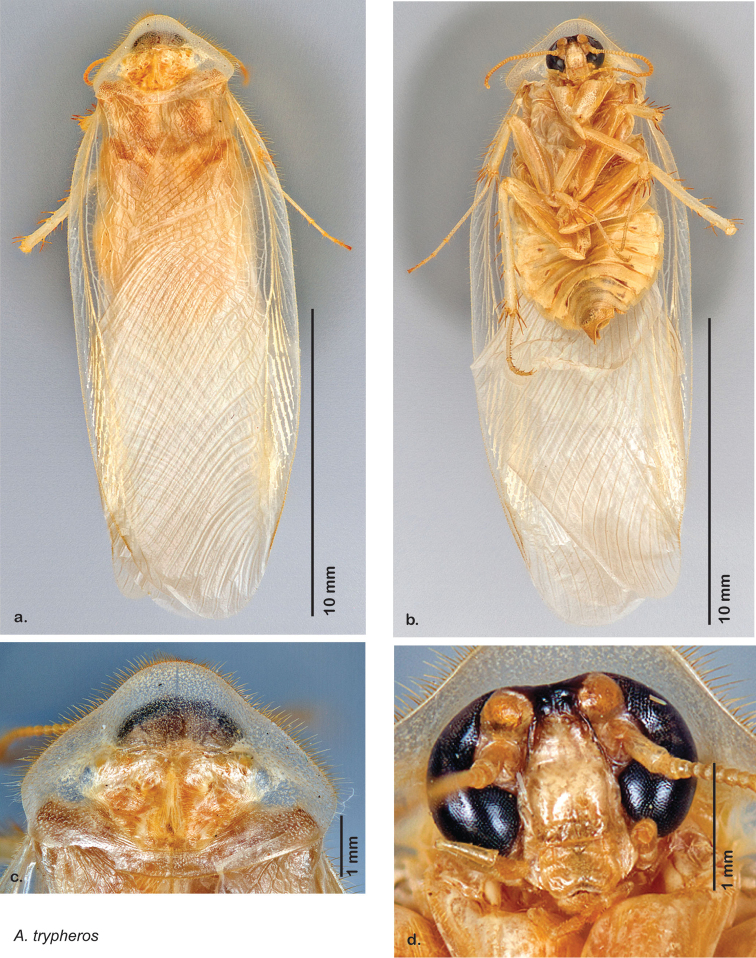
*Arenivaga trypheros* **a** dorsal habitus **b** ventral habitus **c** pronotum **d** head.

**Figure 151.**
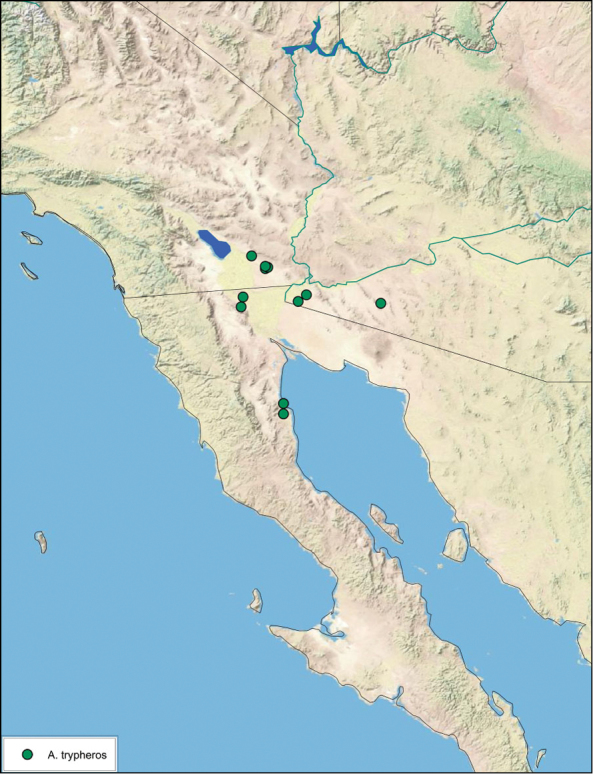
*Arenivaga trypheros*, distribution.

Figures 37 , 150 –151

##### Type locality.

USA, California, Imperial County, 1 mi. S of Glamis.

##### Material examined.

Holotype: ♂ in EMEC labeled “CAL: Imperial Co., 1 mi. S Glamis, 31-III-1978, J. Powell, coll., in pitfall trap” “HOLOTYPE *Arenivaga trypheros* Hopkins, 2012” [red label with black border].

Paratypes (18): USA: CA, Imperial Co., 17 mi. NW of Glamis, 6/27/1978, D & J Powell, blacklight trap (6, EMEC); CA, Imperial Co., 3 mi. SW of Glamis, 7/12/1974, J Doyen, blacklight trap (2, EMEC); CA, Imperial Co., 2 mi. W of Glamis, 6/2/1971, AJ Gilbert, pit trap,71F7-28 (1, CSCA); AZ, Yuma Co., Goldwater Military Range, San Cristoball Dunes, 3/4/1997, CA Olson, Pitfall traps (2, UAIC); AZ, Yuma Co., Yuma desert 9 mi. E of San Luis, 3/16/1980, Werner,Olson,Metz & MacLachlan (1, UAIC); AZ, Yuma Co., Large sand dunes SE of Yuma, 4/16/1994, 32.27N, 114.25W, WB Warner (1, WB Warner). MEXICO: BC, 10 mi. S of San Felipe, 3/25/1961, EL Sleeper (2, CAS); BC, Sierra Pinta Dunes, 6.5 mi. S of Mexicali, 4/?/1953, ER Tinkham, died 8/15/1953 (1, USNM); BC, San Felipe, 3/25/1963, GI Stage (1, HEH); BC, 3 mi. N of La Puerta, 4/20/1973, Chandler & Levin (1, UAIC). All paratypes labeled “Paratype *Arenivaga trypheros* Hopkins 2012” [blue label with black border].

##### Etymology.

The name is an adjective in the nominative singular. This species is named *trypheros*, Greek for “delicate, dainty, soft” because it is very delicate and dainty in its morphology.

##### Distribution.

This species is found in far southwestern Arizona, far southeastern California, northeastern Baja California, Mexico and a short distance down the western coast of the Sea of Cortez. See Fig. 151.

##### Diagnosis.

*Arenivaga trypheros* sp. n. is characterized by its long, narrow iridescent wings that extend a long distance beyond the end of the abdomen. The genitalia are indistinguishable from that of *Arenivaga darwini*, a sympatric species that is otherwise phenotypically quite different. See Fig. 37.

##### Description.

**Male.** *Measurements*. Holotype TL = 20.9 mm, GW = 7.4 mm, PW = 4.87 mm, PL = 3.57 mm, TL/GW = 2.82, PL/PW = 0.73. One of the longest and narrowest species of *Arenivaga*. EW = 0.35 mm; OW = 0.3 mm. The only notable difference in measurements among paratypes was in total length; the holotype total length is equal to the longest observed, the shortest TL = 16.7 mm.

*Head*. Two ocelli large, ovoid and strongly protruding (0.45 × 0.30 mm); vertex brown with small ridges in rays around upper apices of eyes and extending onto ocellar tubercles; interocellar space deeply concave, brown anteriorly fading to lighter brown towards frons. Posterior frons deeply concave, light brown; anterior frons bulbous and light brown; anteclypeus broad, flat, light brown. See Fig. 150d.

*Pronotum*. Pronotum pale, waxy beige-gold; anterior half of dorsal surface of pronotum covered in fine pale setae with scattering of thicker golden setae throughout; pronotal pattern ranges in color from same waxy beige-gold of background, to orange-brown, brown and very dark brown depending on specimen, “panther face” pattern, not well-defined or detailed in its presentation; no aura. See Fig. 150c.

*Body*. Wing brace present. Two tarsal claws present. Legs and body light waxy beige-gold, subgenital plate dramatically asymmetrical with strongly emarginated posterior edge and pointed apices. See Fig. 140b.

*Forewings*. Wings extended well beyond abdominal apex (> 50% of wing length); color varies from pale iridescent gold to iridescent grey-brown. See Fig. 150a.

*Genitalia*. Right dorsal phallomere composed of large bulbous lightly sclerotized hook-shaped lobe, articulated with right ventral phallomere on lateral side; unmodified. Small central sclerite consists solely of thin half circle of sclerotized material beginning near front of right ventral phallomere and sweeping around to rear of same phallomere; right ventral phallomere extends from articulation to form structure rounded at posterior apex and expanding to shagreened and more sclerotized area dorsally; attached anteriorly is U-shaped shagreened lobe bordered by rolled shagreened lip. Left phallomere unmodified. Genital hook with long extension to pointed head and short hook; arm with shallow curve. See Fig. 37.

##### Habitat and natural history.

This species occurs in sparsely vegetated sand dunes that are extremely dry and hot. All other life history elements remain unobserved.

#### Arenivaga umbratilis sp. n.

http://zoobank.org/E6D74644-4076-423E-9379-737C8B4C71F7

http://species-id.net/wiki/Arenivaga_umbratilis

Figures 152 – 154

##### Type locality.

USA, Arizona, Maricopa County, Phoenix.

##### Material examined.

Holotype: ♂ in ANSP labeled “Phoenix, X.9.03, Ariz. Kunze, Hebard Collection” “HOLOTYPE *Arenivaga umbratilis* Hopkins, 2012” [red label with black border].

Paratypes: None at this time.

##### Etymology.

This species is named from the Latin phrase meaning “in retirement”, as there is only one specimen of this species and it was collected in 1903. I strongly suspect this is either a hybrid, or an extinct species.

##### Distribution.

This species is known only from the type locality. See Fig. 154.

##### Diagnosis.

*Arenivaga umbratilis* has the external appearance of *Arenivaga tonkawa* but the genitalia of *Arenivaga pratchetti*. This species will be known when, upon genitalic dissection, a specimen has the genitalia of *Arenivaga pratchetti* but a subgenital plate with rounded apices. *Arenivaga pratchetti* has pointed apices on its subgenital plate (See Fig. 7). See Figs 153 and 127.

##### Description.

**Male.** *Measurements*. Holotype TL = 21.0 mm, GW = 10.0 mm, PW = 6.27 mm, PL = 4.58 mm, TL/GW = 2.10, PL/PW = 0.73. EW = 0.20 mm; OW = 0.50 mm.

**Figure 152.**
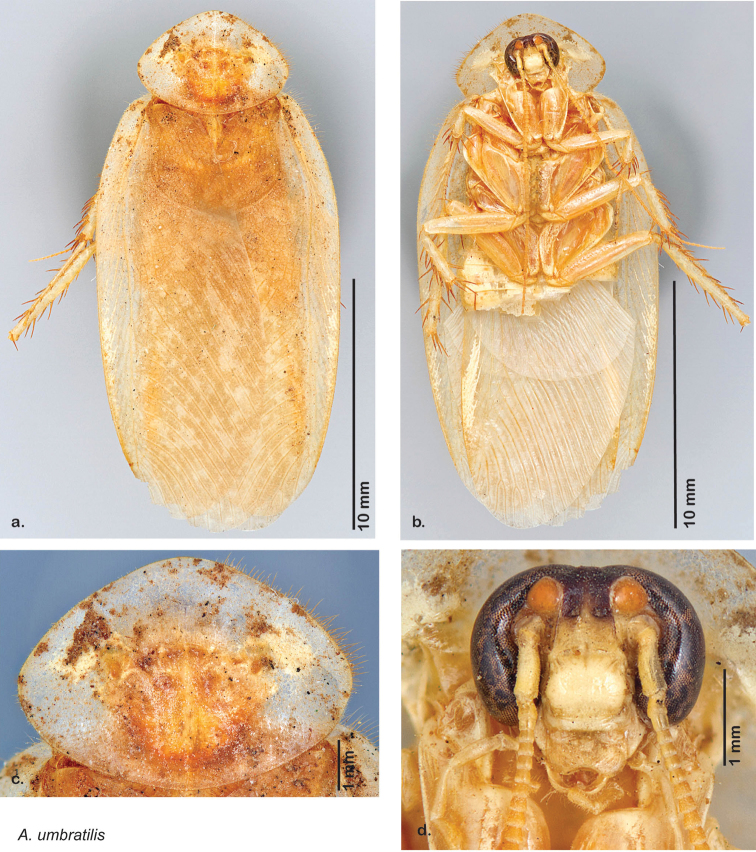
*Arenivaga umbratilis* **a** dorsal habitus **b** ventral habitus **c** pronotum **d** head.

*Head*. Two ocelli very large, ovoid and protruding (0.40 × 0.30 mm); vertex medium brown with small ridges in rays around upper apices of eyes and extending onto ocellar tubercles; interocellar space concave, smooth, medium brown, paler medially with two triangular shaped indentations. Frons very light brown fading to waxy white; posterior frons mildly concave, bound on either side by ridges extending from inner apex of ocelli outwards to lateral edges of clypeus; ridges with occasional long setae. Anterior portion of frons bulbous; clypeal suture demarcates waxy white smooth anteclypeus; no setae apparent. See Fig. 152d.

*Pronotum*. Pronotum translucent, waxy beige; variable length orange-brown setae along anterior margin; dorsal surface of pronotum thickly encrusted with sand and specimen too fragile to clean so surface setae undetectable; pronotal pattern light orange-brown to yellow “panther face” with no detail or aura. See Fig. 152c.

*Body*. Wing brace present. Legs and body light orange-brown. Subgenital plate dissected and cleared; asymmetrical with concave posterior edge and rounded apices. See Fig. 152b.

*Forewings*. Wings extended well beyond abdominal apex; light orange-brown with light brown blotches; surface matte and opaque. See Fig. 152a.

**Figure 153.**
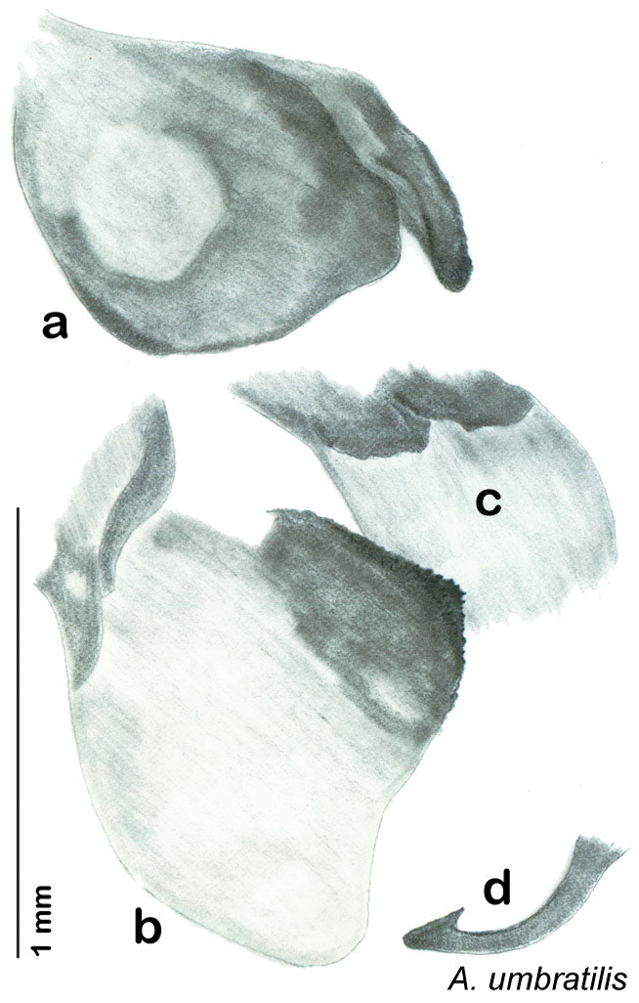
*Arenivaga umbratilis*, genitalia: a) right dorsal phallomere **b** right ventral phallomere **c** small central sclerite **d** genital hook.

*Genitalia*. Right dorsal phallomere composed of bulbous lightly sclerotized hook-shaped lobe, articulated with right ventral phallomere on lateral side; central field lightly sclerotized; medial margin more heavily sclerotized, shagreened with toothed edge and slight thickening centrally creating small bulge along rim. Small central sclerite concave, nondescript in shape, finely punctate with an irregular shagreened projection on internal ventral surface; right ventral phallomere extends from articulation to form smooth rounded structure becoming punctate and narrower anteriorly; attached anteriorly is slightly dorsally projecting flanged concave punctate arm that extends to depth of phallomere; shagreened edge. Folded anterior portion of left phallomere of moderate width, setose, otherwise unmodified. Genital hook with moderate extension to pointed head with short hook. See Fig. 153.

**Figure 154.**
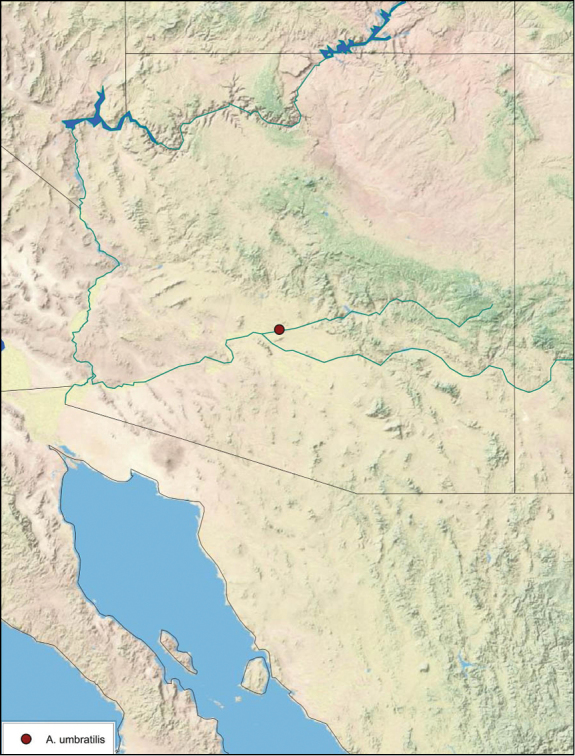
*Arenivaga umbratilis*, distribution.

##### Habitat and natural history.

All life history elements remain unobserved.

### Key to the males of *Arenivaga* (Rehn 1903)

NB: Either maps of the southwest and Mexico or Google Earth are required to use this key.

**Table:** 

1	Large (average 25 mm long × 12 mm wide, may be as large as 30 mm × 16 mm); generally medium (below left) to dark brown (below right); usually with no distinct pattern on pronotum	2
	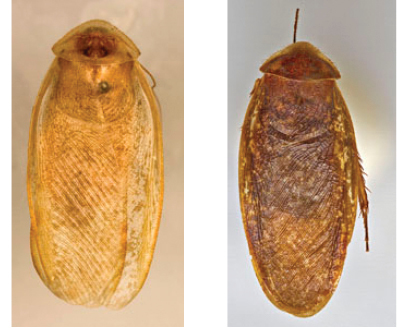	
1’	Smaller than 25 mm × 12 mm; any color from pale (below left) to dark brown (below right)	3
	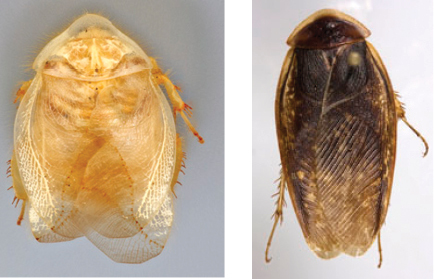	
2	Medial margin sclerotized but simple, with no complexity in the point of articulation between the two right phallomeres (below left); from Texas or portions of eastern Mexico adjacent to Texas (Fig. 35). (Specimens occasionally shorter and very dark brown; see below right.)	*Arenivaga bolliana* (Saussure 1893)
	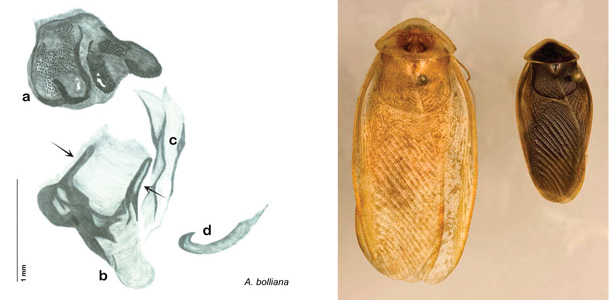	
2’	Hook-shaped lobe unique in shape, shagreened ridge running interior to the point of articulation on the right dorsal phallomere (below); from Arizona or western and central Mexico (Fig. 74)	*Arenivaga grata* Hebard 1920
	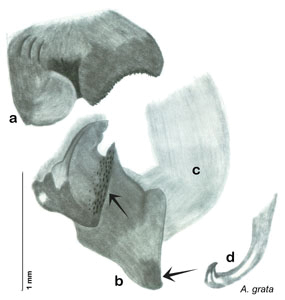	
3	Length < 20 mm; pumpkin seed shaped (below)	4
	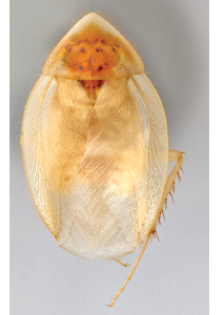	
3’	Length > 20 mm; not pumpkin seed shaped	7
4	Pale or very pale in color	5
4’	Brown in color	6
5	Very pale (below); one tarsal claw; southeastern California to southwestern Arizona and eastern Gulf of California (Fig. 38)	*Arenivaga darwini* sp. n.
	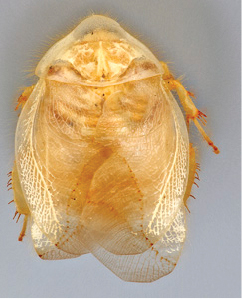	
5’	Pale; pronotum triangular (below); central Rio Grande in Texas (Fig. 137)	*Arenivaga ricei* sp. n.
	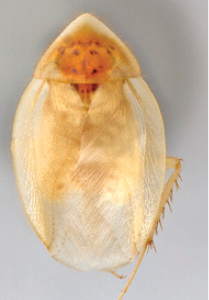	
6	Medium orange-brown or mottled medium orange-brown (below); Florida (Fig. 56)	*Arenivaga floridensis* Caudell 1918
	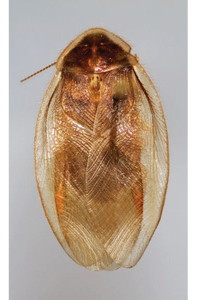	
6’	Concolorous medium brown; pronotum triangular with impressed dark brown pattern, densely setose (below); Baja California, Mexico (Fig. 20)	*Arenivaga alichenas* sp. n.
	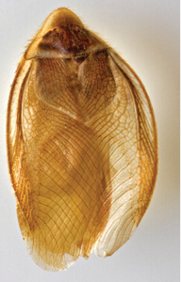	
7	Narrow and delicate (TL/GW > 2.0) (below)	8
	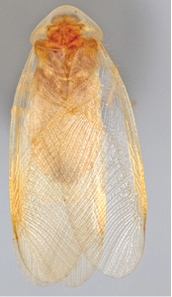	
7’	Not narrow and delicate (TL/GW <2.0)	11
8	Length > 17 mm	9
8’	Length < 17 mm	10
9	Very long and pale (TL/GW ~ 2.8) (below left); sharply pointed apices on subgenital plate (below right); southeastern California, southwestern Arizona, western side of Gulf of California (Fig. 151)	*Arenivaga trypheros* sp. n.
	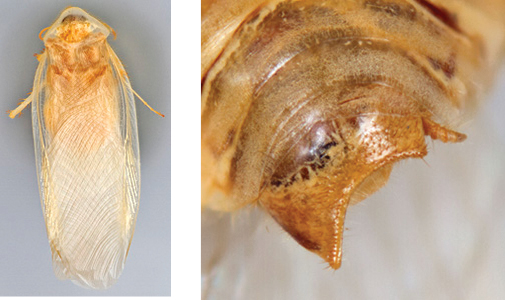	
9’	Not as long as in *trypheros* (above) (TL/GW ~ 2.2); rounded apices on subgenital plate (below left); small dorsal sclerite clamshell in shape with distinctive cross band of teeth (below right); only known from US (Fig. 41)	*Arenivaga delicata* sp. n.
	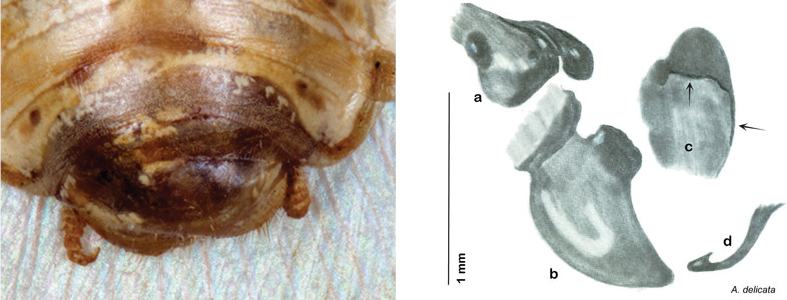	
10	Dark brown or dark orange-brown impressed pronotal pattern (below left); long serrated edge on medial margin of right dorsal phallomere, central field deeply incised (below right); central Baja peninsula (Fig. 44); often with no genicular spines	*Arenivaga diaphana* sp. n.
	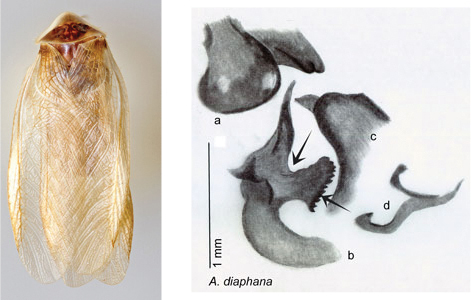	
10’	Slight and delicate; transparent wings and dark brown impressed pronotal pattern (below left); medial margin of right dorsal phallomere projects anteriorly into rounded, shagreened lobe with toothed margin (below right); Death Valley, California (Fig. 110)	*Arenivaga mortisvallisensis* sp. n.
	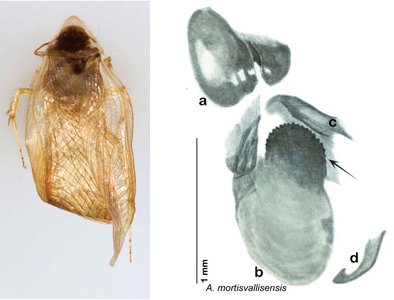	
11	From the Baja peninsula, Mexico or southwestern California	12
11’	Not from the Baja peninsula, Mexico or southwestern California	18
12	Very small (~14 mm × 7 mm) (below); Baja California Sur (Fig. 131)	*Arenivaga pumila* sp. n.
	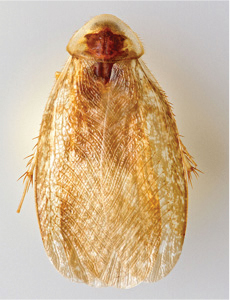	
12’	Larger than 14 mm × 7 mm	13
13	Setose pronotum; dark brown or dark red-brown pronotal pattern with no detail or aura (below); 7 miles SW of La Paz (Fig. 26)	*Arenivaga apaeninsula* sp. n.
	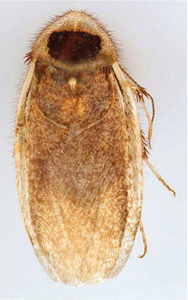	
13’	Not as in previous	14
14	Pronotal pattern light orange-brown with little detail (below left); narrow sweeping hook-shaped lobe on the right dorsal phallomere; broad short spine on medioventral side of posterior end of medial margin (below right); southeastern Baja California (outlier in Mexico may be mislabeled or transported) (Fig. 119)	*Arenivaga nocturna* sp. n.
	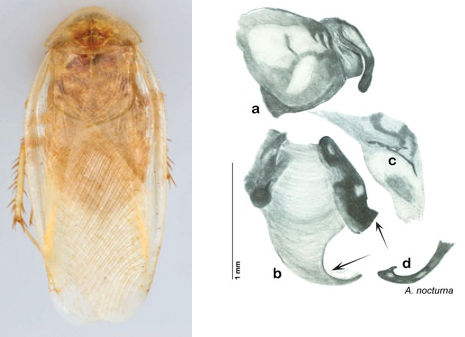	
14’	Pronotal pattern darker or more detailed than in previous	15
15	Right dorsal phallomere with long posterior projecting spine and second medially projecting spine located midway along medial margin; spine on right ventral phallomere (below); central Baja peninsula and adjacent islands (Fig. 116)	*Arenivaga nicklei* sp. n.
	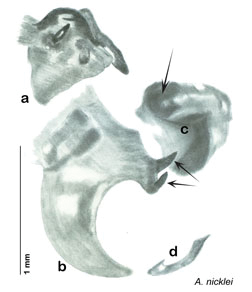	
15’	Genitalia with no spines on any phallomeres	16
16	Broad shagreened concavity interior to point of articulation on right dorsal phallomere; narrow central field, broad hook-shaped lobe with no curve; medial margin smooth (below); Baja peninsula (Fig. 134); may have one or both rudimentary styli	*Arenivaga rehni* Hebard 1917
	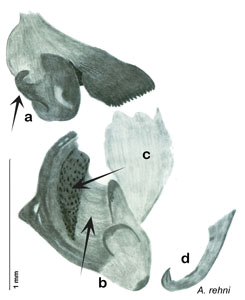	
16’	Right dorsal phallomere with serrate medial margin; left phallomere with setose, medially-projecting, scoop-shaped extension (two examples shown below)	17
	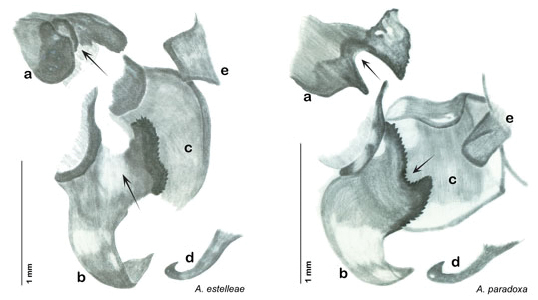	
17	Deeply sinuous, serrate medial margin (below); 15 miles south of San Quintin, Baja California (Fig. 125)	*Arenivaga paradoxa* sp. n.
	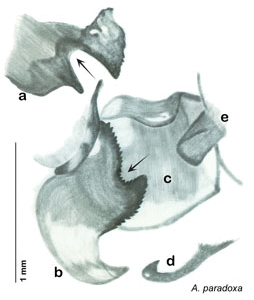	
17’	Medial margin with central indentation; broad central field (below); southwestern California and northern Baja California (Fig. 53)	*Arenivaga estelleae* sp. n.
	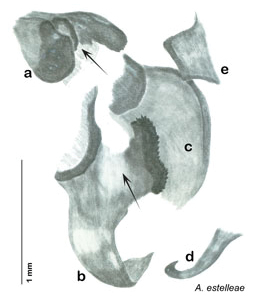	
18	Brown maculations scattered over pronotum (below)	19
	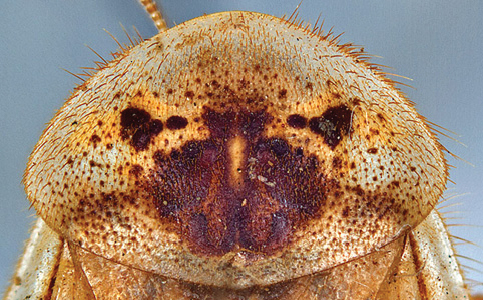	
18’	No maculations on pronotum	22
19	Long posteriorly projecting extension of medial margin ending in two-pronged hook (below); Texas or central Mexico (Fig. 77)	*Arenivaga gumperzae* sp. n.
	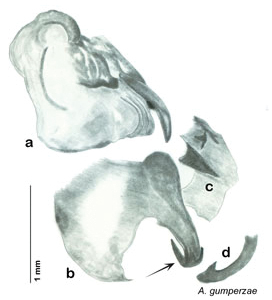	
19’	No projections from medial margin; tip of hook-shaped lobe turned dorsally; wide gap on right ventral phallomere (below); California	20
	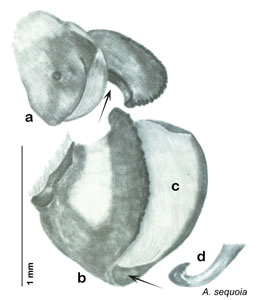	
20	Medial margin slightly convex (below); southern and western California (Fig. 143)	*Arenivaga sequoia* sp. n.
	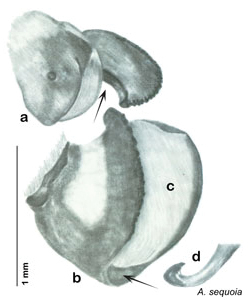	
20’	Medial margin not straight or convex	21
21	Medial margin shallowly sinuous (below); along the San Gabriel Mountains, southern California (Fig. 101)	*Arenivaga mckittrickae* sp. n.
	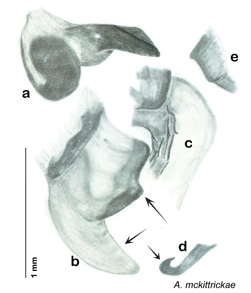	
21’	Medial margin with deep V-shaped emargination bordered by two broad flat points (below); in and around San Bernardino and Riverside, California (Fig. 62)	*Arenivaga gaiophanes* sp. n.
	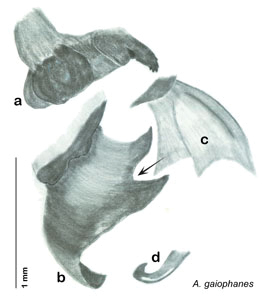	
22	From mainland Mexico or the Texas/Mexico border	23
22’	Not from mainland Mexico or the Texas/Mexico border	29
23	Forewings light to medium blotchy brown or blotchy orange-brown, with dark orange-brown or dark brown pronotal pattern (examples below)	24
	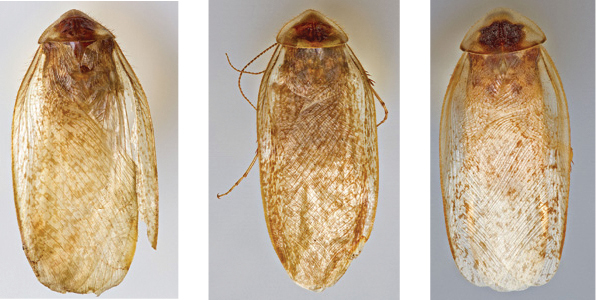	
23’	Not as in previous	26
24	Angular-headed genital hook (below)	25
	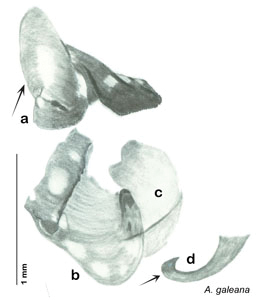	
24’	With widely curved genital hook and posteriorly directed laterally compressed bulge on the right ventral phallomere (below); southern foothills of San Madre Oriental Mountains, Hidalgo, Mexico (Fig. 59)	*Arenivaga florilega* sp. n.
	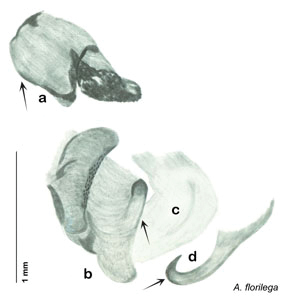	
25	Broad serrated arm extending anteriorly from right ventral phallomere (below); western foothills of the San Madre Oriental Mountains, Mexico (Fig. 65)	*Arenivaga galeana* sp. n.
	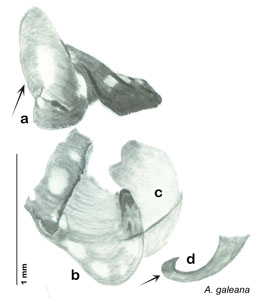	
25’	Narrow serrated arm extending anteriorly from right ventral phallomere; ridge of serration on lateral edge of open field (below); central Mexico to Texas border (Fig. 92)	*Arenivaga hypogaios* sp. n.
	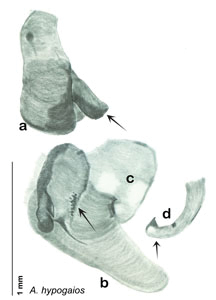	
26	Pale in color with light orange-brown pronotal pattern (below left); large gap in right ventral phallomere and long narrow pointed head on genital hook (below right); central Mexico to the Texas border (Fig. 140)	*Arenivaga rothi* sp. n.
	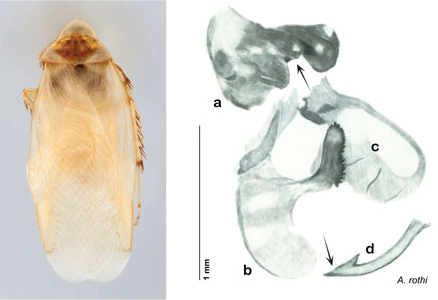	
26’	Medium orange-brown to very dark orange-brown with similarly colored pronotal pattern and aura (examples below)	27
	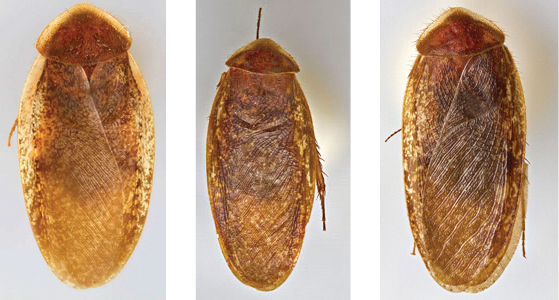	
27	Abbreviated hook-shaped lobe; unusual curvature and modeling of medial margin (below); Michoacan, Mexico (Fig. 80)	*Arenivaga gurneyi* sp. n.
	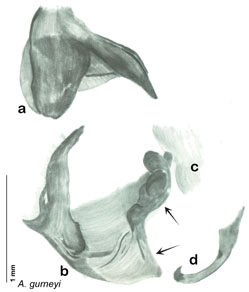	
27’	Hook-shaped lobe not abbreviated	28
28	Broad heavily serrated arm extending anteriorly from right ventral phallomere, with pronounced medial emargination (below); Puebla, Mexico (Fig. 47)	*Arenivaga dnopheros* sp. n.
	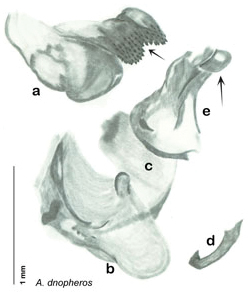	
28’	Arm extending from right ventral phallomere lightly serrated with no medial emargination; small ridge projecting from ventrolateral edge of left phallomere (below); Guerrero and Morelos, Mexico (Fig. 29)	*Arenivaga aquila* sp. n.
	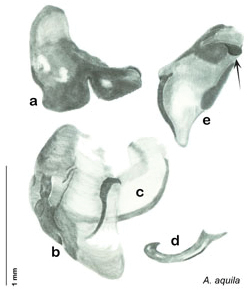	
29	Spines on at least two phallomeres (example below); Arizona, southwestern New Mexico, western mainland Mexico south of Arizona	30
	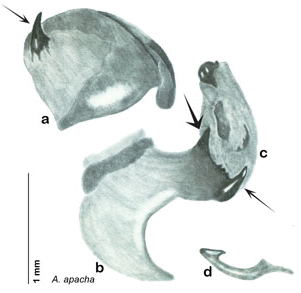	
29’	Spines on more or less than two phallomeres	36
30	Spines at either end of the medial margin (anterior spine may be small to absent); spine on right ventral phallomere; no spine on the left phallomere (below); Arizona (Fig. 23)	*Arenivaga apacha* (Saussure 1893)
	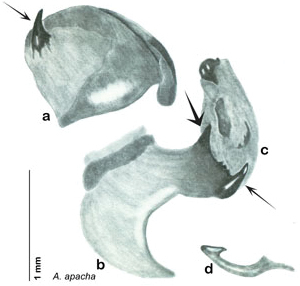	
30’	Spine on left phallomere	31
31	Three spines arrayed along the medial margin (anterior spine may be quite small); spine on right ventral phallomere (below); Sonora, Mexico (Fig. 17)	*Arenivaga akanthikos* sp. n.
	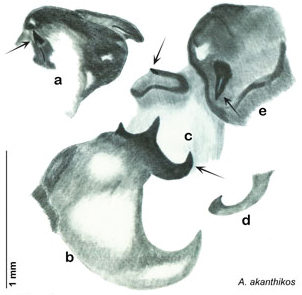	
31’	Without three spines arrayed along medial margin	32
32	Two spines along very contracted medial margin; spine on right ventral phallomere (below); southern Arizona, Sonora and Tiburon Island, Mexico (Fig. 89)	*Arenivaga hopkinsorum* sp. n.
	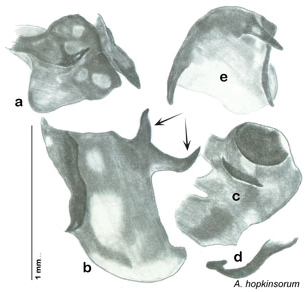	
32’	Without two spines along very contracted medial margin	33
33	Robust double spine at posterior end of medial margin; at least one posteriorly projecting spine on anterior edge of small central sclerite (below); southern Sonora, Mexico (Fig. 86)	*Arenivaga hebardi* sp. n.
	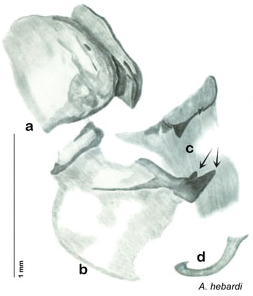	
33’	Without robust double spine at posterior end of medial margin or posteriorly projecting spine on anterior edge of small central sclerite	34
34	Right ventral phallomere with knob and three spines on dorsal surface; broadly sclerotized medial margin with two broad spines dorsally (below); southern Arizona and northern Sonora, Mexico (Fig. 68)	*Arenivaga genitalis* Caudell 1918
	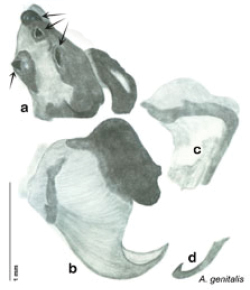	
34’	Without knob and three spines on dorsal surface of right ventral phallomere or broadly sclerotized medial margin with two broad spines dorsally	35
35	Large medially projecting spine on posterior end of medial margin; large setose spine on left phallomere; large bilobed bulge extending medially from small central sclerite; spine on right ventral phallomere (below); Sinaloa and Sonora, Mexico to the Arizona border (Fig. 14)	*Arenivaga adamsi* sp. n.
	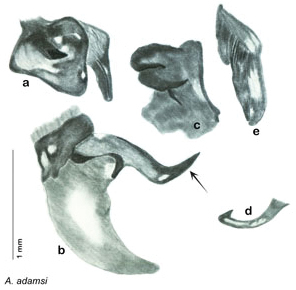	
35’	Large ventrally projecting curved spine on posterior end of medial margin; spine on left phallomere smaller than in previous and glabrous; bulge on small central sclerite single-lobed and smaller than in previous; double pronged spine on right ventral phallomere (below); Sierra de la Madera mountains, Sonora, Mexico (Fig. 107)	*Arenivaga moctezuma* sp. n.
	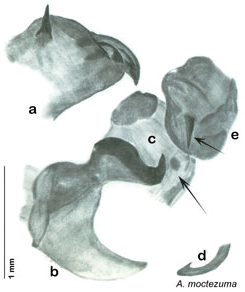	
36	From southwestern Arizona, New Mexico, or parts of Texas and Mexico south of New Mexico (Figs 50 and 146)	37
36’	Not from southwestern Arizona, New Mexico, or parts of Texas and Mexico south of New Mexico	38
37	Serrated posterior end to medial margin of right dorsal phallomere, and short spine projecting medially halfway along same margin; small central sclerite with sinuous line of teeth on lateral edge (below)	*Arenivaga tenax* sp. n.
	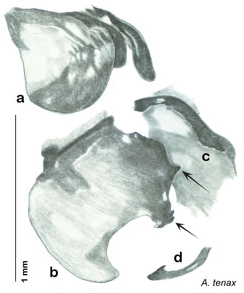	
37’	Straight shagreened medial margin, often two short teeth projecting medially a short distance from each end; small central sclerite with heavy shagreened rim along anterior edge (below)	*Arenivaga erratica* (Rehn 1903)
	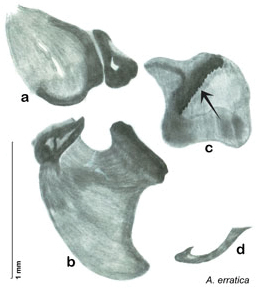	
38	From central or northwestern Arizona (isolated specimens from the Nevada/Arizona and Utah/Arizona borders	39
38’	Not from central or northwestern Arizona	43
39	Anteriorly directed shagreened tongue rising out of central field (below); along Colorado River at bottom of Grand Canyon (Fig. 71)	*Arenivaga grandiscanyonensis* sp. n.
	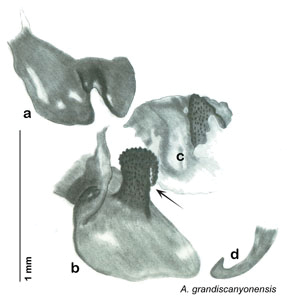	
39’	With no such modification to the central field	40
40	Medial margin bent at a 90 degree angle to the central field (below); along Colorado River at bottom of Grand Canyon and northwards along Utah and Nevada borders with Arizona (Fig. 122)	*Arenivaga pagana* sp. n.
	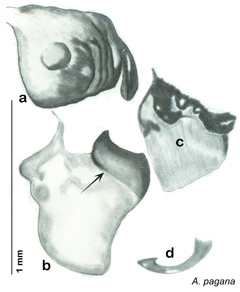	
40’	Without 90 degree bend in medial margin relative to central field	41
41	Larger than most species (~ 24 mm × 11 mm), generally light to medium brown in color; large shagreened medial margin, with toothed flange at posterior end and adjacent spine; delicate genital hook (below); northwestern Arizona south of the Colorado river (Fig. 95)	*Arenivaga impensa* sp. n.
	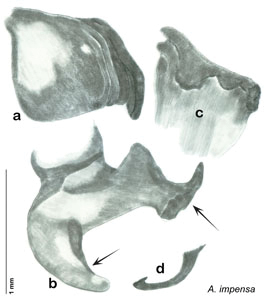	
41’	Not larger than average; without large shagreened medial margin	42
42	Light orange-brown; shagreened medial margin with slight central thickening creating small convexity (below); Gila River west of Phoenix, Arizona (Fig. 154)	*Arenivaga umbratilis* sp. n.
	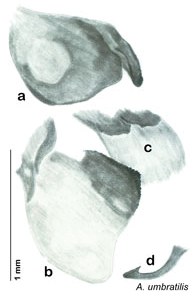	
42’	Medium to dark brown or red-brown with similarly colored pronotal pattern and aura; long shagreened convex medial margin that extends posteriorly beyond the rest of the phallomere (below); northwestern Arizona and southern Nevada (Fig. 83)	*Arenivaga haringtoni* sp. n.
	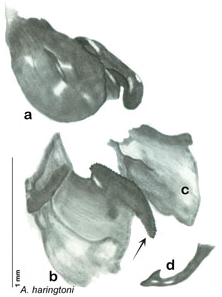	
43	From southeastern California, southern Nevada, southern Utah, along the western Arizona border or from northeastern Baja California, or northwestern Sonora, Mexico	44
43’	One spine at the posterior end and second spine medially on medial margin (below); disjunct distribution, one part being Arizona, southern California or western mainland Mexico south of Arizona (occasional specimens from Nevada, Utah and Colorado), the second part being central Texas, western Oklahoma, or eastern Mexico south of Texas (Fig. 149)	*Arenivaga tonkawa* Hebard 1920
	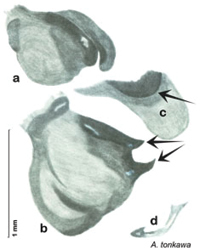	
44	Pale, though pronotal pattern may range from yellow to orange-brown to dark orange-brown; strongly asymmetrical subgenital plate with pointed apices (below left); internal genitalia the same as *umbratilis* (below right)	*Arenivaga pratchetti* sp. n.
	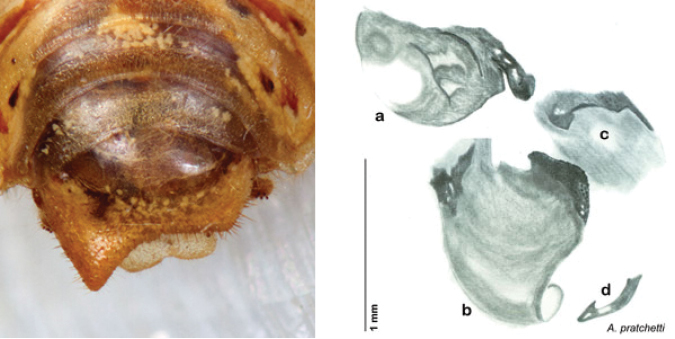	
44’	Rounded apices on subgenital plate	45
45	Spine extending from lobe of right ventral phallomere (below); southeastern California into southern Nevada, northern Mexico and western Arizona (Fig. 98)	*Arenivaga investigata* Friauf and Edney 1969
	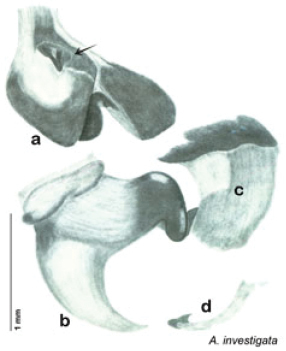	
45’	No spine on right ventral phallomere	46
46	Shagreened medial margin that extends beyond rest of phallomere at each end; small central sclerite with posteriorly projecting heavily toothed flanges dorsally and ventrally (below); central Nevada/California border (California coastal record may be label error or transported specimen) (Fig. 104)	*Arenivaga milleri* sp. n.
	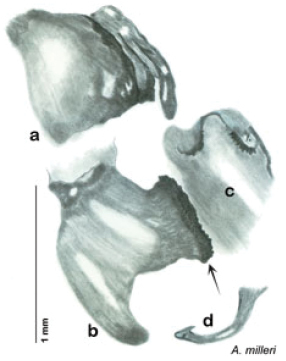	
46’	Medially projecting spine interior to posterior end of medial margin	47
47	Hook-shaped lobe bulbous with no bend; lobe portion of right ventral phallomere somewhat narrowed and lengthened (below); southern California, with isolated records in Mexico, Arizona, and Nevada (Fig. 113)	*Arenivaga nalepae* sp. n.
	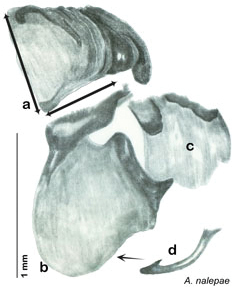	
47’	Hook-shaped lobe with indentation along medial edge; lobe portion of right ventral phallomere stout and short (below); southern Utah, southern Nevada into eastern California (Fig. 32)	*Arenivaga belli* sp. n.
	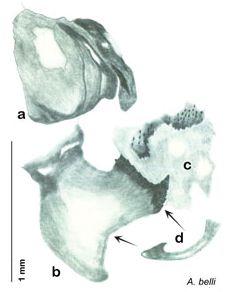	

## Discussion

Hebard was not exaggerating when he stated that this was the most difficult genus of cockroaches found in this country (Hebard 1920). This is now the most speciose genus of cockroaches in the United States with 48 species (39 of them new). An additional two new genera were also discovered (to be described separately). It seems likely, though, that my efforts here only scratch the surface of the mysteries surrounding the group. In particular, few specimens from Mexico were examined, and it seems likely that many new species remain to be found there. Additional collecting in the United States, particularly northern Arizona, southern Utah, and southern Colorado, will likely produce additional new species.

There are many intriguing puzzles within *Arenivaga* that cannot be fully resolved at this time nor by a species level evolutionary biologist such as me. For example, there are several groups of *Arenivaga* that occur along genitalic morphoclines. *Arenivaga belli* (eastern distribution), *Arenivaga milleri* (northern distribution) and *Arenivaga nalepae* (western distribution) have very similar genitalia and parapatric distributions. While I have described these as separate species, a population level study could reveal an argument that they are in fact one. The same can be said for the two species *Arenivaga aquila* (western distribution) and *Arenivaga dnopheros* (eastern distribution); for the group *Arenivaga florilega* (southern distribution), *Arenivaga galeana* (high elevation/eastern distribution), and *Arenivaga hypogaios* (western distribution); for the group *Arenivaga paradoxa* (southern distribution), *Arenivaga estelleae* (central distribution), and *Arenivaga gaiophanes* (northern distribution); and finally for the two species *Arenivaga adamsi* (western distribution) and *Arenivaga moctezuma* (eastern/valley isolate distribution). *Arenivaga* provides a rich field of study for a population geneticist with the funding, time, and interest in what drives populations towards speciation.

On the other hand, there are cases of possible hybrid specimens. The description of *Arenivaga umbratilis* is based on one specimen. This specimen is externally similar to *Arenivaga tonkawa* but has genitalia similar to *Arenivaga pratchetti* and is located geographically between the ranges of these two species. Given the available specimens and data, I recognize this as a species, but additional specimens will be needed to better assess species limits in these taxa. Based upon intermediate characteristics, *Arenivaga moctezuma* could also be a hybrid between sympatric species *Arenivaga adamsi* and *Arenivaga hopkinsorum*. Finally, and perhaps most curious of all, are *Arenivaga darwini* and *Arenivaga trypheros* that may represent two morphs of a species since they have very different external morphology, occur sympatrically, and have identical male genitalia. In this work I can only acknowledge these issues in species delimitation within *Arenivaga*; I must leave the search for answers to future workers.

## Supplementary Material

XML Treatment for Arenivaga

XML Treatment for Arenivaga adamsi

XML Treatment for Arenivaga akanthikos

XML Treatment for Arenivaga alichenas

XML Treatment for Arenivaga apacha

XML Treatment for Arenivaga apaeninsula

XML Treatment for Arenivaga aquila

XML Treatment for Arenivaga belli

XML Treatment for Arenivaga bolliana

XML Treatment for Arenivaga darwini

XML Treatment for Arenivaga delicata

XML Treatment for Arenivaga diaphana

XML Treatment for Arenivaga dnopheros

XML Treatment for Arenivaga erratica

XML Treatment for Arenivaga estelleae

XML Treatment for Arenivaga floridensis

XML Treatment for Arenivaga florilega

XML Treatment for Arenivaga gaiophanes

XML Treatment for Arenivaga galeana

XML Treatment for Arenivaga genitalis

XML Treatment for Arenivaga grandiscanyonensis

XML Treatment for Arenivaga grata

XML Treatment for Arenivaga gumperzae

XML Treatment for Arenivaga gurneyi

XML Treatment for Arenivaga haringtoni

XML Treatment for Arenivaga hebardi

XML Treatment for Arenivaga hopkinsorum

XML Treatment for Arenivaga hypogaios

XML Treatment for Arenivaga impensa

XML Treatment for Arenivaga investigata

XML Treatment for Arenivaga mckittrickae

XML Treatment for Arenivaga milleri

XML Treatment for Arenivaga moctezuma

XML Treatment for Arenivaga mortisvallisensis

XML Treatment for Arenivaga nalepae

XML Treatment for Arenivaga nicklei

XML Treatment for Arenivaga nocturna

XML Treatment for Arenivaga pagana

XML Treatment for Arenivaga paradoxa

XML Treatment for Arenivaga pratchetti

XML Treatment for Arenivaga pumila

XML Treatment for Arenivaga rehni

XML Treatment for Arenivaga ricei

XML Treatment for Arenivaga rothi

XML Treatment for Arenivaga sequoia

XML Treatment for Arenivaga tenax

XML Treatment for Arenivaga tonkawa

XML Treatment for Arenivaga trypheros

XML Treatment for Arenivaga umbratilis

## References

[B1] Appel AG Van Dyke AM Rust MK (1983) A Technique for Rearing and Some Notes on the Biology of a Desert Sand Cockroach *Arenivaga investigata* (Dictyoptera: Polyphagidae). Proceedings of the Entomological Society of Washington 85: 598-600.

[B2] Beccaloni GW Eggleton P (2011) Order Blattodea Brunner von Wattenwil, 1882. In: Zhang Z-Q (Ed) Animal biodiversity: An outline of higher-level classification and survey of taxonomic richness. Zootaxa 3148: 199–200. 10.11646/zootaxa.3703.1.1 26146682

[B3] Beccaloni GW Eggleton P (2013) Order Blattodea. In: Zhang Z-Q (Ed) Animal biodiversity: An outline of higher-level classification and survey of taxonomic richness (Addenda 2013) Zootaxa 3703: 46–48. 10.11646/zootaxa.3703.1.1 26146682

[B4] Caudell AN (1913) Notes on Nearctic Orthopterous Insects. I. Nonsaltatorial Forms. Proceedings of U.S. National Museum 44: 595-614.

[B5] Caudell AN (1918) Two New Species of the Blattid Genus *Arenivaga* (Orth.). Proceedings of the Entomological Society of Washington 20: 154-157.

[B6] Cohen AC Cohen JL (1976) Nest Structure and Micro-climate of the Desert Cockroach, *Arenivaga apacha* (Polyphagidae, Dictyoptera). Bulletin of the Southern California Academy of Sciences 75: 273-277.

[B7] Cohen AC Cohen JL (1981) Microclimate, Temperature and Water Relations of Two Species of Desert Cockroaches. Comparative Biochemical Physiology 69A: 165–167. doi: 10.1016/0300-9629(81)90656-3

[B8] Djernaes M Klass K-D Picker MD Damgaard J (2012) Phylogeny of cockroaches (Insecta, Dictyoptera, Blattodea), with placement of aberrant taxa and exploration of out-group sampling. Systematic Entomology 37: 65-83. doi: 10.1111/j.1365-3113.2011.00598.x

[B9] Edney EB (1968) The effect of water loss on the Haemolymph of *Arenivaga* sp. and *Periplaneta* Americana. Comparative Biochemical Physiology 25: 149-158. doi: 10.1016/0010-406X(68)90921-3 5657192

[B10] Edney EB Franco P Wood R (1978) The Reponses of *Arenivaga investigata* (Dictyoptera) to Gradients of Temperature and Humidity in Sand Studies by Tagging with Technetium 99m. Physiological Zoology 51: 241-255.

[B11] Edney EB Haynes S Gibo D (1974) Distribution and Activity of the Desert Cockroach *Arenivaga investigata* (Polyphagidae) in Relation to Microclimate. Ecology 55: 420-427. doi: 10.2307/1935230

[B12] Edney EB McFarlane J (1974) The Effect of Temperature on Transpiration in the Desert Burrowing Cockroach, *Arenivaga investigata* and in *Periplaneta americana*. Physiological Zoology 47: 1-12.

[B13] Friauf JJ Edney EB (1969) A New Species of *Arenivaga* from Desert Sand Dunes in Southern California (Dictyoptera: Polyphagidae). Proceedings of the Entomological Society of Washington 71: 1-7.

[B14] Grandcolas P (1996) A phylogeny of cockroach families: a cladistics appraisal of morpho-anatomical data. Canadian Journal of Zoology 74: 508-527. doi: 10.1139/z96-059

[B15] Hartman HB Bennett LP Moulton BA (1987) Anatomy of Equilibrium Receptors and Cerci of the Burrowing Desert Cockroach *Arenivaga* (Insecta, Blattodea). Zoomorphology 107: 81–87. doi: 10.1007/BF00312117

[B16] Hawke SE Farley RD (1971a) Antennal Chemoreceptors of the Desert Burrowing Cockroach, *Arenivaga* sp. Tissue and Cell 3: 649-664. doi: 10.1016/S0040-8166(71)80011-3 18631579

[B17] Hawke SD Farley RD (1971b) The Role of Pore Structures in the Selective Permeability of Antennal Sensilla of the Desert Burrowing Cockroach, *Arenivaga* sp. Tissue and Cell 3: 665–674. doi: 10.1016/S0040-8166(71)80012-5 18631580

[B18] Hawke SD Farley RD (1973) Ecology and Behavior of the Desert Burrowing Cockroach, *Arenivaga* sp. (Dictyoptera, Polyphagidae). Oecologia 11: 263-279. doi: 10.1007/BF01882784 28307166

[B19] Hebard M (1917) The Blattidae of North America North of the Mexican Boundary. Memoirs of the American Entomological Society 2: 1-284.

[B20] Hebard M (1920) Revisionary Studies in the Genus *Arenivaga* (Orthoptera, Blattidae, Polyphaginae). Transactions of the American Entomological Society 46: 197-217.

[B21] Jackson LL (1983) Epicuticular Lipid Composition of the Sand Cockroach, *Arenivaga investigata*. Comparative Biochemical Physiology 74B: 225–257.

[B22] Kirby WF (1904) A Synonymic Catalogue of Orthoptera, Vol. 1 Longman’s & Co., London, 310 pp.

[B23] Klass K-D (1997) The External Male Genitalia and the Phylogeny of Blattaria and Mantodea. Bonner Zoologische Monographien Nr. 42. Zoologisches Forschungsinstitut und Museum Alexander Koenig, Bonn, 341 pp.

[B24] McKittrick FA (1964) Evolutionary Studies of Cockroaches. Memoirs of the NY Argricultural Experimental Station No. 389, Ithaca.

[B25] O’Donnell MJ (1977) Site of Water Vapour Absorption in the Desert Cockroach, *Arenivaga investigata*. Proceedings of the National Academy of Sciences of the USA 4: 1757-1760. doi: 10.1073/pnas.74.4.1757 PMC430873 266217

[B26] O’Donnell MJ (1981) Fluid Movements During Water-Vapor Absorption by the Desert Burrowing Cockroach, *Arenivaga investigata*. Journal of Insect Physiology 27: 877-887. doi: 10.1016/0022-1910(81)90089-5

[B27] O’Donnell MJ (1982) Hydrophilic Cuticle–The Basis for Water Vapour Absorption by the Desert Burrowing Cockroach, *Arenivaga investigata*. Journal of Experimental Biology 99: 43–60.

[B28] Rehn JAG (1903) A Revision of the Orthopterous Genus *Homoeogamia*. Proceedings of the Academy of Natural Sciences of Philadelphia 55: 177-192.

[B29] Saussure H de (1893) Revue Suisse de Zoologie et Annals du Musee D'Histoire Naturelle de Geneve, Tome I Aubert-Schuchardt, Geneva.

[B30] Saussure H de Zehntner L (1894) Biologia Centrali-Americana, Insecta, Orthoptera, Volume I [Published for the editors by R.H. Porter, London]: 1893–1899.

[B31] Walthall WW Hartman HB (1981) Receptors and Giant Interneurons Signaling Gravity Orientation Information in the Cockroach *Arenivaga*. Journal of Comparative Physiology A 142: 359-369. doi: 10.1007/BF00605448

